# Antennal and abdominal transcriptomes reveal chemosensory gene families in the coconut hispine beetle, *Brontispa longissima*

**DOI:** 10.1038/s41598-017-03263-1

**Published:** 2017-06-05

**Authors:** Shu-Ying Bin, Meng-Qiu Qu, Ke-Ming Li, Zheng-Qiang Peng, Zhong-Zhen Wu, Jin-Tian Lin

**Affiliations:** 1 Institute for Management of Invasive Alien Species, 314 Yingdong teaching building, Zhongkai University of Agriculture and Engineering, Guangzhou, 510225 PR China; 2 Institute of Banana and Plantain, Chinese Academy of Tropical Agricultural Sciences, Haikou, 570102 PR China; 3 Institute of Environment and Plant Protection, Chinese Academy of Tropical Agricultural Sciences, Haikou, 570101 PR China

## Abstract

Antennal and abdominal transcriptomes of males and females of the coconut hispine beetle *Brontispa longissima* were sequenced to identify and compare the expression patterns of genes involved in odorant reception and detection. Representative proteins from the chemosensory gene families likely essential for insect olfaction were identified. These include 48 odorant receptors (ORs), 19 ionotropic receptors (IRs), 4 sensory neuron membrane proteins (SNMPs), 34 odorant binding proteins (OBPs) and 16 chemosensory proteins (CSPs). Phylogenetic analysis revealed the evolutionary relationship of these proteins with homologs from Coleopterans or other insects, and led to the identification of putative aggregation pheromone receptors in *B. longissima*. Comparative expression analysis performed by calculating FPKM values were also validated using quantitative real time-PCR (qPCR). The results revealed that all ORs and antennal IRs, two IR co-receptors (BlonIR8a and BlonIR25a) and one SNMP (BlonSNMP1a) were predominantly expressed in antennae when compared to abdomens, and approximately half of the OBPs (19) and CSPs (7) were enriched in antennae. These findings for the first time reveal the identification of key molecular components in *B. longissima* olfaction and provide a valuable resource for future functional analyses of olfaction, and identification of potential targets to control this quarantine pest.

## Introduction

Insects are well-known for their sophisticated olfactory systems, which responds to a diverse array of environmental chemical signals to evaluate and locate food, shelter, mates and oviposition sites as well as to avoid predators or other dangers. Olfaction in insects is mediated via major peripheral olfactory proteins including odorant receptors (ORs), ionotropic receptors (IRs), sensory neuron membrane proteins (SNMPs), odorant binding proteins (OBPs) and chemosensory proteins (CSPs)^1^. Odorant binding to two major chemoreceptor families, ORs and IRs, located in the dendritic membrane of ORNs activates them thus generating an electrical signal that is processed and transmitted to higher-order centers^1–3^. ORs are multi-transmembrane domain receptors and are composed of heteromeric complexes with two subunits, a highly conserved protein acting as an ion channel across insect orders (the OR co-receptor (Or83b), ORco)^4^, and a variable partner (ORX) that determines ligand specificity^5, 6^. IRs are closely related to ionotropic glutamate receptors (iGluR), which encode ligand-gated cation channels with atypical binding domains that are conserved across protostomians and are proposed to be more ancient than ORs^7, 8^. SNMPs are members of the CD36 family with two transmembrane domains located in the dendritic membranes of pheromone sensitive neurons, and contribute to pheromone responses^9–11^. OBPs are soluble proteins that bind and carry hydrophobic odorants to the chemoreceptors in the dendrites of olfactory sensory neurons (ORNs) across the hydrophilic sensillar lymph^1^, and in one case it has been suggested that the OBP-odorant complex directly activates the receptor rather than odorant released from the OBP^12^. Another family of binding proteins, the CSP family, may have diverse functions as well^13^. In some insect species, antenna-specific or antenna predominant CSPs exhibit binding affinity towards odorants from hosts and pheromones^14–17^.

Transcriptomic analyses of chemosensory appendages have allowed the identification and characterization of chemosensory families in insect species without a reference genome^18–20^. Additionally, sex- and tissue-specific profiles of chemosensory gene expression as well as functional analyses of candidate chemosensory genes are the primary steps to investigate the molecular mechanisms underlying insect olfaction. Insect transcriptome analyses have benefited from RNA sequencing (RNA-seq) in recent years by saving time, reducing cost, and becoming highly efficient. RNA-seq has helped to understand olfactory proteins from various important agricultural pest species whose genomes have not been sequenced, providing essential information to develop successful pest control strategies.

The coconut hispine beetle, *Brontispa longissima* (Gestro) (Coleopteran: Chrysomelidae) is one of the most invasive and destructive pests of Palmae plants, harming nearly every palm species with primary damage to coconut palm (*Cocos nucifera*)^21^. Both larvae and adults feed on the youngest unopened leaves of coconut palms and cause leaf browning and eventual destruction of the plant. *B. longissima* is native to tropical areas such as Indonesia and Papua New Guinea. More recently, it has expanded to the Pacific, Southeast Asia and China. Large-scale outbreaks have resulted in unprecedented economic losses to both the coconut industry and the tropical tourism industry^22–25^. In alignment with other beetles, the behaviors of *B. longissima* are olfactory sense driven and olfaction is important for host tree selection, aggregation and colonization. Additionally, it has been reported that the ratios of key components of the coconut palm leaf volatiles elicit attraction, aggregation and oviposition by this beetle^26, 27^. Therefore, a better understanding of the molecular components involved in olfactory perception in this pest will increase opportunities to develop highly effective surveillance and control strategies against this beetle.

In the present study, using next-generation sequencing technology, we sequenced the transcriptomes from the main olfactory organs (the adult antennae) and non-olfactory organs (abdominal segments) of males and females of *B. longissimi*. Abdomen is involved in reproduction or potential sex pheromone production. This study resulted in the identification of 121 candidate chemosensory genes comprising 48 ORs, 19 IRs, 4 SNMPs, 34 OBPs and 16 CSPs. The phylogenetic relationships of *B. longissima* chemosensory genes were then analyzed in comparison with *Tribolium castaneum, D*. *melanogaster* and other coleopteran insects. Furthermore, differential expression analyses were performed based on RNA-seq data, which was validated by qPCR on approximately one third of the identified ORs, antennal IRs, all OBPs and CSPs.

## Results

### Summary of transcriptome assembly

To identify candidate chemosensory genes and compare their transcript abundance in the olfactory and non-olfactory organs of *B. longissima*, we constructed transcriptomes from the antennae and abdomens of male and female beetles via deep-sequencing. Raw data are submitted to the Sequence Read Archive (SRA) in NCBI (accession number SRP077039). After trimming adaptor sequences and eliminating low quality reads, Illumina sequencing generated a total of 47,162,716, 46,654,298, 42,811,068, and 47,324,228 clean reads with an average length of 150 bp, in the library made from female antennae (FA), 46,654,298 from male antennae (MA), 42,811,068 from female terminal abdomens (FAB) and 47,324,228 from male terminal abdomens (MAB). A combined transcriptomes assembly generated 42,724 unigenes, with a mean length of 1,592 bp and included 29,508 (FA), 28,215 (MA), 24,151 (FAB), and 27,948 (MAB), respectively (Supplementary Table S1). Information of the identified ORs, IRs, SNMPs, OBPs, CSPs, and endogenous reference genes including the gene name, unigene ID, unigene sequences, lengths, predicted proteins, the best BLASTx hits, predicted protein domains and transcript abundance are listed in Supplementary Data File.

### Genes involved in odorant reception

In this study, a total of 121 transcripts belonging to gene families putatively involved in odorant reception were identified. These included ORs (48 transcripts), IRs (19 transcripts), SNMPs (four transcripts), OBPs (34 transcripts) and CSPs (16 transcripts). A great majority of the candidate genes (105/121) identified here had full-length ORFs indicating the integrity of these transcriptomes. A list of these genes with details of genetic characteristics, nr annotation, predicted protein domains and transcript abundance can be found in Supplementary Data File.

#### ORs

**Figure 1:**
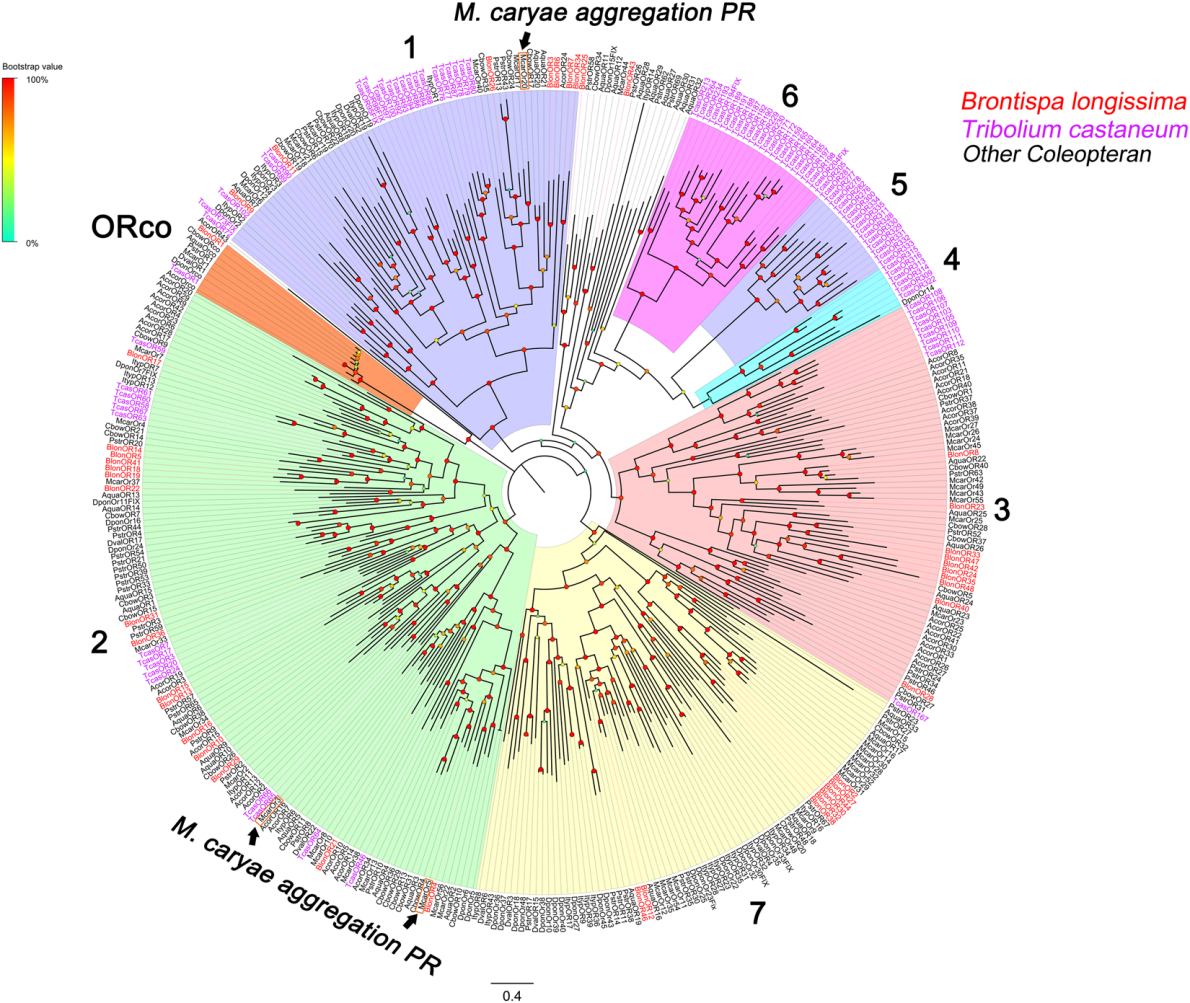
Phylogenetic tree of ORs from *B*. *longissima* (red labels), *T*. *castaneum* (purple labels) and other coleopterans (black labels). The tree was constructed using MEGA (v.6.0) with the LG + G substitution model and NNI topology search, based on an amino acid alignment by MAFFT. Branch support (circles at the branch nodes) was estimated using an approximate likelihood ratio test based on the scale indicated at the top left. Bars indicate branch lengths in proportion to amino acid substitutions per site. The *M*. *caryae* aggregation pheromone receptor (PR) is outlined in orange rectangles. OR prefixes indicate the following: Acor, *A*. *corpulenta*; Aqua, *A*. *quadriimpressum*; Blon, *B*. *longissima*; Cbow, *C*. *bowringi*; Dpon, *D*. *ponderosae*; Dval, *D*. *valens*; Ityp, *I*. *typographus*; Mcar, *M*. *caryae*; Pstr, *P*. *striolata*; Tcas, *T*. *castaneum*.

In the combined transcriptomes, 48 were identified as candidate ORs and matched the odorant receptor protein domain. Among these transcripts, 41 transcripts likely contain full-length ORFs encoding 314 to 477 amino acid residues with 5 to 7 transmembrane domains, which are typical for insect ORs. We identified one OR sequence (BlonOR1) that shared a high level of amino acid identity with the conserved ORco proteins from other insect species and named it BlonORco. Following phylogenetic analysis, ORs from several Coleopteran species were clustered into multiple subgroups (Fig. 1) and numbered from 1 to 7 based on previous studies^28, 29^. The BlonORs largely grouped into the previously defined coleopteran OR subgroups 1, 2, 3 and 7 as well as the ORco subgroup. We found that BlonOR4 and McarOR5, a functionally characterized gene from the cerambycid beetle, *Megacyllene caryae* ^30^, clustered in the same branches. (Fig. 1).

**Figure 2:**
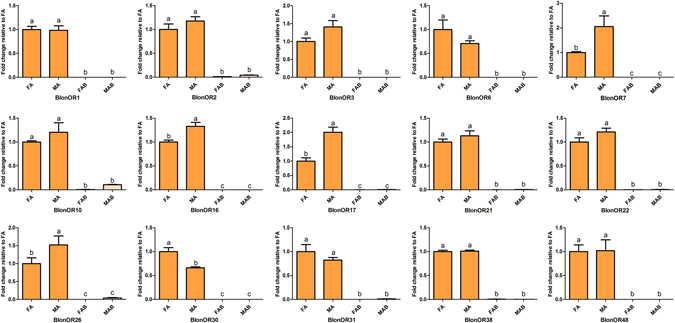
Relative expression levels of the top 15 overexpressed ORs using qPCR. FA, female antennae; MA, male antennae; FAB, female terminal abdomens; MAB, male terminal abdomens. The relative expression level is indicated as mean ± SE (N = 3). Different letters indicate significant differences between tissues (P < 0.05, ANOVA, LSD).

Our transcriptomic analyses showed that all OR genes were exclusively expressed in the antennae of both sexes, and almost no expression (FPKM value less than 1) was observed in the terminal abdomens (see Supplementary Figure S1). BlonORco had the highest level of expression in male and female antennae (FPKM value: approximately 300) followed by BlonOR30 (FPKM value: 228 and 173, respectively in female and male antennae). The remaining ORs showed relatively low level expression in the antennae of both sexes. The total number of most highly expressed OR genes in antennae was 15. qPCR analysis further confirmed their predominant expression in the male and female antennae (Fig. 2 and Supplementary Figure S1). Among these, BlonOR7, BlonOR16, BlonOR17 and BlonOR26 were significantly up-regulated in the male antennae while BlonOR30 was up-regulated in the female antennae (Fig. 2).

#### IRs

**Figure 3:**
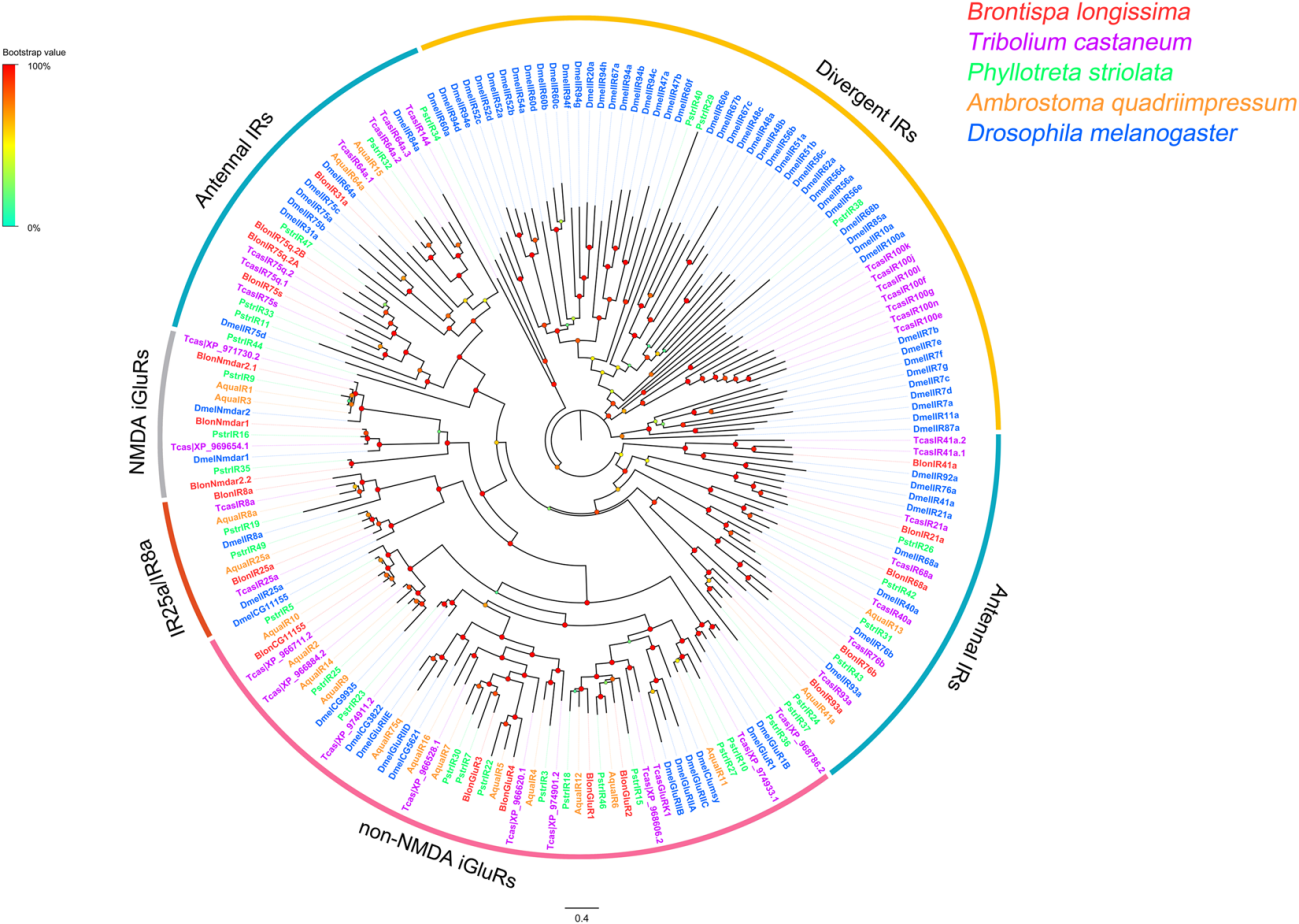
Phylogenetic tree of IRs from *B. longissima* (red labels), *T. castaneum* (purple labels) and other insects (*P*. *striolata*, green labels; *A*. *quadriimpressum*, orange labels; *Drosophila melanogaster*, blue labels). The tree was constructed using FastTree 2.1.7 with the JTT substitution model, based on an amino acid alignment by MAFFT. Branch support (circles at the branch nodes) was estimated using an approximate likelihood ratio test based on the scale indicated at the top left. Bars indicate branch lengths in proportion to amino acid substitutions per site. IR prefixes indicate the following: Aqua, *A*. *quadriimpressum*; Blon, *B*. *longissima*; Dmel, *D*. *melanogaster*; Tcas, *T*. *castaneum*.

In total, we identified 19 candidate iGluRs/IRs from the combined transcriptomes. These are predicted to encode ligand-gated cation channels (S1 and S2) with three transmembrane domains (M1, M2, and M3) or part of domains (Supplementary Figure S2). Among these, 14 iGluRs/IR transcripts likely contain full-length ORFs encoding more than 551 amino acid residues. All identified iGluRs/IRs in *B. longissima* clustered with their orthologs from *T. castaneum*, *D. melanogaster* and other coleopteran insects, and were assigned to four phylogenetic groups including N-Methyl-D-aspartic acid (NMDA) iGluRs, non-NMDA iGluRs, IR co-receptors (IR25a/IR8a) and antennal IRs. No member of the divergent IR clade was identified in the combined transcriptomes of *B. longissima*. Subsequently, the 17 *B. longissima* IRs/iGluRs were correspondingly named after their putative *T*. *castaneum* homologs (Fig. 3).

**Figure 4:**
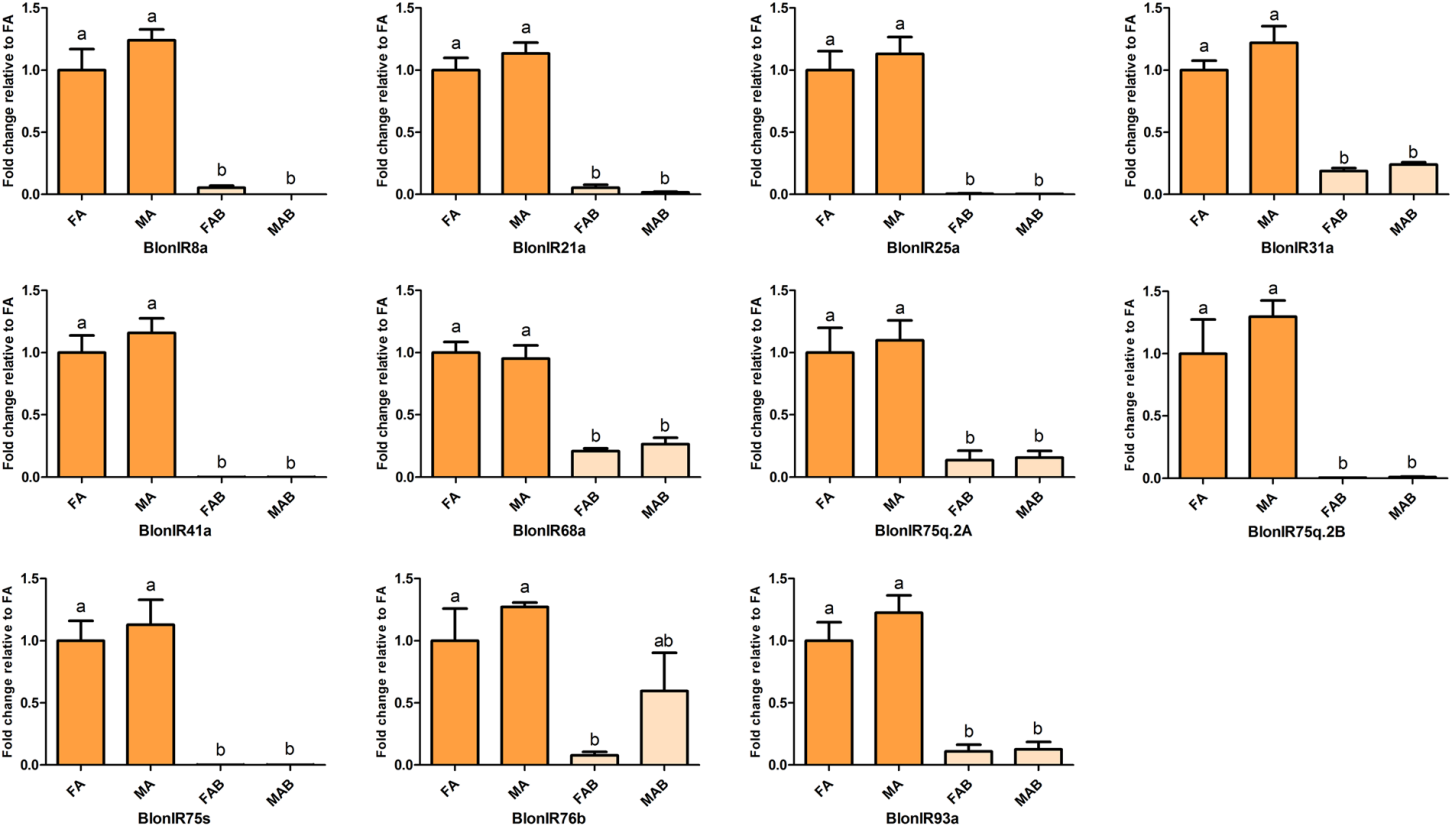
Relative expression levels of antennal IRs using qPCR. FA, female antennae; MA, male antennae; FAB, female terminal abdomens; MAB, male terminal abdomens. The relative expression level is indicated as mean ± SE (N = 3). Different letters indicate significant differences between tissues (P < 0.05, ANOVA, LSD).

All but one (BlonIR76b) of the antennal IRs and two IR co-receptors (BlonIR8a and BlonIR25a) were predominantly expressed in the antennae, whereas the non-NMDA iGluRs (BlonGluR1-4) showed up-regulated expression in the terminal abdomen. Besides, the co-receptor, BlonIR76b, was highly expressed in the antennae of both sexes and in the male terminal abdomens. The FPKM results showed that BlonIR25a displayed the highest expression level in the antennae of both sexes similar to other coleopteran homologs^31^ (see Supplementary Figure S3 **)**. Lastly, qPCR data showed that the expression pattern of the antennal IRs was enriched in antennae and mirrored the RNA-seq data, and sex-biased expression levels were not found (Fig. 4).

#### SNMPs

In the combined transcriptomes, four candidate SNMP transcripts were identified that matched members from the CD36 family. Among these, three (BlonSNMP1a, BlonSNMP1b and BlonSNMP2b) likely contain full-length ORFs. BLASTx results against the NCBI nr database indicated that BlonSNMPs shared a 41–61% amino acid sequence identity to their homologs from other coleopteran insects. Based on the phylogenetic analysis, we found that BlonSNMP1a and BlonSNMP1b clustered with homologous SNMP1 group from other insects. Similarly, BlonSNMP2a and BlonSNMP2b clustered with SNMP2 group homologs from other insects (see Supplementary Figure S4). The FPKM results revealed that BlonSNMP1a had the highest expression level in antennae. In contrast, BlonSNMP1b and BlonSNMP2b were expressed in smaller amounts in the antennae, while BlonSNMP2a was not detectable in the antennae (see Supplementary Figure S5).

#### OBPs

**Figure 5:**
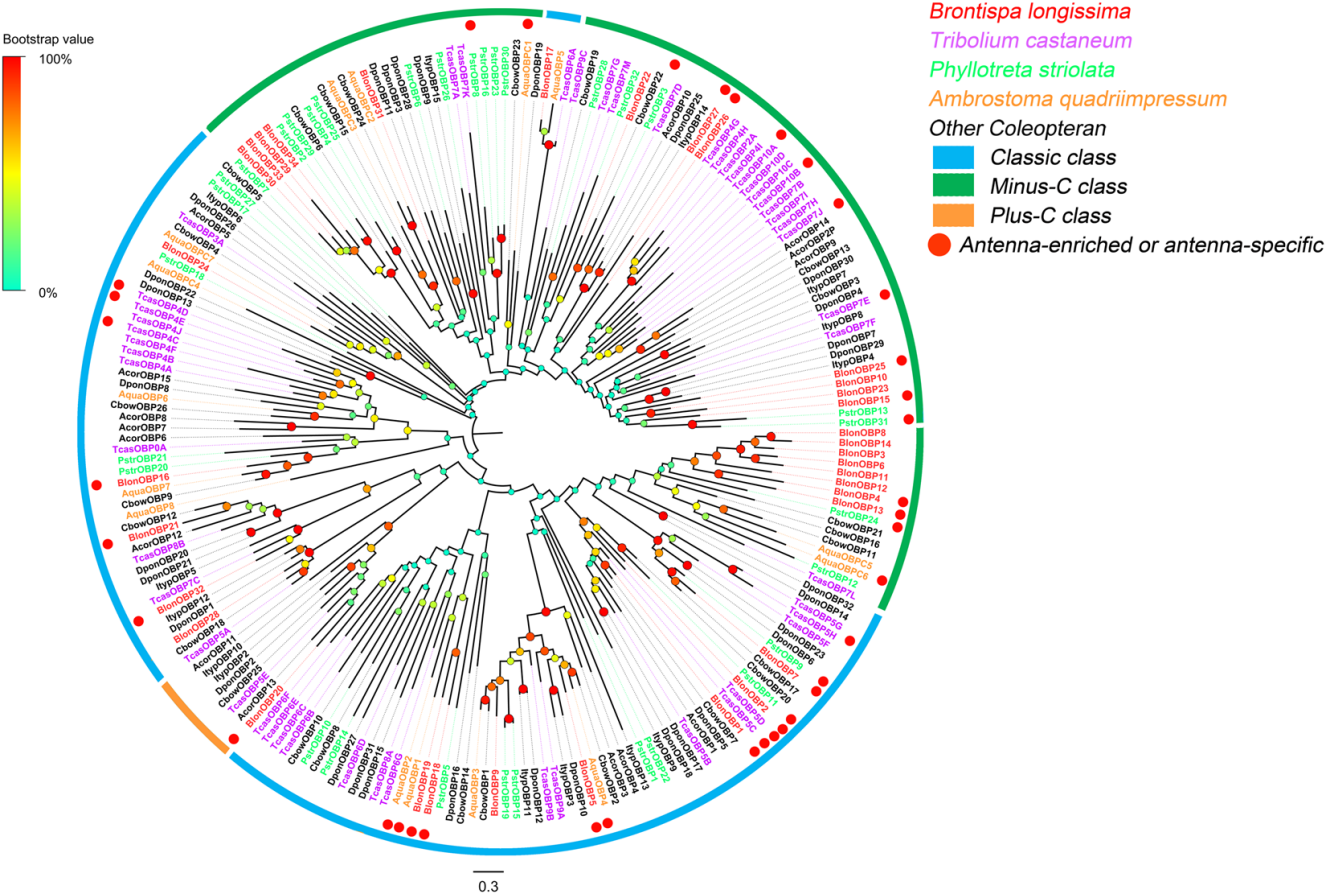
Phylogenetic tree of candidate OBPs from *B. longissima* (red labels), *T. castaneum* (purple labels), *A. quadriimpressum* (orange labels), *P*. *striolata* (green labels) and other coleopteran insects (black labels). Antennae-rich OBP in *B. longissima* are indicated by red dots. The tree was constructed using MEGA (v.6.0) with the WAG + G substitution model and NNI topology search, based on an amino acid alignment by MAFFT. Branch support (circles at the branch nodes) was estimated using an approximate likelihood ratio test based on the scale indicated at the top left. Bars indicate branch lengths in proportion to amino acid substitutions per site. OBP prefixes indicate the following: Acor, *A*. *corpulenta*; Aqua, *A*. *quadriimpressum*; Blon, *B*. *longissima*; Cbow, *C*. *bowringi*; Dpon, *D*. *ponderosae*; Ityp, *I*. *typographus*; Pstr, *P*. *striolata*; Tcas, *T*. *castaneum*.

Among the identified transcripts in the *B. longissima* transcriptomes, 34 were candidate OBPs and matched with insect pheromone/OBP domains. All these OBPs encoded 116 to 263 amino acid residues, which is similar to previous reports from other insects, and likely contain full-length ORFs. Signal peptide prediction in the 34 BlonOBPs suggested the presence of signal peptides in all but three OBPs (BlonOBP2, 16, and 24). We further found that based on the presence of cysteine residues^32^, 12 BlonOBPs (BlonOBP1, 2, 5, 7, 9, 16, 17, 18, 19, 21, 28 and 32) with six highly conserved cysteine residues belonged to the Classic class, one belonged to the Plus-C class (BlonOBP20) with 4–6 additional cysteines and one characteristic proline, and the remaining BlonOBPs belonged to the Minus-C class with two cysteine residues at specific places (C2 and C5) (see Supplementary Figure S6). Phylogenetic analysis indicated that most of the Minus-C OBPs clustered into two independent groups with the exception of BlonOBP17 (Classic OBP) that appeared in this Minus-C cluster. The Plus-C BlonOBP20 clustered with other coleopteran Plus-C OBPs to form an independent clade (Fig. 5).

**Figure 6:**
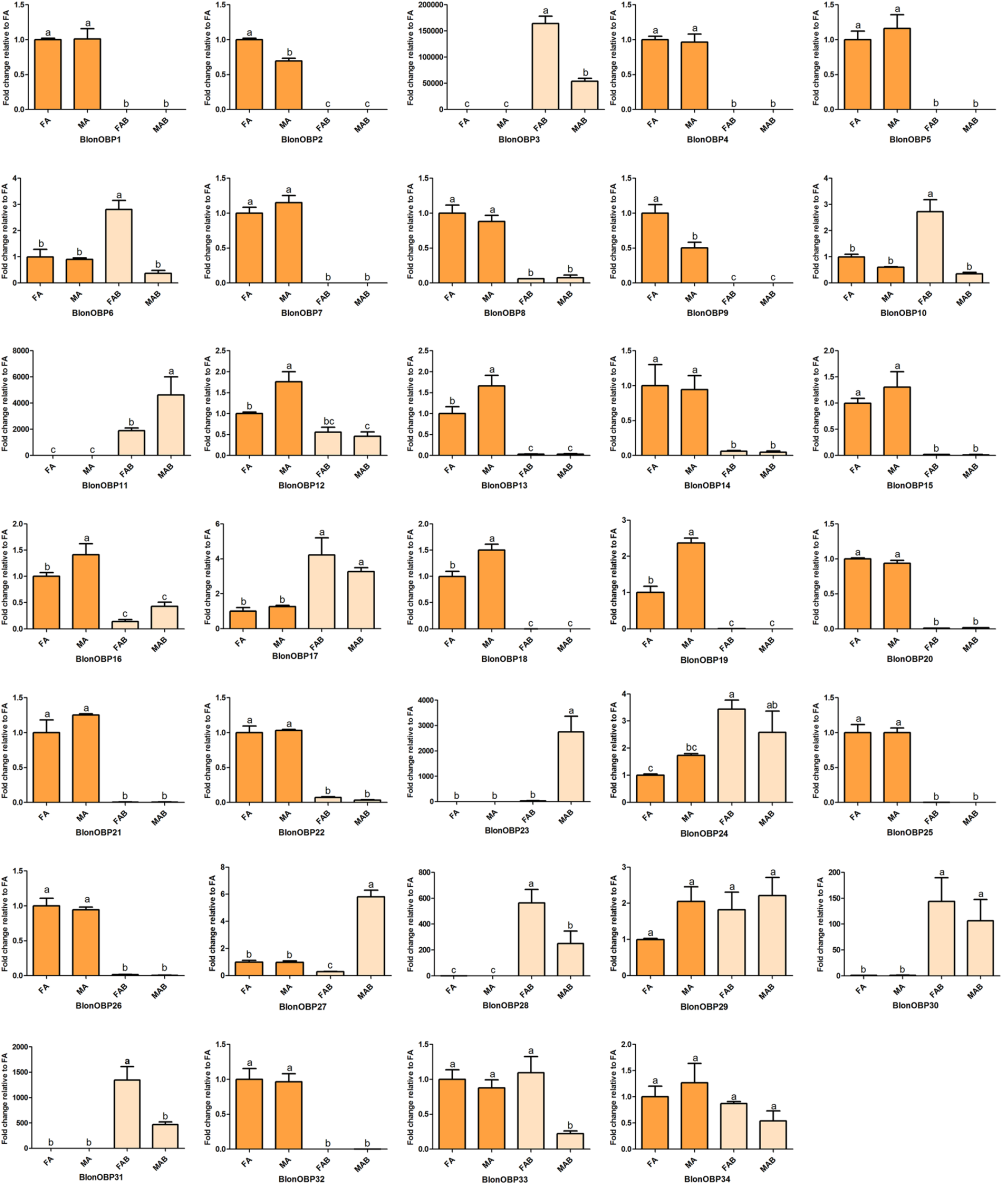
Relative expression levels of all OBPs in the four tissues, using qPCR. FA, female antennae; MA, male antennae; FAB, female terminal abdomens; MAB, male terminal abdomens. The relative expression level is indicated as mean ± SE (N = 3). Different letters indicate significant differences between tissues (P < 0.05, ANOVA, LSD).

All BlonOBPs showed expression in at least one of the analyzed tissues. In both sexes, approximately half (19) of the OBPs (BlonOBP1, 2, 4, 5, 7, 8, 9, 13, 14, 15, 16, 18, 19, 20, 21, 22, 25, 26 and 32) were expressed at higher levels in the antennae when compared to those in the terminal abdomens, while seven OBPs (BlonOBP11, 17, 23, 24, 27, 29 and 31) showed higher levels of expression in the terminal abdomens than in the antennae. Additionally, FPKM values revealed that nine OBPs (BlonOBP2, 4, 7, 9, 10, 13, 15, 18 and 25) were highly abundant in the antennae (FPKM > 1500) with BlonOBP4 showing the highest FPKM values in both sexes (Female: 25263; Male: 24227) (see Supplementary Figure S7). We used qPCR to validate the transcriptome data and compare differential expression of all identified OBP transcripts. RNA-seq data confirmed that 19 BlonOBPs (BlonOBP1, 2, 4, 5, 7, 8, 9, 13, 14, 15, 16, 18, 19, 20, 21, 22, 25, 26 and 32) were enriched in antennae (Fig. 6). Additionally, sex-biased expression of these antennae-enriched OBPs was further determined by qPCR. BlonOBP2 and BlonOBP9 were expressed at higher levels in females, and BlonOBP18 and BlonOBP19 were expressed at higher levels in males. Moreover, BlonOBP12 was also expressed at higher levels in males as well.

#### CSPs

**Figure 7:**
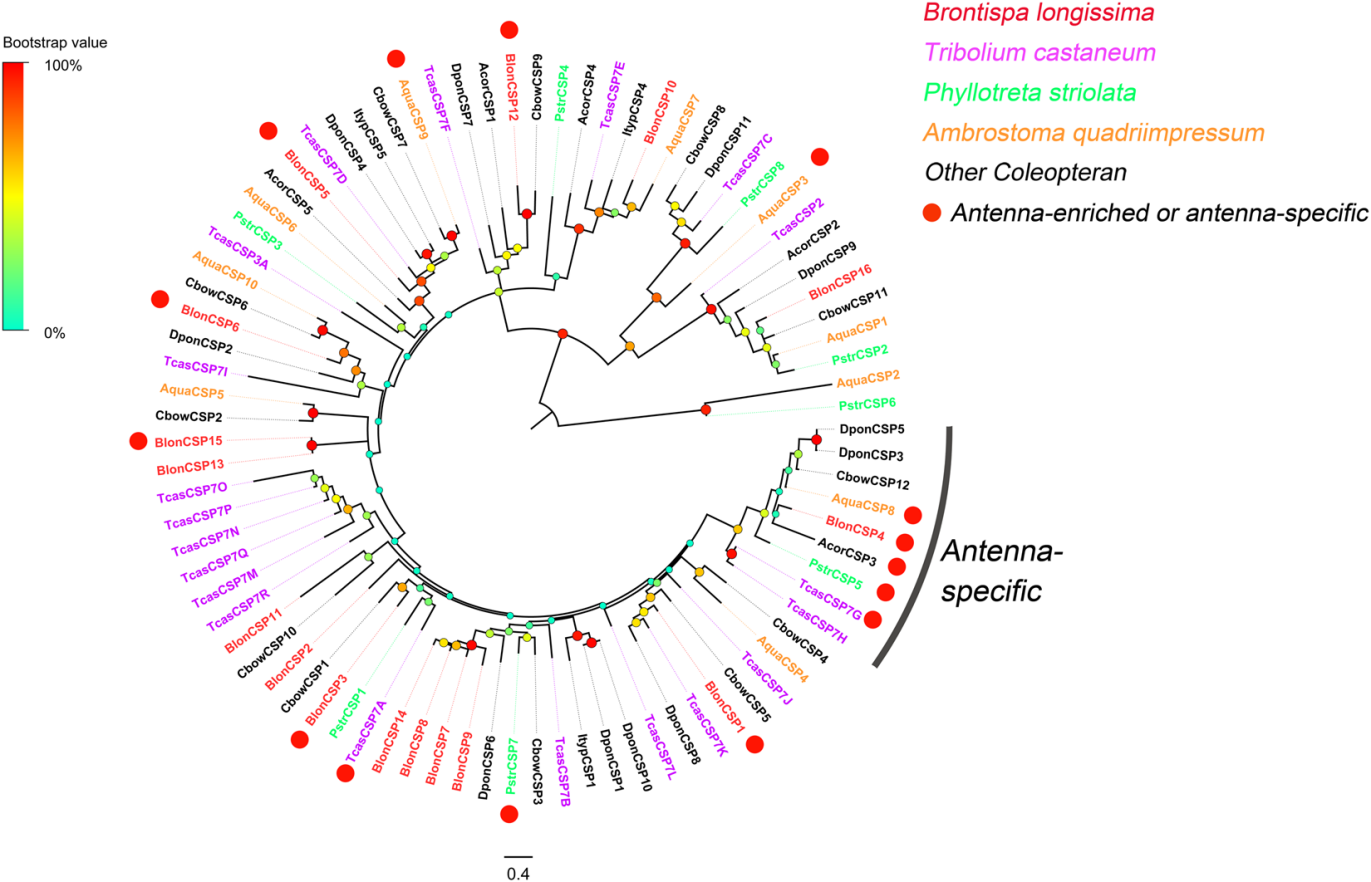
Phylogenetic tree of candidate CSPs from *B. longissima* (red labels), *T. castaneum* (purple labels), *A. quadriimpressum* (orange labels), *P*. *striolata* (green labels) and other coleopteran insects (black labels). Antennae-rich CSP in *B. longissima* are indicated as red dots. The tree was constructed using MEGA (v.6.0) with the WAG + G substitution model and NNI topology search, based on an amino acid alignment by MAFFT. Branch support (circles at the branch nodes) was estimated using an approximate likelihood ratio test based on the scale indicated at the top left. Bars indicate branch lengths in proportion to amino acid substitutions per site. CSP prefixes indicate the following: Acor, *A*. *corpulenta*; Aqua, *A*. *quadriimpressum*; Blon, *B*. *longissima*; Cbow, *C*. *bowringi*; Dpon, *D*. *ponderosae*; Ityp, *I*. *typographus*; Pstr, *P*. *striolata*; Tcas, *T*. *castaneum*.

**Figure 8:**
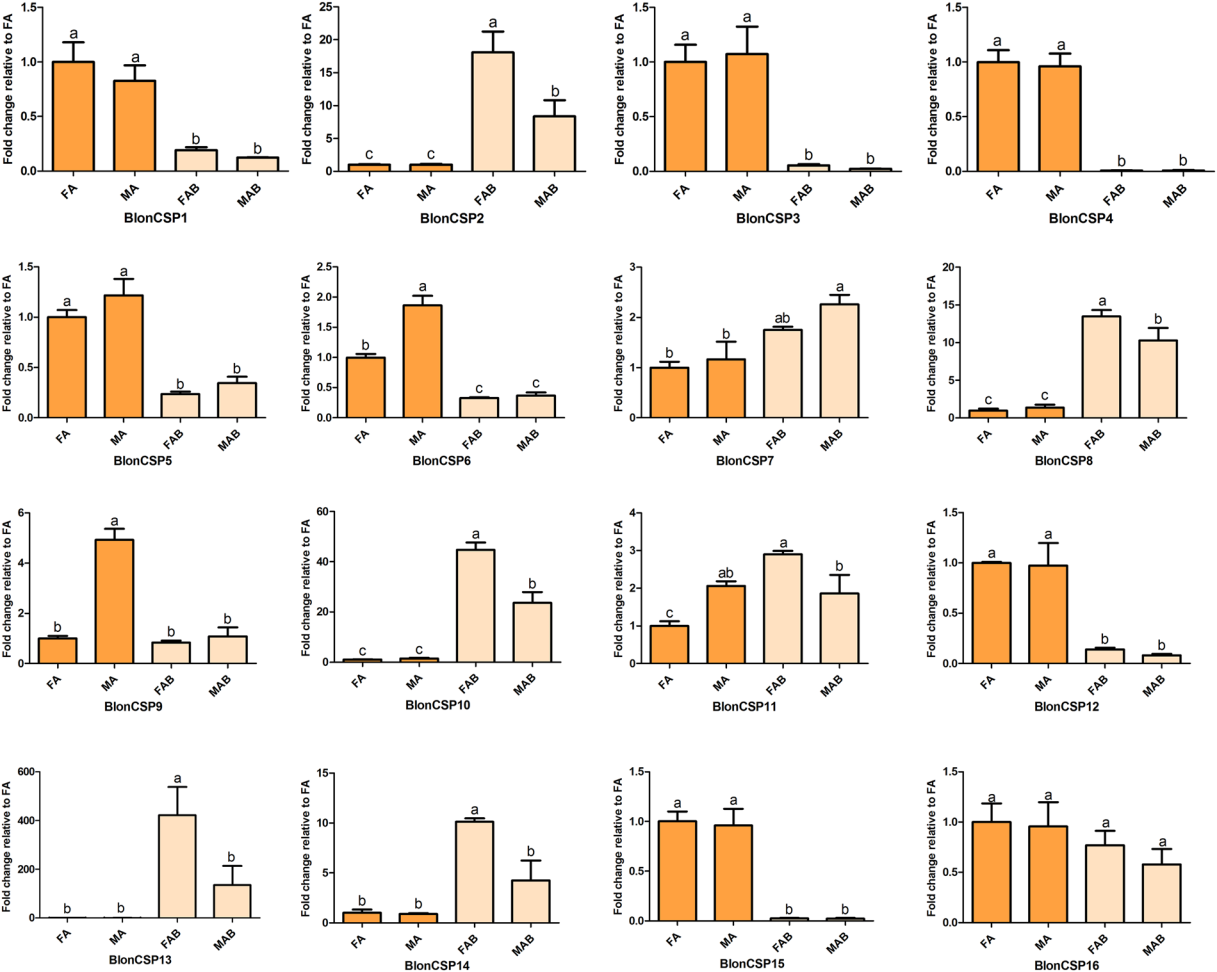
Relative expression levels of all CSPs in the four tissues, using qPCR. FA, female antennae; MA, male antennae; FAB, female terminal abdomens; MAB, male terminal abdomens. The relative expression level is indicated as mean ± SE (N = 3). Different letters indicate significant differences between tissues (P < 0.05, ANOVA, LSD).

Analyses of the four transcriptomes identified 16 transcripts encoding candidate CSPs. All these matched the OS-D/chemosensory protein domains. Among the BlonCSPs, 14 transcripts likely contain full-length ORFs, and possess a signal peptide and the highly conserved four-cysteine profile (see Supplementary Figure S8) in the putative protein sequences. In the phylogenetic tree, two principal branches were observed in conjunction with other coleopteran CSPs; BlonCSP16 clustered with some coleopteran CSPs to form a small clade, while the remaining clustered to form a large clade (Fig. 7). Both RNA-seq data and qPCR results revealed that seven BlonCSPs (BlonCSP1, 3, 4, 5, 6, 12 and 15) displayed predominantly higher expression in antennae of both sexes, while BlonCSP4 was expressed specifically in antennae (Fig. 8 and Supplementary Figure S9). BlonCSP3 had the highest FPKM values followed by BlonCSP6 and BlonCSP1.

## Discussion

Transcriptome sequencing is a powerful technique that allows identification of new genes and large-scale analyses of gene expression. In this study, we constructed four different transcriptomes from the antennae and abdomens of males and females of *B. longissima* and identified potential chemosensory genes in this species. This study represents the first transcriptome analysis of the chemosensory gene repertoire in olfactory (antennae) and non-olfactory (abdomens) tissues to identify and determine differential expression of key molecular components in the coconut hispine beetle.

The numbers of known functional OR genes among different insect species varies from 10 in the human body louse, *Pediculus humanus humanus* ^33^ to about 60 in *D*. *melanogaster* ^34–36^, 262 in *T. castaneum* ^28, 37^, and 350 in the fire ant, *Solenopsis invicta* ^38^. It is suggested that the number of insect ORs contributes to adaptation to a range of habitats and ecological niches^39^. Compared with the numbers of ORs genes identified from other Coleoptera antennal transcriptome, the 48 candidate ORs identified in *B. longissima* were much less than the numbers identified in the heads of adult *T. castaneum* (111)^28^ and in the antennae of *Rhynchophorus ferrugineus* (76)^40^, but more than that identified in most other coleopteran species^29–31, 41–49^. It may reflect a diverse range of odor detection among these coleopterans, e.g. the narrow range of odor detection in adult *B. longissimi* compared to *T. castaneum* and *Rhynchophorus ferrugineus*. Although *B*. *longissima* and *R*. *ferrugineus* share the same host plant, the coconut palm, they have obviously different ecological interests because they inhabit and feed on two different plant parts, leaf and trunk, respectively. However, differences in the identified OR gene numbers may also result from sequencing methods and depth, and/or sample preparation.

As expected, phylogenetic analysis revealed that the expressed *B*. *longissima* ORs grouped into four subfamilies 1, 2, 3 and 7, with other coleopterans. Remarkably, similar to what has been observed in other coleopterans, a species-specific expansion of ORs (subfamilies 2: BlonOR5/14/18/19/41, subfamilies 3: BlonOR24/35/42/48 and subfamilies 7: BlonOR2/27/30/32/38) was also found in *B*. *longissima*, which may indicate that these distinct species inhabit different ecological niches. The functional analysis of *M*. *caryae* ORs (McarOR3, McarOR20, and McarOR5) has revealed that they were tuned respectively to three aggregation pheromone components ((S)-2-methyl-1-butanol, (2 S, 3 R)-2, 3-hexanediol and 2-phenylethanol)^30^. Phylogenetic analysis showed that BlonOR4 clustered together with McarOR5, indicating that BlonOR4 may have a function similar to that reported in *M*. *caryae* (Fig. 1).

Insect IRs are divided into several major subfamilies based on their chemoreception; IR25a/IR8a are ubiquitously expressed and function as co-receptors, “antennal IRs” are expressed in antennae, highly conserved across insect orders and function in odor perception, and the “divergent IRs” are species-specific with some expressed in gustatory organs and function in taste perception^8^. In our study, the two IR co-receptors (BlonIR25a/IR8a) and all antennal IRs we identified were enriched in antennae, which is consistent with the expression characteristics of insect IRs. A phylogenetic analysis revealed that 4 BlonIRs (BlonIR21a, 68a, 76b and 93a) grouped with their homologs in *T. castaneum*, *Phyllotreta striolata* (two coleopterans) and *D. melanogaster*, and 3 BlonIRs (BlonIR75q.2 A, 75q.2B and 75 s) clustered with their homologs from *T. castaneum* and *P. striolata* thus displaying coleopteran-specific clades (Fig. 3). These findings indicate the high degree of conservation in these antennal IRs across insect orders, and perhaps may play the same or similar roles in chemodetection. Interestingly, we did not identify divergent IRs in this study.

Insects generally possess two SNMP subfamilies (SNMP1 and SNMP2). SNMP1 is located in the pheromone-specific olfactory neurons in the antennae consistent with its role in pheromone (11-cis vaccenyl acetate, cVA) detection in *D. melanogaster* ^9, 10^. In this study, as expected, two SNMP1 homologs (BlonSNMP1a and BlonSNMP1b) from the coconut hispine beetle had higher expression in antennae when compared to abdomens. Additionally, transcriptomic studies showed that BlonSNMP1a is the only SNMP gene to have highest expression in antennae compared to others, suggesting that BlonSNMP1a may play a role similar to its homolog in *D. melanogaster*.

Expression profiles of both OBPs and CSPs in the coconut hispine beetle displayed equal numbers of transcripts preferentially expressed in antennae and abdomens. This is consistent with the general understanding that insect CSPs and OBPs are not restricted to the main chemosensory tissues^13^. To further explore the role of these proteins in chemosensory perception, we focused only on the antenna-enriched or antenna-specific OBPs and CSPs. Compared to the previously characterized coleopteran OBPs and CSPs from *T. castaneum*, *Ambrostoma quadriimpressum* and *P. striolata* ^41, 42, 48^, 16 OBPs and 7 CSPs were up-regulated in *B*. *longissima* antennae, which is more than the number up-regulated in the antennae of *T. castaneum* (11 OBP and 3 CSPs)^48^, *P*. *striolata* (10 OBPs and 2 CSP)^42^ and *A. quadriimpressum* ^41^. Because different OBPs are present in a particular olfactory sensillum, OBPs may also play a role in olfactory coding^50, 51^. Thus *B. longissima* with higher numbers of antenna-rich OBPs and CSPs may require a high number of different carrier proteins. In addition, phylogenetic analyses revealed that the *B*. *longissima* OBPs up-regulated in antennae were assigned to the Minus-C OBPs, Classic OBP and Plus-C OBP classes, and some grouped with *T. castaneum*, *P. striolata* and *A. quadriimpressum*. For example, BlonOBP18/19 grouped with AquaOBP1/2, BlonOBP5 with AquaOBP4, and BlonOBP1/2 with TcasOBP5C/5D (Fig. 5). This indicates that these OBPs in Coleopterans likely possess similar olfactory perception functions. In the phylogenetic tree of CSPs, we also observed a clade that included BlonCSP4 and most coleopteran CSPs exclusively expressed in antennae (Fig. 7). It appears that these members in Coleopterans are highly conserved, and likely endowed with olfactory perception functions. In addition, some interesting patterns of expression were found; a number of OBPs were preferentially expressed in female terminal abdomens (BlonOBP3, 6, 10 and 31) or male terminal abdomens (BlonOBP23 and 27), indicating a possible involvement of these OBPs in non-sensory functions. In some cases, insect OBPs have been reported both in pheromone glands and in reproductive organs, where they likely solubilize and bind sex-specific pheromones^13^. However, some discrepancies were observed between the qPCR data and RNA-seq data for BlonOBP27, BlonCSP5 and BlonIR68a. For these transcripts, we presume that the qPCR data with three technical replicates and three biological replicates may be more reliable than the RNA-seq data with one biological and technical replicate.

## Conclusion

*B. longissima* (Gestro) is one of the most dangerous pests to Palmae plants particularly for the economically important tropical plants such as the coconut palm. In this study, wegenerated four antennal and abdominal transcriptomes from both sexes of *B. longissima*, and identified an extensive set of candidate genes including OR, IR, SNMP, OBP, and CSP. Phylogenetic analyses were conducted to determine the evolutionary relationship between the chemosensory genes of *B. longissima, D. melanogaster* and other coleopteran insects, and identified putative aggregation pheromone receptors in *B. longissima*. Transcriptome analysis along with qPCR data revealed clear sex- and tissue-specific expression patterns in chemosensory gene families. These findings reveal the identification of key molecular components in *B. longissima* olfaction and provide a valuable resource for future functional analysis of olfaction.

## Methods

### Insect rearing and tissue collection

Beetles used in the study were obtained from the Chinese Academy of Tropical Agricultural Sciences in Hainan and was originally collected from a coconut palm in Sanya (18°150′N, 109°300′E), Hainan, China, in 2012. Larvae and adults were reared on fresh coconut palm leaves in plastic containers (15.5 cm × 11.5 cm × 5.0 cm), with a mesh window, maintained at 25 ± 2°С with 75–80% relative humidity, and a 12 h light:12 h dark photoperiod. Newly emerged adults were separated into females and males, and were transferred to a new plastic container with fresh leaves and reared under the same conditions as the stock culture for 30 days. For transcriptome sequencing, antennae (approximately 300 from each sex) and terminal abdomens (the last two abdominal segments including the genitals, approximately 100 from each sex) from males and females were excised from 30-day-old adults, and immediately frozen and stored in liquid nitrogen until RNA extraction.

### RNA extraction, cDNA library construction and Illumina sequencing

Male antennae, female antennae, male abdomens and female abdomens collected as described above, were each homogenized into powder, and then total RNA was extracted with the RNeasy Mini kit (Qiagen) according to the manufacturer’s instruction. RNA concentration was determined on a NanoDrop ND-2000 spectrophotometer (Thermo Scientific, America), and RNA integrity value (RIN) was checked on the Bioanalyzer 2100 system (Agilent Technologies, USA). The cDNA libraries were generated according to Illumina’s sample preparation instructions (Illumina, San Diego, CA). Briefly, each cDNA library was constructed using a TruSeq™ RNA Kit (Illumina, San Diego, CA) in the following steps: purification of mRNA, fragmentation of mRNA, synthesis of the first and second cDNA strands, cDNA end repair and adenylation at the 3′ end, and adapter ligation and cDNA fragment enrichment. The products were amplified by PCR and purified using a QIAquick PCR Purification Kit (Qiagen, Valencia, CA, USA) to create the final cDNA library, which was validated and quantified on the Bioanalyzer 2100 system. Libraries were sequenced using the Illumina HiSeq4000 system (Illumina Inc. San Diego, CA) to obtain paired-end reads.

### Assembly and functional annotation

The unknown (poly-N) or low-quality sequences and adaptor sequences were removed from raw reads to obtain clean reads for further analysis. *De novo* assembly of all clean reads was carried out using the Trinity program^52, 53^ to generate unigenes with min_kmer_cov set to 2 and all other parameters set at default. The resulting unigenes were first aligned using BLASTx against the NCBI non-redundant (nr) protein database (E-value < 10^−5^) to retrieve proteins with the highest sequence similarity to the given unigenes along with their putative functional annotations.

### Identification of putative chemosensory genes

Putative *B. longissima* chemosensory genes were identified in the following steps. First, tBLASTn algorithm with E-value < 10^−5^ was used to identify homologs of *B. longissima* unigenes using as queries published chemosensory gene sequences (ORs, IRs, SNMPs, OBPs and CSPs) from other coleopteran species (*T. castaneum* ^28, 37, 48^, *Tomicus yunnanensis* ^54^, *Megacyllene caryae* ^30^, *Ips typographus* and *Dendroctonus ponderosae* ^29^, *Agrilus planipennis* ^49^, *Batocera horsfieldi* ^55^, *Anomala corpulenta* ^44, 47^, *Colaphellus bowringi* ^31^, *Tenebrio molitor* ^46^, *Dendroctonus valens* ^45^, *Anoplophora glabripennis* ^43^, *Phyllotreta striolata* ^42^, *Ambrostoma quadriimpressum*)^41^. Then, the resulting candidates were used as queries further to identify additional genes (tBLASTx and BLASTp). Finally, results of tBLASTx and BLASTp were verified using the BLASTx algorithm against the NCBI nr protein database. All candidate *B. longissima* chemosensory genes identified were named with the prefix Blon followed by the gene family name such as BlonORs, BlonIRs, BlonSNMPs, BlonOBPs and BlonCSPs. The open reading frames (ORFs) of these unigenes were predicted using the ORF finder tool at NCBI. Presence of definitive domain(s) in ORs, IRs, SNMPs, OBPs and CSPs was further confirmed against InterPro using the InterProScan tool plug-in in Geneious version 9.1.3^56^.

### Sequence alignment and phylogenetic analysis

Multiple predicted amino acid sequences of putative BlonOBPs and BlonCSPs (without the signal peptides, predicted using SignalP 4.1, http://www.cbs.dtu.dk/services/SignalP)^57^, and BlonIRs (combination of BlonIRs and iGluRs and IRs of *T*. *castaneum*, listed in Supplementary Table S3) were aligned with the E-INS-I strategy in the MAFFT^58^ alignment tool plug-in in Geneious (version 9.1.3.)^59^, and further visualized.

Amino acid sequences (after removing the signal peptides in OBP and CSP datasets) were aligned using the MAFFT (v7.149)^58^ alignment tool plug-in in Geneious software 9.1.3, with the E-INS-I parameter set. Dendrograms of ORs, SNMPs, OBPs and CSPs were calculated using maximum likelihood analysis with MEGA6^60^ with the corresponding best substitution model (WAG + G model), while for IRs it was calculated using FastTree2^61, 62^ with the JTT substitution model, and visualized with FigTree (http://tree.bio.ed.ac.uk/software/figtree). Node support of phylogenetic tree was assessed using the bootstrap method with 1000 bootstrap replicates.

Phylogenetic analysis of BlonORs, BlonIRs, BlonSNMPs, BlonOBPs and BlonCSPs was performed in conjunction with the published chemosensory genes sequences in other insects. Incomplete transcripts without sufficient overlap in alignments and transcripts translating lesser than 180 amino acids in length (some OBP and CSP transcripts could be translated only to 50 amino acids and in most cases the full-length ORF transcripts are shorter than 200 amino acids) were excluded from phylogenetic analyses to ensure that the analyzed transcripts corresponded to individual genes and that greater accuracy in the analyses was maintained. All amino acid sequences used for phylogenetic tree construction are listed in Supplementary Table S3.

### Expression level analysis and differentially expressed genes (DEGs)

Clean reads were mapped back to the assembled transcriptome and read count for BlonORs, BlonIRs, BlonSNMPs, BlonOBPs and BlonCSPs was obtained from the mapping results. The expression levels of these candidates were assessed using the fragment per kilobases per million reads (FPKM) method^63^ using RSEM^64^. To normalize the count numbers, FPKM values were calculated and plotted as log_10_ FPKM + 1. The values were visualized using edgeR^65^. DESeq package^66^ was used to test for differential expression between two samples and the following three comparisons were made: female antennae vs. male antennae, female antennae vs. female terminal abdomen, and male antennae vs. male terminal abdomen. False discovery rate (FDR) and *p* value were used to detect genes with differential expression between samples. Genes with FDR ≤ 0.01 were considered as candidates and *p* values less than 0.001 represented significant differences in the differential expression of genes. The fold change of FPKM was computed, and genes with fold change ≥ 3 were characterized as DEGs.

### Quantitative real time-PCR (qPCR) validation

qPCR experiments were then conducted to verify the gene expression levels estimated with a repeat RNA-seq data. The chemosensory gene validation included most of the ORs (RNA-seq expression levels of the top 15 overexpressed OR genes in antennae), and all antennal IRs, whole OBPs and CSPs. An equal amount of total RNA (3 ng) extracted from male antennae (100), female antennae (100), male terminal abdomens (50), and female terminal abdomens (50) was digested with DNase I to remove potential DNA contamination, and reverse transcribed into cDNA using PrimeScript RT reagent Kit with gDNA Eraser (Takara, China). The qPCR was performed on a Light Cycler 480 System (Roche Applied Science) using a mixture of 10 μl SYBR Green I Master mix (Roche Applied Science), 1 μl of each primer (0.5 μM), 2 μl (2.0 ng) of sample cDNA and 6 μl sterilized PCR grade water. The qPCR reaction consisted of the following programs: denaturation at 95 °C for 5 min, followed by 45 cycles of 95 °C for 10 sec, and 60 °C for 20 sec. A melting curve analysis was then performed at 95 °C for 20 sec, 60 °C for 30 sec, and 95 °C for 30 sec in order to determine the specificity of the PCR products. Specific primers were designed for the qPCR using Primer3web (version 4.0.0) (http://primer3.ut.ee/) (Supplementary Table S2). To verify the absence of genomic DNA in the samples, gene specific primers outside an intron were used to amplify and visualize on an agarose gel electrophoresis. The specificity of all primers was validated by melt-curve analysis and efficiency was calculated by analyzing standard curves with a five-fold cDNA dilution series. To normalize the gene expression studies, two endogenous reference genes, actin alpha 1 (BlonACT1) and glyceraldehyde-3-phosphate dehydrogenase 2 (BlonGAPDH2) were selected (listed in Supplementary Data File). Negative controls for the amplification replaced water for cDNA. For each sample, three biological replications (3 separate RNA extractions from samples) were performed with each biological replication having three technical replications. The results were analyzed using the LightCycler 480 Gene Scanning Software. The comparative 2^−ΔΔCT^ method was used to calculate the relative expression levels of genes between tissues^67^. The statistical significance of the differences of each target gene among the various tissues were determined using one-way nested analysis of variance (ANOVA), followed by the least significant difference test (LSD) for mean comparison using SPSS Statistics 17.0 (SPSS Inc., Chicago, IL, USA) All values are presented as the mean ± SE.

## Electronic supplementary material

Supplementary information

Supplementary Dataset
