# Supplementary material for: Antennal and abdominal transcriptomes reveal chemosensory gene families in the coconut hispine beetle, Brontispa longissima
Source: Sci Rep. 2017 Jun 5;7:2809. doi: 10.1038/s41598-017-03263-1 (PMC5459851; doi:10.1038/s41598-017-03263-1)
Supplement: Supplementary file 1 — Supplementary information [file 41598_2017_3263_MOESM1_ESM.pdf]

Supplementary Information for

Antennal and abdominal transcriptomes reveal chemosensory gene families in the coconut hispine beetle, *Brontispa longissima*

Shu-Ying Bin[1], Meng-Qiu Qu[1], Ke-Ming Li[2, 3], Zheng-Qiang Peng[3], Zhong-Zhen Wu[1*], Jin-Tian Lin[1*]

[1]Institute for Management of Invasive Alien Species, 314 Yingdong teaching building, Zhongkai University of Agriculture and Engineering, Guangzhou 510225, PR China

[2]Institute of Banana and Plantain, Chinese Academy of Tropical Agricultural Sciences, Haikou 570102, PR China

[3]Institute of Environment and Plant Protection, Chinese Academy of Tropical Agricultural Sciences, Haikou 570101, PR China

Correspondence and requests for materials should be addressed to Zhong-Zhen Wu (zhongzhen_wu@163.com) or Jin-Tian Lin (linjtian@163.com)

[*]These authors contributed equally to this work.

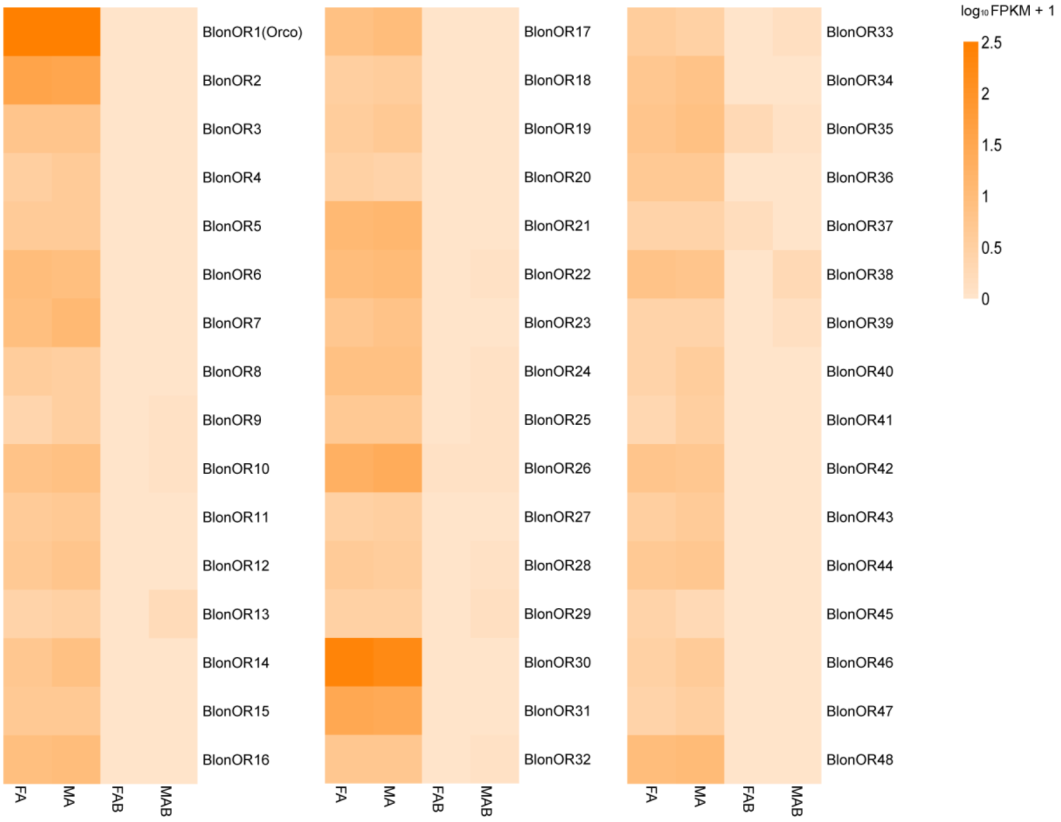

Supplementary Figure S1. Expression profiles of candidate *B. longissima* ORs. Expression levels in the four transcriptomes are represented as heat plots based on $\log_{10}$ FPKM values and the different shades represent various expression levels based on the scale indicated on the right. Zero expression is represented by the lightest orange color. FA, female antennae; MA, male antennae; FAB, female terminal abdomens; MAB, male terminal abdomens.

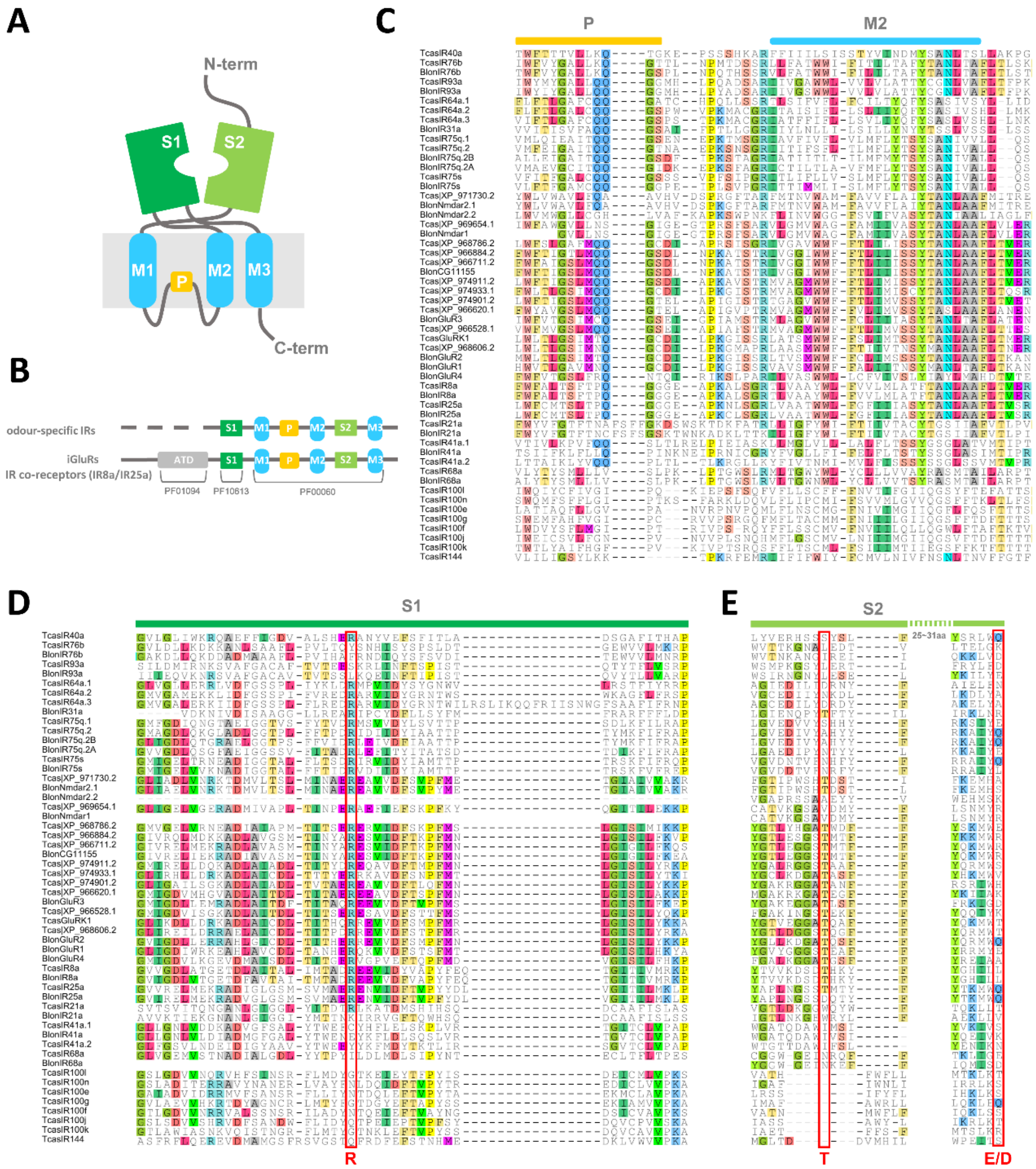

Supplementary Figure S2. Excerpts from the amino acids alignment showing the predicted iGluRs/IRs binding domains. Predicted protein domain organization of IRs and iGluRs/IR co-receptors illustrated in diagram (A) and linear (B) form with associated Pfam predicted domains (adapted from [7, 8]). (C) MAFFT amino acid alignment of the ion channel pore (P) and second transmembrane (M2) domains of *B. longissima* IRs and *Tribolium castaneum* IRs and iGluRs. MAFFT amino acid alignments of the S1 (A) and part of S2 (B) ligand binding domains of candidate *B. longissima* IRs and *T. castaneum* IRs and iGluRs. The key binding residues in iGluRs are in red boxes.

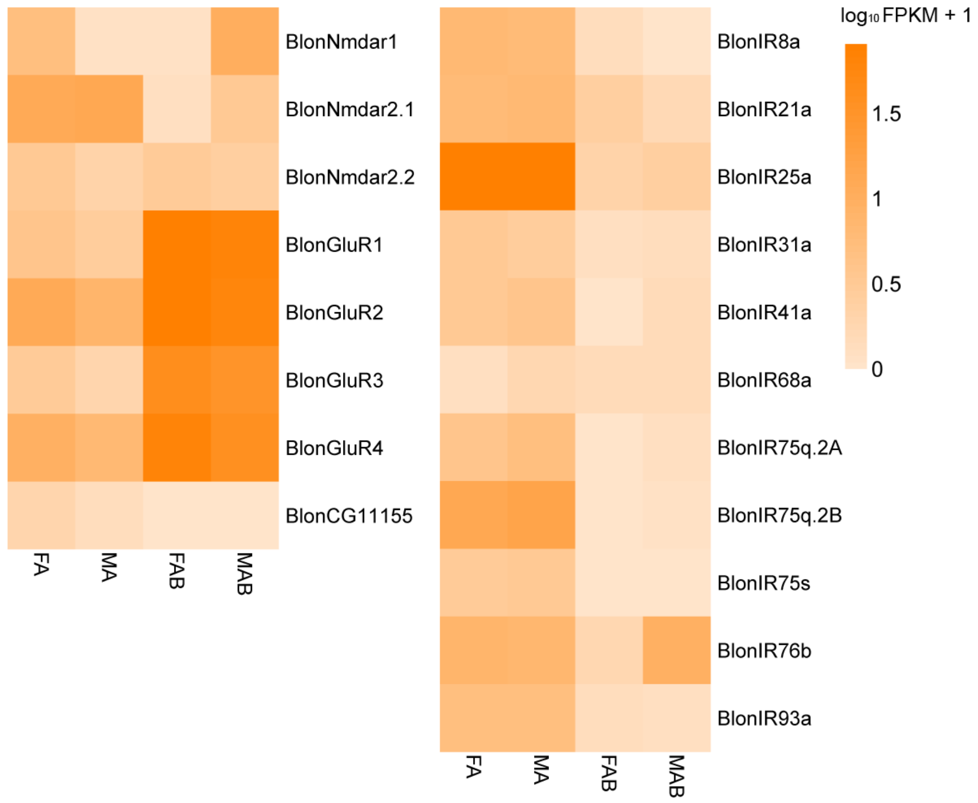

Supplementary Figure S3. Expression profiles of candidate *B. longissima* IRs. Expression levels of the IRs in the four transcriptomes are represented as heat plots based on log_{10} FPKM values and the different shades represent various expression levels based on the scale indicated on the right. Zero expression is represented by the lightest orange color. FA, female antennae; MA, male antennae; FAB, female terminal abdomens; MAB, male terminal abdomens.

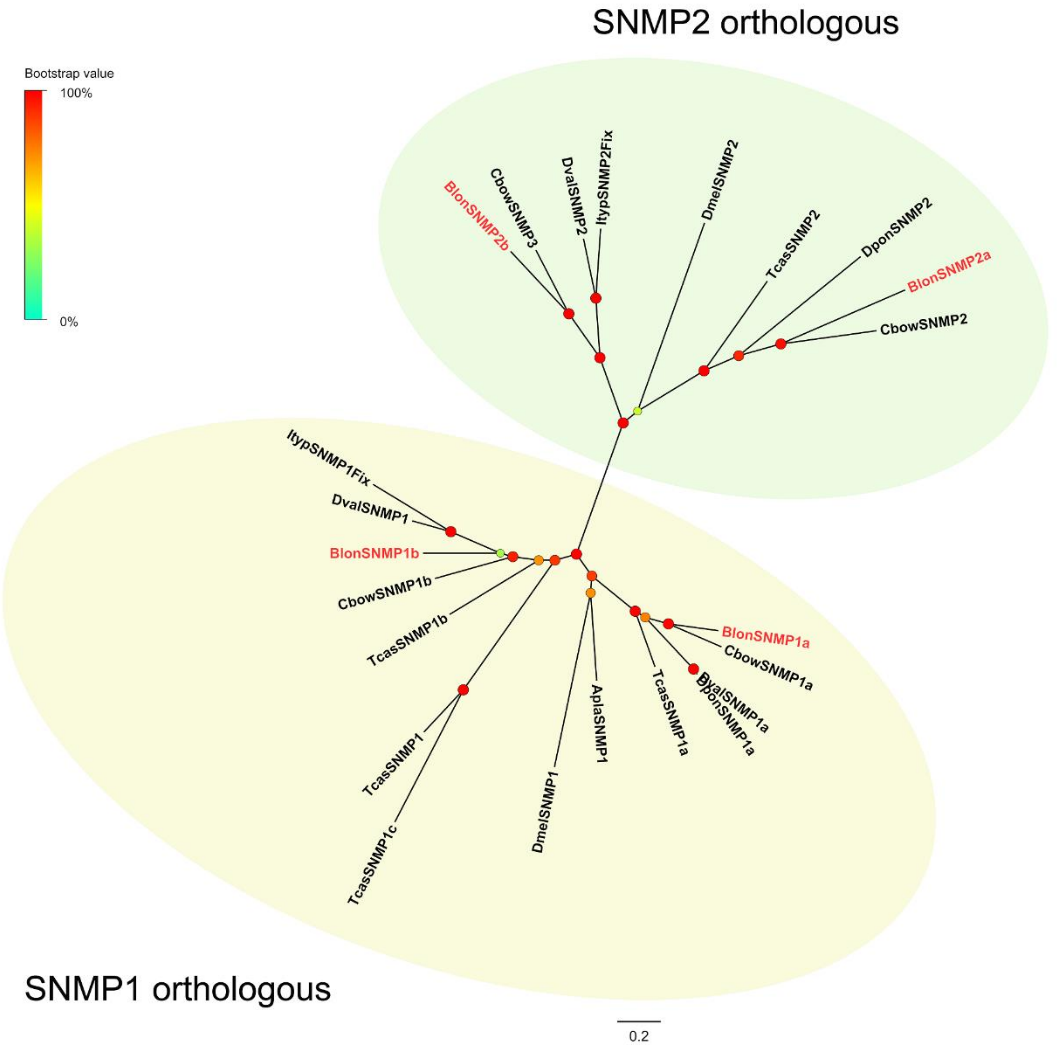

Supplementary Figure S4. Phylogenetic tree of candidate *B. longissima* SNMPs (red labels) with other insect SNMPs. The tree was constructed using MEGA (v.6.0) with the WAG+G substitution model and NNI topology search, based on an amino acid alignment by MAFFT. Branch support (circles at the branch nodes) was estimated using an approximate likelihood ratio test based on the scale indicated at the top left. Bars indicate branch lengths in proportion to amino acid substitutions per site. Other insect SNMP sequences used to construct the tree are labelled black. SNMP prefixes indicate the following: Apal, *A. planipennis*; Blon, *B. longissima*; Cbow, *C. bowringi*; Dmel, *D. melanogaster*; Dval, *D. valens*; Dpon, *D. ponderosae*; Ityp, *I. typographus*; Tcas, *T. castanuem*.

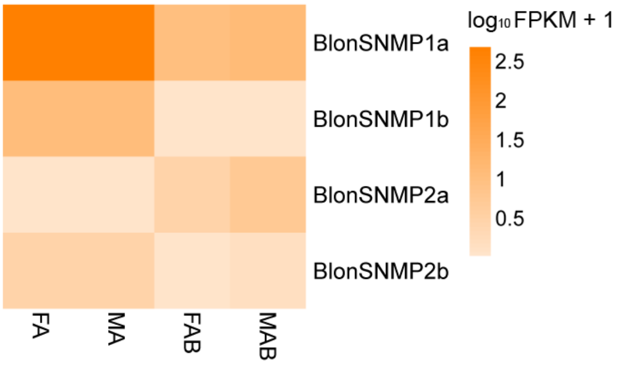

Supplementary Figure S5. Expression profiles of candidate *B. longissima* SNMPs. Expression levels of the SNMPs in the four transcriptomes represented as heat plots based on $\log_{10}$ FPKM values and the different shades represent various expression levels based on the scale indicated on the right. Zero expression is represented by the lightest orange color. FA, female antennae; MA, male antennae; FAB, female terminal abdomens; MAB, male terminal abdomens.

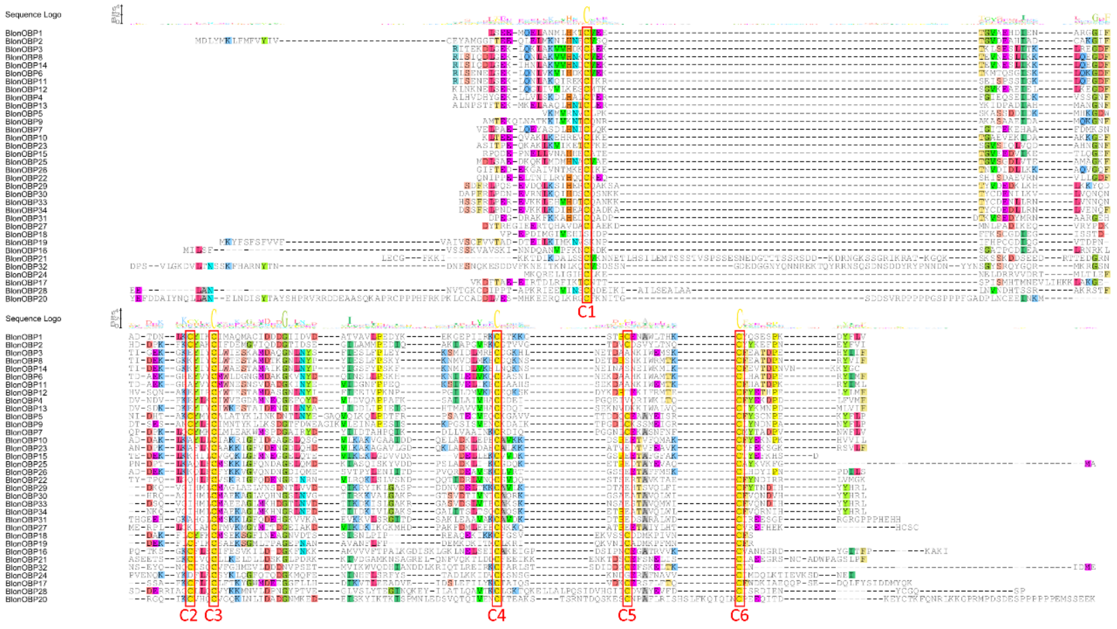

Supplementary Figure S6. Excerpts from the amino acids alignment showing the predicted *B. longissima* OBPs and conserved cysteine residues. In the sequence logo stacks, the height of each stack corresponds to the degree of sequence conservation at that position.

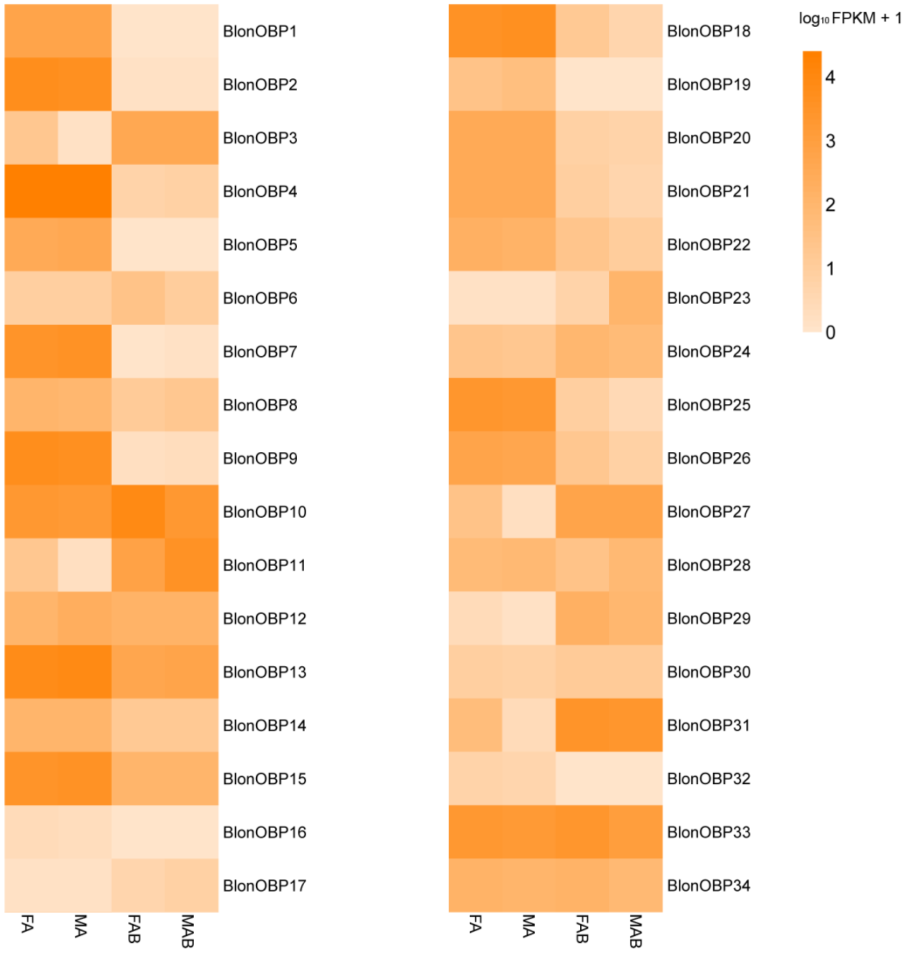

Supplementary Figure S7. Expression profiles of candidate *B. longissima* OBPs. Expression levels of the OBPs in the four transcriptomes represented as heat plots based on log_{10} FPKM values and the different shades represent various expression levels based on the scale indicated on the right. Zero expression is represented by the lightest orange color. FA, female antennae; MA, male antennae; FAB, female terminal abdomens; MAB, male terminal abdomens.

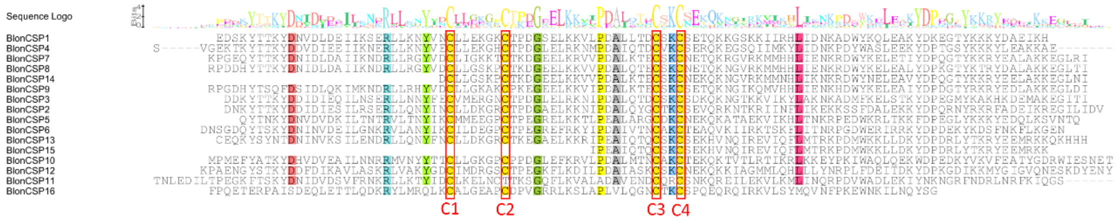

Supplementary Figure S8. Excerpts from the amino acids alignment showing the predicted *B. longissima* CSPs and conserved cysteine residues. In the sequence logo stacks, the height of each stack corresponds to the degree of sequence conservation at

that position.

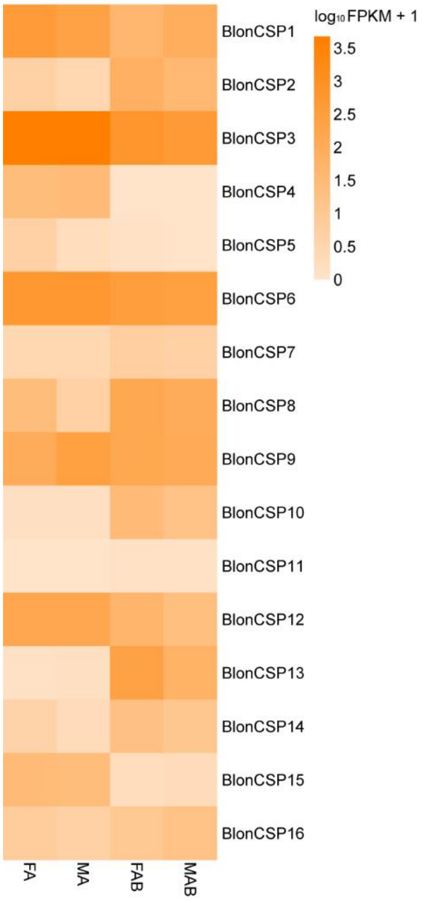

Supplementary Figure S9. Expression profiles of candidate *B. longissima* CSPs. Expression levels of the CSPs in the four transcriptomes represented as heat plots based on log_{10} FPKM values and the different shades represent various expression levels based on the scale indicated on the right. Zero expression is represented by the lightest orange color. FA, female antennae; MA, male antennae; FAB, female terminal abdomens; MAB, male terminal abdomens.

Supplementary Table S1. An overview of the sequencing and assembly process.

	**FA**	**MA**	**FAB**	**MAB**
Read Length	150	150	150	150
Total Raw Reads	47,263,100	46,757,326	42,911,788	47,394,060
Total Clean Reads	47,162,716	46,654,298	42,811,068	47,324,228
GC percentage	40.2%	40.21%	40.22%	40.28%
Total Raw Bases	7,089,465,000	7,013,598,900	6,436,768,200	7,109,109,000
Total Clean Bases	7,074,407,400	6,998,144,700	6,421,660,200	7,098,634,200
Total Adapter Reads	97,418	97,280	95,424	66,812
Total Small Insert Reads	0	0	0	0
Total PolyA Reads	0	0	0	0
Total Low Quality Reads	2,966	5,748	5,296	3,020
Total Contig Number	84,703	78,729	60,444	67,998
Total Contig Length (nt)	41,064,957	39,303,819	33,637,865	37,616,309
Contig Mean Length (nt)	485	499	557	553
Total Unigene Number	29,508	28,215	24,151	27,948
Total Unigene Length (nt)	44,471,026	42,786,501	36,891,540	43,295,774
Contig Unigene Length (nt)	1,507	1,516	1,528	1,549

Supplementary Table S2. List of primers used in qPCR.

Gene Name	Forward Primer (5'-3')	Reverse Primer (5'-3')	TM (℃)	Product size (bp)
BlonACT1	TCCTCATGCCATCCTACGTC	AACTGGAACTTGCTGCTGTG	59	191
BlonGAPDH2	CTTGACGCCTACAATCCAGC	CAGTTGATGCGGGGATGATG	59	226
BlonCSP1	TGCTATTGGTTTGCGTCTTCA	GAGCATTTTGAGCAGTCCGT	59	214
BlonCSP2	TCCGTTTTGATAAGTGTTTGTGC	CGTCAGGGGTACATCTTCCT	59	154
BlonCSP3	TGTAGCTGTCGTATCTGGGG	CACTGCATTTAGAACAGTCGGT	59	197
BlonCSP4	GCCATTCAAACGGACTGTGT	GTTCAGCTCGCAGTGGTTTC	59	196
BlonCSP5	TGCTTTATCACCGTTGCTGG	CGAGAGCTTCAGGTAGGGTT	59	184
BlonCSP6	GAAGGACCCTGCACACCA	TTCCCAATCACCAGGTCTGT	59	150
BlonCSP7	GTTGGCCTTTGTGTAGTAGCA	TCTTTTCTGTGTCTCGTTGCA	59	225
BlonCSP8	TGAGAGGTTACGTGGACTGC	ACCAATCACGCTTGTTTTCGA	59	174
BlonCSP9	GCTTTCTTGCTCTTTGGGCA	TCTTCTCCTTCTTTGGGGCA	59	163
BlonCSP10	CCAAAAGCACCACCACTACC	GCGATATTAGCCCCAGTTGC	59	196
BlonCSP11	GCTGACGCTGTTGAAAATGAC	ATCTGTCGTTGAATCTGCCG	59	151
BlonCSP12	GCGAAACCAGCGGAAAATG	GCAAAAGGTGTTGAAGCATCA	59	235
BlonCSP13	TGTCGTTTGTGCTTGTGAACA	AGCATTGGGTTTGAATCGCT	59	182
BlonCSP14	CCTGATGCACTGAAGACCGA	ACGTTTCTTGTAAGTACCCTGAG	59	150
BlonCSP15	TCAAGTGCAACCAGATCTCTT	AGCATTGGGTTTGAATCGCT	59	164
BlonCSP16	AGAAACCGAACGACCTGCTA	TCTTTGTTCTTGTGGGCTGC	59	190
BlonOBP1	TGTGGTGGCAAATAAGTGTCT	GGTTGTCAGTGTCTGCGAAT	59	183
BlonOBP2	CATCGCTGACTGTGCCAAAG	ACTCCAGGTGCTATTTTCGC	59	168
BlonOBP3	TGTGCCGAGAAAACAAAGCT	TCCATCGCATTCCCTCATCA	59	213
BlonOBP4	TGCTTGCTCTGATGATATTGGC	AGCATCCCCTTCTACCCAAA	59	221
BlonOBP5	ACCAAAGTCAAAAGCGTCGA	TGCTCCTTTACACTGCTCCA	59	223
BlonOBP6	TGGATGGCAATGGGATGGAT	AGGGTCGGTCGCATGTAAA	59	182
BlonOBP7	AACACGCAGCCTTCGATATG	CTTTGATTTGCGGATGGGCT	59	150
BlonOBP8	TGAGAGCACGGCAATGGATA	AACGATTTGGGTCTGTTGCC	59	188
BlonOBP9	TGCTGGTTATTCTGTTCGTGA	CCAATCAAAAGTACCGTCGCT	59	245
BlonOBP10	AGGCGTCGTTCTTGTTTCTT	GTAACTCTCCAGCGCCATCT	59	233
BlonOBP11	ATTGCGTTGTTCAGTGGCTT	ATCTGCATCCACCGAATTGC	59	219
BlonOBP12	CAGCTATGGATGCAAGTGGT	CGTAGAAGCACTCCGTCATTC	59	162
BlonOBP13	TCTGGAATTCGGTGTGCTCT	CGGTGCTCTCTTTCCATATGC	59	193
BlonOBP14	GCGTTTGATTGTTTTCACTGCA	TTGCCCTTAATAGCCATCGC	59	218
BlonOBP15	TGTGTGTGCCTTGGTATTGG	TGACTTCGGTTTGCAGTTCA	59	227
BlonOBP16	GATAAATCCGGTGCTACGCC	GAATCCGGACCGATCTCCTT	59	242
BlonOBP17	TTCACTGCCTGTTCACGAAG	CTCGCACTTATCCACCGACT	59	158
BlonOBP18	AAGTTGCGGTGACATTGAGG	ATCTTCTCCTGAGCTTCCCG	59	162
BlonOBP19	TCAGTTTTGTGGTGTTTGTGG	TTCTCGTCATTGGGATCCGT	59	151
BlonOBP20	CCTCCACCGCATTTTCGAAA	AGTTTCTGTCCAACGCACTG	59	245
BlonOBP21	TGCTAGTGAAGAAACGTCCG	TCCCTCTTCAGCCAAACAAC	59	250
BlonOBP22	TCGCCGTACTTGTCTACTGT	TGTTGACCGAGTTGTGGAGT	59	172
BlonOBP23	CAATTGCGGCGAGTATCACA	TCTTTGCAGCACACACCAAA	59	165
BlonOBP24	TGACAGAAGAGTTGTGGACAGA	GTTCACCGTCGTTCTTTCCC	59	227
BlonOBP25	CGGTTTCCAAAATGATGCTGG	CACATTGCGCCACTTCGTA	59	155
BlonOBP26	TGGTTGTTTGCTCCTTGTCG	TCGTTTTGCACACCGACTTT	59	208
BlonOBP27	ACACAACACGCTGTAGATGC	CTTTGCGATCTCACCGTCTG	59	171
BlonOBP28	TCTCGTGCTGAAGAACTTGC	CTGGAGGTGTGGTCGTTTAC	59	164
BlonOBP29	TTGTGTATGGCTGGTTTGGC	GTGTGTTCAGCAGTGTCCTT	59	155
BlonOBP30	TACTTTGTGTGCCGTTGGTG	CGGCTTGTCTGTGGTTTTGA	59	170
BlonOBP31	GTCCAAGAAACTGGGCTTTCA	CCACTTTCCTCGCGAATACA	59	186
BlonOBP32	AGAAGAAACAGCCAGTCGGA	CGTTCGCAATGTGATCCTGT	59	238
BlonOBP33	GTACACGACACTTGCCAAGC	TTCGCACATTCGGTCACAAG	59	233
BlonOBP34	CTGATTCTTCGTTCCGCCTC	GGATTTGGCTCCGAGGACTA	59	245
BlonOR1(Orco)	CGGTGTCGCTCTTCTTCTTC	AGCCATCGTACCAGTGACAA	59	227
BlonOR2	TGTTCCGGCTCCAGTGTATT	TTTGAAGGTCGCCATGTTCC	59	155
BlonOR3	TGTAGCGGAATCCCTTTGGT	CTCAAAATAATGTGTTCAGCGGA	59	156
BlonOR6	CTGGTGGCCTTTTAACATCGA	CCACAGCTTTGCCGAATTTT	59	227
BlonOR7	GCCAAGAAGTACCAGTCCCT	AACAGGTGCCCAAGTGTTTG	59	225
BlonOR10	TGTTCTTGGGTGGTGGAGTT	GTTGTTGCAGTCTACCCACG	59	212
BlonOR16	TGGCTGAGATGCTTCGGTAT	GTTCTTCCCTGTTCCCAAAGT	59	154
BlonOR17	ACAGTTCGCTTTCATCTGCA	GCCAAGTCTCTGCCAAAGTC	59	156
BlonOR21	CCCTGGTGATGAGAAGCTCT	ACCATTTGTGCCGCTGTTAG	59	228
BlonOR22	TTTGACGATTTCCAGTCCGC	TGACAGCCCACTCATGTACA	59	243
BlonOR24	TGGAACATATCGCCGAGCAT	AGGTAAAACGTCAGCAAGCA	59	230
BlonOR26	CTCCAAAAGGCCAGCTCTTG	AGCACATAAGTCCGTAGCCA	59	195
BlonOR30	CACTTTAACGTACACTGCCGT	GAACCATGATACGCTCGCAA	59	217
BlonOR31	GCCAATTTGCAGCCAGTACT	CCATGGACAACTGTAAGCGG	59	200
BlonOR38	ATGGCCGCTGTTCTCATTTT	AAATCACCATTCCTCCAGCG	59	181
BlonOR48	CAGGGAGCGGTTTTGGAATT	ACTAGGCACAGCGGTATGAA	59	159
BlonIR8a	GCAATCGGATGAACTGGACC	CACCGAAGTTGTTGAAGCGA	59	160
BlonIR21a	TCTAGACTACAGGGAGGCGA	CTGTGCCATTGAGTGAACCC	59	153
BlonIR25a	TTCATGGACAGACTGGGGAC	TTGGGGTGTTGTCTCCGTTA	59	196
BlonIR31a	GCCATCAAGTGTGCCTACTG	TGGTTTGGAACTTAGCAGCG	59	231
BlonIR41a	GGAAGTGAGATGTCCGTTGC	GCACCGAATCCCATATCTGC	59	164
BlonIR68a	TAGCAAGACCAACACCGAAG	TGTGCAACTTTCTCAACTGCT	59	190
BlonIR75q.2A	AGTTGTGGGAGACTTGCAGT	ACCAAACGCTCCTGTTGAAC	59	191
BlonIR75q.2B	TGAATCTGATCGAGCCCTGG	ATTGCTTCCCTTTGAGTGGC	59	158
BlonIR75s	CGTCTACATGTTGCCCTTCG	GCCCCAAACGTGAACAAAAC	59	192
BlonIR76b	CCTCATGAATCTGCCAACGG	TGCTTCAACAGGGCTCCATA	59	203
BlonIR93a	CTAACCGGCTTCTTTGGAGC	TCGTATTCAACACCCCACGA	59	221

Supplementary Table S3. Amino acid sequences of *B. longissima* and other insect species used in phylogenetic analyses.

Gene	Species	Annotation	All number	Selected Genes	References
OR	*Brontispa longissima*	Transcriptomes	48	44	In this study
	Tribolium castaneum	Genomes	111	92	[1]
	Megacyllene caryae	Transcriptomes	57	48	[2]
	Anomala corpulenta	Transcriptomes	43	41	[3]
	Ips typographus	Transcriptomes	43	26	[4]
	Dendroctonus ponderosae	Transcriptomes	49	32	
	Dendroctonus valens	Transcriptomes	22	8	[5]
	Colaphellus bowringi	Transcriptomes	43	31	[6]
	Ambrostoma	Transcriptomes	34	26	[7]
	Phyllotreta striolata	Transcriptomes	73	43	[8]
iGluRs/IRs	*Brontispa longissima*	Transcriptomes	19	19	In this study
	Drosophila melanogaster	Genomes	80	80	[9]
	Tribolium castaneum	Genomes	35	35	
	Ambrostoma	Transcriptomes	20	20	[7]
	Phyllotreta striolata	Transcriptomes	49	33	[8]
SNMP	*Brontispa longissima*	Transcriptomes	4	4	In this study
	Drosophila melanogaster	Genomes	2	2	[10]
	Tribolium castaneum	Genomes	5	5	
	Ips typographus	Transcriptomes	3	2	[4]
	Dendroctonus ponderosae	Transcriptomes	3	2	
	Dendroctonus valens	Transcriptomes	4	3	[5]
	Colaphellus bowringi	Transcriptomes	4	4	[6]
	Agrilus planipennis	Transcriptomes	1	1	[11]
OBP	*Brontispa longissima*	Transcriptomes	34	34	In this study
	Tribolium castaneum	Transcriptomes	50	50	[12]
	Anomala corpulenta	Transcriptomes	24	15	[3]
	Ips typographus	Transcriptomes	15	14	[4]
	Dendroctonus ponderosae	Transcriptomes	31	31	
	Colaphellus bowringi	Transcriptomes	26	26	[6]
	Ambrostoma	Transcriptomes	16	15	[7]
	Phyllotreta striolata	Transcriptomes	32	32	[8]
CSP	*Brontispa longissima*	Transcriptomes	16	16	In this study
	Tribolium castaneum	Transcriptomes	20	20	[12]
	Anomala corpulenta	Transcriptomes	6	5	[3]
	Ips typographus	Transcriptomes	6	3	[4]
	Dendroctonus ponderosae	Transcriptomes	11	11	
	Colaphellus bowringi	Transcriptomes	12	12	[6]
	Ambrostoma	Transcriptomes	10	10	[7]
	Phyllotreta striolata	Transcriptomes	8	8	[8]

References

1. Engsontia, P. *et al.* The red flour beetle's large nose: an expanded odorant receptor gene family in *Tribolium castaneum*. *Insect Biochem. Mol. Biol.* 38, 387-397 (2008).

2. Mitchell, R.F. *et al.* Sequencing and characterizing odorant receptors of the cerambycid beetle *Megacyllene caryae*. *Insect Biochem. Mol. Biol.* 42, 499-505 (2012).
3. Li, X. *et al.* Chemosensory Gene Families in Adult Antennae of *Anomala corpulenta* Motschulsky (Coleoptera: Scarabaeidae: Rutelinae). *PloS one* 10, e121504 (2015).
4. Andersson, M.N. *et al.* Antennal transcriptome analysis of the chemosensory gene families in the tree killing bark beetles, *Ips typographus* and *Dendroctonus ponderosae* (Coleoptera: Curculionidae: Scolytinae). *BMC Genomics* 14, 198 (2013).
5. Gu, X.C., Zhang, Y.N., Kang, K., Dong, S.L. & Zhang, L.W. Antennal Transcriptome Analysis of Odorant Reception Genes in the Red Turpentine Beetle (RTB), *Dendroctonus valens*. *PloS one* 10, e125159 (2015).
6. Li X.M. *et al.* Candidate chemosensory genes identified in *Colaphellus bowringi* by antennal transcriptome analysis. *BMC Genomics* 16, 1028 (2015).
7. Wang, Y., Chen, Q., Zhao, H. & Ren, B. Identification and Comparison of Candidate Olfactory Genes in the Olfactory and Non-Olfactory Organs of Elm Pest *Ambrostoma quadriimpressum* (Coleoptera: Chrysomelidae) Based on Transcriptome Analysis. *PloS one* 11, e147144 (2016).
8. Wu, Z., Bin, S., He, H., Wang, Z., Li, M. & Lin, J. Differential Expression Analysis of Chemoreception Genes in the Striped Flea Beetle *Phyllotreta striolata* Using a Transcriptomic Approach. *PloS one* 11, e153067 (2016).
9. Croset, V. *et al.* Ancient protostome origin of chemosensory ionotropic glutamate receptors and the evolution of insect taste and olfaction. *PLoS Genet.* 6, e1001064 (2010).
10. Vogt, R.G. *et al.* The insect SNMP gene family. *Insect Biochem. Mol. Biol.* 39, 448-456 (2009).
11. Mamidala, P. *et al.* Identification of Odor-Processing Genes in the Emerald Ash Borer, *Agrilus planipennis*. *PloS one* 8, e56555 (2013).
12. Vieira, F.G. & Rozas, J. Comparative Genomics of the Odorant-Binding and Chemosensory Protein Gene Families across the Arthropoda: Origin and Evolutionary History of the Chemosensory System. *Genome Biol. Evol*. 3, 476-490 (2011).

OR sequences

>BlonOR1
MMKFKVSGLVADLMPNIRLIQGSGHFMFNYHADNTGALHSLRVGYSCMHLVLCLFQYGGIFANLVLERDDVNDLAANTITVLFF
THCITKFVYFAVRSKLFYRTLGIWNQSNSHPLFVESNNRYHSLALKKMRTLLISVMATTILSASAWTAITFVGDSVHHIKDPDNPN
ETITEEIPRLLVRSWHPWNAMSGTAYYASLVFQTYYVFFSLAHANLLDSLFCSWLIFACEQLQHLKEIMKPLMELSATLDTYVPKS
ADLFKASSATSQDNLLEHDYNEKNEELNLKGIYNSRQELGGHFRTGTLQTFGGGGVGPNGLSKKQELMVRSAIKYWVERHKHV
VRLVTAIGDAYGVALLLHMLTSTVMLTLLAYQATKIDGVNKYAATVIGYLVYSLAQVFHFCIFGNRLIEESSSVMEAAYSCHWYD
GSEEAKTFVQIVCQQCQKAMSISGAKFFTISLDLFASVLGAVVTYFMVLVQLK
>BlonOR2
MDNNDVNHFFKFQKYIMLLTGEWDFRHQKNNFTYVYDKVKYLVLIGFSMTPPVFLMSVFFTKNCKDIAAENFFNFMHCGVVS
MIAILLHSKATKKIKDEIYNFENFVLENESDQVKEIYMRAARENMFVGILYTVLALPASILWFVNGRTQKYGERVGCQIHEGVTFQ
IYYFFDIEHRMWVPSIFDSITLVPAPVYFVYSRVMPTSMLFFMLSQIKIFIEKFRQLDKDARELVSREHGDLQKVIEALLKKHIQHHI
KLISLMQTIEESCRSIMFILFFANAINLAIFAYQLMFGDPHLFSRNIGPFFLILMQSYISCNVANDVILYSEHICDIIYNDFDWITYSKE
NKSLLLIIMARCKHPLSIKADIIGEMSVETFKSIMRMCYTTTTYFTSM

>BlonOR3
MNEDLAEKIALGQYSLLILRYIQVIPKNILKENFGKLYYIKYALIYCNPFIIVGGMFSHFFIKLKERSYSNIDLDMVYIFHWTGILFF
NYSYFRRIKLTTILYRKLSDFKTFGIPTNFDETNKKLNNLSKLHHLYILVVIMCFSFMPLTETKNCEAGRVANTQALCGVAESLWFP
FNINFFPVKHLYIAYEIVCNFFVITSASAISYSIAESAEHIILRIKHLKILLIESFLEQNEATRLYKFGKAIQYHNAIIEVSKLMNKCFVR
CMFLHGIYTSVIIGCMGYRLSKSFSLSIMSLWTGWVTAMSMIAFSGQRLIGESTSIGECVYNFPWYNLDCKFQKNLSLVLMMCQK
PIFLDVSPFGPMNYNFVFTILKTSYSYITVLKNTGSH
>BlonOR4
MATIEYPQKFFYTNEWIYHLAGIWLYNPENISVFRRIWQVIWTLLVYVVSVYFLILEFLIFRETIHDIKKFFSQFGLLLTHVLGIVRV
GLLLSQHQRLLKLQKSLQDEEYYYESCGDFVPGKLMREAKTLSSRFSIMLYTLYSLVGVLSFISTEIDIELKIEEERLPNNITCYDY
MPYYFVIPFDPTTKTNCRWANMLMSYNLSSYAWFISCHDGLLAALLNCLKTQLVILCGAIRTIRPRILTRVNLPVNLEVHQDDEFP
KLEEALYEELIHMNKHLVVLLNAADELEDIFTYMTLGQTLASLITFASCLLVATSVPVTSPTFFSQTEYFVCVFIQFSVFCWFGSGV
TIASEDINLSLYNSNWYSASKRFKSALITTMCRMQKPMYLTVGKFSPLTLTAMVSVCKGSLSYYAVFKSVGN
>BlonOR5
MEVYDFSACFKTEKRILNALGFYPYKLRNLSYSKIILRYIISISLLVNMIQMILALFYVSFDTFISYSMIWFTYYAFFLKLASFMYYG
QTLLDLEKSLKSTFSLKIPESYIQLFQNDWKFADRLASFTRLSLNIFTTYFCFYMPFSDSTNIHLLYPGPQPCNTKEKVCFFIIYTTE
VLFTYVSVFININLECMYSKLITICCCLFEVVKQNFINIDFNNNEKATEDLKINIALHIEVIRFAEGIDKIYNKIAFFQCFTSMFVICLI
CFQITLAPITSAKFWELTKYSLDLASEVAFYCIFGNKIIEKSDEIREACYFSNWYESNLILRNSLIIVMERAQRPFHMNAGGFFTLSL
ATLTSIFKTAYAFTRFLSNTIDVEME
>BlonOR6
MSPKSQMHTNIIILRYTLGVLRGTFMLPRNLTEANFGKKYYVNYVLVNISAMGLLIGTLLHCWKNIQDNTYINFDLDLVYISHWS
GIYYFNYCYFVKVKLSIKLYKILSDFRTFGIPINFNETNERLNKFSRIHHVYITGVVAGFTLFPLLEAKECHAKNLKNNLNELCGIV
GTAWWPFNIDFFPVKQLYLMYEMYCGYMLVITASGVTFALAECTEHVILRLKHVKFLFIESFQENNPYLRNEKFGKAVAYHNDIL
GIAKLVNTCFMRCMIMHGICTGVILGVIGFKLSEGFSFAAVSLWIGWVVSLIVVSICGQRLINESVSLGEELYNFPWYELDYRFQK
NLIPVIMRSQKGTSLDFSPFGTLNYQFIMAILNASYSYITLLKNL
>BlonOR7
MPEDDQYPVIRLMMKCLQIQYLWPKSGEKCTPKFYFILYSRHLLTVPLILGLIAKKVEIFHGTGKFQFDGDFLVIFTQTGLVCFILA
YGGIVDELAKKYQSLLELNSYGVPEDFYNFNNKMDLYTKIAMIYSQWTILLAVASPLLDYKNCMNREENVYTVCGVTNTWAPV
DITVFPYNLITYIITCYSYFSIYSSILTITFYAAIISNYLTFQINYAKTLFGKTDKVRDYTVKKTLLIEAIKYHNEILRLAQQILDTLSNR
VNFLHMVQTSVVLGVSIYGNMKEFQLVYLALCYGYFCQLSIQCYCGQKLMEECEDIGRMIYTETNWYEWELDLQKLVVIILRRS
QIPVKTRAGPFGYLNYENLINVCKAAYSYLMFIESGTT
>BlonOR8
MYEQEFKYVFRLFGFVGMHPMKNMIKPLVIFNFILTMYVTILIILRLLLQTDLLVVESVGVFSQVWLKFIILTLKRKEIKQVLDDSQ
EFWKDDVPGSENKELLRYLNKGMKIFLTYIFLSTCMFLFKPLLVRGTTIYYYYDIPQIPFVVSYAIEFYVTVVTMAMVIACNLFISV
LIVIGAGQFSNLNATMRMLDLSKATSPNNLNLCLEEMKQSIKYHSFLIQYVQRINDVFKILFGVLMAIVTSLLCMNMYVISMPNT
TLVDYVRAGTMVCALTTEFLLLYGVPAQKLLDEAEEISNSAFYHCEWYLPNIIPVRKKLLMIIQRGQKPVSLSAGGLIDINRQTVIS
MLKTAYSFFTFLQTMETTDVKV
>BlonOR9
YDILPFFATKNCEIHRTQHMRDYGIPCKVIVRYVIPFKYDYSPYYELVIAEQVLVASLGTIVVMTVTMLVCGILTHIAINLRHLKTMI
SQISMSDGDELENLVKLCVKYHTIILDIADETNDAFSIMMLVHITWTSFIISVLGFEIVTEPNFWNSLRFLMHLGGWLLMLFVDCF
YGQIVINESSDISQVVYDTKWYEKSPKIRRYLLLMLLRSQKPVVLKAASVRFMSLSTFLGVLYSAYSYFTVLLKIKP
>BlonOR10
MTRDMTCPPFDYTDCFPHHIMIYKYFGFWKPTKNMRFKFLYNFYKNICIAIWIMFLTSQVIYMVINKNNINEVTATLSIAITFTVILI
KMLVLYSEMDRIQSFIRNLNQPIFQVKNWRQHRIAEEGKRYCLWYFYLCVFFGGTTDVFWCLAPFLGKTREPYEKGWYPYDWE
VSPHYEIFYAFQGFSCIMNSCLCVNQDSFYATLTTQVSLQCELLCDTLNHLDSFQTIDGVLFQIEESSMLLMRSDREVFSNEMTKN

LLVCITHHKEILRIANDIMEIHYKSVYIMFLGGGVILCCSLYQIYNVEFGSIAFLRILFYIFSMLTEQFTYCWFGNEVIVQSAKINDAV
FNTPWVDCNNKYRKIALQFMTKATKPIIMSAGLLEISIETYLLILRTAYSYFTLLKKIGQDQRDQNF
>BlonOR11
MSELNKNNTYHLSLTFLKWFKIYPDKTKRNSKQFYAKKALLPLATLVLTLLNAVHLIVVLQGNTDADFSEDLTFLSSGSGLLITCV
IFAYNHKKWSKLFYKITNFREYGTPRQLQKSLNHNRFFSRIYVFYCFTGYALYSLISLYKSKCQVIENRFTFYSAIVPIALPTVFTSQ
SVILINFLYQFVTIGATELSGAMISLIIWETSQLIALHINHFKDMLHSNITVRNISKHKQMLKVCAAYHLQIMRFSNDLNYLTMICFG
HISLLAPINLAAIANQILKSNPIGAIINLTAWMVGVFLLCDAGQRITDENESVGNMIFYNLRWYEMEGDLQKKFLFMIMRTQKPIY
LDALPLGSLNYSLFHKILKTSYSFFTLLLQAT
>BlonOR12
MTIFPKNEHLKIPMYTNAPMGVWPFIFEDNPGWKKWYDVYSKVIFTYFFLFIITSYMKLVELLLADTINFDEVFSNLVTTLLHSAT
ISRVWALKTDRIKILVKEILATENKILTSQDPQITKIYRSFAYQSQITNVVFVVNILIITFLYFVHPLLIDNEVVFDEKTNTTKIIKALPL
TSWFPYDPQEYYVQTYLLQLLDGLVGASFIMCSDIFAFSLIIYPLGQINILSYILDNFQQYALKLKDQLNCSYENGSCIAVRECIIQH
NIIIRYVNEFNKAMRNVMVLDFLQSSLQLAAIVIQLFVVQLTLPNFIYFTYSVLTILIRLLLYYWYANEILLQSSEIARFPSKWYDES
PEVKQMMHVIMIRSSKELFLEIGPFNKMSLGTLLIILKATYSYVSLVYSG
>BlonOR13
MYPKFNSASKVTEYVIRALIFAGFFNGGIVISELINCYKSIGNLEELTKGSFLTLSNVIALYKFYILLRYQPKIIKLINSLNRNEFRPKN
SKQRQILTEYIKLSKNMTKTILTLCSLTCMFWIIYPFTSGDEKYILPIPAWMPFNTSGSPNFEIAYVYESVGTIIGGITDLNSDCFMAA
LIMVVSAQLNILNDTFGSLLKDAELGNSATFKSISKETQTNVNKILERKLLECICHHRLIINFANDCMWLFSNVIFAQISISVVINCF
TLFQLTLVPVASLQFLSYIFYESCILMEVFLFCYFGNEVILESYKLTNSAYHCEWVNASKTFKQNFIFFMTRSQRMLTCYAGGYFAL
SLATFVTILKSSWSYFAVLIQVSGKKNF
>BlonOR14
MDKTLKYNFQALFETEKKILDFFGFYPYQKMRSRKVLYKKIKTTCMLLSLMIYIGQMFLGIYKDFKNMIEPCMILVTLFGFMCKL
TVFMYNGENILKITSKLESSLFSEIPKDYLSGIKEDANITNKVTNIYRFIIASYLLYECVVKPLLDKSVVLLFAGYAPCTLENISCFAL
FYIFVAINGYISALTNSGIDCFFCKITTICCILFDVVQQNLKNIDYRDSATAKKVLRRNVIQHIEIIRYAEAIYHIFNKIAFFQCFTSTFSI
CITLVQMSLIPTFSFEMFVHFNYLMSILVQLAVYCWYGNNIMIKSEEVSTACYMSNWYEESVNLRKTLIIFMERTKRPFIMKAGGF
FTLTLSTFTSILKSAYSFLRLVSDLYD
>BlonOR15
MENVFDFQEIINLNLHLHYFLGNLCPKFDSKSKVIEYGIRAVIFVGFFNAGIVMSELMNCYKNLGDLEELTKGLFITLSNLLAVYK
FYILIRYQPKILELINDLNRDEFRPKNPNQTKILVEFIKLSKNASKLTLVLCCITCLIFTIYPFTDQDEKNKFPIPAWMPFNTSSSPNFEI
AFVYEAVATVIAGITDINMDFFIIALIMALSAQLNILNDTLQNIREDAKLECFDSIKSTPEEQRRNVNRIMERKLFECVRHHHLIINF
ANECNWLFSYAVFVQMFVSVLINCFTLFQLTLIPIDSFQFFAYLFYELSVLTEVFFFCYSGNEVIIQSYKLTNYAYHCEWIDTSPAFK
KNLLFFMTRSHRTMEFSAGGIFILSLDTYVTILKSSWSYCAVLMQLSDQK
>BlonOR16
MEKTFTKTSQLGKSCSIKWRERKELSFNFAEILSLNLQWLRCFGILLPGKDTHFCLRFLRTAFFIGFTYTSMILSEFINFALNFGNRE
ELSSNLFLFLTHFVQLKKYYQIFCYQSRLKCLIATTQKSEFAPKTAKQRHILQKYIKMSKLITTVFMVACIATCLLWGVYPFTDPNI
RLPLAGWFPWETNSSPNFELTYTYQIIAATLNGLTNISIDTLISGLLMVVCSQLHILNDSLENIQELALLELKKVVVETKKDKDML
NEILIAKLQECIQHHRTILKFAKEVSFIFSTSIFGQFVISGIIICLTLFEMTLLSVGSIKFMSFLLYLYCMLMEIFLWCYFGNEVTYQSA
RLTNSAFHCGWYKCCKKFRRNLLFFITRSQRRLNIYAGGFFALSLDMFVRILKSSWSYFAVLLQVNEITTK
>BlonOR17
MTNLLATFRIDKTLLTIGGFYPVQKIRYKTLYYLSRTFNWGLSFIELVLMLAYIISRISYLKEISENLFFSVTQFAFICKLTNFMFHGK
KLRNIEQQLKQKRFTQLSLHEEKLVMDLIDFGRDLAKIFRVLCFLVVFFYALFPFFDKNSDGSMKLPLPMSFPFKPEDYYYPIYCA
SIAAIIVAASTNSNIDILTVMLISLGTGQVEVLKYRFENLIPPGLKEPEEIVLEKLKENVRHLDDIYRYVNSVQEVFSNGIFVQFACS
VVVICMTGFLMLITPMTSIQFQLYVVVYFSCMMCQIVLYCWYGHMIMDSSDGLTDACYRCCWYESSLKVRKALIIIRERTKRQIV
MKGGNFFALNLQTLMVILRSSYSYFAMLRQVYVKNEIK

>BlonOR18

MQRIEFYDAYHMERGVLSFFGLYPEKSSKLSFWKNVRKTLCLLSYHIHLIELVINSFNQSFEETPFSVVWVSEIGFLLKLLTFLQHTETLLHFEDMLDQEIFKNVPNSCFDSVSKTIEFVKIFTTSKILLLLLFVSYISCYVPLVVQSGKRHLPCDPYVPCSLENDFCYVGFFIFHLANCILAALLDTIFDCLYCKLTTICCCLLDVTYTSLEEMDYGDSNESLKLIHHKAILHVKVLKLIAVVDKLFNKIAFIQCLASIFSICSSLLRLTAVPVNSMDFFAIFNYFMIMFVQITFFCYFGNNIIVKTDLISQACYMSNWYESKMEVRKVLFIMMEGSRRPFVVTAGGFFTLSLPTLVSILKSAYSFWRVFSV

>BlonOR19

MMVHDFVEDYYLERTILCCFGFYPERTIKVWTWRKMRLIVSVIMLFIHEVQILMHMLYENFASARSSVLWVTEIVFIMKLITFWYYGDIILAIDNLLRNPILSNYPKSYNFVRRNIQLPKIFSKIDYASVTFFIIIFSFFEPFIRQSGERHLPSDAHVPCNLNVDICYYGFYAFHTINFYITGFINVGMDCMFCKLATICSSLFKILYYNLISINYENSKESTELLKENVCRYIEVIKLSKYIDKIFNKIAFLQCMGSIFTICLTLIQLNRAVVGSLIFFQVTSYLCSMFLQITIFCYFGDKIIEESDRISNACCLSNWYEADIFTKKLLLMLIQGTQKPFTITAGGFFPLCLGTLVSILKSSYSFWRLYSAVYN

>BlonOR21

MVTSQDTKRFFDFNRWCLRILGMWPTESDSKVVRNLHYFYNKFILLAIMYFVFADWICIKDNLESDMFTIYTIFTVALIETVIVIKDIYISFNKERFDRLLETLESKEIRYEAFEEKAYYPKELVRQCKRNTKLVMNYLVLTCCGTFISSFLPATIDIIYMAVDKEYPVPTKLPFFMWVPFSADTRFKFIMAVLYQAVETFYHAVGVIATDTLYINIINCITFNFVIIQGAFSTIRERCANRLHGVPLLEPNGLSNSAELEREMRREFKKILSHLQKIYWACEFVEEIYRIIVLLQIIAPMFILCTQMYLILLTPPGDEKLYMELFLMSVMIMQVSFYCWYGNNLTIEAAKIPSAIWHSEWLEASTRFKKDLVFVMIRANRPLYLTAAQMVNLTLETLMKVFKASYSFMAVINS

>BlonOR22

MSLEETGFLKLLNKEKKMLFYIGAYPDLTTCNKHLQLLTRTFTLTVQILGYATLLLNCITNFDDFQSATDSALVFLIMTPIYTKLYRFSAKKGKLVVLENILRNVHIDLNYDDNKMVLKKTKKTCVSYSFLYTTAMYMSGLSNLFLPLASDDKRALPLATWVPWTTENLILYIITFIWHLLIISNGIGTNVVIDVATNKLIIIINGMFEVLKNDLNRIDYTKRDGSVEMMLKKCVIMDNQIKKAVRNIQEIVSIGICIQLISSILIICFNGFKLLVTSPLVFYFYIFLLYLISMLIQVSAYCWFGHDIVKNTEEINEAIYTSNWYESDLKARATLLTMMVCCHKPLILSATSIFPLTIATLNNIMHTSYSFFTMLRTLKP

>BlonOR23

MKLRSSKIISICLIMMTIIGTDPRKMHSKFQFFVWLLNITSLLIVILRGLVTLIFNDGLDFNLVSGIMEFVFLMTHVSTNLITLYLKMKNVEHLLDLTDNFWNIEDFSADEDMIIIQNRVKYFQWFVYYFLCVGILTAFIGYFYKPFFTNSRVLIYDGSVPENDILYYMLLMLETYSMWITGFANISFDLFFAAMLMFASAQFRMLGIEIRNLISSKVHDDEKRIVIRQRLKKSIDHHNFLLGFVDSLNDIMRVPLLVYIAVMVLSMCMELFMMTTYSKRDLFKFVQVGFYILDLLNEFIVCLCISGEILTMKAQELLNYIYNSDWYENSAISKDEITIMMMGAKKPVRLAAGKILNINMETCMATYKTVFSYYMFLVSVSTEEN

>BlonOR24

MNVYMEICLKLLSMIGVNPLKEKDYVQLVIFIIVTFILSFWVYLNIMVPLSKEGSLKFTDYTETFWNISPSIYSFLIIWTLFSGKNKLSDLLDRTDRNFWRVDQFVITEKDKSFCNTCESRIKWAFYFLSVIHISCLLTFYLPPFFAEGDVLAISCYRPPWMTLFQLRVFQGAQVAITQMLPSQTILMLLTNLMNLTQIQFRLLNVELRNIFHVQQKKFEIQRKVAEIVCHHNFLVRFITDMNNIFSVFLVGYLMSLTVWITIDMFSLTIYFSYEVFGLSLMCIAALLWEFVVILCIPSESLTNEIARIPDSLYFSDWLDDTGFQKEVLMIMINGQKPVAITAGGIVDLNMRTALETIKGMFSYFTFLQTINVSAQ

>BlonOR25

MDNIKHQLKKFTPHDFFLWMKKFCLLVGLLPPKKRVLRYVYLVIATSYFILLFLGLLSQWSAIFINSDQKNVAHMMVRISMCIYCVIIDFRVIMWLLLKKKMTVIVGIIRRTSFNFDCFKICRISCNNLKKVAMEETVNIRLAKYYQFISNLYQQHIKLEFESTNNSLEEFRKTSKVHTTLFCCTIYIVLVCLTCMSSLLLFATEYTLEPYEKFDVKRNKTVIYRQSKLDLNVPFDTSVSDGHHLFVFLFEILNIYARTMSFLPIDVLLCGTLIHLISQTVVVQEAIKKIPQGIAYDENTENMLLLQKIRIVKCINEIQEIYRAMEKVEELFSIQFLITYTLAEILLCTLLYSITLVQDMKEVLSLTIMITACLGEIFMYTFYGQTLTLELQKIATNVFDLDWINYPPALRKDLVFLIKRVQNPFHITVGKLFHLDLTFFMGVLQKSYSFYTLITNRRN

>BlonOR26

MSLIEDGVKQIGEDEKPKEPEIVCMPTSIKVFRIYGGFPPKGQLLNPGKLFYFRFVITAVYSSLILVGSILHLVQNIKDNVYHHVELD
FTYIISMSAGYGLMCSYFSHVKASVQLYLFLSNFDEFGKPLHFDEINKKYNRYSIYHCYLESIVIFILLSSQMFKTKQCELDNEKL
HIHEVCGLFTNTWMPFNIDYFPIKQFYLFTQLFGAHYVYMFAGLAAWMVLESVEHIATRIRHVTYLFSEALKEPDNKLRREKFNL
AVKYHNAVLELENMLNQTFSVFMFTHMVMTGGIMGYGVYAYIKGKNLSPFLLAIGWLVGLLLDCKGGQRLQNASAGIGEALYN
ADWSECDNEMKKDILFVLMRCRRYMVLQAASFGKMDHPMFLAVLKASYSYVTLLSQSDNKRK
>BlonOR27
MTTKDTNMFLSILKYFMIISGQWNSRNKNSKLVVIYQKLRYVFVFVYLLTPVCSGMTIVKAWECKKIVIENFFNLMYSSVVVILV
ELLNSKETKKIVDSIYYFENVRLKSLSVECQKIYAKATKKNNQMVIYFYVLALYAASSWFYYGRSIEVEIEEVCGVSNGMSFHIW
YPFDIEKHKWVSTLFDATSLSLATVFFVYSRAMPISLILSVVPHLKMLQVEIKSLDARASVRTEGTNIQQVKTMLIKEHIRRHQEIIS
LMDDLRGATKTIAFFLYFGNVLNLATYIFDMLFGDNTLFLKNLGIFILTISQVYVFCNSANELIIHGNKVSDVVYNDIEWISCNKEN
RFLLLLIIQRSQRNLALKVNIIGEMSMETFKTIMRLCYTTTTFCASVYQ
>BlonOR28
MIYYYGNRIRNVLREIWEKFWPVSVDSEVLEIVKGRYSIVLNGIVAFIVSTFIFVIGVAVAPLLLGNGTALPYPTVYPFDWTVNPVY
PIVFTLQTICDVLIPVIVLGYDFLFFSLCFTITAQYLCLHRVIRKLGTKEMGSVIQKLGYDSDEIPDNHDDISKLFIKIFIKQHVTLVRI
TQEIEDIFNIAAFLQLFNSMLAMCISCLITTMADTDMMQIIYLFSALLAYFMQLAIYCAHGNELSYQANLLQDYIFGSNWYEIESV
KTKEQVILLLLRGSSATVKLTALKFFNINFATFIQVLRVSFSFFTLLSTVTN
>BlonOR29
MAVENINYAHYFNYNITLFKVLGFWKPDENMRFKKIYKLYTILCLIIWATFLLSQIKLIYEKLDNVVELTGMMYITGAFTMILIRIIS
VYLKMDLIKELLNNLQQPIFQVKSEEHVRLAKEMEQSMKSVLFIYVYFGLQSYISFSAIPFLSPEKTTLTDGWFPFDWTVSPYYEL
VYVFQNFVTVLNILICLNMDTFTFGLLMFIGLQCDFLCCVLNSMGDFESADENVEVVETSGRRCIVNEKDTKTVSREMLKKLIVC
IEHYAEIKRFISKVENIYETSALILFIGGGIIICSGVFQLSVVKFGSLESFMLLSYLSCMLIEQFLYCYFGSVVMYKSYNIFLSTYNIPW
LKCDNNVKKILLQFMVGTQKGLTLKAGNFLTMSIDVYVMILRTSYSYFTLLQNFQE
>BlonOR30
MTAKDTDKFLGVLKYFMMVAGHWRFRNEKNKFVSFYEKSKYTFLIIYMYSPVIFVITIIKMWKCEEILTENLFTLTYTAVVVLVII
LLNSEPTRKIIDYIHNFEDVELDSLSEECQVIYQKATDKNNRMILFFFFVAALASVSWFYTGKSFEIETTEYCDFASGMSIHIWYPID
IHKHMWVCNVYESVSLSLATVFFVYSKVVPITMLLLVVTHVKILHVQLKDLDGKVSALAAKENLHIQRAKTILIKEQIRKHQEIIS
LMNEVQRAIKLIALILYFANVLNLAAYIFDMMFGDLLKIIKIGGAFLLTVAQVYLLCNSANEVIYQGSMISDVVYNEIDWISCNRE
NRFLLYLIIQGSQRGLAVKIDLIGDMSIETFKTIMRLCYSTTTFLATMYQ
>BlonOR31
MENKIFLEYYNKDFLSIKIFGGFTLIANDPHNKYWMIYLMFVNFFFPLLVNMAQVINLFQLSDLIKLVSSGYIVAIACMGSFKSFLL
FKNRVQLAELMNLLKNEKFLPKNNAQRMIVRKELGFHKTVKTVLLTICTMSVMASVITPVFNYQDRRLPFAAWYPFDITPMPIY
VLVYIHQSITDFYNTYMNVYTDIIIAGFTTFVGIQCDILCDDFINMKTEEGELALKKCIEHHELILRFARTTEKVFSQIYFGQFAAST
AALCMTLFLLTLIDKSSFEFFYLVLYQSSMFSLLLIPCWFSSEMEKKSENISNAAYSCPWVNASQPFKKELLYFIHIVQTPISLYAVG
LFHICVETFMKILRSSFSYYTVLNNLNMQN
>BlonOR32
MQSTEKSLFLKSANFAQMLAGMWTFEGNTTVASIVYQIYSYFIRFWVVFFHLLYIVTFIFDFECKHYITETMTTFCRSVGSLLIPIL
LSSSKVQEMLSIVSEIENEIINFEEDDTVRIYNRYAKKINTLVAISTSSMCLVMFLWTVNIFTVVIDDLDPKDMCAKAEGQIYQFHLP
INVIIQKIIASFIPLIGGLMILYGRAIPTTTAGNILTHVKILQEYIRKLDKKVQEASEKSNVGVHQCRFIILKDIARRHQNIIRLTAKVQS
LNRYVVFLFYFGDSAFTASFMMIIVTTHDFSKTLPELFVVIVTLIGLYVFSLIANDLIVESTNMADIIFFETNWQDFDQKSKSIIYFM
LLRSQKHLAMSTPIIGDMSLGTFLQIIKLCYTIQTFFVTQNE
>BlonOR33
MAYFYTIEKCLKYLTIVGIHPEKGYAFPQIILFVVNLVGVNIMFTLVLLNFFYNNISKTVTNVTDAASYLLLFAHGLLKSSTMFLTK
QRLLKLIKAQEKWNHDEYTDDQKIQKYYKVIKLTFFFILGMHMINVVGFDVIGDGTLPIECYIPNWLNFYTLLIIQHITAFQSIVFP
VVTMDIIFMSILRLTQIQFRRLNLEMRKMFDEHGGVKKNIISERLRTCIRKHNDLLRYANEINDAFSKSLLIFLVIIVLSMCVEMYVL

TTQFDWLSYRKALTYTATGCIQFMLCYCYPCQGLMDEIEDLYEAIYFSKWYNHLDSKETVIMLIRGQHKFLIRPGGLIHMNLPTG
LTTLKTMVSYAMFLKSISKTENALEK
>BlonOR34
MLKNQDYPILGFIIKTLQVQLAWPKSKNVKSSFYILHSLKMLLGVPFIVGLVVRKIQVLRESGNFFLDSEFLLIFTLSVLLYSGFAFS
YIVEKIMDFYDATFELDSYGIPANFPEIKEKIDFYSKIFVVGLEFSIFLGLTSPLLEYDNCIAKNNEYFTCGVANTWAPFNITVFPYRE
IVYLVNAYTQFSFYSEVCSLNIFAACFSIYLTFRLEYAVVLFKRVNSERNSINKRYLLFEAIEYHQKITSLTHNILDAFSPTINYMHM
MQTGLILGLAIYGNVQEFNTVHLTVAYGYFCDLYLQCYWGQKLMEQGENVGARLYTETNWYEWEVDLQKHLVIILKECHRPIK
TKAGPFAYLNHENFINVCKTAYTFLMFLETVGI
>BlonOR35
MQFSNIDMCYKIFYIVGLHPEKGRNPLQLLWFFFITLNLWYEMYLEVLLLVETEHSIAEIIQIGYNITFNFYGVLYMTVLFLKRDGI
SKLVYDAKEYFWKIEDMNITEVERKRFIKKHKTIRILFFTIVSFYVMGIISFFGPSLIAKDIFSPFPSHQPAWMSNSLLISTQIFVGVFA
EIIPLVSFSAFLMSIIVLIHQQFIVLNKEIERLLGYGNRLEIGELKKLLDYHTFLLRYVEDINSTFSGFILGYVLTITVSMCLQMYSISE
MASLEECFKAGLTILAVMLEFTVFLTIPAQELSDEIEKLGDVAYFSDWYLHKKLVSNVLMMIMNSQRKVAVKVGGLIEINVITGFQ
ITKAMFSYCAFLRTVSLTD
>BlonOR36
MEHISNYFTTSIKSLTLLGIWFDPKTFGKKSILVTIYILFVNICFIFMSQICHFIYMYKARHNVVDFADEFYISLSSLMVVFKDYSLIK
NFDKIQAMLKDIDSDIFVPKSRKQRSIIYQTMKTWHLIYWFHTFSCISFVLLYLITPIYNSYKQNTRILPLVDCYPFNIFVTPVYHFM
YAYQCGVVTALVMHGVHWDQFLIGLLFFASGECDILCDKLRNLKLGETMELEEHSEKLIECIKHHKKIRRFLQDVQYCFSAVLFE
LYCCSFITFCTVMFKLSLAEPFSQDFIILVIYQTALFIEMFMFCWTGTNLTSKSEDIRLAAIECDWLTASKKFKTNLVLFLQSTQKPF
YIKLVLFPVSLQNFVQLVKTSFSYYTMLTKLNED
>BlonOR38
MTLGSTREAAFLRTVNFIMLLAGKKVAEDNRKFSTIAYKIYSYFINFYMVFFHLLYILAFVLDLECKYYFRETMSAFCRSIGCFLLP
FLINSSLRQEILEEINQNEDDITNLAESDIKQIYSKHRKRINILVGISILVMAAVLILWMIHVFMLDIDEMDPQDKCSITEGQIFQLHFI
TNTLIQKFIANLTPCAGGMVILYARALPATTVGYISLKIKVLQAYMRKIDANIQEELRNNNTEIDQCRLVVLKKIAQEHQHIIRLTK
KVQSMNRAVVFLFYFGDAAFTASFVISITTSEDISASFFDIWVVLATLIGLYAFSLLASDIIFESTQMADILFYETNWTDYDKKSKSV
IYLMILRSQQPLAINSPIIGVMSLGTFLQIIKLCYTIHTFFATMNTK
>BlonOR40
MESTIMKICIIFLNFAGIDKEKWRISQVTVFFYSTLNGVFVMVLVSLLFVYKDSPIVMTDITNALSCILVILHGIAAWITTMFLTKAK
ILHLTKLMKENFWNMDCLEYQEDKQIFIDMHREIKNKTRFLLAIYQLALLGYLAPFLTKGQNLVFDCYRPQWLSYYFILFMEEYA
CIMTIFLPINVTDFLFLIFSTMTGYQFKLLAQRFNRIYNVSDDSLSQGRCIKAGLKECIEHHIFLLEFVKKLDAAFSIYMFLYLGDFL
LCMCVEMFKFTTESDATILFKCVAYILSGFVELMFCYCMPAQAVRDEAEKIANMIYASKWYNNINSSKEIIFVINKSQKQVNITGG
GVVRLDLATGLLALKTTVSYYTFLKTIGITEKQE
>BlonOR41
MQLASDTFCVKNNIKCIMQSIDLYHSYFMERKVLSFFGMYPEKSAKQSFWKILRETIFLLSFHIHLIEILMNSLYQNFSDSPFAVVW
VTELGFVLKLLAFLQQSDTVLHYENLLNQQIFKIIPKNGLKSITNSLEFAKIFTTTNMILVLLFVNYISFFVPLVIQSGERHLPSEAYV
PCSLENDLCYVGFFIFHAANCNMTAILNTAFDCLFCKLITICCCLLKVVYVNLEEINYEDGDDSLNQVHKNAALHIEVLKLITLVD
KIFNKIAFFQCLTSIFAICSSLLRLTSVPLNSMEFFGVIHYFIIMFMQISVFCYFGNNI
>BlonOR42
MMFLIGLNVDKGRGIIQFIWYWSINIAGIVITYLAILLLLQKPDLTMKDCIDMVSSISTSIHGLLLTISLFYKHPEICDLLKQMDKNF
WRPEDISLSQEELHAIQQVYQSMKQKFYGIIFLCSTSYLAFDCQTLFAGEKVLPFPGYIPSWVTFNQMMFYQMLTPCFSQLLPALA
MDGLMVTFVGLTQLQFKLLAWEIKNIFHLEENDTEKNYTIKKRIKAVVDHHNFLISFTKAINSTFSGFMICYVVIIIASICVEMYNL
STGNSVGIFVKSMAYTSGILFEFILLFCIPAQNLTDEADRISYNSYFSRWYEHPEHAMAIQMITLTGQRQVNITAGGIIAINLETGLAT
VKTMISYCMFLRTMSL
>BlonOR43

MSLIYPKHFERGIIDDPLAKMKFCLNIHGFQTRWKTYFWHTAAILKMLILIGRTVYAFQTLDQPKKLAELVATYPVRFLAVCKMIV
LYLHRDLVTYFYQSVKVDFWNFHIAGPRVEKRILTRFLYVNIIIMSHLIASLTCLLLLILFPLSNMPDGVRPLPNIIWTPFDTNPSPLH
ELLYVIMIWNLGLSVFGNAFYDVLYVYSVQHLYVQFILLKELIINLSKGIVDDYSDLEKFQSNNFQRTVTDRLKICLEHHSKLLRF
GKNLEVFSSQVLVPQLIMSYAVLVINGYILSSSRMEMAKTIMLSNLTGSCIIQLAVFTIQASDLNEQSISITEAINKAQWYLFKAPLK
KYLCCLMVNSKKGISIKAGGMVKVDNEVLVTVTRKALSAITLLRALISEEDPSNK
>BlonOR44
MGPKHADKFLNVMKYFMMLSGQWDFRNEKSKFVPVYEKLKYVFFIWYALGPFVFLVTVIAGWKCREIVEENLFNFMYTSVVV
IVVCMLNSNDTKNIVNYVFHFENNQLNSSSKECQDLYAKAAKKNNVMVIGFYAVGLIACIVWFTAGKSIIVGTTDSCDFANGLSL
HLWYPFDIKKHMWVSNFFDSTSLFLATIYFVYSKTMPISLLLVVVTHLKIFQLDLKCLDAKAIAEAAKENLPVQQAKTILIKQHIR
KHQEIISLMNDVQNATRTLAFFLYFANVVNLATFIFDMLFGDSILFFKLVAAFILTLGQVYIFCDAGNDLMYHGSKISDIIYNDIDW
VSCNKENRLLLCLLIQRSQRGLALKIDIIGEMSLETFKTIMRLCYSTTTFCASVYQ
>BlonOR46
MYTSALMGTWPFIFRDNPGWRKCYDVYSKAVFTFFFLFIVTSYMKFVELLLAEELNFQELFANLVITLLYSVTFARVWALKSQRV
KTLVKEILETENKVLVSQDPHLTKIYQSFANQSQITNMLFVVNIFIVTTLYFVHPLFVDDVILFDEKSNTTKIIKALPLSSWFPFDPQ
DYYTQTYCLHLLDGTVGALFVMCSDIFAFSLIIYPLGQINILSYILDNFQHYAVKVKDQLNCSYEIASYATMRECIMLHNIII
>BlonOR47
MYIFAIKTACKLLNVSGINPANDYTLIQYALYLNNILGSIFAMLLIVLQFVLETNNFSVPEIADIIIFFSIYLQGVIKSVTLFHKKQSVM
KIIQRQMTWESTHMKDERLHKHYRVLKMSCIFVSFLHMSNGTLFFLTPVLTEKNLLVQCYRPKWLKYHYLLALQYLTSGVGILW
PIWMMDIIFLNIVRLTAIEFKRLNLQMSMLFNDMSSVEKKTRTEAALRASIQQHNHLLEFAHTVTRTFSQSLLWYIVINGSSICLEM
YVASTQTNQQYRVKALLCVITLLVQFMICYCYPCQDLTDQIEETSKNIYCSKWYNDLHQSRNILNIIRRSQRKFTVKAGNFIDVNL
EAGLTTIKTMVSYAMFLKSMDGNSANLPTR
>BlonOR48
MKIDNIEVCMRLWNVIGLHPKKGGSWFSLFRFCFVTVCLLFEISLATMNVYDKDTSLIEILRSFYTISFNFWGLIFTTTLFLKRSEIL
NLITLIRERFWNYEVMNLTEKNRKYCANTYKMMNNLILLVITANSIVILIFFFIPLCLVESPTTFPLHRLPWMSITQVKILEMIVASF
GEIIPHSMTMCFLMTIHYSTQLQFRMLSNEIEEMFNEEEGFSSKEVKKIIDYHNFLLGFVRDINRTFSGFNFAYFSLITPCLCLQLYT
MSVEPTVKICANSLLALTVVGSEFTILFLMPIQALSDEIESIGNTVYCSNWTSDRNVGSIIVMMINKSQKRVVITGAGLLEISLGISL
RVGQAIFSYLTFLRTISET
>TcasOR1
MMKFKVTGLVADLMPNIRLIQASGHFMLNYHADNSGALHTLRLGYCCMHLVFVLVQTFSCNFVNLVLERGDVNDLAANTITVL
FFTHCVTKFVYFAVRSKLFYRTLGIWNQPNSHPLFVESNNRYHGIALKKMRRLLYIIIIWTSFSAIAWTGITFVGDSVHNIKDPENE
NLTITEPIPRLLVKAWYPWDAMSGMPYYITLVFQVYYVFFSLAHANLLDSLFCSWLIFACEQLQHLKEIMKPLMELSATLDTYVP
KSADLFRAPSATSQDQLIENGTNPAKKNEDLKGVYSTRQELGGHFRGGALQNFGSGGVGPNGLTKKQELMVRSAIKYWVERHK
HVVRLVTAIGDAYGVALLLHMLTSTIMLTLLAYQATKITGVDKYAATVLGYLLFALAQVFHFCIFGNRLIEESSSVMEAAYSCHW
YDGSEEAKTFVQIVCQQCQKAMSISGAKFFTISLDLFASVLGAVVTYFMVLVQLK
>TcasOR3
MKLSSVTTCLFSSDFHTRMNFDWKDTIKLNFLMMKIVGLWPKEKYKINFYTLYTLISVNLFICGHVIFHTVAVFVVGRDLKHLIG
ALYMSLTETLLLVKICYFIKNSRLVKSLLTSLDGDIFQPKNEKQLELTNPSLIFWKKVHKSFAILVANTVFLFVSLPILSKSTKLYRLP
LEAWYPYNTQKSPNYEITYLYQFISTLFRGMASVSMDTFIAALNMYIGVQCDILCDNLRNLNETNFMENLSLCIKHHKAIVSFAR
ECNKFYNGIVLGQFFSTSIALGLAMFLLSLVTPLSTESNTLLFYLGATTSEIFLYCWFGNEVDVKSSKIPYSAFESDWTGAPIEAKK
NLLIFILRTQKPIKMSAINLFSLSLETFTTILRTSWSYFAVLRQVNGQA
>TcasOR7
MNKLQKFDWKATIRPNIAFLHYLGIWPEGEEYYKLNFYTLKTILYIIILVISTIVFQVINIFFTLDDLTSLTANIYVLLTEILYFIKLCFL
VKNMPALKLLMKTLDHKLFQPKANQIVIIQPLLNFWKLIFLAFVITCSFTVLFWAIFPILDSSEEEKRLPLLAWYPYDTKISPNYELT
YLHQVASYIYICYSHLNIDTFITALNTYIQCQFDILCDNLKNIKSDTKNVDTKLAKCIKHHLLILMFANTSNEFFSWIIFFQFTSSAAI

TGMTLFQLTVVKPFTTEFYNFMAYVTAEVVQIFMYCWFGNEVQVKSSNIPYAAFGSDWTEFSPNKQKSLLFLITRSQKSVKMSA
FNVFDLTTDSFILKSAWSYFALLNQVNS
>TcasOR17
MDDFNWISTVKTNLLLLHIGGIWPRGDGTHKLNLYTIYAIFITFTFTTYHCFSQIINFFFVDDLQALTESIFISLIQSMALVKAFYILK
NMRILKNILKNLETNKMLQPRNLKQIKMVQPSLTQWRLLSQMFWISAVFAMCLFGAFPIVESTYKEFRLPYLAWYPFDTKSSPFY
EIMYLHQFVSSYTIAIVDIGADTLIAALNVFVATQCEILCDNIRNINGSVEEMDSKWKECFTHHKEILKVARHCQKFFNWIVLMQF
CASVICIGLTMFQLTLVVSFSSEFFSSLFYFGAITVQIFMYCWFGNEVELKSSKILYATFEANWVEAPHQVKKNILIFAIRCQNPIKM
SSLNVFYLTLETFMAIFRTSWSYFAVLRQIQNRISEE
>TcasOR20
MNSFNWQESIKTNLKALRLVGLWPKSDFYKFDLYTFCTSLTVGVIVCGHNLSQIVYILQVYSDLKALTATIFVASINFLGAVKMYF
FIKHIKTVKILFKMLKTYQFKPKNIHQTQLIKPFLNLWKILYVGYSINVYLIVAMWSLLPVLNGWTWQKKLPFPARYPFDVTKSPY
YELAYVYQFICIWYITVANLNLDTINIALMMYTSCQCDLLCDDLKNLTETRFFHKKLIECIKHHKAILVFAEKSNGLFNMIVLSQIA
TSTVVLALTMFQLSMVSPLSSEGLNHLFYIGGIIMQILLYCWFGNEVEAKSSNILYAIYESTWFEASKNSKKNLLIFSIRCQRPIKAT
AVKLFALSLRTFITIVRSGWSYFAVLYNVGSK
>TcasOR24
MEEDFDFLSSLQTTLFCLRCVGTWPSNTYKLDAYTLYATASITICLFGHNFFQTVNIFFIFNDLNTLTGVIFVALTCLVAILKSLLFIFN
MRRLKKLLLVDIRQKLFKPRNRQQVVMVQSRVNFWKKIYFMFTGMGVATMFFWALFPIMDGTVKEHRLPFLAWYPFSVNKSPF
YEITYIYQIVSVFFIVIVNMNSDMLLVALMNILGVQCDLLCDNLKNIQFRERINEEFLRCVNHHMQILSYASDCNKFFNTIVLAQFF
TTVVSLGLTMYQLTIVTPFTSEFYSFIVYGGAVLMEIFLYCWFGNEVEFKSLNIPFASFGFDWTIGSVGLQKNLIIFIAKSQRPIRMS
ALNLFHLSLETFVKILRTAYSYFALLNNVNSLN
>TcasOR46
MSKSEKIHTLATYFDSNIAFLKLTAFWIYDDETTRRKKYLQHAYNIFWIFYLFVAYQPAELLYVYYSFNDLSVFLRALRDIGNHVS
LAYKAFNYFIMRRDILKLMETLQHGNYHYEDCGDFQPKLIVDEEKKEALKWTKYFLNFCNAICLSMFANGVFTFIFLSDKQYVE
RNGQRVYHQEQPVNTVSPFGSGTKLRFFVTFIYTMIALTFYAWTIVALDSLFITIMSCISSHLKILQGAFKTVRARFIKLCASLSKLLI
SVSGKLESIYSTQTFVQTFISLGEMCFSLYLLSETADQNIGNEITYLIATGFELLMYCWFGNRITEASLKISYALYESDWFPTSLSFK
KQIIFTMTRMQKPINVTIGKITPLAFSTFLTIARGAYSFFTFLKQRHGINH
>TcasOR58
MPFTIKDYDLRNAFETERTLLTLSGFYPRRTKKYNFFYNTSALINLFIAYGQLFSMVVQMVIDRNELSKLSETLLFFMTHFTFLCKL
TNFVYYKKKMFEIEDNLSRKIFYGFELWQIKPKIDSCKFIAKIFRILCILVVLFYTLVPYLDDKEDLSLPLPGWLPYNTKKYYYPTV
IFQVMSVSVSAYNNSSIDVLTCMLITVASAEFNLLKGALKTIDFHPKGHNTKQLIEAKFENCVNHHKEIVKFAYQIETIFSKGIFLQF
FASIIVICFTGFQMIVVPIPSMQFIFLIIYFSCMMCQVAMYCWYGHDIITTSDSIGQAFYMSNWYESDVKIRKNICIFLERTKKPVILT
AGKFVTLSLTTFTTILRSSYSYFAVLQHLYKEDS
>TcasOR59
MDEEFLIGTFETEKKFLRYGSFYPCGKRIKFIFLGLFMFVYSWTEFLSMITVLFVERDNLTKLSETLLFCMTQAAFLFKLVNFLYH
NKTMLRIESILKNPILNCLDQFEKNIIEKYMIRVKYLARLFRILCILTVSFYGLFPFIDEDPDHMLPLPGWFPFDVKTHQIELVIAQTC
GIAIGAFLNSTLDILPTILITLGSAQFDILKIRLENITSVDTSKSWLVKKAIKKCVIYHTILLNYITQIEILFHKGIFVQFTASVVVICLT
GFQMLVISVRSIQFILLMIYFSTMTCQIALYCWYGNELMYRSMGLSDACYMSEWNKCDTSVCKSLAIIMERGKRPVVLKAGNIFS
LKLTTLMTVLKSSYSYFAVLQRLYATSE
>TcasOR60
MSEDYTFRNVFAREKKILTISGFYPLREYEKNYFHFFSGTIQWIISLGMLFSMIIQSVIKRNDLMVLSETLYFLTTHLTFVCKLANLE
YHKKLLLDIEDMLKTTRFQKTLSLDLIEKTGMNEKIRKFNLVAKTFRIVCVWCVVLYVLVPYFDPGKSKTLPTPGWFPFNWTDK
YYYGTYFFEVAGISITAHMDSSIDILSWLLVTIASFQCDILKENLKNIYYNYDKEHDIRETFKDCIRHHEEIIKFTTKVEQSFSQGILL
QFLCSALVICFTGFLMLVVPVLTFQFANTIMYFCCMMIQLGMYCWYGHEIMTTSDEIGQYFYLANWYDSSLTLRKDFAIFLERAK
RPITLTAGGFVVLSLNTFTRILRSSYSYFAVLKHLYNKS

>TcasOR61
MGDYDFRAAFAFEKAIFSLSGYYQRQAGFSSLIICAIASLITIAQFLSMVMQIIVAGNDLTVLSETLLFFMTHFTYMCKLVNLLFYK
SKLLHIEDLLSRPRFYGFSQNELTIIKDGIEATNTVANLFRIFCVLACIAYGLVPYLDHTKAMALPLPGWLPYDTTKYYYPTYFFQM
VAVSITASVNSTIDILTWKLITIASVQFDILKRKLKDLDYKLETTSLQIQFKTCVKHHKEIVNYVKNVEKTFSKGIFIQFFASVIVICF
AGFLIIITPVLSMQFLYLTLYFMCMISQVAIYCWYGHYVMTTSDEIGQDFYMSNWYESDVAFRKDIIIFMERVKKPVTFTAGNFITL
SLVTLTRILRSSYSYVAVLQHLYNEV
>TcasOR63
MGFMIQDYDLRNAFSLERKLMLVVGFYPKRDNKHEILYWLSAFFNLLISYGQLTTMIIQMVFDRSDLSKLTESLLYFFTHFTFLCK
LLNFQYYSKDLIEIENFLTDPIFYGYSFEQLDIIKAKIRSCAFISNAFRICCTFTCSFYCLVPFIDESRKKILPLPGWFPYDTTNYYYST
FFVQSLSLFISAYCNTAIDILTWKLITLASAQFEILKENLTKIDYEGGFNETKGALVRCITHHAKIVNYTERVEAIFSKGIFLQLFGSVI
VICTTGFQLIVVPIPSVQFAVLGTYLCGMTTQVATYCYYGHEVMTTSDAIGMSLYLSNWYASHVKIRKIVMIFLEKTKKPTIVKAG
NFITLSLATLTQILRSAYSYFAVLQRLYKDS
>TcasOR64
MMSDEYVKDVFIANRWMLRCAGLWTPSTRSKLVQIPYKIYAIVVFLFVNVYFTSTEFLSLFYTHKNLYNFIKNVNFFLTHFMGAV
KVIFWFFKGHVLRDLMRTLESPEFHYEPCEGFQPGLIWRKYRRIGFKYSLGFLALAHMTLSSSYIPPLLTKLPYFSWMPFSYSTPR
SYLLALGYQAGPMFSYAYSIVGMDTLFMNIMNFIAAHLVILQGAFASSKMRVLDPGQMNNEMKRNCRHLQTILRVSEDLERVHR
YLTLGQLTATLFILCTSLYLISTTPASSKQFYAELVYMVAMGFQLYLYCWFGNEVTLMASEIPVNVWKADWYDCDQSFKKSMIFT
MTRMQKPIYMTVGKFAPLTLQTFVVYILRTSYSIFAVIKNTSI
>TcasOR65
MTATKSLKEIPPIYLRVHLTVLQILGIDILPVESVPQNLFYTYTALIISTMCLFTIAEFLDMVLNYEDIYRLTFGLCYCVTHVLGTVK
MFLMLYLRKKLWGNLTTLEEGIFKPNPTRGGPEELQIVNDAITMCNRQGYVFYTLVFLIIGARLLYASLANWPYDKHNYFDGNV
TVIVNTKEMPYTTWMPFDYNDSPLYETIFAFQIFSTTVYGFYIGAADAVICGFMMLIKAQFLIVKRELETLIERAQKAAIAENPDNE
DNFGREIERIELLDKRTQDYVAKYANECVYHHQELIALCDHAEEDFCYLMLLQFISSLLIVCFQLFQVSTLSPDSVEFFSMVCYLL
LMLFQLLCYCWHGNEVQIVSGELSRYAFGINWIIMRESPKKTLLLLMMRAQRPCYFTAGKFSLLSLQTFMTIVRGAGSYFMFLR
QMNI
>TcasOR66
MSKNLKEIPPVYLKVHLTVLQILGIDILPNERIPQTLFYTYSVLLIATMVVFTTAECLDLVLNYEDIYKLTFGLCCCVTHVLGAAKM
FLMLYLRKKLWGYFTTLENGIFKPNPCRGGAEEFEIVTSAINMCKRQGYVFYVLTVGVTGGQGLYAALANLPYDKHNYFDGNV
TVVVNTKQMPYATWTPFDYNDSPLYEIMFAFQIFSTTLYGFYIGAADAVICGFLMLIKAQFLIVKRELETLVERAQRAGNPDRGDF
GGGINRIEMLDDGTQVFVEKCANECVYHHQELIALCEHAEEDFCYLMLLQFISSLLIVCFQLFQLSTLSPGTFEFFSMACFLLFILF
QLLCYCWHGNEVQFVSGELSRYAFSINWIIMRESPKKTLLLLMMRAQRPCYFTAGKFSLLSLQTFMTVVRGAGSYFMFLKQMN
T
>TcasOR67
MDFTIRDFDLRNSFSLERKLLLVLGFYPIRDKEKHRILHQLSAFLNLLLYYGQLLTIIIQMVIDRNDLSKLTDSTLYFLTLFTFLCKLF
NFQYYGKDLIEVEKSLTDPIFYGYSFHKLQIIKAKVRSCTLVCLAFRISCTCSCFIYSVVPFIDRSGQKTLSIPGWFPYDTAKHFYITF
FLQSLSLFISAHCNSATDTLPCKLISLATAQFELLKDNLRTIDYENSFEETKHALVKCITHHRKIVNYTKRVETIFSKGIFLQLFASVL
VICTTGFQLVIVPFGSLKFAIHGIYLCAMTAQIAIYCYYGHDVMITSDEIGTSLYMSNWYASHIKIRKIMVIFLEKTKKPTIVLAGNF
ITLSLVTLTQILRSAYSYFAVLRRLYADD
>TcasOR72
MAKLEYLTGATFTLKCAVLYPIDSNNPKIKKILYAVWAIFFILTFVTGFIQCFVFVCINPFDLVQEAMIIMSLVFYSTTFFYFIVFYKN
WQNMVALVTNINKNFHRATDNVIEKISMDQASELSDKLAYVWTSSLAVGSVVPVVLAIATGNLEMPMPAWFPYDYNKSPVFEIT
YLWQVFCLITLAIIYGASDMFFPCITIIIGQQFKILASNFKNNFYTSLIKLGAEESIVQNFSKDIKTHEFRSFYIKYGNIFKILNNAKFQ
TLNRAFLKRNIKHHKLLLRFCEDLNKILNTFLLIRVSAIVFNLIFIGFNIIINADFLWTLYECPWYLCDVTYQKMLILVQMRVKRMV
STKAGNFFTMIAPSFIAFQRAVFSYITLLKEVTDLGKD

>TcasOR73FIX

MTRKHIFLNFTVTILKLSFLWPSNDNYDQWRLVKDASLIVSLMPCALPILAHFVLQITGDVYNMVTITENLIALICIIGMIYMTICFVKNRKLVKTLVKNLPAFTKYSKTTDIILTDKKANLYTKIFVFYGVIGNVVYMIMPYLNIEKCQQRQNNDVPCGLVTRCWFPFKFDYSPVFEIVFVHQFYTCLMVSVIILDLTMLICGFLMHITNQLKHLRGFIKRFDCSSQKIAEDVIYCVKFHTAIITYSEKTNEAFGTMMMLHITLTSLVISALGFEILIVDNFNDSLRFTLHLLGWLVLLLLICYYGQLLIDESIAVAEDIYYVPWHLAPVDVQKDIYMILMRSQKPLTLNAANIGVMSFPTFLRVISSAYSYFTLLLNIKS

>TcasOR76

MMESTVTRLKRMYLWPTASVTSRKPAFFLITFSCFLLYGSVMHLIVNDISMEEVHVIETTAGQFGVLYYLTLFTIYRKGILEIYADLSNFTKFGKPYNFDKRNKQLNQWSRWFSVVLYFFVISVFAWPGIFTQSCEDLNVALNKTEVCGVVSPVWLPFRFDYKPMKQFVYFWQSFCCLYSNGGAGTISFAMSETIEHLILRVEDLKILFPKIVAERSPEVRRKMLAKWVDYHLWLLSIGKLMNDTYRYSFSVIVLCAGTLFGCIGYTVMKNASTNFNSSFIFFGWMESVFVICVCGQRLMDAFHSVGTTVYNSEWCDTDVDFQKGVILITIRAQKPVRIYAGPFSYVSHLLILTVFQTSYSYINLLNASS

>TcasOR77

MKYILMKKTIAFLSVTGFWPKTKESTKTRAFCILFSSSFLLFGSLGYLIVYRKFGSDDIDSIETATSHFGVLYFMFFWILKRDGLVHIVNLLSDFSKFGEPRFFNDRNRQLDYLLQYCIFVLSVATGGVFLCPIIFVKNCEMVKQEKNLTKVCGLVSNVWAPFDYSEYPMKRVVSLWESYCCFINFGCGGIMSFTMIKTMEHLHIRVEQLKDMFPDVVNEKNLAVRKQKLEKWVKYHLHLYDIGELMNNTYRYCLSVIVLCVGILFGCIGISTMQPGSSHNSLFLFMGWFQSICILCMVGQRLLDVFLSVGVMAYDSAWYEKDVDFQKAVLMIMIRARRPVLIYAGPFTNLSHLLILGVLQTSYSYINLLNAK

>TcasOR78

MGHAIMTEILTYLTLMGFWPRSPKSSKASAFLIILSTSFLFFGILFYLIVNRQFGSSEIDSIETITSQFGVLYYLILFTWKRNDIVEIVELLSDFSKFGKPPFFDQRSTRLNYRLSCIVLILIVANIVVAALPVIYIDSCHKANEQLNLTKTCGLIAPVWLPFDYNEYPRKHLVFAWEVYCCVMNYVGSGIGALTMVGTMEHVIIRIEQLKYIFPKILDQPNPRIREQMLKNWVRYHLALFEIGRLMNDAYKWSLSVIVLCVGALFACIGISMLQSTASQINSICLFFGWFPSIAFLCMWGQRLLDSSLSVGTAVYSSRWYDMDVAFQKSVLMILIRSQKPIRISVGPFTHLSMLLLLGVFQSAYSYINLLNATS

>TcasOR79

MGHVIMNEILTYVTLLGLWPRSRKSTKTISYLIILSSSFLFFGSLLYLVVHRKFGSNEIDSIETVTSQFAVLYYMTFFTLKREGTVRIIDQMSDFSKFGKPPLFDQHNKRLNYLLSYFVICLFVAIVGVVALPAIYTGSCHKANEQLNLTKTCGLVAPVWLPFDYNGYPLKFLVFAWEGYCCIITYACSGISSLVLVGTMEHLIIRIEQLKLMFPEILNEANRHIREQKLKNWVQYHLALFGIGKLMTATYTYCLSVIVLCVGILFGCIGVSTMQSASSNNSVFLFLGWFQSLIVLSVCGQRLIDTCLSVGIAVYNSRWYDMDVSFQKSVHMILIRSQKPILIYTGPFSYLSHLLILSVLQTAYSYINLLSARG

>TcasOR80

MGHVIMNEILTYLTFLGLWPRSRKSTKTVAYLIISSTSFLFFGSLFYLIAHRKFGSNEIDSIETVTSQFGILYYWVLFTLKREGTVEIVERLSDFSKFGKPRFFDQRNRRLNYLLSYFVLVLMVAIGGVVALPVVYIDSCHKANERLNLTKTCGLIAPVWLPFDYNEYPRKNFVFAWEVYCCIMTYACCGIAALVLVGTMEHLIIRFEQLKLMFPEILDEPDRHTRQQKLKNWIEYHLTLFDIGKLMTSNYTYCLSVIVLCVGILFGCIGVSTMQSASSHNSVFLFFGWFQSIGVLCIWGQRLLDTCLSVGIAVYSSRWYDMDVSFQKSVLMILIRSQKPILIYAGPFSYLSHLLILSVFQTAYSYINLLGAKG

>TcasOR84

MTEEKELRLCLWSCYYLKLSLMWPLKREEFKSSKGLYLRLLVFVIISGSTFTAMIFMHLYKSLKVGSYDVSEDLAILASNIGYVLMMTMYVSRQKDLELLLLDLSDFKTYGKPPNFDKVRKRMDLYAHLIFFYSMFGSFVYNMDKIILIDKCKEARRINEVCGSAIPFWTPFETEDLFTLTLVITYVLINIFVVVKVAMTVSVQVLEISSHINLRIEQLKIFIAGCFDRDFKASRERLDFCIRYHNVIIDFSERFSRCFSYVMFIHLAITGIIIGCLENQIVQEHQPEAMLHMGGWSTATFIACYGGQLLMDASTSIADEFYNCPWYEADVKMRKDLILIILRAQKALFVSTGPFNVLSFALFVSIMKLSYSIFTVLS

>TcasOR86

MALNQEDAICSKSCFYLRYSFLWPEEAPTRSFYAKFILVLILSFLTAFLPLFIHFLILVERGLDPSEDLFVIISYTGFALIMIIYVIHVKK

TSYLIVQLSDFEKFGKPRGFDYWDKKFRLISSGVYYYVLIASSGLNLGRWVGMAECRKERDFQVCGIVIPYWLPWKVDSWLFFI
LLDLYVLKMTLVVNCALFLIIIQILEITTHLKLRIDHLKEMLVKCFDSDSQTNRKQLVNCIRYHTYIINCSKLFKKCFTHAMFSLIVT
MALSCGCLESQVVKFDLWALPPISAWIFILFIACMAGQILMNASLSIGDAGYHSKWYQTDANFRKYLILVLMRSHKALVLSAGPF
NILCFELFVAIMKFSYSVFMLLNQN
>TcasOR87
MKHVIMDELLIFLTFLGLWPRTPTSPKIISYLMIYSTSFLFFGSSIYLILHRKFGSDEIDTIEIITSQFGVLYYLTLLVVKRDGITKIVNL
LSDFSKFGKPPLFDQRSRRLNLLLRLFVTVLLAATVAIVSVPVVFINSCNKQNLQLNATKICGLAAPVWLPFDYTQNPRKYFVSA
MEIYCATMNYAGSGSGAFLVIGTMEHLVIRIEHLKNMFPEILNEPDKQIREKRLKKWIEYHLSIFEIGELMNETYKWPLSVIVLCV
GILFGCIGVSTMQSVSFQNSSVFLFFGWFQSIFVLCFWGQRLLDSCLSIRKAVYNSKWHEMDVSFQKSVLMILIRSERPVLIHAGP
FSYLSNLLVLGVLQTAYSYINLLNARS
>TcasOR88
MTEEKQLRICLSSCFFLKWSFMWPTKSEEFRTSKGLYFRLLAFVIISGLTFTAMIVMHLLKSVEAGDYDISEDIAILATNTGYILMM
LLYIIRQKDLESLLVDLSSFKKYQKPPKFDEVNRKLEWCTRMVFGYCVFGSVFYNLVKILAIPSCKKSRRINEVCGVAIPYWVWF
DTENWSIKLPLILHTFLVIIIVDKVTLLVSLQVLEIACNIKLRLDQLNCMLVSCFDGDVEASRRRLNECIKYHKEIISYSEIFSKCFSIE
MFTHLTTTGIICGCLENQVVQEHRPEAILHIGGWITAIFVSSFGGQILIDSSLSVAEAAYSSAWYEADVSLRKDLILVILRAQKALFV
STGPFNVLSFALFVSIMKMSYSILTILQ
>TcasOR89
MKEAVLQQSKKEMHLLNLWPKGHVKHFRFRYVITLIIVSPFTLGTLTHFINVLKENLDVDLSGDISVIAVVTGLHFMLITFVWGH
KKIAYLWENLGPHEYFGKPDNFEKRCKQLNFYSRLYAYYCYLGLTVYIIMKNRGGIECRRLNVERNLTEICGLVTTFWAPFDIDFF
PFRQILFVDQVFATYFIVKGGAAISFTTLEVGEYIILKIKHLKRLVKEVFDDPREEVQRKKLVFCIKYHQYIISIQELYDGRYKHCNG
CYILMVGIIIASLSNEIMKNHNIEALLHLVGWVFSFYICCFSGQSLLSESLTIPDAAFESKWYEAPVYMQKDLLLMMLRSQKPLML
HATPIGVMSLSLFITLVKTSYSYFTLLNQST
>TcasOR90
MAKDTSPVLRESIEVMKYLQLWPQNERTNLRRRYFIVIFLCSPLHLGLATHLVVCLKDNLDVDLSANIAVLSAVTGLTYMLIVFV
WSQDKLVHLLAKLDTHEIFGTPDNLTKRSRRLNFYAKLYSYYCYFGIVIYSLVQIIEMPQCRKMNEEKGLSEICGMIVPFWAPFDI
DWFPLKQIFWLNQLLGIYIIIKGGAAVSITTFEVAQYICLKIKHLNRLLREAFDDPCDVVVEQKLLHCIRYQQHIIRTNELFNVCFK
HCNGCYVVMVGIIIASLLNQILKEKSVGALVHFAGWICSFFICCHAGQAVISESLTIPEAALDSHWYEAPVKYKKVLLLLLVRSQK
AFNLQATPIGIMSFDLFIALLKTSYSYFTLLHKST
>TcasOR92
MKNQEIKICRATLTVLKYSLIWPSEADEMNPGKWYYIRVVTFILFTCPWVLSVFMHLIVSIRNNADIHLSEDVALMVAFTGVYYM
TIIYVKKQPKVAFLLRDLSYFQFGKPPGFDETERILGFLSKLTFCYSVMAVVIYNYIKYRQKPECERMNKLKGLKENCGMLTPTW
WPFEINYSPAFQLIFLYIFTSTQVMMKLSLMISFNVLEMAHHIILRINHLKTMILESLDEQDYEASKRKIKTCILYHLEILGFAERMD
DCFSNGMFAHLTITAAICGCLEKQFVDGDNQLGSLLHIFGWILALFLACLGGQHLINASETISDAIWSSKWYDADLRLRKDLIFM
MARSQVGLYLNVGGFGILSYALFLSVIKMSYSILAMLTS
>TcasOR93
MTNLEIKICRATLKILKYSLIWPNEADEMNPGKWYYIRVATFLLITSLWVLSVFMHIVMSIIHDADVHLSEEVAFCVAFCGLYYMT
MIYVKNQPKVALLLRDLSKFQFGKPPGFEEKERILGFLSQFFFYYCVMAVMVYNLVKLLQKPDCEKMNEIKGLKENCGLLTPTW
LPFDINYFPAFHLTFLYVFISTQILMKLALIISFNALEMAYHVILRIDHLKIMITECLDQRNYEVSRRKLKTCILYHLEILSLSNRLND
CFSNIMFAHLTITAAICGCLEKQFVDGDNRLGALLHVCGWISALFVACIGGQHLLNASLSIPDAIWSSKWYEADVRIRKDLLFMM
AKSQVGLHLNVGSFGVLSFSVFFSVLKMSYSILAMLTS
>TcasOR94
MAIKICKFTRKNMQISLIWPREFEEINPGKWYYIRIVIFLITYGVFPFCTFLHAVVVIHNNLDIRISEDIGAVVSNIGISYMAIIYVQQQ
NQIAYLLKDLSDFKDFGKPPFFEEENKRLNFWSICTFIYPTCGASLYNLSKILEKSECNKINEENGLPATCGFIFPIWVPFNINYFPLF
HIMLISTWFCTTMFVRLHLSISYNAFEIAHHIILRIKHLNGMIITCFDCQDYKISRQKFTTCVLYYKQILDLSNRLNQSFSSIMFVHF

TMTSAVCGCLEKQFVDGEYVGGFIHLVGWIISLFIASVGGQDLVNASQSISEAIWSSKWYLADIRLKKDVLFMLMRSQKDLHMSVGSFGVLSYAFFVSVLKMSYSILAMLTS

>TcasOR95FIX

MVVKESEIKVSRVTRKILQYSLIWPKEGDEINPGKWYYIRIFTFLSFTSLWCIAICMHFIIVLKDKIDWDVTEEIAIIIAIYGTYYMVLAYVKNQKKAARILRDLSNFERFGVPPGFEEEEKRLKVYIIGIFIYAFLTITFYNFFKLSQKGACERFNEEHHLDENCGLLSPVWIPFKVDRFPQFELVFLYLFTCCHLLMKLPLVVSYNALEMVHHIILRINHLKIMITECFDEPEYEISRRKLTQCILYHIEILEFATRVDDCFSNCMFAHLTLTGAICACLEKQIVAGISRFGAILHFIGWILALFIGCLGGQHFINASDTIPESIWASKWYNANLRLRKDLLLMMMRSQRDLHITAGPFGVVSYALFLSVLKMSYSILCVLTS

>TcasOR97

MNNQKIQISNMTRKVLRYSLLWPKTNEELNPGIEYQFSVLGFFLVTGVLVLCITIRFFITIKAVHEVDAEVLAILIASYGSYYMICAHLKNQHKVALLMRDLSVFNNFGKPPNFDKRNNQLNFVAKLLALYSFLATIFYNGEQLINKTECKRINKEKGLSDHYCGLLAPCWLPFEIDYFPVFHLILIYAFTSGYLLIKMAIHISYNAFEIVSNIVLRIEHLKAMILETFENRNKQVCHKKFLQCILYHIEILDFAARLDDSFFNSMFGHLALTGGICACLEKQIVSGVNVVAGTLHFIGWILALFIGCVAGQYLINASEILPSAIWTAKWYDADLELKKKVLFMLARSQKSLFIRAGPFGILCYPLFVTVLKTSYSILCMLTS

>TcasOR98FIX

MVKKESEIKISRVTRKLLQYSLLWPTEGEELNPGKWFYFRIFAFLSFTSLWCIAICMHFIFVMKDKPDWDPTEEIAIIIAIYGTYYIVLAYVKNQRKAAGILRDLSNFDKFGVPPGFEEEEQRLRVYIICVFIYGFITITFYNFYKMSQKKSCERFNIEHNLHENCGLLSPVWIPFRIDKFPRYELVFLYLLTCCHLLMKLPLIVSYNALEMVHHIILRINHLKIMITECFDDPDYEISRRKLTQCILYHTEILEFATRVDDCFSNCMFAHLTLTGTICACLEKQIVAGFSRFGAILHFFGWILALFIACLGGQQFINASDTIPEALWASKWYNADLRLRGDLLLMMMRSQRDLHITAGPFGVVSYALFVSVLKASYSILCVLTS

>TcasOR100

MSPKDKIKICGITRKVLRYSLLWPVENDELSPGIRYKLTILAFFSITGILVFSISVYSVLEIKQGYDIDVEDVAILIAVYGTYYMVSAYLNNQHQIALLERDLSQFYKFGKPPGFEQLNSQLNFAVKVLIIYSFLGTFVYNGTKMLLREECKKNSQEKGLSDNHCGLIATFMFPFRVDYFPVFYIVLVITFLLAHTLIKLCMHISFNAYEIVNHIVLRIEHLKEMILSCFNERNQTIVQKKLRVCILYHIEILDMAARLDKNFFNTMFGHFALTGAICACLEKQIVLGVNIVAGTLHFIGWIIALFVGCVAGQCLLNASEIIPNALWAAKWYHADLRTQKTLLFMLARSQKELTIKAGPFGILCFPLFVSVLKTSYSILCMLTS

>TcasOR102

MQNQSKPCQLDMMDETYLQFFVKSFTYLNMLPEKTTFCTTIQQYYVSVIITITTFPILADLVSQFYEESISFTSVNENFVALSALFAVIYVSVCFINRKHKIRALIADLALFETFSSKAVITETDKSVKFYTKLFIVYGIVGNLCYGLLPILGYKKCHESKSVHMTRYGIPCGLVVRFLFPFKFDYSPLAELVALYEILVCILGTSVVIVVTTLICGVLIHITVQLQCLRKIILDLSQVNDLEILEHKMKFCVKYHTAILDYGIRTDLAFNQMMLLHITWTGFIISVLGFEISTTDDYVEAFRFFMHLLGWLGMLFVVCYYGQKILDESLAIADAVYTFLWYKKSVIVQRYVLLILLRSQKPLTLRACGVKVMSLATFLGVLYSAYSYFTLLLKLKP

>TcasOR103

MKQALKLADVLGFNPLKNDNLTKLKKYSSLICMISVVVSAILEFVSNFSALETYESAPESLVPQFQTLAKISSLLLSQKDITELIDEIKYFWKLDQFGDFHTRKLKKIYKYVTIFFYFYTLMLSGACVLFTITTVIFTPEKPLFLCYGGLHGLPSPQFEIYFVVDLAAIVIMSFGVAAYDGIFFYFAFHVYAEFKLVKVAFKGKSTFIEAVKHHDFLLKYLRKLNEIYSPIFLCQFFSNLLGICFCLFMLSRSGMPPELTSFSKYFISLVAFTVQTYIFCLIGDLVSELSLDISNVIFYVDWLDDEVYKSKTARLVIMNKAQSPVKLTIGKFTGMDLRTFLLIVRNAYSFLAFVNNALD

>TcasOR105

MKPALKLANVLGLDPLRNDNYTQLKKMFCALCIVSLFVSAYLEFFSNFTTFETYETAPESLIPHFQTMFKMYSLIFSRTEIVELIQMAEQFYKFSQCDERKKLTKLYKRVDLFFYVYASLVAAACVLFAIVTLIFKPGKPIFLCYGGLHGLESPEFEIYLVVDLIGIVIISVTVPAFDGLFFYFALYIYTEFKLLKIAFKTMSGQELREAVKHHDFLLKYIKKLNSVYSPIFLYQFFCNLLAICFCLFMLSRSGIPPEMVSFSKYFLCLLAFLVQSYTFCSIGDLITELSEDVSNAIFYTDWLDDEAYENKTARLIIMSRAQNPVMLTIGKFANMNLRTFILIVRNAYSFLAFVNHALN

>TcasOR106
MESALKLIDIIGLHPLKSDKYSTMRKTISFLSLVVILISAQLEFLSHLSVFEVYNSGPHSTIPPLQSLLKMATLHFYKNELIDLMEKS
KSFWKLDKFGDLYKQELSKLHRLVTIIVYIYIALLTATCVQLAVLTLIFRRGKPIFLCYGGLYGLESPHYEIYSILDAIGIGVISIAVSG
YDAMFFFFALDIYTEFKMIKSAFKRHSDQTVSSYNKQFIEAVKHHDFLLQYINQVNDIFSPMFLFQFFSGLLGICFSLFMISRSGLQ
DINTLSIYSAGLLGFTAQSYTFCLVGEVISELSEDISNEIFYTDWLDDEVYRNKTAILIVMNRAQESPKLTIGKFADMNLRTFIMIVR
NAYSFLAFINNALD
>TcasOR107
MENPLKLLHIIGLDPRQSDKYSTIKKVISFLIVLAVLLSALIEFFLHHNESQVYDTAPQSTVPNLQALLKMFALIIYKKELIDLFTKG
NHFWKLDKFGDCHKQKLTKLHKYVDLFFYVYAVIITGAFLQLALLILIFEPGKPIFLCYGGLYGLESPQFEFYAVLDFLAIGVIAISV
TAYDSIFFYFALYIYTEFKMIKIAFKRENCAQFIEAVKHHDFLLQYISKVNEVFSVIFLTQFFSGLLGICFNLFMISTQGTRDMKSFST
YFVGLVGYTAQSFTFCLIGELISELSEDISNEIFYTDWLDDEVYRNTTARLIVMNRAQESPKLTIGKFADMNLRTFIIILRNAYSFLA
FINEVLD
>TcasOR108
MGSILLLNSVLKKMEKALKLVNILGLDPRKNDTFSKFRSIFCFTILISASFSSHLEFFLNFKGLETCERAAESIIPQYQTMCKMATFL
LYKTEMLDLIKKSERFWKLDRFGDLQAKNLHSTYPIFQIFFYVYVVILFLTCAMFALVNWIFDTGKPISLCYGESEGLETPWVEFY
IVLQSVEVTIIFLGITGYDMVFLYYAGSVCIQFQMLKMAFAERKMNERQFLKAVKHHEFLLQYVEQLGDIYSMWFLLQYFSSLFG
ICFGLFLISKEGLPTEPERLSKYFPYIFSFTMQSFTFCMTGTMLSDWSSEISDEIFHSDWSDDQVYKNKTARLIVMNRAQRPAKISIG
KFLDLNLRSFILLMRSVFSFLAFVNNILNRIN
>TcasOR109
MGKVKFTEPLEFLNVVGLNPENCSNFSLFRRVISLGFFLVVITLGLLELLLHFEGLETCSRASEAMIVQYQLFIKIAVLLKHRKNLV
VLMQKTRKFWPLDKFGQDAKIERPHKLLKAFFFAYKLIMILMALQYILRKFVSKNGKPLAIAFGESKGLSPKVDHLYFVLHSTST
FVVLHAVTGFDRLFFFLIGHVLTELKLVKKSYRLTQNRREKFLETVQHHAFALEFVRKLNRIYSQVLLNQHLSCLFGICFGLFLVS
KDGIPPDLGHVTKYVPYVISFITQTFTFCFIGSLLITWSLQVPDAIFYNDWGKNQAYKYKTDKIIAMIRGQRAAKLTLGGFGDLDL
ESFNLVVKNAFSFFTFVNAMNQK
>TcasOR110
MDKVEFSDPLFFLNVIGMHPFKADKFSKFRLAFSIAVYFAVIFSGVLELIVNSQGLETYARASDTLIPQCQLVCKIFVLAKYKKQIA
RLLNGSQRFWDLGQFGARYGNSFGKTHKYLKSFFLLYKVMLTFTCLQFLAVKIIFKIPKPIAISFGETKGLEPLYDHLYLVLHAMIT
LVTINLVNGFDGLFFYFIGHVLTELKMVKVAFGDSPIETNWSEEKRFKFAVRHHRFVLDFIEQFNIVYCTMLLVQHLTCLFGICFGV
FLMTKDGVPPDLDRASKYLPYIVTFIFQTFTFCFAGNLLLSWSLEIPNEIFYHDWAKKTTYENKLAKIISMKRGQRAARLTLGGFA
NLDLDSFRMVLKNALSFFTFVNAMMNKKAVTSV
>TcasOR111
MEKVRLTEPLFLLHIVGMSPHDSGTFARIRKIFSILVYTSTVVLSMAELFFNYKDLETVIRATESFFTQYGLAWKIAVFVVYKTELA
QIIRLCDNLWPLDEFGTGHNFQFLHKFLRRFFLLYTGNLALLCTQFAVTAFFDDQFKSVMVYYGEKESRSQIYDNFVFTLQVIYLY
VGCFVVAGFDCFFFYLLGHAVTELKMLTISFSCKEIGRNWGYEERFKCSVKHHIHVLELLDKINKVYSVMLLNQHLCSLFGICFGI
FLMTKDGIPPNVDHFSKWSTYIFTFILQVWTYCFAGDQIMHWSLKIPDEIFYDNYWNKYSLKNGLNKIIAIQRGQKAAGVSLGGF
AMLDIESFNVVIKNAVNFFMFMDKMYKRE
>TcasOR112
MITRLMAQFAIKGRVGTGGYIMDKVKLAQPLAHLNIIGLDPLKNDRFSKIRTVITVAVFALCNVFSFSELFLHYNNPHVIVRSSEVV
FPFFQNDWKIAIMLVYKKNLAQLIQNTSRFWQIDAFGKNYQYSMGIKHKYVRIFYLVYRLMLMFSCSQYILLTIGSDRPMILSFGE
TGGLGSGALLFYLIFHIVYLLIIFNVINGFDGLFFFLVAHVLSELQMVKVAFSSSKVITFWNHKRRFKSAIQHHRFVLDYINRLNSIY
SILLLNQHISCLFGICFGLYLFISDGFPPDYEHISKYVPYVIYYITQVWVFCFAGQLIIDWSVNISDEIFYHDWTLNRTYENKTDKLIII
QRAQHAARLSLAGYGNLDLQSFNLVLKNGLSFFTFVNAVIHK
>TcasOR159
MRGKTIESTTNPYSSLKKVFIDFAYSKLVISYTKASLTFHVLSLLLEVYYLVTNFSVELICRYGCMMCLMTYMYSKKLKLLEKPCL

LDFWKVYNSSTATQRLISEKSSKTNRRLYCALTCCFFLAIILFPIWGDLNEFFIFSQVYEKYFTSWAPAFCYFYVSTLLWCCFYCFH
LPGIIMYLTLHLDLQFKLIKDKITEIDKNCSQKEIYQILRLCISHHVALKKWMDKLADLLVTIMPFFFLFGALNSIATSFFVLYTLQN
TTMILKIRLGTLTLCNFIIVSTFAEVGQIFSGQNNSLFEQLMDCSWYLWNIKNRKTLLMFMLNCMKPKTFSWGGITLNYSFVLFIL
KTSLSYASVLFKLRGETF
>TcasOR160
MSGKTKRITTKTIHLSNPYSSFKKVFSDFAYSKIMIFYTIATLAFHMLSLFLQIYYVATNYSVELICRYGPMMCLAIYVVTAKVVGV
FYYKTFTMLENQCLFVLWKTCNSSPTTQRLILNKSLKMNQKLHLALMSYFLLAIVMLPTWGDLNELFIFSQVYERYFKFWAPVL
YYFYISTFLWCSYYSFHLPGCILYLTLLLDVQIKLINDKITEIDQNFSQNEISETLRLCISHHIALKRWMSTLAKMVNSVMPVFVLL
GALSTVAVSFFVLNTLQNTTMILKIRLAILTVCNFVIVSTFAELGQIFSDQNNSLFEHLIDCPWYLWNVKNRKILLMFMANCMKPK
TFSWGGITLDYSFAISILKTSFSYALILFKLRGETIRN
>TcasOR164
MSGKTKRTTTTRKINLANPYSSLKKVFIDFAYSKIMMFYTKATLAFHVLSLLLELYYVATNFSVDLICRYGCMICLMTYVVTAKV
VGIMFSKPFKLLEKQCLFVFWKTYNSGPTTQRLILDDSLKMNRKLYLALMFYLLLAIVLLPVWGDLNEIFIFNQVYETYFKFWAP
VLYYFYISTFLWCCYYSFHLPGSIFYLTLHLDLQIRLINDKITEIDQNFCQNEISETLRMCISHHIALKSWMSKLAKLVDAVMPVFV
LLGALSTVAVSFFVLNTLENTSLILKIRLTTLTVCNVFIVSTFAELGQIFSNQNNTVFEHLMNCPWYLWNITNRKTLLMFMLNCMK
PKTFSWGGITLDYRFALTILKTSFSYALVLYQLRGETN
>TcasOR165
MSDNTKKATTKSLDLTNPYSSLKKVFINFAYSKIMIVYTSATLIFHILSLMLEIYYLATNFSVELICRYGCMMCLITYMVTAKFFGM
LFSNQFKFLEEQCLLDFWKAFNSGPTTQRLILKESSKMNRKIHLALTFYVILAIIMLPIWEDVNDFFMFSQVYENYFANWAPVLYY
FYISTFVWCSYYSFHFAGVIMYLTLLLDLQFRLINDKITEIDQNSTQNEICGTLRLCISHHIALKRWMNKLANSVDTAMPVFILLGA
LSTIAVSFFVLNTLQSTSVILKIRLATITVCNLIVVATFAELGQIFSDQNNSLLEHLMDSPWYLWDVENRKTLLMFMANCMKPKTF
SWGGITLDYSFALSIFKTSFSYALVLYQLRGNTF
>TcasOR167
MAKTGDIFPVRDPVKRCLFIPKLLLESTNFWPEKRNFLTKFANWVMLIICVLIESGQIAFVVVNIKDITKIASAMSTVSTTFQAITKL
TVLYIYNDKLRLILKSVWYEFWPSYTAGREINTKLETYNKIVIVSFLTILISGICFAFGFLSSPLISGERILPFETVYPFDWTKSPYYEII
YVTEWMTNIAFILIGICGHDFLFMGLCSNVVGQFTLLRELFGYLGTKNVAQIIKKLGHDTNIEPNRQLLRICIIHHVRVTEICKEIAE
IFSFSCFIQLLSSVTALCVGALIMTFADIDAALFTVSSAYIVGHLLQLFLYATLGNEVIYYASRLPNAIFHSHWYNIDLEVKKDILFVL
QRAQKEVKISAMGVSVLDYQTFIQVLRLSFSFYTMLSKVTDH
>TcasOR171
MVKLFLLLKHLTMKAQDSDNPYIVLRRVFVDFAFTSHMIIYTKITFVFHFLTLLLETYYMITNFNVELFSRYGCMMCLMTYSNVQ
IVLAKLLEILFARHIKFLEEERLSHFWKLEESSEETQKVVNAESSKIRKKTFFVLSWFVALGFVLFPIFGDLNDLFMFGRVYRNYFG
SWAIIPFCIYVSTFPSIAYNSICLPAVVSYFIFHLNLQISLINDKLGKISEKSRQSEIYQKLCSCVAHHVRLRRWTNIFQNELESALPFY
LFLGAINSIAVSFFILYNLQNMTLIFEIRLVVISVCNVLILWIFAEAGQEFSDNSDSIFDAVVACPWYSWNAQNRKIMLIFMLNCLKP
MTFSWGGVKLDYQFTVTIVKMSYSYALVLYNWRYEK
>TcasOR172
MSFQALKHLLKMCAEKTPDLDNPYLTLRRVFIDFPYSKSMKIHTCITLLFHFLSLILEIHYLVTNFSFELSSRYGCMMCLMTYVISV
KIFVIMFAKPLKILEEQRELHFWKIGDSSHAMQQSVATEALQVKKQTYFALSCFVLLAVILYPVWGHVNDLFMFSQVYEKYFGD
WSVIPYYFYVFTFMSSSFNSFQLPGVILYFTLHLNLQISLINEKITKISGENYCQDEVFKQLRDCISYHVALERWMARLIDLTKTAM
PVFILLGALSSIAVSFFVLYSLENTRFILKIRLTVVAICNVLIVATFAKAGQRFSDKTGLIFDAIATCPWYSWNVPNRKIVLIFMANCL
KPKTFSWAGITLNYQFAIKIVRTSCSYALVLYKLRNGNY
>TcasOR187
MSTKREVVKNFPYYYLFKICIDFGYSNVVKRLNICCITMIVMFHLTQIHYMQENFSKELILKYGSGIALGIYTILSMSVQMLIEHEI
KDLIAEALFSMWAVDSCGPQVEKLILRRAKVMNIIYCSIFAWFALMATVMLPMWGDHSEWLLYDPILVEDVKTRLKIIYYLSTFII
FPMIAFSAIRLPGILLYGILQIHMQIMLINHKLVQVSEDLDDLNNVKKIDQDDYQERIYKELCLCVEHHIKIKLWLNKLMKIVQLL

MPPYFLLGSINAIYLLFFVVYNDTSNILKVRLCILLIVGGQILCMFAEAGQALGEETGRIFDTLVNCPWYLWNKKNKQALTIFLSN
SFQPYTIAFAGFTLNYSLALALLRSSVSYALVLYNMRN
>TcasOR188
MFVKRQVLEGFPYYYLLQLCLDVGYSKMMKIANIFCIIINLLNVLAQIGYIKQNFGKELLLRYACGIQLTIYTIVTMLFEFLVEQNV
KKLMDEALSEMWPIDFCGLEIKKLILKRSTVMNSIFYFMFAWFAILAIVMLPMWGDQSEWLLYDRICKEFFATWWKIPYYFYFTT
FPVVAFSGIRLPGLLLYTILQTHMQIILINQKLVQISGGLDGINDVRMIDQKNYQKRIYKGLRLCVAHHVAIKRWLQKPVKIVQSL
MPIYIIMGSTIFISLLFATVYSFRDSSNILKVRMSVVLMICCLILCMGAEAGQALSNETSRVFDTLVNCPWHLWDQKNKKALTIFLP
NTLQPVTITLAGITLNYSFAVGLLKSSASYALVLYNMRN
>TcasOR189
MEKMFPQIRTEDMKKFPYYYLLKICIVFGYSKIVKLLNVVCIIITSSTIVLQVYYLKQNFSKELILKYGCGISLTIYTIASMLVEFLIE
QKTKKLLNEAGTILWPVNFCGVKVEKLILKRVTVMNIIYYFMSAWFALMGIIMLPIWGDHSEWLLCDVISNEYFETRWKILYFAC
SCFSFPVIAFSSIRLPVILLCTILQTHMQIILINQKLNQISEQMGNLNNIKLVDDKCYQKRIFEDLRLCVSHHGKIKKWLNKVLKLVQ
SIMPLYIILGCLNFISLLFFASDGLQNASNILKARLCVVLIVCCLVLSMFAEAGQALSDETSGVFDTLLTCPWYLWDKNNKKVLSIF
LSNSFQPDSISVAGITLNYDFAVALLKTSSSYALVLYNMKN
>TcasOR190
MSTKKQDLLKHFPYYYLWKVFINFGYSKLTKLVTISCIIIHSSSLFVEIYYIYCNYNKEIIFKYGCMMSLLGYITISMVVELLLEKDT
NNLVCEARSLFWTIDSCGVQAQQIIHKRAVVMNATFGFILMWVATLGVIMFPIWGDQSEWVLCVKIFENYFENWSQMANFVFFS
TFPMVAYSTIRLPAMLLYGILQTHMQIFLINQKITEISRSKDQEKIYKELCLCVSHHVEIKRWLQRFLKMVQLTMLMLIPLGVLSCV
CVLFFVIYSFLDTSNILKMRLTVVVACTVLIVYIFAEAGQDFSDEISCIFDTLVTCPWYFWDQKNKKALVLFLANSLKPYTLSIAKI
TLNYDFAVALVRTSVSYALVLYNMKN
>TcasOR191
MRLEIEALKNFPYYYLLKICIDFGYSKIVKCINVVCIIINSSTLFIQVYYVQQHFNKELIFKYGCGMALTIYTIASISVEFLIEKNAKN
LVNDATAFVWPVDFCGEKVKKLILKRATVMNKICYFMSAWFALMGIIMLPVWGDHSEWLLCDLLSKEYFETRWKILYFACSCFS
FPVVAFSSIRIPGILLCTILQTHMQIILINQKLNQISEQMGNLNNIKLVDDKCYQKRIFEDLRLCVSHHGKIKKWLNKFLKLVQSIMP
LYIILGCLNFISLLFFASDGLQNASNILKARLCVVLIVCCLVLSMFAEAGQALSDETSGVFDTLLTCPWYLWDKNNKKVLSIFLSNS
FQPDSISVAGITLNYDFAVALLKTSSSYALVLYNMKN
>TcasOR192
MVSEQTLLKNFPYYYLLRIFIDFGYLKITKVLSVACIIIHSLSTLLEIFYICQNFSKELVFQYGCITSLATYVITSMTTGFIIENDAKNLI
RETVTAFWPIDFCGPQVEQLIFKRVARINTFNFFLLAWFAIFGIIMFPVWGDESEWMLCVIAFKKYFPKWWRVPYYVFFATYPMVA
YSAIRIPAMLLYGILQINMQFFLISQKIIQISQKPQNKTHQPGFYQKTVYKKLCQCISQHAEIKRWLQRFLKMVKSVMPVFIFVGGL
CFMSILFFVVYTFQSTSNILKVRLGVILMICNLILVTFAQAGQTVIDESSGIFDTLMTCPWYLWDEKNKKTLVIFFSNSLKPITFSIAS
ITLNYAFAVALLKTSASYAIFLYNIKN
>TcasOR193
MSELEKQLPYYFLMQFCINFFYSKTVKVVTSSCIIIQSLSLLLQVYFIITNFSKELILKYGCEMSLATYLLTSLLVDVVVENTTKQLIS
EGHTSFWSIDSCGHDVKNHIIANSARLSVVIYFILAWFAVLGISVLPVWGDQSEWILFVQIFNTWKKILCYVYLSTLAVMVFLSIRL
PAMLLYGILQIHVQIILINQRIIQIGRENTNDIRMMNQMSYQNRIYKELGFCVSQHARIKRWLKKLLGIVQSAMPIFTVLGGLIFISV
LLFVLYSFENASCFLKIRLGMVVISCSLVLCMFAVAGQAFSDETSRVFDTLMTCPWYLWDQKNKTILLIFLSNSLQPINFSIANITLN
YSFAVALLKTSTSYALILYNMKN
>TcasOR194FIX
MAMKQYPFLYKIFLDFAYAKIGKMVTYSCIIIQSLALQLQVYFIVTHFSKELIVKYGPGVLVVTYLVTSLVVELMIENKTRKIIDFA
RLTFWPTDFCGLEAKNRLIKNSSKVSIVIYLILMWFAAQGIVMFPVWGDTSEWRLHVEIFDQWKLFYYIYVSTFTIIVFSAVRLPGI
LLYSIFQTHMQIVLINQKITQISQNDPNDIRMMNQTGYQKRIYKEMCLCVSQHIAIKRFIKKLLEIVRPVQPIFMVLGLLGVISIFFF
ALYNLENTSNILKIRLVMVVISCILILCLFAEAGQAVSDETSRVFDTLLTCPWYLWDQRNKKALAIFLSNSLQPISFSMAGFTLNYG
FGISMLRNSASYALILYKMKN

>TcasOR195
MFRERVYDDRFIVLKTIFLEFAYCKEMKIYNMFCLVFHLFSFSLQVHFIVLNFSVELITRYGCMLTVFLYLIAAKSFSIIIEKQVRML
EMEATSFFWPIDCCGPQVKKNIYDRAARQNIQNYFTLAWFALFGIIMLPVWGDQSEWFLCIQVFQQYFGCWKLFYYFYFSTFPMI
AFTAFRLPALMLYGILHEHLQLILVNQKIVQLSVRRSLKENIVDNANYQKTVLKKLKLCISHHVKLRDSLGKLIGVIQLAMPVFLF
IGALGSIAVLYFVLYIFLSSSNILKIRLVVITICNGLIVYTFSAAGQALADETGRVFDTLMTCPWNTWNIKNRKVLLIVMSNTIQPLT
FTLAGITLDYKFGLTMLRISCSYALILYNLH
>TcasOR197
MFKKRKFDDRFIVFKKIFFEFAYSKEMKIYNMICLVFHSFSFVLQVYFIVQNFSVELITRYGCILAVFLYLIAAMSFAIFIEKQVKML
EVETTSFFWPIDCCGPQVKKLIYDRSARINILNYFTLAWFTLFGIIMLPVWGDQSEWFLCIQVFQQYFGSCWKLFYYFYFSTCPMI
AFTAFRLPGLMLYGILHIDLQLVLIYQKIAQLSARRIFSENIVDNAHYQKTVFRKLKLCISHHVKLKTCLRKLIELIQMAMPVFIFV
GAVCSIAVLFFLLYVFSSSSHILKIRLAISVVSNVLIVYTFSAAGQAIADETSHVFDTLMTCPWNAWNNKNRKVLLIIMSNTLRPLT
FTLAGITLNYKFGLTMIRISYTYALILYNLN
>TcasOR198
MPNVTNKRQKRLFSKTRTKSEDPFVMIKDVFVDGGYHPVTKMLNYICLVIHSCSLLLELNYFVHNYHFDLMMKYCCAMSLMG
YIIATMLFAIFQEHSAIDLTKDILSLFWPIDYCGPRVKEEIVKKATKINRIHYIVLLFAGALGITMFPIWGDQKEWFLCVQVYQHYFG
KWSKIPYYVYFFTYPMLAFSSVRLPFMTMYAIVQIRMQVYLLHQHISEISGEYVYDMKNLQILCDQNYQNEIYDKMRLIISHHIM
LKRWMRKLVHTVQISMPVFVLLGTMTSISVLFYAIYSFHNINFILKVRLISVSVCTVLVVYMFSEAGQALSTETTGVFDLLMTCP
WYVWNIKNRRILLIFMANSLEPMTFSLAGVTLDYRFALGMLRTSCSYSLILYKLKTGI
>TcasOR204FIX
MTNFFSNFCSPLKNHWAKTKHLFSKFSLSSDQPFIMIKLVCVDIGYHPVAKTINYICLAIHISSFLLEMNYLRLNFSTDLLIKYGCGI
SAVVYDISTLIVAPMIERPTIGLSEGITTSFWPIDFCGPKVKQLILEDTKKTSKIYYRTLVTIFGFAAVIMLPIWGDQKEWFLCVQVY
EHYFGKWAQIPYHIYFLSFMWFAFTSVRLPLMMSYAIKNIRVQVFLVNQKIAKMSKEYEEAKIEDVNYQNRVYKNLRLCISHHVL
LKWWLRKLQKIVRFCLPVFVVIGILTESSVVFYLIYNFKKVNLLLKIRFLLLACTTGVIIYFFSEAGQSLYIETSQVFDSLISCPWYS
WNVKNRKVLLIFLTNSLQPMFFSLVGFTIDYRFALTMIRTSFSYAIILYNLSSGSQIASI
>TcasOR205
MTNIFSNFSLYFKNTWTKTKQRFSKTLPSSNVPFMMIKLVFVDIGYHPVSKIINYICLAIYMSSFLLEMNFLRLRFSTHLLIKYGCG
SSLSVYFISSMTVAAMTELLAVDLSEGILSSFWPIDFCGPQVKQLILKQSRADKRMHYVVLLVFSITGLAMLPIWGDQKEWFLCV
QVYEYNFGEWSKIPYYIYFFTFPWVAFSSLRLPFMMNYAILNLRMQVFLINQKIAKMSNAYDQTTIEDVNSQKRIFKNLRLCISHH
ILIKWWLRKFVNHVKFCIPIFVIVGIATSISIVFYLIYSFQQVNLVLKIRFLSIACCCWFVIYLFSEAGQSLYEYTEIFHSLISCRWYIW
NVKNRRILLVFLANSLEPMTFSLAGITLNYRFALNMMKTSCSYALILYKLNCDSQIMD
>TcasOR213
MAKFNDPFKFVRTIIFVDMNSYKVIKTCNVLLNIIYSLIHCLLIYYLCKNLEINLLIRYAPAILLFILVIFGAVFSIYMDEDILEVRSVF
RENRWSLSVLKENSQTKLGRKCQFINIFILLVLLLIVSTLAINAPCFGNQRELLICIQVFEEYFGEWSFIPYYFFFLGFPLLYYNFFRL
WMTFVYGLLEGQLQFFILEEYLCGIYETEDSKSWKYLQDSRYQQEIEKSLRLCISHHIGLKKFLKMVENQTLKVMPFYLVFGVLI
LICYFSFIINFADTVTTIGKIRMFMTAICMMGVAILLSWIGQQLIDVTSDIYFTLGGAPWYYWSQKNAKLLLMFLTNCTKNESVTL
AGISLDFTLFVSIVHTTLSYALVLYNLRESSLVSSSQK
>TcasOR229
MSARPLHLRNFPYYFLKVLVFDFEQYSAGKVLSYFCAIVHSISIFLQMHYLVKNFTKETMFQYGCVLTVLTYCVVALFFAIASGNF
VEKLESEISSFVWPLDICGEDVKAAILKRAFYTSLVAYITIIAFPIFSVIMFPVLGDQSDMFLCVRVFNEYFTKWSQIPISLYFYSFPVI
AFSGIRLPGMLLYAILITHIQMFLLNRRIEQISELSNQRRVFETLCSCIELQAKLKRLIRNVFQLVYIAMPIFILLGAVSSVFVLFFVVN
SLETASYFLVLRMGCFFGANVLVVFIFSQSGQSFSDETGRIFDTLVMCSWYNWDKRNKKVLLMFLANSLEPMSITIAGITLDYKF
ALAMLRTSCSYALVLYQMKN
>TcasOR230
MREKPLHLSHFPYYLLKIMLCDTEQYRLGRFLSYSCAVIHSISLLLQMYYLIDNFNKETVSRYGCVVIVTTYCVVALIYEILYAQPS

VSMMSQQISTLWPMDACGEKVKQMILKRAFFTSVVTYSILFSFPIFGIIMFPLWGDQSDMFLCVRVFNEYFTKWSKVPIYLYFCSF
PVLTFSGIRLPGMLLYAILITNIQIILLNQKIAHISDLGDQRLVFGTLCSCVSLQIKLRQMLNKVLQFVYLVMPVFLLLGALTAISVLF
FLFYSLENPSDYLMIRLACFLGGNILVVFTFCESGQALSNDTGRIFDILLTCPWYKWDKKNKNILLMFLVNSLKPMSITIAGITLDY
KLAVTLIRTCCSYALVLYQMKN
>TcasOR234
MQQAALRNFPWHYIKRIFIDFGYHRTMKIFTIVYFILYSGSLLLDLYYLFNNFSIAAMVRYGCMIMLISYVIAGMLFCFIFEKQLLN
LLSEAETIFWPPEMITSELPKFIHRTNVLNYFIIAWFGLLGVILFPVWGDQSEWFLNVWAYKAYFGSWWYIPYNLFYYSQPMAAW
TCVRLPFIMMYFSLQIKLQIFLLNQQILEIPKGHNTNSETAPDDLSYQEAVSQKMCLCISHNVKIKRWTKSFLRKVIQAMPVFVLL
GILGSIFVTFSVLYSFESTSTILKIRLVVVVGCTILSVYMFVEGSQRLCDESSQMFEMLAYSPWYLYNKNNRRILLTFMTNTLEPITI
TWGGIILNYNFGLTMLRMSFSYALFLYNIH
>TcasOR264
MVYLKDPFITLRVMFLNFNKYKIVKCCDFSFIIFYSLVFCLQIYYLISYFSANPLIRYATTILLVLWGIVGAILSVTLEKQILEATAFLD
EMCWPLNMVRKEAQTKLERSCRIINIYITCSLLLILITVVFNMLCFSSQRDFFINIQIFEEYFGELSHVFNGLYFTGFPYLCYHGARL
CYVFVYAILQIQLQFSLIEEYLLQVYEIDCLKSWRYLRDTRYQQEMGKSLRLCITHHNALKKFVKMINDMSLICMPFCLVLGVLIL
ISCLAFVINFGDTLTIFVKLRILIFVVSCLCVLSVFCWSGQQLTDVSSYIFLTLARAPWYYWRLENIKILLTFSTNCTKNDSIVLAGIR
LEYMLFVSMLRISCSYALVLFNLRK
>TcasOR276
MTMQFIVKRATRGIFHDLRVLKFISSDIFDIKIMKLCLFITFLIHLTACAITIHAFMFNNFSRREFISCAPVLFGCFYGLLGLGTILFKP
SMTRTLMLELKAWDITAADDAVSSRIKFEINVITVFCLVNYLLALVASFFYYMSFYGDEEIFYLIRFLEDHCPNHKRVLIKLYKISFV
LLGYVMVVHACQVLYATQHVRFQLILCAHFMANVTKQAKNIKDEHLPDDNNYQNMIRERLKFCIIRHQEIRRFYFDKLEEMGN
LIGGFALLGCFLGISFAMHMLTSEFLRYHFARTVSSIIAGVTTFATVIAAGQSVETEVDISTRVVKEVKWYTFNESNKRSYMLMLL
NSMQTYKIKFSENYSINYELGLSIVRGVFSIVSVVVQLDY
>TcasOR277
MDQVLEKFPENDWLRGVKFISSDIFQRKLVKAVLFMVLLVHLTASVITIRAILIKDITAKEFTFYGPVFFGCFYGMLAIYIILFEKNFI
ANLSGELKMWSFRSAGAEITRQIRFESRVVTIYAIINFVMVVIASCLHITPLESDYETFYMIRFFEDKIPDYANVCKTSYRSTFLVM
GYVMMVHVYQIIYATQHGKFQIMLYLEYVKRVTQFNEKIGEKCLFYNESFQKMVARKLKNCVIRHNEFLKYHRKNTREMSHWI
VAFSLCGCLLGISVFFYILSGVIYREQYFRVAVLLTTAASTFVAFIVAGQSLESRVDNGYSVVSRIEWYNFSETNKKTYFLLLVMLM
QPWKIKFSDKYSINYELGLSIVRGIYSIISVMVNIRFDS
>TcasOR278
MNQPQESFLKNDYLKVLKLISSDVFEPRLVRAILFVVFAVQLTASIITVRALLIKELTAKEFVLYGPVFFGCFYGMLAIYIIIFQSSFIT
NMSQELEMWSYSSGGEEINRRVKFQSRVITIYALVNFLLAIVASYLYFSPLDSDNETFYMVRFIEEKIPDYAKICKIAYRTTFLAMG
YVMIVHSYQVIYASQHVRFQIIFFTEYVKKVVEFDEKISEECLFYNERFQTIVGKRLQNCVIRHIQFLKFDRIKIKEMSNLIAAFSLC
GCLLGISISFYVLSGIFYREHFLRVALISVTAVSTFFALILAGQSMESKANSAHIIMNNIKWYNFNQSNKKAYLLLLMMSMKQYKIK
FSENYSINYELGLTIVRGIYSIISVMANMHFDN
>TcasOR281
MDYSEKSLIQGDCLKLLKVISSDIFQPKLVKLILLIVFGVHLVVDLLTLRALLVNELDFKEFIFYGPVFFGSFYGMMALLTLVLKDD
FISNLKQEFRLWPLDCAGDEIYSQIKFENKIIKIFVVFNCIVTFIGSYLYFLPLDSDNETFYAVRFIEENYPDHRNLLHGLYRSTFLIFG
YAMTVHVYQVIYNSQHLRYQIIIFTEYVASIGNPDKRKENELFYDKGFQKVVYERLKFCIMRHQEFLVISNKKVGDMRVFIVGYS
LCGCLLGISLTFYIFSGKFYREHFPRVSVACVGAVTTFWAVITAGQAIESEYDSLLSTLLGKIEWYYFNDSNKKNYLIMLINLMQPW
KIKFSEEYAVNYELGLAIVRAIYSIVSVIASMHFEA
>TcasOR282
MHDYCFEPLTKNDYLKTVRFLCCDVFEAKIVKLGLWITFGTHLIVSVVTVRALLYDLTINEFVHYAPVFVGSFYGLLALWTILFRI
EMVRDVRKQFKFWTIDCAGQEAHSRIKSEIRITTVLSVLNFIITLYASYWYVYPIEGDKEIYYALKFFEEYCPRHKMVLSVVYRAT
FPLLSYAMIVQAYQVIYTTQHIRFQAILFIEFVLNIGHQTKNLSEEKLFYDTDYQKIVGERFKFCIMRHHEFIAFRRLKLNEMSNLIV

GFSILGCLLLFSFGLFVLTGKLHREHFWRFGLGSLAAVCTFGSVIWAGQSIEIESENVVNSLNSVKWYTFDENNKRNYIIMLVNTM
QPYKLKFSENFSINYSLGVSIVRAFFSILSVAAKLYFNHV
>TcasOR300
MIGLTNGDYSPRPSMEGDCLKILKFFAVDIFNPKIVRFFLWIMLLYHVVFTLVTAYFMLYVLSNSEIIGYTPAFLGNFYPMLCVWSV
LFISRLIYVKEDMPLWAIDTAGAKVQASIKRKIFLYTAFGIFNLVLSLSAGSFYLKNVSEDVNVFLALRIFRDYFPNYYQVLDLIYR
LIYFCFSYLMVAPSYLLIYYILHVRIQAIIFAAYVAHIDGHSDYGTDIDLFDNEEFQSEVERRFKFCIKRQIEFLLMESKKLSQISNLIA
AFSLAGCLFGISIIFHLFTGQLIQEYYFRIGLTSLAAIATFSAFIYTGQSTEVQIELVDNAIDNLCWYNFNRSNKLLYLIAKADLARVR
KIKFSGQWAVNYDLGFAIVKGIYSIISVVVSMW
>TcasOR309
MPFEWTIRKNKIKPILQNDVLLNLMLVPNTIISNKFLVILNYFYFGFIILQSVFVAVIIITKDEWKLLNGQYAGYTSGCAIVWSSYIT
MYTYVDKFLNLYKEIFPHLWSLDVVGQDHFNKFSKMAKVLKLGKNILLVVGFLSATVGLPWYRDEYEIIITVRVYKDYVDKWT
TLLYFVLFSSLYHIALTVIFCVLCLVYMVLHLHNQCVMLNKRLEALDDEQLFLDNDNYQDFVTKELKFCIQQHQFLLKFAKRLN
DILYYPTFYYVLSGVVTGVSLLLFPKNDIKNLLRCVLIIVLGGGFAISFCFLGQILENASEELLFSAYSARWYLWNIKNRKLLSVFL
LKTQDNIVLSSSGIITINFRLLISLYQSIYSCLTFLLNIK
>TcasOR311
MHHKNIQPMTDDYLKFIKFVSSDIFQLLPVKIFLAVVFLTHAVLDLLTIYFVLFVIEPHDFITYISVFLGEFYAPLFAIVMLLFRGKIT
DSLKHKLAMWTITSTDEKTQSDIKRQIVFFNGFVVLNSVIISIASWFYAARLSDDVNAFFALRLIHEYFPKSIFEVIYRVTNFVLGQ
MMCVHVHQTLYYTQHINIQVQMFKKIIRDLENESKIEQQLKFCIERHAEFIKIITLTTKELRGAFVGFAFGGLLLGVAVAFYIFSGLL
TPEYYLRVGAIGLASVVNFAVTIWFGQSTESHLDELMLAVGEVQWYNFSQRNKKVYLILLMNVMKGRKWRVSEEYSVNYRLG
LAIVRGVYSIISVTSSYKKS
>TcasOR313
MEQLPKNDPLLVLRVLPELLLLHKIVRHFVVFIVCYLTATTIFCLYVLATVRGLWDLFWSQYSLLTFGSVIGFSCYFVAFWKGFKFL
ELRRRVFADYWALTSLGEESFQKIKKLSKSANIFTVGTILASIATSSTCMPWVGDEYDIMFPVRVYTDYFGERAVPLLVPFYLAMY
CTGFVMIATGFIFVHFALHLKFQFFLLNRRLDGLQTEPLVNDFSYQNRVKEELTCCIEYHQKLLKVAKEMNEIVYYPIFIVVSSGIIC
SVCLIFYMKTFENSIVRGTAMAISGGLITFGFGFTGQLMENESGRLFDTSVMLPWHLWCLSNRKLYHIFLTKSQYHVSFSSSGIINL
NHTLFISLYRKVTSIFSFLMNVSNKNST
>TcasOR314
MEQLPKNDPLLVLRALPEILMQHKIIKYVVLFIICYMTVTMILCSYVLATVRGLWDLFWSQYSLLAFGSSIGFSCYFVAFWKGSEF
IKLRRRVFANYWPLTSLGEESFQKIKKLSIFANVFMVATILASLATSTAGLPWVGDEYDIMFPVRVYTDYFGERAVPLLVPFYLAM
YCTGFVMISTGFIFVHFALHLKFQFFLLNKRLDGLRTEPLVNDFLYQNHVKEELTCCIEYHQKLLKVAKEMNDIVYYPIFIVVSCGI
MFSVCLVFYMKNFKNSFVRGTTMAMTGTLTTFGFGFTGQLMENESGRLFDTSVMLPWHLWCLSNRKLYHIFLTKCQYHVSFSS
SGIINLNHTLFISLYTKITSILSFLLNVSKKNHTK
>TcasOR315
MTLVRKLQAAATNAFEIRIKDDILAELFNWPFLVLDSKWSTKFAVFLTVYCVFETLACALVYSTLDVNMMGTYAIVIARFATTFCS
FFSFFTKRKQYFEIINENFPHFWPLQSLGKSTFNRIKMRASSVKFYSFLNVVVMLIGAVILISFTQDESEVYLSVKIYKDYVNKWTT
GFVMFFYVSFIYIGLVVAAISFVLTYTAFHLIFQCFLLNQKLKQINDSIVENEQKQAKFDEKYQSFIYKELISCVKLHQRLILFGKRI
NHLVYAPLLVYIFGGIVVGVALIYYLKSSVQHIFTSLILLLIALINSTTFVINGQMLENEAENIYISLTNLPWYSLNVQNRRVVYVML
MQSQKIIHMSASGLVSLNYQLTIVFFRCIYTGMTFLVNVGL
>TcasOR316
MTLMRKLQTAIRNLFEIQIKDDILAELLDWPTLVLFSKWPKNFAIFSTIYCVFDTLVCTLVYSTLDVEMLGKYAIFIAKSTIALCSFF
SFFAKRKQYHKIINENFPHFWQLQSMGESTFDQMKKIATTVKFYSCLSVVAMLIGAVILILFTEDESEIYLSVKIYKDYVNKWTTG
YIMFFYASFLYIGIVTAAVVFGLTYIVFHLIFQCFLLNQKLKLINSYIVKNGQKLVKLEERNQNFIYKELISCVKLHQRLIYFSNQIND
LLYAPIFMYTFSGIVVGVALIYFLKTSIQYILTSLVLSIVSLIITTTFVINGQLLEDETENIIISLTNLPWYSLNVQNRRVVYVMLMQSQ
KIIHMSASGIVSLNYQLTIVLFRCIYTAMTFLVNMGL

>TcasOR322
MTFHWITTPLEPILKDDPLFVLMALPNKLIGSKLQALVNYFFFVYMVILPVSCFLVIVATNQWQIFYSPYSGYASGVFIVWSCYVSF
FIFGSKYRRVYRDVFPHLWSLDVAGEEHHNRLKKIGKQLRTFKLVLITLAFIGATSGLPWFGDDYDFYIPIKLIVDYCDQWKLFFSI
FFYLSFYHIGVTVLSCFFSLMFLVLHLQNQFYLLKTRLQTFATDSGTSDVFLSMKVKDEEYNRSVTQEIVFCIRHHQSVLMYCDR
LNDLLYLPIFYFTLSFIVTGVSVILFPKYDLQALIRSLFVIVLGMCMTLLFCSLGQLIENESENVLYSLIEAPWYLWNTTNRRLYYLF
LLKAQDTVNLSSSGLITINFQLILTLYRGIYSALTFFLNFS
>TcasOR328
MSYNIKLTKDDRLKLLKIMASDVFQSKTVKIILIVVFLVHAIANSLTIYFALHVSDTKQFISYASVFFSEFYPMLAILTIIFKGEVVQH
LTDDINIWTIDGASKKLQSEIKLKIKILTAFVIINSFSVVIGGFCFVQQLSDDVNLFFAIRLIRDYFPNHSTILEFFYRMTYPICAYLMA
VHAYQCLYYTQHINFQLQMFTEIITELTDLKTISLPENRLFYNKKYQTVIEQRLKFCIKRSQEFIKVCVTKNKEIGSLIPGFAICGLFL
GIGITFFLSTGKFTTEYYLRMGVTSICGLTTFSALIWSAQTTETMINDLVMVINKVSWYNFNQSNKKLYLTFLLNTMKERKIKFTE
KYSVNYQLGLAIVRGIYSVISVVASKRHH
>TcasOR329
MNCENQFAKDDYLKTLKIMASEVFQSKAVKVILIFVFLVHAIANLLTIYFVLYVSDTKLFVNYASVFFSEFYPMLAILTVIFKGQIV
QHLTDEFKIWAIDSASKKLQSEIKLKIKIITAFVITNSLIAVWGGFLYVQPLSEDENLYFALSFIHQYFPNQSSTLEFFYRMTYPILGY
LMTVHAYQCLYYTQHINFQLRMFTEVVAEFAPVKRFLLFEHHLFYNKKYQTEIEQRLKFCIKRSQEFVQICVIKNSEIGSFIPEFAIC
GLLFGIGVTFFLSTGKFTSEYYLRMGVTSFGGVMTFSALIWSGQTTETMTSELVKALNEVRWYNFNQSNKKLYLTLVMNIMKER
KIKFTENYSMNYRLGLAIVRNIYSVISVVVSKRRH
>TcasOR330
MNYKKQFAKDDRLKTLKLMASDVFQSKTVKIILTVVFLVHFIANSLTIYFVLYVFETKLFINYASVFFSEFYPMLAILTIIFKGDVV
QNLTDEITFWTIDSASKNLQHEIKLKIKFLTAFVIINSFTVVMGSFSYVQQLSDDVNLFLAIRLIRDYFPNYSTILEFFYRMTYPICGY
LMAVHAYQCLYYTQHINFQLQMFTEVITELNNSKTSSLLENHLFYNRTYQTNTEQRLKFCIKRSQEFIKICVTKNKEIGSLIPGFAI
CGLFLGIGITFFLSTGTFTTEYYLRMGVTSICGATTFSALIWSAQTTETMTSDLVMVINEVNWYNFNQTNKKLYLTFLMNTMKER
KIKFTENYSVNYQLGLAIVRGIYSVISVVASKRQH
>TcasOR331
MNFFQKKLAKGDFFKTLKFIASDVFQSKAVKMVLILLFLIHAIIYLLTIYFLLYVLEPKQFVNYATVFFAEFYPMLAILTVILKGKIIE
NLTDEIKIWAIENASKNLQSEINLKIKIITTFVIVNTLIAVSGGFLYMHPLPEDVNLFFALRLIRDYFPNHYTSLEFFYRMSFPIFAYLM
TTHANQFLYYTQHINFQIKMFREVCLEVKAWKTVSPFENHLFYNKKYQTEIEQRLKFCIKRSQEFVKISVYKNKEIASFIPGFAICG
LLLGVGLVFFLSNGKITWEYYLRMGFTSLGGVTTFLALVWTGQTTENITSDIERAINEIRWYNFNQSNKKMYLILVMNTMRERKI
KFTEKYSVNYRLGLAIVRGIYSVISVVLSKYQH
>TcasOR332
MEFGNYKLMTDDYLKTIKFMSSDIFQPIPVKILLGFIFALHSAVNLVTAYYMLTTFDAKLFINYSSVFFGDFYPLLATFALISKNNTV
RNLKDELEIWTIDSAGEKLRSEIKLKIKFLNIFVVCNSLLVLVTGLTFIQPLPKDSDIFFAYRLIHEHFPKHGQALEFLYRTTYVLISYI
VAVQPFQIFYYCQHINFQLQISIETLKKISDWKTLSEDGENLIDNVKYQTEIKRRLKFCIQRSQNFICLHTEKIKEVSTFIAGFAVCAC
LLGIGVIFYLISGNFTPEYYVRMGFTSVVGIIIFAATIWAGQSTESAIDEMVTSLNEVEWYNFDQSNKKLYLIFLINSMRERTIKFTE
NYSFNYQLGLAIVRGIYSVISIVL
>TcasOR333
MEFEVKTFMTRDYLKVVKFLASDIFLAKPMKILLLLIFIVQASVQAMTGYFMATAFNAKFFNNYAPIFFGTFFPLLAISILLLKNKI
FHNLKNELKIWSLDNAGEKIHSGITTEIKVVTYFVIVNSVFVLLANSTLAYPLSQDVNVFFGCYLIHKYILTYGRTFEFFYKATYLV
IGHTNTGHVYQLLYYTQHINYQLQLYIEFIKFLDEGKTISKNEDDLFNNPTYQTLINQRLTFLIKRGQEIVKFHIKKTNEIRTLIPAFS
VCTCTMGIGVVFFIISDNFIREYYFRMGMVSLVTVSTFAAGIWSGQSMETNLNEITTALNEVKWYNFNKSNRKLYLIFLTTSMRER
KIKITENYSLNYQLGLTIVRGIYSVISVIINMK
>TcasOR334
MDHPDIKPMTDDSLKLIRFIASDILQPLPVKIFLGVIFLFFTVGSNLLMIYFVLYVYDIREFMDYAPVLFASFYSGVAILSAIFKGKII

HTLPDDISLWALDSGGEKIHSEIRFKARMVTIFVICNTLLIIGGIILNLIPLSDDLHVYFALRFIHEYFPNHKTCLIILLKASIFPVIPHMLVVHAYQILYYTQHSNFQIQLFNKVIAEVDFWETPLRETELFYSKPYQKGIEKKLKFCIQRLQVLINAYIVKTKEIGTLIALFAICGVLMGIGFSLYLFSGKFTPEYYLRLTFMTLVAVTTFSSIIWGGQSTETIITEMITALCQVRWYNFSQTNKKLYLILLTNMMKDRKIKFTENYSINYQLGLAIVRGIYSIMSVVVKMRS

>TcasOR335

MDHPDIKPMTDDPLKLIKFMASDILQPLPVKIILLVTLLALPVGSNVLMIYFVLYVIDIREFIDYAPVLFGGFYPSLAILIAVFKGKLIHNLQDEIKLWAIDSAGEKIHSKIKLKVRMVTIFAICNTLLLIVATVHNLIPLPRDLHIYFVLRLIYDYFPNHKTYLLILMKLMSPVTTYMLLVHAYQILYYTQHINIQIQLYNKFVADVDFWETPLCEPELFYNELYQKRVEKRLKFCIQRSQHFVYVHVAKIKEIGILIALFAVCGVLMGIGISFYLFSGNLTPEYYIRIFIIALVGATTFSSIIWGGQSTETIVTEMIATISQVRWYNFSQTNKKLYLILLTNMMKERKIKFTENYSINYQLGLAIVRGIYSVMSVLVKMYSINT

>McarOR1

MLKFKVVGLVADLMPNIRLIQASGHFMFNYHADNSGALHTLRLGYSCMNLVFVLLQYGAIFGNLVAEKDDVNDLAANTITVLFFTHCVTKFVYFAVRSKLFYRTLGIWNQANSHPLFVESNNRYHALALKKMRILLICVGMTTILSAAAWTGITFVGESVHTIKDPNNENETITEEIPRLLIKSWYPWDAMSGMAYYASLVFQVYYVFFSLSQSNLLDSLFCSWLIFACEQLQHLKEIMKPLMELSASLDTYVPKSADLFRAPSATSQDNLIENEYNAKNEELNLKGIYNTRQELGGHFRSGTLQTFGQGGGGVGPNGLTKKQELMVRSAIKYWVERHKHVVRLVTAIGDAYGVALLLHMLTATVMLTLLAYQATKINGVNTYAASVIGYLVYSLAQVFHFCIFGNRLIEESSSVMEAAYSCHWYDGSEEAKTFVQIVCQQCQKAMSISGAKFFTISLDLFASVLGAVVTYFMVLVQLK

>McarOR2

MKRNMSYDNFDYTVFFTHNILMYKIFGFWRPDDDMKREKLYNCYTLICTIIWLLFLASQYIFIITNIQNVDEVTATSFVTITFSINLIKMLAIYRNMNRIKQLIKDMNLPMFQAKCARHRDIIDYTRIYTIFFYICLYFGNTDRHYFWTIVPFIGDERATLTHGWFPYNETKSVNYEITYVFQTTVSVWNTMLCLNLDTFTGSLLILIGLQCDLLCVTLENLGDFHVENGVLCENSEEYQSSLVNDKVKFSKTMTENLVVCIKHHKEIMRVSKDVEDIHRVSVFILFLGGALIMCCCLFQLSVVPIGSIEFFMLLFFLISILTEQFIYCWFGNEVIQKSSRILHSAYCTPWLDCDINFQKVLLQLMTQTYRPITLKAGGLFTISISVYISVIRTSYSYFTLLKK

>McarOR3

MSQKVDPQYFKKHLKWLTWLGIDIIPIEKVWYAIPYKLYAFVLLVYVYLYSLLEIIDIVKSSDFNSMTFGLSYSVTHILGAAKITILILKKKILRDMLIRLEQGYFVPNKARGGEKEQQLVNASVIRANLHADIFNTLVYLIIGIRCLYAIFDKGVYVEVLDEKLNVTTLKHIRTLPYKAWLPVDLNKSPAYEFMFIIQASCLVLYGYYIGFLDSLIYGMMIHMNNQYLILRNILEHYVELAKNIVLNRNPNSVTDDTSTDYIKLHNGIERQKTLAGPVLDVIENIAYHCAKYHLAIIDYCDDIEKEFSNLMLLQFLSSLYILCFQLFQLSLVTNYFSFDCISMCLYLILMMYQLFCYCWYGNAVMIQSLDISSVIYNTDWLVTNESTKKCLLLMMMRAQRPIIFTAGKFAFLSLPTYMAIVRGSASYFMVLQQMQ

>McarOR4

MAPSYVFDLPKAFEFEKKLLLYTGLYPNTGLVNKYIYYLSGLFHIGITILIEISLIIVISIHIDNLSTITDALMFFVTQIALTWKLTNVCIKRKVFCEIEEILSQPIFYNLSQECENIIHYYVKFSHRFARCFRIICIMVCATNGTLPLVGGKLGHAMLLGWNPWDSEDRIKYYLNSTFQLTALCVSACINSTIDILTVILLAIATAQIEILKNNLVNIKYGEKEAKKLFNENVRLHYEILRFVNAVDRSLSSGILSQIFGSVLVICVTCFQLIIVSVQSIQGAFLLIYLLCMTFQVGLYCWFGHYLIDSSDTIIQAVYMSDWYEANNSLRKAVIIFMERCKQPIVLRIGGLFPLSLGTFTSIMRSSYSYFAVLRKWYEPE

>McarOR5

MTDKGYTPHFFRTNEIIEVYTGAWMYNENLVAPGKKWLLYIWSVLIYIGAVFFLFLEFLKLRDTMKVSNDFIRQCGLISCHSLCVVKFVILVLRHRKIKRLMDTLQDKKYQYEPLGDFSPGQRFDEARKLTHWCTIGVFCLYSCAAVSAHISAEVLINKDAKRERFDGNITCYEYMTFYFAIPFPSDTKAQCEMSFIFMHFCIDIYAWFAAGHDSFYAALLNCLRVQVDILCDAFRTIRPRVLKRLELPQDLSIFHDDDFPKLEEALYRELTHLTEHLMILLRVADDLEEVFNLITLAQTVSSLIVFASCLFITSTIPLSSPEFFAQVEYFTCMLIELSLFCWFGSAATRASEAISPAIYESDWYGTSKRFKQSVLIIMCRMQNPIYLSIGKFCPLKLDTIVMVFKCSFSYYTVFKAVGE

>McarOR6

MPLSVFFIYFLIILSSMQPFAAIAYQFYVGIEDMNIISEAFIGISDLVGFLFIYICFRKHRGLIKETIKASAVFLKYCSPNVMEKAEEEV

QTYTKGLLIYFSIGLTFNGLIPLYDYENCDQRRLSDYYRAHDPCGMPIRIWVPFDARKPVIYYLVFFLHANACLNICYGVLCITMT
LVGLLIHITAQIKNLRQNLLQVFDELPEDGDCYSETLVLKLENKLKFCVKYHIIIINYTDQVFAAFNLMLIVHISLTSLIFGVLGYQI
VTVEDFTEKLRYVMHLGGWIALLFLTCYYGQLILDESTTVANAAYQSKWYNGPTYLRKNLCLIIMRSQKPLKLRAASIGVISLET
FLSVIKTAYSYFALLLSIAE
>McarOR7
VLLYCTTEFAFLCKLMNFVLSKKEIIELEAILESRLFTVDTPEEEAIIKTSTRQIRKLANIYKTLCFLSVTFYALFPLADGGREAQKLP
LPGWFPFNVCNHYYEVFIFEAIGIGLCAWFNSALDLLVVIMMILGKAQFELLRHRLMNIAIYGEDGERRRRVKMCAQHYKSILRF
VMLTESIYSNGIFVQFMSSGIVICFTGFQMLIISLKSIQFVQRILYLSCMMYQIVMYCWYGQVLTDSSNKITEACYLADWINCNVIL
RKSLLIIMERAKYPAKIRAANIFTVNLETLLTILRSSYSYFALIYSIYDTKNETK
>McarOR8
MTVPYADDFFHTNRWILYIGGLWWPDTYKSIYHKILYMSYCAANFLFCNLYFTPTEVLSLASTYKSIYHLIKNFSLSQMHVLGFT
KVLFFVFKGYKMKAIISVLEDKKLHYEDCDEVNFHPGMLTNKYKKIGRVAGIIYLVLPLVVILLAYTLSAIAALRYVEGDSNHQLP
ERLPFYSWMPFSYDTPKKHLIALVYQATPLVSYSFSVIGMDFLFANIMNCIAMNFTIIQQAFRTIRERAAIRVKEPLKVKDELYNSE
PLQRELNKEMRKIIQHLQTVYRMCDELEDVHKYLTLAQTLSQLFILCASFYLTSITPFGNQLVIEGIFMIMVISPIVFYCWFGDEVT
HQGGEISVAIWQSDWLGATKSFKTCMIINMIRTQKPVYLTTGKFAPLTLATLVSIFKASYSFFTVLKNTSNQ
>McarOR9
MTILGIWPVKANWFKKLRLYEVYHRISFWILFEFYYNADNKNFVQVMGKNFSETAEVLGVVIVLLITSFKVKICTSPKIKNLLQQI
EDAEKIIIETTELDTRNIYNQHIKMSTKENTVQLMIGVLAISLYSVRPILANRGLEPESKNKMFIFASWFPFDEQTYYAPAYLIQFISG
LYSTGYTISTTMFLFNAMIFARCEIKILQNQFVNFTYYVKKDAKDNCRTYEESQKVALEDRILKHRKIINFVNTLDTSFKTILLLDF
TVTSFQFSMVVIQMVQRSQLDVAVVSMVMYLSTLALQLYLVYSNAHEIIIESNKIAQAVFESEWYDLPNDVKKAFVIIMLRAQKP
LYLSIGPLYQVRCDMLFKILHALYSYICHFLEVNYIVTIV
>McarOR10
MSSYPKKLFFLNRWILCCVGMWPPDNQNKLFRFLYKVYAIAAFFYIMVLYNVLEIISLIYTYNDTVSFMKNVSAVCVHLAGAAK
SVIFYLRGDKVVEMMITLESEELRYEDCEARNFYPGKISKSCKITVAKLTALCFVMVHIVLLSSFIPPILQILLCIIKRDATVLPDRLP
YLIWIPFKMDTVSRFTLALVFQIFGMFGGAYNISGMDSIFLGLMHCISQNLVIIQGAFLTIKERSVKRIKGPALAADRLNNSECLNA
AMNSEMRKVSRNLQTIFNVCWDLEREYKYLMLLQVLITLLILCSSLYTFSSATPNSKLFYTEIIYISAMMFEPFMYCWFGNEVTH
KADEMSNSVYQCDWLGTDKKFKTSLILNLTRSMKPIYLTAGNFVPLTLATFVAVVKGSYSLFTVIKGSN
>McarOR11
MYFNSLLGVWPFVFERPDFRLWQMMYKMYSNLMLVFGTYVICTQYTQLVMLLQEEEIWVQEIIRNLCLTLLHSMGLAKVYAIR
SDNLKELISEALKVEEDIYRRGDEDIMEIYRLYAWHSRVSNIAFLINIAIETCFYAMHPLYVGELPHFDKATNQTKMIRALPMSAW
VPFDIQEQYLEAYLWQSVEGTVTASFVMYTDIFSFSLIIFPLGQISILSHVLRNFDHYVKKAQEKHGCDRDEASFFIARECVVKHQ
DIIRYICVFNNAMKYIMVFDFLQSSMQLATIVIQLFGSELKMVEVIFHGEFAFCMLMRLMVYYWYANEIMLKSSDITLAIWEGVW
YEESQRVKHMMLMIIRRSNKPLALDIGPFSTMTLQALLGILKATYSYMTIMYNR
>McarOR12
MRANALLGVWPFIFEDNPKLQKIYDVYSRCTFIYYLLFIITAIIKLIFLICDEVFVIQEVIANLCITLLYSVTIMRVWAIKTPRVKNIIR
EIIITEERILKSKDETVITIYNSHAMQSKVSNIIFLVNIFLVTALYFIHPLYVEDRAKFYESKNITVIEKPLPLSSWFPFNEQEHYLVTYL
WHVLDGSIGASFVTYTDIFTFSLIIFPLGQLKILIHIMSNFEKYVDKIQNQLDCSPEEASFTTLRECVLKHNEIIKYINDFNTAMRNI
MVLDFLQSSIQLASIVLQLLVAEFTILNFAYSGQFALSMFIRLLVYYWYANEIMVHSSDVAFALCTSNWYEQPEKVKKMLVVILMR
CNKFLCLEIGPFTTMTLGTFLGILKATYSYMMVIYK
>McarOR13
NTVTSFKEDFFHANRVIYRICSLWLPGKEIPLQLRVMYLTYVFAWYFLFLTFLICEFLIFKDMLQEVSKFVNYFGMLFTHLVGTLK
LSVIILQYKRINNLMSILQDPEYCYESLGDFQPDVLLHKSKIISFIVSVSTFVLYSFVGISAHISSHIVMNQVVKNPTLEKNMSCVDF
VPYYFYTPFTGTTKLQCESMFLLMDICYFIHATIIACHDGVFAGLLNCLRTKLVILGGAFKTIRPRCLKRLNMPTNFTVLHEEENPE
IEKVLYAELNHCIKNLHILLQSRDDIEHCFSYVTLAQSLASLFILASCLYNSSTVPVTSPDFFSQLEYFVCILTQLSLICWFGNEITLA

SNHIILSLYEGDWFSASPRFKRSMILTMCRMQRPLYLSIGKFSPLTLATLVAVCRGSFSYFAVLQSI
>McarOR14
MDGGILRVQKLFMILSNKWEVNTKSVLINKLCRIQGIFFESYFILFTLYLPFNLLLHRKCMLIFYELGGYFLHHTNIIIMNILFRKNA
MKKTLKYIKNYEQVEYSKETQDSKDIYGYYSLLNARLGKYVIVFGTCIAGISWYVSTISYSIKENTEDCAVLEGVMYQVWYPFK
TRYNWLSIIFDLSMAYIAVSMHIFNRMSPITLVLFQLAHIKILANKIRNIDTHAEELASLHDVNIEQALNIAVDECVKSHQEVMSLM
DLLLQATKEMMLIGFFSSSMELASFIIQLFTAASKYHFIRCFIIFPIDLLQMLAFFWFADEIYVESTTLSNVIYNEVDWTRYTKPLRM
KLIVMMIVAQKPIYFNATGIGEMTLEKFKCILNSCFSAVTFFQTMYYN
>McarOR15
MEKLVHIKLLRRMMIICGQWNFKNYNNPALTIYRAYSRFIIYHVIFMTQMILLTIAMQWDCRSRVIEMLMLLYSIHQHLVMIFLTKI
YNLEKSLNYMMDYERVKLKQAGEDEKNVYFKYARVNNNMNVLIIIVCILTAIMWYMTSIRNTFTVRGNEVCPISKGLVYQIWYP
FNFDNQYWLIVINDLIFFLNVVILLTYTKIISITVTIFMLGQIKILQEKIRNLEQDALVLQRINRTEYDESLLLSLKLCIKRHQEIGWF
MEVLQDSTSSIILTQYFSNTFEMAAFLIQMLTEKSLYLIIRSFIVFCMVILQVYIFYWFANEVQIESTAIPDIIYSETKWTENDQIRRYL
LLMMTRSQKKLSFKSAAIGDMSLATFTKLIKLCYSIVAFFRTAYDL
>McarOR16
MSGQTQNIFLHAQRYIMIFVGKWYIDFGSNTKNRIYYIYSVLVELYFLFMTQQILVSMVIYRGCTERVGELICYYIQYTNSNICSFL
SKRSKIRKIFNYIMDNEAEHLKHGTADVYLKYAKVNRKVIVLFLVLTGVAGAIWYILVVRDTFFAEENENCLILRGLNFQIWYPFD
LFNRCYVITLLNDILMYTSAVGVHIYNKISPVSFMIYILGQIKILQEMLRCIEKDAISMHELQGEKYEEAILKNINNCVKMHQEVIK
FMGLIDKGCKEIVLIGFFTNSLELAAFVIKVLMEEDVFGALRTIGILCMTVTQLFMFFWFANEIKVESTYISDVIYYQTNWISYDKH
ARRHLWLMMIRSQRPLTISAAAIGDMSIDTFKRIIKLCYSIATFFKTVYM
>McarOR17
MVKPIETVCCRTTLKILRTCYMYPEEGKEQNPGKLFLLKWLTLMILSSVTFIGSFLHLIISLKDEDYRHLDVDFSITLSMIATYIFTC
FFFARVKFASKFYMHLSNLERLEKPLDFEKKNERLEKFALYHYIYMELLVASLLLFSNVIKGAKCKQENLEFDLHEVCGLFTYTW
MPFDIDYFPVKQIYLFLQLFGTHYLYLIAGTMAWTVVEAIQQIVLRLRHAKYLFTEAIKEVDPVLQRQKFNRAVRYHDAVLGLDD
RLNGTFGVFMFTHLGMTAPILGTAFFAILHGGSGSSLFICLGWFIGVSMDCFSGQHLQNESIDIARALYDTQWYNCSQDIKRDVLF
VLMRCTKPMYLKATSFGIMDRVMLLGVLKATYSYIALLTQTQ
>McarOR18
MDEIKEEEPFTHSLKMLNVMDAFPLEHNFFSNGIFFVRFWILRTMSFCISCVLPTVHMVTSVKDGIKLIISEDLSVIVGTMVSLITT
CIFVFKRNSWSKLLSDIADLKIYGSFSDFDIVKVKLNLFSRIYFWYCVNATFVYGSVSFIDTSQCEEINKLGKEWREVCGTYLPMR
LPFDANIQWMRVSIFFTQMFFTLLSLAPSALACSIIFQSTGFIIAHIENLKKHLVGAFDSTDVQETSNILRYCISYHNHILRLSARLQD
LVGTNISHVLLMSAVVFAGIGNQILKTKPVGGTLYFIGYMIALFLLCHSGQRLIDETASIGSAAYNSKWYKGNTSMIRDTLLIIYRS
QKPCTLEVLSLGSLNYPLFLLIIKTSYSYLTLLQQTA
>McarOR19
MFKIQKGAPFYSTLLALSLFGQIPVEFKETYSKSFLVKCILSRFIGFVLVSVAPILQYVMATKGSIEVDISENISITISSIGALLTGTFLT
CQYKKWLKFFEDITDHKAFGKPPDYEDLVKNFNRFSAFYTIYCTGSVPVYAVTVYFNSMRCDEAALKAGFFCKSFTPIWLPIDGS
SLQLRVIIYIVQMLLGGTIVCTSAVINFMVWESTEMLISHINSLKIHFNKISEKSTDKERSEQLGFCVRYHNHILRLSSRLNGLIKWT
SGHMSLTAALIFASIGNQISNSKSVGAFLYLIGYVGALFFICHAGQRIKDELMSVGDAVYNADWYATDVKTIRSLRFIIARCQIPFH
YEAIPLGVVDYPLFLMIIKTSYSYVTLLSQTT
>McarOR20
MIKIRDIVCCTVSIKILQVCFLFPLKGKELEPNYLRGFIFFFLMGFSSLTVIGSFLHFIISIKNHVYYHIDLDMAIMISMFTTYSFSIVFF
FNIKSAVRLYMTLSDFDEHGKPRNFDKRTKLIDKVVTYYYIYIEFLIIFMLSTSNVSSSGKCKKKNKKYGLNEVCGLFSYTWMPFE
IDYYPVKQIYTICQLVGTHYLIILAGVVSCLMAETMEQIITRIHHARYLFLEAIKEKDYAKQRQMFNTAVRYHIGVLDLEDPLNET
YGFFMLTHLAMTAPIIGTALYSILYGGSGSSTFICLGWFIGVMKDCCCGQRLQSQSNTVPIAIYDSEWYTCNEEIKKDILFVLMRCR
RPMYPKAISFGVLDHVMFLGVVKAAYSYIALLSQTT
>McarOR21

MSTDRICVPFSTSIAILNFNLLFTHGKSKSMLIEVMGWSLRLLSFINLCIFPIHHLITSIRDGIEVDISEDISSSAGGFIAVILLCLFKFK
EREWSKLINDIVHFSKIQKKSEFEKLTKKMNKISAITYSCFTIALLIYAIVVYNSTGHCRQMNKDMGLHEFCGSFTSIRLPFEGNSLI
IRLPIFLTQMFVNANALMSAAHITFFVYEITQYLVMHVDILKQNLLQVYDVGSSEEISQKFKSCIMYHNRILRLMIRLNQVTKFTV
GELSLTAAIIFACIGNQILKGSSMAGIAYFQGYVTELLFLCHSGQIIMDQTESIGASLYQSKWYEVEAKLMRNVIPVLRRCQKPMTL
QALPQGNFSYALFLMMMNSAYTYFTLLAQTT
>McarOR22
MATRYPADYFGMIKIMYGIAAIWLFNPNGSLIVKCLKYSWTLILYSIVVTFVIFEYIMIEVMFKDIFTLIAQMGLLLCGHISLLKATV
LIKNHKKLAEITDFLEDERYHYKSVGNFDPGKLVVDEKRFTNNFLKMLLVCFGLVGVSPHLAAEKIIHQEVKGNYFSENVTCYD
YLPFIFYIPFPSETKHMCEVAVLFMDMSISGIAIIIACHDGFLVVLLNCVRVQFVIVGEAFSTLRERVLHRLNLPEDFEIFYDEQHPQL
EAELYKELNVVTRHYALLLKISEDLEEIFNKIILVQTLLCLLVFATCIYAGTTVPITSPTFGASVQCFSCVLAELALFCWFGNGVTTS
SEGILLALYKSDWFSASKRFKSSMILTMTRVQKPVYITLGKFGPLTLVSLVSVCKASFSYYTLLKKMNI
>McarOR23
MHNLKQRKSSQSKGNKSTHFHNIRIVLRMFEWSGMNPEHSSTWQSIKFITITICCVLIIYLVILFVLHKENISAAKIADSLESISLIVH
GIVKGVSIFINKKKILGLLDSIKYKFWKIEDIPDEKERKHQRNELRFLKIVFYIYMTGCCLTATSFLGKPLIRKGKHTPFDSYRPDWF
PYHVLFVFEYIVYMYGLYFPIVGVDLFIFTLFFLISFQFQILNYEMQKIFDVEENEEHIIGINENKKINPKIFERRIKRCVEHHKFLQD
FVKHMNDVLSLPLLFYNGIIILFMCIDMYLISIEDSLQDAIKSTIYTVNCILQYTFCFCMPAQAITNEAEKVSTSVYFSKWYEHPSNF
VRIALIMMINSGQTPIIIVSGGFLNINMETCLKTIQTIVSYCMFLRTMEF
>McarOR24
FKTGLRRLEICGANPLQNRKIGYIIINIFSTITLAILIVLKVIYDPDLQATELWISFILMSTKYIILLHKKTALEGLFKNLESFWKIESKD
EKIDEMLSYLKRLATCWNAILYIGMVVYWIKPIFLRGSSVFFCYIPERVPFICVYLLETHFIFYTAHTFIGFNLIITTFIILTVIQFRLINI
RLEQLDMERIQDQSGWESCHNTLRICVKHHNFLIRMVDDLNDTLYAAVAFLIGATTILMCMHMYVFINIDLTFAEIARLLLSFSAM
AFEFMFGYGLPSQMLMDEAGYMTDALYSSCSWYLAPTLIKKEMLLMLMRSQMIICISVKKLVIVNKQTFLMMLKTAYSFYTFLR
TMA
>McarOR25
FALKYSLNMMNLVGLHPEKGNSIIQNIQCIGTIFLNFMVLILILLLLAKHRHTFVMTDITEVFESIFMIVHGSSKLILLYTNRSKLLAI
LEGTKQFWDMEKINDAQVKKSCDRSIKRLLCVYLLFFIFAVFVTVIFVFRPVYQKGSLMFNSYVPKDVPFSVIAILQTYVFIWGIF
LPTLGFDLFFTTIITLIEIQFKMLNLNIQRIYDFNESKKDLSTEKKLKKWVKHHNFMLDYVDLLNKTVSLCMLVYFGIIVLSMCME
LYNMLIPETLSNKLRAALYIIAVFFQFVLCYCIPSQILTNEADNICISIWSSTWYEFSTQMKFSMKQIMMRSQKTMYIMAGNIIYINL
STCLATLKTIVSYYMFLKTMTESSK
>McarOR26
MIEMFKKPLGWLKTCGANPLQKRKIGYMLVNISSTICLAALIVLKMIFGPYLEAAELWITFMLMALKYLVLLQKKEKLQSLFKDF
ESFWVLESKDDKISQMLIYLKRLTLCWTFILYVGLAVYLVKPIFLRDASVFFCYIPQHVPFLCVYLVEVYYLFFVAHSFVGFNLLIT
TFIILTTTQFRQINMRLQRLDMERIQDQSGWESCHNILRICVKHHNFLIRVVDDLNNTLYTAVGLLIGATTVVMCMHMYVLITIDL
TSVEIARFLISFSAMVFEFMFGYGLPSQMLMDEAGYMTEALYNSCSWYLAPTSIKKEMLLMLMRSQRIVCISVKKVVIVNNQTF
LMMLKTAYSFYTFLRTLA
>McarOR27
MLKELEYPLHLLDTCGVHPFHKINIFFLIINVTSIIFITVLLVLKFIFDADLQAVELTNVFLMMSLKYIVVIFKRSEIKSLVKEVDGFW
THQSTDDGISHTFYYLKFITTCWRMLYYFGMFFYIVKPIFLRGSSIFSCYIPQNIPFYVVYAVEVYFIFTGAGAFVGFNLFLITVITLA
VAQFREINMKLEQLDIEGIVDGAGLDKRLSTLKTCVRHHNFLIKVIDNLNEILYIPVGMLIGVTTLLLCMHMYILSSSHVSIMEFIRI
TFTSTAVCFEFMFGYGIPAQMLMNEAEDVTYAIYCRCSWYLATSSVKKILLIMLMRSQMVVCITIKKLIIVNNQTFLMMLKTAYSF
YTFLRTITIV
>McarOR28
MNEGFLKIQRICMILLGKWQFDQKNLMQNKLYKIYGWFIIIYYFAVIQSMPFVIKSRWACEDIITKLGTIYLNHMNVFMTNLMSNT
DHLKKLVSYILNYEKITYPKETDFVRKTYDFYCAMNYKMTALYIVTPSSLAIVYYAFEVTFTFLKDENDPCAPNKGRIYQFWLPF

DTNKYFYVAMPFELLEITIVAILNTYNKLIPSSMSTFQLGQIKILQEMLRHVDDEAIKLHTNQHIEWEQAVDITVTKCAKKLLEILS
LMDILHKATGPLMLLVFSSNALETAIFIIRMLRAKTIVESLISVGVSFIIFVQLLSFFWQANEVYLESQNIIHVIYNETNWVDYNVSV
RRKLIMMMTKCQKPLSFQGSGIGSMTIETYKKLVKSCYSVVTFFTSSYG
>McarOR29
MGRKFLKIHRFYMLILGKWKITPRNTLQNKLYKIYGWFIHIFFIGILQTMPYINRTKINCWDVITKRGALYMICNNMFIISLISNSVN
MQKLVEFILNYEKLLYPSEKNFVQKTYDFYCKMNYNIVMIFIVIPSFFAYVYYGFEVILTFLKDPNDPCFTTKGLIFQFWLPFDTDK
YFYIAILFEFFLMSLAICFNTYNKLIPCSMSTFQLGQIKMLQEMLRHVDEEARELNASQCVEMDEAVDAFVTECIKKLQDILSLMN
LLHKATRPVMLLAFFTNILETAFFMIRMLTAKSDAEAITALGVSSVIFIQILCFFWQANEVQLESQNILDVIYNETNWVDYNISVRK
KLLIMMTMVQKPMSFEALGIGSMTIETFKKILKSCYSIVTFFKTAYN
>McarOR30
MKLHKLLRLQNHLLILLGRFEESFGNIFVDKLYYIYSWFISCYYILMLQSIPFIVLTKWECTELAMKMASIFLHHSNSFFLSRLAIKP
PMKKTLSHILNYENLIYPNEAKDRQNKYNHFANLNFIVSLLAVVLPTQYGWLYYYLEVKRSYIEQENPNCALKKGLAYQLWYPF
NVEKYLYIARIFDFLELLMATIYHSFNKSLPIGMACYQLAQIEILHLMLRNLDTDAKKLQHGNIGRDEAVETLLNECIRRHQIIIDF
MGLANKAMRTIMFIVFFTSSMEMAVFLIQMITAQTTTDRLLTCGVSCVLALQILSFFWFANEVFLQSMKVSEIIYNEMNWVDYTI
GQQKKLVIMMAQSQKSLSFRATMVGAMTLETYKAMIKSCYTLVTYFKTVYE
>McarOR31
MGCRFLKVHRIYMLILGRWKVSSRNTLQNKLYQIYSWFVPIFFIGIIQSMPYIIRTQMNCSYIVTKRASLYMHSTNILIISLLSNNAN
MQKLLKYILDYEKLIYPSEKKFVQKTYDFYCKMNYNIVTVFILIPSIFPYIYFTLEVTLTFLKDPNDPCATTRGLMYEFWLPFDTEK
YFYIAMLVEFYELFLAVCFNTYNKLIPCGMSTFQLAQIKMLQEMLRHVDEEARELNASQCVDMDEAVDRFVTECAKKLQDILSL
MDRLHKATKPVMLLAIFTNSLETAFFIIQMLTAKTTTEAIIAVGLSCVIFSQTFGFFWQANEVHLESQNISDVIYNETNWVDYNVR
VRKKLLIMMTMVQRPMSFEALGIGSMTIETFKKILKSCYSIVTFFKTAYN
>McarOR32
MNDFLKIQRICMLLLGTWKLDPKNVLRNKLIKIYSWFVIIYYIGIIQSMPFVLKSKWTCIDFIKKVGTLYLNHTNVFITSLMSNTDN
LKILVAYIMNYEKLIYPTERYFIQNTYDFYCKINFYITAICIVAPSSFWCCLLCYRSYLHIFGGMKNDPCASMKGRIYEFWLPFDTKT
YFYMTMPFELFEILIAVVLNTNNKLIPSSMSTFQLGQIKILHEMLRQVDDEARELHESQHVEFDEAVDLLVTKCVKKIKNEILSLM
DIMHKATRPLMLLVFSSNSVETALFIIRMLTAETVGEGLVSVTVSFMIFTQLLTFFWQANEVYLESLNIINVIYNETNWVDYNVSV
RRKLIMMMTQCQKPLSFEGLGIGIMTIETYKKLLKTCYSVVTFFETAYD
>McarOR33
MDPVDKMSDYFKHNMISFKYTGIWLNLFDVRRTSLLVIFYSVIINSLFMMSPQVCHVIYMYKARNNVQAFADEFYVSLASLLVV
LKSYSLLKNFDIIKKCWKPWTLIFFKQKRLSAKANTAGYGNMEMIYWLYATFALLYVFLLLISVLLERILQGTKVLPLVVCYPFEV
NVSPVYELMFLYQAIALSWLVIQNFNLDTFITGLLTVAAVQCDLLCNDLENLTPEKLECQKGEENDIMDEKLVDCIKHYQEIRRFV
NDISHCFSMNIFQQFSCSVITICTTLFEFSTKEPLSQEYFAIIIYQSSIFIQLFIFCWTGSELTEKSKRIPISAYASKWEDASKTFKSNLLI
FLHNVQRPLEI
>McarOR34
IVNMIMVLGDIEKMTEASFLALTHLVQVMKLFYVLRYENKLKLLINSINRKSFQPKNLEQYVILQKYVRESNIISKTFLSAGFVTC
CFWGVSPLTQSGDIVLPLAGWYPFDTDRSPAFEIIFAYQFVASVTNALSNISLDTLMSGLIMVVCAQLNILNDSLRNIRKYAEAESD
DGRAVSREELQRRMDERLVECVVHHKHILEFSNEVTFLFTNSILGQFIVSVVIICITLFEITLLPWGSLKFFSLILYQFCMLLEIFLLC
YYGNEVILQSMQLTKFAYFSDWTDCSTKFKRNLLFFMTRSQVPLRIYAGGFFTLSLETFVKILKSSWSYF
>McarOR35
EHYWVAYVWLVSSGGVAALNVAFIDTFMFNLIIFPLGQIDILMHHIRNFNKYALEVQNRVGCSNEEASFLLMRDFILKHKEIIRYID
DYNIEQRYIMVFEFILSSIQLASIAVQLVLSHLGFFDIVHWGGFALCMLLRLLVYYWYANEIMIKSSNLGNAIWESDWLEKSYKVK
QLMLIFIARTQKPLGLEIGPFTTMTLERFLGIVKATYSYVMIMYR
>McarOR37
PIPGWLPFDINVDFYYYPTHVLQVTTILISSSINTSLDIINYVLITIVCCQFDIIVDELRRIDLKISSGEMQLKMVIKRHKEILKFSKNI

NTMYSNIVFIQCTSSVLIICLLGLQFLMVPLGSTKFVSSASFLFTMFLQIANYCWFGHTVIIRSTEVGDACYDTNWNESNTKIQKLL
FIIMERAKMPVTFTAGGFFTLSLTTLTKIMKSSYSYLAVLQQLYET
>McarOR38
NPDTPYRTKLAQHSYNIFWTIYLCFIYLPTEFLALFRAGNNFTNLIRSFRDLGNHIALIIKAVNWYVKRRRILTLLNVLEHTAIRSEN
VEYQTEFIIQEHGRRAQKWSSILFRLINTICGCMLFRGYSFLYFYGEDSYVWENGRRWYRQKLPVEVFPLFGNRSRIQFLVTFIYEL
VALTFYGWMITGLDSVFITILSCMSAQLEITKNALRTIRSRCCRKLGIIPKNDLYDPLVLQMEMQKELRIWLIHLQEIYRCAEEVEEI
YSIQNLLQIFISLYEICFCMYLLSISSYESMGSELIYLFSTSFQLLLYCWFGNILTDASTNVSYALYDSQWFATSISFRKEIIFTMTRLN
KPIYVTVGKITPLSFTTFITIARGAYSYFTFL
>McarOR40
IHTVTAGEYNHIDLDLSYVLSMTSGFLLFTLFSFNITTATKMYMFLSEFKEFGKPPKFDKYNAFLNKVAKFHVIYLNINITLFAAGS
NVFKGAQCKKDNIELGYKEICGLVTNTYLPFDIDYFPLKQIYVGLQFFSIYYVYTISGTITFMVMETMMHIGFRLDHVKQLFDEAI
SEKNVERSRKRFNFAARYHARVLELEHEVNACFSYAMFSHMILTAAIIGCAAFGVMQSGSANPFAVCIGWFNGISFVCLSDQHLIN
KSLEVGTAVYSSKWHQAHPSLQRDLVIVIMRCQKAMILRSAGFGVMNRATILAAVQASYSYITLLSRSP
>McarOR42
KAINGRFILNLAILIIVTILVVFPHYCEFSHVATRSFHIYLLRMDKIRWHPNTEKAPSEKMLETIKLFKPNDFLSKKNTIYRKYVSWL
KGYAAYLVVCLFLVMGNSLATDTATVFETYRPKIVPRAVLYIAELHVLLLSYFSTVGASVLASTLLLTVAQQLNMLNMKIQTYDL
KELETEEGSRKFMKEFKKDIAYHIFLLKCITQLNEIFSLPLLMQLLGDVYVLCINMYGFSSPKSTTVDLFRHGIISVEVIVAFVFLFG
YPSQLIMETAEGVGESVYSHCIWYLPNIIPVRKDLLLILVRSQKTNTISAGGFTDVNNRTLLFLLKTAYSFYAYLQTVS
>McarOR43
YLRHLTFLNHFIIFCFINLLVMLLRPFIFGNPSIFTCYYPKQIPFLVTYVIEAYTLITIVSSYVGTSIFICTLILQVVMQFQLLGDKIRAV
DLTRVESGQDTLPCLKELEKHVFYHNYLLNYVEELKKSLSLSFFIQIAVDVLMMCSNMYIVSSKATSLLDTGRMILVCMVLIFEN
YFFYGSPAQLLTDQAENLSDIIYCDTDWYQPNIIQARKYLLLMMVRSQKPVCISAEGFLDINKRTALFLAKTAYSFYTFLQMLEAK
RE
>McarOR44
MQLIYPKEHECTIKHDPIWQVKFFLRLLSFNGIKKIFWFCAIVLKILTLYAETTFAFQNIQEPRKLIIVLATYPVRIMAVWKLIHMYV
DKERVDRFYRTIEKDFWEFHIAGPTLERKIRKRFLATNIFVFSNIIFNVICVTLFALGNIRLTPEGKRPLPNHNIWAPFNMDSSPVYE
VLYVFLLWNLYLTALGNAFYDMVFVYCVQHLIVQFIMLKELLKNISLGIMDHKSDVEMFNSEYFQKIVIERLKICTKHHNKLLIY
GKNIEHFCKLVLVPQLIMSFAILVINGYNVTVESDDFSTTLMLTITCFIQLAVYALQATELREESLSILNSIIECKWYLFKSPLKKTLV
FILMNAEEGIVIDAAGMTNVDNPLLVDIIQKVFSSITLLQAIINED
>McarOR45
LKCLEICGASPLKDRKIRYILVHYFSTICVGTLIVLKVIFEPDLQAAELWITFILMSIKYLILIQKKTALESLFKDLESFWKLEYKDEK
IAQMLSYLKRLAKCWHVILYIGLIAYWIKPIFLKGSSIFFCYIPEHVPFFCVYVLEIYLFISGHAFIGFNLMITSFIILTAIQFRQINMTL
ERLDIEGIKDQRGWESCHNTLRMCVKHHNFLIRVVEDLNNSLYVIVALLIGTTTLLMCMHMLCFVKHRFNYSRNRTAFVKFFVR
WCFEFMFGYGLPSQMLMDEAGYMADTLYNSCSWYLAPTSIKKEILLMLTRSQMIVCISVKKVVIVNNQTFLMMLKTAYSFYTC
LRTLE
>McarOR48
MSKRIMLLLGLWPATSSHDWKYICYKAYFCAVRFNYGVFIITLIMGIFSKYTLNAGPLEKADIIQNYLLYILLLWKMILITTSQILKL
IECISEREMHIMNLEEEDIKNIYAKNVNYNFKIFLIVTATIVVGLTQFCILVSFEIHGLKNNIERKLPLPEWFPFNVGKHFLTSYYFQV
FNRIYSGMIIVAMDSMYFSLIYFPITRLKILGHNLMFFQELCNKNYSETPDNILKALILEHQDIMRYVKDFNRLMKWYLFMDFLVR
SYHISLVLFKIMALQEFYGNGSDGVLLLTFSINYLMVLLLQMHIFYYHSNELRLESMEISSYIFQGNWYDQSPSCKKSFLIMMMR
AQKPLEIHIGNLNTITSYLIVKVLKAGYTYVILSRK
>McarOR49
ENIRLSLRILNSLGLHPETKIFRPVAILNLTLFACIGMVVTANIAFNFSLEQLQVTCYILNTWSKIGILIIKRRGLIDLLKSREKFSNIDF
NNPNYSIYLTYFKFLKRYAIWAIFHVLVIVSKPFVLDDPSLFNCSYPKNIPFLVTYLFEFHGTVFIFTTFVGSSILICNMIVQVVAQLQ

LLREEIQKIYLTQVESKEDLLLCLGILRKRVVHHEFLLSYVEQLKQTLSMSFFCQLLINVLLLCINMYVLSSEEATFMDKTNVCLVS
MACIFEKLLLYGFPSQLLMDEAEELCNTVYCDTDWYLPNIIKYLLLMMVRSQKSVCISAEGFLDINNQTILFLLKTGYSFYTYLQ
MLTTK
>McarOR52
MRYEFLKIQRICMLILGKWELNSKNELINKVYKIYGWFVPIYFIGIIQSIPFILRTKLDCTDIITKIASLYMISTNVFVISIVSNSANMR
KLVSYILHYEKFIYPTEGFLYKKTYDFYSTMNFNIITLFIVCPSSFGIVYYGYETSVTFWRDENDPCAPKKGLLFQFWLPFDTEKYF
YIAMMFELFEVCLLVIINAYNKLIPNSMSTFQLGQIKILQEMIKHVDEEARELHVSEYFEMDKAVDILITKCAKKLQEIISLMQILH
QATRPVMFLAFFTNILETATFIIRMMTAKSAGEGIIAVGVSCIIFIQIVSFYWQANEVPLETQNVINVIYNEINWVDYNVRSRKKLIL
MMTMCQKPLYFEALGIGRLTIETFKKIVKSCYSIVTFFKAAYN
>McarOR54
MVLFPKNDHFKVTMYANALLGVWPFIFEHNPFMRKLYHIYANFTFIYFMLFIVTAYMELVVLLMAKELRVQEIVGNLCITLLYSIT
IARVYAIKSDSVKNLIREVIEVEEVIYKSDDEEVIGIYKEYTHHSHISNIIFLVNITIETIFYFTHPLYVGETIVIDEATNATKVVRALPL
SSWFPFDEQEYYHLTYGWQMADGTVGASYVMYTDIFTFSLIIFPLGQIRILMNILRNFDKYVKMTQDQYGYERDEASFLTARECI
LKHKNIIRYINEYNRVMRNIMVFDFLQSSLQLASIVIQLFVSEVRLFNVIFHGEFALCMLIRLLVYYWYANEIMVQSSNVALAIWD
GGWYEEPQKVKHMMMMMIMRSNKPLVLDIGPFSPMTLSALLGIMKATYSYMMIIYN
>McarOR55
KYIRFSLRLVRLVGLHPETPILHPLSIINLTILLVFVVVMGVNMVISFELEQSEILCFHYSILGLKFYILKIQRDRVINLLQSSSSFLTVN
FQNPSYFLYLEHLKFFNHFTAYCVINLLGILMRPFMFGFSSMYNCHYPERVPFLVTYLMESYMLIAIVCTYVGTSILMCTLMLQVV
MQFQLLREKIQAVDLTMVESGQDSLSCLRVLEKHVFYHNFLLGYVEELKKTLSLSFFIQIAVDVLIMCLYMYIISSNARSLLDMGR
MSLVCVILIFE
>McarOR56
SPHVTTVMSLQPRSKGIFKNLTCYDFMPYTFLYPLFPSQTKRRCEIVALYMDIAISGVALGIACYDTFFAAILNCLTAQLTVVNKAF
RTIRQRVLQKLKVASNIKLFYDDENPRLEKELYAELTFTTRHFVALLQIAEDLGKIYNLVILGQTVECLLIFAACVYIATTVPITSPEF
AAASQYFAAVFVQLTLFCWFGNGVTTASEDILLALYECEWYSASRRFKSSLLITMIRMQRPVYLTLGKFGNLTLVSLVSVCQGSFS
YYALFKKY
>AcorORco
MMQFKPQGLVADLIPNIKLMQFSGHFMLNYYAETTGAVHTLRLGFCFGHLFLLLLQFGFTFGNLVQQSDDVNDLAANTITVLFFT
HCITKFVYFAVRQKLFYRTLGIWNQSNSHPLFLESNNRYHQLALTKMRRLLIVIMIGTIGSWIAWTTITFFGDSVHTRKDPNNENE
TITEEVPRLLVRSWYPWDAMSGAAYYVSLVYQIYYVGFSMLHSNLLDSLFCSWLIFACEQLQHLKEIMKPLMELSATLDTYVPK
SADLFRAPSASSQDNLVDSDYNQSNEDANLRNLYTTHQEMGVTYRSGNLQEFSSGGIGPNGLSKKQELMVRSAIKYWVERHKH
VVRLVTAIGDAYGIALLLHMLTSTIMLTLLAYQATKIDGVNKYALTVIGYLLYALAQVFHFCIFGNRLIEESSSVMEAAYSCHWYD
GSEEAKTFVQIVCQQCQKAMSISGAKFFTISLDLFASVLGATVTYFMVLVQLK
>AcorOR1
MSWGYKIGSFLNSKILKNEDEVFQDDAKYITMFSQVFAKIINLWPGDDGVSKKFTFGLMLTSVITQELSLVMYLLTTKINVDVVIT
SMASMIILLQSIVKSCVYFYNARKLKKLIQTVRKEFWPANIMGETTHNDIKYNSKILSLVFVVQYASAILFLWFSVLLPLAKPGRK
LPHTSWFPFDSTVSPLYEIIYVWEVYLTAYINANIVCSYDTLFCSICGNCISQFRLLSAAVKCIGISGKENQISNRLLRLQGVDYNPV
RTNQGKEDRESRRLLVICVNHHQKLIKITQELNEVFGPGHLAQFFASALGTCTACYKIPIEKNPGDLFVLIAFYIAHVSQLLEYCAL
SHELSYWGIKLGDAIFESFWYIKKHSQIRKCLPIIILRCQRAISMNALGIFELNYPSFLIIMRFTFSLYTFFNNMSKSTI
>AcorOR2
MVSKSYFKVQIFCFKMMGILMEDIDWNRFTSRAYAIYSFLLLSCFIYLFIITEAIDLILKWGDLDNMTFNLCYLVTHFAGLCKIAVI
MHQKSKIRTFYQSLESGYFLPNHERGGNEEFRIISSAIWQSNMQTYVFYTFVTVIVANRGFYAGFDKGYFIQFTSVNGSETTNKHY
KVMPYTTWIPFDTNVSPYYEIAFAYQIVSALIYGLLIGTCDSFIAGFMVHIKAQLLILKNSFGNYIYAAKVKTQVKNDFENLSFLK
NCSDNGLKTNKDIKNLNPETLIYVQRYLRDCIIHHQQTIKLVEIVESEFNYLMLIQFLGSLLLLCLSLFQLSINDIRSTRFFSMICFAG
LMLFQLLIFCWNGNEVLVESLEIAFAAYGSDWFLCDLATQKALVLVIQKAQRALQLSAGKFAYLTLETYMNILRASGSYYMVLR

KVNE

>AcorOR3

MDDENSNGNPDVGADFFKPFFSILKVILYWPLYTSKSQLLQHVHMLLSAVKVTFTVVLYIFLECFTVYKRSNLQERLAGSFMMFTDVSYLTKVLFFIINRKKILLVFKAVSDDVFSPKDTLRKEMITTTMTNWKKVFYIYITNCCFTVTLWAIIPSLQNGSIVLPYNYHYPFDVTSSPAREFAYIYEAGILYLIVVSHVSLDALMVGIMAFISAQLDVLNYNLRGLNNLDEVNSYSKHQFLKEEQIRCMIFHKKVMRLVKMLNQILAVPLVTQSCFGAIILCLSLYKLSTLNPLSGEGLSFINYYFGMLIQLSVYFWYGNEIIWKSNELSRSAYQCKWVNTSKPFRRHLLFFMLSTREPLKIYGGRVLELSLQPLISILKFSYSCYTLLKSV

>AcorOR4

MEKETLQYNAFDLERRILWFYGIYFSKDFRPQKLHYLRVIATSFVINTLVLGIVMEMIVDHNNLETVFQSIYYIIVVIIGQIKTFSLYRSLSQFNSLEDMLQDVIFNAEISTGCTSISKAVDTYKIFKRIFWGTASFCASVYSLMPLLSGDLSIPIWYPSESFKLYCQLFEIVCIWTIAASLLSIDVIIMGLIYFMSAQINTLNYNLRNATDRNPDYDAGTQEKQVQDNLRICIRHHLAISEFVSKLEEIFKFGLLLQIFSSIIAISAGGVYMVFVTLTPSSFIILVSSMTMILAQIAMYCWVGQGLLTESDQIGESCYMSEWYTCNIATRKMFFIIMERSKRVISFKAGNFFELSFATLVMIIKNAYSYFTVIITAFK

>AcorOR5

MVEMRFFDLNVRILKLSGLWVPNFTNKWKYRRTIAYNSICILYSMIYFTIAELISFKESAANLNDLVKNLNMLMSFLLTLIKVIVWFRYRKDILKIIRFLETPRNVFKDYNLNNEEIILKELEFKDTWTKSFFIMSTLVPLSAGILSITETLTTGEKYVVFRNDSSLIYIQKLPYYSWIPFDHTSSKCAFRIAVVSQCIALLNCGYITVGLDMLFVALASSITAHFMLTKEAFRSIGNFGSNEFDNAQNDNYIKFKNCMMHLQTLIKICQRLEVIYSFLILMQVLVSLVVLCTCLYLVSSIPVGVRLLGNELAYLLAIEIQVAVYCFVGNKLTDAALQIPLAIYECNWLNTTSNFKKAVIITTIRMQKPIYITIGKFSPLTLNTFVMIGKTSYSIFTVLKSRN

>AcorOR6

MKRDITQYNALNFERKILWFYGFYSGKDYLARKHRNIPLIISSCFSFTFITGMVLKVLEYQDDLETIFETAHACITSITGAIKLFCLYRALPHFNILEESLIDPIFNLEIFEGSNFISKAVKEYVSFSKFYWTMVFITFVLYGVFPVISGEIPIPIWYPADCPKFYVQIFEVISILILSCSYPGIELVLFGLFYLMSAQLDTLNYNLTNSTQWNGEDNREVQEEKIQDRLKCCIEHHLAIIRFINGLKDIFSFGIFAQIVLNVLIICTSALQFLRNSVTIVTLLSSIMYTLTILTQIGMLCWVGQNITTKSSLIGESCYMSDWYTYSVSTRKMFFIIMEKSKLVISFKVGNLFEISFKTFIMIIRSAYSFFAIVITMYK

>AcorOR7

MSLRDEKDPEILSDYFWFHKLVLKICGVSFKEQENLIYKIYSRFVQCLIGFLIFGEIYTIILSRNDLQLMAEIISVSTSHFLGLFKLSVLWKNRASIADALNALHTGVFLPNSRRSGLSEKILIKNCITKVYMLIAFHSATLVATVFNIIGSSLVTKFKFDDYELWKMPWIPFRLFPITTTIVYYTIYVYQTVTLILFACIIITTDLMVVAVLVHITTQFHILASVMRTLVENNSVNYISEQYDLYLRKKLKYAAYYHQELIKLTDRFEELFNMLVLAIFMGNCIVLCFGMYLMSSGELELNQLISEFTYLITVVMQIFLYCYYGNMITEASDAISFACYETDFVGTDLRFQKGLLLIMMRSQRPIVLTAGKFAQISLAAFVAILRASYSYFMVIKSSSVEA

>AcorOR8

MEEEYVMFSGLYSLNLVGLHPFKSSFKKGVVALIFIVFTLICYALNLAGVILKYDGLKSLADSIDAVPAGQQVMVKLLSVLFLRNEMKHLYNTVEKKWDNKIYGEELENTIKKLSLKFKKIYNTYRMTIWVTAILYVSKPLILFSRTLLTEMYIPCNLSQNYCYISFIAIQTIYIFDLAFVVYTFDGIFYAFLFYVYCELEKIKYGFANLNVSITTDLNNNEEDCYTKFCEIVKHHYSMIKFLQDVNKVYYLQLLNHFVTITATIVFGIFFMNMDGFPPALGKVSRYIPYLMSYHFQLYIYCMWGQQVFDQVCSVCDVIYQSQWYVRYQPKLAKGMLLVMKVSQIQNKLTIGDMWKLNLGTFMSVIKTSMSFHAFMQTVYKSDEVLVESYNQTIF

>AcorOR9

MTDRPKLLDFTKLPRRVLWCFGFYFGDDMEEHIFHRLICIISLTLTIPFPVMIMGKILLDLRHDLQALLETLHYFFLHFWIVVKIMLFLYGFRRLRNLEAWLQTEIFNSYTRRQDHFLNKAMRKQMSFFKLLWSSTLCFTSMFALFLPKDKVSLNIPIWMPFKMDRIFWHIYEVLCYVITASTYPAIDCIITGLVANMTAQLQILGDNLERIHLDHEGGLLKCEDKIQERFKRFIQHHIAILQFINETEAVFSYSLFCQILFSVLGICLSGFQFLLVPPGNTKFMLACGYLTVMFFQIYFTCWFVEDLIIQGSDVVTSCYASEWYNYGSTTKKLLFILMERAKKPISFRAGYFFTLSLATFVMILRNSYSYFAILRHVYKE

>AcorOR10

MDSYIPNFFKVNVTFLKHGAVWSPKDKTQRSYKVYKMYQVIVLFLTLGCSSYSVVMGIIIHLKNFAILIEVLSVSFTILLAAVKTSF
WLIKGEKIKIIMNRLETDLFHCEKIDDFDPEGMLNQAKLSGIKYACLLLFFSHLVLGLGYIPVMSLACWYYFKDLQITDVPTFKTL
PYYTHIPFDHDTPLKYIFACLLQCVPMYLYVNAFVGVDSLFMNLMNFIATRMLILQGAFRTMRKRCLQKIIGYDLAPDSLHNSNE
MEEYMMSDMKKCIQHLQLLLRSCKDIEDNFQYVSLVQALGTIYILCSTLLLLSTSPPFTKEFGRNIFYLLGVIVQLGLYCWFGNQL
TLKAANVPIAVWESQWLETRKPFKICMLLTMMRMKRPLLINAGKFVPLILDTQIAVLKGSYSYYTVLKGMSK

>AcorOR11

MAFYKISIKTSLTMLNLKGLNPLISTRSSNTRAITFLLTEVLATITVASSLFTKTLEADSVVDNISGIVFSIQTICKEVTMLLCRDEFV
ALLNYVEEFWPVNEFGIESGTNIRNIQKSTSKALKIFRCLLLMCTVIIISEPFFAEGRQFPVSWIDISCIQTSLICYGVIYGFLCGCTIG
LVIFLSLIDGLFFNLLSYGYCELEQVKYALFNLSIDGDVRGDQVETLREIAVLVRHHVTSLEYVQRVKKLMSKVMLYQFSSSLFTL
CTGLYVLTYQGFPPSVEAAVKFVPFVACAAFQIFAYCVAGQKISEQTESIANAAYECRWWFKHQPRLQRSICLIIQRSHRRIVLSAG
GLWNLDMDTFIRILKASFSLLTFMQTMYIPE

>AcorOR12

MNSTSIYKNYFKVHFFVLTCLGIELKPMKGSIANFIYKIYAVMIFTVVYLYFPYSEILSLVYEENFESAIYNLAFLLTQILGLIKISLILI
YKKKIRMFCKFIETPPFLPDQNRSGEAEFLYVKEAIRACNTQGYIFYGLTAGIISQMMYDALSNPGYTKCFVDSATNITITKHIRAL
PFNSKLPFETIDSPYYEIACIYGSLSGAIFGYSVGAMDAIICGIMCHIRAQLLILQECLKTFIPRGIYQMRENVKLTNNDQKLLQSIT
NNLNETIEIPNTLQKYVHIAVCNIITHHQKIIKLAQDAEELFSPLMLVQFLFSLGILCFQLFQLSITDIESVHFFGMSSYLILMLFQIYL
FCYRGNEIMLHSHNITDAVFESLWFLTDLKTQKLLLIMMIRACRPIKMTAGKFVFLSLEAFVSIVRGSGSYFMVLKNTNAPATEL

>AcorOR13

MASNKTRTLCKNYFRIHYFVLMLLGVSIQPMEKNIFSYLYKFYSIVMFTIVYIYFPLAEILYLVYNTDLENITSGTTYICTHTLGTLK
IILILIFRKRITAFCELIETKPFLPDPHRSGDIEFDYVQEAINACNYQGYTFHIFVVAIVLPKIFYSLRDSEYETVFNDFQNVTFLVQRQ
RAGPFNCVMPFNTINSPYFEITAIYQASCAAILGCVIGSIDAIICGIMCHVKAQILILKKSLGTYIQQGLFLMEEDNIDGKNVIGVDE
FEMIRNSKTSIQLENVPISLQKYVDISVTQIIIHHQKVIKLSQDAEETFSLLMLVQFLFSLSIICCQLFQLSILKMGSAQFYSMCFYAM
LMLFQIFLFCYRGNEVIVHSYDLIDAIFQSNWTELNLKTQKSLLLMMTRACRPIRMTAGKFVFLSLEAFMSIVRGSGSYFMVLRSI
NMPEE

>AcorOR14

MDNFFDVNFTMLRMSGIWIPDTSSQPIIKLLYLLYNTLWICYSCLFFCPSELVYFANTVTYYVPDLVKNVNMGMTHFLANIKVCLW
FYHRKEIMAIIETLGIYGRRYESYGDFDTDKIVQNAKRFKDIFSVLFLNFAMFTSISSCLICFLNVITAEIPPGEEIDMKLPYFSYVPF
NYKASKVAFSIAIWYQFFPVFNYAYIIVGFDTLYTAILGYVSAQLDIIQGAFETIRPRCMVRLGLKLSQNILRDPPTLMDEMHKEM
NKVVNHLQVLLDICRRLEEIFTNVILAQVMISLIVFCTCIFLVSNLPMMSLNFAAEMIYMIAIECQLLIYCVFGNKVTVSSGNISSSI
YNGDWYSTSTSFKRSMLITMSRMQKPIYFTIGKFTPLTLSTFLTISRASYSFFAVLKNSDFSN

>AcorOR15

MDYNYKNIFNLNLTILRVLGYFPSNTGSKPFNAMYKSCTCIAYFLALLFIASQAVEMVLMAKAQNLEKLSTTCLKLFLNVSYFVK
LTFFINNNNRVKLLIRKIEHKLVSPSSPQQDESMTKHIKCMTTFSRTFLYMSVITCVLFAIFPLIDKNEELEEINTDGWYPFKSSNTV
TLVAYVYLSLEELLAGLCNVSMDCIVIGCLSYICMQVRFLKHNLKHMKDICTNTLAKIPDQSLLNKNDYRRQLQEHMDDTLINCI
LQYQTIVRIKRDIEEIFGMGIFIMFMFDCLALCMTMFQLLIISFKSIQFFCVIIYMMCITMELMAYCWFGNELLVISSQVPVAAYESD
WIDTPVYFQKNLLMFITIAMKPMKVTVIHFSLSVETFTVIMRTAWSYFAVLRQKYNEEH

>AcorOR16

MTRREMNYPKNYFHKPLRNLALCGLWLYEPKKMNYKILHYLWFIILATCACFYLLTEYTHIIKHLHEMQEVTLALCYIFCHSMIF
GKIVIFIIKKGKISKMVKLLESGPFLPNVARGGPEEIDIIRRTIHLTNVQLKIFGAVIVVMMTTGVLPYLKNGRTFNEISPNITQVIVKF
PYPSTLPFEVDYTASPCYELMFTFQVFSMNLYGWYFSNIDALIIGLMMHIIAQFKILVSAIENVTKRAENMAAHDKSSALSQVIKKI
TFIKYNIDCDNFIIIERVETYSETIMANLKKCINEYAYYHQEVINLVDDMEKSLNFLFLIQFIGCLLTIVVGLYQISLVPFGSSSFTNM
ASFSFAITFEVFMHCIYGDEISFYSAEVGKAAYNCEWIKADDRIRKNLLLMTLRCQRKCFLTFGKFSKINLVLFLSIMRGAFSYFTF
LQKMNEELNM

>AcorOR17

MSEVSFKSQKVAQYDTIDVSRKILWAFGVYTGKKYPNRILCKISLAINCILTFTFMISMLINILLNMDDLETVFVITHLLVTELGYTTKTYYFMRMLKEFNALEELLEEPIFNDHSLEQDNFVAREIRTSKILSNIFRCLSWCAQLTYTICPILDGDIWAIPIWVPLTDGSPKLIYQAYEALCFMSLASVEPALDLIPVGFISTMAAQLDILNDNLKHSADKNEDELEEGKIRKRLAKCVKHHLAILSFLKKLEEIFSFGIFIQIFTSVGAICMSGLQFLVVPVRSATFVAVFIYFWVMVVQIGTCCWVGQTLITKSNQIRDACYESTWYNCNTSTKRIFFIIMERSKKTISFRAGNFFNISLATFVMIIRNSYSYFAVLMQMYK

>AcorOR18

MDLLEKYSLRIMQLRSLNARESSIVNRAKAIGWVFVDLTFLGSTLYYLLFHVTDIIEAVDCITVIIVVCQTLMKQLSLLIYQQEYADILNTVDKFWAYDKFGAAPNKKLTSIQNLIEKLVQCHMVVIIACGFFYYFKAALQREKVLIMGWVTVCGIENNMCYAVNYAGQVMWIAWLMPIFLGYDTMSLLLLGRVYCELEQIKYGFINLEVQGEESEVLKQVSALVRQHNLVLDFLEKIGGLFSSILLCLFLTVLMALCTSFFLLTATGFPPSFSVLSRLGPYLAGSCGQNLLYCIVGQIISDQTLSVADAAYDSKWFATKSLGLRKAICLVIQRSQRSTQLAAGGIFNLNLETFVAVTKASASALAFLNTMYN

>AcorOR19

MRSEFSTYTNFLKVLLYWPIESKNVYIVIAYDIMSSIKFILVYIVFFIMDCIVIYENSANVEQMVSESFLVFTLCNFFVKSFQYTNCNRRIRGVLEDISLDIFEPKDKQSEDVTKKAMAYSKRIFYIYVITCCSFGFLLGAGAIISEEHNALPFNFSYPFDKTHGFGYKVALAYEALSISSNAMTHATMDCVVYSILAFVRVQLSLLNEDLCNLGSIQYNTYQASLEQQILCIEKHQAIKRIVKELNEIITFPMFVQSTLSAITLCMSVYKLTGVELLSEEGISYIMYCAVMHAQLGIPFWYGNEIISKSNELTISAYQCNWIEENKHFKSNLLIFLMSTIKPIEVSGGYFIVMSLEPLITILKCSWSYTTILRSI

>AcorOR20

MLWVFGLHLLNFKETGFRLILFKIRTLITIVSSTTMLLFLLVKIIQSKNDLMSAFETCYYSLIQAAFVIKLYIYLHYLPILIELENKLESNIFNGHKQDQLHFISDAIKSHQTYLGFYKICCVSTAIFYSIFPALDGQQLAVPIYSPLDLKKYRLIVYLYEACNFFITACNNTAFDGTVIALITIMAAQIDVLKDNLIRATLRDQTVDAKDQERCIHKRLKHCVIHHDAILDFTKTTQKIFSNGVFVQILVSVLGICMTGIVFLTVPLKSMKFISMVLFLITQVVQIGMFCWFGENIRAKSSEIAQSCYMAHEWYSNNMSNKKILFIIMERAKVPIIFKANGVFVLTLNTFVMVLRSGYSYFTVLRHISQNMDLS

>AcorOR21

MDTLEKYALRVLEARGLNPVKSSIMSKVNAIFWTIVDGTFVITILMELVSNTSDIFTFVDNFSAITVASQVVTKEIGLLTHQQEFRTVITCLKEFWPKDKFGKQVKAKLDNIESFSKRFLQIYICSVACAVSLYMLKPILEGNKILPIMWVTFCSLEESLYCYIFNYILQVVWAACGLHMLVGFDCLFILLLLCGYCELEQIKHALISLDPDETTDEDDAPLLDLIASLIEQHNRVLNLLKRIQDLLGSLLLLQFVATLLSLCASLFVLTSVDFPPSLPVVSKSLPYIFSVFTQNLIYCVAGQVISDQTLSVADAAYASKWWIKAQPQLRRMILLMILRSQRPEEMTAGGVFALNLETFVAIMKTTGSALAFMNTVYGEEE

>AcorOR22

MPVPKPLIRKLSAEFSKSMYKDDVKRCILPGKILLQGVCCWPDDETLFYKSLGWFLFWNLIIVEIFHAAYVVKNYKDIEDAVTAGATVTTTMEGIVRLHTILTKRNIINSILVKVWKRFWPLDVVDPIKRIQLRKRAQLALVLTSIFLASSIISNSQMVAVPYIKNRTMLLKSTFPFDWDQLYYYEIVYVWHYFSDWFVLFMINSFDFFFVALVTICSIQFAIMQEVFKLILSKQSLRHRAVIFGQRGKTMDDKEMLLKCLEQHQLLIGICNDLEKSFNITILIQFFVSTSAICAASLLLKVDYSQFLKMLMYAAAHLSQLFYFCFVGHELSYESGQLSDAIYECNWHLSYDRDFRKALILIIQRSHRVQWLTAAGMVKLDFATFLKIMRLSFSFYTLLHDMLMKNLDLN

>AcorOR23

MEKETLQYNAFHLERRILWFCGIYSGKDFQSQKRHFLRAMGVFLVLLIFDLSMLMKIIVDHNKLEIVFQTAYHIIVIIIDQIKTVSLYRALSRFNNLEDMLRDPIFNTETSTRCTFIAKAVNNLINLKRVVWRMTIFIATLYSLMALINGNLPIPLWCPSIVLDMFNPYYQLYEVLCIWVTTATISTDLILIGLLYLMSAQIKTLNFNLRNVTDTNPDYDAERQEKQVQDNLRISIRHHLAISDFVSKLEEIFSFGLFLQIFSSIIAISAGGVYMVFVTLTPSSFIILVSSMTMILAQIAMYCWVGQGLLTESDQIGESCYMSEWYTCNIATRKMFFIIMERSKRVISFKAGKFFELSFTTLVMIIKNAYSYFTVIITAFK

>AcorOR24

MLEVLYTLGEYTGVFPQKNKTDDYKNVLNILVVFYAAIGVFWFGVNVLMNSKATLLDMVDAIYTMESECSLLYYYMISIHTRKPALKLYNDVTDFTTFGKPKTLEREETRICKIYKIIVGYGIVAPTVTNGFYLATYDWCMRSHRNDDDIQYCGYTFGIYYPYNFEKGV

SFIIHTAINWYSFVFLSLVALTLVGYTICVARYLVLKIDHLNTMLSEVLKNDAVNRRELLKKCIRYHKHIISLVDGLNELHSMNNAP
AIFLYSTIIGVCLFYLTNEYNTKAIALACGYIGGIFCLNFAGQMILEKSETVGVAAYNMEWYNADSSTAKDIMFIIIRSQVPLKYKA
GPFGTMSLIFFGSILRGAYTYMTMQTDIKEK
>AcorOR25
MEEELPHKIRRLSKDFNRDISNDGAKRIMIPGKIFLECLLVWPDREMKYITVFNWFMFINVIIFEITHACFVVPNIADYTTVIAVLVT
VTATFQFLVKFYVIVFKKSIINQILLNIWREYWPLSVLSPKKVKRHSSTCKVKLRLILGCYTLAVIFAAIITFAPFLTNTELIVKSIFPF
QWNKTYTYELVYTWQFVTAWYITFLINSFDMLMISVVIISAVQFAVLQNVVKNILTEKGERQRRYLYNKDISNQDMFKRWLEQQ
RMLIDTCNKLEEAFRIPILHQLFCSITGLCASSVILKVDQSKFLEMSTIALANMFQLFYYCFASNELTLQSEKTSDAVYFCNWQISQ
DMKFKKALVLVLQRCQKPLSLTAAGFIDLNFLSYIAVLRLCFSFYTLLTDLIVSKLEAAELQ
>AcorOR26
MASQQELSFLNRFKIFQINREIIMDGAKYLLIPGLVSAKIIMAWPEDVRSKSFEILVFVMSFIQCVTIITSIVLNIVDVNTTIMMISAFE
AVLQVVGKFSALIIKSKDLDKLIKTVRYEFWPSDITNKDTAEKIRKDSRILFKIMMIESSICIMVVLTIAFGPLLKTGRVLPYPTWYP
FDTSASPVYEIVYILQSYFGLHLSVPPIIAYDMLYYSLCANCTAQFRLLCDALRCIGNGTEDEMITKLVEFDESQKELRGTSEQKLL
ILCIKHHQRLINTANEISQAFGNGHLVQLMGSASGICTACYILTSNPDLSSVANALVQYIAHVGQIFIYCAVSNELSYWSTLVPTAAY
ESLWYKKKYPNIRQCLAILITRSQIAISMQAFGLFELNYTSFLSIMRFTFSLYTFLSSFA
>AcorOR27
PEDTSFNIYHGLILAACFLNIVSTTISVIVNTEDTDALVLKLFSIGALAGISVKYAALLYKSKDFTKLITIIRVEFWSSDILDLDCDRSI
YKDTKLLLVIIVTEYLAGCTCSSLMAIIPYIKSSKELPYSLWLPFDWTRSPYYEILYLLQGYIAVFLMNAVFGYDSLYYTMCANCTA
QFKLLCCAIKCIGTGTEHEIIRKLLNIPGLIHEWNPRISDEERILFICIKHHQKLIKMCNDINVVFGNGHLIQLIGSTIGICAACYRITT
EPNFNDLLTCIAYYMAYVGQIFIYCAVSNELTYWSSCVSIAAYHSLWYKKKYANVKQCLSIMMLRSQKPVSMQAFGLFELNYAFF
VTVMRSTFSLYTFLTKMATK
>AcorOR28
MTLNQNELQYTIINTERRILWANGVYTGKQYPRSITNKTVTIINTILTFTFLTSMLIKMILTKDDLGVVFEIIHMFITEVVWALKACY
FVLTTKQFNALEESLKDPVFNEFTRDQKNLVSQQINKTKRIGNIFRNMSLTTCACYILCPILDGDVWAIPLWIPFTDGDPTIYYQLY
ESICFLSLASAHPSFDMLVIGFLNIMAAQLDILNDNLMNSTDRNEDEDFEVQEAKIKSRLRKCVIHHLAIVRFLSKLERLFSFGIVM
QIILSTIAICVVGLQFLRVSLYSGTFLGIMFYFWTMVIEIGLYCWAGQNIITKSTHITTACYTSNWYNCSTSTKKMFFIIMERSKHEII
FRGANFFDISLTTFVMILRKSYSYFAVLVQVYK
>AcorOR29
MSFTVETLQKEDLIHVISFGTRILWVCGLYHCKHLKKHFAYNFARILVIALSLPFPCLIITGLVVSHSDLSGFLELGMFFLGSSWISIE
TTAQIYSLRQVAEIEEMLESDYDYFKPKTELQCQFILAKRRRLVLITQIVWFCVYSFMIIFVLCPLVTNASLVIPMWIPFGNKEFSAY
LYQSFYLLILCGIYTLLHNTFLGPILMATAQFQILKDNLIHATDRSEDEDGFAQEKRIQERLKRCVKQHNAILKLVSTVQGMLAPVF
LGNISFTILGICFTVLQIILATDSGNFILLTSYLGILILQMFLTCWVGNDLISETSDITQACYLSEWYNCTPSTKKMFLIIMSNTQQPIS
LQSIIFPVSFGTFVMILRSSYSYYTVFSQVYD
>AcorOR30
MSTKTGKLEKLSLKFTQDIYQDGVKKCLLPVKVLLQSVCCWPDDELPYGKAIGWIFFSFLFINGVFNATYILMHGKDISEAVGAS
VTVTINFEALVRIYCILRNRRVFNEILVKIWKQFWPVKAVDDKTQAHLENKAVFAITVISIVLITSIFSNTFITTMPFLKYNQLISKST
FPFDWNKHFVYELIYIWQYFLNWYILFAVLAFDFFFVALVSMCAIQFSIWQHVMRNILNEESKEQRRVIFGKMENEMTDKEMLR
HCWQQHKLLNNICDDMESAFSITILLQFVVSTCANCAAFLTMKVDSSQFSKMLSFSMGHTTQLFYYCYSGQELMYQSEQLSHAI
YECNWHLSYDRDFRKALVLMLHKSQRIQCLTAANFTTLDFTSFIRILRLTFSFYTLLDNLVEDTAENGI
>AcorOR33
LPPAAILSNTKISLKPLLVHKLLFKSSFPFNSQALYIFEIIYTWQYFVDWFVMFMACGFDFFFISLMSVCITQYIILQDVIRAVFSKES
KKHRKIIFGERGINMTDKEMLFECLKQHKLLIRICSDLEEAITTTILLQFAVSVGANCIAFLILNIESSLFVEVFPYCGAHLLQLFYFC
YVGQNLTHESGNLSVAIYESGWHLCYDLQLRKSLVLMIQRSQQEQRITAVGLIELNLESFIKLLRLSFSIYTLLDSFLVVDDE
>AcorOR34

FLPMEFVKLITSYQNVKSTMEQLGVVTMHMISTLKIVNLYFKRNEISRIIDELHYNDLTETSGSLERKNLQNKFHRKIRRLCMFFF
HMGNCTSTILCATSLIHLIICKHETVYQEFCSTVQPIVISTPIHIRSLIYSRWIICAFQWMCMFLYGWQIVAHDTLFAAILIKIACNIRIL
QMDFKNITAESDQNITKMNYKMNQLTFQLQKLIRTCQCAANVFQYIILLQVLSSLFILITCLYVAASVPVFGIEFVFQLQYYLTVVT
QLSMYCWFADEVTILFSQMPVSIYQNYWICGDQSFKRSMLINMIRMNKPIYFIIGTVAPLNINVLVYILRASYSYFAIIKNK
>AcorOR35
SDNYCYTFFLVIQIVYVSAMVFTTFVFDVVFHAFLFHAYCELEKIKYGLQHLGISEDVDNDTIVYKKFCNIVKYHNFTLKFLDKIN
DVYYLQLLNHFATFVAAIVFGIFFMNIDGFPPSPDKLSKYIPYLITHQFQLYMYCVLGEIVYNQVNSISDVVYHSKWYIKRQSKLT
RGMLMVMIVSRLKNKPTIGNIWKLNLATFMQVLKTSMSFHAFMQTVYKTDN
>AcorOR37
DEFNRNISKTLAVRYKIVKFIQLGVVGISIVGVLAFFLRPVFISDVTFMLETWIFIDSNLLAGIVLMLQYYYFSVIISVLLGYDFIYMS
LCIDMISQMELLKHKISQILSDNIANVTLELVTCIRHHQILLSVYRRMREVYSLMLLFHYFVTLIGTCTTFYEFLGKSDVPDFVINL
VTVSVLFLQFGCYAFPAEQVALEFFDLSNFTYMSKWYECSIRVQKLVLFIMTISHKELCFSGGGIMDINANAFGSVMRK
>AcorOR38
AKRTHFLYSTVQRMFVTLAILGIILYSFRPLATKGGLVFPSRIFVDLVGFQAVLLFSQYYFLLIIAAVVPGYDIIYICYSAHVIIQIRML
KYKFEHITKNVEIETINSYIRHHQFMLNIFDRMKGVYFWMLFFVYSLTLITGCSQLYILILGNTQLSDLLASAVFITALFFEFGLYTF
PVEEIVSQFTDISSSVYKSLWYERALEDRKVLLYVMMKGQRQSYFSAGGLIEINVNTFGSVIRKIFSFYAILKNVLNK
>AcorOR39
KIASNTKLIYNYTKMVQTFLLCVFITSVHFYFLKPFFNSDDVFPFNVWINFNSLLLNVMVLASQYYCLCIVTPVVLTYDVIYFSICL
HVIIQLRLLKYKISRSSNNTQNELKIWVCHHQLLSSIFTRIQEIYSGTLLLQYLMTLGMTCIQLYILNTGQLDVADTTELILYLATMY
TEFGYYSIPVEEMSFEFLDVGNAVYESLWYETDARTKRSMLFVMMYAQDLKYLNGGGLIRVNIDTF
>AcorOR40
SWTTFCDIDHSVCYAFNYIAQLLYVLWGLMGLLCYDIMIMLLLAAGYREFEQIKSGFLELSIDETVGEENIKALEQIRALVKQHNL
VLDFIDGIGSFLSKILMFQFIAIVFTNCSSLFLLSVVGFPPPASTTCRIIPYLACLFGQNFVYCIAGQLISDQSVSVADAAYGSKWWA
KTQPSLRRAICLVIQRSQRRSQISAGGLINLDLNTFMAVTKTTSSVLAFANAVFQ
>AcorOR41
MPGKKITVRKLSLEFTRDIYKDSVKRCILPGKILLQSVCSWPDDERLFYKAIGWFFFWSFLVVEIFHVAYIVKHFRDISDAVLTGTT
VTALLEALVRLYIILTKRSIINHILLKIWKQFWNVNVVINRITRNQLKKKARVSTILTSIFLVSSIICSIKITSDAFLQNRGMVLKSVFP
FDSTKPFSYELIYIIHYCTVWCGLFVINAFDFFFVALVTNCSIQFAILQDAFKNILTGTSKGQRVAIFGQKHSNISDKDMLLKCLEQH
QILIGICNELEESFNISILIQFVVSISAICAASLILKVDSNQFLKMVMYAAAHLAQLFYYCFAGHGLSYESDKLSDAIYGCNWHLFY
DRDFRKALVLIIQRSQRVQYLTAAGIAKLDFASFIKVMRLSFSFYTLLNSLLAKNI
>AcorOR42
YNAFDLERRILWFYGIYFSKDFRPQKLHYLRVIATSFVINTLVLGIVMEMIVDHNNLETVFQSIYYIIVVIIGQIKTFSLYRSLSQFNS
LEDMLQDVIFNAEISTGCTSISKAVDTYKIFKRIFWGTASFCASVYSLMPLLSGDLSIPIWYPSESFKLYCQLFEIVCIWTIAASLLSI
DVIIMGLIYFMSAQINTLNYNLRNATDRNPDYDAEKQEKQVQDNLRICIRHHLAISEFVSKLEEIFKFGLLLQIFSSIIAISSGGIYVV
VVPLTPSSYLLLGTSMSVVLLQIAMYCWAGQGLITESDQIGESCYMSEWYTCNTATRKMFFIIMERSKR
>AcorOR43
MDYGNNTPIHCARNPHQRKIEGNVKAQRLSDAGLENSAKGDSEPDPELLEQFDSFYQTTKSLLVLFQIMGVMPIERSAKGITTFR
WFSGATIYAYSLFVAETIFVTIIFKERLLLVLQKGKRFDEYIYSIIFLSILIPHFLLPIAAWTNGHEVAHFKNMWTHFQLKYYQVTGT
AIVFHNLTLISYSLCIFSWVLGVAIMLAQYYLQPDMQLWHTFAYYHILAMLNSLCSLWFINCTAKGRVAEDLAQNLHNALESPDP
ASRLAEYRDLWVDLSHMMQQFGKAYSGMYGMYCILILLTTIVAFYGCLTEILDHGLSFKEAGLFLIAFYCMCLLYIICNEAHYTT
ARMGPEFRERLLSVNLMAVDSRTRQEVHMFLTAIDKNPPTMNLNQYADINRRLISSTVTSMATYLVMLMQFRSTLMRNAAIAAK
RSAMNLNRTGTNATT
>DponORco
MINKFKVVGLVADLMPNIRLIQASGHFMFNYYADNSGSLHILRLGYCCMHLFFVLVQYGCIFGNLVKEKDNVSHLAANTITILFF

THCLSKFIYFAARSKLFYRTLGIWNQANSHPIFLESSNRYHALALKKMRSLLYIILFGTIFSASAWTAITFVGESVHFIKDPDNDNET
ITEEIPRLLIKSWYPFDAMSGMTYYVALVFQIYYVFFSLFQANLLDNLFCSWLIFACEQLQHLKEIMKPLMELSATLDTFVPKSAD
LFKSPGSATSQDHLIENDFNAKNDDLKGVYNIRQELGNLNFRSGALQTFGQGGGGVGPNGLTKKQELMVRSAIKYWVERHKHV
VRLVTAIGDAYGVALLLHMLTATVMLTLLAYEATKIDGLNTYAATTLGYLLYSLAQVFHFCIFGNRLIEESSSVMEAAYSCHWYD
GSEEAKTFVQIVCQQCQKSLFISGAKFFTISLDLFASVLGATVTYFMVLVQLK
>DponOR2
MKFFKKTEENVFFGFNIMVLKACGLWPDLIDYRYDKWRLMKDCLMISSLMPCAVPIMADFIMQLYDGVPNLTAAVENMIALNCI
IGMIYMVICFIANRRTIIKLMVNLKYFNKYGNSRKTKEVDEKANLFSKIFMFYGILGNFVYMLMPQLSIDKCHNNRTAKMIDDGV
PCGLVVRSVFPFKFDYKPVFEIIFVHQIYTCTMVSIVVLALTMLLCGFLMHIVNQLKNLREFIAKLRHCPQDKPGERLFFIIQYHID
VIEYSQNTAKAFSTMLLFYITLTSLVLSVLCFEVIMVDAFEDSVRFALHLVGWLAILLSVCYNGQLMIDESVEVANDIYSLNWFNF
PVGIQKKIQMIIMRSQKPLILDAAGMGLVSLPAFLKVLSSAYSFFTLLLKLK
>DponOR3
MMENKSYRIMQLHTNLLKCLFLWPVDTFSPGLNSLLMYGSFCISMCCGIPIISAAGYQFYVGIEDVNILLEALIGVYDIIGNTVTY
VCFLRKQRQIQEIIDDIKDFFQYCGYETVRKIDSEIMCHTKYFLFYVTVSVILNLAWPMLSVNNCLKSRRSDFYIKHDPCGMPTQN
LYPFEASEGIVFWVLYIIEAIFCYHTCCFFSLATVIVIGFLKHITAQLKCCAYKFEHICDYMGSCKNDENVIIREFVHLIKYHQRIIKY
AEKVFGIFDVMIIVYIGVTSFTLAIIGYQIAIPKTNLEDRIRYTMLLIGWVLLFYSICFYGQQVRDESMKVGEAIYKSEWYQHQTLL
GMKTDIMFVMRRTQKPLDFKATLLGEVSLIVFVAVMKRAYQLFTLLLTVTEDGP
>DponOR5
MDTLGPALTKVRLRLPSLDIPDSKGTDYPKNLYSAIDRISFLCGQAKLSKNRSWILRFAYSLYSYTLIIIAIMFIISEIITFRKSLTELTT
VLSEIGMMFTHLVGMVKFWILIHKRDEIEQVKNKLRDVQFEYVGIDDFQPGLKMRKEKLFIIIISTFIFALYNFVGISAHISAASMM
YKYTANGNFLGNTTCETFVPYYFYYPFDVSSPSSCHYLLFYMDLSLDIYASYIATFDSVFVILLNLLATQLNILGDALRTIRKRCVK
RLQMKVDSSSLYDADNPLLENEMYNELTHCTKHLYLLLEVGNDIESIFTFLTLLQTIASLLIFASCLFVAARVKPTTPIFYSQLEYFS
AVLSQLTVYCWFGNEITLASSAIPYSIYSSDWFSSSERFKKSMLLTMARLQRPLYVSIGKFTPLALTTLLSVIKGSFSYFTLFQSAGT
D
>DponOR6
MDTLGPALTKVRLRLPSLDIPDSKGTDYPKNLYSAIDRISFLCGQAKLSKNRSWILRFAYSLYSYTLIIIAIMFIISEIITFRKSLTELTT
VLSEIGMMFTHLVGMVKFWILIHKRDEIEQVKNKLRDVQFEYVGIDDFQPGLKMRKEKLFIIIISTFIFALYNFVGISAHISAASMM
YKYTANGNFLGNTTCETFVPYYFYYPFDVSSPSSCHYLLFYMDLSLDIYASYIATFDSVFVILLNLLATQLNILGDALRTIRKRCVK
RLQMKVDSSSLYDADNPLLENEMYNELTHCTKHLYLLLEVGNDIESIFTFLTLLQTIASLLIFASCLFVAARVKPTTPIFYSQLEYFS
AVLSQLTVYCWFGNEITLASSAIPYSIYSSDWFSSSERFKKSMLLTMARLQRPLYVSIGKFTPLALTTLLSVIKGSFSYFTLFQSAGT
D
>DponOR7FIX
MANLKQALQIYDICSFLEGEHIRLGIGGFYPRRIKRTFIVNLVTVFAYIITIAQMAVVINFVLSITDIVTITEVLLFSMTQVGFVNKLV
NFHRNSRKVATLDELISQEIFTRVTVAEMDIMKTSFQRCQKVLNIFLLSCFGVTLLYGVVPAVNGIMTGTKMYPFPGKFPFNPDDY
FVLIYGGEVATVAVSAWNNGAMDCLFTKHTVIATTLFRILRKKIKDLHYNTNEGERPLENRIKHCVRYYNEIIKYVSAIENIFAYGI
LVQFMCSAIVICLTGFQLLVVASESGQSGLLVVYLFCMMFQLVLYCWYGHMLMEESNRITEACYAINWHEMKIGQQKMLITIME
RAKKPIALKALGIFRLNLSTLMTILRSSYSYFAVLQQIYRNDKIMALVTN
>DponOR10
MTIIRSADNEQFIKLAKVALISTGVWIMPITENRSVAFAFKIYSLFMKGSCVMYFSLLFAETIRLIIFKYDMDVILASVGVLFNAAKI
MLKVFIYLKYHILEHFEDVIEKERALWNSDNEELKALYRTKVRHCNVFVITVFTSSLMAVTALQLSGAFTAFELAEYSKANNITIE
PHVMYQSLFPFSKLDNLYWWLASQALWWWVGLTYNTMTHAVFAILLIYAATQLEILQIRLRNCIEPEFSETPSQMLIKEKVLLLR
KLTQDHKYVIDYVKHFNECTKYAILLEFLLTSLDTASVSVNIIKMKGAELSWLLSFLVLLVMQISLIAWTCNEIQVQSMAIADAIFA
SRWYCLLDKEAIAYVHFMIVRAQKPLLMTIGPFGPMTTASALMVFKAAYSYVSIMKE
>DponOR11FIX

MNLSKFIEFPRKTLVLTGSWPQQHSSWPYLSRRIIVMTSIALLLLALVYNASFHFDDPIKLSESLFILISVVNVFLKLIIMVVNEKVF
LNLIARLETSTFIKGGILYQPIYVKFMEIVRPVYVTYFILVCGCVTFRSSFPVFSLSSVAYDNMAIDLLVVGVVSIAAVQLQVLNSKL
RDTKQNVQFLPNYSISNHEALTVGYLKDCCIHYSDIEEYIKCLLDMFSIIILVQLGSSIVVICSSGMVLLSLKPLSIEAISLYFYLITMF
TELGMYCWFGNFVYVESLEVINSCYLSHWEERGPAVRKTLFMLMERAKRPLEIKAVRFFTLSFDTFIVILKWSYSYFALLRNWM
AD
>DponOR12
MEKDKFKILSLHINVLIFLQFWPNPLFNNVVNNHIMSIVCFITVTSCIPCIWTIYKVFEGMYDIGILFESFICFVNIMAYLTAYWTIFR
NKAVIENLINDICIFLPYCPTNLIRDTDASSIRYTKYLIVYVTLGVFVNLAWPAISPEGCMRQRQSEYLLKHDPCGMPTHNYYPFDA
SKPIPFWIAFACEALLTCNICILFSMVTAIILGLLMQITEQIKHCCDKFEHINFKGDVETARKEFLECVRYHRAILEYAERVFTVFAPV
MSAYLVVTSFATALIGYQIVETDNTQDRFRYAMLLLAWGCLFFMICLYAQILQDESVLIADALYNSDWTCNSIYFRHYIIRVIARAH
KPLYFNISFLGKISLTRFVSVMKTAYTVFTVLVTVVDRK
>DponOR14
MLDISVSAVSGNCVIFAYTLVILLQCVIEVTGQLYLLTTKPSIGKILLLAPMFFTSFMVITTIIALLFQRSSHGRIYSQYEALSMEQLR
RANKPILAEVRQLFDAGIYQFAAFLAAVALGSIGYLPLNEFDYEHNVWPAIQALKLIEDGPKAVIYTIATLNYLVMPGKGCILLLYG
LHIMHLCSLYCVSSILLRGKLKGIAVNRPAAADLMLSDQAWVTQELKSCIKQDVRLKTFCSAIIDHFKWILLFHVALAVIILSLLYYI
NLFLNGGYSPLGSRCFVAIANILSFTYSYNSENFREQVENIRQEICNLPWYSFNKCNQKLVHVFLSNMLQPRYLSVGGLFDANHEF
LVLVMHKVFNVLTILSNLDPDRAGN
>DponOR15FIX
MGGSGEFSSNILNLGISALHLCATNRVSRWFLIKNEFEVILANLRKINTDFSLFDYQSHQSGFKMDANEPEIHNEEYIGQMAWRNL
NCTQATGFKLRYADDTASSAISNAIIKYKTTKLLETKRYCLGIFLTFLINVALNISISYTINYGNPLYEKWNPLLNKTSVYRDYPYPL
LYPFDTSVSDGHYLLGFFYQPYAFFCLMCAFFCIEYLCVGTIIHLTTHVNILGYAFSYVDENIDPMLDYSKVIMLKEKRIIKLSGEL
KEIYNCAKELNAVFSGQLLMQEFLMSTVMCCCVYRVTTNISTAEIGYLSTMVAVCVAEMFTVSWFNQCFTLELFKIQQRIYELEW
IDYPPKLRRVLLFLMCRVQKPFNFTMGFGFPLDVNVFLSMIKTSYSFYTLITRSGSKFSNEDV
>DponOR16
MDFIQLFAPFKLILNISGFWPQKNPKPIVEVRKVFTLLVNLFFCLSLSIQCLFLRSQIEEFLDVLTVITPPVAYLFKQIVFFGHSGAFLT
LMDFLKDDDLVSIPLQLRKQISDSLQVAKIIGVGYQACCTMTILFIVIWPMFTEHQLPVQFTLFDLGDFYAFMYLLQIFALANAAA
NSSSLDLIALTLMCIVKGQICVLNDKIRSLGEINASKGHGAQRVKYVSGCVLHHTKIIELVALIENVYSQIVLIEYLTSMVVICNIGF
QLVIVELASFAFLLMLTFLVTMLCQLGMYCWFGNEIMLHSAAIRDACYESDWIHSCPQVRKMLLMIMERSKRPLYLTAGKFSILS
LNSFTSVIHSAYSFFALMQRMYGKSTTF
>DponOR18
MKDRVKLMHSFKFIMILAGLWRLKLTDNRFYQWLYLLYSMVLQALFTLLTFGSIRNISEAISNRDYTGVIDNIWLSLLTMVILFKM
IMFQRKRFLDLILQSIDEESKIYTEETTCIQEIFEHNYKIVKHIILLVCVCVSSSGFSVCVCNTITYFQYRHINQTEMKPMMAPHIQFP
LNRHKYFMETYLLSIGTTFGGVIYYTLTQVYFVVVTAFVISQLKIIQRLARDFHLCSNGGDQEKEALTVFRKIYNKHLYIIDFVGEL
NVLMKYLIFVECTVISIIIASVLFQLIFVPTSLAASTLYIAVSCTVLSQIFVLSWISNEIEVESLSISDALFESRWYEQTQKVKKIIIIIM
MRSRKPLRIMIGPFYPLTIHTALNSLRAAYSYVTLIFAMSSSGQLQI
>DponOR19
MYPIRKDLPFYASLRMLESIGFYSENTNGFKKRSIVRTLIFCILCWSIILLSAVLLIYENLNDKNYASVFFNIAVAVASTSTYCCTLLF
VKYQEKWSDILTALVNYEKFGKPRRYNQLKERGDRVAMACWGGILTGVFLYMLFAILHENDCELEKTGGGVCGLIIPTWLPAPY
DNSLLARRLVLLYDIPNAAAVSSFVLVTHLNIQVNEFNIARIDHLSLLFNDIEFCKDPQAQLNKMKHCIEYHQDIIRVSLQFKNLSK
RTMGHMTLTFTIVTASMGCQLLQTSKNFYQENAFFFEIIYVINMFIMCYCGQRLEYKMKTVGDFLYSTHWYNLNPKLQSLIPLVI
LNSQKTIRMDAVPIGYLNYELFVTLLKTTFSYFSVLTQLT
>DponOR20
MLYPVRKGLPFYHNLLVLKLAGYYPKSSNYNKKLFFYCLVCWMSLWTGTLWNLIILLYLSIQNKSSHGITEAMGYLIGNSSLALI
CLHFALKHQDWSHLMDALIDFQKYGKPPKFNTVQTNASKVGITFFKVLFCAAVMYCVLQVLLEEECEKKLTFGKTSCGMLLPT

WFPASHAESKLAKRLLLIYQLLACWAIAPFTVIVSLVLQANEFIAYRIDHLKSLLRKVGSNENPDLQLHQFLAYVQYHHHIIRLCR
KLNYVAKYTTGHVALTFVTVVACFGHHSIQEKSLQSLTYQATYVISMCILCYAGQNMQDQMRSIGDALYSSTWYNCSLKVQKMI
PLVLLRTQQPIGLDAVPLGVFNYMLMVMVLKTTYSYMSFLSRTI
>DponOR22
MICSVAFGCFPWEFYFPDNKSRRRAYEIYGRIMFAYYIVFIFTIFVQLVVMLKRPDFDIDAVCANMCVTLINTVTLFRQLVFHFNPK
FKKIIQKVIDMESQVYRKGDEKICSIYTKYVKGINKSLKLYFCFIGSLLVIFCVRPLLADPVEQRVGDEVKLVRPLPQSSWFPIDTEE
QYLYVYTWGCINCMISAFFVTCSDLIMFALLTQPMGHLNILHEILQNFDEHKRNFALRNQIVNDDIAAYWTLVDVIKHHNEIITYV
NEINDCMSFVMLCDFLQSSLQVACILTQALENDIDVFLVLFMISFIGSMFLRLILYYHYGNELLTTSQNIALSAWQSNWYDQSPQV
KIMMFTVIARAQKPLKFYLGQFGVMSLQAFISVLRASYSYMTLMYGLN
>DponOR23Fix
MAIYPKCRLIQISMISSSLVGTFPWQFMFQDNKILKNMYAMYSKLMLGYFTLFVFSQQLELLILITDEEVMRNAIFANISVTPIYTIT
LAKQLIMMLNSSFRATIKQIIDTEKCKSPIEDDEVFEIELRIVQRSNKLVKYYGLMMFVLGTLFCVKPILMTPNIVSSGNTTKAIGFF
PLSSWFPFDEQKHYPYAYMWQTLSLLQGTMYVTTTDILMFNLIVFTAVQLRKLKHLLKNFVHYKERFMTLYNIVDDEQAAKITL
IYFIRRHKEIIEYVRLFNESMEIVMVFDFLQSSLHIASVLPEVLMSEISLMVVLTVASFLGSMLFRLSLYYYHANNVIILSAELSYSIY
ESNWFDQTPKVKQMILIFMLRTQEPLTLRIGGFAVMSIESLIAILKATYSYVMLMI
>DponOR24
MSVLGETTQIKKKWRSSIAITEKVLVITEIWPNDDTSLYRTMKVVFITIVCIVFNLTVIDELKMLAIRQDYKTLSMHLSTFGLYIGFS
VKIILFQFTKHGPLKNMLDSMDSPIFHAYPPEMQKHQDNCIRVSNLIGKFFVYLVGGTILFYLNKPFYSSYPLPITFSHPLTTTTFYL
LLTLQCVCSFYLIMIGICFDMLVMGLANVATAQLDMLIEEITTFTPTSIETLEKEEHRFIKRCAERHNAIISYVNSIEDVFTYIFLAQC
VVSVTCICNGLFQLTHVAPVFSIHFYYNCIFTFNVLFEIGICCWFATLMTNKGNDVADACYNYNWLHSSSATRKLLLIMLCRSQKP
LFITVGKIIQLSIGSFLSVLKTAYSYYALMQHLYDKTSQ
>DponOR25
MAIYPKCRLIQISMISSSLLGTFPWQFLFQDNKTFKNMYAMYSKLMLGHFTLFLFTAQLQLWILITDEELMRNAIFANLSVTFIYNI
TLAKQLIIMLNSNFRATIKQIIETENCKSPIEDDEVTNEIEFKMVQRSDKIVKCYGFLLIVLTILFFVKPFLMTPTIVSIGNTTKVIRDL
PISSWLPFDEQEHYSYAYIWQVLNALQGSTYVASTDILMFNLIVFPAVQLRKLQHLLKNFAHYKEKVKTLYNIADDEQAAKITLV
YFISRHMEIIQYVRRFNESMEIVMMFDFLQSSLHIASILPEVLMSEFSVMVVLMVASFLVSMIFRLILYYYHANNVMILSAELSYSM
YESNWFDQTPKVQQMILIFMLRAQEPLTLRFGGFGVMSIESMIAILKATYSYVMLMI
>DponOR27
LLLCVIQPYNCLKMAIRTSLFQFAKYFMTFCGLWKVPFSPKVQRFYVVFSNVSHFVYCSFVLSLFVKALLIVVGFESSDNVFNAIS
VAVIMFDINFKAMIYVKFGLPRLFHQLMKEEQSIEENSHQEISEYYLRQCELYVNICTLQLVTTIFPTYFYILVNILQFPLDAENFMY
EMWIPFPVQWKVLAIFKIVICQYGIFMNTAVRSALQSLMMFITSQLWILQVNIRNVFSEFSEEAAREELGKLIRKHQFLIGFVEKVN
DSVKYILLLEYLLDSINMAAAMLQIATASSVTEMTFTLVYFILLLTQLVILAWSANEINTQSVEVSNAIYQSNWMDQ
>DponOR30FIX
MFIRNQRAFVARTCKLAGLYPVQLLPEDENLRKLYTIYYQALIMLYFICLISFCTELFHLLRAEKATVDDILKSISMTTLFAMTALR
QWVIRSSPDVQKILRKAGNVEQRVYEENDPEVVNIFERAGHVALLYYIYYAVGTVFLCLGCILEPLYDNQKVFSGNATAFSRKLPL
PLWFPYDIQAHYWETFCVTILLICLLVVFQVAVDVLFFYFIRSPVIQLEILHHFFKRFNDYTGRISVEPGNVASNVMMRKCIDMHR
KVIKFVDIFNENFSNIIVLDFVQSSFRLASISAAIIMIESFTVTSFVFTLIFLWITLVREYYIYHAGNEIIFLSSGLVHSVYETDWYIENR
QFKYMVKMFMVRAGKPLDIKIGRFGSLGFPALLSILQASYSYVTLVRGIQKS
>DponOR32
MNSKVNQAEKLDPVDKRSKVFPSSDFLRLYAIFTGQMGIFPWQLMFERNKTYQNLYNLYSKLILSYYMYVTVSMWLALVFLCL
EDTLRIPEITKNITVSVICTVTIIRLFVMKLHPAFLRNITFIIDAEQYILSSNDAEVHRIYKNCKIISNRHTIFFIVLSYLMALFISLRPFF
TDAYEINYKNESLQITSLPLSIWVPLNEQEHFLSVYFWNVLNNLVMTSIVLSIDIITFLLLIYPVGQLQILHHILSKFENYKNRMKLN
YPGLDDDTIGAITLKACIDLHRNIIAYVDDLNACMNIFMVVDFAQSSLLLTSVFAQLLWVEPSITFYGFVFMYVTYLNQRLFMNY
YYSNEVWLLSENLNLSVWKSNWYEQSHYVKFMIYFFIMRTRKSLKFKIGPFGFMNLSTYIAILKASYSYIALLHSTQK

>DponOR33FIX
MKNDFFGFCIPLARFIYIMPDKTPQNVYGWRNKLWAVFMYGLAVFCHLTEIIKLFQIVTAKYFLLGEFIRNFVITSLHFTSLGKAMF
IGGKTGKKAFEKILDFEKHVYKNLGDDIRLIYKNKVTSIQKVKKYYLIGIILVVIFYVAAPIFREPIHIQDGNQTIRFRQVPLSSWSPF
EQYYWLTFIWTGLTGIYLSIFFVTTDLICYSYVQMINEGMKNLMVLDFLPGSVQLAGMIYQMMTNLSVIQCILLGQFICSLIARVFI
YSNSANNLSQLSKQLAVDWFEIDWTELPKDVTNNLNFCIMRSQKNLQITVGDLSVITMESFLTILKGTYSYLMLLMTI
>DponOR35
MKNDFFGFCIPLAKYIYILPDISTQDRTKPLLHKICAVIIYVLALFCYFSEVVKLYQIVTGEYFVYDELIRNYTVSYFHFTSLIKAINI
KGAISARAFKTIIGFEDNIYNGEDEDIRKVYKASVTPIQRVRKYYMAGMVMVVICYACAPAFRDPIEIQRENETIRIRQLPVSAWSP
VEEYFWLDFVWKSLVGAYLAYFFVTTDLILYSFIAFGACQVRILQHYIHNFNRYCEEIMHTEGVPKNESARLLQKQLIAMHQDVI
SYVNMINGSIKQLMMLEFIPGSVQLAGMLYQLMTNLNAIQCIFLGQFISCLIARIFIYTNSANDLSQLSQQLAADWFEIDWIELPKD
IKMNLNICILRCQKNLCITVGDLNAIDMTTFLTILKGSYSFLTLLTTI
>DponOR36
MKPIEDQSLFRACKILVLCGGMWRGNIPNWPIAHQKLYKVFLRGAQFAYFFCLPSLVLSLWVNVDQDNEKAISVLKNITFVVVIF
CKMIIIQSRPVTMLIEAASEKEQQAILSEDPQISEIHRRHVVYTEFVVKSIMLCTFLAGLAYVVGDLYLANEFYKLHPNAAPTDPKP
HSIYFWFPFNPDEYYKIALTYEFVHIVQTVIYNGASHAVVNSAIIFVKVELKILEYEIRHMMSKPNLSNLTPAQLMKIHIRKHQELIK
WVCKFNDSFKYIILLEYSVVSLTLASTLTEILQGIKMVFNGIFFLLSTLSLFILSWNANEIIVTSVFDLSDALYHFPWYELDKEAQEL
VLFMMLRCKRSLNISNGPFGFLTLRGAVSRLKLAYSVVSVLSR
>DponOR37
MFSASKWVIMSSGSWGLEVDSKYRILYKIYVLYIRFIYITSTVAVFAMFLVNLGSNNDKAIEALSLTLCSVSCIIRLAVCLKQKVVN
LLKIVMEDQFNYAVNDPKIKVMLQEYKSYVTFLCVFVVCYTYSLVILFNIFNGIIEFQSFRKLHPNATEYPQYLVSIWLPFNVQTHF
TLALICQTVLFLQSCVLNYSSTVLFNTLMIYVVIKLKILQHLFQNFNTYPKNLENFHMELRDVLAIDNLKHLIRQHQDIISFVKELD
KNIKIGVLIEYTITSLMLATISIQVLTGNKVASFSFYGLILIYQLFLLSWNAAEIKTQSEKIAGAIYATDWYVYGPGVKQIIHFIIMRCS
KGLSLDIGPFGPNDLGAASARLKLAYSYVSVMGNNK
>DponOR38
MKPSKTKKAQLISWTKVFMIGGGFWNQPLTNSYIGEKVYFCYSIFMKCGCFMWWSMMVGELFRLVAYGYDVEIILAQFGLVVN
ASKIMFKLVVYIRENLLALFKDITEKDVEIWNLDNEEIHTVYWKNIKLIKSYVLALSVSTSLCLGMLDVSGIHVILKTVEHNKAFN
DTLEAHAMYQTILPLNKLDNLPLLFTLQAYLAIIGFVYNCLTHLMFATLLVYAATQIQILQIRSKNFIGADQLSGSDMRDKLLVLKE
ISQDHQYIIGFVENLNSRTRYIVLVEFILSSFDLASVSVNLITLDFSSSDIAGQLIFNLSFFVLLSIQISILGWSCNEIKCESEELANALY
ASNWYLLNPKGQKMMQIMMARAQKPLIMTIGPFGAMTTNSVLAILKGAYSYVSIMRK
>DponOR39
MSDSKSKKVFAVMRIILMCAGFWNQSISKNRLINKIFHGYSVFVKLSCLVFWLLILAETSRLIICQYEITIITASVAILLTDTKIVVKIV
IFLKHNILDIVADVIENKDEIRTEHYKEIKMLYDRKANFLKFAISVLGGSTTGAVFLLQGSGGALVLQDRKHNKLFNDTVETHGM
YQTIFPLHRNNNHIYWLFATEVFWSYLGITANIVTQLICVIILLHAASRLEVLQVRFKHLIMPDFQIKASDEDMKAKVTELKSIIRKY
QLTIRFINEFNQSTKYITMIEFCLSTFDMASCCASLTKMKGYESVWLLFFMMVLLTQLYLIGWTANEIRVQSEAIATALYESNWYE
LNKEGRQLILISMIRAQRPLNINIGPMGPMTTRSILTVLKGAYSYINIMR
>DponOR40
MYSKQESLLGMLKPMMMFTGTWRLDGMNSTVRWFYWLYSLIFHGFGVLFIISVVAKFVEFVKSGADSEDIISSQVFLLSGTCIFS
KFLIYQICNVSDILKAILQEEEKIWLSKDSESISAYQADIKHVRKWNWGILLSTMFTGVALMSAGVASLIQADISSINSEGNEKEEW
SMIPMWLPYNEREHRSTVVVLKCIFTFIYVCMFIVSGMTFVALMIYSLGLLKMEQVKIGKCNWTSYNMADLSVDMKTLLINNRR
VFRFIKHLDRSIRYVVLVDVLLNSISIAALATNITNVQRGDFVFCTGFLLMQVTQVFVLGWFANDIIMLSRTRADVLYNLNWYYL
DLKNRKLFGMMLMQCQQLSVISIGPFGPMTIGSVISVIKAAYSYMMLMQSYK
>DponOR43
KFFLKLSGLWPFKISDNVLVDKIYRFYTLCQICYYLCVILGLSINLVILIIRFDEPQRIIRDINLFIIAFEICLKVVIFQFRNVPHMLYQIT
GYEDTIEGSSDAEVKAYYAKDAIYCRRINVFQFIATFLACASFAQDSVVIFLTSDDMSVFKETPFMHDLWYPFNRADYIYLVICIAF

ICDTQGLICNTASQTTLLCVMIYARTRLKILQIRLRKFDKIAVEEYEGDVVRAVKDLIAEHQYLINFVKSLNDRTQHVLLLEFMLNSLCLASGTSQFIIIDTTSGWLATVFLNLYVIVQIFILSWHANEISVEGLAVSDAIAASQWQKQSKEVQKLLIIMMMRAQKPIGLTAGPFFRMTNSTAVQTMKVAYSYASIMTQNMPE

>DponOR45

MAKTLSFIQLSKFYLLLSGLWPFKISDNFLVDKIYRIYTLCQICYYLCVIFGLLVNLVILIIRFDQPQRIIGDINLFIIVFECCLKVVIFQIGKVPFMLNQITQFEDSMEASGDAEVKDYYAKDAIYCRRINVIQSIATIIACASFAQDSVVTFITSDDMSVFKDNPFMHDLWYPFNREDYIYLVICIAFICDMQGLVCNAACQTTLLCLMIYARTRLKILQIRLRKFDKIAVEEYEGDVVRAVKDLIAEHQYLINFVKSLNDRTQHVLLLEFMLNSLCLASGTSQFIIIDTTSGWLATVFLNLYVIVQIFILSWHANEISVEGLAVSDAIAASQWQKQSKEVQKLLIIMMMRAQKPIGLTAGPFFRMTNSTAVQTMKVAYSYASIMTQNMPE

>DponOR48

MMTLHVRYPLNRNKYFFETYILSLSSTLTGAIYCTISQVYFIVLTAFVISQLKVIKRLARDFHLSTESNFNEEEALTAIRNIYNKHLYVIRFVHEVNLDIKYLILADCMVNSIIIAFLIVQVLFVTTSLSSLMFYVAVSCTVLSQIFIISWFANEIEVMSTSISDALFESHWWEQTEKVKRVISIIMMRSRKPLRIMIGPFYPLTIQTALNSLRAAYSYVTLIFAISTRNTMHV

>ItypOR1

MWITMFCTKNRHVLISLVQGLSDFTGFPPNFDKFMQQLNFYSKIHLCYLTGGSLMYFVLFAPLHKRNCDELKREKNLTETCSLLLPLNAPFVEYQSFGKFPTLQLLNIIIFLSLMYMYMCAGTIVWLNVELVEHIRIRIRHLKHMILRALKSNDKQFRREKFRKAVRYHEYICSMSRLADEFFGTELFLHVVLTGAILGISAYLIGDGSLETVMIFVGWLNAIIMGSVAGQRLINESLGISDIIYEVDWYNFETALKKDILFFLVYAARNLCLLGLGMW

>ItypOR2

MKVLQRETEITFFKFNIWVLKTCLLWPEDLNYKYDKKRFIKDLTMVASLMPCFLPILADFLQQLYEEVPDLTEAVENMIALNCLIGMFYMVICFVRNRRMIIQLMIDIRTFNKYGNDSITQEVDNKANLFSKMFMFYGILGNFVYMAMPQIRVSKCHLNRTEDMIEKGVPCGLVVRSYFPFKFDYSPVFEIVFVHQIYTCTMVSVVVLVLTMLFCGFLMHIVNQLKHLRVLIARLKNVPPEKFERKLIFVVRYHVAIIQYSQNTAGAFSTMLLFYITLTSVVLSVLCFEILMVDAFADSVRFTLHLLGWLIILLSICYNAQLVLDQSQEVANDVYSLDWVSILSVDVQKKSKXVIMRSQKALVMEAGGMGVVSLSAFLKVLSSAYSFFTLLLKFK

>ItypOR3

MPTRNLYPFDASQGWSFWILFVIEAIFCYHTCCVFTLATVTLIGFLKHILAQLRYCGHEFETIFDGVNEESGGKHLTLQHFIRVVKYHQEILRYTEKVFSTFNVMIVVYTGVTSFILAITGFQITSPETGGEDKIRYTMLIIGWALLFYWICYYGQQIQDEASQIADAIYNSKWYENTNTVVLVRRDIIIIYLRTKRVLDFKVQFLGAVNMEVFVAVMRRAYQIFTLLLSVT

>ItypOR4

MDKQSQILKFHVKVLKFLMIWPFDGLDPNQNYYLMRGCFAYACFCSIPVFSGAAFQFCVGIDNVKVLLEVLVGVGNITGYNIAYVCFLKNQEKIQFLIRDFQEFVQFSGPEIIQNTEEKTTRYTKYLLGYASIGLVITFSWQMLSTESCVAQRGGDYYVRHDPCWLPVRNWYPFDASQPKLFWIVFPIEAIYSIHICLFFSLATSTIIGFLMQITSQLQYCSNRFEHVFDEVDLKPFQQIKPDFLFLIKYHKKILDYSKKLFNVFDALIVVYISLTSFIMAIICYQIVDPKISAQDRIKYAILLIAWCLLVYLICYYGQKVQDEALKIGQSIFKSHWYGGTTAVELKPYILFTLARTQIPLEFKAQLFGTISLLQFMKVMKWSYSGLTLLLAVTDED

>ItypOR6

RYATDFFFGKPMDVKKGWFTEALKQKQGSKYSLMFLMLAHATLTSSYVFSTITTIQHMKGNSTVALPDRLPYYSWMPFSYDTGPKYLLAIGRIKQVPMFSYAYSIVGMDSLFMNIMNCIAANVTIIQGAFKTIRERALPGQPNNVPHESKADMDVLRVELRKIVNHLQTIFKACDKLENVHRMVTLCQVTATLFILCTCLYLVSIAPPLSKQFLVEFVYMLAMSFQLYLYCWFGNEVTIKFQELPRYIWASSWLATDTQFKKALLFTIMRTKRPVFLTAGKFSRLILPTFMSILKTSYSIFALIRNTSK

>ItypOR7

VFFDQLRVCYFNVWGGTLNGQRLQWKEKLSFSRKFPFNPDDYYVAIFLGEVIAVAVSAWNNGSMDCLFAKHAVIATTLCKILRKRISTMLQITEDDRTIEDKLKHCVIYYDEIISYVTKIQRIYSYGVLIQFLCSAIVICLTGFQLLVVSSKNGKIGLLLVYLTCMTIQLVLYCWYGHILTEESNGITMACYVVDWHELKVTNQKMLIMLMERAKKPLGLQAMGVFRLNLFTLMKILRSSYSYFAVLQQIYKN

>ItypOR8

MSELFLLHFPRILMVICGVWRLPYFKSKKVQTVYDIFSIFLQFTFSLMCLSMFFELVNLINTWNVLNLIEFSRVALSSYLCIIKALVL
RNSSIQRIMVYMIKEERNVLRSKHQQPKALYMDTVKLINRVSFLLLLVVLPTLLAFSADCLRKGIIDFDDAVKYVYLPLIDQKKY
KTVQLTVQTIFINLIGFYYCMTQAFMVTAMKFAQGQLELLQLYFREFDYYAARQSTTEIAYLKTLLEYHQKIIHFVETLNKEMRLV
IIIEFFFSAVNIACSLFTLLTMATNLIDILFSVNCLVFLLAQLAILSYLGNEIYQAGLNIASASYELKWYEKNREFQKNLLLVIKRSQK
PLVLSVGPLGPLTNETFVSVLKASYSYFNLMTRYN
>ItypOR9
MFSSKNLYKYYSHFASTSIIMYTIMLTIRLVQLVIEGQTPSAKLYRCFTINIVIYMMTANLIIFRRYGLPDLISQVMKDEEAALNSLD
KDIRKTYLAQTKIYEFTSVAQVVSTFASGLMFIALNVYMKVKGLLKHEAFMYELWFPFNRENHDGFVIFFNLYIVVLIMFCNVAS
RIIPQTMIIYANAQLRVLQILLEKAFDAPCSDPLVKIQELVKKHQDLINFITFLNSALRNVIFMEYIINAINVAAGLLQFITVRAAMDL
VYAFVHFSLLVIQIFVLALNANNVSTQSEAIANAAYNSQWMDQSNNIKKIIYIMIMRAQKPLVLNIGAFGVMNAESALTTMKAAY
TYVSIGLQR
>ItypOR11
MDVIKDILRNFKENSLLQAKFERHLKVAIKTKKRGDFLFLYGTFLGFGTQLFWSIYPFSQNKKLLPVNGWYPYNPMNSPSYELTS
FFQIFASAFNISHTMNIDSFTINLMMQTGMQCDFLQLTLKNIMQFHTVDGILCQSLQQNTEPLEDILTPNLKTCIRHYIEIKRIARKL
EDIYRTSMAVVFLGGAFIFCAIFYQMLHSQRDPTEIFYLLFFLFSMLTEQFIFCWFGNEITFKSGQIHGALYTIPWVDCSVKFRKML
>ItypOR12
PFNPDDYYWPIFFGEYFATGLSALCNGCVDRLFAKHVSIATGLLKILRNRIKQIMDDDDHNVIEAKIKHCVLYYNEVIKYGKVIER
QFSFGILFQFLVSCLVICLTEFQFLVATSSGSFGLLFAYLACMITQISIYCWYGHQLMEESDSVSMEFYNLNWLDMSIKNQKTMLTS
KERAKNSITLKASGVFQLNLTTLMTILRASYSCFAVLHQIYTK
>ItypOR13
MSNYQRYNIQTFLKEERLFLGITGFVSGNLKLHLPTGVTYVLTTMQVLASIYYGLSTTDLAEITAAFMITLSHINTLNKLFGLHLK
SLTQLDRILVKQIFALADDRELTILKRTLTACHNMLTMYLVTVLGSILLYGATPLVANYTTMERNYPTLAKFPFNPDDYYWAVFAG
EFFIVALSALSNGCMDRLFAKHVAIATGLLKILRHKIKQIMDVDDQKVIEAKMKHCVLYYNEVMGYANQIENQFSFGIFIQFLCSCI
VICLIEFQVLLATSSETIGLLLTYLTCMITQVTIYCWYGHQLMEESNSISMEFYDLNWIEMSVKNRKTMLTSKERAKYPIVLKASG
VFPLNLATLMKILRTSYSYFAVLHQVYTK
>ItypOR14
MEYSFHYHDGFRSHMFLYVMLQGKIAVIILIDYVCSINHMKNCSETTMKHLKIIIKKHLEIIRYNQFVLKSNQYFYLLYLFNEVFFG
LLPMFEYFSPNRSIIAVSSATVYFFCLVYAVNDAAEEYITLVEYFRTEIFNLKWYEWDVSCRKVYSILITYLNEPMKVDFVVVNINR
ALFCQILKLCIQYLISFIQPTELVDSSFCNSALYVLSLIISHYW
>ItypOR16
MYNIPENERKNYFLKFSRVTMLMLGIWPVRRGGDLLEKLYESYFLTTFLYYIAFNLSGLALAIRTWSNNYLTTASSMGIVIEYMSN
AYKVWLFKTSVFKSLIKEIQDREREIFEGPDEAFKEIYIRNAESNKKVVLFYTIMGTSGISLYFITPLVSNVLMPLGYNNVTGVYEH
YFIVFNWFPFDPNRYYWAAYLIQFTGCLIGYSYIVHCGAFYISILNFIRTQLKILRHVIVNMSEYSLLYKNTYKLTEEQSQFVLLRAV
VLEHQRIISFVTKTNHTIQLFTLINFVISSFQLALLVYQIFQVAILQQVTVLSYFITLSTQLFLTYYAAHMILFESSNIASSIFEGNWDT
YPPQTLKLLQMICMRAQKPLAMTIGPMAAVKVTALFQIFKALYSYICLIIKF
>ItypOR17
MGLMTTPRFFLQLWGIWPVNTLLPPKYVMYRFCIIGWYSFFNIFQFIASIRLILNNESFERISRCISVMVTLVLMLVNSLIYQKNCIP
QLCSTVMEIEQXLARSNDAKITQTYHTTVAKNKYLNLYIVGSSLFTLVAFIGLSLLDVIKAGPAFWDFDNVTFMHELYVPFNRGN
HQALIITTNIFTACESVVVNGVIQTTFYALVMYGALRFKILQLNLKKIEQTEGDRKWRMRELIQDHQYCIRFVGELNQATKNVLL
MSFVLNSLKVASVLFPLMAIREFTDLAFPLIYSSMLVSEVVFQGWMCNEITEQSLQVAQTIYDTFWYKESKQYNVLLQLMLMRA
QRPVTMRIGPFGAMTTSTILTTMRAAYSYATLMMNSS
>ItypOR18
MYTLSKNKPFYYALVLLRAFFWYPSSPKCSVTFILCSLVLRLLSTLAALGTLAHLVLNLTGETKAEISEDIGDLTGFVGCMSACLN
FLWHRSRWSSFINRLLTFKQFGTPPGYVKVVRRGNLITLACILYTIPGMLWYSHLTHLDIPRCEALNREFGMKEACGMVNPTWIPP

GYDRRNGWRFWVLYVLQCTGIFVYLPSTFVISNIPLEAVGVIVTRIBHLKYHLKRCGSDLGRLYHCVKYHQDIIEVSKELSDLVQA
TLGTLLLTGAVVIGSLGSQVIKASTPKAVTFILGYVTTIFMVCHAGQKLINQSLTLADQVYWMEWYEQQPKIRKDLRFVLARCQK
PLGLVGPPSMGXAGYSLFLIMLKTSYSYLTLLNEVIS

>ItypOR19

MLIFGNIISANVIVLFGSSLINGDYFLVTSSFPFALAIIVVNGSTLSFAVNHKQWSNLFKSLTDCQKFGKPPNYDLLKKNGDRKGMI
CMTCYISTCTLFAIIEAVEEQRCLKNVSKHEICGFIIPVWWPTNYHPSTFLKTMVQVYEVGTIIILSNFTIIATLQYQVCEYIAAKAA
HLGLNFNAIDPNSDAKTQFEQFKLFVDYHQHIISLCAEFDSSCKRTVGHVTFTTAAISALFSYHGMQGNYKLLAFLALYILNLGF
MCHTGQNLEDAMLGISNSIYSSKWYELNIRVRQRIPFLLARTQKRIGLDAVPIGYLNYALFMTVLKTTCTYLNLLNHTI

>ItypOR23

MAVYPKSEYLKVPAIYCSTIGIFPWKFMFQDNKNLQTIYRCYSIVMLAWCIGFVVTDYIQLVILLTSKTLDMQEISFNTCITLLFTCI
GLRAVIVYFSPNSANLIQSIIDSEKVTYLDDAECMKLEKKHLRSVRLISHCYFIFIIFSTTSRCVYSFPKEPDIIQNGNETEIVKEHML
SIWFPFNQEKYYLTVYNIELLDSFLGTFFVAYVDIYTFNMISYPKGQLKKLQHIMKHFHNYKAKYSSETNEENDFIVFKDLVQRH
KQIIQHINAFNELMEFVAIFEFVQSSAQIACGLTQSSLENLTIGSFLFVMSFLISMLVRLFLYYYAANDVTVESTKLAQCIWESNWY
EESQKIKLSMLMVIIRAQKPLIFKIGGFGAMSVQSIVTILKATYSYITLAYKRT

>ItypOR27

MLNGDVLKMDAICSNICLTLAFTCSALRATVMRVGPNLLKIIEQVMHAEKNPASIEDQTSFNLERKSIKTMRKLSHLYAVAITMIA
SSKCALAPFEKGEIVHIGNTTIIDRPLIMSAWVPFNKNTHYWAAYIIQIYFAALGAWHVAYVDMFMFNMLGYPIGQLKKLHYYIK
NITTLTRNDDSLEEFKNVIRQHQQIISYVKFYNDSMGTFAIFEFLQSSVQIASIFIQTSPSDMNLGQFGFIGGFFIGMLFRLFLYYYTA
NEVMTESEKVGVSVWESDWYEQPTNLKMALLTVMMRGQRPLYYKIGGFGLMSVQSIVAILKATYTYLTVVVRNN

>ItypOR28

MGLYPASRYFKNPIMWSSILGAFPWQMIFQENAKLQQVYRWYSNFMLTWYFGMVTTEYIQLYHILNANVIQMDEVCENVCMSL
VFTCTGLRVWVMRRTNGLSEIIQTVVDAEREADGLDDEKTRQYEDIHVKHMEKVSFIYAAFVFMSVTNGCLATLYADTKSVIIGN
STIVEKPLIISTWFPFDKNEHYWVAYGLQVFDGYMAALTVACTDILMFNMISYPIGQLTKLQHLVRNMAVYKTHFEAFPTFTKIVQ
RHKHIIKYVELFNQSMGTFAIFEFVQSSVQIASVLVQTSPDDLTLMSFCFIVLFFTSMLTRLFMYYYSANEVIIQSINLGDSVWESS
WYHQPHQLKQAMLMVLVRAQKPVSYKIGGFGIMSMQSIVAILKATYTYISVILRN

>ItypOR31

MHIFPKSDHLDFSFALCSMLGILPWKLVFQDNSFLQTLYYLYSKTLLIITVIFITTEWMEVCRILNXDPVNLTDLNNAIAPVLLFTVT
AIRMIIFNRNPDFMKLLNYIINRQEFMAQRDDEIRKPSQKFIKINKWTGVGYLIMYLAVIYQLLALPLVLGPIEQQTANQTTTIRILP
LLSWIPFDTQQHYWGCYLWQALNLQLASCNICHIDVLMFALILYPIEELGYIKHVLRNFDSFKSRTGIENSNFASITVFKDVIKIHN
NVINYVGTVNDTLSFVMLLDFLQSSLHIAVILGAVVVGGPTDLASLSFVGTHFFSMVLRPFLYYYYANQVMVLGGDLTKEVWNV
DWFDESKDVKYMVRFFNMRAQKPLQYFVGSFEVMNLQSFISILRVAYSYVMLLHTLQ

>ItypOR32

RVKQLRLVSILCGLIGILPYKFISPDKKLYQDIYRAWSILSNLIFVLGMFLAYMKLYTLFNEEKIRFVELSRNLAVTMLCTMTMARQ
IIIRVKPEVSNMLSQILETEEAILIKNDKTVAEIYTKNANNLYRKSVYFWFIMFNVVVSHIARPFYIFEEIQQGNVTVIHKTLTLSLWF
PVDDQEYFWECYTISSLYAVCFASFQSYTDIFMYAMITYPVGQLQILFHTIKHFQTYKMELQRLLDSMNDSIAAKLMMKNCVEM
HRLIIKYIEDYNSCMSILTVFDFFQTSIQITSILSQIVMIEITVFLAVFIMIFLTMVFYRLLMLYYYANEIIITSEDLCQAVWQSEWYNE
PPHCKFMMQIMMMRSKTPLMLKIGPFGSMSLRAFLSILQASYSYFMLVYSQKDN

>ItypOR34

MKFRELIHNDFLGICIQLGYYFCIIPEKAVTTDKIESRNYFFYTCIVRTLILYCHICQWVKMYQIITADIFIFDELVRNCAITSIHFQSF
VKTSIFRQNYQLFENVIDFENVLYKNNDQKVLLIYRDTLQAIKNSRLVYVFGILIVIVFYIAAPLFRGPYYVEMGNETVTIIQLPLSA
WSPTNNYFSNFAVTGAMGAYLAMVFVQTDLLYYCFLYFSICQLNILEHYIVHFHHYSNELVNDHKCSHVMALSLTQKIYIKYHQ
NIIKNVKQLNDALKNSLLIDLVPSSIQFANQFYIIATNLNIMQCVCIGFFTIMLMSRVMAYCYLANQISVQSQKIGSAWFQMDWSD
FPNEMKKMISFCIMRAQKPLVITLGNFGNITLMTFVGILQASYSYVMLFITL

>ItypOR35

MDNQPKFLLFTKRLAFALGIFPSKLTFQDNNWRNQCYTLYTKFCLGLFNLYLLTSFVQLFVIMSSNPIDFTELSKNLIITPLFTVTVI
RQICMSQPGFIKLIQHLISHEMYLDTXTDKYVAYIIQSSNIVFCSTFDTVGEILLTTILVYPTVRLKILKHVFENFQKYERNFTDPHSA
DRLMKVCLKIHTDIIRYVEQFNSVMGTCMFLDFIQSSVHMACVLAEILTGDVSLIELVSTSAYLVILNFRLFILFTTTANEVIVLSQG
IGVAILNTNWYKKNSSV
>ItypOR36
MKIPHMFEHAMKHERNIFKSRDLELIICYQEQVKYGRRVNLSQFLVTTLSTSTFAVTALLDVYWAADMSKYEKEPFMHDLWFPF
RRETHMNWVIFFNLFMIVQGTCFNTATQATLINLMIYSSSRLKLLGLKLRKFDAIASQNGRDILETVHDLIFEHQDLLSFVESLNV
RIKYVLLMEFILNELGLASGIIQLIVIDTTSYMVSVVTIIILQLFQIFVIAWTANEITIQGAKIADSVMASNWVEQPTNIKKLFLIMVM
RAQRPLGLTAGPFFNMNANTAVSTVKAAYTYLTFMMNNYN
>ItypOR39
MHDLWFPFRRETHMNWVIFSNLFMIVQGTCFNTATQATLINLMIYSSSRLKLLGLKLRKFDAIASQNGRXILETVHDLIFEHQDLL
RFVESLNVRIKYVLLMEFILNELGLASGIIQLIVIDTTSYMVSVVTIIILQLFQIFVIAWTANEITIQGSKIADSVMASNWVEQPTNIK
KLFLIMVMRAQRPLGLTAGPFFNMNANTAVSTVKAAYTYLTFMMNNYN
>ItypOR43
MDAALALMKNITFVAVVVFKTVVVQSDAIVKLVKAASVEEEKIRNLTDQAIRKIYKSNVDYCNRVTKVIITYLYGSGTIYVLDGL
YKSYTYYENHPNVKPEDPKPHTVLFWFPFDHNRYYKIAIAYESFHIFQTLNYNGVAQSVVSSVMVFLKIELKVLQHHIRAIQGGS
RDYQKVLIKCAIKHQQIIQWVNDFNNNFRFIILFEYSMISLTLATILIDILQGTKICFNATFFALNFTQLFVLAWNANQISDESSISISD
ALYACSWYEFDKTTQDFVLFMTLRCKKPLNISNGPFGYINMDAALSRVKLAYTVVSVLSTSTK
>DvalOR1
MINKFKVVGLVADLMPNIRLIQASGHFMFNYYADNSGSLHILRLGYCCMHLFLVLVQYGCIFGNLVKEKDNVSHLAANTITILFF
THCLTKFIYFAARSKLFYRTLGIWNQANSHPIFVESSNRYHALALKKMRNLLYIILVGTLFSASAWTGITFVGESVHFIKDPDNDNE
TITEEIPRLLIKSWYPFDAMSGMTYYAALVFQIYYVFFSLLQANLLDNLFCSWLIFACEQLQHLKEIMKPLMELSATLDTFVPKSA
DL
>DvalOR2
MLYPVRKGLPFYPNLLILKLAGYYPKSSNYNKKLFFYCLFCWMLIWTGTSWNLIILLYLSIQNKTNYGFVEAMGYLLGITSFTLA
CLHFALKHEDWSHLMDDLMDFQKYGKPPKFNKVQTSASKLGIAFTTGFFSAAVIYAILQVLLEEKCEKLSFGNTSCGMLLPTWF
PASYAENKLIKRLVLIYQVLACCTMAPYTMIVSLVLEANEFIAYRIDHLKSQLRKVGCNGDSDLFLASVQYHHDIIRQVGQTFSIY
MDLCAHCVF
>DvalOR3
MMAPHVQFPLNRHKYFMETYVLSIGTTLGGVIYYTLTQVYFVVVTAFVISQLKIIQRLAREFHLYSNGGDQEKEALTVFRKIYHK
HLYIIDFVGELNVLMKYLIFAECTVISIIIASVLFQLIFVPTSLAASTLYIAVSCTVLSQIFVLSWISNEIEVESLSISDALFESRWYEQT
QKVKKIIIIIMMRSRKPLRIMIGPFYPLTIHTALNSLRAAYSYVTLIFAMSSSGQLQI
>DvalOR4
MAGMVMVVICYVCAPAFRDPIEIQKENETIRIRQLPVSAWSPVEEYFLLDFVWKSLVGAYLAYFFVTTDLILYSFIAFGACQVRIL
QHYIRNFNRYCEEIMHTQGCSKNESAKVLQKQLIAMHQDVISYVNMINGSIKQLMMLEFLPGSVQLAGMLYQLMTNLNAIQCIF
LGQFISCLIARIYIYTNSANDLSQLSQQLAVDWFEIDWIELPKDIKMNLNICIIRCQKNLCITVGDLNAIDMTTFLTILKGSYSFLTLL
TTI
>DvalOR6
MMFLAHFHFDQNVHYKRALLLQTITVLLSIIYFCLCPILYLGVLGFIRAELQVLQYRFRNFDSCKRSVSDVEMMKQLIQTHQFIIW
FVKDFNESVRSLILIEFLISSVNIACVAFQLISANRITDDIFTPCFLFVLFGQLLVLAWPANEMSMEASNVAFNWRKYTTVFQSVGV
SIAIYDFPWYEKSIPIQNMARVVLMRAQSPLKLTIGMFNPLTTDTVIKVDFFFRGFATGTEAIGFRC
>DvalOR15
MKPSKTKKAQLISWTKIFMIGGGFWNQPLSKSYIGEKVYFCYSIFMKCGCLMWWSMMVAELLRFVAYGYTLDVILAQFGLVIN
ASKIMFKLIVYIKENLLALFKDITEKDIEIWNLDNEEIHTIYRKNIKLIKSYVLALSVSTSLCLGMLDVSGIHIILKTVEHNRAFNDT

LEAHAMYETILPLNKLDNLPLLFTWQAYLAVIGLLYNCLTHLMFATLLVYAATQIQILQIRSKNFIGTEQLSDSDMKDKLLVLKDISKDHQYIIGFVENLNSRTRYIVLVEFILSSFDLASVSVNLITLDFSSNDIAGQLIFNLSFFVLLSIQISILGWSCNEIKCESEELANALYASNWYLLNPKGQKMMQIMMARAQKPLIMTIGPFGAMTTTSVLAILKGAYSYVSIMKK

>DvalOR17

MSVFEETTQIRKKWRSSIAITEKVLVITEIWPSDDTSLLRTIKILFITIACIVFNITVIDELKMLSIRQDYKSLSMHLTTFGLYIGFSVKIMLFQLTKHGPLKKMLDSMDSPIFHAYPPEMQKYQDNCIRVSNLIGKFFVYLVGGTILFYLSKPLYSPYPLPVTFSHPLTTTTFYMLLTLQCFCSYYFIMIGICFDMLVMGLANVATAQLDMLIEEITTFTPKNKEPLEKEEHRFIKRCAQRHNAIIRYINSIEDVFTYIFLA

>DvalOR22

MGAVKVIFWYFYGHLLRDIMNALENPRLHYEGYMDFSPNRISHLYKAIGRRYSLLFLSLAHATLISSYIPPLMAVAEYLTQPEGGIQQLPSRLPYFCWMPFSYDTPGKYLLAVAYQAGPMFSYAYSVVGMDALFMNILNCIAENLVLIQGAFRTVRERSSLVPHWDGIRESPLVRRQMDLEMKKIIKHLQITLRACKHVEGIYHVITLSQVTATLFILCTSLYLVSTASPFSKQFFAELVYMMAMLFELFLYCWFGNEVTLKASY

>CbowOR1

MIGFLKRQTLVDKLKIHVTFFFILILDVLILVKISTTHNKTLEDIMTSYEAVGSYLQMTTKILTLIIYNGDLKQILAMTNQFWKYDKFGPVISNKQQKYPRMMPSFITAYFFFCICTLTVLMLKPVLFHELPRSCCIPEGEVWFYVVSAIQNETLFYCTFTVFAFDAMFALLYTEAAMQFCLLNEAFSRMKNHGDLKECVDYHVFLYNFVKKLNDVYWMFLLVQSFDCLSETCFQLLTMVHTQENLTLRVKAVLYAIALYMQLSFFCFPVGFLQDESQASSTAISACPWYLKDAKFKRSVFIVMIRAQKKISVRAGGFFEMDRQAFIYLCKSSFSVYTLLKSIN

>CbowORco

MMKFKVSGLVADLMPNIRLIQASGHFMFNYHADNSGALHALRLGYSCMHLVFCLFQFGCTFGNLVVERDNVNDLAANTITVLFFTHCITKFVVYFAVRSKLFYRTLGIWNQANSHPLFVESNNRYHALALKKMRTLLVCVMATTVLSASAWTGITFVGDSIHHIKDPDNENETIIEEIPRLLVKSWYPWDAMSGTAYYASLIFQIYYVFFSLAHANLMDSLFCSWLIFACEQLQHLKEIMKPLMELSASLDTYVPKSADLFRAPSANSQDNLIENDYNAKNEEINLKGIYNTRQELGINFRSGALQTFGQGGGGVGPNGLSKKQELMVRSAIKYWVERHKHVVRLVTAIGDAYGVALLLHMLTSTVMLTLLAYQATQIGGVNKYAATVIGYLVYSLAQVFHFCIFGNRLIEESSSVMEAAYSCHWYDGSEEAKTFVQIVCQQCQKAMSISGAKFFTISLDLFASVLGAVVTYFMVLVQLK

>CbowOR3

MATITNSLFVILEIGICIIKFLPFKNDPKKIRKTLFALNQDMFNRATESQRRFIEETEAACRNIFAIFMTFCLLSLFSWPIKVLFYEQRRFPIDVWLPFDPFENVSIYLGVFAYLFIATGNAPIGNAAIDTLIAGLIIHAACQFRILKDNLRCLSQRADEKLNGLPQELKEMKRNEIVYRNIRECILHYDAIYDFVKEVEKTFSVVIFSQFAVSILVICISCFQLSIAEPLTITFFAMVIYVVSLLLEIFLYCYYGTVLYEESNTLIAAIFDSEWYDLDEKSKKALFILMERAKRPMMLTTGKLLSVSLETWTMIIRRSYSLLAVLKNHQ

>CbowOR4

MLFYVCGFAVVICEYMMFKESIKDIGKFVSHIGMVLTHLAGIVKFCLLTIGHGKILKLMHVLQNKDYQYCSLEDSKPGEVLRKGQTVNNVIAYSTFVMYTLVGITGHISSVRNLNEQIKGDNFEGTNKTCYDFLPYMFYIPIPSETKWQCEMVFNLMDIGFALHAFVIAAHDGIFAGLLICLKSQLLIVCDVYKTIRQRSLKNMHLPENYTITNDMENPALENEMYRLLVHSMEHLKILLWVRDELEYIFTMVVLTQTVASLFILASNFYVASTILTASLEFFAKLEYTFCIFFQLSLICWFGDDITRASDLIKLSLYESDWLSSSPRFKHAMVLTMIRMQRPVFLSIGKFTPITLSTLVAVCRGSFSYFALFKSIQK

>CbowOR5

MIKMIQIRFHTLGFLACMKIFMLSFGQLLSKTLPLKCYSAKWIPFHVMWFYQSFLTSVCIIMPIIAMDLLLMTFISLTHIQFKMLNLEIDRVFRRSERAKKSEIARLVDHHNFLIDFSNRINNTFSTMLLAYIFVFVISMCVEMYKSSANPSFSVFMNAVTYLSAAVFGLIFLFCIPGQNLTDEANNIPNAVYFTDWYRDSKQSTSVLMMISNGQRDISIKAGEVIKINLATSLSTIKTLLSYFMFLRTVVLDE

>CbowOR6

MYTIKKSQPFYSSLRTLRFFLVYREFVQKSTFMLLSFFSSFMSFSAFLVFVCGCILHAVMSIRENIGGDISEDLSVSIGGLAMMVNVAMFKYHQDKWSNFFKDVTNFEKFGKPTDFDATKDRANLLSTLYMIYCITGTIVYSCVGVIESSCDELSEETKQKVICGTLAPIWLPFEDVSLTVRNTILLVQYVLANYIITPSAVICFLPFETTELLICHINFLKDKLLKVFGNEDGMIRNDKLRFCVAYHTHILGMADQLK

YVVKFSVGHMSLVCALVFGCIGNQIFRAKPVGAVIFLLGYMVSLFLLCYAGQRIMNESLSIVDVIYNSKWYKGNTQIKKNVRFM
MARCQIPVTLDAWPFGIFSFPLFMMIVKTSYSYLTLLRQST
>CbowOR7
MISPTSYLIKLIIFKSKSVHVLEMLSFLELTEFNNYPKGLSGIVERTVKFSRYLGYAYQFMCCLVITLYSTIPLFTKADLPIRFSHDVG
KLKPAVYIFQVIGLSSAASNNSCLDVLAMSLMGICSAQIDILNKKLITLGKNEDEDETDGSNNSYLRLKKCAKHHVEIIRFQKALE
RVFSSIFLAQFATSVMVICNIGFQLVHVQPASVQFALMLFYFIAMNTQLVMYCWYGNEIIVKVYHCLSILSSAIRDACYKFEWFDS
NMETKKLLLIIMEHSKKHLYLTAGKISVLSLESFTSVMRTSYSYFALLQTLYRNNQD
>CbowOR9
MLTFSLIHITSLVQVSETLSFNLTQLAYLCKLLNFQIHSKRLLELEDFLRKTTLTNVTVEEEAIIRNTMKGSRRLATVYRSLCVIIVFL
YALFPLIDENSGDEKKLPLPMWFPFDTNNHFGKVWFFEIFSIAIGAWTNSNLDVICVTMITLTTCQFNIMNSRLSNLRKSTDDVEE
EDTVQKALKECVIHYNDIISFQILVETTFSLSIFGQFVFSVLVICMTGFQMLVISFKSVQFVLLLSYLLGQTCQIVMYCWYGQSILDS
SEAINDACYSSEWFNCSKETQKMFLIIMERSKRPVKMRAGKFFFLNLDTLMSILKSSYSYFAVLRHIYSSKFT
>CbowOR10
MMVKQTKNEAKRFFRYIGMLFTPTQALGCYVLLRIKQHGIEKVREELLDEQFHYKSCGSFRPGKIFNDAKSFCDKFVVITIILYSL
VVASAHISAYVTLNLAFEGEYFPANITCYDFMPNYFVIPFPTPTKSSCKNALTYMDVSLNVYATLLASYDTTFCSVLICFKTKLQIL
SGAMRSMRERVTTEMNLPLNSSLELDDPEVEAKLYEEIKQCARHLESLLSVCKQIEDIFKYGTLMQIVNALVIISSCMFVLSITPQS
DPDFFVMIHYIIALFVQLFTVCYFGNEITEVADELNNSLYQSNWLSCSKRHKQCMIIMMSRMQKKIHVMIGKFSPLTMNMFVAVV
KGALSYCAVFRAVDNAEI
>CbowOR11
MGAVKVLFFYFRGDKLIKIMATLESTDLHYEKCQKRKFFPGSISTNYKKVGIKYTLLFFMLAHATLISSYIPPTIAAIQSELDNPGK
SLPDRLPYYSWMPFKFDTSTTYLIALGYQAIPMFSYAYSIVGMDTLFMNIMNCIGMNLEIIQGAFLSLRERAADKIAGPLMTQDG
LHNSHELKTALNREMKKVCRHLQIIYRLCEDLENVHTFLTLAQTVATLFILCSCLYLVSTTPASSKQFLSEIVYMVAMGFQLILYC
WFGNEVTLKADMIPFFIWQSDWISADREFKHAMIFTMIRAKRQLHLTAGKFAPLTLTTFIAIIKASYSFYAVIKNTST
>CbowOR13
MDYGYPKNFFEANDVVKRISGIMLLQGKEDNIFFKWYQIIYIIFVYSSTVVFTVGQYIMTKNSVNKISNLVSSLGVLLTTHVGHFK
FWLLLSKKKELENLKNDIEGENYQYATIGNSNPGLLLTNEKKFCTVCTYVYLAGCYLIGIFGNITTVVRLNGALTGNNTFESINMT
CNDFASFTFYIPFFTANKWQCIISSTVMYSGMAFESGIHAACDLLFFAMIHCLKIQLRIIADVFRTIRRRSLLKLNVPEDYTVLHDEE
NSALEEELYRQLSHSTEHLNILLRVTQEIEHLFTYVLLAQTLSSLLIVASFLYLTSTISINDADFFLQMQFLLVILIQLALLCWSGNEIT
EGFQLIKTALYESDWLSCSHRFKRSMILTMIRLQRPVLLTLGGFSPLTLATLVGVCQGSFSYFTLFKSFQ
>CbowOR14
MATEFQKAFETEKWILSLFGFYPQWNPEPLWKHARRVFCIAVTLTYIISMCTGPFLENSVMILCTVMGLSKMVQLLTSKRQFREIE
HYISNMKPSIIRRSSLIGAFRVSVVTLVGFLGIMPLSMKNQRMLPYKSWLPYSVEGASPYYSTFIFEVISIVMAAFTNSTIDMMYYC
LVDICCAELDVLKLNIIEIDMSDHVDIVEDELKKIVIHHHKIIRLVGIIQEIFSSVVFVQCMASVLVICFLGFQLIYVDKLPSVKALIEL
SFIACMLIQIFCYCWFGHNITMKSSEVGDICYHTKWFESDLMIRKIILIIMERCKKPVELRAKIFTLNLQTLLAILRSSYSYMAILRT
LYTDE
>CbowOR15
MKEVHFKNTVLNFYEFYNTDFKLLKFFGIWIPDSSNSKFHKIYFIVINFVFCAIFNLAQVSNLLHEINNLKNLAACGYVVAIACMA
NVRSYYFLKNREEFLYLIRSLNDSHFQPESEDQICSAKKSLRFYSKVKMIVSILCTITVFISMSTPVFYKKNELNLPFASWYPFDVSS
YPIYQIAYVHQCISVIYVTSINTYVDIIMAGFNTFIGIQCDLLCSRLYNISKDHSSEENETTLLDCIRHHKLIVRFANNTEILFNRIYLG
QFIACTSALCMALFLLTLHQESRFESSFLVFYLTAIFSLLFIPCWFSSEMQGKSENIPEAAYSCNWVTASKLFKKDLIFFILRAQKPL
KFYAVGFFQISVETFVLIVRSSFSYYTVLNNMIMKEG
>CbowOR17
MNIEHIKEIEVLENSLKFLRIFFLFPRKSEINDPKKNVYWKFILLSLSTAYFSIGAAIHLVVNVRSGAFVNVDKDVGTIISYYGALYFI
SRYLGNIKYIIILYKQFSDFKTYGLPNNFEKTNKLLNKFSKIYFVYHMFIVTGMTTSTLLTIGTCEEENLENNINDICGLVGPTWLPF

EFDYFPLKQIVYGYQVYCSFVIFQLAGHLSYTLMESVEHLIIRFEHVGHTFVEALNEKNSYTRREKFYVAIQYHNDVIQMGKLLN
SCFAPSLIVHISLTGPVLGVAGYRFLTEIPLDSTCLFFGWMFSTFIVCRGGQRLSEASLAVGDVIYRVNWYNLETDLQRDLKMVML
RSRKPVYLRAGPFGPMTYSTIVTILKTCYSYITLLKQTM
>CbowOR19
MFRVGDAIAFQSTINYMTFFKVFTVQTDTRYVGTILRFWSFCLICLFNTFHLVYYVKIENIDVDTSEDLVVILGGMGILLICIFSASSS
RRWTSFLHNLIDFEKYGKPDGIEHAIERGNYWAHFFGLYYIIGTVIYGIVTYMEAPSCHRLNNEKNLHLICDTFVAIWLPFDIPLKSI
RLSVFFIQFVLITCNVNPAAMACFLTWECTEILRCHLRHLKKHFHKMVKEGDVRKRPDGIGYWIRYHNHILSLSYELKSLFKISVG
HTSLISGLVIGCTENQILKSKPLGASLFFLGWMAAMMLLCHAGEILMEETLSVADTLRDSQWYLADLETRRDMVFIMLRSQKPV
HLEAMPLGVMNYALFVMILRTSYSFMTLLNQSS
>CbowOR20
MRDVEEAEKSLMVTGWFPFDTREYFAVAYLFQLQIAIIGGLFLVALDSLIISLIMVAPLRLKVLANYFRHFGDKKSMNSLLSLKNLI
SEHQGIIRYVEDLNASLKWLFLADFVVKSYNISIVLSNAVSIYEFRNIVIIIYYVLQTRGRNKSELAFSALFLCFLLSQLYCFYFHAN
EILLESTNLAENIFKSKWYEQNSQIKRSLIIVMIRSQKPLQITIGDLHAIENILFVKIVKAAYTFLLFQYLGL
>CbowOR21
MKTQFQKAFQKEKWILTSLGSYPQWEPEALWQHCRRFFCMTVSITYITLMCTGPLLENSIMILCTVMGVVKEMQLLFSKQQFRE
IEEYIGSMKPCKIPISRLLGSFRNSVVTVIIFLGLMPYTKRSLRMLPYKSWLPYDVSRAPVYYYVTFVAEVLTIIMAAFTNTTIDVLYY
CLIDICCAELDLLKMELMEIDMSESYEVVQNKLKNVVIYHQRIIRLVEVIQDVFSSVVFIQCMTSVLVICFLGFQIVYYVDEIPSGKA
TIEVSFIGCMLLQIFSYCWFGQSIMMKSLEVADVCYNSNWYDADLRIRKMIFIIMERCKQPLELRAKIITINLQTLLAILRSSYSYM
AILRTLYSD
>CbowOR22
MSSFSESPENEEQPFSATTKMMRLLCVYPLGFEKWQMVRFYVNVVVVKLFSFFFCCVLCLLHLVMTKIDGAHKADLSEDVSMI
MAGTGMLATNLLFAYKVKKWNSLMGKVADSPEMRNIQNFEAIKKRCNRLARLFTMYCVIGAGIYLLSGYYESLVCIRKNEENG
SNEICRTLMPVWLPFRLSSAAELTLFALQAFAGINLSLPGANMPFLVWEITEMISLRISHLKKISESIVVEKNIKSRQERLKHWVMS
HQQIIECISLLNEQVRLCFGHISTIAALVLGCLANQAINSVHLGAMAELGGWMVGLFLLCSSGQKITDITESVAEAIYAMEWYSTD
VQTMRDIRFILMRSQKPLVLQAGPLGALNYPLYMMMVKASYTYLTLLANTI
>CbowOR24
MMTEKDEKMPKKLEIVCMPTSIRIFRFYCAFPPSDKLLNPGKMFYIRFALIALFSSVVLVGSTMHLIKNVKDRTYNHIELDFTYIVS
NLAGYGLLCSYFTKVNAAVQLYLILSDFEEFGKPINFDNTNKKFNKYAKYQYCYLESITVCILLGSNMFRGAQCRKDNAELDQH
EVCGLFAYTWLPFDIDFFPVKQIYLACQLFGIHYVYMMAGLASWMVLESVEHIATRLRHVSHFFNEALKEAEQRKRREKFNFAV
RYHVAVLDLESKLNQTFSVFMFTHMVMSGIIMGYGVYSYMKGKNVSTILIATGWLIGLLMDCYSGQRIQDESTLVGTALYDADW
SDADDELKRDIRFVMMRCQKPMIVQATSFGIMDHPLFLAVLKATYSYVTLLSQSDL
>CbowOR26
MKHKKLYNFYTIFCTSVWVTFILSQLVYMFSSFSNMDEMTSIIYVAGTVTIDLVKMLAIYSNMDRIKPLLNDLNNPLFQPKCKEH
VELALAVKKFHSRLFYFCLYFGVQTYICFSAIPFILEENVTLTQGWFPIDWTYSPNYEIVYAFQNIVILWNTLIFLNLDTFTSGLLMQ
VGLQCDFLSTTFNKIDAFHVSSGVLIENGEQMALSLKDNHEFFNRVMTENLIVCVKHYRKIRRLATEIEDIHHISVFILFLGGAIIIC
ADLFQLSIVQTGGVEFVLFVSFLMCMLMEQFMYCWFGNEIIFKSDNIFAASYNTPWLDCDLKFRKILLNFMTQSIDPIGLKAGGL
FTMSIKAFVSVLKSAYSYFTLLQRIQEKECSELN
>CbowOR27
MIPSEDNSLSSMWLTKLILKSIFMWPDDYSDTKRKTFYKISMTICLFIQSGLVLNLTQNYHDWEKNLAVVSSMSTIFQTVFKMTAL
YQNSDHIKFVLMCMCRKFWPHNLDNENSEYIFKQSHSRRMRLMVFLLASGFLFSLGSVISPMFTRDTPFKSDYPFNWRRSPFYE
LIYLIQVAANGYLINMTVIGFDFLFMDICAALTNQYVLLGSCFERLGTENMQDFYARIRERGCQKWPPKVGGARRFLGICVQHHQ
LLTQITKVVGHIFNVVAFLQLCSSVVAICVSGFIATKDDVTTSQIATMGSYLIGHLIQLYIYCSVGNELLFQSSTLTNHIFGSNWYNL
DSTTTKKDIIFIMKKAQIPAKLNAFKVFPLNFATFIAVVRLSFSSYTLLTSITNK
>CbowOR28

MYASNLDWCLKLNFLLGVHPAKQKSFTQTLQYLFIIFGSCAIMILTVLLLYYKEDAVSMKDITDVSTNFTMFPHGMIKLTTLYMK
RAEILDLLRRTKEHFWQIKDDREDVKRSYKLAKLLKNLFFNSVVLFIISAIVKPIIIGGNTLTYKCHKPELIPRWLFLIFQDAMCVAI
LFTLSCTDVLILTLLILTQIQFRMLNEKIQTIHDNEVDFHDRLKECVDHQNFLMDFVDRFSKVFSKTILLFIGNIILSLCMCMYIITTE
SANINVQMEALFHLIAGLNEICLCYSIPAQTLMNEADEVGKNAYFSKWYEHPKDAKLILQIMIRDQKRMVITAGDFVRIDMEMFL
TACKTIVSYCMFLRTMSMVDQ
>CbowOR29
MTCNEFASFTFYIPFFTATKWQCIISSTAMYSGMLFEAGIHAVHDGIFFGLIYCSKIQLQIIGDVFRTIRQRSLSKLNIPEDYSVLHDE
ENPALEEELYRQLSHSTDHLKILLRVQDEIKKLFTYVLLAQIISMLFTLASFLYLVSTISINDPGLFLQFFWFMTILLQLTLFCWSGN
GITEGFDSINTALYESEWLSCSRRFKKSMILTMTRLQRPVLLSLGGFSPLTLATLVGVCQGSFSYFTLFQSYQ
>CbowOR31
MMAFGYPKNFFHMNEATVRFLGVWLPSKKHHILIRLLHPFYFIFVYSTLIYFVIGQYMKVEMKNVTTIISSLAVLLTTHAGHVKG
SLVVFGGRRIQEIKDILQDVNYQYYPVGEVNPGSTFQKEKTFYTMLSYALLVGFMMPGASGTVTTASRLMIEMKGNNTFESIDK
NCNDFITYTFYVPIFIETKWECIISSSLMYSGMTMYEGIAHAAHDGLLAGLLICLKTQLLILGDIFRTLRQRVLSRLNIPEDYSVIHD
EENPALEEEMYRQLCLCTEHLKILLTARDKIEKTFTYMLLMQTIASFPVFASSLYAASQTPLSSTDFYTNIDFFGCVLVQLAMFCWF
GNGITEAGEAIRSALYEGDWYSCSPRFKKSMILTMTRMQRPVYLSIGRFSPLTITTLVSVCQGSFSYFTLFKSL
>CbowOR32
MEEDQKIYHFKTLKYILILMGQWKFRNRSRFFIKLYELIISRLVVAYLILSAHMFLINVAFAWDCKARVMEMLTAYMQNLNVIIVT
LIMRSQRMRNVLRYVQHYENIKLKDENIAVRDIYMQYVSINHKICRLLIGVVVFTALFYFATGMRNSFLISSTEECPMMKGIMFQL
WYPMDTKKYFYLVVLNDNLIIINIVITIIHAKGLIIANMIFAISQMKILQYELTLVGQIEQETDEDVELRVKKCIMIHQEIARMMDLF
VSASKDIILMQYFITSSELALYLLQMMLADSFAVFGRLLMNFIYLFVEVFLLFWCANNVLVESMAISDVIYNESNWITLSNGAKK
DMLMMLSRAQVPMAFKATFVGNISLETFTKLLKLCYSVVAFLSNVVRE
>CbowOR34
MTFFFKIKGLSPPKTTAVKIIYLSLALPHILMCTFVLILSEWMAFALSQQTFKERMFNMSVATLDTILVFRTTVWAFNKAKLDEIRAI
ITRKSFNFRCFDLLKVGCEQVLTVGRTKEVEKERGLSCKEIKQLWQKAKFVTEKKCNEMDVFRKELMLNTRLLCCFIHLAITIISV
LTYTLSFVNDFTNDTYEAYNPILNRTSLYRKFQYPLYLPFDTSFDGYYWLAYFYNCYAHLGNIITFLPIETTLTCSLIHLISQTAVLKE
AFKYVDENNFGYQSFGEASIIKEIRIVKCINEIQEIYRAVELLENLCNVQLMVQYGFATFLLCSICYVIPLVENTMEGICCLIFFAASL
GQIFTFSYCCHTLALELQAIGVSVYNLDWTNYPSKLKRTLNISILRTQKPANLTAGKIIVIDLLFFIQVVQKSYSFYTLITKTN
>CbowOR35
MSHNEIVCMTTSIKILRTYGVFPSQSRELHPGMVFYIRFLVLAVVTSLTLVGSTLHLIKTIQNNEYNYTEMDLVYIVSFVTAYALIGS
FVMKVKASGEMFVFLSNFEEFGKPINFDKNNKLFNRYSKYHYVYLESLILIILFSSNIFKSKTCRLENELYNLKEVCGLFTYTWMP
FNIDYTPVREIYLTIQLLGNHHIYMLAGLVAWQVFETIQHIIIRIRHVKHLFVEALQEGDVKVRRKKFNFAVRYHNAVLSAFFSLED
KLNAAFGIFMVTHMVLTAAVIGTGIYCLFRRRSLSSFLVCMGWFWGLFMDCFSGQRLQDESLELAVALYDSPWYEMDKEFIKDI
MFVLSRCQIPMKLRAYAFGVIDRAMFLAVMKGTYSYITLLRSQ
>CbowOR36
MYFFYVSFLYGTGVIFFVCEFMIFNETIGKISKFVSHIGMLFTHVVGILKMSILIFGRYRILKIMNVLQNEKYHYAPLEDSQPGLLVV
KEKFVSSGISILVFVLYTFVGVSAHISSLITINEEIKGDSFEGTNKTCHDYMPYFFYIPFPTETKGQCGIAFAFMDVGLGIFAWVIACH
DGVFVGLLNCLKTQLLIVCNIFTTIRARSLKAVNLPKNYKILHDEYNPALEKELYRQLSHCTEHLKLLLVVRDDLEIMFTFVTLSQ
TLASLLIFASCLYVASTVPMTSPEFFAQMEYFLCVLVQLSLICWFGNEITRASELIRLSLYESDWLSCSRRFKSSMILTMIRMQRPVY
LSIGKFSPLTLATLVAVCRGSFSYFALFKSVQ
>CbowOR37
MYFNTIQKVLPILYIIGADPREGFTKSQFFLYFYNIFSAIGMVYLLVLKFANAENKVTVKDITDAVICLFLFCHGMVKSTTMFVKK
NSVQTLLAQMEKHFWPMNNYKYSYIHNGILNICKTIRNTTNFIWFMHFCNAMGFLVGPLITKDPVLPFECYRPEWMGYYTLLLF
EDVTSIITILCPVLAMDVFFVTIIKLTQIQWKMLNSEIQSMFDLSPSGKISREDEENIMVMKIKKCVVHHNFLLNYQQLLNDTFSIPL
FFFLIVIVLCMCVEMYVISTVSDWESLRTAIVYTATGCLEFMLCYCYPCQDLSDEADNISYSIYFSNWYRNPEYFRDTQLIMQKGQ

KLVAIRPGGFMIMDLKTGLSVGIFPSP
>CbowOR38
MFAGCIFTCTFWAICPFTEDVASLPIAAWIPFKTDSSPSFELAFAYEIIATVIGGITDLNADCFMAGFIMVVCAQMKILNDSLLNLRH
FAVEELNAETGGNNDDDGIAEELQKIMNRKLVECVHHHRYILEFAEEANSLFTTSILGQFAVSVIIICTTLFEMTLVPFASIKFISLILY
QYCMLMEIFIVCYFGNEVILESSKLTKYAYHSDWRDCSQEFKRNLLFFMTRSQRALKLYAGGFFTLSLDTYVKILKSSWSYFVVLI
QVNKDSG
>CbowOR40
MYEKEFRNVFWLLNFVGMHPLKKYVTPFIVFNAILTFYITVLIILKLLWDKELVAVESLCVFSQIWLKIFVLTTKKRKIKQVIDDTQ
QFWENDPENSENKQLLKNLAKLERIFLTYISCSTCMFLFKPLLVKGTSIYYYYKIPQIPFYVSYPIEFYVTIVTMALAIAVNLFISIVI
VIGAGQFSNLNAKMKQLDLSIAEDCQDGLRTCTLEMNKNIEYHDFLIKYVSHLDEIFSMLFVVLTGIITSLLCMNMYVLSQPTTTA
VDMIRCGTMVCAFTSEFLFLYGVPAQRLMDEAEEVANSAFYHCQWYLPNIIPLRKSLSFMIHRSQKSVCLSAMGFIDINRQTIVA
MIKTAYSFFTFLQTIETTGGA
>AquaOrco
MMKFKVSGLVADLMPNIRLIQASGHFMFNYHADNSGALHALRLGYSCLHLVLCLVQFGCTFGNLVIERNDVNDLAANTITVLFF
THCITKFVYFAVRSKLFYRTLGIWNKANSHPLFLESNNRYHALSLKKMRTLLICVMTTTILSASAWTAITFVGDSVHNVKDPDND
NETITEEIPRLLIKSWYPWNAMSGTAYYVSVSFQIYYVFFSLAHSNLMDSLFCSWLIFACEQLQHLKEIMKPLMELSASLDTYVPK
SADLFRAPSANSQDNLIENEYNEKNEGLNLKGVYNTRQEMGANFRSGALQTFGQGGGGVGPNGLSKKQELMVRSAIKYWVER
HKHVVRLVTAIGDAYGVALLLHMLTATVMLTLLAYQATKIDGVNKYAATVIGYLVYSLAQVFHFCIFGNRLIEESSSVMEAAYSC
HWYDGSEEAKTFVQIVCQQCQKALSISGAKFFTISLDLFASVLGAVVTYFMVLVQLK
>AquaOR1
MDDLKNFTACGYVIAIACMANVRSFYFLKNRKELLHLIDSLDDAQFSPVNKEQYRMARKSLLFYQTVKRFARNTEILFNRIYLG
QFIACTSALCMALFLLTLQQESNFECLFLVFYLASIFSLLLIPCWFSSEMCRKSENIANAAYSCHWITASKTFKKDLTFFIHRSQTPIR
FYAVGIFHISVETFMSIVRSSFSFYTVLNNLALEEE
>AquaOR2
MDASFSILGCYLASYDTTFTSILICLKVKIQILNEAIKSIRERALSKLNQHSESDLELVDPKLEKILYGKIVHCAKHLDSLLSVCVET
ENIFKYVTLLQMIDSLLVMASCLFVASLISQSDPDFIAMAQYMISVLTQLLTICYFGNEITEVSSTLNSSLYQSNWLNCSKRYKQCIL
IMMCRMQKKLCMTIGNFSPLTLNTFLAVVKGSFSYCAVFQRVNNE
>AquaOR3
MFKESIKKISTFASHIGMVITHFAGIVKLCLLTFGHGKILRIMQVLQDKKYRYESLGESRPGYMMQKEKKVNNVTTYSTIILYTLV
GISGHISSLINLNREIEGDSFEGTNKTCYDFLPYMFYIPLPSSKKWQCKLVLSFMDIGFAISAFVIAAHDGIFTGLLNQLKTQLLIVC
DVFKTIRSRSLKNTGLSEDYLITRDVDNPELENELYKLLTHATEHLNILLGVRNDLEFIFTYVILIQALASLFILSSCLYVASTVPIGSP
EMIAQFEYFFCIFLQLALICWFGNEITRASELIRFSLFQSDWLSSSPRFKQAMILTFIRMQRPVYLSIGKFSPLTLATLVAVCRGSFSY
>AquaOR4
MSDSENNLGYPSNFFHTNEVIRKISGIWLPGSEYHLALRGLYFLYVSFLYGTGLALFICEFLIFHETITGISKFVSHIGMLFTHVVGIL
KMSILVFGRKKLQKIMDVLQDKKYFYSPSGDSHPGSLVVGEKFISSGFSILVFVLYTFVGVSAHISSLITINKEVKGDSFSETNKTC
YDYMPYYLHIPFSTETKGQCGVAFAFMDVGLGIFAWVIACHDGVFFGLLNCLKTQLLIVCNVFKTIRVRSLEAVNLPKNYTVLQD
THNPLLELELYRQLTHCTEHLRILLKVRDDLENIFTFVTLSQTLASLLIFASCLYVASTVPMTSPEFFAQMEYFICVLVQFSLICWFG
NEITSASELIKLSLYESDWLSSSPRFKSSMILTMIRMQRPVYLSIGKFTPLTLTTLVAVCRGSFSYFALFKSVQ
>AquaOR6
MEAHDINFRNIIIVNFNFLYFFGIMYPEFDTFGSVVIYIIRILILLGFFFAGIVTCEVINWYFSLGDLEATVNASFLTLSNIVSIAKFYVI
ARHQEKILKLAELINRKEFKPKSEEQRLILKNYIKTSKVISGLTYCGCVFTCAFWAIYPFTEDGDAFLPIAAWVPFRTDSSPYFEIAF
VYEIIATVIGGLTDLSADCLIAGFIMVICAQLKILNDSLSNIRKFSLEEMKIELGENEDKISPKLQDTMNKKLLECVIHHRYILEFAGE
VTFLFTTSILGQFAVSAIIICTTLFEMTLVPFTSVKFLSLILYQYCMLMEIFIVCYFGNEVILESSKLTNFAYHSDWQDCSQEFKRNLIF
FMTRSQR

>AquaOR7
MKPPRRSYLAFTIEILELVNMWTEKRGFLSLLREYYAMIIIVGSTVAIMTDFFLQFYDEQSHFTSLIESLIGGSALCSVIYVSICFLLK
KEQIKRLVASLDIFEEYLPEKGIEEAEESARFYTKSFLFYGIVGNGLYEASPFMSFRECNEERTENMIKMGIPCKVIVRYVLPFKYD
TSPFYELVILEQVTVAILGTIVVMTISMLVCGILTHIAANLKHLKKMIKFISQIEEHKLKEHVNLCIKYHTVILEVSDKTNEAFSGM
MLIHITWTSFIISVLGFGI
>AquaOR8
MSIQGESSVSPDLYKIGKNKPFSATLDTLTSCFMYPKNLQKCYMVRFYTFAAILIVASFVFCCFLSLLHLIMAKVDGKNTDISEDVS
LITAGTGLVFANAMFCYRCRGWSAVMSRIARLCQLEDSPKTETTIKRCNKLSKMFTGYCFLSTIIYGTAAYYESFNCVKINEEKGL
DEICRTFMTVWLPFKLSVLGEFVLFGLQAAFAIAVVVPAVTVFFVNWESAELISLCCDHLKDSSLSILEEMNIDRRHEKLKRWIFH
HQQILGVIAQFNKEMRFSVGHLSLIAALVFACLINQATNYKVCGALFELLGWLISLFLICDAGQKISDSTLSISEAIYEMEWYSTDT
QTMKYLLVILMRSQRPMVLDALPLGTLNYTLYLMMLKASYSFWTLLSHTT
>AquaOR9
MAKIINYTKFFSINMFVFRVLGFWTPDHNMRYKQLYNLYTILCTISWFLFLSSQATYLITSLNGIGELTIVFFTAVTFGANFIKTMAI
YRKNDVIKSWMKNLHQPLLQPKCKKHHKMAQSTERFHIKLFYICLYLGVQTYLFFSALPFLQEETIMLSQGWFPFDWRKSPNYE
IIYIFQNSVTLWNTIMCLNLDTFSAGLLSQIGLQCDYLIVTLSSLDEFSIENGSLRESDESSIHLSRRDPEIFSSKMTHNLIICIEHYQKI
RRLSKEIEKIHETSIFFLFSGGGLIICSGLFQLTVVRIGSIQFLMVVSFLMSMLTEQFLYCWFGNEIIYKSTEISNAAYNTPWLDCDIR
YKKILLNFMIQTKNPIEIMVGGLFSMSIHAFKSVVQSSYSGFALLKKLQDKRA
>AquaOR10
MQEYVDFTKYFSFHISTFKLLGFWKPNEDLRHKTLYNVYTFFCTAIWLSFVLSQLIYVFTSFTNTKEMAAILYVAGTVSLDLIKML
AIYSNMDLIKHLLKELNNQFFQPKCKEHLDLARNVKKFHTSLFYFCLYFGLQTYLCFSFVPFLYDETVTLTPGWFPIDWSKPFNY
GIVYAYQNIVILWNALIFLNLDTFSSGLLMQVGLQCDFLSITLSNIENFHVCDGVLHENDVSVFDWSEYSPGEFSDQMLENLMICI
EHYQKIKTLSKEIEDIHHTSVFLLFLGGAMIICAGLFQLSLVSISIHMIIRMAVPRFHNLFGLNLLNIQS
>AquaOR11
MPRMILRKVRCRNTKEPMFLPFDTSLDGYYRLEYFFSFYTHLGNVLLFLPIETTLTCSIIHLISQTAILKEAFGYIDKGVSDEQESKS
IAIIKEFRIVKCINELQQIYRAVQKLEDFCNIQLMIQYGFATFLLCTICHVIPLMNNMVEGVSNLIFVSLSLGQIFVFSYCCQTLCLDL
QGICIPIYNLQWIDYPLKVRRSLAFLIRRLQKPANLTAGKMVIIDLLFFIQVIQKSYSFYTLITNTNKRK
>AquaOR14
MSCFITLLLFSSMPIFTQRDLPMPFSFDIGKFKPAMYVFQIMGMLTTSVNNSSLDLLAISSMGICAAQIQILNRKIIGLSQWKENEG
NTLHNVNSDLCKCVKHHVEIIRFNGIVEKVFSYISFAQYAASAITICNTGFQLVHVQPTSLKFMIMVFFLTVLMIQLVMYCWCGDE
IIVKSLDTTNACYNFKWYESDLKTRKTLIIIMERSKRPLFLTAGKLSILSLQSFTSVIRTSYSYFTVMQTLYTDQN
>AquaOR15
MKIVGIYPYDNWPMMYKIYAQFSYIFFTAPTPILAAMSFIVSTEKDMEKICDNAFLVAQLGILLVKLWPFKNNPEAVKRTVNGLNR
EIFNSYRPDQEYIIRGVIREYNFIFFGTAFASLASLFTWFGKAFFYENRRFPLDIWLPFEPFEDTVVYVAILVYLFFSVLSGALDNISL
DTLIVGMIFQSAAQVKILKNNLQLLNERVEQDVNSNYNSNGPPRNDFKAKLVYENICKCIDHYDAIYEYTKEVETVYSLVVFTQL
FASIVVICISCLQLSIVQPFSFSFFGMVTYIITMLIQLFLYCYYGAILYEESNTLSTAIYGSEWYNYDQKSKKCLVILMERAKRPLKQT
AGKFFDLSLDTFTMILRRSYSLLAVMKNY
>AquaOR17
MVLILVKSTNIQNSIDFILNYEKTQLKNEDIGIRHLYLKHANLNNRMTILIFMLISSVATFWYITGIRKTFSAEESEECPLMKGVLYQI
WYPMIIRKYYWLVLINDLGLLFNALNITVYCKVMVVSMMVFMLSQIKILQFQLTTIGKKSLEQSMKKDSTEIAAIIGCCRRHQQIL
KLMDLVKSAVKEIILIQYFGSTSEIAAYLIQTLTSRNVSDILRNCAALSMLITEVFIVFWFANETKIQSEVISDIVYDGMPWYLYEKD
ANMILLLMMRRSHTPMSFKAIFLGDISLATFTKLMKLCYSVVMCFSSLIDI
>AquaOR18
MMSTYFLKYTKRCMLLMGIMKPEFSTIYLTHIYRTWGLFIVCCYFLFCVSFTIGMRSVPKDLLLKNDELTYCWTFAMIIVKLAFS
QTDTMRNVIDEMFKLEDGIDKNDSGAKKKYLMHSRYNFKIFYVLASIYFGTLCQLFWLMKYNWAQKSLFFTAWFPFDRTYYYT

FTFLFQIVNGIIITFFTLTFDTLFTAIIIFPSMLLDILGHKFEHFEDYCKRELHPKLVLRKLILEHKAVIRYVEKLDHSLKWFFFIDFLVK
SYHLSVMIITLATAADEMDVNFAFTMSKFAYWSLEAWCLYYHGNELIVKSLAITNRIFGSNWYEHDLVVKKMFLIIMLRSKKPLK
LQVGVFQTLSNDLMVKFFKAGYSVMAWSDKLRG
>AquaOR20
MNIDTIKEIAVLKNSMYFLRIPFLFPQKDEINDPNRNVYFKFILLSLTTIYLSVGAAIHLVLRIQNGTYVNLDRDIGTIISYHGALYFT
FRYLGNIKDIITLYKQFSDFKTYGTPKNFERKNKLLNKYSRWYFAYHMCVVTGMTTSALLTVDKCEAENIEKNLNEVCGLIGPT
WLPFEFDYFPLKFIVYGYQVYCSFVIYQTAGVLSYTMMETVEHLIIRFEHVGDTFKEALAEENLSKRREKFYVAVQYHKDVIKM
GKLLNSCFGPCMMVHISLTGPVLGVAGYRFVTEIALDSTSLFFGWMFSTFIVCRGGQRLSEASVAVGDVIYTVEWYNLESDLQKD
LKMVMMRCQKPVLLRAGPFGPMTFSTIVAILKTSYSYITLLKQTM
>AquaOR22
MKMFEKEFRNVFRLLSFVGMHPMKKFVKPLVIFNSVLTFYVTVLITLKLFLGRELVAVESLGVFSQIWLKFFILTTKRAKIKQVIED
TQLFWKNDPPNSQNQQQLKYLAKFERIFLTYICCSTCMFLFKPLLVKGTTIYYYYRIPRIPFYVSYAIEFYVTLVTMSMVIAVNLFI
AIVIVLGAGQFSNLNANMKQLDLSRAEGNDEGLRSCLLEINKNVEYHDFLIRYVRRLDDIFSMLFVVLIGIITALLCMNMYVLSLP
HTTVVDIIRCGTMVCAFTIEFLLLYGVPAQRLMDEAEEVANSAFHHCQWYLPNIIPARRSLTFIIHRSQKSVCLSAMGFIDINRQTIL
AMLKTAYSFFTFLQTVESTGEAKK
>AquaOR23
MIDISESMISIFSFSHAAMKLINLYLKRSVLLDVLDSMRDNFWKLEDIEDHDGTNIYLTFTKDLKRKFHLFLTMTTICVAYYFVAIL
LTNKGRTGDNLPFESYIPPNVSYEVLFSLQFFAAILMIIPFFTTDVLVLTIITLTSIQFKLLNVVIAKMFEGMTDSDEDAHIINMRLKK
YCDYHSFLLIFRKKLNNMLSAGTMGYMALTILTQCFVMYVVCSQGSQKESIKAILYATTLFFQLFMCYCIPAQNLADEVEKLPDV
IYGSNWNQYPNHSKDILLFLGKSQLKMSISAGGIADINLQTGFSAMKTVVSYFMFLRTISDK
>AquaOR24
MPSNLLMKYINQCCTILKWLGVHPEKVQPWYFFFTFLNIVMIFLVILVLFYKDHAITFTDIIDTFSNCALMLHGIARLLNFYLKRAS
LLDLIQGVQHRFWKLEGLTTQERTHYYEMIRVTKLKFYLLAFSAVSKSIVNSTMALFSDNDMVIINCYRFEAISPYVTWLYQTIFT
HFCIHLPVISMDSLIMIFISLTQMQYEMLNREIERVFRRAVRGDRYDSNIAIGRIIDHHNFLIEYTNRINDTFSNVLLAYLFLYGSSMC
LEVYNSSEVSSLEASMNALTYLIAATFGFVFLFCIPAQNLTNEANKTANAVYFSDWYHETKHSTAIMMMIANGQREVSIMAGRVV
NINLATGLATFKSVVSYFMFLRTVTTVE
>AquaOR26
MICALTMVTLLILKFTNKHESERVTVIKVTDGVTCLFLFCHGMLKSTTMFVKKHKVQELLNKMEEHFWKQDHFETSTIYTEVLR
IYRVTTNTYNFIFTLHFLNAMGFLIGPLLTTDTILPFDCYRPEWSGYYVLLLFEDLTSVISILCPVLSMDLFFMSVIRLTQIQWNLLN
KESISMFDRVHARDGIEEDTEAKIRKCIHHHTFLLHYGDLINETFSTSLFLYMVIIVLSMCVEMYVATTMSEWQTLRKALVYTATG
CIEFMLCYCYPCQNLMDEAAKISSSIYFSKWYKYPEYSKAAQMILLRGQNMVIIRAGRFITMDLKTGLATLKTMVSYSMFLRTM
SSVDN
>AquaOR27
MTLKKDLFFFLIWPVGAWKLRACHIAVVIFTGTLQSLQIYYISQVGLQDPCVLIIFFLHCYGIICSLSCIIFEKQMEKWVVRILNAFL
SIELMSESAKEEMKKLIRYGRIYKAVVGLLMLFISCSVTPRYHLCVVIFKEYFPDVATILIVVYFLDAYFIAYAISALSFIILYCLFHVK
LQEKIVSDAIDRICDGFEIHVDLNDDLEYQKIVEARLHSCFRRIVWIRRSISMLRKTLKNDIFTHTICGMLAASSIIILFSVTAEKKRY
HRDSHDFFAIGSWIYHFWNVIER
>AquaOR29
MLLFMKLKPALPKWLELITNVLGYRKLCSNKLKRRIEHECGVRRLLIYTSIIAPIFVCLPYGWRGADGQHFFLLFIIEKFVKGIGIT
VIFVGCFLYPVFVATVSFNASIYGCTHIYLQLLILADNLEKLNVRYTENENYQLHNDQYQKIIRRKLILCWEQYLNIRRLKALAND
TSYWPIYSGYMHAILIMGTSCIYFLLGGHPSENPGIVLTPIFVWVQILLFLEHGQYIKNESENIHIALGKTQWYQWNTENRKFLLIF
LIATEEPMQIESFCCTQSREIYVKVFTGLYSIITCINTLRNRT
>AquaOR30
MLNLRMTFLMFAVIFIVNTCVPFPKICEPFHNMAQEYFHDWTFVIDMIVFFIFIPFTGYGLACGVWKLTFFYKHGDIQFFIVNALL

NSLTTEANKNLSDDEYQRIVYEKLIHIVIAHQNAKKFNRMMVNRFGWILHPTSIYASLGAVSLIYLSVSEYHTIGNSPYWGTISTF
VVLGCVVSVLIDGGQKLFDESESVFFNAYKCPWYTWNENNKRVLLFLMTNSVEPIRVSSYGIVNLDRSTAVKIVRMGWSLMAV
M
>AquaOR31
MLQWLLGQSLTLSGGIEIYWNGNPNMYRKQTPYLVLIQTVTMMILVSQITYREYSKIIKKSSWSWTMAGDETFRKLSSYSRHSK
GVCCTIAGLVIISTALLIPVFGDETEWQFTAYIWKKYLDDRMHILFTIFTGTLSISSGVFISSLICLRLYCNRHVECQFHLLNALICRL
HTKALYHVNDVRYQERVDLQLKYCIRVHVEIIKLFKLLMRYANSILLSMTIFGLSIFVSSTYFIVTDLSPESNFRMYFLLMTACVIL
WAFCESCQRITDESAMIFHNACKCPWEQFDKQNRRTLLMLLQNSATPTRVSCHGVYDANYQLVAKMWRVCYTSHTLFTSLD
>AquaOR32
MNAYQQLKSGLEEDPLQFVFRSSFLTWFSVKNTKRILYFKWFSAQLLAVLGGIEIYNGSPDGYWRYTPYLIMAEANALVFLMGA
SNCDQCQDYSRLIKMQAWSWKLADDETYKELLSQGNRLKVLCCAMTCITITSLVVSVPIIGDEADWQITTLMSKKYLDDWMYT
TYIILFSAEMLILGIYLASLFSVCIYCTCHMKFQLQVLNSIVRRLHTTSVFHVDDVRYQERVNVQLKCCVKVNVGILRLIRNITERT
SPIVLAMAGYGLYVYIFSAYFLMAELSPRSNFRIYMYTIDTCIVLWAFCEYCQQFTNESEEVFFNACKCPWEQFDNRNRRTLWML
LLNNCNPLTLSCYGVLEANRKLVVTMWRMAYSVCTLFTNFN
>AquaOR33
MERAKRPMLLTTGKLLDLSLTTWTMVFVITLLALSSGADKTSKPKPKKRQSLLFDSGNQFSIDSSFAQPSITPAISFESSGGLVSHD
LGTTFGSGFGSVGSGFGSVGSGLGAASFGIDSLRSGIGSFPSSIGINGLASTGSYGIPYQTIPVGGFGIYAGAYNVPFHANYAYGVPA
FFQNGNYGYGQFGNGLNYGSGYIQPIQNIRTINQHVPVPVPQAVPVTVTKTVPVPTPYKVSVPKPYAVPVKVKVPVTVPKPYAVK
VDQKVPVPVAVRVPVEIPTPYIVYKTKKVEVESETPVFVKDPVNVKVPVPQPYPFEVRTGVPVKVPHPITVKVPQLVGGQWIGQG
SFGTVENGFPAQTLPAFGGSFDDLSGLYGFGNPGGIYGTNGIYGTNGIYGTDGIYGINGIY
>PstrOR1
MMKFKVAGLVADLMPNIRLIQASGHFMFNYHADNSGSLHTLRVAYSCMHLVFCLVQYGCTFGNLLLERGDVNDLAANTITVLFF
SHCITKFVYFALRSKLFYRTLGVWNQSNSHPLFVESNNRYHALALKKMRTLLICVFTTTVLSAAAWTGITFVGDSVHNIKDPENE
NLTIVEEIPRLLVKSWYPWNAMSGGAYYITLVFQVYYVLFSLMHANLLDSLFCSWLIFACEQLQHLKEIMKPLMELSASLDTYVP
KSADLFRASSANSQDNLIENDYNQKNEELNLKGVYNTRQEMGANFRSGALQNFGQGGGGVGPNGLTKKQELMVRSAIKYWVE
RHKHVVRLVTAIGDAYGVALLLHMLTSTVMLTLLAYQATKINGVDTYAATVIGYLVYALAQVFHFCIFGNRLIEESSSVMEAAYS
CHWYDGSEEAKTFVQIVCQQCQKAMSISGAKFFTISLDLFASVLGATVTYFMVLVQLK
>PstrOR2
MVFIFQNIVVFWNDIICLNLDTFTSGLLMQIGMQCDFLSHSLNNLGAYRVRNGILVEQNNIAETSLCRKTFSEIVMENLVVCIQHH
KKIKRTANEIEKLHKKTVFILFLGGGMIICSGLFQLSTAKLGSIQSFMLISYTACMLIEQFMYCWFGNEVIDKSSKIFNSAYNIPWIQ
CDMKVKRTLLQFMTLTKYPIKLKVGGMIIMSNAVFVSVVKSSYSIFTLLKKIQK
>PstrOR3
MSGDRVTFDFRKFFERDFFMLKFLGIYYLTENAKLGQILYSFIVSLPLMVVATMVQIINMFCVEADLRKMAASGYLGASCLMAVI
KELFLFKNKKFLLNIQNDMNRSEFQPKDAEGVELVERCLSGYRKTRKVVLIVCSIAVSGCLTMPFLSSTQLLPLSAWYPFDVTYSP
VYELIYIHQSVSLIYLAYMNIYIDILVSGFTTFVSLQCALLCHKMESQRNINSVKEFVIHHKWILNFVFNIEKLITEMYFLQFSACTV
IFCLSWFLLTMIDVNSFEFVYLFAYQTAIGSLLLIPCWYSSEMERQSQLIPFSVYSLPWRTNTHQFKKDVYFFLQGVQKPIFINIVGL
FPLSVDTFTKILRSSFSYYTVLNNMNLKSQANN
>PstrOR4
LMMEIYVLSSDLKFNIFLDILVVLIIATGHLVKIVIFKVKTKVWLDIFDQLKSPCFNDYPEEFEYNNKRHVNFSKNFGNLFQFSCC
WSTGMMIFQPLFSNVDLPMNCSFVTEPFIPYMYAGQVMFILLCAFSHSSIDVLITDLIGIIVGQLDVLGEKIKAYDTNKTKEKIDLK
QCVQHHVEIIKFVNNLRQATSVIILIEYVKSALVVCFLGVQAVHIDPMSLTFLKMPVYLIVMLFQLLIYCFFANKIIDKTQEIGDAC
YFSMWYFYKNTCDKRSLILIMERSKRPLYLT
>PstrOR8
MPDSYPEDFFVVNRWILKFAGMWRPDTDNGIIQALYTTYVIGIFLFVNIFFTSTEFLSLLYTYGNEYDLIKNISFALTHLMGAVKVV

FFYYQGRSLKSIMSTLESDELRYESCEKSGFFPGLVAKSYKRTGIKYTVIFFMMAHATLTSSYLPPTLAALKYNEDDPESLLPDRLP
YYSWMPFGFDTAGTYLIALGYQAIPMFSYAYSIVGMDTLFMNIMNCVGMNLEIIQGAFLSIRDRSLEKIDGAPLTADGLYNTTALN
GVMRAEMRKISRHLQTVYRMCERLEDVHKFLTLAQTVATLFIICSCLYLVSSTPIGSKAFLAEIVYLIAMGFQLTLYCWFGNEVTL
KANEMPFYIWQCDWLTADDDFKKSMIMSMVRSKKPVYLTAGKFAPLTLPTFVSVSITHEKEHSLVKFIFTDTEGFVLVLRSHQKH
QRVESSNQTFFTV
>PstrOR9
MGEMRDINFKEIVSVQVFMLEGFSYMLPQYYTIPKVAYFCAAFLIYIVGFFYSQLACEFFKIYFNAENVTDVLSDSFLFLTHLVQT
TKLGYMYYYRKRLWDLIESLNQPAFRPVSLAQRDTLGRYIGMAKFISYKFQILCLLTCMFWTFYPFTLEELMLPLDSWYPFNTTY
NPNFGIAYFHTSVGSWLNGTSNIAADTLFAGLIMAACAQLEILRDTLTNLRDYAKARLGGLGENSGKSGGIPAILMDEMSALLVD
CVNHHRCILNFVREFQFIFSYAILAQFVVSVIIICTTMYNLTLIPFGSMQSISLIIYQYCILLEIFLWCYFGNEVMIQSNLLCDAAYKC
DWTDCSPTFKKHLLYFMTRSQMEMNIYAGSFFTLSLTTFVKIVKSSWSYFAVLISMNK
>PstrOR10
MLPETRSKHGYPADFFEANELIRKITGMWLPTKHHSIITKTFYFSYVFCLYGFGFYFLVCEIIIVNDVSTEISKLVSYIGMLFTHLVG
CLKFSILVFGRTKLQKIMNILQDSRYFYEPNENFSPGLFLVEGKKTSAQFSMLVLIMYSCVGASAHISSLVTFDRIIEGDSLEGTNFT
CQDFMQYYFKIPFETNTKERCKLAFFFMDFGLLVFALVIACYDGVLVTILNCLKCQILVVCNVFKTLRSRCLSQLGLPVWYETFS
DHEHVALEREMYRQLSHTTRHLQALLSVTNDIEFMFTFVTLAQTLASLLIFASCLYVASSVSMTSPEFFAQMEYFSCVLVQFLLIC
WYGNEITTSGELIRSSLYESDWYSSSMRFKNSMIITMMRMQRPLYLSIGKFSPLTLVTFVGVCRGSFSYFALFKSVQ
>PstrOR11
MTSEGIVPECKHLKFALPYIIVGGVWPNIFNSSKFWEIVYKAFSHFMFYASVCIVLGCAGQFFVLLQDRPLKIEELLSGFSVTTIWT
MAIARANGLRKPVFKKLIQSIIDSEKRIFFSGDKDFINIQKSYISKNNFICGIYSTTLSLYTYAFSLKFMFINSEVVDPLLKETVIRQPH
TIQIWLPFDKDLHYRSAISLEGFLTVMITYLLIGSEVCSYSIQTYVVSQLKMLNHILMNMDKFELRVMKQIGCGEEEAMFIVLRETI
LFHQFLLGYIEDYNKSMDIVALIDFILLSAQLAACVLPEVIGLPPVIGISYLFLGLIILSKFFVYYWYAEEITSEADKVAESIYNSNWT
EKPLKIKYMFIFVMMRSMKMTGLSIGPLGVMSMRVYLMIIRSTYSFVALVHSFF
>PstrOR13
MENDIKLASMSFSIKVFQFLGGFPKDQEFTNPSKVFYFKYVMRAVLTSVLLVSSSLHLTQNIIENIFNHIELDIAYTTAMFTGYSLM
FSFLLKMDLIVRLYKELSNFEEYEKPMDFEETNKKYNKYSLMHLIYLQIIIMMSTLGSNIFKVDTCRQENVEKGIHEVCGLYLYT
WLPFDIDFTPVRQIYLIWQLVSAQYIYTFIGVCSWMVFESVEHVNIRFKHSAHLYSLVPEEEHAQKRRLLFNKAVRYHQYSIRLAE
WIAETYSVFMFAHMFMTSVILGYDLYAFLKTYDVSTFIMSIAWVNGVFMVSHGGQRLQDMSRQVGDEVYASNWTDYDISMQK
EVSFVIMRCQKHMSLKAIFIGDVGYMPFLSVLKAAYSYVMLMTNTGS
>PstrOR14
MTSEGILPKCKHLKSALPYIILGGVWPDIFNNLSLFWKIVYKMYSLLIFYSSVGVTLCSFAQFYILLQDRPLLITAELLTSFSLANLW
SMASVRAYGIRKPIIKKLLKGVIDSEKRIYIGDDKDFIDIQNTYISKNNTICFIYSNCLSIYLVFYFLTFYFVTYKMSDPALLQTDVIK
KPHIIEMWFPIDRDKYYATTIFIEGIVTVTVTYLVVGVDCYSFSVQTYIVSQLKMLNHIFENLEKFELRVKTQLGCDENEAKFLVLR
ESIIFHQYILRYIDDYNSTMDIVALTEFILLSMQLATCVLPQVLGLSPIIGVFFLLLGVIISSKFFIYYWFAEEITSGANDVTDSIYNSN
WTEKPQKIKYMFILVMMRSMKMTGLSIGPLGVMSMRVYLMIIRSTYSFVALVHSFF
>PstrOR15
MHYPIEKGKPYYSTLLSMKFLGVYPKSSSEASLKASMVTTVCTIIFAVVVICVGGMGHLIASFKGSKSVEMSEDLAVAVGSMAFL
LCALFFKLNWRNWANFFHSITDFKTYGKPEDFDSVTIRCNFLSMMYSIYISGGLCVYAVISIFETKCVRDEQNNFCGTLTQIRLPIE
GEISTLAINIIFLCQLSLCIWACVESGNVFFLSHESSEFIICHITALKKHIIGIFNYEDEMSKREQLLHCIRYHKQIIEWGYDLNRLTKS
TLGHMSLIAAIQVGMIGNQILHKYKMFGAAIYLAGYIMAIFLVSHAGQRLSDESISIAKAIFDSDWTKASTGMRKDLTFMLARSQI
PLFISCLPLGNFNYALFVTMLKASYSYLTLLKQSTSNKDD
>PstrOR17
MKHLQIAKWIMIVTGFWTFKKEYFTKQQHLFYRAYGIIMQLYFSCFIGALCVGLKAVQDNIQLIDAIGLLIFCSVMLIKIIICQRKS
VRSIMNRIVDFDDESDTLTGKFRDIYLKNSRYNVIYGLSLFLVSAMCGVTLVFCDVAIYSYDMKRVKSGDLNSSDVRRPLPYHIW

RPIDEKKHYYWAFLLDVLTATIGCTYNTATQIVYLSILTFILGQIKILQVKFEQMGAVCSRLGSEQERFMYLRSLIVEHQNIISFVKDLDENMKYLLFVEFTINPLQITCNLYEVLVFKVDGMFLFRVVLLLCMLIQMFTLTWHSNEIQVLGMAISQSVYDGEWYDLSEKFKQRLLIVMMRAQRPLTLAVGPFFILTNSTAVTSVKAAYSYLALLNNKRGD

>PstrOR20

MLLLSAFLQVLLRDCSFSDLLDSCIFVVNETTFLFKLLQIHQKAELLEKLRLYLQDIRATDVSKRMMENLLVRQSKRNKIALIYRSLLCLFVVEHFLMLFIQTERKFLPIMTWIPYDYSKFSLYYPTFSFQIVVVSISALSDISLDCIYYALIDVVCCQLDILIYYFKALNFSKNRDFIKTKLKMNIVYHQKVIDFVKDIEKLYSNIIFSELLKSLIEICFIGFRLTVLDVSSMEFFMQLFYFMGMFCEILCYCWFGEILTLKSKEIGDACYMTPWNEFDKELKNMLLIMITRSQVPLVIRAGFMKLSLRTLTGVLKSSYSYAAVLKTLYK

>PstrOR21

MTNQKLLRKSFWQIEFLLKLNGLLPGERYQILYSFYTSCLLFVFKIVFPILGYTFIYKADPSERMILIGNSFVYIEVLVLVFKHWPFITNPDLTKRLLNQWNESIFNTEVEIDKHIIEESMRQRKIKHNVYFYSATSAPFVMLINVILSDHSDLKLPIWMPVDVLNSTSWYVCTNAFVFSAYLHGVLGHFCVDMLIVSYMLYCAAQLKLIKYKLENMEQYLDTDLTTPDQIGLDDLVQKKIYAQITQCVKLYDAVFSYVQELENVYSIGVFAQFLVASIVLSICLYNMSQLESLEMNLAYVMSFFVPMTYLLYLYCNLGTLVMEESTTIGDAILRSQWYNYDINSKRSLMILIERSKRPIRFTAGKLIDLSLDTFVLMVKRAYSLLALLKNFN

>PstrOR23

MILYQDFLEKKETTSLFITETMSYVSYITVPFVTSLSLLFKKNHWVEFMLKMKEMRVKMKLKSKRAYFSKKVLLVVFFLHLIECANIGTHLTLDSKDVLLTTYFYLYETGLLYIILLFFSINRVLQVSYKDLDDKFKEINRIGSEHSFCTDNSNARIIAKLRECFYIYVDLHGFVKDVNCLFGWIVMVIFIYALTNSVDILNWYVNGADILNADLLDLIVTIVSTSGYALFKN

>PstrOR24

MIMDEIAYKIMKTKYYGKMVIWLPKILLGLVVMWPKAKVSLMRRFIVTSGSIFIISFTAIGLLNTLNFTFESINHNLIGSLLIMSCFQGVSKIILMSIKFKDIADIIDNISLKFWPDDLTGYVEVDKEIKKFYIFTMSTCLVFLGTVAVFFSIFFITPLLIEERILPFNVEYSFDWKVSPNYELIYLSQVFYVHILALYVIGTDFFYLCTVVSVIIQFKILQQCLSLLNSNEMVEIMNKLDINSIIKSDKMDDLCKEYLKKCVKHHLLLLRFIKKLNAVFNLIILTEWSTVMLSMCLLIYMLVQNYAVNTFFETALALSMVIAYLQQLAFYCIVGSIFNYQINLLPEHLFGSKWIVINNEIKKDLTFVLQHSQQNLTLNVYNIYELNMVSYLQVLKLAFSVYTLFSNVSN

>PstrOR26

MDLVYPQPDQHRVTDDPLGRMRSCLTLHGFQGKRKYFWHLAGILKAAILFGRTIYACQTLNQPKKLAELVATYPIRFLAVVKISVLFIDRKRVKYFYDSVSRDFWNFDVAGPEIEKQVKRRFHWVNLTALCQFSIAIVALIIFIMFPLVEMPEGKRTLPNIIWTPFDTNPSPVYEIMYVVLLWNLFMSVLGNAFYDATFIYSTQHLFVQFTLLKELIRNVSSGIMSESSDLERFDSEHFQKAVNERLKICIEHHVKLLKYGKNIEEFSTTVMVPQLIMSYAALVINGYIISVDHADVAKTMGLINLTCGSVVQLVLYSLLASDIKAQSISVIDEIIKTDWYLFKAPIKRALIFMMMNVKDGIVITAGGMANVDNEVFVAVLINNLDVYEFIAVEFSRWFLKQYQPLLY

>PstrOR27

MQGNNFLKYPILMSKATGQWQFEHTSALVSIYNKFSDFLLIIFIFCTHYIVLALPFYYKCEKALIEIIFYYVHFIIAIVLTVLLKSKSIRSFLRWIIRYEKSTRFEHQQEIEIYQYYCKMNNNITVFFVIVINIVSWMWYALNKRVSPSEELGPHCPDIRGYVFKIWYPFEIEDFPWICATFDFVTVFCGMMLFCYYKIMPMSLIIFLMCQLNLLKFRIRSFDETKDRNGLARSERLAGIFRMHQLCIRLMNWIQYSLKEIILIQYCSYVLDTAAFMIQLLTEETMRGKMPCFAGFMMTVIQMYIFFWFANEIQEESKTLSDVIYNEINWIDACKSEKKMLMLMMMRSQQMLTLKVAAIGDLSLNSFTKIMRLCYSVVTFFTTVYDK

>PstrOR30

MLFNFHPLGFESIIINNNNSTSLKRPLPLSSWFPWDEQKYYSLSYTFHILDVTMASLFMASTETLSYAITIYLLGQIVIFNSILSNFQLYCWKIQDQLKVDYENAIFLTLRECVKKHNEIIKQIKIFNREMKNAILYDFLQSSMGLAAGVLNLIYFDVTPFKLMFFGTFICGAFVRILVSYWYANEIIVQSSNMLTSLWNSTWYDEPEKTKKIKYLMMLVCSRPLYIDIGPFTNMSLQTLIRIVKATYSYMTLIYKSKN

>PstrOR31

MTRRNAETEECSYEFLRTDDCSKKTLLLPECILRFIFMWPRSDVKKQKVFYLFSMILFIFTQWGLVRFLIVNFDDYTRSLNVVSSMSTIFQASLKMSVLLYHSRSLSLILNDISHRFWPYDLLPNCTDRLRNSYRTKLVAMCTLSFTGLLFSLGSIVMPLLSHNAKELPYKCLYDFDTSVTPLYQIMYITESVLNAYIINCTVLGFDFLFMGVGENLINQYIMLRLTIENFGTKWVTNFNNKIRKLGLAEHLEGEDNTV

FLKNYISHHQIIIRSTKIVENIFNLIAALQLCSSVVAICVSAFIATRENVGTAQIATMGSYLIGHLIQLNFYCAVGNDLLYESNSLTNH
MFASNWYRIKNISLQKDFIFVIKNAQVPAKITAFKVFTLDYSTYIKVLRLSFSFYTLLSSLVEVKN
>PstrOR33
KRYQLQTAKNAKIKGYCKKFLPQCMTEKKSLIRENFYKTEMMLRNQGIYPVEGYPIASLFYAYLLYFGFTALIPLLGFCNIIFAESS
ERMGMLSNSFIFFEMGVSMFKHWPFIRHPDITKKMMDKWTTEMFTSDEPMEMPIIKKSLKAIDFVFYLFLAFSIGGPVGLTLNVL
FSSHDELKLDIYTFGVDVIHNKFLYVLTNVYLIIGYVHGTLGHFAVDIFIAGLMSHCSLQLSLIKFRLENMDKYLDKDLRSVQDDN
TFTEEVNDKIYRRISHCVDHYDTVMNYVKEIEDMYSVAAFSQFLAGSLVYAICLLYFMQLKALDFNFVYILTFFFPMIMVVFTYC
YYGDRISEESSSIGGAIFSSPFYNYNKKCHSSLIILMEKTKRPIKFTAGKLLELSLDTFVLIIKRSYSLLAVLRNYNN
>PstrOR34
MFFDRLAYRLMKTTDFCKLPLWFTKLLLQSALLWPVQKASATKKLLLFAVIMICCTFIKFGLLVTLTSKSSNIVESAAQIRDITLILQ
ASVKIVFLYYNHKKFQPFINMILNDFWPSNLFDEKSENRLKTFYNAVITMMILNTMFSTAYCITVTVSALLKGEFQMDTIYPFLDY
KSSPFYELNVMLQYVSLQYFCHVGIFGADFLFMSICTCVISQYKLLCRALSAFGTDEMMEINEKLRGIGTDQLIGRNYGLHKEYF
VRCVNHHRMLLMLTENVNSIFSFMTFLTLTVCTAGVCTGVFTLSSLETITVPMVLIIANYTMGFLTELFVYCIIGNEFYEEALLLPE
FIFAGNWNEYANNGMTKDLKFMIHRSQSVSPLHAYGLYNINMDSFAKVLKLSFSVYTFLSTIKNK
>PstrOR35
MYANAVFGIWPFIFEDYPILKKIYDLYCKFTLTYYAFFITTGYIKFFQLITEFPLNVTEVMLNVSITFLYSCTFVRAKRMRNPRMLK
NIQNIMDYEKKINDSKDEEMMKILEYYAWQNKIVSQAFIITVIIVTSLFVVHPLGLDPVEKFDSRTNTTRTIKPLPLSSWFPFNEQV
HYLPAYIWHIFDCYVGASFVNNTDIFSFGLITFPLGQIVVLNHILAHFEDYVVKVQKELGVERDEASFVAFRECILMHKNIIDYIYD
MNYEMSMIALMDFLQSSLQLAAIVLELMFFEVNLFNTVYSFMFVICMLTRLFGYYWYANEIIVESLNIPTSIWNGKWYEEPLKTQ
KMMLIMMKKCSRPLTMDIGPFNIMSINTLIGILKATYSYSMLIYRGKQKK
>PstrOR37
MSWSKRIIDIYSYILHFLGFSERQTKFDMFLIYFSALGAILNDCFILYNLKYTAFTIENITNFTETAFCFLEITFYSAIAIFKRSDFTALL
EMQSLCWDHAAFDRQFSRHTAKIFKLCLAFSVFFGLLGEITAISSSVLAFLDRGVPFYCYVPDGIVWYFIVFITQAYCSSYICVFAV
SMLIVYATIMFEMHYQLRMLNARFAEMATMGDLRRCVEHHGFLYGYFQRSRRMFSGVLLMLYVRSIGLSCMELLIAVDKNKPA
GLRFKALCFVPLIFLELCLVCVPISIVKNESENTPDAISRSDLLLHGDLRVRKNAIFLLKRCQKAFTFKAGDLYELDFNTPVQIFKSG
LSIYTLMKSFEN
>PstrOR38
MFLRDIFPKNEHFRVTMYSCSILGLWPFVFPKNPLYQKMYFRYSKFIYIFTWLFLSTYVVQLIKLLLDEVIFVEEIMRNISITAIQSLS
LIRAYALKSKRSKNMINEVLSTEQRILGSQNSGVIGIYNEFAKKNNSFITIYMASMVLDTCFFVIYPLCAPPIEIFYPTRNETVTKRM
LMLSAWFPFDEQKHYMAAYLFQFSCGPITEFYIILSDALTIGLVIYAIGQFRILNELFANFDKYAIQVQNQLKCSKEEACLTALRECT
IMHKKMLSFIDDFNAVMGNIMVLDFLQSSFQLASIVLQVLTTKVTLINFLLGFQFALSMVMRLLIYYWCANEIIVESSRISVSIYNS
CWYEQPEVVKKSLLLLMQRCNKPCTLEIGSFGIMSLETFISIMRATYSFITLIYNVNNEK
>PstrOR39
MTNQDSALRKSFKLDEFFLNLNGLLPGEKYKLLYSFYSFGMLLVYIIMIPILGYVEIFLAEPSRRVNMVDKSFVYLEIFVSVFKHW
PFITKPARTKRLLNRWNESIFNTDVDIDKHIIHESIRRFKLMRFLFFLMTVMSPLLLIQSIIFSSHDDLKLPIWVPVDIYNNTMLYTIT
NVYVVTGYIHGVFGYFSVDFLIAGYILYCATQIRLIKFKLENMDKYVEKEAIIDPQKTTTDGLPHKKIYDQIIQCIKVYDAVFGCVR
NFHVNLR
>PstrOR43
MENEIKRVSMSFSIKLFQYYGGFPQNHEFLNPGKIFYIKFVVKAVLSSFLLIGSILHLVHSIIVNIFNHVELDVAYSTALLIGYFLMLS
LILKMDKIGKFYTELSNFKEYEKPMDFEETNRRYNKYALMHFIYIMIFVLTSSLFSNIFKIETCRRENQEKGIHEVCGLYLYTWLPF
DIDFTPVRQMYLLWQIISGQFVHSFIGVCAWMVLESSEHVAIRFKHSGYLFTVAADEEDDQKRRVLLNKAVKYHKNSIRLAELLE
DIYSVFMFAHMFMTSVILGYDLYSFIQTPQLSTFMMCIGWVVGVFLVCQGGQRLQDTSLKVAELVYASNWTNYDISMQKEVSFV
IMRCQKHMSLKAIFIGDVGYMPFLSVLKAAYSYVMLMTNTAN
>PstrOR44

MPIKNAKPLESALIIPKLILIFAGRWPQHSSTLFTRVRVVTFNSMEILFNILMMVEVYLICTSDFNFNMLFDIMGALITASGHLFKIW
LFSAHRKIWLDILQQLKSTYFNDYPEEFHLIGRKPVNFSKNFGNMFQLGCCFSSTAYILAPLFSNIDLPIKCSFVTKAMIPYAYPIQS
FVVCFCALTHSSVDVMITDLIGVAVGQLDLLGEKIKAVRTNLIKQKNRFR
>PstrOR46
MFFDRLAFRVAGTTDFAKLFTWIPQMLLQFIFFWPKQKTYLKTVLFAIAMVFLIFINVGLAVEFSSTFDDLVRFVMHVTYITVTVQ
GTAKTLVIYINNGELEVILDDIMTKFWPYDLLETKLKKELKSFYYVIVIIMISLVTFGVLYSSMIMITPWASRKFPFDVSYPNIDYNA
SPYYELIYLVQIFTIQYFLFCLVLGCDYSFLAICSCVIAQYKLLQNALMAFHTPLMEEVNRKLRRIGNDGLEGKMYPIHKEYFIRC
VNHHHLLLGITKKMNSMFSSIEMVQLSCSMTGVCIGLFSLTALEDPPVSSLVIILSFTAIYFNELFVYCAIGNELHYQASFLPEFVFK
SNWNELADKELTRDFMFMLQRSQDIPQLSAYNLYDINMESYIKVFKLSFSFYTFLTTMKEK
>PstrOR48
MEEKTYFFPIARFFMEITTIWQPNKMTETSRKVYTALAYLFKGNYCIFIASYFIGLITTDSEEDRESSLQYLPTMIGIAVRMLFLHTK
EIKAIFSGIYRLETKLASEENKKLRNLFFDEVKYNKNLTKLLIALNSFTNIQMFILSIYLCMKLKMILLAVAWYPFDKHQYIYLVAL
HQIFSTSYSTVVYLGLDITIVPLILFATTRLKLLCYKFEHFKQFTDNIGLSPKDYLSLLIEEHQDIISYVVSVNHSMKWCFIVDFMLK
SYTFTQYLYTVLKSFNEDRARLGLALFAFIVVSVEIWYISYHGNELILASQELSKCIFANNNWYELDIGLQNDLLMIMVRSERPLSI
VVGNFYTIDNNLFLKIMKAGYTFLLFYNV
>PstrOR50
MTNQDSALRKSFKLDEFFLNLNGLLPGEKYKLLYSFYSFGMLLVYIIMIPILGYVEIFLAEPSRRVNMVDKSFVYLEIFVSVFKHW
PFITKPARTKRLLNRWNESIFNTAVEIDEHIVQDSIRYRIFFFQIYFTSGVISVSVLLISSIFSFNDRELKLPIWLPDQVLNNTFLYVIAN
IYIITGYTHAFFGHASTDSLLQTYLLYCATQLRLIKFKLKNIHKYFKKVATSEDSHKTTIEGLPQKKIYDQIVQCIKVYEAVNGYVQ
EMENMYSVGIFMQLIVVSLIFSICAYNLTTVRIYLRVSQERLGS
>PstrOR52
MYHENLNWCLKVYTFLGVHPRKKGIFRTIHYVFNVSCPFVITSLTLIMLHLNRPFNIEYLGEASTALTTFPHAIMKLTTLFLQQSKII
DLLERTRSQFWIVDDDNVEIKRAKRIGKVLKNAYFYSVAFFMFTTILKTILTHDLAYRCYQPKWIPKTLLIIYQDYTCCVILFTLMS
FDIMFQTLLFQTQLQFKMLNKKYKHLFDYENLGPTALKSMLKECVDHQSFLIDFVNRIKDTFSLSLLLFVGNIIVSLCMSVYIILSD
NSKLNLKIEAVVHTIAGLNEIGLCYSIPAQMFMDEAAEIRNSIYFSNWYQRPRLAKEIIPLLIREQKPLTITAGGFVMIDLQMFLVAC
KTILSYSMFLNTITQLQ
>PstrOR53
MRNQQSELRKSLKLLEFLLNVNGLLPGKKFKFFYSIFSYAFVIFFVIMIPVLGYVNIYLAEPSKRVDYLDKSFVYLEMFVLVFKHW
PFITNPNLTKILLDRWNESLFNTQVDVDKHIIAETLRRRRIWIVRNVLITAISPIILIQSMIFSSHDELMLPIWLPVDVSKNPVAFTVTN
MYIVIGYMHGFFGYFAVDILVISYIMYCATEMRLIKYKLKNVEKYIEKEATNNSDKTTSDGLPHKKIYDQIIQCIKVYDAVWKYAQ
ELESTYSFGIFIQFFVASMVLSICMYNLSKIKSIIEILYTASFLLPTILVIYYYCDQGSLIIDESTTIGDAIMKSPWYTYDEKTKHLLITF
MERTKRPIKFTVGKLVDVSLETFVLVNNRK
>PstrOR54
MTDQEFAIRKSFAHSELVMQVNGLLPGENFKSLYTFFAYALMIGFNILLPVLGYINLFKADPDERFTLLSNSFIYVEMFILVFKNWS
LVVTPDLTKKMFNRWNDDIFNTQVDEDKHIIDEAVRDHRIKYILYFILTGIAPTFLLINIIFSAHDDLKLPIWLPVDIYNSTLRYTLAN
VYIIVGYTHAVFAHFAIDVYLSSFMLYCASQLRLIKYKLENMEKYFEEDLLCISDEIFNDKLLQKKVYDKITQCAKVYDAVYSYIQ
ELEEIYSGGVFSQFLVGSMVVSICLYNMSKAQSLNLELIYAMGFLMPMTYLLYLYCNHGTLVIEESLTIGDAIIKSPWYNYDMKSK
RLLMILIERSKKPINFSAGKIVELSLETFVLIVKRSYSLLAVLRNY
>PstrOR57
MPPLPYDFKDHVEINVNFMYFFGIMRPKLDTKLENFLHNVRVAVLSVFMFGGIMSAELANLYYSFGDLEQLMRALFLTLSNLVSI
AKFHAVWRNQRRILDLIDAMNVAALRPRSRRQLAALGRYARLGKALTLGCAGAVTATCAFWAVYPITDDDGPFLPIPAYVPFDVR
NSTFRFALVYSFEIVATAVGGQMDLAADCLMAVLLMTFCAQLNVLNDSLVNLQPMETNRTKSLVKNMNAKLVHCIRQHRLIIEFT
ETWSGMFTYTIFSQFVVSVFVICATFFEMTQVPASSIRFFSMALYQMCMFLEIYPICYYGNEVIKESDKLTDSAYHCDWSECPLEF
QKNLVFFMTRSQRPIKLYAGNFFALSLETFIKILKSSWSFVAVLIQVNNDKID

>PstrOR58
MKYLGFNPPKRGYKRAVYIIFAAFHILCISIVLNALDWLEFFRVFRYNDMKLIMLRATMVLFSLVMGQRILLFACTFKKYQVIKDAVKRSSFNFECFGINNINVDSVSNGSRRKEKLSFSSIWKGAKLSFSESNELESFRLKSMVEMGARCSAILNAAIALCVILSIFWNYKETHGEFFEDYNPYLNKTSLYRKTQLRFYMPFDSSLEGHDALATCVTLYTRFGILLLFLPIDSVLPSILAFLIAQTRIVQEAFKYVDKNISPLQSPRNVFLIKEIRIVKCINELQEIYRILDMLQNLYSFQTLLQCGLVTFLLCTLGYIAPMVSDGSELFCFGTFLIAVTEEIFILSYVSNTLTIELQNVAKDLYNIEWDCYPIRVKKLIGFTLRRIQKPASLTACKLFNIDLAVFIQMLQTAYSFYTVINSTSKKLVA
>PstrOR59
MSSPDRPSRYFDTHRYLFNAFGIWTNKRFIANSLILTVIYAILVVALFIIAPQFCHVVYMYKARNDVVAFADEFYVSIASIVVVFKDYVLIKDGGAIREMLHTMDEALMRPSESQRRKFERTMGFWDKLFKCMLAGNYFFCACILVTPLTNRMELGTREVPLVDCYPFYIYSTPVYQFMYVYQSFLLVYLISHTVLFDTFVAGIVSVCSGECDILYENLVGLQSLRDESDYERELVKCIKHHEEIRKLVRNIEKRFSLIILEQYFTSLISCCTTMFKLSMAEPMSMEFFRTLSYQLNIFLQMYLFCWCASEVTEKSNRLPTAVYEALRMESSKSTKTTMCLFLGKTQKPLTLRILIFDLSMQLYLQLIRSSFSYYTVLMSLNEQ
>PstrOR62
MYGNMMRKFRMVNKMFQTAIKLITHNKRAANVSLKNEMLKSSKTLKHICLTFHFIFSPIAYNLYMFFQVTETTLPIKIMKALSLNRSYLLKFYHIMIFYIIPIHSITYIPTPVYTIVYCSRHIKFSLKQVVEEMKNFQRNDDQLKASITERLHSNKYQQKVKKELIYYTKQYNAIKRAADYVNHCIKWRFLSIFIVGGLSAAFNLVLILQVDFKSQIFQAGIVLIILAYNVITAECGEQIQLECESIYTNAQQFNWYNWNKSNCQLLFMLLMQTREPLQLKCYDMAVMNRVMILAFYQRVYSFISIFGAR
>PstrOR63
MHPLKGYIKPLIVFNAILTAYVTVLILLRLFLSKELVTVESLGVFFQVWVKFFVLTIKKDEIFKVIQYTQLFWKEDPAGSENSKILASLRKTEKYFLIYILFSTCMFLFKPLLVQGTTIYYYYKIEQIPFVVSYLIEFYVTLATMSMVIGVNLFICITINLGAGQFSNLNAKMRQLDLSETQQSGRGLEMCTVELREDIEYHECLISYVRQLDGIFSWLFTLLISIITSLLCMNMYVLSQPHNTVVDLIRCGTMVCAFTSEFLLLYGVPAQKLIDEAENVANSVFYHCKWYLPNIIRLRKAMSFMIFRSQKMVCLSALGFIDINRQTIVAMVKTAYSFFTFLQTMESPENAQN
>PstrOR65
MVLSEILKLLRSLNDLEKLMDSLFLTVTNLVSIVKFISIVRNERKIMKLLAQIDKREFRPKSQHQKNILESNIKAANVIALIVYSGCFVTVLFWVISAVIGDEVSLPVPSYIPFELTSTPVFVITFIYESIATLIGGFSNLSADCLMISFVIVISAQFNILNDTIENLEHFCEEEFMEKRMRIDRTSPEFQNLMNKKLIGCIEHYQCITEFSKTTNDLFSVTIFLQLFLSIVIISSTLYEISTGSITSQVKTFSMCAYLFCILGEIFPVCYFANEINEGSSRLTTSAYTCDWVDCSVEFKKNLLTFMTRTQKILRLYAGNFVEISLQMFMKIIKSSWSYWAVINQVDK
>PstrOR67
MASNKCEHVHMRFIKTFVVLSGIWPLDLKGIKKHLYDLYFRATFLFYVLNVCTVYVTAKEVAFRGKTTEITDYVANTIFATLLILKALTYRSKGIKNLLDSILELEKEVLEKYDDESRSIYMENVKSAEYLGSVYIFQGCFMSLCAQVFPLIMTLMSVPDEHGRVEKYYIVPNWEPFDKYEHYYAAFALQFVYTIIAETYIVFCGTFFLFVLKNVQGQLRVLQHRFREGHSVKDCIRFHQAIIQLFNDFNSTFYMFIFME
>PstrOR69
MESSIVIFIYELFFSKRLNRNYVANIVGIINSQPFIIMAFINPYFVLLFKNIERDLAKFCGSLTTEDANFNRTISREILKTKFTFFGLAVLLFLCNYINAVFNLKGEMKIYLVISYILEYTNEMATTIMLLSMVTMLSCHAVVSLSFIIYCIAHVICQIRLLRYLLQENLNNLSHSIDDENYQNYIGEILKFCAKHHNSIAYFNNYYLSLMQMPTLVLSALGGVAIAVNYYFVYAKTDPFYDTTMYCNIGMSLILITTYTHYGQLLADESENLYNTLCDTPWIHWNKQNRQMLQIMMVNSMEPLKISLIQGQTFNYAYNLSVLQTCYSVFMLATSL
>PstrOR70
MRFEMIELINKRPYYVCTKAMVYTCLLPKKNCPMSIFYLYSTVYRLILTIPYALIILNTFLKIKNYQKATGVNFSEELAMCCSYGGMYLTIIIFQVKHKDWLRFIDKVVDHSEFGIPPTFDEAQRNSNIVTAMMFLYAAIGVTFYALVPLFGIEECRRMNQEKHLKESCSGLPVWLPFETTNNKQLFILTFYELASSMSICVPASSVAFMAYNSSKILISKIDQFKELLLEALKEKDVRVRRDKLRYCVKYHNHILSLGDELKRLVRVTVGSYGIMAALSIGGIGNQIIQEQSARSIVHFFGFAVAAFTMCHAGQIIATESVSIKEVIYATDWYLCDAKTMKDIRFMLARSQIPITLEMLPLGWLNYEFFTMIMKTSYSYITLLSKAT

IR sequences

>DmelGluRIIC
MWQRILLLGCMWSAFFMCRSRGQQINIGAFFYDDELELEKEFMTVVNAINGPESEQTMRFYPLIKRLKPEDGSVTMQEHACDLIDNGVAAIFGPSSKAASDIVALVCNSTGIPHIEFDISDEGIQAEKPNHQMTLNLYPAQAILSKAYADIVQNFGWRKFTIVYDADDARAAARLQDLLQLREVHNDVVRVRKFHKDDDFRVMWKSIRGERRVVLDCEPNMLVELLNSSTEFGLTGQYNHIFLTNLETYTDHLEELAADNETFAVNITAARLLVNPDPPPYSLPYGYVTQRDNIVYESSDPPRTLIHDLIHDALQLFAQSWRNASFFYPDRMVVPRITCDFAASGGRTWAMGRYLARLMKGTSGVNNTNFRTSILQFDEDGQRITFNIEVYDPLDGIGIAIWDPRGQITQLNVDVKAQKKMIYRVATRIGPPYFSYNETARELNLTGNALYQGYAVDLIDAIARHVGFEYVFVPVADQQYGKLDKETKQWNGIIGEIINNDAHMGICDLTITQARKTAVDFTVPFMQLGVSILAYKSPHVEKTLDAYLAPFGGEVWIWILISVFVMTFLKTIVARISKMDWENPHPCNRDPEVLENQWRIHNTGWLTVASIMTAGCDILPRSPQVRMFEATWWIFAIIIANSYTANLAAFLTSSKMEGSIANLKDLSAQKKVKFGTIYGGSTYNLLADSNETVYRLAFNLMNNDDPSAYTKDNLEGVDRVRKNRGDYMFLMETTTLEYHREQNCDLRSVGEKFGEKHYAIAVPFGAEYRSNLSVAILKLSERGELYDLKQKWWKNPNASCFEEPDPDATPDMTFEELRGIFYTLYAGILIAFLIGITEFLVYVQQVALEERLTFKDAFKKEIRFVLCVWNNRKPIVAGTPISSVRTTPRRSLDKSLDRTPKSSRRVVIGRSSEEMREMAQGSGSSSGSNNAGRGEKEARV

>DmelGluRIIA
MRLCPVVIYAFIIIIGFLEGIIALGGDDRNEITVGAIFYENEKEIELSFDQAFREVNNMKFSELRFVTIKRYMPTNDSFLLQQITCELISNGVAAIFGPSSKAASDIVAQIANATGIPHIEYDLKLEATRQEQLNHQMSINVAPSLSVLSRAYFEIIKSNYEWRTFTLIYETPEGLARLQDLMNIQALNSDYVKLRNLADYADDYRILWKETDETFHEQRIILDCEPKTLKELLKVSIDFKLQGPFRNWFLTHLDTHNSGLRDIYNEDFKANITSVRLKVVDANPFERKKTRLTKVDQILGNQTMLPILIYDAVVLFASSARNVIAAMQPFHPPNRHCGSSSPWMLGAFIVNEMKTISEDDVEPHFKTENMKLDEYGQRIHFNLEIYKPTVNEPMMVWTPDNGIKKRLLNLELESAGTTQDFSEQRKVYTVVTHYEEPYFMMKEDHENFRGREKYEGYAVDLISKLSELMEFDYEFMIVNGNGKYNPETKQWDGIIRKLIDHHAQIGVCDLTITQMRRSVVDFTVPFMQLGISILHYKSPPEPKNQFAFLEPFAVEVWIYMIFAQLIMTLAFVFIARLSYREWLPPNPAIQDPDELENIWNVNNSTWLMVGSIMQQGCDILPRGPHMRILTGMWWFFALMMLSTYTANLAAFLTSNKWQSSIKSLQDLIEQDKVHFGSMRGGSTSLFFSESNDTDYQRAWNQMKDFNPSAFTSTNKEGVARVRKEKGGYAFLMETTSLTYNIERNCDLTQIGEQIGEKHYGLAVPLGSDYRTNLSVSILQLSERGELQKMKNKWWKNHNVTCDSYHEVDGDELSIIELGGVFLVLAGGVLIGVILGIFEFLWNVQNVAVEERVTPWQAFKAELIFALKFWVRKKPMRISSSSDKSSSRRSSGSRRSSKEKSRSKTVS

>DmelGluRIIB
MHGLQFLVLLALAIASGANEDTLVIKIGAIFFDTEMKLADAFSAALEEVNAINPALKLDAIKRYVTVDDSIVLQDISCDLIGSGVAAIFGPSSKTNSDIVEVLCNMTGIPHLQFDWHPQQSNRERMNHQLTVNVAPMELFLSAAFSDILASKTFDWKSFTIAYERSSHLIRLQHILAWKQLHKAGIKMQEFERGDDYRILWKRINNAREKFVLLDCPSDILVDVINASIGYNMTGSFNHLFLTNLDTHLSGIDGFYSRDFTVAVAAVRIRTYVPPPVHDEIDVFDNSVDTRFSSLGSQLVYDSIVLFYNALLEISQRPGFYIPNFSCGRGFWQPGPRLVEQMKQITPKMVKPPFKTQRLQINADGQREDFNLEVYNPIIDRVTHIWNKEFQLVDFEKLRENSTQALKQKRLQNKEDFSQKPIRYTVATRVGKPYFSWREEPEGVHYEGNERFEGYAVDLIYMLAQECKFDFNFEPVRDNKYGSYDANTDEWDGIIRQLIDNNAQIGICDLTITQARRSVVDFTVPFMQLGISILSYKEPPPKADIYAFLNPYNAEVWLFVMIAMMITAFALIFTGRIDQYEWDQPVENVNREMERQNIWHLSNALWLVLGSMLNQGCDLLPRGLPMRLLTAFWWIFALLISQTYIAKLAAFITSSKIAGDIGSLHDLVDQNKVQFGTIRGGATSVYFSESNDTDNRMAWNKMLSFKPDAFTKNNEEGVDRVKLSKGTYAFLMETTNLQYYVQRNCELTQIGESFGEKHYGIAVPLNADFRSNLSVGILRLSERGELFKLRNKWFNSNESTCDSNVPTIDDGQFDMDSVGGLFVVLIVGVVVGLVIGVAEFLWHVQRISVKEKIPPMLALKAEFYFVIRFWLTRKPLHTYRQSRDSTSTGYSSLEQITSASSAKKKKKTRRIEK

>DmelClumsy
MYSLFLTHFLLIALPVLADIDRSQFMVGSIFTSDKDESEIAFRTAVDRANILERNVELVPIVVYANTDDSFIMEKMVCNLISQGVIAIFGPSTGSSSDIIASICDTLDIPHIVYDWIPNESIPDREHSTMTLNVHPDNLLLSQGLAEIVQSFAWRSFTVVYETDKELQQLQDILQVGEPISNPTTVKQLGPGDDHRPFLKEIKLSTDNCLILHCAPDNLLKILQQANELKMLGEYQSVFIPLLDTHSIDFGELSGVEANITTVRLMDPSDFHVKNVVHDWEEREKREGRYFKVDPNRVKSQMILLNDAVWLFSKGLTELGIFEELTAPDLECRRKKPWPFGKRIIEFIKARSEETSTGRIDFNENGQRSFFTLRFMELNSDGFLDLATWDPVNGLDVLNDDEESEKRVGQKLSNKTFIVSSRLGAPFLTLREP

QEGEILTGNSRYEGYSIDLINEIAKMLNFKFEFRMSPDGKYGALNKVTQTWDGIVRQLIDGNADLGICDLTMTSSRRQAVDFTPPF
MTLGISILFSKPPTPPTDLFSFLSPFSLDVWIYMGSAYLFISLLLFALARMAPDDWENPHPCKEPEEVENIWSIMNTTWLSIGSLMG
QGCDILPKAASTRLVTGMWWFFALMMLNSYTANLAAFLTNSRQANSINSAEDLAAQSKIKYGAMAGGSTMGFFRDSNFSTYQK
MWTAMESASPSVFTKTNDEGVERVQKGKNLYAFLMESTTLEYNVERKCDLVQIGGWLDYKSYGIAMPFNSPYRKQISAAVLKL
GELGQLAELKRKWWKEMHGGGNCEKSDEDGGDTPELGLENVGGVFLVLGLGLLSAMVLGCTEFLWNVKSVAIEEKISLKEAF
KSEALFAARIWITTKPVHTSSESGSSNSSSSSSSRSKHSFKSQGLSMKSLKSSGYQDVEASVHSKLKKIGSMFSLKSQKTVTPPPEI
GWKLDKSTQIDVVPTSDVDQELIPEVEPHLPHRHHHHHHHRHHHHHHQPDQEHDRNPSPPE
>DmelGluRIID
MHFCWISLIILSLSRVQAQFYGGNAYEASSGQSIRLGLITDDATDRIRQTFEHAISVVNNELGVPLVGETEQVAYGNSVQAFAQLC
RLMQSGVGAVFGPAARHTASHLLNACDSKDIPFIYPHLSWGSNPDGFNLHPSPEDIANALYDIVNQFEWSRFIFCYESAEYLKILD
HLMTRYGIKGPVIKVMRYDLNLNGNYKSVLRRIRKSEDSRIVVVGSTTGVAELLRQAQQVGIMNEDYTYIIGNLNLHTFDLEEY
KYSEANITGIRMFSPDQEEVRDLMEKLHQELGESEPVNSGSTFITMEMALTYDAVRVIAETTKHLPYQPQMLNCSERHDNVQPD
GSTFRNYMRSLEIKEKTITGRIYFEGNVRKGFTFDVIELQTSGLVKVGTWEEGKDFEFQRPPQAVNFNDIDDGSLVNKTFIVLISVA
TKPYASLVESIDTLIGNNQFQGYGVDLIKELADKLGFNFTFRDGGNDYGSFNKTTNSTSGMLKEIVEGRADLAITDLTITSEREEVI
DFSIPFMNLGIAILYVKPQKAPPALFSFMDPFSSEVWLYLGIAYLGVSLCFFIIGRLSPIEWDNPYPCIEEPEELENQFTINNSLWFTT
GALLQQGSEIAPKALSTRTISAIWWFFTLIMVSSYTANLAAFLTIENPTSPINSVKDLADNKDDVQYGAKRTGSTRNFFSTSEEPIYI
KMNEYLNAHPEMLMENNQQGVDKVKSGTKYAFLMESTSIEFNTVRECNLTKVGDPLDEKGYGIAMVKNWPYRDKFNKALLE
LQEQGVLARLKNKWWNEVGAGVCSAKSDDDGPSELGVDNLSGIYVVLVIGSIISIIISILCWCYFVYKKAKNYEVPFCDALAEEF
RIVIRFSENERPLKSAQSIYSRSRNSSQSIESLKTDSEENMPVED
>DmelGluRIIE
MFFNHFVILWSLFSIHISVNWAQYENFGGYDNYQSLESVPIGLLTDQNTEQMNIVFDHAIDVANQEVGTSLTSLKEEVNYGDAYQ
SYGKLCRMLETGIAGVFGPSSRHTAVHLMSICDAMDIPHIYSYMSENAEGFNLHPHPADLAKALYSLITEFNWTRFIFLYESAEYL
NILNELTTMLGKSGTVITVLRYDMQLNGNYKQVLRRVRKSVDNRIVVVGSSETMPEFLNQAQQVGIINEDYKYIIGNLDFHSFDL
EEYKYSEANITGLRLFSPEKMAVKELLMKLGYPTDQDEFRNGSCPITVEMALTYDAVQLFAQTLKNLPFKPMPQNCSQRTESVR
DDGSSFKNYMRTLRLTDRLLTGPIYFEGNVRKGYHLDVIELQPSGIVKVGTWDEDRQYRPQRLAPTTAQFDSVDNSLANKTFIIL
LSVPNKPYAQLVETYKQLEGNSQYEGYGVDLIKELADKLGFNFTFVNGGNDYGSYNKSTNESTGMLREIMTGRADLAITDLTITS
EREQALDFTIPFMNLGIAILYLKPQKATPELFTFMDPFSEEVWWFLGFSFLGVSLSFFILGRLSPSEWDNPYPCIEEPEELENQFTLG
NSIWFTTGALLQQGSEIGPKALSTRTVASFWWFFTLIVVSSYTANLAAFLTIEKPQSLINSVDDLADNKDGVVYGAKKTGSTRNFF
MTSAEERYKKMNKFMSENPQYLTEDNMEGVNRVKTNTHYAFLMESTSIEYNTKRECNLKKIGDALDEKGYGIAMRKDWPHRG
KFNNALLELQEQGVLEKMKNKWWNEVGTGICATKEDAPDATPLDMNNLEGVFFVLLVGSCCALLYGIISWVLFVMKKAHHYR
VPLRDALKEEFQFVIDFNNYVRVLKNSASIYSRSRQSSMSVASVAQESQ
>DmelCG3822
MRSSGVLVLPLLLLQLILNCRKAQSLPDIIKIGGLFHPADDHQELAFRQAVDRINADRSILPRSKLVAQIERISPFDSFHAGKRVCGL
LNIGVAAIFGPQSSHTASHVQSICDNMEIPHLENRWDYRLRRESCLVNLYPHPNTLSKAYVDIVRHWGWKTFTIIYENNDGIVRLQ
ELLKAHGMTPFPITVRQLSDSGDYRPLLKQIKNSAEAHIVLDCSTERIHEVLKQAQQIGMMSDYHSYLVTSLDLHTVNLDEFRYG
GTNITGFRLINEKIVSDVVRQWSIDEKGLLRSANLTTVRSETALMYDAVHLFAKALHDLDTSQQIDIHPISCDGQSTWQHGFSLIN
YMKIVEMKGLTNVIKFDHQGFRTDFMLDIVELTPAGIRKIGTWNSTLPDGINFTRTFSQKQQEIEANLKNKTLVVTTILSNPYCMR
KESAIPLSGNDQFEGYAVDLIHEISKSLGFNYKIQLVPDGSYGSLNKLTGEWNGMIRELLEQRADLAIADLTITFEREQAVDFTTPF
MNLGVSILYRKPIKQPPNLFSFLSPLSLDVWIYMATAYLGVSVLLFILAKFTPYEWPAYTDAHGEKVESQFTLLNCMWFAIGSLMQ
QGCDFLPKALSTRMVAGIWWFFTLIMISSYTANLAAFLTVERMDSPIESAEDLAKQTRIKYGALKGGSTAAFFRDSKISTYQRMW
SFMESARPSVFTASNGEGVERVAKGKGSYAFLMESTSIEYVTERNCELTQVGGMLDTKSYGIATPPNSPYRTAINSVILKLQEEGK
LHILKTKWWKEKRGGGKCRVETSKSSSAANELGLANVGGVFVVLMGGMGVACVIAVCEFVWKSRKVAVEERLSAILNE
>DmelCG5621
MISTEASFPLGFILTSLLLAFPGCRGERTNVGLVYENTDPDLEKIFHLAISKANEENEDLQLHGVSVSIEPGNSFETSKKLCKMLRQ

NLVAVFGPTSNLAARHAMSICDAKELPFLDTRWDFGAQLPTINLHPHPATLGVALRDMVVALGWESFTIIYESGEYLPTVRELLQ
MYGTAGPTVTVRRYELDLNGNYRNVLRRIRNADDFSFVVVGSMATLPEFFKQAQQVGLVTSDYRYIIGNLDWHTMDLEPYQHA
GTNITGLRLVSPDSEQVQEVAKALYESEEPFQNVSCPLTNSMALVYDGVQLLAETYKHVNFRPVALSCNDDSAWDKGYTLVNY
MKSLTLNGLTGPIRFDYEGLRTDFKLEVIELAVSGMQKIGQWSGEDGFQENRPAPAHSLEPDMRSLVNKSFVVITAISEPYGMLKE
TSEKLEGNDQFEGFGIELIDELSKKLGFSYTWRLQEDNKYGGIDPKTGEWNGMLREIIDSRADMGITDLTMTSERESGVDFTIPFM
SLGIGILFRKPMKEPPKLFSFMSPFSGEVWLWLGLAYMGVSISMFVLGRLSPAEWDNPYPCIEEPTELENQFSFANCLWFSIGALL
QQGSELAPKAYSTRAVAASWWFFTLILVSSYTANLAAFLTVESLVTPINDADDLSKNKGGVNYGAKIGGATFNFFKESNYPTYQR
MYEFMRDNPQYMTNTNQEGVDRVENSNYAFLMESTTIEYITERRCTLTQVGALLDEKGYGIAMRKNWPYRDTLSQAVLEMQE
QGLLTKMKTKWWQEKRGGGACSDADEDSGAVALEISNLGGVFLVMGVGSFFGIFVSLLEMVLGVKERSDENQEAPDSDASSLG
FANLGGVYLVMFVGSCFGSIYGLVNCVVSVYLRARENKVSFKTELLDEIRFILQCSGNTKAVKYPKNSSRSNASSKSKGSSMSVD
SLPEDTSEADASGKHNHGKK
>DmelCG9935
MLIASGFLLFQFLSYGLGVPPLVRIGAIFSNQPGMYNSELAFRYAIHRLNMDKSLLPETTVDYYVEYVNRFDSFETVQKVCKLIRV
GVQAVFSPTDSVLATHINSICDALDIPNIGRSAHDFSINVYPSKQLVNYAFNDVIQYLNWTRFGILHEKENGIINLHQLSRSFHGEV
HMRQVSRDSYVSALNEFKGKEIHNIIIDTNSNGISILLKNILQQQMNEYKYHYLFTSFDLETYDLEDFKYNFVNITSFRLVDTADV
GVKQILKDIGLYSHHIFKKPYLNLHIKKSTILESEPALMFDSVYVFAIGLQTLEQSHSLTLLNISCEEENSWDGGLSLINYLNAVEW
KGLTGPIQFKDGQRVQFKLDLIKLKQHSIVKVGEWTPHGHLNITEPSMFFDAGSMNVTLVVITILETPYVMMHYGKNFTGNERF
YGFCVDILETISREVGFDYILDLVPDRKYGAKDPETGEWNGMVAQLMKYKADLAVGSMTITYARESVIDFTKPFMNLGISILFKV
PTSEPTRLFSFMNPLAIEIWIYVLIAYFLVSLCIYIVGKLSPIEWKCINACDLENISIGNQFSLTDSFWFTIGTFMQQSPDIYPRAMSTR
IISSTWGFFSLIIVASYTANLAAFLTTERMINPIENAEDLASQTEISYGTLDSGSTMTFFRDSVIETYKKIWRSMDNKKPSAFTTTYE
DGIKRVNQGNYAFLMESTMLDYIVQRDCNLTQIGGLLDTKGYGIATPKGSPWRDKISLAILELQERGDIQMLYDKWWKNTDETC
TRKNTSKQSKANSLGLESIGGVFVVLIAGIIVAAVVAFFEFWYNFRYNYEATPSQSVVNNKYNQDGILESERNYTPPDRSFWIEIAE
ELRYASWCMNKQKRPALTRTCSKCTIPKGQRINKL
>DmelCG11155
MVRKKREIVIKENIQGRSYLKKICCSYIILSILVISNALPPVIRVGAIFTEDERESSIESAFKYAIYRINKEKTLLPNTQLVYDIEYVPR
DDSFRTTKKVCSQLEAGVQAIFGPTDALLASHVQSICEAYDIPHIEGRIDLEYNSKEFSINLYPSHTLLTLAYRDIMVYLNWTKVAII
YEEDYGLFNLMHSSTETKAEMYIRQASPDSYRQVLRAIRQKEIYKIIVDTNPSHIKSFFRSILQLQMNDHRYHYMFTTFDLETYDL
EDFRYNSVNITAFRLVDVDSKRYLEVINQMQKLQHNGLDTINGSPYIQTESALMFDSVYAFANGLHFLNLDNHQNFYIKNLSCTS
DQTWNDGISLYNQINAAITDGLTGTVQFVEGRRNIFKLDILKLKQEKIQKVGYWHPDDGVNISDPTAFYDSNIANITLVVMTREE
RPYVMVKEDKNLTGNLRFEGFCIDLLKAIATQVGFQYKIELVPDNMYGVYIPETNSWNGIVQELMERRADLAVASMTINYARES
VIDFTKPFMNLGIGILFKVPTSQPTRLFSFMNPLAIEIWLYVLAAYILVSFALFVMARFSPYEWKNPHPCYKETDIVENQFSISNSFW
FITGTFLRQGSGLNPKATSTRIVGGCWFFFCLIIISSYTANLAAFLTVERMISPIESASDLAEQTEISYGTLEGGSTMTFFRDSKIGIYQ
KMWRYMENRKTAVFVKTYEDGIKRVMEGSYAFLMESTMLDYAVQRDCNLTQIGGLLDSKGYGIATPKGSPWRDKISLAILELQE
KGIIQILYDKWWKNTGDVCNRDDKSKESKANALGVENIGGVFVVLLCGLALAVVVAIFEFCWNSRKNLNTENQSLCSEMAEEL
RFAMHCHGSKSRHRPRKRSCLNCSSVPTYVPSNVSTSNVGVYYNYFN
>DmelGluR1
MHSRLKFLAYLHFICASSIFWPEFSSAQQQQQTVSLTEKIPLGAIFEQGTDDVQSAFKYAMLNHNLNVSSRRFELQAYVDVINTAD
AFKLSRLICNQFSRGVYSMLGAVSPDSFDTLHSYSNTFQMPFVTPWFPEKVLAPSSGLLDFAISMRPDYHQAIIDTIQYYGWQSIIY
LYDSHDGLLRLQQIYQELKPGNETFRVQMVKRIANVTMAIEFLHTLEDLGRFSKKRIVLDCPAEMAKEIIVQHVRDIKLGRRTYH
YLLSGLVMDNHWPSDVVEFGAINITGFRIVDSNRRAVRDFHDSRKRLEPSGQSQSQNAGGPNSLPAISAQAALMYDAVFVLVEAF
NRILRKKPDQFRSNHLQRRSHGGSSSSSATGTNESSALLDCNTSKGWVTPWEQGEKISRVLRKVEIDGLSGEIRFDEDGRRINYTL
HVVEMSVNSTLQQVAEWRDDAGLLPLHSHNYASSSRSASASTGDYDRNHTYIVSSLLEEPYLSLKQYTYGESLVGNDRFEGYCK
DLADMLAAQLGIKYEIRLVQDGNYGAENQYAPGGWDGMVGELIRKEADIAISAMTITAERERVIDFSKPFMTLGISIMIKKPVKQ
TPGVFSFLNPLSQEIWISVILSYVGVSFVLYFVTRFPPYEWRIVRRPQADSTAQQPPGIIGGATLSEPQAHVPPVPPNEFTMLNSFW

YSLAAFMQQGCDITPPSIAGRIAAAVWWFFTIILISSYTANLAAFLTVERMVAPIKTPEDLTMQTDVNYGTLLYGSTWEFFRRSQIG
LHNKMWEYMNANQHHSVHTYDEGIRRVRQSKGKYALLVESPKNEYVNARPPCDTMKVGRNIDTKGFGVATPIGSPLRKRLNEA
VLTLKENGELLRIRNKWWFDKTECNLDQETSTPNELSLSNVAGIYYILIGGLLLAVIVAIMEFFCRNKTPQLKSPGSNGSAGGVPG
MLASSTYQRDSLSDAIMHSQAKLAMQASSEYDERLVGVELASNVRYQYSM
>DmelGluR1B
MRFGLKLSCLWPSFLLWLTWSSGGGGGSGVGVSAQPSLTEKIPLGAIFEQGTDEVQSAFKYAMLNHNLNVSSRRFELQAYVDVI
NTADAFKLSRLICNQFSRGVYSMLGAVSPDSFDTLHSYSNTFQMPFVTPWFPEKVLTPSSGFLDFALSMRPDYHQAIIDTIQFYGW
RKIIYLYDSHDGLLRLQQIYQGLRPGNESFQVELVKRISNVSMAIEFLHTLEQIGRFENKHIVLDCPTEMAKQILIQHVRDLRLGRR
TYHYLLSGLVMDDRWESEIIEFGAINITGFRIVDTNRRLVREFYDSWKRLDPQMSVGAGRESISAQAALMYDAVFVLVEAFNKIL
RKKPDQFRNNVQRRSQTLMVAQAAASTSSDGYNYSASGGGGGNGGAGGGFAGSDSGGSGGMASRALDCNTAKGWVNAWEH
GDKISRYLRKVEIEGLTGDIKFNDDGRRVNYTLHVVEMTVNSAMVKVAEWNDDAGLQPLNAKYVRLRPHVEFEKNRTYIVTTV
LEEPYIMLKQVAFGEKLHGNNRFEGYCKDLADLLAKELGINYELRLVKDGNYGSEKSSAHGGWDGMVGELVRKEADIAIAAMT
ITAERERVIDFSKPFMSLGISIMIKKPVKQTPGVFSFMNPLSQEIWVSVIFSYIGVSIVLFFVSRFSPHEWRLVQQQPQQSQSPDPHA
HHEQLANQQPPGIIGGAPLPAPPGPPTPGAQTAAGAAALQAALSAGSPGSGGSSSAVVNEFSVWNSFWFSLAAFMQQGCDLSPR
SVSGRIAAASWFFFTLILISSYTANLAAFLTVERMVTPINSPEDLAMQTEVQYGTLLHGSTWDFFRRSQIGLHNKMWEYMNSRK
HVFVPTYDEGIKRVRNSKGKYALLVESPKNEYVNAREPCDTMKVGRNLDTKGFGIATPLGSALKDPINLAVLTLKENGELIKLRN
KWWYEKAECSTHKDGETSHSELSLSNVAGIFYILIGGLLVSVFVAILEYCFRSRDSRSASSGSGMGLGMGLGGGMSGGSLGKAN
GSMMLGPSSAVPGGMPSSHQRSTLTDTMHAKAKLTIQASRDYDNGRVGYLNCASLQYYPPAQLSATPPDAGDSLHMNAHGQV
>DmelNmdar2
MMPSRVKLKRGTDGPTPTPTPMPTTMRKHTPIATLNTASCQHNSTTSRRKRILTPPSGPISLLLLTVLTLLILDTRSCQGLRLTNGG
GSLSKGAAANKEQLNIGLIAPHTNFGKREYLRSINNAVTGLTKTRGAKLTFLKDYSFEQKNIHFDMMSLTPSPTAILSTLCKEFLRV
NVSAILYMMNNEQFGHSTASAQYFLQLAGYLGIPVISWNADNSGLERRASQSTLQLQLAPSIEHQSAAMLSILERYKWHQFSVV
TSQIAGHDDFVQAVRERVAEMQEHFKFTILNSIVVTRTSDLMELVNSEARVMLLYATQTEAITILRAAEEMKLTGENYVWVVSQS
VIEKKDAHSQFPVGMLGVHFDTSSAALMNEISNAIKIYSYGVEAYLTDPANRDRRLTTQSLSCEDEGRGRWDNGEIFFKYLRNVS
IEGDLNKPNIEFTADGDLRSAELKIMNLRPSANNKNLVWEEIGVWKSWETQKLDIRDIAWPGNSHAPPQGVPEKFHLKITFLEEA
PYINLSPADPVSGKCLMDRGVLCRVAADHEMAADIDVGQAHRNESFYQCCSGFCIDLLEKFAEELGFTYELVRVEDGKWGTLEN
GKWNGLIADLVNRKTDMVLTSLMINTEREAVVDFSEPFMETGIAIVVAKRTGIISPTAFLEPFDTASWMLVGIVAIQAATFMIFLFE
WLSPSGYDMKLYLQNTNVTPYRFSLFRTYWLVWAVLFQAAVHVDSPRGFTSRFMTNVWALFAVVFLAIYTANLAAFMITREEFH
EFSGLNDSRLVHPFSHKPSFKFGTIPYSHTDSTIHKYFNVMHNYMRQYNKTSVADGVAAVLNGNLDSFIYDGTVLDYLVAQDED
CRLMTVGSWYAMTGYGLAFSRNSKYVQMFNKRLLEFRANGDLERLRRYWMTGTCRPGKQEHKSSDPLALEQFLSAFLLLMA
GILLAALLLLLEHVYFKYIRKRLAKKDGGHCCALISLSMGKSLTFRGAVFEATEILKKHRCNDPICDTHLWKVKHELDMSRLRVR
QLEKVMDKHGIKAPQLRLASSSDLLNHHHLKERPPLLGNLSLAASAQDLYRWSYKTEIAEMETVL
>DmelNmdar1
MAMAEFVFCRPLFGLAIVLLVAPIDAAQRHTASDNPSTYNIGGVLSNSDSEEHFSTTIKHLNFDQQYVPRKVTYYDKTIRMDKNPI
KTVFNVCDKLIENRVYAVVVSHEQTSGDLSPAAVSYTSGFYSIPVIGISSRDAAFSDKNIHVSFLRTVPPYYHQADVWLEMLSHFA
YTKVIIIHSSDTDGRAILGRFQTTSQTYYDDVDVRATVELIVEFEPKLESFTEHLIDMKTAQSRVYLMYASTEDAQVIFRDAGEYN
MTGEGHVWIVTEQALFSNNTPDGVLGLQLEHAHSDKGHIRDSVYVLASAIKEMISNETIAEAPKDCGDSAVNWESGKRLFQYLK
SRNITGETGQVAFDDNGDRIYAGYDVINIREQQKKHVVGKFSYDSMRAKMRMRINDSEIIWPGKQRRKPEGIMIPTHLRLLTIEEK
PFVYVRRMGDDEFRCEPDERPCPLFNNSDATANEFCCRGYCIDLLIELSKRINFTYDLALSPDGQFGHYILRNNTGAMTLRKEWT
GLIGELVNERADMIVAPLTINPERAEYIEFSKPFKYQGITILEKKPSRSSTLVSFLQPFSNTLWILVMVSVHVVALVLYLLDRFSPFGR
FKLSHSDSNEEKALNLSSAVWFAWGVLLNSGIGEGTPRSFSARVLGMVWAGFAMIIVASYTANLAAFLVLERPKTKLSGINDARL
RNTMENLTCATVKGSSVDMYFRRQVELSNMYRTMEANNYATAEQAIQDVKKGKLMAFIWDSSRLEYEASKDCELVTAGELFGR
SGYGIGLQKGSPWTDAVTLAILEFHESGFMEKLDKQWIFHGHVQQNCELFEKTPNTLGLKNMAGVFILVGVGIAGGVGLIIIEVIY
KKHQVKKQKRLDIARHAADKWRGTIEKRKTIRASLAMQRQYNVGLNSTHAPGTISLAVDKRRYPRLGQRLGPERAWPGDAAD

VLRIRRPYELGNPGQSPKVMAANQPGMPMPMLGKTRPQQSVLPPRYSPGYTSDVSHLVV
>DmelIR8a
MELPLLVLLLALRFAGSEVLKITFWIEPVQRAEFDTDIAMVLKELDALRLDVKVDDTTLTLTRSEDGLDMQRFCEILSTVGASAVI
DLTYSHWEEGYNLVRSLGIGYVRLERIMRPFLDMFGDFMRQKRANNVAMVFMNARDAVEAMQQMLVGYPFRTLIMDASQTDP
GQHFLERIRSLRPAPTYIALFARAAAMNGIFEKVQKADLFQRPLEWHFVFLDTRDRVFKYRRQAELCTRFTLNPRAICRSMPMPD
LYCGSGFTMQRAMLLNVLRSLINAAQVSPGYPLAIYQDCNATASSSEVSDPLEKDDYNWLDMVHWSNFLAYAPPLPHIQDQFQS
PVPGLTFAVNISAGYYSSEHEAKTDLAAWSSVGEMRLLNETISPARRFFRIGTAESIPWSYLRREEGTGELIRDRSGLPIWEGYCIDF
IIRLSQKLNFEFEIVAPEVGHMGELNELGEWDGVVGDLVRGETDFAIAALKMYSEREEVIDFLPPYYEQTGISIAIRKPVRRTSLFK
FMTVLRLEVWLSIVAALVGTAIMIWFMDKYSPYSSRNNRQAYPYACREFTLRESFWFALTSFTPQGGGEAPKAISGRMLVAAYWL
FVVLMLATFTANLAAFLTVERMQTPVQSLEQLARQSRINYTVVKDSDTHQYFVNMKFAEDTLYRMWKELALNASKDFKKFRIW
DYPIKEQYGHILLAINSSQPVADAKEGFANVDAHENADYAFIHDSAEIKYEITRNCNLTEVGEVFAEQPYAVAVQQGSHLGDELSY
AILELQKDRFFEELKAKYWNQSNLPNCPLSEDQEGITLESLGGVFIATLFGLVLAMMTLGMEVLYYKKKQNALEITQVRPVNDSS
GSGGNSSTAPPTATSTTKQAWHIPVLEAEEKPAKVSPPPSFETATFRGKKLPARITLGDGKFKPRHGLYARRNLGASDSHSGYME
>DmelIR25a
MILMNPKTSKILWLLGFLSLLSSFSLEIAAQTTQNINVLFINEVDNEPAAKAVEVVLTYLKKNIRYGLSVQLDSIEANKSDAKVLLE
AICNKYATSIEKKQTPHLILDTTKSGIASETVKSFTQALGLPTISASYGQQGDLRQWRDLDEAKQKYLLQVMPPADIIPEAIRSIVIH
MNITNAAILYDDSFVMDHKYKSLLQNIQTRHVITAIAKDGKREREEQIEKLRNLDINNFFILGTLQSIRMVLESVKPAYFERNFAW
HAITQNEGEISSQRDNATIMFMKPMAYTQYRDRLGLLRTTYNLNEEPQLSSAFYFDLALRSFLTIKEMLQSGAWPKDMEYLNCD
DFQGGNTPQRNLDLRDYFTKITEPTSYGTFDLVTQSTQPFNGHSFMKFEMDINVLQIRGGSSVNSKSIGKWISGLNSELIVKDEEQ
MKNLTADTVYRIFTVVQAPFIMRDETAPKGYKGYCIDLINEIAAIVHFDYTIQEVEDGKFGNMDENGQWNGIVKKLMDKQADIG
LGSMSVMAEREIVIDFTVPYYDLVGITIMMQRPSSPSSLFKFLTVLETNVWLCILAAYFFTSFLMWIFDRWSPYSYQNNREKYKD
DEEKREFNLKECLWFCMTSLTPQGGGEAPKNLSGRLVAATWWLFGFIIIASYTANLAAFLTVSRLDTPVESLDDLAKQYKILYAPL
NGSSAMTYFERMSNIEQMFYEIWKDLSLNDSLTAVERSKLAVWDYPVSDKYTKMWQAMQEAKLPATLDEAVARVRNSTAATGF
AFLGDATDIRYLQLTNCDLQVVGEEFSRKPYAIAVQQGSHLKDQFNNAILTLLNKRQLEKLKEKWWKNDEALAKCDKPEDQSD
GISIQNIGGVFIVIFVGIGMACITLVFEYWWYRYRKNPRIIDVAEANAERSNAADHPGKLVDGVILGHSGEKFEKSKAALRPRFNQ
YPATFKPRF
>DmelIR21a
MSYYWVALVLFTAQAFSIEGDRSASYQEKCISRRLINHYQLNKEIFGVGMCDGNNENEFRQKRRIVPTFQGNPRPRGELLASKFH
VNSYNFEQTNSLVGLVNKIAQEYLNKCPPVIYYDSFVEKSDGLILENLFKTIPITFYHGEINADYEAKNKRFTSHIDCNCKSYILFLS
DPLMTRKILGPQTESRVVLVSRSTQWRLRDFLSSELSSNIVNLLVIGESLMADPMRERPYVLYTHKLYADGLGSNTPVVLTSWIKG
ALSRPHINLFPSKFQFGFAGHRFQISAANQPPFIFRIRTLDSSGMGQLRWDGVEFRLLTMISKRLNFSIDITETPTRSNTRGVVDTIQ
EQIIERTVDIGMSGIYITQERLMDSAMSVGHSPDCAAFITLASKALPKYRAIMGPFQWPVWVALICVYLGGIFPIVFTDRLTLSHLM
GNWGEVENMFWYVFGMFTNAFSFTGKYSWSNTRKNSTRLLIGAYWLFTIIITSCYTGSIIAFVTLPAFPDTVDSVLDLLGLFFRVG
TLNNGGWETWFQNSTHIPTSRLYKKMEFVGSVDEGIGNVTQSFFWNYAFLGSKAQLEYLVQSNFSDENISRRSALHLSEECFALF
QIGFLFPRESVYKIKIDSMILLAQQSGLIAKINNEVSWVMQRSSSGRLLQASSSNSLREIIQEERQLTTADTEGMFLLMALGYFLGA
TALVSEIVGGITNKCRQIIKRSRKSAASSWSSASSGSMLRTNAEQLSHDKRKANRREAAEVAQKMSFGMRELNLTRATLREIYGS
YGAPETDHGQLDIVHTEFPNSSAKLNNIEDEESREALESLQRLDEFMDQMDNDGNPSSHTFRIDN
>DmelIR31a
MNLLISMFILILAAGEGEIIPSMEESVVTNFVKSLVKTKQAIVFSCLFKDFKEISLALMRINQFVSVVNLNQSYSLTSILTRENYARTS
VMVNARCSGSSELLFEASENRYFNKTYQWFLWGVDLEVQSLFPLNLNYVGPNAQITYVNETADGYAYWDIHSKGRHLKSNLEI
NLIATLINDTLNIARDIFHLQSIDFRGQFNGLTLRGASVIDKEDIISNEQIESILSRPTKDAGVAAFIKYHYELLGLLRERFNFTVNFR
NSRGWAGRLGNTTFRLGLLGIVMRNEADIAASGAFNRINRFAEFDTIHQSWKFETAFLYRYTSDLDTHGKSGNFLSPFSDRVWLF
CLLTLGAFSIIWVLFEIIDYKILRIRVNSQKLEHLNQKSSVICIKTTCIERILQTFGACCQQGLDPNPVDRSVRFLVMTLFLFSLVMYN
YYTSSVVGGLLSSSDQGPSTVDEITASPLKISFEDIGYYKVLFRESQNRSITRLIEKKLSSSRSLNELPIFSHIEDAVPYLKAGGFAFH

CEVVDAYPVISEYFDANEICDLREVSGLMEVEILNWILHKNSQYTEIFKTAMCNAQEKGFVERILRRRQIKKPACQSLYTVYPVSL
SGVLPGFVILICKSINKFS
>DmelIR40a
MHKFLALGLLPYLLGLLNSTRLTFIGNDESDTAIALTQIVRGLQQSSLAILALPSLALSDGVCQKERNVYLDDFLQRLHRSNYKSV
VFSQTELFFQHIEENLQGANECISLILDEPNQLLNSLHDRHLGHRLSLFIFYWGARWPPSSRVIRFREPLRVVVVTRPRKKAFRIYY
NQARPCSDSQLQLVNWYDGDNLGLQRIPLLPTALSVYANFKGRTFRVPVFHSPPWFWVTYCNNSFEEDEEFNSLDSIEKRKVRVT
GGRDHRLLMLLSKHMNFRFKYIEAPGRTQGSMRSEDGKDSNDSFTGGIGLLQSGQQADFFLGDVGLSWERRKAIEFSFFTLADS
GAFATHAPRRLNEALAIMRPFKQDIWPHLILTIIFSGPIFYGIIALPYIWRRRWANSDVEHLGELYIHMTYLKEITPRLLKLKPRTVLS
AHQMPHQLFQKCIWFTLRLFLKQSCNELHNGYRAKFLTIVYWIAATYVLADVYSAQLTSQFARPAREPPINTLQRLQAAMIHDG
YRLYVEKESSSLEMLENGTELFRQLYALMRQQVINDPQGFFIDSVEAGIKLIAEGGEDKAVLGGRETLFFNVQQYGSNNFQLSQK
LYTRYSAVAVQIGCPFLGSLNNVLMQLFESGILDKMTAAEYAKQYQEVEATRIYKGSVQAKNSEAYSRTESYDSTVISPLNLRML
QGAFIALGVGSLAAAALNNTINVRSLNSRDKFICGGPVKIWYYLVLLLWYYFNRGLVGIYQLWHKTSIRNTGKGMPFLGE
>DmelIR64a
MHWWLLVFLPLSCQGLPEHELLELELDYGLAEPQRTSLLQSSLILQFSQDYKHIPRITYFTCQKPHLQTPNQIPNAAEHRDAFAAK
NFQLIKSLYESELFVRIVLLDVLAQSPTSGRPNRPGNGPTGGFSQTPSQAQSNSEWLEGVLRMEALRQIAVVDLACGAVSRRFLEL
ASAKMLYSEKFHWLLIEDFAWHGRTQTAEGSGKRDDGEMEEEEPPGQQIQATDDEDLPSIESFLGGMNLYMNTELTLAKRMSEA
AHYTLFDVWNPGLNYGGHVNLTEIGSFTPTEGIQLHTWFRTTSTVRRRMDMQHARVRCMVVVTNKNMTGTLMYYLTHTMSG
HIDTMNRFNFNLLMAVRDMFNWTFVLSRTTSWGYVKNGRFDGMIGALIRNETDIGGAPIFYWLERHKWIDVAGRSWSSRPCFIF
RHPRSTQKDRIVFLQPFTNDVWILIVGCGVLTVFILWFLTTIEWKLVPHDGSALIKPKGGAPPRHHYQQQQQEQVEAPVRPITAV
SVVVSKEKVEEKQEEYEDSTPIDAGTLWQRCYQKLNKYIKDRKAKQKKAPERVGLFLESVLFFVGIICQQGLGFSTSFVSGRCIVI
TSLLFSFCIYQFYSASIVGTLLMEKPKTIKTLSDLVHSSLKVGMEDILYNRDYFLHTKDPVSMELYAKKITSVPTTKENEADEDEPV
DPNPVSTDPAKSYRDIVHSHETGAHAKDNAASNWLDPETGLLRVKHERFAFHVDVAAAYKIIAETFSEQDICDLTEVSMFPPQKT
VSIMQKNSPMRKVISYGLRRVTETGILTYHFNVWHSRKPPCVKKIETSDLHVDMDTVSSALLILLFSYAITLMILGTEILYSKWHN
RIQLKWVGAT
>DmelIR75a
MQLVQLANFVLDNLVQSRIGFIVLFHCWQSDESLKFAQQFMKPIHPILVYHQFVQMRGVLNWSHLELSYMGHTQPTLAIYVDIK
CDQTQDLLEEASREQIYNQHYHWLLVGNQSKLEFYDLFGLFNISIDADVSYVKEQIQDNNDSVAYAVHDVYNNGKIIGGQLNVT
GSHEMSCDPFVCRRTRHLSSLQKRSKYGNREQLTDVVLRVATVVTQRPLTLSDDELIRFLSQENDTHIDSLARFGFHLTLILRDLL
HCKMKFIFSDSWSKSDVVGGSVGAVVDQTADLTATPSLATEGRLKYLSAIIETGFFRSVCIFRTPHNAGLRGDVFLQPFSPLVWYL
FGGVLSLIGVLLWITFYMECKRMQKRWRLDYLPSLLSTFLISFGAACIQSSSLIPRSAGGRLIYFALFLISFIMYNYYTSVVVSSLLS
SPVKSKIKTMRQLAESSLTVGLEPLPFTKSYLNYSRLPEIHLFIKRKIESQTQNPELWLPAEQGVLRVRDNPGYVYVFETSSGYAYV
ERYFTAQEICDLNEVLFRPEQLFYTHLHRNSTYKELFRLRFLRILETGVYRKQRSYWVHMKLHCVAQNFVITVGMEYVAPLLLM
LICADILVVVILLVELAWKRFFTRHLTFHP
>DmelIR75b
MNFSVLESHFKEAQIFVDADVTYVTHDPFSKNFLLYDVYNKGRQLGGELNITADREIFCNKTNCRVERYLSELYTRSALQHRKSF
TGLTMRATAVVTALPLNVSIKEIFDFMNSKYRIQLDTYARLGYQARQPLRDMLDCKFKYIFRDRWSDGNATGGMIGDLILDKADL
AIAPFIYSFDRALFLQPITKFSVFREICMFRNPRSVSAGLSATEFLQPFSGGVWLTFALLLLLAGCLLWVTFILERRKQWKPSLLTSC
LLSFGAGCIQGAWLTPRSMGGRMAFFALMVTSYLMYNYYTSIVVSKLLGQPIKSNIRTLQQLADSNLDVGIEPTVYTRIYVETSE
EPDVRDLYRKKVLGSKRSPDKIWIPTEAGVLSVRDQEGFVYITGVATGYEFVRKHFLAHQICELNEIPLRDASHTHTVLAKRSPYA
ELIKLSELRMLETGVHFKHERSWMETKLHCYQHNHTVAVGLEYAAPLFIILLGAIILCMGILGLEVIWHRHCTLH
>DmelIR75c
MTSWPLYRLIVFNLLEINLSNLMVFHCWSIKEAFPLVEMLNQNGIFSQYIDVQNPDNLANVHKEYLDSDLVSLNADVTYVSRED
EERFILHDVYNKGSHLGGKLNITVDQTLQCNRSHCQVKEYLSELHLRPRLQHRMDLSSVTFRLAALVSVLPINSSEEELLEFLNSD
RDSHMDSISRIGNRLIMHTQEILGFNVQDAFGGAIGMLTNESAELCTTPFVPSWNRLHYLHPMTEQAQFRAVCMFRTPHNAGIKA

AVFLEPFMPSVWFAFAGLLIFAGVLLWMIFHLERHWMQRCLDFIPSLLSSCLISFGAACIQGSYLMPKSAGGRLAFIAVMLTSFLM
YNYYTSIVVSTLLGSPVRSNIRTIQQLADSSLDVGFDTVPFTKTYLVSSPRPDIRSLYKQKVESKRDPNSVWLSPEEGVIRVRDQPG
FVYTSEASFMYHFVEKHYLPREISDLNEIILRPESAVYGMVHLNSTYRQLLTQLQVRMLETGITSKQSRFFSKTKLHTFSNSFVIQV
GMEYAAPLFISLLVAYFLALLILILEICWARYAKKKFSTIIPQNQ
>DmelIR75d
MKVQVAHWLPLIFFLLVSGTPRVAGSWRSEYSRQDPDPKTRWGNQLPDMLVAYYRHHGVHSLMLVVCHTDIADFRLWKLWQH
FNLNNFYVQVSTESSLRDLQHVDALDEHKDAPPPKSFHANNSTHWETSFLLPALPYKMGILLLEFSSECALNLLRWSAASEHNYF
TTNRFWLLLTEDPGDIDLLEDPEIFIPPDSELRVLHYENVGNFSCSLIDLYKVAAWKPLKRTLVGHNIRNSRHVIHALQHFGSAITY
RQDLEGIVFNSAIVIAFPDLFTNIEDLSLRHIDTISKVNHRLMLELANRLNMSYNTYQTVNYGWRQPNGSFDGLMGRFQRYELDL
AQLAIFMRLDRIALVDFVAETYRVRAGIMFRQPPLSAVANIFAMPFENDVWVSILMLLIITTVVLVLELFFSPHNHDMSYMDTLNF
VWGAMCQQGFYVEVRNRSARIIVFTTFVAALFLFTSFSANIVALLQSPSDAIQSLSDLGQSPLEIGVQDTQYNKIYFTESTDPVTKN
LYHKKIASKGENIYMRPLLGMEKMRTGLFAYQVELQAGYQIVSDTFSEPEKCGLMELEPFQLPMLAIPTRKNFPYKELIRRQLRW
QREVSLVNREERKWIPQKPKCEGGVGGFVSIGITECRYALGIFGCGAAVSFVLFLFEFIFRHFKQVYRIIKGYREVQR
>DmelIR76a
MENLLVESYYFSTVLSFFAQQFFADSHATCIFWHPAFDFRLETVHPMPLIIMDWHRWANRSDQDVYDYKIKEDEFEGKGIPYND
WTLRLTVAIERSHCETFIAFQEQIPEFARYFYHASIYSIWRSLRNRFMFVYTKEFEDKKDSYLSGYIFQDQPNILVITSQYLNSSTFEI
KTNRFVGPRNFNKNPEPVEFYILQRFDAKGTKATWETQSAMSSKMRNLKGREVVIGIFDYKPFMLLDYEKPPLYYDRFMNTTD
VTIDGTDIQLMLIFCELYNCTIQVDTSEPYDWGDIYLNASGYGLVGMILDRRNDYGVGGMYLWYEAYEYMDMTHFLGRSGVTC
LVPAPNRLISWTLLLRPFQFVLWMCVMLCLLLESLALGITRRWEHSSVAAGNSWISSLRFGCISTLKLFVNQSTNYVTSSYALRTV
LVASYMIDIILTTVYSGGLAAILTLPTLEEAADSRQRLFDHKLIWTGTSQAWITTIDERSADPVLLGLMEHYRVYDANLISAFSHTE
QMGFVVERLQFGHLGNTELIENDALKRLKLMVDDIYFAFTVAFVPRLWPHLNAYNDFILAWHSSGFDKFWEWKIAAEYMNAHR
QNRIVASEKTNLDIGPVKLGIDNFIGLILLWCFGMICSLLTFLGELWRGQG
>DmelIR76b
MATGIELLVAAALCVACPPLNDSPPTNLIQMGENGTLSPVTELPMDVDASEAGFDADAPVETLETINRKKPKLREMLDWIGGKHL
RIATLEDFPLSYTEVLENGTRVGHGVSFQIIDFLKKKFNFTYEVVVPQDNIIGSPSDFDRSLIEMVNSSTVDLAAAFIPSLSDQRSFV
YYSTTTLDEGEWIMVMQRPRESASGSGLLAPFEFWVWILILVSLLAVGPIIYALIILRNRLTGDGQQTPYSLGHCAWFVYGALMK
QGSTLSPIADSTRLLFATWWIFITILTSFYTANLTAFLTLSKFTLPYNTVNDILTKNKHFVSMRGGGVEYAIRTTNESLSMLNRMIQN
NYAVFSDETNDTYNLQNYVEKNGYVFVRDRPAINIMLYRDYLYRKTVSFSDEKVHCPFAMAKEPFLKKKRTFAYPIGSNLSQLFD
PELLHLVESGIVKHLSKRNLPSAEICPQDLGGTERQLRNGDLMMTYYIMLAGFATALAVFSTELMFRYVNSRQEANKWARHGIG
RTPNGQSVAPSRWLRGWRRLNSGHGQLLGASTHGQNVTPPPPYQSIFNGGSHGDPLNRWRRPLANGNALGNGVLLGGDSEGGV
RRLINGRDYMVFRNPNGQSQLVPVRSPSAALFQYSYTE
>DmelIR84a
MIKLQVKVISWPLIILTAFLRVLQIESINTNFLELAAFEDFLRSEHLSHVLVVRGDDADGDWKIECHQKLLANYRVQFYRPEMSAN
FEDLMFYGSPRTAVLVLNSEHVLVRRQVFGVASEAGYFNNSLAWFILGSGRESLPVEQLIDQLLSGYRMGIDADITVALRGPDNAS
MLFYDVYRISRQANTPLIIEKKGLWTHSGGYQKFGNFKNTWVIRRRNFLNVTLIGSTVLTEKPPGFGDMEYLADDKQLQQLDPM
QRKTYQLFQLVERMFNLSLAISLTDKWGELLDNGSWSGVMGQVTSREADFAVCPIRFVLDRQPYVQYSAVLHTQNIHFLFRHPR
RSHIKNIFFEPLSNQVWWCVLALVTGSTILLLFHVRLERMLSNMENRFSFVWFTMLETYLQQGPANEIFRLFSTRLLISLSCIFSFM
LMQFYGAFIVGSLLSESARSIVNLQALYDSNLAIGMENISYNFPIFTNTSNQLVRDVYVKKICKSGEHNIMSLQQGAERIIQGRFAF
HTAIDRMYRLLLELQMDEAEFCDLQEVMFNLPYDSGSVMPKGSPWREHLAHALLHFRATGLLQYNDKKWMVRRPDCSLFKTS
QAEVDLEHFAPALFALALAMVASALVFLLELFLHWLPDFRRRLGTMST
>DmelIR92a
MLLQPLVMHLSQLLRIIVGQYFAEFPSILIVYNNSASTTPLQLEYLSALELVLRELSKPIRLQWINVAFLKDLNDLEDQVMGALNSS
VTEGFITILSQTHHFIHARYYATRNANVRLKDKRYLFLCEDESPAELLCMDILQFYPHHLMVRPGTETAPTGPTGPHPDPRRGGGA
SVSTKNKDDGEGGAGNKTTSPYRDINFELWTQKFVGAVGNLDALLLDAFLPNETFANRVELYPNKLLNLQRRSLLVGSITYVPYT

ITNYVPAGQGDVDPIHPQWPNRSLTFDGAEANVMKTFCQVHNCHLRVEAYGADNWGGIYDNESSDGMLGDIYEQRVEMAIGCI
YNWYDGITETSHTIARSSVTILGPAPAPLPSWRTNIMPFNNRAWLVLISTLVICGTFLYFMKYVSYRLRYSGTQVKFHHSRKLEKS
MLDIFALFIQQPSAPLSFDRFAPRFFLATILCATITLENIYSGQLKSMLTFPFYSAPVDTIEKWAQSGWKWSAPSIIWVHTVQSSDLE
TEQILARNFEVHDYSYLSNVSFMPNYGFGIERLSSGSLSVGDYVSTEALENRIVLHDDLYFDYTRAVSIRGWILMPELNKHIRTCQ
ETGLYFHWELEFIDKYMDKKKQEVLMDLANGHKVKGAPQALDVRNIAGALFVLAFGVAFAGCALVAELLIHRMDLSK
>DmelIR93a
MNPGEMRPSACLLLLAGLQLSILVPTEANDFSSFLSANASLAVVVDHEYMTVHGENILAHFEKILSDVIRENLRNGGINVKYFSW
NAVRLKKDFLAAITVTDCENTWNFYKNTQETSILLIAITDSDCPRLPLNRALMTVECRINAVVFVDQTILEENALLVKSIVHESITN
HITPISLILYEINDSLRGQQKRVALRQALSQFAPKKHEEMRQQFLVISAFHEDIIEIAETLNMFHVGNQWMIFVLDMVARDFDAGT
VTINLDEGANIAFALNETDPNCQDSLNCTISEISLALVNAISKITVEEESIYGEISDEEWEAIRFTKQEKQAEILEYMKEFLKTNAKC
SSCARWRVETAITWGKSQENRKFRSTPQRDAKNRNFEFINIGYWTPVLGFVCQELAFPHIEHHFRNITMDILTVHNPPWQILTKNS
NGVIVEHKGIVMEIVKELSRALNFSYYLHEASAWKEEDSLSTSAGGNESDELVGSMTFRIPYRVVEMVQGNQFFIAAVAATVEDP
DQKPFNYTQPISVQKYSFITRKPDEVSRIYLFTAPFTVETWFCLMGIILLTAPTLYAINRLAPLKEMRIVGLSTVKSCFWYIFGALLQ
QGGMYLPTADSGRLVVGFWWIVVIVLVTTYCGNLVAFLTFPKFQPGVDYLNQLEDHKDIVQYGLRNGTFFERYVQSTTREDFKH
YLERAKIYGSAQEEDIEAVKRGERINIDWRINLQLIVQRHFEREKECHFALGRESFVDEQIAMIVPAQSAYLHLVNRHIKSMFRMG
FIERWHQMNLPSAGKCNGKSAQRQVTNHKVNMDDMQGCFLVLLLGFTLALLIVCGEFWYRRFRASRKRRQFTN
>DmelIR7a
MFHHLWLLMGLRSLAMGALHPPQPEAMTPLVAAALEILAEQVSPSQSTLAVMDLTQDAEHRDERQEQLMTIILRSVGSEMALRT
FQKPPAEVPASFVVFLVNSAQAFNTLGFHFTDIHSTREFNFLILLTHRMSSRAERLQVLRDISRTCVRFHTSNVILLTEKRDGVVLV
YAYRLLNMDCDLSVNLELIDIYKNGLFRHGHEARSFNRVLSLSGCPLQVSWYPLPPFVSFIGNSSDPEERAQIWRLTGIDGELIKLL
ASIFDFRILLEEPCNKCLSPDIKDDCSGCFDQVIISNSSILIGAMSGSHQHRSHFSFTSSYHQSSLVFIMHMSSQFGAVAQLAVPFTVI
VWLALVVSSLLLVLVLWMRNRLVCGRSDLASHALQVLTTLMGNPLEARSLPRSSRLRILYAGWLLLVLVLRVVYQGKLFDSFRL
PYHKPLPTEISELIRSNYTLINQEYLDYYPRELTVLTRNGSKDRFDYIQGLGKEGKFTTTSLIATMEYYNMMHWSTSRLTHIKEHIF
LYQMVIYLRRHSLLKFAFDRKIKQLLSAGIIGYFVREFDACQYRKPFEEDYEVTPIPLDSFCGLYYISLIWLSAAVVAFILELLSQRI
VWLRRIFE
>DmelIR7b
MKYWLYILSCCSLVASTMESSSDWDLAEALAQVVANSEMGRFKTLYIYTHTNSQSTGGHLEELLDQVLMIVPNNLQARRLLLQQ
SMEYKPYVHAVLALVDGLPSLSAIYARIRATQDLSHTLIYMSMPTDAYGEEMQATLRFLWRLSVLNVGVVLRPPGDHILMVSYFP
FSALHGCQVISANVVNRYQVGTKRWASQDYFPSKLGNFYGCLLTCATWEDMPYLVWRPDGSGSFVGIEGALLQFMAENLNFTV
GLYWMNKEEVLATFDESGRIFDEIFGHHADFSLGGFHFKPSAGSEIPYSQSTYYFMSHIMLVTNLQSAYSAYEKLSFPFTPLLWRAI
GLVLILACLLLMLLVRWRHHHELPRNPYYELLVLTMGGNLEDRWVPQRFPSRLVLLTWLFATLVLRSGYQSGMYQLLRQDTQRN
PPQTISEVLAQHFTIQLAEVNEARILASLPELRPEQLVYLEGSELQSFPALAQQSGSSARVAILTPYEYFGYFRKVHPMSRRLHLVRE
RIYTQQLAFYVRRHSHLVGVLNKQIQHAHTHGFLEHWTRQYVSAVDEKDESVARIASTSYSTLDGIDGDPSLSESEEDQQVAPVR
QNVLSMRELAALFWLILWANLGAVVVFVLELLLPRIKLRKILRKMKKSTRASATTTSTLSSPSTTKDIPFSCKDGFQDSWPKCSLL
VS
>DmelIR7c
MLHSAVHNVSLVYALVWAIDNYYGMATSTPLAVVQFPTSRESRRLHNDLIDAALGRSSGTGRIQFLLEDDRVEMTETDTDPPPPS
GLTGRPIAIWFLDSLRSYFRLEMYLNQLGSPYKRNGFFLVIYTGLEDQPMESLKIMFRRLLNMYVLNVNVFLQRDGTVHLYTYY
PYGPHHCQSSLPVYYTAFQDLAAPANGFGLTKPLFPRKLTNMHGCEMVVATFEHRPYVIIEDDPKTPGGRSIHGIEGLIFRSLAER
MNFTIKLVEQKDKNRGEILPDGNFTGILKMMVDGEVNLTFVCFMYSKARSDLMLPSTSYTSFPIVLVVPSGGSISPMGRLTRPFRY
IIWSCILVSLIFGFVLICLLKITALPGLRNLVLGRRNRLPFMGMWASLLGGLALYNPQRNFARYILVMWLLQTLILRAAYTGQLYLL
LQDVEMRSPIKSLSEVLAKDYEFRILPALRTIFKDSMPTTNFHAVLSLEESLYRLRDEDDPGITVALLQPTVNQFDFRSGPNKRHLT
VLPDPLMTAPLTFYMRPHSYFKRRIDRLIMAMMSSGIVARYRKMYMDRIKRVSKRRNLEPKPLSIWRLSGIFVCCAGLYLVALIVF
ILEILTTNHRRLRRAFNVINRYAA

>DmelIR7d
MDIRCVVALLLGLCKVQAVVWPHQHLLEEQLASQISATLQKIFINGLAVYNFGVFISTSYEEMDRDRVILVHQVLNRNLYPPNFPV
AVVLASKMNRKITAQVFTQLLFVQNAEQAIAIAEGVNRNGLCVIVLLTSQPERPIMTKIFTYFMQERYNINVVILVPRLHGVQAFN
VRPYTPTSCSSLEPVEIDIKDGDLWDVFPRRLKNLHGCPLSVIVWDIPPYMRINWKSSDPMDGLDGLDGLLLRIVARKMNFTLKLI
PNEPNGLIGGSSFMNGTFTGAYKMLRERRANITIGCAACTPERSTFLEATSPYSQMSYIIVLQARGGYSIYEVMLFPFEKYTWLLL
STILGLHWIVGSRWRMPSPILAGWMLWIFVIRASYEASVFNFIQNSPVKPSPRTLDQALSGGFRFITDHASYRMTLKIPSFQGKTLI
SAGQPVDVFDALLKAPWKTGAFTSRAFLADHLVRHRKHRNQLVILAEKIVDNMLCMYFPHGSYFAWEINKLLFNMRSFGIFQH
HSQILAWDNLPTTTDTDTPGKRIHSSTESVATGFAESMSFVVAALNCLMGALCISIVVFGLELLSRRRHWTGLEWLFERV
>DmelIR7e
MNHINEFVARAVLHVVHHYILSVTPSLVLTLCCRSNHTCNFYNKMMSTLFREWGLAPLQIVNVLRGVPWHPVPGRRHFNVIFTD
SFAAFEEIRMEYYSREYNYNEHYFIFLQARDRLLQGEMRLIFDYCWRYRLIHCSIQVQKSNGDILFYSYYPFGEHGCSDMEPQLIN
RYNGSMLVEPDLFPRKLRNFFGCPLRCALWDVPPFLTLDEDQEEVLRVNGGYEGRLLLLALAEKMNFTIAVRKVHVNMRDEALE
MLRRDEVDLTLGGIRQTVARGMVATSSHNYHQTREVFGVLASSYELSSFDILFYPYRLQIWMGILGVVALSALIQLIVGRMLRER
MGSRFWLNLELVFVGMPLLECPRSHTARLYCVMLMMYTLIIRTIYQGLLYHLIRTHQLNRWPQTIESLVQKNFTVVLTPIVQEVLD
EIPSVQHMRFRLLEANSELDPLYFLEANHQLRQHVTASALDIFIHFNRLSADKVHQRGEQGSGAHFEIVPEDIISMQLTMYLAKHS
FLIDQLNEEIMWMRSVGLLSVWSRWELSESYLRNEQSFQVLGTMELYAIFLMVLVGLIVGLLVFILELVSMRSIYLRKLFT
>DmelIR7f
MQGEDANLYVARALRLVIENVLAQLSTTLVVTISTRHLGTAHWFEYMMNILMDSWRMVAVQLLRIRPDLVVNPVPGRKRVSLL
MVDSYQGLLDTNITASNANFDDPDYYFIFLQARDHLIPKELQLILDHCLAHFWLHCNVMIQTAQVEVLVYTYYPYTADACQKAY
PIPVNTFDGRKWKASQMFPDKLSQMHGCPLTVLTWHQPPFVELVWDPKHNRSRGSGFEIQLVEHLARRMNFSLELVNIALLRPN
AYRLAEGSSEGPIEKLLQRNVNISMGYFRKTARRNQLLTTPMSYYSANLVAVLQLERYRIGSLALLVFPFELSVWMLLLLALLIHL
GIHLPSARRGNEEDGGGGLQVVALLLGAALARLPRSWRHRFIAAHWLWASIPLRISYQSLLFHLIRLQLYNTPSFSLDQLLAEGFQ
GICTANTQRLLLEMPQLARDPDSIQSVDTPFDWDVLNVLTRNRNRKIFAVANQDVTLSFLHSSAHPNAFHVVKQPVNVEYAGMY
MPKHSFLYEKMDDDIRRLDASGFIHAWRRASFASVHRKEQVHMTSRRYINHAKLSGIYMVMAGLYLLAGLLFAGEVLLRQRN
>DmelIR7g
MNVTSLLNFESMKYIGAQTQAASINHHVAQALRVFIEDFYQRIAPAFIVVLSCRRPSPMNFYRNIMQLLYESVDTMIVQLVLVELG
RPRRIAGPRTHNLLLVDSLDALLDIEIHTYTAQSDTSEYYFIFLQQRDALIPHDMQGVFAYCWRHQLINCNVMTQSSGGQVLLHT
YFPYAPGQCNDSQPTRINMFLGESWKHRDYFPSKLHNLNGCPLIVLARKVSPFLDLDEGQRELRGLEGRLLQELSRRMNFSIQFS
GLQDQLKNRTTWTEKQLLQKLVQERIAHLAIGYVRKRIQYATNLTPVFPHYSNRVVGCLLLNAHNLTSLEIWSFPFQALTWICLVA
GDRLALVLAVYAASLGLPIDPPERPSLQLLFASWLIFGLIVRSMYSALLFFILRYHLHQRLPGNLQDLTHGDYAAVMGRTTLQDLR
EVPSLQDLLGLKSVIVTSEREEEVLRTLDRCTLREGAGSHPLFFGLISQDALLHLTQRGHRAGAYHIIPQDVLEQQLAIYLQKHSH
LASHLDHLVMSIRSVGLVHHWAGQMASERYFRSRFLYREKRIRQPDLWAVYILTAGLYLLSLVVFICELLASRRAGL
>DmelIR10a
MAVLGTVFLLFMLDLKTLNLTRLNGLLVEPTRDLPQLELWLRAGSDHQDAENPYVQWFLLRTEIPLSIVTYQENRYWMDDPFGR
RNLVLVMSLDQLLTNRGAAAPIQKASTFFYILADQDKDLSADEQLRLEGSCRQLWTQHKVYNRFFLTRDGVWIYDPFKRRDSAF
GRLVRYYGSETLDKLLFRDMAGYPLRIQMFRSVYTRPEFDKETGLLTRVTGVDFLVAQMLRERLNFTMLLQQPEKKYFGERSAN
GSYNGAIGSIIKDGLDICLTGFFVKDYLVQQYMDFTVAVYDDELCIYVPKASRIPQSILPIFAVGYDIWLGFVLTAFACALIWLTLRV
INLKLRIVSLGNQHIVGQALGIMVDTWVVWVRLNLSHLPASYAERMFIGTLCLVSVIFGAIFESSLATVYIHPLYYKDINTMQELD
ESGLKVVYKYSSMADDLFFSETSPXWNRDLRADVIDEVARFRNKAGVSRYTSLILESSHFTLLRKIWVVPECPKYYTISYVMPRD
SPWEDAVNALLLRFLNAGLIVKWIQDEKSWVDIKMRSNILEADAESELVRVLTIGDLQLAFYVVIGGNLLAFLGFLAEHFRWKLQ
KKGV
>DmelIR11a
MRFAILWLFSGCLLPGIQVGIWVVVRAQPTGRDVLLSRLGNQQNELNTRRLANASSYLTRNYIANRINTLVVREICVECPYELSER
QRQLVDQILASLAPELSVLLHKGTAEETTWEYTLFVVNDHTAFTGQVFIFPDELLEREFFCIVVVSEIQSRQFVRQTVGSIVKSNLQ

MHFVNVVVVAQLEDGTVGTYSYKLFKANCTPGITVRQINHFDRITGKPQQSMPDLYPVRNGHLGDCPFNVGAAHMPPHLIYKR
HKDPPPASNVSIPAEDLAGIDWDLLQLLAKALKFRIQLYMPQEPSQIFGEGNVSGCFRQLADGTVSIAIGGLSGSDKRRSLFSKSTV
YHQSNFVMVVRRDRYLGRLGPLILPFRGKLWGVIIVILLLAVLSTCWLRSRLGLSHPIEDLLTVIVGNPIPDHRLPGKGFLRYLLAS
WMLLTLVLRCAYQARLFDVLRLSRHRPLPKDLSGLIKDNYTMVANGYHDFYPLELTCRQPLDFSARFERVQRAAPDERLTTIALI
SNLAYWNHKHPNISRLTFVRQPIYMYHLVIYFPRRFFLRPAIDRKIKQLLSAGVMAHIERRYMQYENKRKVASNDPVLLRRITKSI
MNGAYRIHGLVIVLATGMFILELLAGRSNGRLRRWMEWVHQ
>DmelIR20a
MLASLNRSTGLSAELLDLYGLVVHFLLSGEHTTLVYFNPAGLDCSWGVLWQRNLTAHPQIVWQRNYSYPDLYYQFNAKLLVLA
CLPMDSRAAIQLEILANSLSHLRTVVRLLIEVAGPDQVTLARQYLSFCLRRSMLHVELYFRDYHHSLILYSFRAFPSFELVMRWISV
GQGVKLFLHKLDDLRGHRLRVIPDLSPPNTFFYRDARGDNQVTGYLWDFLATFAGRLNAGLEVVRPSWRAGSASDSSYMLEYS
AKGLIDVGLTTTLITKWNLWAIHQYTYPLLVSSWCTMLPVEKPLATPDLFGRIVCPTLAMTLLLIILVTWLVFRQLRCLTRLKNSRP
ARIVPHLLTLLLLTTCSAQLLSLLIFPPYHVRIASFEDLLRGDQKILGMRNEFYNFDGAFRARYAGVFYLIDDPNELYDLRNHFNTT
WAYTMPYIKWLVIKTQQRHFSKPLFRWSKDLCFFDFMPTSVIVAPDSIYWESIKDFTFRIHQAGLMKHWIRKSFYDMIKAGKMSI
KDYSDLETLKPLNIGDLEIVWRVCGAAIAVASAIFIMELLYFYINVFFNSL
>DmelIR41a
MFIDLSWSLVLSAIVGKYLNESTICIFWNDKFEFQLLHKSDYISFVGINIKSFDDNGGHYIIDTGLKKKELQNKHLFLDELVIKIIISI
EVTHCETFVVFDKDIDRFVNAFNKASVYSIWRSLHNKFVFAHIANESPESRNHFFEDQPNILFVVRDHSSASSFDIKTNKFVGRKA
ENPSQMILVDRYLASEQRFQFGKSLFADKLNNLQGREVIIAGFDYPPYTVIKHNMSTNAQDMGVSGESDFKNVYIDGTETRIVLN
FCEQFNCTIQIDSSAANDWGKVYPNMSGDGALGMLINRKADICIGAMYSWYEDYTYLDLSMYLVRSGITCLVPAPLRLTSWYLP
LEPFKETLWAAILLCLCAEATGLVLAYKSEQALYVLPGYREGWWTCTSFGVCTTFKLFISQSGNSKAYSLTVRVLLFACFLNDLIIT
SIYGGGLASILTIPSMDEAADTVTRLRFHRLQWAANSEAWVSAIRASDEALVKDILYNFHIYSDDELLRLAQDQHMRIGFTVERLP
FGHFAIGNYLGPQAIDQLVIMKDDIYFQYTVAFVPRLWPLLDKLNTLIYSWHSSGFDKYWEYRVVADNLNLKIQQQVQETMTGT
KDIGPVPLGMSNFAGFIIVWILGSAIATLTFLLELSLTYILKQSNLK
>DmelIR47a
MRQIKLLVWLLVVGVVSSTEQLQFLKNFLEAVHKERSISTILLIQRKVHKNDFLHGLYPIFWPIICLDETKRVELVNNFNKDFLALV
YMESEADTLLLSALAADLNHIRDARIMIWLQMSPSENFLDRIVFQASKQKFLNLVVIENTLKTRRFYPFPQPKVQVIDKPFEEKEI
YPALWRNFMGKNAIAVPDLVPPRSFNSFDPKTGHRRESGSIYNVFKAFTQRYNITMLLKWPLIRNTTQEEIIGKSVRGEIDLPITGQ
LISFRHPNGSRSQPLLGMTALSIAVPCGPELPMFDRFFLFYGLATPITITGYYVLLNTIEIILGTLSDRIKRHPRRKKILNLVLNLRVFS
CILSLPTPQGNRLRSVKGQLTMVMSITGLILSCIVAAQTSTILTMKPQYRHIKNFQELSDSNITVVCNHLNYLTIKQQMDPKFMAK
FMQNIWIVNSIEQMKMIFDLNTSYAYQTFSYKKDPFTLLQMHTTRKAFCRTPGLDLVSGLAYTAVLEKNSIYALALQDYTLKAFS
AGLVYYWAEESIRDLISTVGRTQFEKLPIVIGYQSLKLQDYNVCWKILLIGGALAFCVFIVEVVVGLINRRI
>DmelIR47b
MREAQIIIFLLTSAAAVTLKQYEFLXSFLKAGEQEQTITTLLMMQKHVHTKNLLQGLYPXPWPIIHFVETQRIKFIALLYMSSEKDI
FLSSLAANLKFERLDKPFGKSNIFPVLWRNYMGXIALTLDHLVEPRSFYWTDPRTNIKRRTGYIYMLITNFAEQHNITLQLXSPPNE
DMSQMVIIERTHKGPRSTHNWADDQLETFERXQDSLLPWHGSMAIVVPCGQEMSAYERFHAAHAFRAPIIFFGFHIFLSLIDFLLR
TISDRIRCNPRRIQLLQTVLSLCVLRCILSTSLPNSNXLRSRLRDNSPXXXVLQAXSYSALWXLTGTAXQXHNRDFQSHKLHDYX
TTDGSXHSIEVPGLLKARNXXIXLFHIFSSLGTKFDLRIGSAGSHTSGVEFRYYELLDRXSSLENNIVSQVFTILKLPYSRFRVLKLE
DCRGCWQTLFVGFSIATFVFIVNVLMGFFRNINQKK
>DmelIR48a
MHLLITETYMIIGKTLHDILNELNERLIISTNIIFCKQFDNLIHFEAQTSRFVYSSLEAFNITSLWNHVGNDNKLFVIVGNVPPYELFA
KLELSSPENCTQFILNNTVDMCADALVKNSKAFSVSRELRIAPANVIVPHGKPLLSYRYLAAPFNTKVWIALGTYVFLISGFLCLI
HWLRSGKWDFSQNLLEVYSSLLFTVFHLKATNGIERYILFGVLFISGFVYSTSYLRLLKSMLIAETFEKQIQTFEELAESNIPLLINP
YDRMIFQHHHIPKSLWTAVRTVSSETLLNHRSHGYVRLCPAILTASKIPSHTHRHLFSVCRFSHEQEVVPKGSSXXSLVPCIRKRNR
EXNHLGCLSGVSWPGISXFFHYGALGGEAFGSILLHDANYFPSPRLFRRLAELHYGSY

>DmelIR48b
MILQQSSNLLKLLLLLAISSVRTQGLNDIIIELNQRLLISNNFLYCNQSDKLNEYEIKYLQHMPPISLMIFTSIESMNFTQVEYNLGA
DNKLFLIMGNEEPPYDFLHALNLHFQFAEYIIVIDEPVDLKKSTKWLDFVNHLWQQGYVQLLIYTSYDEKLYHKIIFPETVIEETLV
EQYISIRGSFNNLYGYPVRVAAYNNAPRSMLYVNRWGKHIFAGFYMRFLRAFIDARNGSFVPVLTPSNSPGNCTLNLVNETVDVC
ADALAANPAAFSLTHGFRIASANVLVTHAKPLHSYRYLTAPFQWSVWACLVIYVLLVVNFLSFIGWLRSGKWEFSKYLLEVFSSL
LFSGFYLKEIRGRERYILFGVLFIAGFVYSTEYLGLLKSMLISEVFEKQIDTFEALVESNITLMVDPYDKILFAKYNMPEILSPIMELV
SFETLLKHRNRFDQDYAYILFSDRMALYDYAQQFLKHPKLLRIPIDFSFLYTGIPMRKRWFLKHHLGRAWYWAFESGLTRKLALD
ADFEAVRVGYLSFLITEHVEAQPLNVDYFVMPAIALAIGYILALLSFVIEMTAWRIREFLGCRKATMTSTGCSEGGHVDVD
>DmelIR48c
MSLLRIILIIIFLRIVSSIPDTIISHLSAELQIKIQIYFGLGNDLYDFSRLDGNYQKIIISHNISEEFKTYHDEPVLIIIRLERDLNLNLATLD
VLRSYLTDRQYNDILLIDNDEENLNSYVDIRKAYWNAGFSQVLIYNSQQRTWSIKPYPYLQIRPTSLKEYIENRNTRNLMGYPLR
VLVTNDPPHCFVDKDELPGSPNRYKGSIVTMLKIFADQLNATFQANPFREFRRYSTADCVQMVSDDEIDACGSIFIRTYTYATSQP
VRLNRVVIMAPFGNPIEKFYYFFRPFDLYVWIGTGIIVVYIAVMGSLLHRWHFKEWNVGQYLLLAVQTLLNRELSLPQSSSGSKF
MLLLLLFAIGFILSNLYVALLSMMLTTKLYQRPIENLADLKAANVNILLQTHNIRPNSVYGSSEELRERFLLVEESQHLEKRNGLDP
SYAYVDSEDRMDFYLYQQKFLRRRRMKKLSNPVGYTWAVQVIKQNWVLEKHYNDHVQRFFETGLQNKLVDDVHELAVKAGF
LHFFPTQTQTIEPLRLEDIVMAAMVLGGGHALAVICFLVELFA
>DmelIR51a
MYNVLVLFLLLFTRAQMEPHRRGHNMTLLRSVLTVIRGRENWKNTPIFLGGHCNSDDLNNLMSWLQNTMEVTCHTVDTSTSA
KNENALGHFNINADNSLGLLFCQSSHELIWFNMDKRLRRLRGIRLIVILSDKRSSSSKAIMSTFKRLWHFQFQXNFQGYVVSTPV
ENDIPRVFFVKDKKTGRKQIRGFGYRTFVEYLHRYNASLHVSNSQQEHAINSSVNMGRIINQIVDGQLEISLHPYVDVPENMGDN
SYPLLIASNCLIVPVRNEISRYMYLLLPLNQSSWILLLGSVIYISGVLYYIQPGLLHRTWDQRIGLNILDSISRIINICSPSRIYNPSLRY
FIVSVHLSILGFVVTNLYSIMLGSFFTTLVVGEQVDSMQQLIQXQQKVLVKYYEVSTFLRHVEPDLVDGVAQLLVGVNASEQVSA
LLGFNRSYAYPFTLERWEFFSLQQQYAFKPIFRFSSACLGSPIIGYPMKSDCHLQSSLNMFIMRIQAAGLLRHWVVSDFNDAMRA
GYVRLLENFLGFHSLDVDSLRLRWAVLLCGWLLSTLIFLCER
>DmelIR51b
MCKVLTLLVVILLLALTNAAYNVTLLKSVLSLISTREPWINTPIFVGHNTQGGDLNDLIIWLHQTMGVTSLTMNLFLQPEHIRPLG
HFKITRYNGIALFFCHDKHDIMWLTLDRNLRKLRRIRLIIILRNQRSGSQGAIKSIFNALWQYQFLNVLVLQRDQLYSYTPYPAMRF
FKLDIHTEPLFPHAARNFHGYVVSTPAENDIPRVFHVHDPLTKSRKVLGYAYRTFVEYLDHYNASLRLTNPDENLDPTTSVNMNH
IVQLIIDGQLEISLHPYVFTPPTATKSYPLLIYPNCLIVPMRNEIPRHMYLLRPFQLYSWYILLFAVFYITGILYCISPKLNKSSWPQRL
GLNFLDAISKILFISPPITIYRPTWRHLIIFLQLSVLGFMSTSWYNIELDSFFTTIVVGEQVNSMDQLVHQQQRVLVKEYEINTFLRH
VEPRLVEKVSRLLVPVNASEQVSALLSFNRSFAYPFTEERWQFFAMQQQYAFKPIFRFSSACLGSPHIGYPMRVDSHLETSLNHFIL
KIQDTGLLNHWVVSDFNDAMRAGYVRFVDNVLGYQSIDVDTLRLGWCVLGIGWILSALVFSCEYWHLYPWRFIA
>DmelIR52a
MALGWSVIILGFIGQLSAQILNYTQSRDLELLEGSLFRVLSRLNLEEEYNTLLIYGKECVFHSLLRKLEISAVTVPSGSTDYDWSFS
TAILILSCGYDAENEENSYTLMKLQRTRRLIYLEDNSEPESVCMRYSLKEQHNIAMVKSDFDQSDTFYSCRLFQTPNYVEGHFFK
DQPIYIENFQNMRGATIRTVADSLVPRTILYRDEKSGETKMMGYLGHMINTYAQKLNAKLHFIDTSKLGAKKPSVLDIMNWVNE
DIVDIGTALASSLQFKNMDSVWYPYLLTGYCLMVPVPAKMPYNLVYSMIVDPLVLSIIFVMLCLFSVLIIYTQHLSWKNLTLANIL
LNDKSLRGLLGQSFPFPPNPSKHLKLIIFVLCFASVMITTMYEAYLQSYFTQPPSEPYIRSFRDIGNSSLKMAISRLEVNVLTSLNNS
HFREISEDHLLIFDDLSEYLVLRDSFNTSFIFPVSVDRWNGYEEQQKLFAEPAFYLATNLCFNQFMLFSPPLRRYLPHRHLFEDHM
MRQHEFGLVTFWKSQSFIEMVRLGLASMEDLSRKRNEEVSLLLDDISWILKLYLGAMFISSFCFILEILRCGERCKRLWRCRW
>DmelIR52b
MTWLVILLCFLGYMAAHIADISVQNQSLMDNELINLLLKLRNEEFYDTLLVYGKDCEFHSVIKNVDVAVVLVSDSMNFEWNFSS
LTLILSCGPDIDNGGPNSTSIKLQRNRRLVLLKEDFQPSNICNIYTQKEQYNIALVRENFTKSKSIYTCRYFQDPNVDEVNLSGTKPI
FIEQFQNMKGKAIRIVPDLLPPRVMLYQDANDGELKMIGYVANLITNFAQKVNATLQLDFLKPSTSITEISRMAKDDELDMGITLE

ASLNTSNLETSSYPYLLTSYCLMVQVPAKFPYNLVYALIVDPLVLGIIFVLFLLLSVLLIYSQKMSWQDLSVANILLNDKSLRGLLG
QSFPFPLNASKKLRLIFTILCFASIMLTTMYEAYLQSFFTNPPSEPEICSFQDVGSYNRRIAMSALEVNGLIKTNNSHFREIRMDDLEI
FDNMPECYELRDAFNLSYNYVVTGDRWRSYAEQQTLFKEPVFYFARDLCFSRLIFLSVPLRRHLPYRHLFDEHMMQQHEFGFVN
YWMSHSFFDMVRLGLTSLKDLSRPLAYTPSLLMDDISWIMKIYLAAIVLCVFCFLLEIGVDKWKRWMKFRNLQILNTC
>DmelIR52c
MVWLIIILFCLGNSSSQILDVTNNSHLDFDYRLFGLLQRLQVEKSYDTLLVYGEDCAIPSLFERLQVPAVLVSSGSTNFDWNFSSLT
LILSCNFQDEREENYRTLMKLQTSRRLILLKGHIKPESVCDFYSKKEQHNVAMVKENFYQLEVVYSCRLFQDQNYEKLNLFDGK
SIYKDQFRNMHGAPIRTLSDKEPPRTIPYIDSKTGEEKFKGYVGMLISQFVKKVNATMQIREDLIKDDEEVSFVDITNFTSNDILDI
GICEARTLEMSNYDAISYPYLMSSYCFMAPLPDSLPFSDVYMAIVAPSILIMFLIIFCICSVLIIYIQERSYRSLTIRSVLMNDICLRGF
LAQPFPFPRQYNRKLKLIFMLVCFSSLISTTMYTAYLQAFLWGPPIEPRLTSFDDVKKSRYTMAINIYEREFLEALNVSLEDVEIYD
YGKFSKLRSTFNTNYLFPVTALQWFTINEEQKLFKYKIFYYCDAFCLNQFDILSIPLRRHLPYRDIFEEHMLLQKEFGLTKYWIDQ
SYRDMIRANLTTFKDFSPLLENDYIEVHNLYWVFTMYFVGMGMGLCFFILEILRPLRYWRNCKIKCEYCYAFLKNFAK
>DmelIR52d
MVRIIIILLCLGYTKARILDATNTNHTDLEERLLSLLLRLQQEQFFNTLLIYGEDCAFSSLSRRLQVPTILVSSGSTSFEWNYSSLALI
LTCEFKAEREENYQTLKKLQMNRRLILLNGNIKPDSVCDFYSKKDQYNIAMVNNNFHQVGIIYACRLFQERNYEKVYLSEGNPIY
VDQFRNMQGALLKSITFNLIPGSMAYRDPKTGQEKHIGYVANLLNNFVEKVNATLDMQVKLHKAGKKTSFYNITKWASEDLVDI
GMSYAAYFEMTNFDTISYPYLMTSTCFMVPLPDMMPNSEIYMGIVDPPVLVVLIAIFCIFSVMLNYIKQRSWRSLSLVNVLLNDIC
LRGFLAQPFPFPRQSNRKLKLISMLVCFFSVITTTMYTSYLQSFMWGPPIDPKMCSFADLENSRYKLAIRRYDIEMLRPFNVSMDH
VVVFDESSQLEYLRDSFDDNYMYPMSALSWSAFKEQQKLFAFPLFYYSEKLCLKPISFFSFPIRRHLPYRDLFEEHMLQQNEFGL
STYWIDRSFSDMVRLKLATMNDFSPPRLEDYIEVSDLSWVFGMYFTGLGISCCCFGLELLGLPSWTRRLRLTNWLRVRN
>DmelIR54a
MWTVITGIVLWAPVLVAGSAVDFIFRAAAEHSLSVIMIRIDYCPYNWAKDIFENQTIPVVVLSDSETFINIRMFSRPLHVACLPGHE
LQKDLALLENFTSSLMDFPSQKKIVYISNNFSDPTRMDYIFETCYHRRIWNIVGLLASDEHRYFYRYHLYPSFRTEYRSLESSTIFD
KDFPNMHGHPLTVMPDQWLPRSVLYVDRRTGKQILAGSVGRFFHVLSWKLNATLQLSKKVTTGRFLNATALKELSESFSVDVPA
SLTIMERVEQLASTSYPMEVTHVCLMVPVARRIPIKDIYFILSSASNMFLAIVIVSSYGLALNLLRNMTHRDVRLVDFVLNDKALR
GILGQSFNLPLSRSFSTRLIFLMLGIVGLNVSSIFGAGLDTLMAHPPRQFQARSFAGLRRTKIPLVTTEEDFPTWMKLRVPMLVVNV
SEYNHLRNGRNTSNAYFASRLYWNLFSEQQKRFTRELFIYSTDDCLWSLALLSFQWPQNSLFTEPVSQLILEVNANGLYDFWVG
MHYYDMTAAGLSGLEDPSLQLKEREHPTSLRIVDFQWMWQAYGTFMVIAILVFLLEVSWHRITSLFVSLVY
>DmelIR56a
MGSRFFIRNLILFGLLASSNMQIPFGELEKKFELDVDFLLGVTELVGHIQGLYSITVYADCIDIHPSIQQRIMDKFMVPVNTIGSNLS
RPNYHKLDNSRIRIVLFTGLNDTILVNLNKTDVPYSDNFYMLAYASAIKNKCIELDFIEEVFTLLWKMSIQNAILLIRGEFMMEMW
SYLYMGKIHKIKLTKPNSYLESLRKYNYRFSLEVINDPPAIFWYNSSEQADVTGGGNLSVSGPLGLIIINFLRHLNVTIDIVPIPGKQ
TSQYELFQQPDNLRAENGVNMVGSALLKYSPMVTQSRMCLLVSNRRMIPFSRFLDRLVSPGVHKLTFVSSIGIFVIKYFSHRPRSF
VDAIFCTIRFFFAIPLPSIILNRLPVVDRFIEVFIIIFVQILLSSNISITTSALTTGFWEPPIINVETMRASGLHILTEDPTILQAFKENILPSS
LADLVILVDEDTYFHHVTTLNNSYVYVVQAHNWQIFRLYQQQMTNEPFEIASEELCSKWRILGIPLNPKSPLRFMFKDYFYRILES
GLREQWVHSGFKKFCEFNNLKKLPVDSVDSWQPLSIEFYSNVIRAYIIGLVIATLAFVAELLHNGYRRKNVKKT
>DmelIR56b
MLLDTDLASGVIRSPYSFDIPHAFIFNETQFVVPKFCGPYMEIVKHFAEVYHYQLFLDSLESLPKKSVVEQDIISGKYNLSLHGVII
RPEETSDFFNATQHSYPLELMTNCVMVPLAPELPKWMYMVWPLGKYIWTCLFLGTFYVALLLRYVHWREPGNATRSYTRNVL
HAMALLMFSANMNMSVKLKHASIRVIIFYTLLYIFGFILTNYHLSHMTAFDMKPVFLRPIDTWSDLIHSRLRIVIHDSLLEELRWLP
VEYQALLASPSRSYAYVVTQDAWLFFNRQQKVLIQPYFHLSKVCFGGLFNALPMASNASFADSLNKFILNVWQAGLWNYWEEL
AFRYAEQAGYAKVFLDTYPVEPLNLEFFTTAWIVLSAGIPISSLAFCLELFIHRRKQRRPQYERFECYDY
>DmelIR56c
MRSSFRICLFLLTTYHPSHGWNMQHLLNLLAPFGRMNVFQEIVWFVSPHQRLDQLDEFIMRIDEAFGKSATQTVVNNNTEMRMI

YSSARRNHMSFVFTTGAEDPIMKVFSKVLLGRHFYVSMVIYVDKVGDMHPIYDLLTFAYNQQFFNSMVHFESMEGVNQLFGVS
KFPVMSFENRTDFLKYMGKIWKQVQNARSDVGGFGFTTPLRQDLPHLFQSQGHYDGSTYRIIETFVRFINGSFKELIMPPDSLGG
QVINMKDALQLIRERKMEFCAHAYALFMSDEELEKSYPLLVVQWCLMVPLYNSVSTYFYPLQPFDWNVWFFALGALLALVLLE
LMWLRMFGGWSGYRGAVLNSFCYIINVPIEGQLQQPCLLRFLLLATVFFHGFFLSAYYTSNLGSILTVNLFHAQINTMNDIVSAQL
PVMIIDYEMEFLLNLNKELPQEFLELLRPVDSAVFSEHQTSFNSSFAYFVTEDHWEFLDEQQKHLKQRLFKLSSICFGSYHLAFPL
QMDSSLWRDIEYFTFRIHSSGLLNFYARSSFGSALHAGLVQRMPDTQEYTSAGLQHLAIAFILLLVMSFLAGIVFVLETLSR
>DmelIR56d
MDNRAAELILRERNIFPTNGSDNITLLNNMFVLEMFYRITQLYHFKNFIFYISERLDLNNKDSQEFFHNFWTYFPMAPNLIITREHH
LGIPMMQFISTPSLVMVFTTGKDDPIMELASHNQQGIHWLKTIFVLFPSLQSRDFETNPESLAQFTAEIKDVYDWVWRKQFINTFL
ITIKDNVFILDPYPTPSIVNKTGVWQAEEFFHKYAKNMKGYLVRTPILYDMPRVFKSDRPTNRYEKNFIHGTSGNLFLGFLEFVNA
TLMDTSANVTADYLNMTNLLDLVSQGVYETLIHSFTEITTKFVVSYSYPIGINDCCIMVPYRNQSPADQYMHEALQENVWVLISL
FTLYITVAIYLCSPLRPRDLSAAFLQSICTLTYSVPTFIIRTPTLRMRYLYILLAIWGIVTSNLYISRMTSYFTTAPPVRQINTVQDVVE
ANLRIKMLAIEYERMAKSPLQYPESYLNQVDLVDKHMLDLHRDPFNTSFGYTVSSDRWRFLNLQQLHLRKPIFRLTEICEGPFYH
VFPLHKDSHMRSVMTEYIMIAQQAGLMNHWERETFWEAVHLHRIHVHLFDDEPMALSLDFFSSLLRTWTLGLILAGLAFAAEM
KWHEHVTFKRRPVIRITRKPRSFLRRFMKL
>DmelIR56e
ERXAFRNQWAFCFPRTXAIEVVLSAWSPXCPGQRSKPQPISXPHHXGSCWRKRKWKXKPRLLVVDKRTLVEHLNSLNDGYAYCI
IAGHWQVGMM
>DmelIR60a
MWCNNPGLIIIIFLGQILNLCQGIVNLSNETANTVIFMLPEKDLGPDVWKAGVGCLDSFAQIFFFRNPKERFTRAYNLMLVHAFHL
SSPADQIQEGFSKLINEAVTNPGPPDREELFQMRVASDYNITNGTEDKGELILADNYVIVVDSVDRLKELMKKKIVEMRSWNPGA
RFLVLFHNATCRNRPLGVASNIFKDLMEMFYVHRVALLYANSTMNYNLLVNDYYSNVNCRILNVQSVGQCHDGKLYPNNAVVK
ASMQDYVSGFSPRNCTFFACSSISAPFVEADCILGLEMRILGFMKNRLKFDVNQTCSLESRGEMDGPANWTGLLGKVQNNECDF
VFGGYYPDNEVADHFWGSDTYLQDAHTWYIKMADRRPAWQALVGIFEAYTWIGFILILIISWLFWFTLVMILPEPKYYQQLSLTAI
NALAVTISIAVQERPICETTRLFFMALTLYGLNVVATYTSKMIATFQDPGYLHQLDELTEVVAAGIPFGGHEESRDWFENDDDMWI
FNGYNISPEFIPQSKNLEAVKWGQRCILSNRMYTMQSPLADVIYAFPNNVFSSPVQMIMKAGFPFLFEMNSIIRLMRDVGIFQKID
ADFRYNNTYLNRINKMRPQFPETAIVLTTEHLKGPFFILVVGSCWAALTFIGELIIHRWRTQLVSTSEQQDRRSDKRRRRRRRRKPE
KDNRWQRQVQVAPVVRFTPVKRRKVFQGQTSQK
>DmelIR60b
MRRSLYLIIAIGLVDVHCVSLRYILNALENELQYRAILLVESASEIESCWEQKYIQGAVPILNFNANQSLYLKDALNTNILALVCLN
ENVESTMQALYENLEDMRDTPTILFVLSDSKVQDVFLECLRRKMLNVLAFKGLDRGFVYSFRAFPTFRVIERNVMDILQYFEQQ
LEDLGGHTLTTLPDNIIPRTVVYKSPDGSRQLAGYLYPFLRNYVSTINATLKVCWHLVPEDGMIQLGEVVRLSEIHDVDFPLGMH
GIEHGSTSQNVPLEVSSWFLMLPMEPSLSRAQFFIMLGFEKVTPVLLLLTILLSTAHRIEMGLRPSWRCYVLGDRVLQGTLGQAFF
LPRRLSVKLMLVYSLILLNGFTFSNYSITSLETWLVHPPSGHPIHSWEQMRTLNLKVLIVPSELDSMTKALGKQFTESNSDLFELSK
SGNFQDKRLAMDQSYAYPVTCTLWPLLEHAQIRLPKPEFRRSREMVLIPLLIMAMPLPKNSMFHKSLNRYRALTHQSGLYEFWF
KRSFNELVALRKIHYKVNGDHQIYRDFEWQDFSYVWLGFVGGTIASILVLLAEIGYHRWQLNQN
>DmelIR60c
MEMRLALFFTFACLAGAHDGSLRNMLKSLEDELGYRTILLLEGFVYSFKAFPTLRVVKRRVKDVRRYFEPQLEDLGGCVLKVVP
DGIMPRTMVYQGEDGELQMGGYLSHFIRNYVSTINASLQIRWDLFPEDGDFDMDSLTGSNHVDFPLGLGSLSFQTLHKDVAMEI
SSWFLMLPMEPSLPRARFFIRFGISLYLIPLIILLAIVLSNAHRFEAGLTPSWRCCSMGNTVLRGVLAQAFVLPKGLSPKLMFVYWL
LLVSGFFVSNYVIVYLTAWLIQPPTSDPVTDFDQMRRAKLKILMVPTDMDYLKSIRGAEYVDAHSDVFQTADSTDFQTQRMSME
LHFAFSVTGTLWPLLRQAQVKLHRPIFRRSKEMVFLPFVIMGMTMPNNSIFLSSLKQYRLRTSEAGLYLLWFKKSFSELVAIHKISY
KEDWVHDSYSDLKWEDFLFAWLGFLGGTTVSCLALLAEIGYHRWLWKRTHQ
>DmelIR60d

MRLAIYVAFLSSIGNRSGFLSSLLMSLGKELHYKTILLVGGSSTCWSLEPFETGVPILNLRGENNAYPQDTFNSQMLALACLQTES
EDAVKLLYRSLKDMRDTPTLLFASSEEHIHDTLFLGCFRENMLNVLALTASSKEFIYSYQAFPTFRVIKRKLVEIHRYFEPQLKDLG
GHIVSALPGNIMPRTMCYRNAEGERQLAGYLNTFIRNYVESINGTLRISWGLVPEDDMRHLTISRLSKIQHVDFPLGIIPLYNKTDK
QHVYMEISSWFLMLPMETSVPRAHLFVKLGLERLLPIIVVVGAVLGNAHRIEVGLGPSWRCYYLADKVLRGALAQPIVLPRRLSP
KLMLIYSLLLLSGFFLSNYYMASLTTWLVHPPASDRILEWDQLRYLHLKVLTIPEEFKYMSLILGTDFMTAYGSIFQLTNSTDFQRR
RISMDPSYAYPVTTSLWPFLELSQVRLRRPLFRRSYDMVLQPFQVMSLPLPRNSIFHKSLLRYAALTRETGLYYYWFRRSYYELVA
LGKISYKEEEGNPYCDLKWNDFRIVWLAFLGGTIISCLALLLEVAHYRWHLGNSSL
>DmelIR60e
MVIKMISFLLVSVLLCLVGASDSESMQVQVLQDLNLALQTELNVFIDFECCATSEILHKLDSPRILLSSNSREARDLRIRGNFTEST
LIIVSVMDSDLNPLVASLLPRLLDELHELHIVFLSNEEPGFPKQDLYTYCFKEGFVNVILMSGKGLYSYLPYPSIQPISLSNVSEYFD
RARIIRNFQGFPVRILRSTLAPRDFEYSNEQGGLVRAGYLFTAVKELTYRYNATIESVPIPDLPEYDVYLAVAEMLHTKKIDIVCYF
KDFSLEVAYTAPLSIIREYFMAPHARPISSYLYYSKPFGWTLWAVVISTVLYGTVMLHLAARGARVEIGKCLLYSLSHILYNCHQKI
RVAGWRDVAIHGILTIGGFILTNVYLATLSSILTSGLYDEEYNTLEDLARAPYPSLHDEYYRSQMKAKTFLPERLRRNSLSLNATLL
KAYRDGLNQSYIYILYEDRLELILMQQYLLKTPRFNMIRQAVGFTLESYCVSNSLPYLAMTSEFMRRLQEHGISIKMKADTFRELI
HQGIYTLMRDDEPPAKAFDLDYYFFAFVLXTVGLISSLLVFFAELVSGHL
>DmelIR60f
MRFHLNIANSGLLGLHLCPTRSALPEQNPCFSKAGAVIXNLTLPWRRWRERCLLGALRPXTLPTPELQCXSKYLPXRKSQQENAS
SGLPGFCXGDXQTELHRGSRAIALPRSPYHDLYYVWIAYLGGTMIGIGMLAVEIACFKWDLLRRPPIXMY
>DmelIR62a
MYLQFLFALFLSRYQIVATENFDRAFELALFLDRIGRVHRLHAITIVNSLGSVDPSYLDDLHRGLMCNSSNHFYMLPQMTATDKD
SSHVHFSSLQDEETIYLVFARDSKDAVIYLQAERARGRRYTRTMFLLRKQESQKDIKYFFELLWKLQFRSALVVVAARNFYQMDP
YPTVRVIRMRRLSSYDPHHVFPPANRKNFRGYRMRLPVQQDVPNTFWYKNRRTKAWELAGLGGILINQLMMHLNVTMDLFRF
EVNGSSLLNMAALTDLIVKGKVELSPHLYDTLQSNTSVDYSYPTQVAPRCFMIPLDNEISRSLYVFLPFSLTMWLCLLFVLLVVHF
VYVRRLIPDGHFWAILGVPGAGQVRYGNRKPVRRFSTFLILFGIFILGQTYSTKLTSSLTVTLIRRPDNSLEELFLLPYRILVLPTDVY
AIVDSLGHAEQFSTKFSCTDAENFSQKRISMHPEYIYPISTIRWRFFDMQQRFLRKKRFYFSKICHGSFPYQYQLRVDSHLKDALH
RFLLHVQQAGLHDLWLDTCYRKAHRMGYLKDFSTLAELEEKLRLRPLALNLLVPAFSLFLCGMLGSGIAFLVEIRHSFGCRQKPP
SINRNPGD
>DmelIR67a
MLPILVPVLLLFNETSWINPILTSIYKDRHHETVLLLQHSQHGNASGLERFPWPVFSFNEQMDFYVRGKYNSEMLVLIWQTGNSD
WDLDLWQALDRSLLNMRKVRVLLLRKWEKIPTADVAATAEHLLFLHVAVIGQQGNRIYRLQPYAPQSWLQVDPIESPIFIKIRNYFG
RYIVTLPDQFPPRSIVYRNPKTDEIQMTGYVYKFLLEFIRIYNFTFRWQRPIVQGERMNLILLRNMTLNGTINLAISLCGFETPSXLG
VFSDVYDMEEWYIMVPRAQEISIADVYVVMVSGNFLIVLIIFYFIFTILDTCFGPLLLKERVDWSNLMLNERMISGIMGQSFNMSA
RNTISSKVTNATLFLLGLVLSTLYAAHLKTLLTKRPTSQQISNFKQLRDSPVTVFFEEAERFYLKHAWDRPIRYIKDQLNFRETIEY
NALRMGLNRSNAFSALTSEWMIVAKRQELFKQPIFTVQPELRVIQTSVLLSLVMQSNSIYEDHINDLIHRVQSAGIVEYWKHQTLR
EMITMGMISQKDPFPYVAFREFKVGDLFWIWLLWVSFLFMSFVIFLCELLVDCFISKTLIRNKRPH
>DmelIR67b
MELLYLNTLQSLSLLEGNRLVQTVQELNNIYQTELNVFLEFGNGADILESAQGTFVPTLWIKNPQNQKVMKGNFTSCTLTILYLE
DEHLDRGLYYLANWLWEYHHLEVLIFFNGGSYDKLIQIFSRCFNEGFVNVLVMLPGSDELYTFMPYQDLKILNLKSIKEFYSLSR
KKMDLNGYNITSGLVIAGAPRWFSFRDRQNRLILTGYMLRMIVDFTNHFNGSVRLMNVLTVNDGLELLANRTIDFFPFLIRPLKSF
SMSNILYLENCGLIVPTSRPLPNWVYLLRPYAFDTWIAWLIMLIYCSLALRILSKGQISISAAFLKVLRLVMYLSGSRDMGTRPTTR
RLFLFVILTTSGFILTNLYVAQLSSNSAAGLYEKQINTWEDLDKSDSIWPLIDVDIKTMEKLIPDRTKLLKKIVPTLEADVDTYRRNL
NTSCIHSGFFDRIDFALYQQKFLRFPIFRKFPHLLYQQPLQISAAFGRPYLQLFNWFVRKIFESGIYLKMKDDAYRHGIQSGLLNLA
FRDRHLEVKSNDVEYYYLIAGLWFGGLTLATVCFLLELLIGYAKIKVTISCKMNIM
>DmelIR67c

MFCWLIFLNIILLSDRSESWSAREVIHQFNHDQQLQLNIYLDCNDVELQIGQEVSNLFVNSTADKMKILGRFSSHSLIIACFKDSTR
NRTLNGVKELLWGLQYLPILFVVDSNMDFYFQQALRHGFIHVLALNFMNGSLYTYKPYPKVEVHQIKDMQKFYKLTKLRNLQG
QAVRTTVETMTPRCFRYRNRHGQLVYAGYMYRMVKEFISTYNGTEEHVFGNVDTVPYKEGLAALKNGEIDMMPRIIHALEWY
YFYRSHILYNIKTYIMVPWAEPLPKSLYFIQPFRGTVWITIMVSFVYASIVIWWIRYRQQGNSSLTQSFMDVLQLLFQLPLSKIWHF
NMGTHQVVSFIVLFVFGFMLTNLYTAQLSSYLTTGLFKSQINTFDDLFREKRTLLVESFDAEVLHNMTKEKIIQKEFESIILITSIEEV
FKHRKSLNTSYAYEAYEDRIAFELSQQRYLRVPIFKILKEVYDQRPVFVALRHGLPYVELFNNYLRRIFESGIWIKLQEDSFLEGIAS
GEISFRKSKSREIKIFDKDFYFFAYILLGMGWCVSTIALFLELWSFKYSVTNVLHEG
>DmelIR68a
MRCLWILIVAFISLAMATSIPIPIANPAPLSGYEMQLKILLQKILWVANVKRCFAVITDDLHYPIYDRIFFESVGRRVIPFFVMRTNES
DDLQRPSRQVELFVKAIKSSDCELNVITILNGWQVQRFLGYIYDNRSLNMQKKFVLLHDLRLFESDMIHLWSVFIDAIFLKRQLD
NKYTISTIAFPGILSGVLVMKNIANWELGKGLNGRILFADKTSNLFGTSLPVAISEHVPMVLWANATKSFQGVEVEIMNALGKAL
NFKPVYYKPNQTENMDWTELDGGASVAYGSGNPDGYAQNGTHIDSMLVDEVAAHSARFAIGDLHLFQVYLKLVELSAPHNFEC
LTFLTPESSTDNSWQTFILPFSAGMWVGVLLSLFVVGTVFYAISFLNAIINGNVSSEFFRCLRPNRNVPMDPKIYRRISFRIAISRYRS
SKGDRMPRDLFDGYTNCILLTYSMLLYVALPRMPRNWPLRVLTGWYWIYCILLVATYRASFTAILANPAARVTIDTLEDLLRSHIP
PSTGATENRQFFLEANDEVARKVGEKMEVFGYSDDLTSRIAKGQCAYYDNEFYLRYLRVADESGSALHIMKECVLYMPVVLAM
EKNSALKPRVDASIQHLAEGGLIAKWLKDAIEHLPAEALAQQEALMNIQKFWSSFVALLIGYVISMLTLLAERWHFKHIVMKHP
MYDVYNPSLYYNFKRIYPQH
>DmelIR68b
MKFLVGLLLQWYLPGIYALAEIACRIAVEQNVQVTYLYRCASCPASFDADYSALELDLYRCVGSRLPVITRNMEAHELEPFRRTD
SLSIFQIPAAEKGDSLVRRILDMLNPHQRRKHMHKYLFVWPNAGRHQLLRLFRGSWAKKLLYGLAITGRENGTFDFDPFAWGGL
QVIQRLDGEVPYARKVKDLRGYPLRFSMFTDPLMAMPRSPVETAGYQAVDGVAARVVGEMLNASVTYVFPEDNESYGRCLPNG
NYTGVVSDIVGGHTHFAPNSRFVLDCIWPAVEVLYPYTRRNLHLVVPASAIQPEYLIFVRVFRRTVWYLLLVTLLVVVLVFWVMQ
RLQRRIPRRGVIQFQATWYEILEMFGKTHVGEPAGRLSSFSSMRTFLMGWILFSYVLSTIYFAKLESGFVRPSYEEQVDRVDDLVH
LDVHIYAVTTMYDAVRSALTEHQYGLLENRSRQLPLGIATSYYQPVVRRRDRRAAFIMRDFHARDFLAITYDSQAERPAYHIARE
YLRSMICTYILPRGSPFLHRLESLYSGFLEHGFFEHWRQMDLITRVGASPDAEEFLEDLGDQTDTDSGSNELAIRNKKVVLTLDIL
QGAFYLWSVGIGISCLGFAVEHAHWFWRRQTLRNAVEARTS
>DmelIR85a
MSIQWLKHILLLAILVNLAGTRENHIPLDLKKSSIVMVKMSQILCKARIKVLFVYFENQTSHEHTGQILKEVTKCDISNQNTPLEA
VKDDGILMYMVMITTNISQPLELSLIRKKSAAKHRSHVFLLVRDADTVSDAWMRASFRQFWKIWLLNIVILYWRDGRLNAYRY
NPFMDNYLIPVDNKPNEVPTLEQLFPKTIPNMQRKPLRMCIYKDDVRAIFWRQGTILGTDGLLAAYVAERLNATMMITRPHSYN
NHNLSSDICFLEVAKEYVDVAMNIRFLVPDTFRKQAESTVSHTRDDLCVIVPKAKTAPTFWNIFRSFGSLVWALILVSVLVANVFC
YILKSEVGRVPMQLFAGALTMPMTQIPPNHSIRLFLIFWLYFGLLICSAFKGNLTSMMVFQPYLPDINQLGALARSHYHIIIRPRHV
KHIQHFLTLGHKHESRIREQMLEVSDTQMYEMMRNNDIRFAYLEKYHIARFQVNSRVHMHLGRPLFHLMNSCLVPFHAVYIVPY
GSPYLGFLDSLIRSSHEFGFERYWDRIMNSAFIKSGVKVVNRRRGSGNDEPVVLKLQHFHAVFALWLVGIGMACIVLAWEHLTH
NYNLAVTKRRD
>DmelIR87a
MSTPEQRFWLAALLFLLSQHSEVRGFGINLMKVQTEDKGQEACILALLRKYFDSGDGLSGSVLCINRNYQLPNIEEQLLRGVNN
YENYPWSLLITNSREGPSPAKFLMNEKPQCYFLIVDNLEDEDLDEVFEHWKGMVNWNPLAQFVVYLASLEETDEEMNDLMVEL
LLTFINKKIFNVNVIGQSEENQFYYGKTVFPYHPDNNCGNRVISVELLDACDYPSEETDSEDENDEDEGDGAQEEDDGPQEEGDG
EQEEEDGPQEQEDGDQAKGDEGQENDDGGLENKVENEFRIGASDDDELENDLSSNSSEPEAIIEEFFRAKFEDKFPRDLSGCPLT
ASFRPWEPYIFRNSEEQPVDDYYYGLQGDEDDYNDTSPNYGESDDESYADPGEDGDGAIPDTETQSGGKLKLSGIEYEMVQTIA
ERLHVSIEMQGENSNLYHLFQQLIDGEIEMIVGGIDEDPSISQFVSSSIPYHQDELTWCVARAKRRHGFFNFVATFNADAGFLIGIFV
VTCSLVVWLAQRVSGFQLRNLNGYFPTCLRVLGILLNQAIPAQDFPITLRQLFALSFLMGFFFSNTYQSFLISTLTTPRSSYQIHTLQ
EIYSNKMTVMGTSEHVRHLNKDGEIFKYIREKFQMCYNLVDCLNDAAQNEHIAVAVSRQHSFYNPRIQRDRLYCFDRRESLYVYL

VTMLLPKKYHLLHQINPVIQHIIESGHMQKWARDLDMRRMIHEEITRVREDPFKALTFDQFRGAIAFSGGLLLVASCVFAFELCYV
KYVYRTEKRERKTKKITKKVHNIKIQHD
>DmelIR94a
MALPKQLKFINIFLVLLIIYGSSDGTENQHEIFLNRLLQAVHNERSVETLFLLHHSNLANCSLQDWNPPRIPTIRSNELTVFNVEKTF
NHNALALVCLMKNSYREILNTLAKSFDCMRQERIILMIHRKSDSKFIEDITHEVKNLQFLHLIVLIVQEKYNGQVFASTLRLQSFPE
PHFKRIRNVFAIQRIFYRPINFHGKVLNAIPNDIPILFVALNEMFTEYARRYNSTLRIQNRTIKEDIEITEDNYDIDMKIQLHNSQNFL
HHMNIAMDIGSNSLIILVPCATELRGLDIFKELGVRTLTWLALLFYIIFVLVEMLFVFISNRFNGRNFTMRYTNPLINLRAVRAILGQ
TSPISNRYSLSIQHFFVFMSLFGTLFGGFFDCKLRSFLTKRPYYSQIENFSELRKSGVTVVVDHTTRQFIEQEINANFFRDEVPNVRT
TTIQELINHVYSYDRKFAFVANSIPWRTFREEMKSINQKILCDSKNLTILENVPLTFSIRRNAIFSHHLRNFIINAADSGMITCWFKM
AGKVIRKHIKTTLRESEQQPSHLPLSFDHFKWLWAVLCIAYVMSFMVFVMEILWSKYQRRTRSVSIV
>DmelIR94b
MSLIFNLLFILILSQAVSQETEFLQLKYLNNIVRSMIKLHKMETLVIVKHHLDNNCSLQNWNAHGMGIIRTNDQGKLIMKDTFNSR
TLAIICIGQNSHITLLRNVFETFGKVQQKKIILWTQMELKEKFFQEISKKSRDLKLLNLLVLKAVTKDKLLIYRLNPFPSPHFKRIENI
WTPNDTLFMDTKFNFHGMTAVVKHDYNWTIQMGNIRKFPISRIEDKEVIEFALKYNLTLQFFNDVERFDIELRKRIILKSNSTQPID
SGIPMVFSSLLIVVPCGNYLSIQDVIKVSGIEKWIFYIILVYVIFVLIEITFLGVTILISRQSRHQMIPNTLVNLCAFRAILGLPFPETRR
TSLSLRQLFLAIALFGMIFSIFINCKLSSMLTNPCPRPQVNNFEELKTSGLTVVMDHDAENFIEKEIGVDFFNQYMPRKVTLTFTER
AKLLFSLKGNHAFTLFSESFAIIESYQRSKGLRAHCTSEDLIVAERVPRIYILENNSILDRPLRRFIRQMQESGITNHWLKNIPSSLEK
NLMQITIPYDRERVHPLSIEHLTWLWCILILGYSISMIVFFVEMSLKRRKKNLENRAPNICIC
>DmelIR94c
MSKVFKLLVLPLIYLSLTKGSKNPQLKFLRELINVIEEGREIRTIMVIKHSRDEYCHLDQWNPRGSPILRTNEMGSIRISGYFNDQAV
ILACMGENSDYGLLKSLANAMDNMRQERIILWSEREPTKMLMDYISQQADRYNFAQIIIVTMNEDVDAVPSLHQLNPYPTPRFR
QITNISNIRRTSFFGCGLSFQGKTAILKESVVSNIRFKVWSPSGPIPLSELKDYEIVQFAVKYNLSLKLYDQNESKSDHFDIQLGPLFI
TKDFPTQMAFVSPNTACSLIVIVPCSPKWRFMDVLHKLGVLKLIGCLLIAYAVFVLIETLILWLTHRISGREVRLTSLNQLLNPRAF
RGILGLPFPEFRRSSISLRQLFLVISVFGLVYSNFVSCTLSALLTKPAQNPQVRNFKELRDSGLITIMDKYTHSFIEKHIDPEFFDHVL
PHYLILQKKEALRMIWNFNDSYSYVMYTTTWKSLNTVQKSFDERVFCESESLTIAWNLPRMYVLGNNSVLKWMLSRYITYMPQ
TGIPDSWTEQLPKVLKLLYNVTSPRRIKEGAVPLSIQHLSWIWHLLFIGESIATLVFIVEILLQKSNQHTSNMRERSSEDDDFV
>DmelIR94d
MGQLHLLLVALVLLSPGGDSFYHSLIHHLNRELKIEYVLLLGNFDTTWLDILWQLPVSVLQIKEHSRETYSLLENPSHNVLTIAFV
NDSPEDILEILYRNLRMLNTQPVLLVIRKSTIRVNSLLEWCWHHQLLKVVAIAQDFMESLIVYSYNPFPVLQFIERRLDNSTVIFEK
RLENLHGYEVPIALGGSSPRLIVYRDLEGKLIFSGPVGNFMKSFEQRYNCRLVQPYPFDESAISPARDLIASVQNGSVQIALGAIYP
QVPYTGYSYPIELMSWCLMMPVPEEVPHSQLYSMVFSPMAFGITIVAMVLISLTLSMALRLHGYRVSFSEYFLHDSCLRGVLSQS
FYEVLRAPALIKAMYLVICLLGLLITSWYNSYFSTFVTSAPRFPQLTSYESIRHSNIKIVIWKPEYEMLLFFSENMEKYSSIFQLQED
YKEFLHLRDSFDTRYGYMMPMEKWSLMKEQQRVFSSPLFSLQDDLCVFHTVPIVFPMVKNSIFKEPFDRLILDVTATGLLSRWR
DMSFTEMIKAGQLGLEDRGHPKEFRAMKVGDLIQIWRFVGWMLGLATIVFLLELICFWRHKMWQNMKYMFCRNKNI
>DmelIR94e
MDCPKWILSGLCLISLVSGATVIELLGTLKLELDFEYVLLMKNRNFSLSDQVWNGTSLTKDVMDEVQVPVLQFNENVSYFLHNSI
SRRLVTLGFMSDANLDEHRGLLTALVANLRHMTTSRVIFLVQSKASTDFLYELFRNCWRKKLLNVIVIFQDFETTSTFYSYSNFPIL
QIEERIYETSLQTLPIFPDRLRNLHGYEMPVILGGTAPRMIAYRNKKGNVVYDGTVGHFMTAFQQKYNVKFVQPLQAKNPLDFA
PSMQTVGAVRNETVEISISLTFPTIPPFGFSYPYEQMNWCVMLPVEADVPPFEYYTRVFELAAFLLTLGTLVLISCLLASALSLHGY
ATNISEFLLHDSCLRGVLGQSFVEVFRAPTLVRGIYLEICVLGILITAWYNSYFSSYVTSAPKQPPFRTYDDILASKLKVVAWKPEY
AELVGRLLEFRKYETMFLVEPDFNRYLALRDTLDTRYGYMITTNRWVLINEQQKVFSRPLFQKRDDFCFFNNIPFGFPLHENSVF
MEPVQKLIMELAETGLYYHWITTGFSELIDAGEMHFVDLSPHREFRAMQIQDLQYVWYGYAFMVVLSSLVWLLENLAYTVKSK
TIFPTHFMQRNKK
>DmelIR94f

MSGMWQQVLLAETSNWFRSDVLQRFWTHLRVEIRFRTMLNYRLESCDCWFDNVLGSDNSTALLWNDQTYPHYLRRRQDTDIL
VVSCLRFHQYQEVLLALSLMLDQMRSMPVVLQLCGDEDSMQELNSARLLLKHSQDLKMPNVVLLSSTFFTSATLYSYEMFPEF
NVQKLVYQAYLTLFPYKLGNLKGHPIRTVPDNSEPLTIVRKTLNGSIAIDGLVWQFMIEFAKHINATLQLPIEPHPEKSIKLVQILDL
VRNQTVDIAASLRPYSLNVQRSSTHIYGSPMMVGNWCMMLPTERVIGSHEALTRLMKSPWTWLILLLFYSVHRFLAQKTRLRSS
LIHLIKLLINLSLICFLQAQLSAYFIGPQKVNHISNMQQVEESGLKIRGMRGEFMEYPIDMRSRYASSFLLHDLFFDLAQYRNSLNT
SYGYTVTSVKWELYKEAQRHFRRPLFRYSEEICVQKLSLFSLIQQSNCIYCYRSRIFILRMHEAGLIRLWYRRSYYVMVTAGRFPI
GDLSTVHRAQPIRWTEWQNVVLLHGVGLLFSVVVFVIELTVHYANVCLNNL
>DmelIR94g
MSTAVNSVHSKLVSLISRGQELTSIFFYAPAKEKCHLEDTISSATWGLPLVIWRTDRTVILNGFIGEGLLVLACLPGFHWRALLGSL
ARSLKYLRQARILIELMQDRDEFLVSEVLQFCLSQDMINVNAIFDDFPETENLSSFEAYPSFEVVNQTFTPDTQVSDLYPNKMLNL
RGGVIRTMPDYSEPNTILYQDKEGNKEILGYLWDLLEAYAHKHNAQLQVVNKYADDRPLNFIELLDAAQSGIIDVGASIQPMSM
GSLSRMHEMSYPVNQASWCTMLPVERQLHVSELLTRVIPYPTLALLLLLWIFYEVLRGRWRRHSRLQSIGWLVLATLVSSNYVG
KLLNLFTDPPSLPPVNSLAALMESPVRIISIRSEYSAIEFTQRTKYSAAFHLALHASILIGLRNAFNTSYGYTITSEKWKIYEEQQKR
SSKPVFRYSKDLCFYEMIPFGLVIPENSPHRAPLHSYTLLLRQAGLHDFWVNRGFSYMVKAGKINFTAVGERYEAKTLTITDLRN
VFIIYVSVLLISLILFTCELFVSWVNYWLGF
>DmelIR94h
MLSNISFSSAPELVDLYGLVLKFLVSSETTLFYFNPTGQKCSWETLPRTILSNHPQIIWFREETYPGLYKRHSSNLFVMACLSSTSYD
GQLQLLAESLTRYRSVRVLIEVQDKEGSFLASQILLLCQQHSMLNVVLYFSRWTRTLNVFSYLAFPYFKLLKQRLSGSLRPKIFIN
QLKDLQGYKIRVQPDLSPPNSFSYRDRHGECQVGGFLWRIVENFSKSLKGDTQVLYPTWAKAKVSAAEYMIQFTRNGSSDIGVT
TTMITFKHEERYRDYSYPMYDISWCTMLPVEKPLSVEILFSHVLSPGSALLLILAFILFFLIVPQLIKCLGITFRGRLIGMASRIFALV
MLCSSSAQLLSLLMSPPLHTRIKSFDDLLTSGLKIFGIRSELYFLDGGFRAKYASAFHLTENPNELYDNRNYFNTSWAYTITSVKWN
VIEAQQRHFAHPVFRYSTDLCFSSETPWGLLIAPESFYREPLQHFTLKINQAGLITQWMTQSFHEMVRAGRMTIKDYSRTNLMKP
LRIQDLRKCWVIFAVGLGTSTVVFTIELLLIYTNVFLNSL
>DmelIR100a
MATTLQLIMLALVGGTLGQANNTDHKQVLTSIVKQLEGGLELHLRTSEDGGNDLVQFLMQEKSSIIISAKQEEVPSRAKIMRHHF
FIFDGVHQMQEIRTSLFNTDGFYILALENNTIEDDVLLMEFAADVWLQHGHSRIYYVQLSKKSVLLFNPFLQRLVVVQDSKTYSR
IYKDLEGYHLRIYIFDSVYSSVIGDGENKVLSVTGADAKLAKTVARQLNFTADFVWPDDEFFGGRLANGEYSGGVGRAHRGEV
DIIFAGFFIKDYLTTHIQFSAAVYMDELCLYVKKAQRIPQSILPLFAVHMDVWLCFLLVGLLGALVWLILRAVNLILGIEGVPDGSR
ATRISYFGAARRIFVDTWVIWVRVNVGRFPPFHSERIFVASLCLVSVIFGALLESSLATVYIRPLYYRDVNTLRELDESGQPIYIKHP
AFKDDLFYGHNSEVYRRLDAKMMLVAEGEERLIEMVSKRGGFAGVTRSASLQLSDIRYVMTKKVHKIPECPKNYHIAYVLPRPS
PYLEEVNRIVLRLVAGGIVGLWTGEAKERAKWSIQRFPEYLAELDVGRWKVLTLSDVQLAFYALTIGCLLSAIVCMAEILLGRQR
>TcasIR40a
MRRDHGGDLVSASFDIVAGFLFEEICICFDKNTNINFLQHLLVRFVSNNIAIKLFNITTVEVQDKYFAFLNYQVTNHLGANTIFFSS
HKFYEHVLLEINERDFIRRNLIYIFNWGRRPFSRYFVRNIINVMKVFVITNPRNDTFRIFYNQAVPYKKHHLEMVNWWQHGVGLF
NHPTLPAKYNNVFKDFKENVFKIPVIHKPPWHFVQYGNDSIKVTGGRDDRILSLLSKKLNFRYDYFDPPERIQGSSASENGTFKG
VLGLIWKRQAEFFIGDVALSHERANYVEFSFITLADSGAFITHAPSKLNEALALLRPFQWQVWPAIGVTFVVVGPVLYAIIALPNA
WRPRFRVRSHARLFFDCTWFTTTVLLKQTGKEPSSSHKARFFIIILSISSTYVINDMYSANLTSLLAKPGREKAINNLNQLEKAMAT
RGYDLYVERHSSSYSLFENGTGIYSRLWQMMNRRQTHFLLESVEEGVQLVRDSTNKAVIAGRETLFFDIQRFGASNFHLSEKLNT
AYSAIALQLGCPYIEEINKILMAIFEAGIITKMTENEYEQLGKKKQTTSETEKELIPGVKKENRRVAKVSEDNEKLQPISIKMLQGT
FYLLCIGNIFSGFILLAEILVYKHRKTYKHKKRRHRFVYLRKIRHSVASKFGAVVDAVRRVYRRAMHDAFVATLEYLE
>TcasIR21a
MQRGLIVLKLCLTALALKSLDKRALQKSHEKSQLEKWEDKFLNRDPSFDQTASLVNLISKVALDELSGCSATILYDKFTETSSDLL
LEKLFRTFPIPYLHGQITDKYHMKVPKLQTSQDTCTGYILFLKDVMRSKDVVGPQTNNKVVLVSRSSQWRVYEFLASEQSQSFM
NLLVIAKSEKIVSSSIARLICLALHLKFGTALAIYAPNGGKSAVYPSVIANVPKLGFRSAESVTSVITQNGANLGIGGLYITDTRLKA

TDMSHIHSQDCAAFISLASTALPRYRAIMGPFHWTVWLSLTLVYLFAIFPLAFSDKHTLRHLLDKPEEVENMFWYVFGTFTNAFS
FFGKDSWSKTDKFATRLLIGFYWIFTIIVTACYTGSIIAFVTLPVFPATVDTPEQLVRGKYTVGTLDKGGWQYWFENSTDPITQKLL
TRIDFVPDIESGLKNTTKAFFWPYAFLGSRAQLDYIVRTNFTTINKRSLLHISSECFVPFGVSIIYNKNALYSKIIDQGVLQAVQSGIV
DKIKNDVEWETMRSASGKLLAANSYGKSLKALTVDDRALTLDDTQGMFLLLGIGFLLGGASLLSEWMGGCLHLCKGNRNQSA
TSIQSNYRSHEVPTPREKLDSMQFNSFENHKIEEEIVEERNCIIHRQDDDDIEEHINRLFDFEGVFGEANPDSRTGPEEELSFKNTTK
AFFSLYAFLDSRAQLDYIVRTYFTSMNKRSLLHISSECFVPFGVSIIYNKNALYSKIIDQGVLQAVQSGIVDKIKNDVEWETMRSAS
GKLLAANSYGKSLKALTVDDRALTLDDTQGMFLLLGIGFLLGGASLLSEWMGGCLHLCKGKRNQSATSIQSNYRSHEVPTPREK
LDSMQFNSFENHKIEEEIVEERNCIIHRQDDDDIEEHINRLFDFEGVFGEANPDSRTGPEEELSEENGKK

>TcasIR76b

MGLFEIALAALCLNATCPGEEEPPEFPEVQYLAPDSNDRKTLFAQLTEQLKNENLIITTLKNDRLSGTEKRNNTILGKGIAFDLLNI
LQDKFQFNYTLIEPKANVWGAEKFGVLDLLKDKKANLSAAFLPVLTQYSNHISYSPSLDTGEWVVLMKRPKESATGSGLLAPFN
LPVWLLILLSLVVVGPVIYFIIYLQAKLCKDDNNKVFPLPACIWFVYGALLKQGTTLNPMTDSSRLLFATWWIFITILTAFYTANLT
AFLTLSKFTLPITEPKDIGEKRYKWVTTKGNALEDTVTVNESLTELGKILGQPQRYLYVSDSDILRNYVHKRNWMFIREKPIVEYV
MYDDYKEKTRNQIEEAKRCTYVITKFSVVSFSRAFAYSKDFKYKPLFDSTLVQIVKCHKCFSLLSRIQYLVESGIIKFKLREELPDT
EICPHNLGNKERQLRNSDLLMTYEIVGGGFIISAIVFIIEVIIRRQKKPKTKSLPLQNPNKHTFEINLNNNYEKFGHFPYSSKFVTPPP
PYHTLFNPPHKSDNMKKRNFNGREYWVYDSISGETKMIPMRTPSALLFQYTN

>TcasIR93a

MLLELVLSSAFVCVIRGDSFPSLLTTNATLAVIIDREFLSNEYEVIKHAIESYLVFAKREILKHGGVNVQYYSWTTINIKKDVTAIFSI
ASCPDTWRLFRQARDANLLHMAISESDCPRLPPDEAITVPLITRGEELPQLLLDLRTRQTYNWNSAFILYDDTLSRDQVTRVVKSI
TAQYSNLRVNAAAISFVKLETRLPMDEIRRQVKEILSSVSIKTVGGNFLAIIGYELVELLMEYAKMFGLVNTRTQWLYIISNTHFRH
KDINRFRQLLSEGDNIAFLYNNTVNNDTCTGGIQCHCEEILSGFTRALDEAILFEWETSSQVSDEEWEAIRPSKLDRRNSLLQGIKT
FLLQRGQCDNCTSWLMKTGDTWGREYQQNGTDSGGLISVGNWRPSDGPSMSDELFPHIVHGFRKRNLPIVTFHNPPWQIIRSNE
SGAVSEYAGVIFELIKELSKNLNFTYTVELAKIGQEFSANLTKNEAQVVTNFIPDSILDMIRNKSVAFGACAFTVTEESKRLINFTSP
ISTQTYTFLVSRPRELSRALLFMSPFTGDTWLCLSASIVSMGPILYYIHKYSPVYEYKGLSKRGLSSVQNCIWYMYGALLQQGGM
HLPQADSARIIVGAWWLVVLVLATTYCGNLVAFLTFPKIDIPITTIDELLAHSGTVTWSMPKGSYLERTLKYTTEPRFRYLFDKKVE
VGNFKNMIEDIENGKHVHIDWKIKLQYIMKQQYLDSDRCDLALGLDEFLNEQLAMVVSQDTPYLEIINDEIKKLHQVGLIQKWL
TDYLPKKDRCWKNNRHIVEVNNHTVNMDDMQGSFFVLFLGFLLSFFITIGEKLWHKYVTKKKMKIIQPFTT

>TcasIR64a.1

NKISLILVILSKTETYIIKSCLSNAIVDFAILANVAFSLRISCYKLFMHIKLIANVFYNQLDQVLNRNHYHLAVIIDSGCIDYADFAIQD
KKYFYETYHWLVPTTPQNLNNSLNFLQKSPLNINSDVNVAILNGEGTKWSILDVYNPASSHHGQFTVTKLGLCDETNGYQAKIA
GNKYWSRKNMTGVQFKSAVVVPDPSIKLNDYLTSDKNRQLHSMHRFQSVTVNYCREMYNFSLEIQRTNSWGYLTPNGHFDGLV
GLLERRLVDFGSSPLIYKLDRMPVIDYSYGNWVLRSTFIYRRPKIIEASYKIFLRPLSRTVWICIVLMMVLLMLFLKVVFSREKRLL
QKRNLVDSSWSFLFLFTLGAFCQQGATCHPQLLSSRTLSIFVFLFCILTYQFYSASIVSYLLIDPPRKINNLKDLSDSNLRAGIEDILI
DRNYFVQTTDPVAIELFNKKIKFSNNNSGFYEPWDGLDLVKQGGFAFHVETSTAYPIIEETFTNEEICELEEVQMYRTQPMHTNLQ
KNSPFREMMNYCMLHLVENGLMYRLRKYWDARKPMCIESAKKFTFNVGLKEFSSGLIVLSYGILISLGLLLREVIVHKK

>TcasIR64a.2

MSPPLPFMILLSVLTQTHALLDINLIENYFTEKSIKSATVFGCFRKTEQLNLVKIFSRGSSPISVLNLNQAGVYQSIKSNHQQIGVVL
DGDCPESESFLITVSPGFTHIAPNVVFISVRSTETXFDVKHHWLILSKSIQFLEKIKNAVVNINADIHVAVQSGTNWTIFDVYNPASE
HGGSLKYTRVGFYSRGRGYNAQTNEAKYWRRKDMTGVTFKTMVVLLVPFEGPLEDYLHNDDNRNINTFNRFQNKLLRFCRDY
YNYSMIVELGSSWGYPFPNGSFDGMVGAMEKKLIDFGSSPIFVREDRARVIDYGRNTWSWKAGFLFRSPKSRTSIEIFLKPLSTSI
WLITGVLATASIVILKMVTTFERNRYHSTSETSWSLSFLFTLGALCQQGSPWVPKMACGRITAISIFLLSLIIYQFYSASIVSHLLMK
PTNKIRNLKDLTDSSLKVGCEDIIYNKDLFAHTTDKVLKDLYAKKIYGKGNTSHFFPPEKGLDLVRQGGYAFHIEVARAYPIIETTF
PDNAICELREVKLFKNTDLYNTMQKGTPFRDMLESCFQRLAEQGILDREKKHWHPRKPECIQSSQAFVTFHVGLDEFYPALLVLL
IGIVISLTVLVVEKQIHIAREKMEREKGVVF

>TcasIR64a.3
FQLRVLMERLFFLSVLAVIIYTTNCTDNHDIITSYIKEKSVKYATVFGCFTKKEKINLVKIISHICPISVFDINRLNIENRMESRHFHTGIILDGDCPSAEKFLINCGRSYLFDVKHHWLIVASSEKIREKFNNVILNINADINVIIPEKPSNWSIIDVYNPASQHGGVLNFTRVGFYNKHDGYKIKYTGVKYWNRKNLTGVTFKSMVVVTYSKTXKNSAYTIFQLPVPFEGTLQHYLDSDDNRDVNTFNRFHSRLISFCRDYYNFSLDIEVSKSWGYTNEDGTFDGMVGALERKIIDFGSSPLFLREDRARVIDYGRNTWILRSLIKQQFRIISNWGFSAAFIFRNPKVRTSLEIFLRPLPSSVWLITGLLAIVSIIILKLATSFERRRYVYDVETSWSISVIFTLGAFCQQGSPSTPKMACGRIATFFIFLLSVLIYQFYSASLVSHLLNKPLTKIKNVRDLLLSPLKAGCEDILYDRDYFLHTTDKVAKELYAKKILGKSNSSNFHTPEAGLKLVAEGGYAFHVETATAYPIIESTFQDQAVCELREVPLFRTQPMHANFQKKSPFRDMFDTCFQRLAEHGLLVRERKHWHPRKPECIQSSKSIRFNVGLDDFYPALVILLVGIVASLLILVIEKEFRILTENPA
>TcasIR75q.1
SFLGTILTVYKQLAEKKIVLNVLTNHWKINQTKLSQHTFLVGDTLCPQFNSLLSHVSKFFCYQNSQQTLGQIITSSXKWLVFDQNSTVNTNDLLLDSNFAVASQISNGRFHLKLCYKRAPNETIKFNEIGVFSNGFEYYNHFIPTRNRSDLSGVNITVSYVVTKPDYPFDVEDYRFRHLEAFSKLSYAMVYPMLEMLNCTKKFIQRSSWGYKGANETQFVGGMFGDIQNGTAEIGGTVSFYTVDRMSVVDYLSVTTPSDLKFILRAPPLSYVNNLFTLPFDTKVWYCLYFIVGVTVLILYVIVRCESTYENALERRNNIDNIKPKFFDVVMLQIEAITQQGSENEPKTMSGRIAVFIVFLVLMFLYTSYSANIVVLLQSTSANINTLQDLLNSKITLGVEDVVYSHHYFETQTEFTRKSIYEKKVAPKNQKSNFMTTEMGIEKMKDEFFAFHVETTAGYKQIMDTFQEHEKCGLIEIDYLNVLYPSITIRKNSPYKEIVKVNFRKIYESGIRHRQLNRIYYKKPHCVGKGGSFKSVGIVDIYFSVEIFAIGCFMALWLLLLEVLFKKKIKFLVQ
>TcasIR75q.2
MKILIVFICLLINETTQNNFTDNLIVNTFNFIKILNVPVKISAHICWTRGKFDSLLMKLYXTVLANTIHFIKSISDKYNTNLIKNVSPKYANPEHQLFIIDLKCNDSLSVLQQAEKFKLFKSPFKWLLLGNSESLPNLYFGTDSQIFVTEPRSQLDDIKTIYKYSPMVPRFVQHSFDRFYTNTKRTNLMGTTIKISYVITNLDSLNHLWDYRLQELKKKLYHFLICRNSHIDAINKLNYILVHNLMDFLNASRQFTMQPTWGYKNSTTGLYSGMAGDLQKGLADLGGTPLFFTPDRIDIIDYIAATTPTYMKFIFRAPPLSYVTNVFTLPFDSAVWHYCFVMVAVVVVCIYVIVVWEWKETKFEEKDTHSHIDTLRPNIFDVVMFEIGAITQQGTNAEPKSNSGRIITIFSFLTLMFLYTSYSANIVALLQSTSDSIKNLEDLLNSRIKLGVEDIVYAHYYFENAQEPVRKAIYQQKVAPKGQKPNFMTAEEGIRKVQQGFFAFHVELSTGYKIIGEVFQEGEKCGLKEIEYVNLIEPWLATQKKSPYKEVMKIGMRKMHETGVQNREIRKIYTRKPQCHSGGSNFGSVGLIDCYSAFLTFGVGIAFAFLLFVMELIVRRYFIRREKERLK
>TcasIR75s
IVLPMINDLIEHFNKTQIILAYLCDKNGTNLLLIRNNNNTNFRRLSGSEPLFXKKLYQVNVLSPNSRDMPYPTPPAFLTYVLDAGCSNTKQLLLLVPVITHXLIFGNNILKASEQKQFATPFKWIVYYNNPVELSFFIDEYFTKTNILVDSDVTLATINPTSGTFDLNKIYKRKINGSIIIENIGIWGRGLGVTDTGYEKITYKRRRNLTKTVLKSCIVITNNDSLNHLTDKRDIHIDSIAKVNYVLVQHLSDTINASLEYSVRGTWGYKDNKSQWSGMIGELTRNEADIGGTALFLTSDRIRVIDYIAMTTPTRSKFIFRQPKLSYVANVFTLPFDASVWASVCGLLVIIAGLLYVVVRWEWKKKDYVQVVVFFAFWVDFPSSVFCRTNRTSRKFTILGSXVFITFGALCQQGSSSVPFSIPGRITLIFLLVSLMFLYTSYSANIVALLQSSSSSIQTLQDILNSRLDVGVDNTVFNHFYFPNATEPIRRAIYQQKVAPPGQKPKFYPIEEGIRKMRQGLFAFHVETGPGYKFVSEIFREDEKCGLQEIQYLQVPDPWLAIQKNSSYKKMLKVGLRLLQENGIQEREVGLIYTKKPQCLARGSSFISVGLVDCYPAAVVLAGGIGAALAVLILEIYVHQRFVGFLL
>Tcas|XP_971730.2
MLRAILLVLILFMSVHSYQNFQDHKDSLNVGLILPYTNFGVREYTRAINNAVSGLHRSRGQRLNWLKKYNFTPKNVHYVLITLTPSPTAILKSLCKEFLSVNVSAILYLMNYEKYGRSTASAQYFLQLAGYLGIPVIAWNADNSGLERRASQSSLQLQLAPSLEHQTAAMLSILERYKWHQFSVVTSPIAGHDDFIQAVRERVSAMQDRFKFTILNAVLVSHHRDLAALVDSEARVMLLYCTSQEAIDILTAAKDFHLTGENYVWVVVTQSVIANPLEAPGQFPVGMLGVHFDTSSSSLVNEITTAIKVYAYGVEDFTNDLANAGRSLNTQLSCEGEGAARWNTGDRFFRVLRNVSVEGEAGKPNLEFTQDGVLKAAELKIMNLRPGVSKQLVWEEIGVWKSWQKEGLDIKDIVWPGNSHTPPQGVPEKFHLKITFLEEPPYISLAPPDPVTGKCSMDRGVLCRIASDADITEVDTTLAHRNGSFYQCCSGFCIDLLQKFSEELGFTYELVRVEDGRWGTNENGKWNGLIADLVNRKTDMVLTSLMINAEREAVVDFSVPFMETGIAIVVAKRTGIISPTAFLEPFDTASWMLVGVVAIQAATFTIFLFEWLSPSGFNMRLSLNQSNDTSHRFSLFRTYWLVWAVLFQAAVHVDSPRGFTARFMTNVWAMFAVVFLAIYT

ANLAAFMITREEFFEFSGLDDHRLSRPYSQKPLIKFGTIPWSHTDSTIAKYFKEMHAYMRQFNKSTVHEGVDAVLSAEMDAFIYD
GTVLDYLTSQDEDCRLLTVGSWYAMTGYGLAFPRNSKYLKMFNKRLLDFRENGDLERLRRYWMTGVCKPGKQEHKSSDPLAL
EQFLSAFLLLMAGILLAALLLFLEHLYFKYVRKHLAKTDRGGCCALISLSMGKSLTFRGAVYEAQDILRHHRCRDPICDTHLWKV
KRELDISQMRCKQLEKELEAHGIKPPPPCKR
>Tcas|XP_969654.1
MFVFVIFALNWLTIRADLWSSNNPTVFNIGGVLSSNESEYYFKETIAHLNFDSQYVPKGVTYYDTAILMDPNPIKTALNVCKYLIT
SRVYAVVVSHPLTGDLSPAAVSYTSGFYHIPVIGISSRDSAFSDKNIHVSFLRTVPPYSHQADVWVEMLKHFNYKKVIFIHSSDTDG
RALLGRFQTTSQSLEDDVEIKVQVESIIEFEPGLETFKEQLSDMKNAQSRVYLMYASKTDAQVIFRDAAEFNMTDAGYAWIVTEQ
ALVANNIPEGILGLRLVNATNEKAHIKDSIYVLASALRDLNQTKEITEAPKDCDDSGQIWETGRDLFDFIKKQVLMNGETGKVAF
DDQGDRINAEYNIVNIQRKRKQVTVGKFFFNRTSNKMRLAVDENNILWPGRQHVKPEGFMIPTHLKVLTIEEKPFVYVRKLVEPQ
DVCTAEEIPCPHFNATQDLAGSYCCKGYCMDLLKELSKKINFTYSLALSPDGQFGNYIIRNSSGSGKKEWTGLIGELVGERADMIV
APLTINPERAEFIEFSKPFKYQGITILEKKPSRSSTLVSFLQPFSNTLWILVMVSVHVVALVLYLLDRFSPFGRFKLANTDGTEEDAL
NLSSAIWFAWGVLLNSGIGEGTPRSFSARVLGMVWAGFAMIIVASYTANLAAFLVLERPKTKLTGINDARLRNTMENLTCATVKG
SAVDMYFRRQVELSNMYRTMEANNYNTAEDAIEDVKVGKLMAFIWDSSRLEFEAAQDCELVTAGELFGRSGYGIGLQKGSPWA
DDITLAILDFHESGFMESLDNKWILQGNVQQCEQFEKTPNTLGLKNMAGVFILVAAGIVGGIGLIVIEMAYKKHQIKKQKRMELA
RHAADKWRGCVEKRKTLRASATTQRRIKSNGVNDPATISLAVDKYQRIGGPERAWPGDSDIRQRRVEDSGGVQPVPRYLPAYTS
DVSHLIV
>Tcas|XP_968786.2
MSVTNVKNIVLLTFFTISVSATGDKIPLGAIFEQGTDEVQTAFKFAMLNHNQNVTARRFELQAYVDVINTADAFKLSRLICNQFQR
GVYSMLGAVSPDSFDTLHSYSNTFQMPFVTPWFPEKVLAPSSGFLDYAISMRPEYHQAIIDTVRYYGWPKIIYLYDSNDGLLRLQ
QIYQGLVPGSESFQVSTVRRISNVTEALQFLRGLEEQSRWEHKYVVLDCSADMAKEIVVSHVRDIALGKRTYHYLLSGLVMDDR
WESEVIEYGAINITGFRIVDSSRKHVKDFLDNWKKLDSTGSQNTGRESISAQAALMYDAVFVLVEAFNKLLRKKQDIFRNNMRR
GQIFNNGSKGLDCNASGGWVIPWEHGDKISRYLRKVEIEGLTGEIRFSEDGRRQNYTLHVVEMTINSAMVKVAEWSDETGFTPV
AAKYIRLKSNAQIERNRTYIVTTIVEEPYIMLRSPEPGETLSGNDRFEGYCKDLADLIAKHLGITYELRVVKDGNYGSENHEVKGN
WDGMVGELVRNEADIAIAPMTITSERERVIDFSKPFMSLGISIMIKKPMKQKPGVFSFLNPLSKEIWVCVIFSYIGVSIVLFTVSRFS
PYEWRLLHLTGEHRDPSGQHSTHNSMANDFTMLNSLWFSLGAFMQQGCDIAPRSISGRIVGAVWWFFTLILISSYTANLAAFLTV
ERMVAPINSPEDLASQTEVEYGTLYHGATWDFFKRSQITLYSKMWEYMNSRKHVFVKSYDEGIRRVRTSKGKYALLIESPKNDYI
NEREPCDTMKVGRNLDAKGFGVATPLGSPLRDAINLAVLNLKENGELTKLMNRWWYDRTECIHDKQDAARNELSLSNVAGIFYI
LIGGLMIALAVALIEFCYKSHTEAVRAKIPLSDAMKAKARLTIGVGRDIDNGRYYTPANQIAGANEQEQAHSNTHTQV
>Tcas|XP_966884.2
WGSWGATLGLAGLLAVAVALPPVVKIGAIFTEDQRDSATELAFKYAVYKINKDKTLLPYTSLVYDIQYVPRDDSFHASKKACNQV
QHGVHAVFGPSDPLLGAHIHSICDALDIPHLEARLDLDTDIREFSINLHPAQHLLNTAFQDVMAFLNWTKVAIIYEEDYGLIKLREL
VRSPHNGDLEIHLRQADPESYRAVLKEIKSKEIHNIVIDTKPSNMQHFLKGILQLQMNDYKYHYLFTTFDMETFDLEDFKYNFVN
MTAFRVVDVTDLSVQEVLRDMARFQANINADSKLNSTYLQAEAALIYDSVFVFAIGLQTLEQSHTLKLSNVSCDKEQPWLEGLS
LINYINAVEFKGLSGPIEFKEGRRIQFKLDLLKLKQHALVKVGEWRPGAGVNITDRAAFFDPGTMNVTLVVTTILEQPYVMLRTQ
TNVVGNERYEGFCIDLLKEIASMVGFEYRIELVPDSKYGVIDLETGEWNGIVRQLMDKKADLAVGSMTINYARESVIDFTKPFMN
LGISILFKVPTDKESAFFSFFSPLGFDIWIFVGGAFFMSSFTLFTLARFTPYEWVYPQPWKRSKYLVNQLSMSNSFWFIAGTLLRQP
SGVNPQVPTSQQARLFSFMNPLAMDIWMYVFSAYVLVSITMFVVARFSPYEWHNPHPCDMENELVENQFSLANSFWFTIGTLM
QQGSDLNPKATSTRIVGGIWWFFTLIIISSYTANLAAFLTVERMITPIENAEDLAGQTEIPYGTLESGSTMTFFRDSMIETYKKMWR
FMENRKPSVFVPTYEEGIQRVLEGNYAFLMESTMLDYTVQRDCNLTQIGGLLDSKGYGIATPMGSPWRDKISLAILELQEKGEIQ
MLYDKWWKNTGETCSRNEKGKESKANSLGVDNIGGVFVVLLCGLAFAVIIAICEFCYNSKKNALTEKRSASAPHQSLCSEMGGE
LCFALRCRGSRQRPALRRQCSKCLPGATYVPAMLDIPPHPPQPPSRPPPTNGLSAHVCPEDTSLRDRMMIPLELQHHMQPQHPLH
QQLDN
>Tcas|XP_966711.2

MSHGAANPPPAISAPIFAEQITLKTTCAIFTEDQKDSSVELAFKYAVYKINKDRVLLSNTTLVYDIQYVPRDDSFRTSKKVCRQMEFGVQAIFGPSDPILGAHIQSICEALDVPHLEARIDFEPLSKDLSINLHPSQEHMNKAFKDLMTFLNWTKVAIIYEEDYGLFKLQELVKAPAAARTEMYIRQAGPTSYRQVLKEVRQKEIYKLIVDTNPRNIQKFFRAILQLQMNDYRYHYMFTTFDLETFDLEDFKYNSVNITAFRIVDVDDPQVKESLEVMEKFQPIGHAILNKSGIIQAEPALMFDSVYVFAKGLAAMGSIKPMNLSCDVEKPWDDGSSLYNYLGDDDLRGLTGNIEFNGGKRSNFKLDLLKLKKEEIRKVGQWTPSGGVNITDPNAFYESHAPNITLVVMTREERPYVMVKDEKNLTGNARYEGFCIDLLKWIAGQVGFQYTIRLVPDHMYGVYDPDTKEWNGIVRELMEKRADLAVASMTINYARESVIDFTKPFMNLGIGILFKQSSKSEPSRLFSFLNPLAMNIWLYMAGAYVLVSITIWIVARFSPLEWKEPELHEHADGRTLEILENGFTIGNSFWFAIGSLMQQGSDLNPKATSTRIVGGIWWFFTLIIISSYTANLAAFLTVERMITPIESAQDLADQTDIAYGTLEGGSTMTFFRDSKIGIYQKMWRFMESRKPSVFVKTYEEGVQRVLEGNYAFLMESTMLDYAVQRDCNLTQIGGLLDSKGYGIATPKGSPWRDKISLAILELQEKGVIQILYDKWWKNTGDVCNRDDKSKESKANALGVENIGGVFVVLLCGLALAILVAILEFCWNSKKNAQTDRQSLCSEMAEELRFAVRCHGSRQRPALRRSCTRCSPATTYVPAALDLPHINGRRLYAGGTSTFACTSSAIRDCCGGPSNSRPQRAATLNYRATPQCPDLAKKSATLGRNSGFCRDTCDIVQSGLQNDVPDKILWKFDCDIGSPNNPDVVTRTLPDAIVSRTTTL

>TcasGluRK1

IWLLIFVAYNYLFCLADDKNKITVGAFFEREDVQSKAALNYAIDTTNMMQQHLKYALKTQILAQNDSFYCCKLWSGLAAIFASKPIFESLSNRLEIPFILTKWRPASYSNKQTTVNFFPDSYLFSHGLAIIVKNLQWKNFVLLYDSDKGLVKLQQILKLNNFNSGSVIVRQLGPGPDHRPLLKEIRALNHNRIILDCDTENIIEILKQAKEVNLMESSYFNYFLTSVDAHTLDFSVLNTTANITTIRILDFTQXNQITRLGYKLXRFFGITLIVIXTETALIQDGIHSFITSVNTLHVTEPIVPSPMACDQKWSHGFRISSFMRVVIVKFWVCGPIGFDSSGRRLNFTIFVVEGNRENVVAKWRPENPEILIYMRGENDSFDALVKNMQKSVLIISSRLGPPYLMERKPRFEGEILTGNSRYEGFSMDLIDAIAGILGFKYEFRLAKDGKYGNYDPETKSWNGLIKDLLDRKADLAICDLTITHQRREVVDFSMPFMRLGISILYKKAEEKDVNIFAFLEPFSPEIWIYTATLYLVVSVILYLVARMAPGDWENPHACNPKPEKLENIWNLKNCLWLTLGSIMTQGCDILPKGISSRLATSMWWFFSLIMTSSYTANLAAFLTMERLEPTIDSAEALAKQTKIKYGTVEGGATQAFFRESNYSTYQKMWTTMIQAKPGVFEKNNADGVKRVQTTKNRLYAFLMESSQIEYEIETKCDLKQVGNWLDNKEYGIAMPIDYPYRSAINTAILKLQEEAKLTELKDKWWKKMRDEPSCPVRTLGKSSTELALDNVGGVFLVLGVGMAVAFVLAILEFLWNVRNISVEEHMTYFEALKVELIFALNVWVTKKRTKPKINSLNCVLXRVIKNQTRKSCLLIKNIVFINNMNKYDKK

>Tcas|XP_968606.2

MKIVYQNVIWWVFFAFYNYLSLAQDEPLTVDVVGFFDEKNGLSEIAFQTAISNLNIMKNSIRFNPLSTVVNTSDSFENSKFLCETAEAGKVGGVFCATSAKIAPIIESVSDNLNIPAVQVAWRPSATYTDMLVNVYPLPKLLFQGLGAIVRNLQWRSVVVFYESAENLIPLQDVLKTQDYNGGNKYNSLMLKELGPGPDYRSALKQIQNKSEYRIILDCKTENIVTILRQAKELKLLEPHFSYFLTSLDAHTVDFKLLNTTANITTVRIFDPASDNFQYAISNWNNYVKKMNIPGVNLDPYSVKTETALMHDAVHMFLKCITDLHATGKSVKPTKLSCENVDKWTPGFDIASFIKAYTHDTDGLYSTTAPISFDNLGRRTNFSIFVVEGNRDDVVAKWNPSDPEVLQFLKSEEDRNKELERKWSEGIVTTRIGPPYLMVKEQKSETDLLEGNNRYEGFSMDLIALLAKDLNIKFRFEVLKSGQRGAYDKTTKSWNGLIREILDRRAELAICDLTITPDRREVVDFSTPFMRLGISILYRKAEAKEADMYAFLDPFSLKLWMYSATLYLALTVVLFFISRISPQDWENPHPCEQEPEELENIWDMKNCLWLTLGSIMNQGCDILPKGMAPRLAASMWWFFTIIVTNSYMANLAAFLTNERSQSEINSAEDLAKQTKIKYGTLDGGSTQGFFRESNYSLYQRMWTAMEQAKPSVFEQSNDAGVARVQNEKNRLYAFLMESSTLEYQIQTKCDLKQVGNWLDSKGYGIAMPLDYPHRSRINEALLRLQEQGEINRLKDKWWKEERKDPLCPKESEDQDANKLALQNVGGVFIVLGVGVALAYIVAVLEFLWNVRSVSVDEHISYMQALKVELLFALDVRKTKKRAKPEVPESSSSSRSPSMARSFLQSAGSFLRLDKMNQMETPGSSRHTSRPLE

>Tcas|XP_974911.2

MEWLTQLLLVLPLMKFSNTLPDVIRIGGLFHPADDKQEIAFRYAVEKINSDRMILPRSKLSAQIEKMSPQDSFHASKKVCHLLRSGVAAIFGPQSAHTASHVQSICDTMEIPHLETRWDYRLRRESCLVNLYPHPTTLSKAYVDLVKAWGWKSFTIIYENNEGLVRLQELLKAHGPYEFPITVRQLGESSDYRPLLKQIKNSAESHIVLDCSTERIYDVLKQAQQIGMMSDYHSYLITSLDLHGVDLEEFKYGGTNITAFRLVDPDGPEVRKVVREWNLSEAKNKKGEISSIIRAETALMYDAVHLFAKALHDLDTSQQIDIKPLSCDAVDTWPHGYSLINYMKIVEMRGLTGVIKFDHQGFRSDFVLDIIELNKEGLKKIGTWNSTEGVNFTRTYGEAYTQIVEIIQNKTFVVTTILSSPYVMRKEASEKLTGNAQFEGYAVDLIHEISRVLGFNYTIRLAPDGRYGSLNRETKEWDGMIRELLDQKADLAIADLTITYDREQAVDFTMPFMNLGISILYRKPIKQPPNLFSFLSPLSLDVWIYMATAYLGVSVLLFILARFTPYEWQNPHPCNPNPDHLENQFTLFNCMWFAIGSLMQQ

GCDFLPKFSPYEWDNPHPCNSDPDVLENQFTLLNSLWFTIGSLMQQGSDIAPKAVSTRMVAGMWWFFTLIMISSYTANLAAFLTVERMDSPIESADDLAKQTKIKYGALRGGSTAAFFRDSNFSTYQRMWSFMESQRPSVFTASNVEGVERVVKGKGSYAFLMESTSIEYVIERNCELTQVGGMLDSKGYGIAMPPNSPFRTAISGTILKLQEEGKLHILKTRWWKEKRGGGACRDDTTKTSSTANELGLANVGGVFVVLMGGMGVACVIAVCEFVWKSRKVAVEERSSLCAEMANELKFAMRCQGSTKPIRKKGRPCDASTGVDDARFHPLGSYTSYGFVVNKEPIN

>Tcas|XP_974933.1

MATFSILFLILLEACASPENPKALKVAFFLNENAALDELAITSATNYINNYAATQYALNFVLAPRIYRIKKSEIYNVGNLACDALREGIAAIFGPENGEANEIIQSMALSLEIPQFQTFWNPNFATYAGLGTANKKEIFNFNLYPSPSVLSKAFATLVRENDWRSYTIIYENDDGLVRLQEVLKALSPNNPLVTYRKLGPEPDHRPVFKEIVASGALHIILDCEADHTIDILSQAKEVKLFEEYHTYLLTSLDAYTIDFRQLGEIKTNVSIVRMLDQKVVDTVIGNWELVDSERKLKIPNKLIKVKTAFLFDALNLFITAYSNLDQEQEMDVRPQSCDTNEISSHGYRLSAFIPLLNMTKGMLGEPISGSLNFNSLGQRVSLKLEVLELRKDEFRVTGIWDSGTPHSIYSTITSADREKELEQQLKGRTFRVVSRIYPPYLSRKPGIDSSVMSGNNAFEGYAMDLMKGICELYECNYVFELVPDNNYGKYDPKTKEWNGLIRHLLDRKADLAICDLTTTYERRKAVDFSNPFMTLGISILYTKIVKEPPDLLAFTNPLSLHVWLYMVTAYMVISMIIFLVARLNPNEWENPHPCNPNPEELENIWNIKNCFWLTLGSIMQQGCDILPKGISTRMVAGMWWFFTLIMISCYTANLAAFLTQSRMGPTIQSAEDLAAQTKIKYGCLKDGATASFFRDTNVTTYHKMWVAMETADPSVFETSNDDGVKRVISKKGKYAFLMESSSIEYEVEKHCELVQVGNRLDTKGYGIAMPTNAPYRTSINQAILKMQEMGRLQRLKEKWWKEKNKANTCKKDEDSKTDSANELSLAHVGGVFVVLVVGMSIAMVIAVCEFLWHVRKIAVTQHVALKEVFLKELRFAMDIWCRQKPANPAANLNLSRETLRQGD

>Tcas|XP_974901.2

MPKPRHETEQKAFHLATDLINAKYKDSSIRLIPDSHLIDNYNAYTTYLTTCELLQKGVIAIFGPSSIHSSPAIQTILDRKEIPHVETYFDRKLSRHDCLLNLHPHPSVMSQAYLEIVNKWGWRSLVVIYDSEESLAKLGLFAASCKQRVTLSRLELDMYDTFRTSLTSIKKTGETNFILECSVDILEAVLKQAQQVGMMTERHSYIITKLDLQTIDLAPFQYSEANITGFRIFNPENAEIMSLADQIYTQEKYKGIPSGWLLRHQTALLIDSVDLLHQAVLDLTLSEQVVIQSQTLYCNTSNNWDSGHTIVNYMKGQTIKGLTGVVHFDNEGFRRDFTLDILELSLGGLLRIGAWSFFSGLSLNRPPNLSKVKIVDDANLVNKTFTVITCLTTPYGMLKETTQQLFGNDRFEGFGIDLMDELSKMLGFNYTIIIQEDGYNGNYNQTTGEWNGLIGAILSGKADLAIADLTVTAEREAVVDFTLQFMNLGISILYKKPKPVPPSLFMFVSPFSYTVWILLVVTYFLVSMCFFVMGRLSPSEWTNPFPCVEEPEYLINQFSIRNSLWFTIGSLMQQGTELAPIGISTRTGAGVWWFFTLIMVSSYTANLAAFLTVETLVTPFSNVKELSEQTEIKYGAKRGGATANFFKNAGNDSVRSRIWHFMATHDEEMTESNDEGVERTEEKHYAFFMESTTIEYVIERHCSLASVGAPLDDKGYAIAMKKNSSYRNDLSAAILRLQETGKIAQLKEKWWKEKRGASNCGAQKSESAATPLNLQNVGGVFLVLFLGTGLGFCISFVELALRVYSTTKKTDQQFRKELIEEIKFFIRFKKNVKSVKPETH

>Tcas|XP_966620.1

MRCLGLTVFLLIFPNFLGQEQDRKEIFLGGIFTEPPDVDDSVLSDEEAFNFAIDIANREYSDVKFTSVSEESDLRTNGPFDSRLQACSLVNQQALVIFGPKNAEEIDIVQSICDNKDLAHVITRWVYSSADFRSVINFYPHSAYLTSAYFSVLKLWNWKTLTVFYEDNESMLRLGDLLNLAKNEGIIVTVKQLYEGLDETPIYRTTLKEAVRSGQKNFIIDCKIESLEEVLKQAQQVGLMTKDYNFFITNLDLQTINLEPFQYSEANITGIRILDPLNEMFHVKAGAIMRQKPNFNLTKMRTETALLIDAVSVITQVISRKLSIKEMEMTEISCNSPKSSRHGYTIANHVKTSKFDEMTGRIEFDGNGVRSNFDLDVIELTQNGISKIGTWNMSKGLVITPHKDEDIVEDPLSLRNKTFKVITCLTDPYCMLKENSGQLFGNDRFEGFAIDLIHELAQMEGFNYTFIIREDKSNGDKNKVTGEWSGMIGDVMHGVADLAITDLTITAEREEAVDFTSPFMNLGISILAKKPGNAPPSFFSFADPFALDTWIMLALAYIAVSVSFFVLGRICPDEWTNPYPCVEEPEFLINQFSLSNSFWYAVGSLMQQGTELAPIGVPTRMVAGMWWFFVLIMVSSYTASLAAFLANENTITLFTDVESLVQNYEEKGIRMGAKRKGATEGFFRGKDSETYKIIAKYMEEHPDDMVGDNKDGVKLANKETYAFFMESISIEYETQRHCDLQQYGGLLDDKGYGIAMRKNSTYRKTLSTAILKLQSSGQLDNLKRTWWEEKRGGGQCLDSGDDATPALDVRNVEGVFYVTIGGTLCAIVLIFFELFLSLLKISKKYKISMREALNNEKKAFLDFNSNVKPAPKAKSKSGSKSSGESGKSNNNTGAPTYGFIPTITKDTLDE

>Tcas|XP_966528.1

MLLLVITISLYFHKFSQAETLKIGAIFDTDDPIKERAFHHAIHQIEPIHGRTIEGLVKNVPPNDPFEAMLAACHLIESGAVAILGPTTHENAHMVQTVCDNKDIPLLDVRSVAHPQNSINFYPLQQILTQIYIKLLEAWNFENFVILYENDDSLIRLAELLKFYGNGHRMVVRQLDKYQNGNYRPTLKEVWRSGATHFVLDCSTDILEEVLHQAQQVGLVTNKQFYIITNLDFHTLDLTSFQYSETNITGMRFIDPDSD

EIQNLGLTLYRNDFTNTEFGFIEAWKVNLEMALIIDAVTMFGEVLNRLPKDFAIPSIDCASDKAWTYGTTLTNLVKSVKYPGYTGL
IQFDNFGLRSAFGLEIIELKEGGIIKIGNWNYSDGLNINRVYPPDPPPLVEGSLVNRTFIVITCLTEPYGMRRDSEVPLYGNERYEGF
GIDLIAELSKKLGFNYTFIIREDKKNGEFDESSGEWTGMIGDVISGKADLAITDLTITSERESAVDFSTTFMSLGISILYQKPKKALPS
FFSFADPFSLTVWKLLAAAFFGASIALFILGRISPSEWQNPYPCVEDEFLVNQLSLRNCVWFMVGSLMQQGSEIAPIAFSTRMVAG
MWWFFTLIMVSSYTANLAAFLTTESPDLPFKDVFELVQVAEKKGIKFGAKINGSTEKFFLDSKHVDEYQQIYKYMKNHEDEVMV
NDNKDGVHKAEHEDYAFFMETTSIEYETQRRCGLTSVGHSLDEKGYGIAMRKNSSYRMALSTAILKLQEEGVLAKLKRKWWEE
QRGGGLCPQGEKSTEGTPLNLKNVEGVFCVTIIGTVLSCVLVFVEMAVHTFKKSLRVKKPFKVLLMDEMRFYFRTSAMLKPVTA
PKPEPYGFITS
>TcasIR8a
MVISENLDKTTANRLKAIRPIPNNFAIVATSSNMEELLQTALDENLVTLPERWNLVFLDFQYQQFDKKRLKNMPINLLHMDEEICC
RFLQSEKCECPHDFNLQENFLSLATNTLAKILKTLTMENLLRADLNCDDSRYSEATRTRFYELLQQEVDSNDLVFKENFGLHVNI
NGVIETGDEKVAEYNYKTGVTVLDGKKVEPITPFFRIGITHALPWSYKETDSSGNTYWTGYCVDFTEELSKLMGFGYEFVEPKS
GTFGKKRDGVWDGVVGDLATGETDLAITALIMTADREEVIDYVAPYFEQTGITIVMRKPVRKTSLFKFMTVLKLEVWLSIVGALI
VTGFMVWFLDKYSPYSARNNKKAYPYPTREFTLKESFWFALTSFTPQGGGEAPKALSGRTLVAAYWLFVVLMLATFTANLAAFL
TVERMQTPVQSLEQLAKQSRINYTVVKDSDTHKYFINMKHAEDTLYRMWKELTLNASTDDTQYRVWDYPIREQYGHILLAIND
SNPVANASEGFRIVNEHTDADFAFIHDSSEIKYEISKNCNLTEVGEVFAERPYAVAVQQGSHLQDEISKTILNLQKDRFFEQLQAKY
WNHSGKGSCPTTDDNEGITLESLGGVFIATLFGLALAMITLVGEVLYYRRKSKIQNSETKKPKTVQTSENWKTDTLMPVSLINKD
KQSVTIGTEFKPVNRNRDLSEFGHITLYPRARNRITQTSNE
>TcasIR25a
MASSSAIIYRIAIYSRIATAHLNYSDFLNNVLTETHKMLKLVAFILYCTNLANGQTTQNINVLFVNEEGNLVAEKAVDVATNYIKKN
NKLGVNADPVKVVGNRTDASGLLDSLCSSYNEMIANSMNPHLVLDTTMTGLASETVKSFTAALGLPTISASFGQEGDLRQWRNI
DENEKEYLVQISPPADVIPEIIRSLVLSKNVTNAAILFDDSFVMDHKYKSLLQNVATRHVIAPIKEADKIGDQLRQLRKLDIVNFFIL
GSFENIKRVLDAADSVGFFNRKFSWHAITQDKGELKCNCRNATITLAKPLIDAQYQDRLGLIKTSYQLNAEPEIAAAFYFDLALYS
FLAVKEMIADGVWKRNNATNYITCDDFDGKNTPRRAGLNLKKYFSKEVSETPTYGPISIVSNGYSFMEFTMQISAVGVRESSSDK
SVPLGSWKAGYDNNLTLVDPQIMKNYTADVVYRVVTVEQKPFIIKDETAPKGYKGYCIDLIQRISEILNFDYEITPVGDQKFGNM
DENGKWNGVVRELMEKRADIGLGSMSVMAERENVIDFTVPYYDLVGITILMKLPKTPTSLFKFLTVLENEVWLCILAAYFFTSFL
MWVFDRWSPYSYQNNREKYKDDEEKREFNLKECLWFCMTSLTPQGGGEAPKNLSGRLVAATWWLFGFIIIASYTANLAAFLTVS
RLDTPIESLDDLSKQYKIQYAPLNGSSTMTYFERMANIEAKFYEIWKDMSLNDSLSEVERAKLAVWDYPVSDKYTKMWQAMKE
AGLPNTLDEAVKRVKDSRSSSEGFAYLGDATDIRYLEITSCDLQMVGEEFSRKPYAIAVQQGSPLKDQFNTAILQLLNRRELERLK
EKWWSKNPEAKKCDKQEDQSDGISIQNIGGVFIVIFVGIGLACITLAFEYWWYKYRKGGKVVDVQAKHSDVATKINDGFHAKIN
KLYPRSRF
>TcasIR144
MQVSKILLLSSLLLNRDETSKCLDAIFKQPVVVLRGVPKNLQNFDAWKPETYLILAPNATVLEQMLEKWSTIESFNPRAKFWLLT
HWHEIKPKTLTILAKFYIVNVAIVTRTGQVFTYYPYKYENIAQPDTKPVLLGQCDNVPSFPDKLPKFWRNTTVQVLTKCLLPYVD
CSDLDQGLETQIFDLVQEFLKFKVRRIFDKSFKFGLAKINGSYSASFRFLQEREVDMAMGSFRSVGSTQFRDFEFSTNHMEDKLV
WVVPKARPMVHWVRLVKIFEPSFWGLLVVLTVAMARVFEKMARFTDEPMGIYRKSGFRVAVLILIGSYLKKTPKRFEMRIIFIFWI
YFCMVLNIVFNSNLTNVFFGTFNTFQVNSFDDIIKSNLEMGLTDDVMHILSQEQNWPEITSTKVISSCAFGPACLNRTIFQRNLVCC
WGERSIKFRMAKFYTTQVHYVDDHLLFFYLLFYFVKGYPIVPQISKMIVQLKSAGFVQFIKSKVDKLEPRQGNELTTKILTLKRLE
GPFYFLLVGWVGGIMIFGYEVVTYERKRRKKVRQEVTKILKKKKMRQNEKVKILEI
>TcasIR41a.1
TKMLFNNFCINILVNFIINNYHKNSRCLLIFTDGDFDYKGEIPTVRIKATNGSFNSYLIFNYHGCQSVIIYTSNVTALLIKFETEIRLK
MERFNERKFLIVPQNPSEDFDKFFNLKQLYFISDLLLVLPTHNDTIFDLKTHKYVGVIDNNEPVLLDRWFSQNQSFLFGKNLYPNK
LQNQLGRPLKMATFTYEPYSIIGNVFEQFFENDFILQGKSVGEHHGSELMSAVQFALKYNMTPVPVINEKDYWGDIFPNWSGNG
LLGNLVDDKADVGFSALYTWEFCYHFLELSKPLVRTGITCLVPAPKLSERWLTPLFSYSSYLWFCIILTLVIAIFVLSLVLFCYNHNK

TLNLNYPLKRKTTYIHFLESAVTIVLKPVFQQSLTLRELPIEIASKLLMGLVLLLALFLTSSYGSGLATVMTIPTYENAINTVEDFAN
SGLDWGATQDAWIMSIQNAEEQRYVKIVSKFHPISEEELFQFSKSGKFGFSIERLPFEDYAIGDYIKEDVIDNFHLMKEDLYWEQC
VIMLRKNSVLLPALDLFILKIFEAGLISHWQNEAVDLYMNPKVQRAVKFYRQGQEHTVVKLQWSHVKGPFALLLIGLCISFIIFILE
LTLKKKRNQF

>TcasIR41a.2
TLGCLTMTNLNVLLQILLKTYFLNTRCIFLFTDSTIDLQVETPIVYFKVSNTLNPSLIFQHHGCQNILIHHENASDIFVQFENLIRLN
NERFNERKYIVTGHNSLKILLTKQLEYVSDLLLVVPKQTGHYELITHVYRHQNRSKINEPVLLDVWYSQNHSFRQENDLFPNKLT
NQNQRVLKIGTLSYEPYSVIGKLTVNXSPYYLNLGKDDYSFDGTETSLVYEFVHKYNLTPSFTIMGDDLWGDVYANWTGIGLFG
SVLNDEIDIGYAAVYTWEEYYKFMDYTKTLIRSGVTCLVPAPQLAAGWVTPLRSFSLGMWIALVIVLLSNTIVLNLLFYRNQKYH
XNQLFQILLFNAFSKRFFIDSLTTAIKLYVQQPLTLTLKRGLLKYFIVTNMIMVLFISSSYSSGLSSVMTVPRYGKSIQTVKDLASSH
LNWTGTTDAWIFSLRQVEEANYENIKNRFVVKTQNDLVTASKQYNFGFSVERLPYGHYAVGPYIQRDVICNYRIMQEDLYWGQ
CTFLLRKNSVLLPLLDKLILRVFEAGLEAYWENQVKCFGRKNMNLRDFLGCLPIHGHVCPKRHYVLYTTYXEHDTIKLTWEHVE
GAFAVLVLGYAASIFTFVIELILDKVRS

>TcasIR68a
MIKNLLPYKCVVLISDDIYGGTFTKSWYRRFGPFITFVVIRVDEYEDLLSPFEETQACLDTAKNEGCQMYLILLSNALQVSRLLRF
GDKYRVINTRAKFVLLYDNRLFDKPLFYLWKRIINVIFIRRYSGQKSDTKKNMPWYEITTVPFPTQITSILIPRRLDIWTKSKFRKGI
DLFRDKTSDLRNQTLKVAAFSHIPGTTKSLQEKTARTVIGNFSGTEVEILQTVSAAMNFHCELYEPVNVDVDLWGGKQSSGKYT
GLVGEMVSTNADIALGDLYYTPYILDLMDLSIPYNTECLTFLTPESLTDNSWKTLILPFKYFRPAMWAAVLVCLLICGAVFHALAR
FHETISQNKSQVLEIHTKRKKIIILSICPEIEKLDSNLKYTKMREQYKPPRFEGQSIGLYQFSEPFNSVLYTYSMLLLVSLPKLPTGWS
LRMLTGWYWLYCLLLVVAYRASMTAILARPTPRVTIDTLQELVNSRLKCGGWGEINRQFFKSSLDPITKLIGENFELVNDSNEAVD
RVAQGVFAFYENSYYLKEALVKRQLRFQIARTTQNQSEREMRDIAREDRNLHIMTDCVIKMPISIGLQKNSPIKPRVDKYIRRVLE
AGLIKKWLQDVMASILNAEVQSTQEEMKAIMNMKKFFGAIVALFIGYFISVVVLIVENVYFHFFVKRNPHYNKYTRSIHHVKKA
E

>TcasIR100l
MPRKLFLWIFFLLVSCYGNLSETHLQFLKRYFVSANSVAISMLQTHHQEVKIRDLAEVISRKLNSIGTPVVVHENHKSGSLNIIMIV
WSLKILRQFLDSLVVPEEKGTYYIIILEQDCATVHSDFAQILEQFWCEHNVLNVVVQNPCSGGTFYLFLPFEHRDNFWGSCKSWD
FNEQMPNKLRNLNQFPLKISLFLYNPTLIAKLPKGLKTNPRYHNLSASKGYGGLDGFLLRELVDYFNFDPVIVENLEEYGRVLPN
GTAFGSLGDVVNQRVHFSINSRFLMDYGTKEIEYTFPYISDEICMLVPKSLKVPTWKTLLKCFNTLSWVLIFVSCLCSTFAWYFVG
PSKNLHKLIWQIYCFIVGIPQKIEPSFSQFVFLLSCFFFNVTIFGIIQGSYFTEFATTSFYPDIDTLEELYESNLPVATHFWFLLDGDTSD
LMTKLKTHKIEATGDCLEQTARQRNIATLGRKSESDLIIRTKYTSRDGTPLVHIVEECHTSLYLCGIVPKGSHFLAPFNQIITRLFEG
GFTTKWYRDVFDGIISEEKPQLDETVSFNSLNMNDLQTAFHILTIGHLFSIMVLIGEVVIKGKHNKKLLT

>TcasIR100k
VTIIILIMMCLSLPKIQTCPIKINHLKEHFKQVKSARIMILQNEIIVTDWLIMELIKDNKITVTVQKAIRNFEPFNTSNLTRFEALEFN
DTIPTLQTDSTCGHLIIVKNEERLYQYLKSDPGFLILNPRHFYAIVAMELFKTNVLREFWSLQVSNILLLDCDTSYTVLPFNGTTIRI
NAYTQRKLLRNFHNYFLQVSMQPKPPTAIVKFPKPLRENPIYKDLVPFKDYAGLDGCLLKVLTQRLNMKYVIVGNGQKYGTVLK
NGTTTGTLAWIASNKVQISTNGRFLMTYGTNKLEFTVPYSSDQVCAVVPKALKIPKIIMLAKSLTPSSWFMIFLIYVICVLIYTLMG
STGSTWTLYAIFHGFPVKIVPTSRQSFFLTSCMLFSIIIMTIIEGSFFKTFTTTTYYKDINTLEELDESELPIAETFFSFTNDKSRIMTSL
KRKKLVINRDDILEQVARKRNIAKLERKRDIKVRLKTEFLDEEGESRLHVVEECFTTFYIGFIVPKNSIFLPTFNNVIRRIFESGLTQ
KWYGDVEFSIFLEKIFKLENNIKHHSFSFDNIVSALCVLFIGLSLALLVFFWEVTKXKQITLIYVSLIYCIISRH

>TcasIR100j
LTLVQVVICLLEVSHYDNEKFVNVYQHFTLVRYLTLTFLNDGVHRIDLNNLVVDLMSRLNFSMMIKEKRLGKNSTTFQESDPFQG
HIMVVYDVKVLLAFLEESTEVVPKARGSFAILFTSLKCPTHYETNHALKQLWTNHGTANLIAFCDNIYVYHPFSKNDSTWGATL
DYSPATETPNLFRNFNGYLLRVSLFKRPPTALKQVPSYISNNPIYRDLKPGDFAGLDGTLLRFLSNYLNFTVVIDESHPTHGRVLKN
GTITGSLSDVVSHRVDFSANDWFLIDYQTPEIEPTVPFSYDQVCPVVSKALKVPQWKAFFFIFDLTSWVLIFFMWLCCVFVWHVL

NPFRDLSTIIWEICSVLFGNPVNVVPLSNQHMFLGSCMVLNIIIMGIIQGSVFTDFTTTTFHKDINTLEELDEAGLKIASSAWYLDFD
TTDLIKRLKTKQIRNYIGSYKDTAFKRGMAVLGRKQDVEHMVKVEFVAEDGSPLLHVTSECLQTFLLVSLFPKGSPFLPTFNNVIT
RLFEAGLTVKWYQDVTSTGTMLQQMKNFANRRPTGLFSLNDAKLAFYALFVGYIASFVTFLTEILTKNHHNNVHNHVDVLKAQ
HHGQVQVDQ
>TcasIR100n
DTFWIVYQTHFLLTDYLTLHILETEDHKFELRQFTQNILKRVNKYGYFLSVRITKSSLNKRNKSYHFPSTAYAPSQNLAKLSDDQE
FYKAKRLSTDSKHGFALIVWDLTTLHLFLDQDYRTIVPEGRGTYAIQVVSKQCDVKNEIAFTLQRLWTEYQVINVVAQTPCSCDK
THIFIYHPFVKREGFWGLATSHTLDQIKGDSRLISNTLSDFNGFPLRISIFPRTPTAMQTLPKLLHYNPIYRNLTWSKGFAGLDGLVL
ATLAEYFNFEVVLVGSLLEDDFGKVLPNGTTVGSLADITERRAVYNANERLVAYFNLDQIDFTVPYTREDICLVVPKAAKIPKWKI
LFQSLDPQSWCFTLFAYVSCFMFWYNIGPSRSLPKVSWQMFSFFLGIPTKSFARKLDQVLFLIPCMIFSVVMLGVVQGSFFTKLTL
FSFYQDVNTLEEMADLELPIGAFIWNLIRDDSDVIRRLKSKSVKPPDNIFDMIAAHRNIATIETRARAQLLIGSKYVDDDGFPLLHI
VNECLTTFLNANIVPKGSALLTVFNAVLGKLFESGLTRKWNNDVVDSLIAEKMISVNRKRVRTKSFSLYDAQGAFFVILVGYACS
VFVFLCEIVLKXDKICYLALIINKT
>TcasIR100e
DDFWRVTKNHFLLVNSLTIQVLQTEEHQYDLNQYTVTLLKRLNSLNLLVALRMQEKFLSGRNFPKHSVTNHTFSTTKPKFDPIGG
EELTQLKRLSSDSSKGYFIVIWDVESLHNFLDEDFQVVVPEARAXYMIHFAFTYSTEACKIVKLQVSSVLTRLWIDNNVFNIIAQT
SCLCDLEVYVHRPFVKRGGFWGLTNSYQMSEIVENPRIIANPLINFNQFPLKIGIFPRPPTVIETLPKLLTDSPIYKNLSFSKGFAGV
DGLVLGTLAECLNFDTTVITSKPNSYGYIYKNGTATGAIADVIDRRMVFSANSRFLLIYNTDQLEFTVPYTAEKMCLAVPKALKV
YKWSSMFRCFNKLTWVSIICSFGICTIFWYLLKWQKLVTALATIAQFLLGVPANVRPNVPQMLFLNSCMGFNIVIMGIIQGFLFQSF
TTTSFYPDINTIEEMVDSELPLRSSIFYFLRIDNSSLIHKLKSRTMAAPPNVYDLVAFHRNIATTDIKSHVDFMVRSRYLDEDGWPLI
HTVDECFETFLIANIVPKGSAFLTVFNNVITKLLEGGLTQKWYEDVINSLILENWINLNRNKSKTHAFSLYDLQVAFYVIIMGCAVA
ILVFVAEIVHKRRNXNNCCNNHHKNIIFAA
>TcasIR100f
DDFWVIFSTHFLLATSLTFITVQTNSKQYDLRLLAQAIIQSMDKDQVMTTRHVILHNYAENINFNVVFKTGTKKNARDFVTDLLA
KTKKLASDSREGFVIITWNVNVLQKFLAQHISEINPRTRATYLFILISSSDSLRKIKHCLHFLWHKYDILNIVVHVLGCGTTTTLIYR
PFCKTKNSWGEITAHQIEEIVQQPLLLTNSLQDLNQYPLQVSLFARDPTALTQLPKLLQNNPIYKNLASFYGLDGSMLSTMAKILN
FEVVIVENHDRLPFGRVWPNGTASGTLGDVVNRRVALSSNSRILADYNTQEIEFTVAYNGDSICVAVPKSLKVPKWRVLFECFDA
ASWLLTLSVFIVCLCFWYCVALKNFARILWDVYSFLMGIPTRIVPSRQYFFLSSCMVFNVIILQLLQGWLFTAFTKTVFYPDLDTL
EVLEKTNLPVATNMWFLFKDNSEVIQKLSSRGIGKTPNSLDLVAYSRNICVLDKRQDLELYSQAKFVGPDGLSLLHIVNQCLTSVL
LVNIVPKGSPFLPVFNDIMSRLFESGFTKKWYSDVVTSRVTEKMVSLGRKERNFSFKIKDLQAAFYVMMAGCVFSLFVFVGELV
THXVFVMNKSSQSKSHRFLLNCNYGV
>TcasIR100g
TLFKIAEVTFFMVTMHEEFLSLLFGNYYHTNLYQTVKIQEKFARTNNKTGAWYENVALDQKLDPPIDQNWQRVKLRTSDSFEGFI
IIVWDPQTLDQFLNQNFSLVVPRARATYFLLFVFSIYENCKLVNHILKRFWSEFSVLNIIAQTPYCCNKVYIHRPFVKTTNSWGVT
QSYTLTEVTQNLALITNPLLDLNQFPLRIALFEKNPTAIRKLPKALQNNPIYRNLSRSKGFAGSDGFLLSAMVEYLNFDPLIDETLE
PMNFGHVLPNGTVCGVLAEVVHKRTDYAGNCRLMTYFGTDGYEFTAPYSSEKIAMVVPKAGKVPRWRSLFNCFNALSWSLIFSI
AIVSTVFWCFLRRSQHLKRASWEMFAHFVGIPCRVVPSRGQFMFLTACMMFNIIILGIIQGSFFTDFTTTSYYPDLNTLEQVLDSNL
PIMAFAWRLLRTNSSPILQKLEQRSIPYEDNVYELVALYRNVAALDRRLDLELEIKTKYSGRDGVSPLHIVDESLVTFLTTSLVPKG
SPFLVVFNHVIRSMFEAGLTAKWYDDVVTSLIIEHKHKTPSFGVKYRPFTLQDVQAAFYVIAFGYSCSVFVFWCEIIVKFSGKIKH
FHYYFVLI
>BlonNmdar2.1
MMISLLFTLGLLESVTCTFYTHSKSLNQQNDTLNIGLILPHTNFGVREYIRAINNAVASLHRSRVMTKGLSFLKKYTFINKNVHHV
LMKLTPSPTAILKSLCKEFLSYNVSAILYLMNYEQYGRSTASAQYFLQLAGYLGIPVIAWNADNSGLERRASQSSLQLQLAPSLEH
QTAAMLSILERYKWHQFSVVTSLIAGHDDFIQAVRERVTAMQDRFKFTILNAVLVANKGDLAALVDSEARVMLLYCTREEAIDILS

AARDLHLTGENYVWVVTQSVIENPLQAPYQFPVGMLGVHFDTSSSSLVNEITTAIKVYAYGVEDFTSDPSNAGRSLNTQLSCEGA
GASRWDTGDRFFRYLRNVSVETEQGKPNLEFTQDGVLKAAELKIMNLRPGVSKQLLWEEIGVWKSWQKEGLDIKDIVWPGNSH
TPPQGVPEKFHLKITFLEEPPYINLAPPDPVTGKCSMDRGMLCRVASDVDITEVDMTQAHRNGSYYQCCSGFCIDLLTKFSEELGF
TYELVRVEDGKWGTSENGKWNGLIAELVNRKTDMVLTSLMINAEREAVVDFSVPYMETGIAIVVAKRTGIISPTAFLEPFDTASW
MLVGIVAIQAATFTIFLFEWLSPSGFNMKLSFNQSPSTSHRFSLFRTYWLVWAVLFQAAVHVDSPKGFTARFMTNVWAMFAVVFL
AIYTANLAAFMITREEFFEFSGIDDHRLSRPTSHKPMIKFGTIPWSHTDSTLAKYFKEMHSYMRQFNRTNVLQGVGDVLSGDLDA
FIYDGTVLDYLTSQDEDCRLLTVGSWYAMTGYGLAFPRNSKYLKMFNKRLLDFRENGDLERLRRYWMTGVCKPGKQEHKSSD
PLALEQFLSAFLLLMSGILLAALLLLLEHLYFRYVRKHLAKTDRGGCCALISLSMGKSLTFRGAVYEAQDILRNHRCRDPICDTQL
WKVKRELDVAVLRIKQLEKEMEMHGIKPPSPCKRVIVSGEYTRARLRHLNQSQGSDLDFSGTRTEIAEMETVL
>BlonGluR4
MFPRIDFVLCSVILCFSQVNSSHKLTIGGLYEDIELHKTAFFYETTVFNKEVTKNDSVLDPLIFPKSKPNHAFSALLSFCSFLNESAV
GIFGSQTSSNLEIVQTLSKKKQIPLILTRWIDNKPLGKFTLNFSPDPALLVQAYLDIVTFLEWDSFTVIYTDSASFLKIADFIKLTKDV
DIPVYVENLDPYHTNNYRPLLRKLRESGETKFVIDCPIRHLKDLLSQIQQVGMLTRNYNYFLTNLDADTQDLEQFMYNEAVITGI
HLLKPMEADAIGVLRELCHLYQVTFDDNCMSRVGKDFPELDVETALIMDAVQIYISVLKYANITGGRSIQCNSDDIWERGEEIYTL
IKEGFYAGITGKIKFGENGYRNAFELVIFQIREKFSEKGFWNTTVGLNMTVEHVEILDLEDIMFNKNLLVEITLRQPFVMLRESSYE
LRGNERYEGFAIDLIDKISKLEGFKYTLVLRNDGKNGEFDKALGKWTGMIGDVLKGEVDMAISDLTITGERVGPVEFTQPFMDV
GISILFHKPTEKPPSFFSFTQPFSTEFLQALGAAYLAVIISLFVIGRLSQTDWKKEETYMYNQLTLKNSFWFVTGSLFRQNTQIRIKS
LPARILVASWWLLCFVIVSLYIAYLMAHDTVTEKIHLFNNVEELVANAETHGIKFGALKGGATGSFFRRSSYSTYREIAAYMEKHP
EDMPTTAEEGIARAENENYAYFMESTSIEYITARHCHLTMVGGLLDTKAYGIALPKDSPYLTALNRAILKLGSSGELTDLKKTWW
EDRNSGRGCDISDQEEVRPRDLAHVRGLIFVTLGGVVIAFLCSIIEFHKNVKRICKKQKLPYGKILGSELSRSFGSRTKKTTYVKKS
NKDNRTVELL
>BlonIR21a
NMNKLKLFIILLPLVMSTEKRALQKSHEKSQWVKWSDAFLGQNKFDQQAYLVKLLKKIAEQYLQGCTTVILYDVFTELHDNLIL
QKLLAGFPTAYIHSQITENYKTSFKASAEDHSQNTCLSYILFIRDVMKVKDIIGDRSHNKIIIVAKSSQWRVLDFLSRQESQFFVNLL
VIVKSEDVGGRFVEAPYILYTHDLYIDALGSSKPVVLTSYQKNKFTRNVNLFPRKISRGFAGHRFIVALAQQPPYIIMRGRNSESDT
IFEGIEFRLMELLGTLYNFTLDYREATENTKIGSTEAVVKTIEKGNANLGIGGIYMTTNKIRRVGFTQWHSQDCAAFISLSSTALPR
YRAIMGPFHWTVWLAITAIYLIGIFPLVFSEKQTLRQLLNNPEEIENMFWYVFGTFTNCFSFSGSKTWNKADKLTTKILIGFYWIFT
IIVTACYTGSIIAFVTLPIYPEVVDSVKQLLDGRYRIGTLNKGEWRYLFLNSSDPYAEKLLVNLDLVSDIESGLKNVSKSFFWKYAF
LGSRAQLDYLVRTNFTTESKRSVMHISKECFVPFNVAITFPLHAIYGKTINDGLNLVIQSGILKKLKSDVEWQTMRSATGKLLAAN
SKIGSLKSLSYEDRSLTLDDTQGMFLLLGAGFLIATFAVLSEFFGGCLNIFTRKRQDSVSTIASNPRTHERQTPRDWQSVQYFRRCS
NHTAEVHQPNNVAKVLDDSPDTNIPVAFQVEENNASKDVDKVFERVFGQKCNHYESDDDNISSI
>BlonCG11155
MFIFHLTVLFVWNTPGYATLPPVIRIGAIFTEDQKNSSIELAFKYAVYKINKEREFLPNTTLVYDIQYVPRDDSFRTFKKVCRQMES
GVQAVFGPSDPVLGAHIQSICEALDVPHIESRIDLEPLSKELSINLHPSQENMNKAYKDLMTFLNWTKVAIIYEEDYGLFKLQELV
KVPAAVRTEMYIRQTSAATYRQVLKEIRQKEIYKLIVDTNPRNINHFFRAILQLQMNDYRYHYMFTTFDLETLDLEDFKYNSVNIT
AFRIVDVDHPKVKETLEIMENFQPIGHAILNRSGIIQADSALMFDSVYVFAKGLSAMDSGHSIKPTSLSCDIEKPWDDGLSLYNYIS
AVSSLHGLTGNLEFNEGKRSNFKVDLLKLKKEEIRKVGYWTPEGGVNITDPNAFYEHQVPNITLTVMTREEPPYVMVREDKNLT
GNARYEGFCIDLLNWIAGQVGFQYTIRLVPDHMYGVYDPSTKEWNGIVRELIEKRADIAVASMTINYARESVIDFTKPFMNLGIGI
LFKEATRAPTQLFAFLTPLAIEIWIYMVGAYILVSLTIWIAACFSPEEWKEAELCNACFTRKYYSMLTDNFEICSLEELPSIEIKEHFD
GSFCHRSLPTEAKESHGEKRLDVLENEFSIGNSFWFTIGSLMQQGSDLNPKATSTRIVGGIWWFFTLIIISSYTANLAAFLTVERMIT
PIESAQDLAEQTEISYGTLEGGSTMTFFRDSKIGIYQKMWRFMENKRPTVFVKTYEDGVKRVLNGNYAFLMESTMLDYAVQRDC
NLTQIGGLLDSKGYGIATPKGSPWRDKISLAILELQEKGVIQILYDKWWKNTGDVCNREDKSKESKANALGVENIGGVFVVLLC
GLALAIVVAILEFCWNSKKNAQTDRQSLCSEMAEELRFAVRCHGSRHRPALQRSCTKCSPATTYVPSALDLPHLNGEGVMLPLK
DMDKSPITCDVDT

>BlonIR93a
MVSLLLLTYLVMHRVGVAKTDSFPSLITANATLAIVLDQDYLGENYEAVKTEVENFLIYGKREILKHGGLNHVFFSWTAINIKREF
MAIFSLASCADTWKLFRSAEIQNVLHMAISEPDCPRLPPDKAITIPLVTKGQEVSQLLLDLRTESIFKWKSVVILYDNTLSPDMTTQ
VIRSLSQTTIYKENAIGVSLVKLPRTTTKTNIKSILSSINPKSVGKNFLAIVSYGLAAVIMEYAKKQELVDINSQWLYVISDTDDRYH
EMGIFDKLLSIGDNIAFVYNITSNNQSCMGGRTCHIENLLTGFFGALEGSIKEEFEIAAQVSDEEWEAIRPTKLERRSFLLLSIKKYL
KNVASCDNCTYWKIQTSESWGVEYENNDKDAQPFFIPVGIWRPVDGPFMNDELFIHVSHGFRRKTLPLVSFHNPPWQILKVNNS
GEVTEYSGLVFDIMKQLAKTLNFTIKVESIDKQKIEVNRTKLINSSIESVLTNNIPGIIIEQVKNRSVAFGACAVTVTSSLKQEINFTIP
ISTQVYTLLVARPKELSRALLFISPFTGDTWLSLAAAIVTIGPILYCINRYSPVYEYEGIPKKGGVSSVQNCIWYIYGALLQQGGMH
LPYADSARIIVGSWWLVVLVLATTYCGNLVAFLTFPKIDVPIVTIDDLIKHRETVSWSIRNGNYLESELKQSREPQYKILYERQYKY
NNRDLGVMNKISQGKHVLIDWKMSLLYIMKSHFQETGRCDYILGHEEFCDEQLALITAHSTPYLTKINEQIKWLHQVGLIEKWL
RDYLPRKDRCWKNKHIVEVNNHTVNLDDMQGSFFVLFLGFVFALMLLVFEKIWKGHFAKKSQKIIHPFVS
>BlonGluR2
MSVAVVFVLKLLVLLRIQYVCSAKDRLSVGFLFNDLKTQSIYPLNSTMHKKIMYTMPVDYKSVIQEISSIDSFEASKTLCNVLKTD
EGVGVIFGAESSVTTPVLDSIATNFGVPYIMTSLYYPNDQEDRYSFNFFPHADLFAKGLAEIIKHHQWTNFAILYETEEGLAKMQE
VLKLQEFQKDGKKNRIILKQLGPGPDYRPLLKRVNSTEGNIILDCKTENILPILEQAKGLKMLDLTDSYFLTSLDAHTLDFSVLDTT
ANITTVRLFDYNTEQFKNAVRRWEIMEFDLNPTRSMYHIEPRSIKTETALYQDALTLVTDSINDISFESGIQSVPITCSGEEKSPDGR
KISMRMKSRPISMSLTGPLTFDNFGNRIDFNIYIEEAITGQSIGIWHARNESTTFIRTLNETIDAAVLNLQKNKVIVSSKIGEPYLMHA
VPEDGQVLEGNARYVGYSMDLIAGIARIIGFEFEFHLTSDNKNGNWDAHTRRWTGVIGDLLERRAHLGICDLTITHERREVVDFS
MPFMNLGISILYKKPDKKDINMFAFLDPFSITVWIYTVTLYLVISIILFFISRMTPGDWENPHPCEEEPEELENMWGIKNCMWLTLG
SIMTQGCDILPKGISSRLTVSMWWFFCLIMSSSYTANLAAFLTKANLEAPIDSAEALAKQTKIKYGLLKDGATQSFFQNSNLSLYQ
RMWQSMKETRPSVFVTDNKDGVNKVLTTKNSLYAFLMESTGIEYELQTKCELKEIGNHLDSKSYGIAMPMNAPYRSAINKAVL
KMQESGELGELKKKWWKEQRSEPSCDEQETEEEDDGGGLALANVGGVFLVLGVGIALAYGLALIEFLWNVRNVSVEEHISYW
GALKLELKFACAIWITRKRAKPLLSESSSGRSDKTDKTDNKSIEQSILKNSDSTHNS
>BlonGluR1
MNQIFTIHVFLWWVFQIHLCIGSQVIPIGVLFDKHQSQAEIPLNSTLFLGLRTYKRTQTFSANVHRISSIDTFEAGKTVCNAINSQDG
VTVMFGATTSISTPLAESICTHFNIPYIITSWRESFYKPSNVILNFHPDADLFAAALTKIVESLDWQGYYILYESAEGLMRLQEVLKL
KRFKEKNTQQIIHVKQLEPGNDQRTLLKNIRNCTSHIILDCKTENIVPILQQAKEINMLNDHFSYLLTSLDAHTIDFSLLDTRANITV
VRLFDFDTDTIRNTFREWELSQYEMFNRKLRVRESAVQTETALFADALTYVSQALEDLATESEITTEPLDCEHDRKFNMGNSIVE
KIRSINKLDTLTGPIKFDENGNRIDFNLHLIDVRTQRKLAIWFQGNSSLTLTRSAEETSSAALSTLTGTIVRISTKLGEPFLMEVKPKE
GEVLVGNARYKGFSKDLMNGIANLLNFTYEFFLTADGQYGNYDVNKKAWNGLIGDIWRKEAHLAVCDLTANHERQKVVDFSTS
FMTLGISILHKYADKKDVKTFAFLDPFDTSVWIYTATLYLAVSVILFFISRMTPGDWENAHPCDENPPELENIWGFMNCHWVTLG
AVMNQGCDILPKGISSRVAVSMWWFFALIITNSYMANLTAFLTKANLEPPIDSAEALAAQHKIKYGVYGGGSTEAFFRNSNVSLY
RRMYETMKSSPTVFATSNKDGVARVLNSQKAVYAFIMESSIIEYEIETKCELKQIGGWLDAKNYAVAMPMNAPYRSSINQAILQLQ
QSGELVRYKTKWWKLERNETSCDVTQRDDTDASLTLAEVAGIFLVLGVGIGVTCIWGFTEFLWNVRNISVEEHISYWEALKVEV
KFACNIWITKKRIKPEISESSSSKSDKTDNRSIIQNFIHSASSFMNINQT
>BlonIR68a
FSPLMWTGVLICLVICILAFHYFARFHNSVTYPKTIESHKPVKSEQHTTILLIYPEIYKLDSNMKYTLMREKYQVSKKDAEVTGLY
QFLEPVNSALYTYSMLLLVSLPKLPTGWSLRVLTGWNWLYCLLVVTSYRASMTAILARPTPKVTIDTLDELVSSKLTYGGWGEIN
KEFFKASFDQTIQMISDNFILVNNSEEAVEKVAQASFAFYENTYFLKEAIVKQQNSVKFRDTNNTNTTTYKSRTSQKENRNLHIMN
DCIIIVPVSLGLQKNSPIKPRIDKIIRRVIEAGLIKKWIDDVMQNIIHTLMQKDNKNTKALMNMKKFSGAFVALVIGYFLSIIMLLCE
ISYFHYFTMKNPHYNKYSRQIEINQNL
>BlonIR8a
MRGVCPVIAKYEPRITLDVSWLKTETNCNLFNNSSYIYLGIDASLRPFIEFLETYLKIRNVESVVIILEQSDELDQIIFPLLNTTRLK
MYIIESMDSTSIKKIQDLKPMPTSYVLLASTTSVESFLTLESTEHLLKLPDRWILMCTDVHGYKIDRYLLKNKLISLLTIDNKLCCV

NKTFLTTCTCEENLNLQKSFLQLVLQNLISNLEVTFKDQLLNYNSQISGFRLDEFFNDTFLTEYSYLIGSTLKMNISGNIEIGNNISS
KVVGKYENKELIILENSTMKPIKAFYKVGITHALPWSFKINNSVTGTSIWSGYCVDFTSKIAEMLNFNYEFVEPNNGTFGEKINGI
WDGVIGDLVTGETDFAVTAITMTADREEVVDFVAPYFEQTGITIVMRKPVRKTSLFKFMTVLKLEVWLSIVAALVATGCMVWLL
DKYSPYSARNNKAAYHYPCRDFTLKESFWFALTSFTPQGGGEAPKALSGRTLVAAYWLFVVLMLATFTANLAAFLTVERMQAPV
QSLEQLARQSRINYTVVKDSDTHKYFINMKNAEDTLYRMWKELTLNATTDDLRYRVWDYPIKEQYGHILLAINDSNPVANAEEG
FNTVNAHLDADYAFIHDSSEIKYEISRNCNLTEVGEVFAEKPYAVAVQQGSRLQDDISKVILKLQKDRFFDELQAKYWNHSSKGY
CPSTDDNEGITLESLGGVFIATLFGLALSLLTLVGEVIYYRKKRKSNTMRQKSQKIGSGKLSLLPPPFIDKGKNPGANKNTTKQKL
EKSIMNKVKEESRIKYPNLHSRNTNKIHIMKESFY
>BlonIR76b
MGLIEVVLAGLCLNATCDVSERTQLEPSNYDTGRESLLALAEDLSHETLRIATFKNGELSGYVNQSGQVIGSGIAFEIIDILQSKFK
FNYTIVVPESGLFLAASRKNGAKDLLQKDDADMAAAFLPVIHAFRNDIQYSFSVDITEWVVLMNRPHESANGSGLLAPFTTPVW
ILIILSILIVGPIIHFIMWLYSKLCKDDHAKVYACHRSTWFVYGALLKQGSPLNPQTHSSRVLFATWWIFILILTAFYTANLTAFLTLS
TFTLPINSAEDIARKHYHWVTNKANGIREIIYEENILGTYQKKLVDVIGNDNNFPDKNDFAILDQYVAKKGMMFIREKSIVDRVLY
EDYKEKTKNGIEESKRCTFVATKFSIVSSHRAFAYSRNFKYSVLFDRAIQQLVESGIIKYKMRENLPDAEICPLNLKSTERKLKNTD
LLLTYEIVGTGVIIATIVFLLEHLLHVTIKKCKERREAPRDHALFKKNNRNLSKFNIPSITPPPSYNALFMPPFAFNPQQGVKKHING
RDYWVVNKNDGFSQLIPLRTPSALLFQYSQ
>BlonIR25a
MKCVIFTYVVIPSFLMCCSGQTTQNINVLFVNEEGNEVADKAVDVAMTYLKKNSRLGVSVDLRKVVGNRTESNVFLEALCSTYN
QMLETQAYPHIVLDTTMTGLGSETVKSFTGALGLPTISASFGQEGDLRQWRNIDDVEKQYLIQISPPADIIPEIVRTIVLNQNISNAA
ILFDNTFVMDHKYKSLLQNVACRHIITPIKGGVQSLADQLTQLRKLDIVNFFVLGSLNSIKNVLNAADSVSFFNRKFAWHAITQDD
GEVKCACKNATIMFAKPSPNAAFMDRLGTMQRTYQLNTEPIIASAFYFDLALHAFLSVKNMIANGDWKKNNVTHYISCDDYNG
DNTPKRYGLMLRRGFNLESSEAPTYGPINLISNGLSYMEFQMQISSVGVRGGASDKSLNLGTWSAGFDNNLTLIDPQVMSNFTA
DLVYRIVTVEQKPFMFRDEKAPKGFRGYCIDLIDKIAEILNFDYEIVAVDSFGIMDENGKWNGVIRELMEKRADVGLGSMSVMA
ERENVIDFTVPFYDLVGITILMKLPETPTSLFKFLTVLENEVWLCILAAYFFTSFLMWVFDHWSPYSYQNNREKYKDDDEKRVFN
LKECLWFCMTSLTPQGGGEAPKCLSGRLVAATWWLFGFIIIASYTANLAAFLTVSRLDTPIESLDDLSKQYKIQYAPLNGSSDQTY
FERMAHIEMRFYEIWKDMSLNDSLSEVERAKLAVWDYPVSDKYTKMWQAMKEAGLPSTLEEAVDRVRASKSSSEGFAYLGDA
TDIKYLELTNCDLTSVGEEFSRKPYAIAIQQGSPLKDQFNTAILQLLNRRDLERLKEKWWNKNPEKMDCEKVDDQSDGISIQNIG
GVFIVIFVGIGLACITLAFEYWWYKYRKQVKVVDIRRQSDFSSKLPFRKTNNANKDKGKNQKLGLSTRPRF
>BlonIR41a
MSEMDIWINILSNLIIKTYFQDSNCLFIFTDKENAFQYVGDLPVVNVETKMSNLSPNIFLQHFGCHGIIIRSDHPVSQFKSFEREIKF
AKERFNSRKFLLLPGNNMKENFSDILQCPELMYVADLDIVELTNNDNGFIFTIWTHFYVGIKSEEKKVLDVWFSNNSTFLYENNL
YPDKLSNQMGRNIRMATFLYEPYSIVDDTVSEYKGSEMSVALTFAKKYNLTPLFVVNEEDFWGEIFPNWSGNGLLGNLVLDIAD
MGFGALYTWENEYKFLDLSQSLVRTGVTCLVPAPKIAAGWLTPLYSYSLKMWLAVGSLFLICVPTIFLLYYSHQGLNVAKNIETKP
KTVTELIVVSTSIIFKLFLLQPINKNEIPRNICGRYFVGLLFIFELFLTSTYSSGLASIMTIPRYENPINTVDEFYRSGLYWGATQDAWI
VSIQNATEHKYQEIVKHFRSLLERELRKLSVQGDFAFSIERLPGGNYAIGSYIKRDVIDNYHLMQEDFYWQQCVFMLRKNSILLP
MLDSFILRVFQYGLISYWQNEAVSLYMDPYVQRVVRYYFVTHDKKNTVIKLKWLHVEGAFGILCLGYILSILIFCLEIFLYKISQEK
GQLLYLP
>BlonIR75q.2A
MEVVVQFLDQQRTSAVVIGYVCWPIGQKVTFWKLLSKHGYAATFSDEVVYHNLANHHQIFVLDTLCPGYKNILNKAKKLKQFN
YPYRWILINYKENNSFLNDFYFGIDSRVFLINEMHQEYCIKSIYKISSKELTFKENYIGLWNNISGFTEYNDFIVSRNRSNLEGLTFN
LAYVTTANDTLLHLEDYRNRHIDATTKVNWLLLPFLFDLVNATARQYFLTTWGYKNTTTGLYNGVVGDLQSGFAEIGGSSVFITA
DRLEFITYITATSDTDIKFIFRAPPLSYVSNIFTQPFNRSVWYCSYAILALLFGVIYLVINWEWSDPLFSETIEGIPDILRPVFFDVVMA
EVGCITQQGIDKEPKSFAGRICTILTLAVFMFLYTSYSANIVALLQSTDENIRSLEDLLYSKISLGAEDIPYNTYYLKNAEGAVKKAI
YETKIAPKGQKPNFMSPEEGMRRVREGFFAFHTELTSGYKIISDTFHESEKCGLRAIRYLSIILPWLPVRKNSTYKEIFKVGMYRIR

ESGIQRRVYLRIYYEKPICLSKGNNFVSVGILDCYGAFMILGIGLLSSLIMGIAEILLHKFMNDKDMVIIKRSKKKSKKRNQNIDQG
YNSLMRNVNIKQVW
>BlonIR75q.2B
MDLNCDNVEDILELASRSDKFYHPYRWMFIGHFNITLFQNLRFSLDARLHVVEKLEGGDNYAIKSFYKLAKESDIFFDNDIASWS
KGSGLFRLNEFSLSKNRSNLLGRTLNVSYVVTNPSTFNHLEDGREKEIDTITKVNWIITKHLLSTVNATSQPIIQTTWGYKNESTGL
YSGLIGDLQTGRAELGGTPSFFVIDRLEIVDFIAATTPTYMKFIFRAPPLSYVTNIFTLPFQSYVWYCTFALVAITFIVIYIISKWEWT
DPLFKRDAATAPYTLRPEILEVALLEIGAITQQGSDFIPKSTAGRIATIITLTILMFMYTSYSANIVALLQSTTDSIKTLDDLLKSRISLG
VEDIVYAHYYFETANEPVRKAIYQQKISPKGQKSNFMTLEEGIKRVQKGFFAFHVELARGYKVVSDIFQENEKCALKEIAFVNLIE
PWVPVRKKSPYKKIFKIGLHKIQESGIQKREVNRIYMKKPTCHSKGSNFGSVGLIDCSGAFIIFGVGLALSFIIFIIELLVKRYKPNSN
FKKVFPIEEAPEKWKNSNISDNEVFEYND
>BlonIR75s
MNLRCVLLHFCIVCMNVDAAMYQDFLIDYLKTFNKPVTLTEYSCSLTDSVLLVKKLMNSGITTRVLQAHHDEVQHENVPNSAVL
LLNIRCSQAARILLTAAEMELFAHPYRWILFCENNCTNLLDNMDISVSSDVNLCKYNGVDRITVEKIFKYNTNEDIVSEEWGYWT
KTENFVLNNTEKNLFRRRQNLKNITLNTCIVVTNNDSLKHLTDKRDKHIDSITKVNYVLVEHLSDIMNITLNYSIQSTWGYKNNK
SEWTGMIGELVVKNADIGGTALFFTIDRVDIIDYIAMTTPTRSKFVFREPKLSYVTNVYMLPFDDFVWASILSLLIIIITFLYFILKWE
WRKQEFQEIKDESTIPELRDSLDDIVLFTFGAMCQQGGPEIPLSVPGRITTIMMLISLMFLYTSYSANIVALLQSSSNSIQSLADLLK
SRLEVGVDDTVFNRFYFPNATEPVRRAIYLQKVAPPGKKDRFMSLEEGVRRLREGLFAFHMETGPGYKLVGEIFEEGEKCGLKEI
QFIQVVDPWLAIQKNSSFKEFLKIGLRKIQESGLQSREVGLIYTKKPICISRGSNFISVGIVDCYPALIVLISGMFLAFIVLLVEKLCW
KRKILQKKTKQKKIDPNKMINSSNHDFLWTYME
>BlonIR31a
LVDKNIVDISAAGGLLREARIPCYDFLLSYFMFRARFFFLDPGIVKPGTEVLKPFSTTTWYATIAIGILIGLAIKCAYWIEWRFLKSR
TNYSAVTSVVITISVFAQQGSAIFPTLLGGRIIYLNLLILSILLYNYYTSSLVSSLLSSKPDVFETIEELIDSDLQLGIENQPYTFTYILQ
RSGDFYIRKLNRSKIYRTNKFLTPEEGIRKVKEGGYGYHT
>BlonNmdar2.2
SEIPLMAFLLPFSPELWIAIFTSLNITAIAVAIYEWLSPFGLNPWGRQRSKNFSMSSALWVMWGLLCGHLVAFKAPKSWPNKFLINV
WGGFSVIFVASYTANIAALIAGLFFHNTVGNYHDRSLLSQKVGAPRSSAAEYYVQKANQLLWEHMSKYSLAHVEEGIERLRNGS
LDILIADTPILDYYRAT
>BlonNmdar1
GVLLNSGIGEGTPRSFSARVLGMVWAGFAMIIVASYTANLAAFLVLERPKTKLTGINDARLRNTMENLTCATVKGSAVDMYFKR
QVELSNMYRTMEANNYDTAESAIDDVKHGKLMAFIWDSSRLEFEAAQDCELVTAGELFGRSGYGIGLQKGSPWSDEVTLAILDF
HESGFMESLDNKWILQGNLQQCEQFEKTPNTLGLKNMAGVFILVGAGIIGGIGLIVIEMAYKKHQIKKQKRMELARHAADKWR
GAVEKRKTLRASSTAQRRIKSNGVNEPSTISSNVLDKFNRIGQLYGPERAWPGDSDIRQRRLEDMAGGGMPNVPRYLPAYT
>BlonGluR3
MIFQWFVLVLLTFVTCDEINFASLFEQNENHLEKAFNFAVQSVNNEKLEDDPELSIIVHTDLQPDEPFEALRHTCDILEKGTVAIFG
PSSYDNIEIVQSICDAKEIPHIITRWKWPIRGGPEINFYPHPPQLARAYFDVIKAWEWTTFTVLYEDYEGLSRINHLVQEAKDFGML
VDIKKLDSSVTGHYRDTIKEIKLSDQKFFVLDCHIDNLDTILRQFQQIGLMNEEHNYFLTNLDAHTENLEPYQYSGANITGIRIINP
ENDLVEKISEELYADEPEISGTGLAAWKLRTEPALLIDAVHMFSQALSDRRKQSSVPLANGSNSLNCEETKSWEHGFSVVNMVKT
NVYDGLTGTIRFNTEGFRSDFGLNIFELKEGGITDIGHWNYSSGVNLARMFPNQSIDAGDSLQNMSFTVIITLTEPYGMLKESPEN
LIGNDRFEGYGIDLIKKLAEMEGFNFTFIVREDKANGVYNPTLGKWTGMIGDLLEMRADLAITDFTITAQREEVVDFTVPFMSLG
ISILFKEPKNAPPSFFSFADPFASDTWLALVGSFFFVSFSFYIIGRMCAEEWTNPYPCVEEPEHLSNQFSLWNSIWFVTGSIMCQGSE
IGPIAMSTRMLAGVWWFFCLIIIASYTANLTAFLATENRVELFKDLQSLYENKHGVKYGAKEGGATLSFFTRAEEGTLFRKIGDFM
EEHPENVKENEEGVKRAESEEENFAFFMESTSIEYTVQRHCTLKQYGGNLDEKGYGIAMRKNSSYRKRLSLAILKLQSNQWLDQ
LKRKWWEERRGGGQCQGQDESSEADPLDIVNCEGCFWVTIYGTILSALVVILEYLIYIISVSRKSKLPFCEVFRQEMSVYIDFNSE
SKAVITKADFSKEETKSKSSEKTDKSKTHTKSRSRSRSPHSKCRSVKKRSNSKSTGNKSPLPYGFIYSQSTERLQSTP

>AquaIR1

MLSILERYKWHQFSVVTSLIAGHDDFIQAVRERVSAMQDRFKFTILNAVLVANKGDLAALVDSEARVMLLYCTREEAIDILTAARDLHLTGENYVWVVTQSVIENPLQAPYQFPVGMLGVHFDTSSSSLVNEITTAIKVYAYGVEDFTNDPTNSRRSLNTQLSCEGAGAARWDTGDRFFRYLRNVSVEADQGRPNLEFTPDGVLRAAELKIMNLRPGVSKQLVWEEIGVWKSWQKEGLDIKDIVWPGSSHTPPQGVPEKFHLKITFLEEPPYINLAPPDPITGKCSMDRGVLCRVASDADITEVDMSQAHKNGSYYQCCSGFCIDLLQKFSEELGFTYELVRVEDGKWGTNENGKWNGLIAELVNRKTDMVLTSLMINAEREAVVDFSVPYMETGIAIVVAKRTGIISPTAFLEPFDTASWMLVGIVAIQAATFTIFLFEWLSPSGFNMKVSFNQNPSASHRFSLFR

>AquaIR2

MNRAFRDLMTFLNWTKVAIIYEEDYGLFKLQELVETPAATRIEMYIRQAGPASYRQVLHEVRQKEIYKLIVDTKSRNINQFFRAILQLQMNDYRYHYMFTTFDLETFDLED

>AquaIR3

MQGVADVLSGDMDAFIYDGTVLDYLTSQDEDCRLLTVGSWYAMTGYGLAFPRNSKYLKMFNKRLLDFRENGDLERLRRYWMTGVCKPGKQEVKSSDPLALEQFLSAFLLLMSGILLAALLLFLEHLYFKYVRKHLAKTDRGGCCALISLSMGKSLTFRGAVYEAQDILRNHRCRDQICDTHLWKVKRELDVAQMRIKQLEKEMEVHGIKPPPPCKRVIVSGEQARARLRTLNPDKGSDVDLFGHRTEIAEMETVL

>AquaIR4

MRMFLAEILIVLLMLYVLRTGGYGKPKYNIGGIFYDETQEIAFRIATYSMNNKYPVSQIELVPHTYRISAHNTLEAYRATCELFENQTIALFGPNSASSSPFIQSLCDSKEIPHIETHRSLNLERNDTLVNLYPHPEMLSICFMDLIEAFDWKKVIIIYDDEESLLSVSSLLDLNNRKGRKVLLKQLESAGAKGNNFRPMLSEVKALGETQYVLSCSTDILEDVLKQLQQVGMMTEIYSYLVTDLDMQTVDLEAFRYAGTNITGVRIVDPEQTDVKQIALIVQPNGDGHKVRVETALIIDAVTLFYETMYDLTIDKRMKLKALSLGCASKNSWSDGYTIMNLMKSKSMQGITGLVKFDLEGFRKDFTLDVLELFPDGLLKIGEWNSSIRNIYLNRPDRVKYGNNNPGDNLFNRTFTVLISITPPYGMLKETTMQLNGNDRYEGYGVDVICELSKILGFNYTFVLQEDGLYGNFNKTSGQWNGMLRKIIDERADLAITDLTVTSERENAVDFTMPFMNLGISILYRKPEPVPPSLFMFVSPFSFNVWILLAVSYFVVSISFFVMGRLSPTEWQNPFPCVEEPEFLVNQFTVRNSLWFTVGALMQQGSELAPIAISTRTASGVWWFFVLVMVSSYTANLAAFLTVTTLVTPFKDIEELANQQEIQFGAKKGGATAQYFRDSNLSKYQKVWSYMVQHPELMMDDNDAGVTKVERENYAFLMESTTIEYNTERHCSLARVGGLLDDKGYGIAMKRNSPYRNDLSTAVLQLQEKGILTSLKIKWWKEKRGGGKCSTKSEDSEATPLDLQNVGGVFLVLFVGAILGTIGSFAELALRLYRRSHRDKVSFKEELMKEMRFFVQFKLNVKEIDGTPHDSTSNKNLISSTKSLDTVKIS

>AquaIR5

MFWEICFLSLVFHHGLGTITKLNIAGIFEKVQLHQSAFLYSNQLQDIQRQVSNTTLLPLVDVHVFPDDAFSARKATCSFLEKHIVGIFGPQSSSNLDIVQSITDRKEIPHILTRWVHPSHMAPETINFYPSPTRLAEAFLDILRKLEWKTFTILYTDFENLIQITDFINEAKDQGFIVYMEDVNAYQDGNYRPILQEVMKSGQKNFVLDCPIQYLTELLAQIQQVGMLTTDYNFFLTNLDAHTKAHTGDLTPFMHSDATITGVHIMKPEDDLSTRVSKELCHLYKVTFNEDCGSNALHDYPELDSETASIIDAVHVFSDALNAAGVSQSQALDCNGPDSWQNGLSIINALKSNTFEGLTGTIEFGNDGFRRTFQLTIFQLQDGRLVDRGSWNTTSGIDMNIDMRAIDVEDSEESMMNKKFVVLITLTKPYAMLKETPERLVGNDKYEGFAIDLIEEIAKIEGFEYSFKVREDNKHGVFDPVSGKWTGMIGDIIEMGADMAISDLTITQDRLDPVEFTQPFMSTGISILFHKPTEIPPKFLYFTEPFSAAVWQALGLSYLALVLTLFITGRLAPTEWYQRPNKKYLVNQLSLSNCLWFGAGSYFRQNADVRMSSLSSRIISACWWMICFFTLAMYIAFSISRSSIAEKEVLFNDANELVEKSEQYGIKYGALHGGATQAFFESSASDVYKEISTYMAEHPEDMPSLTEEGVEKAEKENYAFFMESATIEYVIKRHCRLTTYGSLLDNKGFGIAVRKGSPILTPLNKAILTLHTSGDILRIKRKWWEERNVKEICEDDSETDAAPKELSHVIGLLWITLFGTLLALVCSVVEFSLYVYSLSTKLKQTFGRTFFQELGSSFRRKRIHREIQPILLVKTENDQNASPESVEMS

>AquaIR6

MMESYQGVSAVFGVDSPNTFPILESICTNFEIPFITTSWKPTIIRNPDQARALLNFHPEADLYAKGLAEMVKSLEWASFVIVYETEEGLIRMQEILKLQELKKGNKKNTIFVKQLGPGPDYRPLFKEIRNTSEDNIILDCKTENILPILLQAKSLNMLSLHNRYFITSLDAHTIDFTVLNTTANITSVRLHDPHSDDFKNIIHRWELTEFENRNIHMQLDPRSIKTETVLFHDAILLLSDSISDLSVKHGLRTNPISCRENETSLNGFSLRNYMRIRTPSFTLSGPISFNENGDRIKFNLHLVDTVDETVLATWFASNKTVKVTRNYDETVDAAILNLQKIKVIVSSRLS

PPYLQKREPSYEGEELVGNRKYMGYSMDLIDGIAKIIGFQYEFQITEKHGSYDYDLKKWTGLIGDLLEKKGSSCYL

>AquaIR7

MCNNVFRVNLIKTSQFLVMVEMFLGMITLWAFIQGLICEEVNIAGLFENDANLEKAFLHAIDLLNENQNEDEFTFTPLTQNEILEN
EPYSALHHTCLLLNLGVVAVFGPRSFDNIDIVQSVCDSKEIPHVITRWNYWSSRQSSEINFYPHPPLLTKAYLDIILSWEWKTFTVL
YEDDESLVRVTGLIETAKSRGVVVQVFQLEPGNYRAIIRDLKNSGEKFIVLDCQIEHLIEVLTHLQQAGVMNEHYNYFITNLDAHT
EDLTPFMYSNANITGIRMINPDKESVQRSTRKLFSEDEFTGAWKLKIEPALIIDALQMFADIFNHRQNLSPVSIVKNSNSLPCYDTG
SWEHGYSVVNMLKTSSYDGLSGLIRFNNEGFRSEFLLYIYELREGGLTDVGNWNSTSGLNLTRSHSSQEISDEESLRNKSFNVLIT
LTEPYGMLKQTTNLLTGNDRFEGYTIDLIQKLSELEGFNYTFIVREDKKKWSV

>AquaIR8a

MLLPQKKLREFRPTPNNYAIIASTKNMQKLIRVAFRENLVTLPERWNLVFLDFQHKSFDRSLIMSNPVNLLTLDTELCCQLLNQNT
YCECPGSFSAPKELLRIAINVLANTFEELLQKGVQLGDIECDTNTTNYNETSLKHFEEIMNDFVSKHNTIGMDNSILHLKATGSIGI
GNEVFAKYKNETISVIGNKTVKPIRAFYRVGITHALPWSYKIKDSDTGKWIWTGYCADFTAKLAEKMDFDYEFVEPTKGTFGKR
KDGVWDGVIGDLASGQTDLAITAIIMTADKEEVVDFVAPYFEQTGITIVMRKPVRKTSLFKFMTVLKLEVWLSIVAALIVTGFMV
WFLDKYSPYSARNNKKAYPYPCRKFTLKESFWFALTSFTPQGGGEAPKALSGRTLVAAYWLFVVLMLATFTANLAAFLTVERMQ
APVQSLEQLARQSRINYTVVKNSQTHKYFINMKFAEDTLYRMWKELTLNASTDDSRYRVWDYPIREQYGHILLAINDSNPVASA
EEGFRNVDEHLDADYAFIHDSSEIKHEISKNCNLTEVGEVFAERPYAVAVQQGSHLQDAISKMILLLQKDRFFEELHAKYWNNSA
KGDCPNTDDNEGITLESLGGVFIATLFGLALAMMTLAGEVLYYRRKRRNTQLKDRESKSKVFPGKAQELFHQKQFPLESNSITIG
STFKPVNLNEKIRREREELKISHITLYPRARTRVSQAEKN

>AquaIR9

MNDYKYHYLFTSFDMETFDLEDFKYNFVNMTAFRIVDTEDLSVRDMLRNMMKFQASEGVQLINSSNIQAEAALMYDSVFVFAV
GLQMLDQSHNLELLNISCDKAQPWDGGLSLINYINAVELKGISGPIGLKEGRRIQFKLDLLKLKQHALVKVGEWHPGSGVNITDR
EAFFDPGIMNVTLVVTTILETPYVMMHSGKNYTGNSRFYGFCIDILERVSQEVGFDYLLDLVPDRKYGAQDHQTGSWNGMVLQ
LIQHKADLAVGSMTINYARESVIDFTKPFMNLGISILFKAASTRIVGGVWWFFTLIMISSYTANLAAFLTVERMITPIENAEDL

>AquaIR10

MYGVYDPDTKEWNGIVRELMEKRADLAVASMTINYARESVIDFTKPFMNLGIGILFKVPTSQPTRLFSFMNPLAVEIWLYVLAAY
ILVSFTLFVMARFSPYEWNNPHPCHQESDIVENQFSVSN

>AquaIR11

MIQSVSASLQIPQFQTFWNPKLRRPLAEPSASNQIFNLHPSPRTLSQALATLVRENDWKSYTVIYENDEGLLRMQEALKQRSPADP
AVAFRALGPRENHRSVLKEVKSSGVLHVILDCDAGRIMDILRQAKKLKLLSEFHSYILTNLDSHTLDWSEFKHIRTNITALRLVDP
DSSSAQHAALVWNQIMKIDILRNIQNPAIQKELENYRKLNPVAEILPQNIPIRTALLYDALNLFVSTFSKLDAEEELILDPLSCGTNE
TSSHGRRFSEALTEKKRDIKLIVEPLTGQITKFDESGYRRKYKLQIIEFDSTKFRVTGTWDSDFPQVIKLKLSEEDRDTELKKKIQQ
RTFRVISRLGDPYLMRRTDPNGRPLFGNDRYEGYTMDLMGEICKPQNLNCSFSFEIVADGKYGNYDPITKQWNGLIRELLDYKA
DLGVCDLTITYERRKAVDFTMPFMTLGISILYAKAVKEPPELLSFSHPLSFEVWVYIATSYLIISMMMFLVARLNPNDWENPHPCNP
RPTELENIWNVKNCFWLTMGSFMAQGCDILPKGISTRMVAAMWWFFTLIITACYTANMTAFLTMSRMGPTIESADDLAAQTKIK
YGCLGGGSTSSFFKDTNFSTYHRMWVQMESAEPSVFESNNKDGVKRVLTSKRKYAFLMESSSIEYEMERNCELMQVGNNLDSK
GYGIAMPTNAPYRKSINEAILKMQEMGLLHKLKDKWWKEMNGGGQCTKDKIGHDETANEMGLDNVGGVFVVLAAGVAFAFV
IAVCEFLWNVRKVAVVEKLTPKEALIKELRFAMDISSRKKAVFAPSRAISLENIDR

>AquaIR12

MFAFLDPFATAVWIYSATLYLVVSVVLFFIARMTPGDWENPHPCDEQPPELENIWDIKNCHWATMGAIMNQGCDILPKGWSSRM
ALAMWWFFALIITNSYIANLTAFLTKDKMDPPINNAEDLAKQNKIKYGMLEGGSTENFFKDSNDSIFERMYINMKSQRPSVLEKE
NKDGVARVLSTKNGLYAFLMESTQIEYEIEKNCSLRQVGDWLDSKSYGIAMPMNAPYRGAINKAVLRLQELGNLTALKTKWWK
KAKKRGIL

>AquaIR13

MGIFEAGIITKMTENEYEKLGKQKELSSSIAENVQKENTKESRRQTKVNEETDELKPISLKMLQGSFYILCFGNIFSGMILVAELMF

HKNQITHKSKRKTSIKVKKLGELIRVHINQLRSFLRRLHQNIMHDAFLSTLEYME
>AquaIR14
MTFFRDSMIETYKKMWRFMENRKPSVFMPTYEEGIQRVIEGNYAFLMESTMLDFIVQRNCNLTQIGGLLDSKGYGIATPKGSPW
KDKISLVILELQEKGEIQMLYNKWWKKSGETCEKNEKKKGSKANSLGVDSIGGVFVVLLCGLAFAVLIAILEFCYNSKKYKKFQ
GQSRAPNQSLCSEMGGEFCFALKCCGSRQRPAIRRRCSKCLPDITYVPVKNSRHQTHPTVESVPNSQQIFEETRIREVQVRS
>AquaIR15
MTGINFKSAVVMPTLDMPLKEYLASDNDRQFNSMHRFQSVTVNHCKDLYNFSLDIQRTNSWGYIQANGRFDGLVSLLEKRQVD
FGSSPLLYKLDRMPYVDYSYGNWILRSTFIYRRPKVTAKSYEIFLRPLEKDVWITISII
>AquaIR16
MNNPFESVSATCQLLQEGVVGILGPFSEDNSNVVQSVCDLKEIPHIEVRWDDYPLNGTVVNIHPYPDTLTRTYYDIIVGWGWEDF
VILYENNESLQRVGELLKLFEPAKQRIVVRQLDAKEESEEGFRTVLKEVRKSGATHFVLDCSNEILEEVLRQAQQVGLMTDKHNF
IITNLDLHTINLTPFKYSETNITGMRCVDPDKFLADEMDPLAGYQLKLEEALIYDAVKMFAEAIKSVGRMVQPLSIDCYSYEDRLK
SGTTIINFMRNLEYPGLTGPVKFDVRGFRTDFGLDIFELMEGGQTIVGNWNSTKRPHLNVSRVTVKGEDVNDDIRNRTFKVMITL
TEPYGMRVESLEPLYGNDQYEGFAVDLIKLLAEMRGFNYTFVLREDKANGKFDNSTGKWTGIIGDLIDGNADLAICDLTITMERE
AVVDFTVPFMMLGISILYKKPTKAPPSFFSFADPFAFEVWELLMVAWIGVSLILFVVGRISPGEWENPYPCIEEPEFLVNQLDFRNC
LWFVTGSIMQQGSEIELKSFSTRMIAGMWWFFTLLMVSSYTANLAAFLTTENPDPHFTNFKELVENAERKGIKLGAKRIGATESFF
EDKWKADPTSDFGKAWTLILKDRDKIKIPDNSDGVFHAQQGYYAFFMEDKSIEYETQRKCELNQVGGKLDEKGYGMAMRKNS
TYRNSLSTAILKLQNSGKIDEIKRKWWEERKGGGQCSSDGESSDATPLNLKGVEGVFWVTIAGTIIAFLLALLEAILQVTKKSDKD
ENFLRRSTQRRDQILFQIRGDGETSFL
>AquaIR25a
MSINEIFLIFQFVLIGYCHGQTIQNINVLFVNEEGNEVAEKALDVAMTYLKKNNKIGVGVDVRRVVGNRTDSNAFLESLCSTYDS
MLEAQTYPHLVLDTTMTGLGSETVKSFTQALALPTISASFGQEGDLRQWRNIDENEKDFLIQICPPADIIPEIVRTLVLNQNITNAAI
LFDESFVMDHKYKSLLQNVATRHIITAIKQGNQVVEQLNQLRKLDLVNFFVLASLKNIKRVLDAADSVNFFNRKFAWHVITQDEG
EMKCVCRNATIIFVKPSPNAAFQDRLGTMQRTYQLNIEPIISSAFYFDLTLRSFIAIKEMVSDGTWKNSVTNYITCDDYDGENSPK
REGLNLKKYFNKEITETPTYGPITVVSNGLSYMEFQMQLTSVGVREGASDKSTILGTWSAGFDNNLTIVDQQVMVNLTADLVYR
VVTVEQKPFIFRDESAPRGFSGYCIDLIDKIADILQFDYEITAVDHFGTMDESGKWNGVVKELMEKRADVGLGSMSVMAERENV
IDFTVPYYDLVGITILMKLPETPTSLFKFLTVLENEVWLCILAAYFFTSFLMWIFDRWSPYSYQNNRDKYKDDEEKREFNLKECLW
FCMTSLTPQGGGEAPKNLSGRLVAATWWLFGFIIIASYTANLAAFLTVSRLDTPIESLDDLSKQYKIQYAPLNGSSTQTYFERMANI
EARFYEIWKDMSLNDSLSEVERAKLAVWDYPVSDKYTKMWQAMKEAGLPNTMDEALEKVRSSKSSSEGFAFLGDATDIKYLE
KTNCDLIAVGEEFSRKPYAIAVQQGSPLKDQFNTAILQLLNRRELERLKEKWWNRNPEKKDCDTADDQSDGISIQNIGGVFIVIFV
GIGLACITLAFEYWWYKYRKVSRIIDVQGAANQPGRPTLKKNQVISKLKTSEEATSKKRERFFPRSR
>AquaIR41a
MFSLYFPTKSELFPSLLTTNASLAIVIDREYLEDEYDTVKTDIEEYLLYAKREILKHGGVNVHFYSWTAMNVRKDLAAIFSIASCSD
TWRLFHSTDGEELIHMAITESDCPRLPTDAALTVPLIARGQELPQILLDLRIAGVYNWKSVVIYDATLDRDMTTRIIKSVTQMSN
NDGVKATGISLIKLKKNISKSNLKKILSAIDSKTVGGNFLVIASYYLVGTIMEYSKSLKLVDTRNQWLYVIPDTDRRYHDMHVFKD
LLKEGDNVAFIYNTTVTSNNCVGGRKCQIEEIIKAFTRALDEAIQDEFETASQIAEEEWEAIRPTKIERRDFLLNRAKKFVSKNGVC
DNCTFWEMETGETWGKEYQSLEKNVTAALVPVGTWRPSDGTTMTDELFLHIAHGFRGKLLPMVTFHNPPWQILKLSESGNVIE
HKGLVFDIIRELAKNLNFTFRLEVVNKTSFSANATSLSSSYNIVGNSLTNRIPATILNMTKNKFVAMGACAVTVTDEFKYIINFSRPI
STQTYTFLVARPRELSRALLFISPFTGDTWLCLAASIVCMGPILYYIHRFSPVN
>AquaIR64a
MQTTDPDAINLYEKKIRGSTNSSGFYSPFEGLDLVRRGGFAFHVETSTAYPIIEATFSNQEICELDEIQMYRTQPMHTNLQKGSPFR
EMMNFCMLKLVENGNMDRLRKHWDARKPTCIESAKKQEIHVSLSEFSCSTSALIIGICISLIFLISEFVTHFRISLVNLLKPPLEIRG
HPDQVYPFVE
>AquaIR75q

MRKHWEYSDEFNNALLELLEQGRLSELKDKWWKQVGGGVCASKPEQSDPKPLTMENLGGIYIVLVVGSGMALIHAIISWLCFIF
RKARSHKVPLKVAFKEELKFVLEFTLYTRDLKAAASIYSPSRKP
>PstrIR3
MKNGRKIATMISKVVLVFWLAGDVSVTRCANREKFNIGGIFSDEIQEAAFKISTSEINRKHPESGIELVPLTVLVPPTDVLETYKTA
CDFLRTGIVGLFGPSSAHSSPYVQAICDAKEIPHVETRADFAAERNESLVNVHPHPGVLARLFKDLVRAFEWRRLVVLYEGRRSL
LELGSLLEFNNRKGRKVVLRKLESVDKGNYRAFLSEVKSLGETNFVLACSVDVLEEVLKQLQQVGMMTESYSYIITDLNAQTL
DLTAFQYAGTNITAVRMLDPEQIEIMEMAESLQKQNAPGSDLPVLLESWKTKLETVLLFDAVELFHETLRNLTVDGKKKIRSRRID
CDGPTAWNDGYSIINLMKSVDAHPGVNGRRRVRRERLPYELRGGRAGALLGRVDQDRRMEFNGEEAPLPKAPEAIQTRRSSGRF
QHHPHRTHKH
>PstrIR5
MAGNMSIRLCVVLLLCSKIGYSGLPPVIRIGAIFTEDQKNSSVELAFKYAVYKINKDRDLLPNTTLVYDIQYVPRDDSFRTSKKVC
RQVEFGVQAIFGPSDPVLGAHIQSICEALDVPHVEARVDFEPISKELSVNLHPSQAHMNRAFKDLMAFLNWTKVAIIYEEDYGLF
KLQDLVKSPGTARTEMYIRQAGPGSYRQVLREVRQKELYKLIVDTNPRNINQFFRAILQLQMNDYRYHYMFTTFDLETFDLEDF
KYNSVNITAFRIVDVEHPKVKEALDVMEKFQPIGHAILNRSGIIQSEPALMFDSVYVFAKGLSGMESGQSIRPANLSCDIEKPWDD
GLTLYDYIDSVKGLHGLTGNLEFSDGKRTNFKVDLLKLKKEEIRKVGEWTATDGINITDPNAFYENHTPNVTLIVMTREERPYVM
VNEEKNLTGNARFEGFCIDLLKWIAGQVGFQYTIRLVPDHMYGVFDPETKQWNGIVKELMEKRADLAVASMTINYARESVIDFT
KPFMNLGIGILFKVPTSQPTRLFSFMNPLAVEIWLYVLAAYVLVSFTLFVMARFSPYEWNNPHPCHRDSDIVENQFSVSNSFWFIT
GTFLRQGSGLNPKV
>PstrIR7
MASGIWWYFCLIMSASYTANLAAFLATENPIKLFTDLQSLYDSQDITYGAKVNGATFNYFTTAKEGLLQKVGKVLLDHPEYNVL
DNDIGVQRAEDGKYAFFMESSSIEYVTQRHCNLMQYGDHLDEKGYGIAMRKYSPYRKKLSLALLKLQQNHFLDDLKKKWWEE
RRGGGACEGVVESSEADPLELVNVEGCFYMTVFGTILAFGLVLIEHFLYLLRVRKRSGIPFWKIIKAELKAYINFNSPSKPNLEVLK
EIPDEAETEDDKKSEDDKEDSEENRRKSKSASQTRTFTRSRSKSVSRQSSHSRKKRSKSSGKGTPLSYGFIIPSSLENLKDIEETF
>PstrIR9
MKLKQILDVGFLLNALIVSVVSAGPAWKEIQRGGGLKIGSGGGKNTSLLRGNGGTSSGIKLGGSTRGIRTTTTILPEEDHIFAPTTP
SVKTGMNHLHIGIMVPYKSFGVREYKKAASSATAYVQRKLKFFKNHDIQTHLEMQEMTPSPTAILKSLCKEFLSYNVSAILYLMN
YEQYGRSTASAQYFLQLAGYLGIPVIAWNADNSGLERRASQQSSLMLQLAPSLEHQTAAMLSILERYKWHQFSVVTSLIAGHDD
FIQAVRERVSAMQDRFKFTILNAVLVANRGDLAALVDSEARVMLLYCTREEAIDILTAARDLHLTGENYVWVVTQSVVENPQQTP
YQFPVGMLGVHFDTSSSSLVNEIMTAVKVYAYGVEDFTSDPDNDGRSLNTQLSCEGAGAARWDTGDRFFRYLRNVSVEGDQGK
PNLEFTQDGVLRAAELKIMNLRPGVSKQLVWEEIGVWKSWQKEGLDIKDIVWPGNSHTPPQGVPEKFHLKITFLEEPPYINLAPP
DPVTGKCSMDRGVLCRVATDADITEVDMSQAHRNGSYYQCCSGFCIDLLQKFSEELGFTYELVRVEDGRWGTNDNGKWNGLIA
DLVNRKTDMVLTSLMINAEREAVVRN
>PstrIR10
LFADAPGLFENMRKILLVCLVLFFLTLVGGIKKKTINIGIFLNDDDHYETSTIALTSVVRRINLYTKVEYLLRPHIFRTQKHEIWKTG
QTACNLLRKGVAAIFGPEHPEINSMIQSLSTNLEIPQFQTFWNPLQAPFVTNQQPEKPLQIFNLHPSPWSLSKALATLIRENDWKSY
AILYESDEALLRLEQPLNQLHPNDPAVAFKALGSPENYRVVLKEVKNSGVLHFVLDCEVERIMDVLKIAKEQNLMTEFHSYILTN
LDAHTLDWSELKNIRSNITALRMIDPADPHVKNAALIWNQNMKYDILDTVKNAAIQKELNRTRPKRRTYNDILPSKLLTKTILLY
DALNLFISIFSELDSKLNVTLRPLSCESNLTSEHGRLFSDLLILKQNHHLLLQPLTGPITRFDTIGYRRAYRLQIVELEEAKFRVTGT
WDSSAPDKLNLTLTQEERVTELKKKIQKRNFRVVSRLGDPYLMRLPDPPGKPLLGNDRFEG
>PstrIR11
MRACMVILFLSTGLALDPKFVFLKDFFRKKNVPITLVTCPDFKNVTIEAFLSSRTHLDNSMGFQFYGNRPQIIFNSANHRNWHVID
LIFCGKSSDFLFHMNEINAFKIPFKHLILIDISSFDTILLSVCKCDVSIGSEVLLAIFNKTTAEIFETYKFRTTSKCNVLPYGNWSEKN
GITYTTQGASLFQRRRNFQREELVVNAVIKSNKSLQVDFDDIPEATFDEPYFLESAASLTYALTYLNASYSFRVYTVFGHLNHTTG
KFYGMLSDLAKGIGDITGSALYLSLERLVVVDLVGNQRSYGTKFVLKEPSMSYVENIYLMTFTDGVWLASGLVLVIFCFILFVVIN

LEGFKKKNDSGKGRKFTISDAVLLSLEALCQQGTSVDSKTIAGRILLVFLFMVFMFLYAAYAGYILVLLQSTKPIGSVKHLLDSRLE
CGGVNVSFLTDWYLVNNDPVLKELYLKKLKNVGLLSLEKGLSRVREGNFAFHTALNIAYIHIHKTFTDDEICKLQELPGYLNTDL
YYVVPKRSQHKEFFKVSILRINELGFQSRNQYRSSKKPKCYNSIGNFVPVGFYDCYTIIELFFIGTFLSLIIFIVELGTDKWMRSLKM
KRFN
>PstrIR15
MGFISFKIKLLLILFYIFDKTSGLSVGVLLGDYPSQEQRLLNSTISKKFAKGELIFSANVQHVSEFDSFGASETLCEVLSSNSGVMAV
FGPKYTPATTILESICLEFEIPYISYSWRPRAERDRGFFMNFFPETDLYANALGEIVRSFGWKSFAVIYEKDETLLKLKNILNFHQYN
EKDKRNKIAFEKLGPGPDHSDLFRKIERSMQTNIILDCKTELIIPLLQQASEANLLNVYNNYFLLDLDALTLDYSDLNTTANITTIR
LFDDNDESFKESARRIGLAELIHHRKLKTDIALFYDALWYFHDTMKTTQMLFTDPIYCNGTKKFKKGLELSLAMKNRFFFTNLT
NLTGPILFDVQGSRIDFNIIIVDVLADKKIATWFEQNQTLITNAEFDESITVVNNLQSTTVIVSSKLGAPYLMLAEPEDGKVLEGNE
RYVGYSMDLIAEIAKIVGFKFEFRLAEDGSYGRYEVFEDKWTGIMGDLTENRAHLGICDLTITEEREDAVDFSLPFMELGISILFTT
TDYFESNSFLFLIGFEQSLWMTIVYCYIAISVCLYIVLRLSPDDWERKYPCDELDETLVNRWNLKNSLMLTLKALTMQGSDAVPK
GKSARLAISMWWLFSLILTSYYIANIALIANLTSQESAINNVEDLAAQSQIKYGMVMGGSTQEFFKNSNTTVYQKMWNSIEHLPS
VFTKTTQEGIEKVLESNGQYVFFMESTTLDYALERNCKLKQIGGLLDLKHYGIAMPQNAPYRSTINRAILKLQEDGVLAELKKK
WWSVDEEETCDRTVDSSGQLLPENIAGLFIILALGIITALSAALFEFLWNVKRVAKLEGITFCEALGDESKMVCSIWTNRRKLQ
>PstrIR16
MLKFIVFIYTCCDVLSSISANQKDWNSQNPTVFNIGGVLSSNESEKYFQETIAHLNFDSQYVPKGVTYYHTAILMDPNPIRTALNV
CKYLISRKVYAVVVSHPITGDLSPAAVSYTSGFYHIPVIGISSRDSAFSDKNIHVSFLRTVPPYSHQADVWVEMLKHFKYKKVIFIH
SSDTDGRALLGRFQTTSQSLEDDVDIKVQVESVIEFELGLESFKDQLLELKNAQSRVYLLYASKQDAKVIFRDAAVFNMTDAGFA
WIVTEQALEADNVPEGILGLKLVNATNEKAHIRDSIYVLASALRDMNQTKEITEAPEDCDNSGSIWETGRDLFNFIRKQVLLNGE
TGKVAFDDQGDRINSEYNIVNIQRKKKKVVVGKYFFNKELDRMNLKVEENSILWPGKQKEKPEGIEIPTHLKVLTIEEKPFVYYVR
KLMDSQDTCTPDEIECPHYNVSDETSASIYCCKGYCMDLLKELSKKINFTYSLALSPDGQFGNYLIRNSSVAGKKEWTGLIGELV
GDRAEMIVAPLTINPERAEFIEFSKPFKYQGITILEKKPSRSSTLVSFLQPFSNTLWILVMVSVHVVALVLYLLDRFSPFGRFKLANT
DGTEEDALNLSSAIWFAWGVLLNSGIGEGTPRSFSARVLGMVWAGFAMIIVASYTANLAAFLVLERPKTKLTGINDARLRNTMEN
LTCATVKGSAVDMYFRRQVELSNMYRTMEANNYDTAEAAIKDVKEGKLMAFIWDSSRLEFEAAQDCELVTAGELFGRSGYGIG
LQKGSPWSDDVTLAILDFHESGFMESLDNKWILQGNYQQCEQFEKTPNTLGLKNMAGVFILVGAGIVGGIGLIVIEMAYKKHQI
KKQKRMELARHAADKWRGAVEDAKEDKRKSMRASANPQRRIKSNGVNEAVTISGVFDKFQRIGQFGHERTWPGDADIRQRRT
DDVGGGGVQPVPRYLPSYTQDVSHLIV
>PstrIR18
MWWFFALIITNSYIANLTAFLTKANLEPPIKDAEDLSKQNKIKYGCMEGGSTMQFFRDSNISTYQKMYLNMKMQSPKVFEKSNE
DGVSRVRSDKKGWYAFLMESTLIEYYVETNCDLKQIGGWLDTKSYGIAMPMNAPYRGAIDKAILELQETGVLNELKLKWWKK
NRNETSCDIIRNKDKDENENDLDLARTMGIFLVLAVGVSIAIFLGVVEFLWNIRNISVEEHLTYWEALKIELKFAVNIWITRKKVKS
AASEASSSSSGKEDNANKGDKKKMIQNFLHNASSFMNLNTTA
>PstrIR19
MKEKYQDTITEWYNDFVDSIESLQFETIELNIDTFNKTKACDTLSQGAQIVLDITWGKQEEAEQLFSSIGIPYVKIDVAITPYLDLL
DSYLDLRNATDITLIFEDPWYVDQTLHYWMNIPKMRMIMTDTLTAGSLKKLRDIRPIPNNFALFATTENMNRLFKLALRENLVILP
ERWNLVFLDFHSNALDRNLIAKGSVNILTLNPELCCYFTSSNDDCNCPRDFDLRKHFLQKSLTVIADAISGLLKEGTSIETPLCDGK
ISNEGLQKSFDDRLFSTFANDDFMYYNYSKIRLRSFGSIGIGNNGSVELIVSYDDGHATLVENKTIQPIKAFYSIGVSHALPWSYKA
KDPETGEMKWTGYCVDFAEKIAEVMNFNFEIVEPKEGTFGEKVNGVWNGVVGDLVSGRTDLAITALIMTADKEEVIDFVAPYFE
QTGITIVMRKPVRKTSLFKFMTVLKLEVWFSIVAALIVTGFMIWFLDKYSPYSARNNKKAYPYPCREFTLKESFWFALTSFTPQGG
GEAPKSLSGRTLVAAYWLFVVLMLATFTANLAAFLTVERMQVSSPSQTPQKKPTNLSTAGSSPILGAASEAIENQLHCSEELPNAP
VLHQHEVRRGYSLQNVERTDSKRLHRRQAVPRVGLPHKGAVRPHSAGHQRLEPGGYGRRGLQERRRAPGRRLRLHTRLQRDQ
VRDQHELQPDGGRGGVRREALRRGRAARQPHAGRAEQSDSEAAEGPVLRRVAGEVLESFGQRGLPEYG
>PstrIR22

MIFPAKIWYFFIFQCALCAVDNVTYLKIAGIFENIQLHQEAFKYSQRISSLKSTPNNVIVSPVIDPYVLKDEPFTAYKATCSFLRQSV
VGIFGPQSALNFDIVQAITDRKDIPHILTRWIRPSEMRLQTFNFFPNPDRLADGFVDIINALEWETFTVLYTNEEHLIRSNEFIRKAK
DDGIIVYIENIDPFGDGNYRPVLRNAGRSGQKNFVLDCPIQDLRMLLTQLQQVGLLTAEYNYFLTNMDAHTEDLSQFQYSDAVIT
GVHLIKAKDELALRASQDLCMLYNITFKLDCGKPELDIETALIIDSVNVFLQTLNSLGIVEGQYLDCDGEGVWTDGLSVINALRT
GSFEGITGQIEFDNHGFRRTFQLTIYQIRDNTTIRGSWNSTDGLSEDIELTYSDEKEEEDSDLKNKELTVLITLTEPYARLSPTDGEVL
KGNGRFEGFAIDLIEEIANMEGFDYTLVVRSDHNHGNFDRKTGKWDGMIGDIIDGRADLAIADLTINKERVDPIEFTLPFMSVGISI
LFHKPTVIPPPFFHFAQPFSIRFWEYLAGSYLITVLSLFLIGRLSPSEWQRPHTCKEDKKYLVNELTLLNSFWFAAAGLFRQPTNVKI
NSVAAKVIAGAWYIYCFVLFAMYISYSFANNHVEEKEEMFGNVEEFLRYAEENSIGFGAMKNGATEGFFKNSKSEVYQEVAKYM
EENPNDMMATTTDGIQRVLEGNYAFFMESATIAYTVRRHCNLTSYGGLLDSKGFGIAVKKGSNLLGPLNRAIIKLQSSGELQRLK
NVWWNEKYAGDPCDAEDDSVPLDKTVPHVNGLIAITFLGIAIAFVSSVLEFTVYAIRLSRKVKAPFGETFGEELKKCFGKSHTVQ
HVEEIALTKPENGDSAAKENA
>PstrIR23
MHLLIISSFLQVITCLPDLIPIGGLFHPADDKQEIAFRYAIEKINNDRSILTRSKLSAQIEKIPPQDSFHASKKVCHLLRTGIAAIFGPQS
AHTASHVQSICDTMEIPHLETRWDYRLRRESCLVNLYPHPTTLSKAYVDLVKAWGWKSFTIIYENNEGLVRLQELLKAHGPYEFPI
TVRQLGESSDYRPLLKQIKNSAESHIVLDCSTERIYDVLKQAQQIGMMSDYHSYLITSLDLHGVNLEEFKYGGTNITAFRLVDPEG
QDVRRIVRDWNTEIEIKNRKGEPASMYKQDVQDNATFVKAETALMYDAVHLFAKALHDLDTSQQIDIKPLSCDAVDFWPHGYS
LINYMKVVEMKGLTGVIKFDHQGFRTDFVLDIIELSREGLKKIGVWNSTEGVNFTRTYGEAYTQIVEIIQNKTFVVTTLLSAPYVM
LKETSEKLSGNAQFEGYAVDLIHEISRVLGFNYSIKLVPDGRHGSLNRATKEWDGMIRELLDQKADLAIADLTITYDREQAVDFTM
PFMNLGISILYRKPIKQSPNLFSFLSPLSLDVWIYMATAYLGVSVLLFILARFSPYEWDDPHSCRGAPPAVLENQFTLLNSLWFTVG
TLMQQGSDIAPK
>PstrIR24
MVSKRIGALLLVLALCDPVKNETFPSLLTTNASIAVVVDREFLVEDYEDARNEIEEYLVYAKREILKHGGVNVYPYSWTSINVRK
DLTAIFSITSCYDTWKLFRLTQKEELVHLAITESDCPRLPSDTAITIPLVDKGQELPQMLLDLRSEGIYNWKTLVIIYDATLDEDMVT
RVIKAVTQRKYGDVKATGITLMKLEPNLSRTELRDFLSTINPKVLGNNYMCIVSYHLAGNIMEYAKSLQLTSTINQWMYVISDTN
DNFKDIEAFESQLKEGDNIAFLYNSTFKSSNCKEGRKCHIEELLQAFARSLDQAIQDEYETANQVSEEEWEAIRPTKPERRDFLLK
NMNHYLFKNGACDNCTNWAMKTGETWGVEYDASDQRAEPKVVPVGSWRPGDGPVMTDELFLHTAHGFRGKLLPMITFHNPP
WQILKFNSSGEVIESSGLILDIVKELSRNLNFTFRLETLNKTSFNSGTSNSTSFDVDSDFTNQVPKALIDMIKNKTVALGACAVTVT
EDLEKVVNFTVPITVLSYTFLVARPKELSRALLFISPFTMDTWLCLAAAIVSMGPLLYFIHRHSPVYEYKGYPMKGGLASIQNCIW
YMYGALLQQGGMHLPYADSARILVGGWWLVVLVMSTTYCGNLVAFLTFPKVDIPITTLDELIAHKDTVSWSFREGSFLEKELAIS
NEPRYKTILERRLKHTSTDNEGLIRSMVEGKHVYIDWKQKLTFIMKRQFLKTDRCDFVLGLEEFFAEKLALVISPDSPYLPKINDE
>PstrIR25
MSSRWERLGAAIGLVAGVLAVVRAAASSLPPAVKIGAIFTEQEKNGPNELAFKYAIHKINKDKTLLPYTSLVYDIQYVPRDDSFHA
SKKACQLVQYGVHAIFGPSDPLLGAHIHSICDALDIPHIEARLDVDADVREFSINLHPTQYLLNNAFQDVMAFLNWTRIAVIYEKD
YGLLKLRELVGSSQNGGLEIHLRRASPDSYEDVLMEIKNKEIRNIVIDTKPVNLRHFLKGILQLQMNDYKYHYLFTTFDLEAIDLE
DFKYNFVNITAFRIVDNEDVAVKETIRNMLKFQPNRELRASNGTYIQAEPALIYDSVYVFAVGLQTLEQSHTLKLSNLSCDKEQPW
DGGLSLINYINSVEIKGLSGPIEFKEGRRIQFKLDLLKLKQPSLVKVGEWQPGNGVNITDRAAFFDPGTMNVTLVVTTILEMPYVM
MHTAKNFTGNSRFYGFCIDILDRISQEVGFDYLLDLVPDRKYGARDPNTGLWNGMVLQLMQHKADLAVGSMTINYARESVIDFT
KPFMNLGISILFKVPSSRQAKLFSFMNPLATNIWLYVLSAYVLVSITMFVVARFSPCEWQNPHPCE
>PstrIR26
MFWYVFGTFTNCFTFSGKGSWSRANKVTTKLLIGFYWIFTIIITACYTGSIIAFVTLPVFPSVVDSAEQLLSGWYQIGTLDKGEWQ
YLFQNSSDDITKKLLKNVDLVPTVEEGLKNTTKMSFWRYAFLGSKAQLDYIVRTNMTTRGKRSVLHISKECFVPFSVALAYPSNS
VYAEILNSGIERIKEAGIMMKFRTDVEWEMMRSATGKLLAATTGVSLKTLAYEDRALSLEDTQGMFLLLAIGFVFGGGALVVEW
FGGCYKICRRNKRRGSDESIESNPRMHERQLPQSKWKLKERYEHLKRSFEEKLENSSAKTEEYYNDDKNSEQIDNIIDKIFEDVL
KNEEENDKKISSKETKNYDTHEIDY

>PstrIR27
MYGNFDPVTKKWNGIIKELLEYRADLGICDLTITYERRKAVDFTNPFMYLGISILFSKAVKEPPDLLTFSHPLSFEVWIYIATSYLIV
SLIMFIVARLNPNDWENPHPCNPYPSERENIWTFRNCCWLTMGSFMTQGCDLLPKGISTRLVVAMWWFFALIMTACYTANMTAF
LTSSRMGSTIESAEDLAAQTKIKYGCVDGGATSSFFKDTNFSTYHRMWVQMESADPSVFEKSNKDGVKRVVSSKRKYAFVMESS
NIEYEMERNCDLVQVGNLLDSKGYGIAVPFNAPYRKAINEVLLKMQEMGLLQTLKDRWWKEKHGGGKCTKDKQSDDAAATE
MGFDNVGGVFVVLGIGVVFSLMMAVCEFLWNVRKVAVRQKITPKAALIQELKFAVNIWARQKTVNAAISMEKFD
>PstrIR29
MDTDELCNIEFIDLNGVDESEQLRIRNSLQQLDPSVQLPWVKNKKILLRDKQQNNRENDGQNVIESVPTSQHHNPYINHPMITQP
VYYNQQQSVVVQPIITYPHHTNILYYPTNVLSPVSPPYPQQVFIPQTNYSTTYPCTIQTGEAEDLQKEENEGILSKENGIEDNGDDK
NETTPNKVPEIIPSSPIIENGNENVETVTSPAPVNKSWASLFSSSKSSNTSSTKPHPELNIQQTANKKSYENEAFCPIKHPRKSQQFID
PDCYRMGEFLISYAIDGKALSLQPRGLLNQSNYCYINSILQALVACPPLYNLLTGLAQNISSNGKRKPTPVIDGMCRFVKEFKYLPP
NLRNKDKKAEKNPKKDANVLINTDIPFEPTWIYKMLNGIRTDLIEGRQEDAEEFLGFLLNGLNDEMLELIKLVKNDKEEPNESVL
TTDDNGEKEWKVMGPKNKGSITRRTDFDRTPISDIFGGLLKSKIHRAGDLSTENIQPFLTLQLNIEKVKTVREALEALVNKHQLEG
LTSSKTNEEVEAWQQVLLDELPVILILHLKCFDYKQAGCTKIIKALEFPVDLKIDQKLLSSKPQSQKEKHYKLFAVVYHDGKEAS
KGHYVTDAFHIGYSCWLRYDDASVKTVQEEQVLKPQGTRVPYLLFYRRSDTIRGK
>PstrIR30
LVYDNFADGDQFVLMRNVCSIIDEGVVAIFGPRSYRNIDVVQSICDVKEIPHIITRWKSYYIDGDKTIINFYPDSSTLSRAYYDIIAN
MGWKTFTVLYEGDESLLRIKSLVETAHQDGVVTQMMQLDPFYTGNFRDTMRDLKKTQQKSVVIDCHIDSLFQVLTQLQQAGL
MNEQYSYFITNLDTHTENLMPFKYSNANITGIRLVNPDSEYVQKVSSDLLSENLNFLESPAWKIRLEQALTIDAVNMLAAVVIDRQ
KSSDVRIITNKNSLLCAEPDSWEHGLSIVNLLKSYPHDGLTGRVIFDNQGYRSDLILTIFELLEGGITDVGVWSSSYGLNASRPGDV
SGIDDPESMRNKSFVVEIALTEPYGMLKQTTDHLYGNDMYEGYAIDLIEKLAEMEGFNYTFVVREDKSSGSFDKKTQKWGGMIG
DLLDKKADLAICDFTITSDREGAVDFTVPFMSLGISILFREPESAPPSFFSFAD
>PstrIR31
MLSREYKLFIENRSSSHGLLENGTGIYSRLYNLMLQRQGYNNSIVNSVEEGVELVKSSDNVALMAGRETLNFDIQRFGVSNFHLS
EKLNTAYSAIALQLGCPFIEEINKILMAIFEAGIITKMTEKEFEKLGKEKALSSAEIAETVAKDTSKDLQRQIKLKEPRKLKPISLKM
LQGAFYLICFGNVFSGLILMGEIAYKRHQVRQRERNRRLAIGKWKQTVKVNLRKVQNMVLGLHTNPAHDELITPFDYLE
>PstrIR32
MFPWNSTELIIQDVYNPASTRGGRLKKRIMGYYNRTDGYNIVEKEFKYTLRRDMSGVTLRSIIVLPIDFNETLEDYLNNDSDPHIN
TFNRFHYSLLRPCVAYYNFTSNVTMEKSWGYLINDTSFDGLVGALERKLVDYGSSPLFVRADRGTVMEYGRRTWTLKAAFIFRN
PKSLGSADIFLKPLSTSIWVTLICTSGFMVLILRYTQILEKTRIDRTSDTTWSYLLIGATSVFCQQGLGINTPSVYSGRITVLVLLILSY
LVFQFYSASVVSNLLIKPKALIQSIESLLKSPMQAGCEDILYNRDYFKYTTDQISKQLYEKKILKGDNNSNFLVPEKGLEKVRDGG
YAFHVELATAYPIIKDTFPETYVCELNELQMYRTQPMHANFQKHSPYRDMFDTCIQRLAEYGIIYREIKFWHPKKPECVHSKAKL
NYHIGIDNFYPVLCLLAIGMIISLLVLSVEMYLNFKAVKNTVVFPFTN
>PstrIR33
MRLRWILIFLTETINVTAFSVLFSIIQEYGKDHMISEFSCNGKESFNLLKLASQTYQPIRILKIDEKLLQNNVPANTIFVMNLDCNGS
RAILRKSDDLNLLSLPYKWVLFHSKPLGSYEKYFYDLNFSINSEINALQKYKNGSTILRKIYELKGDLQIENMGIWENNIFRSFDIE
RITVRRRKNLKNITLNTCIVITNNDSLNHLTDKRDPHIDSISKVNYVLIGHFKDILNVTLNFTIESTWGYKVNSSWTGMIGLLSRKK
IDIGGTSLFFTEDRVDVIDYVAMITPTRSYFIFREPKLSYVTNVFILPFDMGVWLSTVALVIIAVFFLYVILNWELTKEIYLKKSNSYY
ASNNKPTVLDVVLISFGALCQQASHIVPASVPGRITTIVLFLSLMFLYTSYSSNIVALLQSSSSSIKNLQDLLNSRLEVGVDDTVFNR
FYFPNATEPTRRALYLKKVAPPGKSDHFMPILDGVKRIREGLFAFHVESGPGYKLIGEIFHEDEKCGLHTIQFLQVIDPWLAIQKRS
PYKKLIQIGFRKLMESGIQIRENTLIYHKKPVCVSRGSAFVSVGIVDCYPAVVVLVIGIGTSLFVWAIELLNKRFLVTDYIKTMLKN
YLKIKESKHEDIVFDLKE
>PstrIR34
MGLDDTNRTTLVPVLTSNVESCLENRLIARISARLPFITFAMFNMEDVGYSKTRQTRYNAKFYIFFLDDYRNFTDALAMVRRADS

FNPTAFFLVYVEGNFGGNAITAGGILQVAKINYLPYAAVLVNLDDAFELYQFVYDYGKKKKRKICGTYPKVTVVDTCVNGTMLK
PSKNYNDVRVRENCIVKVVAKEFVPFVLSKTEGFEIDVLQLIGSYLKVTFDVEFYPKSALPNWGGKINGTWTGLVKDVAEKTSV
GVGNIVVGLMDSKEFSYSQSYHYASLVWVIPKALDIPKWRILFALFSPQVWALCLGMVLLFAFVFYLVIKLQGGTPRNSLLTATQI
LIYQPVSHLPRNDLHNVFFLGLCVASIILNSVYTSSLLFYMKNPMTEHQASGLHELIDPFGNPVYEVGGYSKYKSFYNDSETETSR
FVYDHYEVSYGENDTLEYWLRRVAHTRRYWTISTLLYAKYVDALGKNITRTEDGKSMIFVRSKKPVSVNGVGLIMRRSHPLLGK
INKIIQHMLYGGIQLKIRSKYWRVIEKMESLDNSIDMADEPLTFYHLEGAFAFLILGYVLGFLILAMEILIYRCKKRKNRKMKDIRH
LKRKKNNKVHEIR
>PstrIR35
MAFLLPFSPELWIAIFTSLNITAIAVAIYEWLSPFGLNPWGRQRSKNFSMSSALWVMWGLLCGHLVAFKAPKSWPNKFLINVWGG
FSVIFVASYTANIAALIAGLFFHNTVSNYHDRSLLSQKVGAPRSSVAEYYVQKANQLLWQHMHKYSLNSIEEGVLKLRNGSLDILI
ADTPILDYYRATDHGCKLQKIGDTINEDAYAIGMTKGFPLKDSISAVIAKYSNNGYMDILQEKWYGGLPCFKLATEIAQPRPLGVA
AVTGVFILLGVGMALGLLILLVEHLFFRYTLPILRDKPKGSIWRSRNIMFFSQKLYRFINCVELVSPHHAARELVHTIRQGQITSLFQ
KSIKRKEHEQRRRRKSKAQFFEMIQEIRSTIRRQQQEEREPPLESVTEVDAENQQSPEEKKSKLSPSIIRRTFLRSSPKADNGNKSKS
PTTYFNKQLFSPRSKSRNKSTTNLNVRRFSTDSVFNSTSPGKEDYCTVVGRRLSKDASAFLSSSPPDINSRRSSYLDIISSGSKLTGK
SPILSIENLSDCSKYSEPMRKLSDSESIGKKLATLPRYQQDSFDKHKLLQPQYSLTLGRSDEALNSVSKSDTVGQTRSFNNITRPDL
NEKSNLSDDEIARRNRETIIQLQSTAIRNPKPPLKRTKDNNSKPSSSLTNPQIRIQVEDTSEHDTRHPPLSKKPKTRHESLGDAAGPS
ETSTSKRNRLTDQSRSLDSANPNSSDRRRRRSGSRSKSREEDLPPAPPPPNCSPRNSDGRSPLERLSKDELVLLWRSSESELRSHLL
KALRDKEDAAEPP
>PstrIR36
LSGRRGRLRRDARSLFVCETLDRSLRRESGQAMGSANFGLGVVFVTFVSGILASAEKIPLGAIFEQGTDDVQTAFKFAMLQHNQ
NVSSRKFEFQAYVDVINTADAFKLSRLICTQFSRGVYSMLGAVSPDSFDTLHSYSNTFQMPFVTPWFPEKVLAPSSGFMDYAVSM
RPEYHKAIIDTVKYYGWRQIIYLYDSNDGLLRLQQIYQSLTPGGEMFQVQTVRRITNVSEALVFLRGIEEQSRWSNKYVVLDCST
ETAKEIVVGHVRDISLGRRNYHYLLSGLVMDERWESEVVEYGAINITGFRIVDSYKPYVKDFLEGWKRLDPAQSPGAGKDSVSA
QAALMYDAVFVLVEAFNKVLRKKPDLFRTNVRRGIAFNGTTKSLDCNTNGNWVTPWEHGDKISRFLRKIEIEGLTGEIRFSDEGR
RQNYTLHVVEMTVNSAMVKVAEWSDIAGFNPVAAKYVRLKANAHIERNKTYIVTTIVEEPYIMLRKPEPGENLSGNDRFEGYC
KDLANLIAQRLGINYEIRIVKDGKYGTENHEVKGGWDGMVGELVRQEADMAIAPITITSERERVIDFSKPFMSLGISIMIKKPMKQ
KPGVFSFLNPLSKEIWVSILFAYIGVSIVLFVVSRFSPYEWRLLHLSADSRDPNNGQTAMSNDFTMVNNNGQTAMSNDFTMVNLS
PRSISGRIVGACWWFFTLILISSYTANLAAFLTVERMVTPINSPEDLASQTEVEYGTLFHGATWDFFRRSQLSPYSKMWEFMNSRK
HVFVRTYDEGIKRVRSSKGKFALLIESPKNDYTNEREPCDTMKVGRNFDAKGFGIATPLGSPL
>PstrIR37
MRLNFYSFFFIFAIISSVVCSKTRYVHGMHRYYTKHKLSNFNEETFALASADAEIQHQHHLRHQHEEVPWPVKKEAVVEGDLVLG
GLMMVHEREESITCGPVMPQGGVQALETMLYTLDRVNKDEEAVLPGISLGAHILDDCDKDTYGLEMAVDFIKGSISNIDGAEYH
CNKTQVRKVISGVVGAASSVTSIQVANLLRLFKIPQVSFFSTSPELSNKQRFEYFSRTIPSDFYQVKAMVDIVRKLGWSYVSIIYEE
SNYGIKAFEELEDTLSKYSICIAIKEKLVKDSGVAKESVYDSIIQKLMTKPRARGCIVFGSDQEVAELMKAVRRCNATGYFSWIGS
DGWSARSLVSEGNEAEVEGTLSVQPQAGEVAGFKEYFLGLNVENNTRNPWFIEFWERHFNCRYPNSSKTPYNMKIVNVCNGTE
KLTANNTVFEDQLQFVSDAVLAFAYAIRDMHRDHCHGTTGLCDAMKPTNGTELLRYLRKVDFIGLSGDRFHFDENGDAPARYNI
KHFKQVAYGKFAWITVGEYLEGVLKLNMSAIQFKLDQPTPPESVCSLPCLKGQAMKYVEGESCCWHCFNCTQYQIRHKDDPTQ
CVKCPKGTIPDPEHEHCEDIPEEYLRPVSGWAIGAMAFSSTGVLVTAFVFSVFMKHNETPVVRASGRELSYVLLSGILMCYSVTY
TLVLKPTDVVCAIQRFSAGFCFTVVYAALLTKTNRISRIFNASKHSAKRPNFISPRSQLVICFGLVGVQVLINGIWMLISPPKAIHHY
PTREDNLLVCSSYVDASYM
>PstrIR38
LNIPRGFGVMISKQLFERSSGELYFIIEENYEVIRKKLLDKSKRRLNTWSGESENVKEKPISEFFMTINLDDSLEELNKKLMKEKEQ
VLIEYGKDHFERELYSYLYIDPLCPAPYNDSDSEDKKIPAQRIFAYPVQTAATFAENGDERFESWFDEQKKRNRFISKVFSILFIQLL
FTASFIFLAITNGAVKEFIQEQFALIVASLAIETTVYFTLVCVESSRRKTPINYILLALFTAASTYISAYFSVYFGTDMVFLAFGSIA

>PstrIR40
MIAFTYPIFLIVVCTVYAVLTRKIPEAFNESKHIGFTMYTTCVIWLAFVPLYFGTANHVALRITSMSVTISLSASVTVACLFSPKLYIIL
IRPERNIRQSMMPMRYSAINKSSTNTGSGSMMAAVMVTAATCDQKQNVQKHVSPIENVVDDVFYKEPRTKDSSTQTTPSSTDKL
LPFEDPLNRSGSNCNGPNAVDSDESKRNSKGFL
>PstrIR42
MHSLLNRKLFSFPQKMKLELLTVFLLIELTTMYSIHDDLTETTKDTTFGLLLQEIIVKSLSPYKCIMIFTDEINSDVFQRKWFKRFGT
AVSYVLVKVKDYDDLYAPSPEIQLSLHVANNGDCQLYIFLISNGIQMRRLLKFGDRYRAISTFTKFILLHDNRLFEKRFLYLWKRII
NVIFIKKFESKVIKITNSTKENAWFEITTVPFPLPLTNILIPKKVDIWAQSKFRKGADLFRDKTKDLQNQTLKVAIFRHLPGVEKVN
NSESKNMRALMSTTHNETSYFAGTEIEILQTISKSMNFKCEIYEPEGAEEELWGRKLVGGIYTGVIGEIVSTKADIAVGDFYYTSYL
LELMDLTVPYNTECLTFVTPEALTDNSWKTLILPFSSIMWGGLIISLLICMVTFHYLATYYVKISKTKSPDSQTNSISKFKRVLTLSL
QPDFEKIDPNTKYVLMKEKYQVIKKEGQPEGLYQFSEPVNNALYTYSMLLLVSLPKLPTGWSLRMLTGWYWLYCLLVVTAYRA
SLTAILARPVPKVTIDTVQELVDSKLTFGGWGEINLDFFKTSKDPAVKQIGENFVIVNDSNGAVDRVAEASFAFYENTYFLKEAIVK
RQQRFRSFFVNSNATNATSEERKAMLQDVQNDRPLHIMEDCIIKIPVSIALQKNSPLKARMDKYIRRVLEAGFIKKWLGDVMQK
VLIAEIQIEDTESTKALMNMKKFSGALVALLIGYVISILTLISEVLYFHFRVKKNPHFNTYSKQIVIKKAK
>PstrIR43
MGYAEIFFAGLCLNATCDVDLDNVTTSVPSYFQLKFAELEEELKTETLVLTTIQNGELSGYQKIGDDLVGNGIAFDIIQILQNKYKF
NYTIVLPEDETFLGASNQRTAKKLLEKEEVDMAVAFLPIIESIRNEIVYSTSFDVAEWVVLMNRPKESATGSGLLAPFTTEVWIMIIF
SILTVGPLIFLMVLIRARLCKNDDSDVYSLSTCLWFVYGALLKQGSTVNPRTDTSRLLFSTWWLFILILTAFYTANLTAFLTLSKFTL
PITAPEDIGKKHYRWVTNKGNPVRDQILADKNDEHIYQEKLIEKLGNLKVFANYPDMEILDSYVYKRDMMFIREKTVINHIMYK
DYRQKANGGMEESKRCTYVIAKFPIVKFSRAFAYSKNFKYKELFNYAIQRLIEGGIIEFKLKENLPDAEICPLDLGSTERKLRNTDL
LLTYIIVGGGLAVALCVFLMEVLWRVCQRRYRKRKAGRMRANGWPNNHKSRAFRSNLQVTPPPSYQTLFKPPFYYERGGTKKF
VNGRDYWIVDGKNGLKQMIPLRTPSALLFQFSN
>PstrIR44
LILCVVGTGFILSLGMTVNNWVRHKLGGRRREFSESIVWCVGIFTQQGSIWKTRSLTEKIIVLITLFLTVLTYNSYSAFITSILSVELY
KIQSVTDLLESDYKIGYVKNGEDEDYLRSVNISELKQIYLRGYLQHDLINISEGLMKVTSDHFAFFASGQSARTHLLNVTKGRCKF
EIQEIKIPYTNEYMAILMSKNSPYKKLINLSVIKMFETGVYKYINSAVYPAIKQCMKHASFQSARLADLSTAFFIVLIGWMASLVL
MSLECVWKKRRIIRGNLKRRFQNGSDTAGAINAQLDFDTYVKFLVRNNAQPFEY
>PstrIR46
MRVETCLRFLFCLIFYQLHYNEGSKAVVTIGVLLQELASQINIPLNSTIYKKNMFDQRAYFSTKILKVSPSDNFEASQTLCTLLDSD
LGVVAVYSDSSRTTPILESTCTFFEVPFITTSWKAPSPAKRNPEEVRAMINFFPEAELFAKGLAEIVRSLQWPSFIIIYENEEGLIRMQ
EILKLQEINPDSKKNNIVVMHLGPGPDYRPLLKKIHNTTEDNIILDCEVDKILPILTQANSVGMLGLHNRYFITSLDAHTLDFSNLN
TTANITTIRLHDPKSDDFLNTVHRWELTEFENHNRRVPLDPYSIKTETVLFHDAILLLTDTVNAMTIKPGINIKPLACNGTETSDDG
FTLRKYMLINTPSMTLTGPLKFNDEGGRTDFNIHVVDIIDDSVIATWHAGNESMELHRNYNQTFDAAVSNLQKITVIVASKIGEPY
LMLRQPSYEGEILTGNNRYEGYSMDLIAGIAKFIGFNFQFEITDKYGNYDYKEKRWNGLIGELLEKRAHLAVCDFTITPERREVV
DFSMPFMTLGIAILHKSQTEKPIDMFKFLEPFSTRVWLYTGTLYLVISIVLFFISR
>PstrIR47
MRPVLLLLTLFRVITGDQQSVTVDLISDFLLKLNAPTKINAHICWPKEEQIALLKRLSSSNFGCRIGNFNNISYSTPAEHQLFLLDLS
CKGSEKILNKASELNLFNQPFRWLMWGVMDRSILDNFYIRLDSRVFIVEKTETDAYRIESPYKVTENSLEYSMNHVAEWNRIGGF
SVYRELSYSRNRSNLMGLNINISYVVTNKDTMNHLEDYRNKHIDPISKLNWFIMKHLISLLNATSTPLFQPSWGYRDVNDSTKFT
GMIGDLQSGKAEFGGTALFFTIDRIDVIEYIAPSVPTFMKFIFRAPPLSYVSNVFTLPFDTYVWYSCFGLVPLIFVVVYVIVVWEWK
DPVFKEKVGEMHANCISPLRPSFIDVLVMELGAITQQGTDTEPKSNAGRIATVFAFIACMFLYTSYSANIVAILQSTTESIKTLDDLL
NSRISLGVEDIVYAHYYFKTAEEPVRKAIYQQKIAPKGQKPNFMTIEDGMSRVQKGFFAFHVEVSNGYKVVADTFQENEKCSLKE
IAFINLVEPWVSIKRASPYKEIVKVGLRKILESGIQRREINQLYTKKPVCHSKGSNFDSASILDCYAAFLIFGAGLAVSFLCLTFEILL
HRKFKRLMSSNVVLNYELPNDDELAKSSVY

>PstrIR49
MIKMIKMIMRLALFLLLVAQEGRCQTTQNINVLFVNEEGNEVADRALDVALTYLKKNTKLGISVDLRRVVGNRTDSNSFLDSLCS
TYNQLLQTQVYPHLVLDTTMTGLGSETVKSFTAALALPTVSASFGQEGDLRQWRNIDQDEQDYLVQICPPADVIPEIVRSLVLNQ
NVTNAAILFDTSFVMDHKYKSLLQNVATRHLITPIKDGNDVVEQLNQLNKLDLYNYFVLASIANIKRVLDAADTLGFFNRKFAW
HAITQDEGELKCICKNATIMYVKPLPNATYQDRLGTMQRTYQLNTEPIIASAFYFDLALHSFIAIKDLIADGVWKNAMGDYITCD
DFNGNNAPKRKGLDLKKYLNKVSSQVLRVEPANSVGLVQETSEMFTYGPISVVSNGLSYMDFQMTLSSVGVREGASDKSLALG
TWTSGFDRNLTLVDPKAMSNLTADVVYRVVTVEQKPFIFRDETAPKGFNGYCIDLIDKIADILKFDYELVAVDKFGTMDENGKW
NGMVRELMEKRADVALGSMSVMAERENVIDFTVPYYDLVGISILMKLPETLTSLFKFLTVLENDVWLCILAAYFFTSFLMWVFD
RWSPYSYQNNREKYKDDEEKREFNFKECLWFCMTSLTPQGGGEAPKNLSGRLVAATWWLFGFIIIASYTANLAAFLTVSRLDTPI
ESLDDLSKQYKIQYAPLNGSSVQTYFERMANIEARFYEIWKDMSLNDSLSEVERSKLAVWDYPVSDKYTKMWQAMKEAGLPD
SLDEAVRRVRASKSSSEGFAFLGDATDVKYLEITSCDLTAVGEEFSRKPYAIGVQQGSPLKDQFNTAILQLLNRRELERLKEKWWN
KNPEKKNCEQVDDQSDGISIQNIGGVFIVIFVGIGLACVTLAFEYWWYKYRKGGRVVDVQEALPQNKQTNLGDDDAPPFSKGKS
FKAKMAFPRARF

SNMP sequences

>BlonSNMP1a
MRLYLKLAIGSASALVFIVSVGFLIFPKMISSKIKSMVNLGPGTDIREMFVTIPFPLEFRVYLFNVTNPMEIQNGAKPIVKEVGPFCY
EEWKKKVNIEDMEINDTISYNPVDTFLKTKYPGCKTGKEQITIPHPLILGIVNTVSRQKPGALSLVNKAFKSIYSNPTSIFITETADN
ILFDGIVINCGVKDFAGKAICGQLRDSSTLRKVSDDELAFSLFSPVNGTEGKKFQALRGTKNFRNVGKIVQYNDEETMDVWPSEE
CNIIKGTDGTVFPPFLKKEQGLVSFAPNLCRSLEAYFVEDTKYDGIPVRHYTANLGDMSKNEKEKCYCYTPETCMKKGMMDLYK
CSSVPIYASLPHFYDCHESYLRLVDGLAPNKTKHSIKILFESMTGGPVYAKKRLQFSMPVVANEKVELFKNLSESILPIFWIEEGIAL
NNTYTKPIKDLFMLKKIVTIAKWMILVLSLIGLCAAGYLYFKQENQVEITPVEKNKNSDQQNTISRILAKNITGQDNIAMSNNGLD
KYY
>BlonSNMP1b
MLKYQKFATVGVAFVIGSVLFMFLGLDTIVKIGIKNQVSLKKKGEVRDIYLKLPFPLDFRVYFFNITNPNEVQNGAKPIVQEIGPY
CYSEFKEKIEVIDNENEDTLTYTPYDLFRFNKEMSGNLTEDDYVTVLNPLLVGMVNQVVKDSPALLSILNKALYTIFEDPQTVYLT
DKVKNILFDGSRINCNVTDFTAKAVCSQLRLQIPGLKEPQPNIFLFAFLASRNSTKGKRVKVDRGIRSTKRIGTVMEFDGKQELNM
WSSSECNHFNGTDGWIIPPLLEIEEGIAIFSTDLCRNVHAEYINSTTYKHVNVRLYQSNLGDMEHNDEEKCYCEAPNTCLKKGVV
DLSKCLGVPLYATLPHFLLTDESYLDQVEGLRPSMEKHIIELLLEPLTGTPLNAFKRMQFNMQIGPVPKINLMKNLPVALHPIFWIE
EIVDLQGPVLSKIQTVFKALNVTKIFRWIILGSGMLLMGFGGYLYFHNRKMFQVTYINDPKTKSSKSTSEKFDAENLSIQSVPKSE
NIKRSFNNSVMTGREFDRYNIQ
>BlonSNMP3
MKIMVNKKFCGIGDKTIILLEILGLFFIGVGLYGGFKLVPGVIQGKIWEMKVLQEDTEQWNMFLKMPFPFTFKAYLFDVQNPEGV
LSGEVPIVKEKGPFAYKVYRWKTDIEKGPSDEISYFGYTRFEFDSEMSGDHTEEDIITILNTPYLSLFYVAEETQPDALSMIESALPT
VFGQNDGIFVKVKVKEYLFDGLRICEDGGNSGGFVSSMICRQMLSQITRAKNMRLDKNNTILFSALHYKNNTHLGRFTIKSGVS
NRKETATLFRYEGQPYISKWSGEESSCNKIGGVTTVFPNPVTKDMVFESFSEDICRTMKLEYSNDEIVKGILGYRFVGSKDNFNYD
IEGNKCFCVAYNKSLNNAKCLKNGVLDLAPCSGGPVILSFPHLLNADPEYISNLEGLHPESSKHETFVTLEPTSGFPLKLAQRVQF
SMFTRPVPGISFMENITKALIPFMWVEESTTLDDKYIDMLKTKLLSTIHIVDVVKWIVVGSGIACVILASYLAVYIKSA
>BlonSNMP2
VYIFHVNNAAEVMEGAKPNLTEIGPYYYSQSVERVISGLNSEDDSVTYTQIRNLTFNEVKSAPLKKTDRLTVINPILIGITQTPISGF
EWFILKGCLGSILPSNFNSTFIEVEVEDFILNGLVFARDTDVTTMACYPVRDLFIRRSRVIRNVDVVSYENDTVKELKFSLLNFRQS
KEEGIYTMNRGIKDITQLGNIMKWNHRDQFPYWGTDLSLNNDKCNTVRGSDATIYPPGATKTQGYEIFASDICRVVAINYDGTGT
YKGIDGYYYSIDENTLRTKYSDPEQDCYCTKKTSNQTGSPSCFLDGIMDVMPCFKAPILLSWPHFLYAEHYLDGVDGLSKPDPKI

HKTTVLLEPNTGTPLEGEKRVQLNFVLRPNDIDGSKLLPTILPFVWLSEGVKLTQELVDELDSKYYGQIRTANGVIYGLIAVSAAA
FCISLVFLLKKKCCTDDR
>CbowSNMP1a
MRFPVKLAIGSISAFIFIILVGFVLFPRMITSKVKGMVNLAPGNEIRDMFIKVPFALSFKIYLFNVTNPMEIQSGEKPIVKEVGPFCYE
EWKEKMNIEDKEEDDTISYNQKDTYLKKWWPGCRNGQEEVTIPHPLILGIVNTVARQKPGALSLINKAIKSIYSDPSSIFLTAKVD
DILFDGVVINCNVSDFAGKALCGQLRTAEALTKVGEVEKFSLFSSKNATLQKRIKAYRGKKNHRDVGRIVEYNSSKMMDVWPTE
ECNSIEGTDGTIFPPLTKPGEGLFMFSPDLCRSLIAFFVRKSTYDGIPCGEFTADLGDMSKNEKEKCYCSTPETCLKKGMMDLYKC
SGIPIYASFPHFYNSDTSYLKGVGGLSPNKTKHEIKILFESITGSPLYARKRLQFSMPLESTQKVELFKNFTGTVLPIFWIEEGVGLN
RTYTGQLKSLFTLTKVVKVSKWLILIGSLGGLAAAGYLFFKVDGRADITPVHEIRRHESKSGSTVNGAGGHVLSGNGLEKY
>CbowSNMP2
MKMFGASRFCNVKILFVTTVVATVVLIGVLLLSFVGMPLIVNDQLAKKLRLENNTEQWDRFVELPVPLNLNVFVFNVTNSDEVT
NNKATPILQEIGPYCYEERITRKILSANSTEDSITYEQSFNITFDEKRSGQWKESDKIVMVNPLFLILSQITNVIERFVVMGCIDKLFP
PKYSTMFFEVDIKTIMLEGIEFGVASDDIGPACNIVRNKLLEKTLPMKNVERIPSPTDPSVINSLKFAFLQYKIRGPDGQYTTNRGID
DITQLGHIMRWDHSAEIDVWGRGESTNNATCKEVKGSDSTIYPPHVTKSTKLDIFSTDICRTVQIRYKGTGTYQGDSGYYFGIDE
NTFRPATPSPENDCYCIQQTMAPDGEPSCFLDGVVDVYPCFGAPILLSFPHFLYADESYLDGVIGIDPPNSSIHEIFLLIEPNTGTPLQ
GMKRIQLNVVLRPVEFVEYTANLPSTVLPLIWIEEGVNLSQDLLDKLDKMYFNVIKAADAAKYAAIGVLTAFVLISGGFFVRKRY
FK
>CbowSNMP3
MKFYSVLFVVKDRANMLNKFNITVSGKIIVILGVFGLFCIFAGFYVGFKAVPDVITDKIWDMKVLKENTEQWGMFMKTPFPFTF
KVYLFDVQNPQEILQGAKPVLRETGPFVYKVYKWKSEVEWDTPDDISYFSYMRFEFDRKASGIFSEDMKVTLFNTAYYGMLQK
IDETQPEVLSTVEGVLPSIFGENHGLFIKVKVKDYLFDGLKICENEGKDGGFVAGMVCKQMIARLPESKNLRLEDNSILFSNMHY
KNNTHQGRFTVKSGGQNRTETATLTLFNGKSYISSWTGEKSMCNKIRGATTVFPVNIEKNMTFEAYSEDICRTIPLEYSAEETVKD
IVGYKFSAMNDSFSSTKKENFCYCTNTTRTLDGEYGCLKDGVTDLKTCIGSSILVSFPHLLYGDEEYLDSVIGLNPEKSKHETTVIL
EPISGFPLSVTQRIQFNTFLRPIDNVISLENVSKSLFPLLWVEESLILDDQYTDMLKNELFRTIKIVDIVKWVTIGSGAACVLIALILR
MSSKTT
>CbowSNMP1b
MRLPLKLGVAGFLLILLSVIVGFIALNPIVRFGIRQQTALKRKSEIRNIYLKLPFPLDFRVYFFNISNPMEVQKGATPILTEIGPYCYD
EFKEKIDVLDNDAEDSLTYYPYDIYKFNAEKSGKLSDTDYVTILHPALVGMVNQATRDSPALLSIVNKAIGPIFRDPESIYLTAKVK
DILFDGVELNCKVTEFAAKAVCTQIKSQIPGIKSDPEKSIFLFSLLGVKNATVGKSIKVSRGISNSRDLGKVLEFDGKKVLKLWYEE
QCNHFKGTDGWIIPPLLKPEEGLWSFSADLCRNVVAEYVEDSVTKGVKTRRYEATLADMQNNEEDKCYCPTPKTCLRKGVFDL
SKCMGVPILATLPHFLEADEIYLQQVKGLNPILDKHIIRIQLEPMTGTPIEARKRLQFNLPVSASEKITLMRNVSTSLHPIFWIEEGV
ELDGALLEKVTEVFTFLGVFQVFRWLGLLIGFVSIAYAVYHHMKHSRSVHITPISGSSSSDHVDINRSTNELVGKMKEVFQSDKGH
TNPVMTGHEFDRYS
>DvalSNMP1
MSQHQTPLFKGMVNLVLRDSPVFLPIVSKAIPSIFDNPQTIFLTAKVKDILFDGVELNCSGKEFGTTAVCSQMKSQIPGLKFKKDNE
NIFLFSLLGSRNGTLTRRLKVHRGITHAKDLGRLVELDGKKEINIWREAECNRFHGTDGWIFPALTTPEEGLQSFSTDLCRSITLQY
INDTVLKKVPVRVYETDLGDQMTDENEKCYCRSANSCLKKGVFDLTKCMGVPIYATLPHFLRTDPSYINLVDGLAPSELLHAIRV
YFEPMTGTPLFAAKRMQFNLDLQPTNKIPLFSHLPIALFPMFWLEESVDLDGYLLKKVQ
>DvalSNMP1a
MNFPMRLAIGSACSVVFIILVGFVGFPKMIKGKVKDMVNLKPGMEIREMFLKVPFPLSFNVYIFSVLNPAEVQGGAKPHLKEMGP
FCYNEWKTKINVADNEGDDTISYEPVDTFKNAKRPKCLSVDTQVTIPHPMILGMVNTILRQKPGALTLANKAIKSIWSNPSSLFIT
VKAQDLLFNGVVIHCGVSDFAGKAICTNLKAEPSLTHLGGDDLGFSLMGPKNGTAGKRIKAFRGTQDFHKVGRIIEFEGKPKLD
VWNNSKCDAIVGTDGTIFPPMLKKEEGLASFAPDLCRSLIAQFEKHDKYDGIPVSSYFATLGDQSRNPTEKCFCTTPETCLKKGL
MDLYKCAKIPLYVSLPHFYDSHESYLKGVKGLKPDVEKHGIRIMFESLTGSPVSARKRLQFNMPLEPNPKVELFHNFTPTVLPIFW

VEEGVDLNNTFTKPLKTLFLTKKLVNVVKYLVLMMSIGGFCAAGYLYFKSDNSMNVTSVQKVQPDQNGHRNIISTVFNGNHTA
GQDNEAYEDKH
>DvalSNMP2
MKVPFPFQFKVYLFIVENAEEIQAGVKPLVKEQGPYVYQLTRWKDQVAWNHSTDEISYHEYEAYKFDAESSGGLSEQDLVTVLN
PAFLSFLYTAEMDPATRDFLPLIDESLDAIFGSYNSPFFTNITVREFLFDGLRICKNGCNDDGFVAKMACNKIKERMAVTKQMRAD
GTDILYASFHYRNNSHQGYITVAAGQKNETTIGEITQLNHQSTLNVWTKDQFECNRVSGLTTVFPTNVGVDTTFQSFSEDICRTVS
FEYSRREMVGLIKGNRYEALKSTFNTSKNACFCTDRTRNFDGNVGCLYNGVLDLTTCQGAPVLVSFPHLLYADSRYLDKVEGLN
PDPSKHAMFVTLEPTSGTPLKVAKRVQFNLLLRAVRNITSLETVGNSIVPMFWIEESTALPEKYQDFIKNKIYRTLLIMDIVRYTVL
ALALAVIVCCIVLFIYAT
>DponSNMP2
MFRNCCSPRLVFLYNLLAVLLLIASLVLAFWGLPQIISKQIHKQTELTENTDQWDRFKELPFPMEFNIRFFLVTNPADVLNGSMPIL
KESEPYKYKSTIKRTDIRFDDIEEDSVTYRRSFSFEFDGSGTTREDDSITVINPLLMASFQLTNDIQRLAMAGCRKYILEPAGLDQV
FLTTTVRKLLFDGIYFGFQNATGKGVACEMVRKELGKIVANVRVVEHLNDTDCYRLAIFNYKTDNFLKNSPDGIYTINRGRNNAT
ALGSIMRWNGATTSTTYGTSTSINNLTCHSIKGTDSTIYSPELKAGENLMIFNTDLCRTIQLVQVSSNEVFNGINAFRYSTGYTLFRP
ETILKENDCYCSHGTKGADGKPSCFLDGLLDFRPCLGAPVLISQPHFLHADVKYIRAVSGLSPDEDKHDIYLLLEPNTGTPLEGRK
RVQMNSVLRRQPLLSMITPPNMYEAVVPLLWLDEGFTLPQKYLDDLNAKYFKTVRIATGFKFGFIAVALALLVGCLFVACRKMY
FRNAK
>DponSNMP1a
MNFPMRLAIGSACSLLFIILVGFVGFPKMIKGKVKDMVNLKPGMEIREMFVKVPFPLSFNVYIFSVLNPAEVQGGAKPHLKEMGP
FCYNEWKTKINVEDNEGDDTISYDPVDTFENAKRPKCLSVDTLVTIPHPMILGMVNTILRQKPGALTLANKAIKSIWSNPSSLFIT
VKAQDLLFDGVVIHCGVSDFAGKAICTNLKAEPSLTHLGEDDLGFSLMGPKNGTAGKRIKAFRGTQDFHKVGRIIEFDGKSKLD
VWNNSKCDTIVGTDGTIFPPMLKKEEGLASFAPDLCRSLIAQFDKHDKYDGIPVSSFFASLGDQSKNPAEKCFCTTPETCLKRGL
MDLYRCAKIPLYVSLPHFYDSHESYLKGVKGLKPDVEKHGIRIMFELLTGSPLSARKRLQFNMPLEPNPKVELFHNFTPTVLPIFW
VEEAVDLNSTFTKPLKTLFLTKKLVNIVKYLVLLMSIGGFCAAVYLYFKSDDSMNVTSVQKVQPDQNGHRNIISTVFNGNHTAGQ
DNEAYEDKY
>ItypSNMP1Fix
MPHPKNIAWAGGALAFGGVLFKVWLFDVLVRFGVKDQTALRYRNEVRGIYLKIPFPLNFKIYFFNVTNPEEIQNGAKPVLNEVGP
YWYDEYKERVDVIDNDTEDSLTYTPYDLFKFNPNMSTPLSDNDYVTIIHPVIVGMVNLLLRDSPMLLKVVSKAIPFIFNDPKTIFL
TGRVKDILFDGVVLNCTSKEFASTAVCGQMKGQVPGLKPTPGQPNLLLFSLLGPRNATRTGSLKVLRGIKHFQDLGRLLEVNGRK
SIGIWAGDQCNRYDGTDSWIFPPLIQPESGLKSFSTDLCRNIKMKLVNETVVKKIPVGVFEPTWGVKVVTRRKSATVPTLPVXXX
XXVFDLTKCMGVPLYATLPHFLDTDPNYLKLVDGLKPDHEKHRIVVFFETMTGTPLKAAKRMQFNLELQQTNKLELFSKLPAAL
FPIFWLEEGMELEGYFLKKIQTVFMLLLFADVTIYVTIATGLSVCGAGFYQYWKNTKSLSITPLTKNNNGLSEPKLN
>ItypSNMP2Fix
MRFLQRVKFNLKTVFLCGISGVSLLVVALFLGFIIFPKVVNDQLLETKILREDTEQWAIFKKIPFAFTFNVYLFTVENPEEILKGAKP
VVKEKGPYVYKLYKWKEDIIWNYTTDEISYYEYEKYVFDQEASGSLTEHDKVTLLNLPYLTFLYTAEANEATSGFLPLIDEALEFI
FSGHNSPFLVNVTVRDYLFEGVEICKNGCEDDGFVAKMACGKIKDNLKVAKQMRLHHKDILFATFHYRNNTHQKYLTVNSGRQ
NHLEIGAITQLDNSSTMNVWNQFGCNQVSGLTGIFPINLGFKTTFQSFSAEICRPVKLHFSTIKPFGSIKGYKYVALNTTFNTSMVE
NQCYCTGKIPNLDGNLGCLYDGVLDLSTCLGAPIVVSFPHFLYADWRYVNNVKGLSPNETNHQIFVNLEPISGTPLEAATRIQFNL
FLRPVRNITSLDSVADALVPLFWIEELTYLPQKYQDVITGKLYRSIFILNAIKYVLLAIALVIITVCILIFLYTD
>DmelSNMP1
MQVPRVKLLMGSGAMFVFAIIYGWVIFPKILKFMISKQVTLKPGSDVRELWSNTPFPLHFYIYVFNVTNPDEVSEGAKPRLQEVG
PFVFDEWKDKYDLEDDVVEDTVSFTMRNTFIFNPKESLPLTGEEEIILPHPIMLPGGISVQREKAAMMELVSKGLSIVFPDAKAFL
KAKFMDLFFRGINVDCSSEEFSAKALCTVFYTGEIKQAKQVNQTHFLFSFMGQANHSDSGRFTVCRGVKNNKKLGKVVKFADE
PEQDIWPDGECNTFVGTDSTVFAPGLKKEDGLWAFTPDLCRSLGAYYQHKSSYHGMPSMRYTLDLGDIRADEKLHCFCEDPEDL

DTCPPKGTMNLAACVGGPLMASMPHFYLGDPKLVADVDGLNPNEKDHAVYIDFELMSGTPFQAAKRLQFNLDMEPVEGIEPM
KNLPKLILPMFWVEEGVQLNKTYTNLVKYTLFLGLKINSVLRWSLITFSLVGLMFSAYLFYHKSDSLDINSILKDNNKVDDVAST
KEPLPSANPKQSSTVHPVQLPNTLIPGTNPATNPATHHKMEHRERY
>DmelSNMP2
MIHWSLIVSALGVCVAVLGGYCGWILFPNMVHKKVEQSVVIQDGSEQFKRFVNLPQPLNFKVYIFNVTNSDRIQQGAIPIVEEIGP
YVYKQFRQKKVKHFSRDGSKISYVQNVHFDFDAVASAPYTQDDRIVALNMHMNAFLQVFEREITDIFQGFANRLNSRLNQTPGV
RVLKRLMERIRGKRKSVLQISENDPGLALLLVHLNANLKAVFNDPRSMFVSTSVREYLFDGVRFCINPQGIAKAICNQIKESGSKT
IREKSDGSLAFSFFGHKNGSGHEVYEVHTGKGDPMRVLEIQKLDDSHNLQVWLNASSEGETSVCNQINGTDASAYPPFRQRGDS
MYIFSADICRSVQLFYQTDIQYQGIPGYRYSIGENFINDIGPEHDNECFCVDKLANVIKRKNGCLYAGALDLTTCLDAPVILTLPHM
LGASNEYRKMIRGLKPDAKKHQTFVDVQSLTGTPLQGGKRVQFNMFLKSINRIGITENLPTVLMPAIWVEEGIQLNGEMVAFFKK
KLINTLKTLNIVHWATLCGGIGVAVACLIYYIYQRGRVVEPPVK
>TcasSNMP2
MGCSCCTIKVLLVCVVISVALLIVSLALAFKVFPDLLESEVNKAVRLEDGTKQYDRFVELPFPVDFKVYLFNVSNPQQVLDGTEK
PKLEEIGPFVYKQYRKKTILGKNEEEDTISYTQKETFEFDAEASKPLTEESVVTVLNPALMSIYQLAEDLHLAGAADTCIKQTFEN
NQGKVFIEANVRKLLFDGFSFCKNTSPGICGLVNDLICAIAATKRNSDLVLPDYSLIFSYLNYKRKPDDGKYTVKRGLTNIEKLGH
IVAWNDSLYTKFWGEGTTCSEVKGTDSTLYPPRVTTDSAFYIYSTDICRFVKINYKGEESYKGIDGYLFETSEDTLRSSAPEEDCYC
SKLSRDMEGKKSCFLDGVIDMQTCFGVPVLFSFPHFLWADNKYLSAVEGLNPVEEKHKTYLVVEPNTGTPLKGMKRIQLNGVIR
PIVGIKSMLQTKRALLPLLWIEEGVSLPQKYVDELKSSYFDKVQIVDGVRYALIVISAILVGAFGIIILRKRSHAKHHV
>TcasSNMP1a
MRLPVKIAIGCAIGLVVIIVFGFIAFPKMIKGKVKSMINLNKGSEIRQMFVKVPFALDFKIYMFNVTNPMDVQKGALPVLKEVGPF
CFEEWKEKVDLDDNDDEDVMFYNPKDTFYKANGPGCLDGSQMITMAHPLILGMVNTVVRTKPGAISLISKAINSIYGNPDSIFM
TASAMDILFDGVVIKCGVKDFAGKAVCSQLKEAPDLRHVDENDLAFSFIGPKNATPGKRFKVLRGVKESHDVGRILEYDNKKEM
EVWPTKECNQYKGTDGTVFPPYLTKEEGLASYAPDLCRSLVAVYSGDTKYDGIPVRIYTATLGDMSKNADEKCYCPTPDTCLKK
GMMDLFKCAGVPVYVSLPHFYESDESYVKGVVGLNPNKKDHGIQILFESTTGGPVKAAKRLQFNMPLEPNPKLPIFANLPNTVL
PLFWVEEGVALNNTFTKPLKDLFKIMKIVKIAKWLIMLGCLGGLGAAGYLYFSKKGEANITPVHKVKPAENGVSTLGGEVNHA
MSDNEIEKY
>TcasSNMP1b
MVKWQRQLKPGNEVRDFYIKLPIPLDFRVYFFNISNPEEVKQGEKPILKQIGPYCYDAYKEKINVEDDKDNDTLTYNPYDTYFFN
QMRTGDLSQDDYVTILHPLTVGIVNAVATQKPQYLSAVNKALPVIFKENSSIYLTAKVREILFDGVLINCNVKDFSANAVCSQFKG
QPAMVEVEKNIYSFSLLGSRNGSIPTRITIHRGVKNAADIGRVVTIDNKTDLDVWPEPECNAFRGTDGWVFPSFLEKEDGIWTVAS
DLCRSFKAQYVEDLKFHGVVVRKYFADLGDMSSNPAEKCFCPAPEKCLPKGVMDLTKCMKVPLYCTLPHFLRADEKLLQQVEG
LSPELERHIIKIYFEPLTGTPMLGQRRIQFNLQLMPIPKVAMMKTVPEALHPILWIEEGVELEGFLLKKVTSVFTLLKLMTFVRYIM
LGLSIQGILYGGYKLYQESKSKKVSPVQNGTTESKNHNQGKTGGIELPSMNKRNKENTKNA
>TcasSNMP1c
MSYKKITIISACCVVTIIGVAYIYAIRDISHRRNVRYKYIDRVNNVSNDVNGGVVSVGYCYDYKRIDVDNADSTYTYDIYNRSGNS
DDYVTIIHVVSVNYVSVKTHYNDAGKSITAKVRDIDGMINCTSRDTAMAVCTIRTKIGISKDYKYAGNGTTRITVRGIKSNGKVAV
DNVTKSDWSNCNYKGTDGWISGRKTIWMHATTCNIHADVGATSNGAVNKYYSDNICTNCSCGIDVTKCTAIYISHRSDSIRGVK
GNDTSHITRIGTSMAIRNVVKKITIMNVSVIHVWVMGVVNGWRMIKTYTAVMKYISVASGTAYGGYHYKNKKYSKNIVSSK
>TcasSNMP1
MTSTARRRNIMKKVYKIMDRVYNITNSVNGVVKVGYCYDAKKIDVNGDSTYTYTYNDKSGRTADDYVTVHIVGIVNTVSRDSI
VDRAIKSIKDNIYITTKVRDDGMTINCKVDSATAVCTKAIGIIKNVYKSIGRNGTNRYKVRGMKKWHGRVVNHKSTVWSTKKCN
RRGTDGWIIDKVGWTYSSDCRNMHVVTSHGVAKYYADGDMSSNDKCYCKTCKGMMDTRCMGVIYATHRVDKVRRTVRGKIT
DHIVRVIIGTAKRMNIVKKISMKTAHIWIAIVGKMIKVVVAKVDVVKYCAVCAVAGSYCYKRKKKAVTVSKTAKA
>AplaSNMP1

MYMKTPFALDFKVYMFNITNAEAVLNGESPVLDEIGPYCYDLWKEKVDPIDNEVNDTLTYKGKMTWIFNKAKSAPLTGDEMVT
IPHPLILGIAVAVARDKPAMLSLVSKALNSIFNNPPSPFITATTNEILFEGLTVYCNVTDFAGKAACAQIKSEAKNVIYISDKIFKLSFF
GDKNGTVDERPFTVKRGLKNYKDIGRVVEFDNKPNMNVWPTKECNEYHGTDSTIFPPLLQKEEGIVAFSPDICRSLAAVFEKETF
VKEVKVNKYTATLGDMSADDSLKCYCPEPNKCLKKGLMEITKCVGAPLYASLPHFYASDESYVHGVRGLHPNEEEHGIYMYFE
PMTGTPLGARKRLQFSMPLEPIPKISFMKNLPTTILPVFWVEEGADLGDEYVDQIKSAFKMIQIVFTAKWILFVVSAVTGAIAGLM
HFKNSKNILVTPVKNDESKGRSSVINTIDSLNTYVNGSNNKY

OBP sequences

>BlonOBP1
LSEEMQELANMLHKTCVEETGVAEHDIENARGGIFADTDNLKCYIKCIMAQMACIDDDGIIDVDATVAVLPEDYKEKGEPIIRKC
GTKKGSTPCENAWLTHKCYQSESPKDYFLV
>BlonOBP2
MDLYMKLFMFVYIVCEYAMGGFTEEQLELMKNLHNECVSQTGVDEAHIADCAKGIFHDDPKFKCYAKCIFDEMGVIQDDGTID
SEGILAMMPEDIQAKIAPGVRKCGTVVGTDVCDSVYLTNQCYYKEFPNEYFVI
>BlonOBP3
RITEKDLGEKLQKLAKVVHDKCAEKTKLSESLITKLREGDFTIGEKGKEYIYCLWIESKAMDAQGNLNYSFIESLFPLEYKSMIDL
MRECDGKHLNETDAANKIWEMSKCFEATDPEHYIIF
>BlonOBP4
ALHVDHYGEKLLVLSKDLHALCTERFGIEQSEIDKVSSGNFDVNDVFKEYLKCIWVEGDAMNEQGEFQYDVLDVQAPPAFKSAI
LAIVHACDDEIPGQEIVQRIWKLTQCFYEKDPDLFVMF
>BlonOBP5
VKMVRNVCLPKSKASSDDIDKMHKGDWNIDHTAKCYMYCALATYKLINKDNTLNYEGAQVQLKQLPDTFRPSATECVEQCKG
AVVTTDDQCEAAYEISRCFYSCNPEKYFLP
>BlonOBP6
RLSENELGEKLQNLVKVIHDKCVEKTKMTQSGISKLQKGDFTDAEKGREYVYCMWLDGNGMDAKGKVNYDQINSLLPSKHK
NILELVKQCEASNLNEKDAAHKIWAMTKCLHATDPERYIMF
>BlonOBP7
VELPAELQEYASDLHNICLQKIGITEKEHAAFDMKSNQPDPKLQCYMKCLMLEAKWMSPDGAIRYDYIIDTAHPQIKDLLVAAIN
KCRDIQPGGNLCEKASNFNFCMYTADPVNWFLV
>BlonOBP8
RISIQDLGEKIQNLAKVVHNKCVEKTEVNESLIKKLQEGDFTIDEKGKKYIYCLWTESTAMDTKGNLNYPYIESLFPSKYKNMVD
LMKECDQKNSDETDPSNKIWRMTKCFEATDPNRYVIF
>BlonOBP9
AMTEKQLNATKKLVKNTCQNRAKASAAEIDAMQKGNFDTSESANCYLHCIMNTYKLLKSDGTFDWEAGIKVLEINAPESISKPG
SISVENCKDAIKTPGDLCKSSMEIGRCLYNDNPANYFLP
>BlonOBP10
KLTEEQVAKLKEHREVCIKETGAEVEKIDAAKKGEFADDAKLKAYLLCIAKRIGFIDGAGELQSGVLKAKVGAAIDDQELADKL
EPECAVKKDSPEETVFQMAKCFYEKNPKHVVIL
>BlonOBP11
RISENELSEKLQNLAKQIREKCIKRSEISPSSIGKLQEGDFTDAEKGRAYVYCMWNESNSVDADGKINYEVIDGNFPPEQKSIVELI
KECEAAHSSEKDAANKIWEMTKCFHATDPKRYIML
>BlonOBP12
KLNKNELSERLQGLIVVLKESCMTKSGVKEAEIELLKEGDFHVSQNAKEYIRCYWTESTAMDASGNLNYIFIEEIVPKNPPGILD
MVKECEQKSKDYKDPTEKIFRMTECFYETHPELYLMF

>BlonOBP13
ALNPSTFTEKMKELAAQLHNTCLERYKIDPADIAHMANGNFDVSDKDKEYIYCIWKESTAIDENGILNYALIDDLIPIEIGHTMAVVVHACEDQISEKNVDKKIWALVQCIYKMNPDMLVIF

>BlonOBP14
RISIQDLGEKIHNLAKVVHNDCVEKTEVNESLIKKLQEGDFTIDEKGKKYIYCLWAESTAMAIKGNLNYPYIGSFYPSKFKNMIDLVKELNQKNSNEINASNEIWKMIKCFEVTDPNVNKKYGC

>BlonOBP15
RPQDEPNELLVNAHHECATETGVEEDVIKETLQGEFTEDEKLKKHILCVGQKLGFMNEGGELQTEVIKEKLGDVVDNVDELLEKCVVDKGSPEETAFGFAKCTYEFKHSD

>BlonOBP16
MILSFVSSSKVAVSKINNDQANVPFKNCRDKSGATPGDIEALRNRKLPQTKSGKCFLECIFESVKILDDGKFNKKAMVVVFTPALKGDISKLGKLNELSEICAKEIGPDSIPNCEGATRVVKCVANHGRDYGITFPKAKI

>BlonOBP17
VKDFTAEEIRTDLRFIKTCNTTSPISMHTMNEVLIHKKLAKGESSAFKCFLHCLFTKYGWMDEEGGFLLHDIKVTLEEADVEIDSLEFILYKCTAIQSVDKCERSFLFTQCFWDKIAEQQPSEDQLFYSIDDMYQK

>BlonOBP18
VPEPDIMGIVEHLSHDPFFKSCGDIEGISSTDDAKFLCYFKCMSEKSGFINEAGNVDTSSISNLPIPREAQEKIKKCFGSVEKVSSCDDMKPIVNCFS

>BlonOBP19
MKYFSFSFVVFVAIVSCFVVTADDTEILKIMKNVSKNPIFHPCGDIQGVTDPNDEKFLCFIKCSAEKSGMLTPAGEPNANAVANFLFPDEMKDKITMCLKRIQKVNTCADMKPFMNCFR

>BlonOBP20
YEFDDAIYNQLLANELNDISYTAYSHPRVRRDDEAASQKAPRCPPPHFRKPKLCCADDLVESMHKEERQLKRGCFKNITGSDDSVRPPPPPGSPPPFGADPLNCEEINKMRGQIKCVHQCVGQKLNLIDADGNMKEDEISKYIKTKISPMNLEDSVQTQIVENCITEAKSTSRNTDQSKESCNPAFLRISHSLFKQIQLNCPEEQITDKEYCTKFQNRLKKGPRMPDSDESPPPPPPEMSSEEK

>BlonOBP21
LECGFKKIKKTDIKDALSSCVKNNETLHSILEMTSSSTVSPSSESNEDGTTTSSRSDDKDRNGKSSGRIKRATKGQKSKSSKDDSEEDRTTEDGRNASEETSEKCIVQCLLKELDLLDSKGLPDRKKIVDGAMKNSAGRELQNFVQKTLDDCFEEIKKENKTDSCEFSNELISCLAEEGRSNCADWPAGSLPF

>BlonOBP22
QNIPPEELTNILRYHQQCREQSHISDAEVRNVLLGDFTYTPQLGQHLFCVSKRIGFQDENGRINRNVLRQKLSLVSNDQQTIDRLVNQCSVQQSTSERTAVKTAECFYNDIRRLVMGK

>BlonOBP23
ASITPEQKAKLKVIKETCFKESGVSIQLVQDAHNGNFANDPKLKAFLVCAAKKIGFVDENGDLQHDVLKAKAGAVLGDQKLADKLDAECALKKATVEDTVFEAVKCYFEKSGKRSVLF

>BlonOBP24
MKQRELIGIHCLKENELDRRVVDRTMLTLEFPVENQKYKDFLACSYKLQGFQTQDGKMQFENINHFLSRFYSTADLKKIDVCKSNSGKNDGERAFNAVVCIMDQLKTIEVKSDNEI

>BlonOBP25
MDLSAEDKQKLMDMHNACVAETGVSGDLVTEAMAGKFPNDPTLKAQLFCMSKKLGFQNDAGELQMDKIASQISKYVDDPSLADKLIKCGDQKETPEDTAYEVAQCAYKVKFGMA

>BlonOBP26
GIFTEDEKGAIVNTMKECIKETNVDIDLLKKAQVGQFADDEKLKKQLFCFYKKVGVQNDNGDIQMESVTPYLENIITDPVQRDEAVSKCLVRTGSSEETAFFMSKCLHDYIPNPDILS

>BlonOBP27
DYTRHGIEERTQHAVDACIAEKDMNLPADIKEQVRYPDKMERPLLGKIAKCTMVKMGYMTTDGEIAKDVIKDKIKGKMHDPAK
FDMLEEECNKKQATPEETAIYLHTCVREFHEKHCSC
>BlonOBP28
EELANNVTGKCDIPPTAPKRIEEVINSCQDEIKIAILSEALAALNVNDHTSSRAKRSTFSDDERRIAGCLLQCVYKKMNVLDPNGY
PTVEGLVSLYTEGINQKEYILATLQAVTKCLGKTQKELLALPQSIDVHGITCDVAYEVFDCISRDIENYCGQSP
>BlonOBP29
SDFRLPQSEVDQLKSIHERCQAKSATYVDEDKLKHLKKYQDDKQVGIHMLCMAGLAELVNSDNTLVVDQIKRYIKLGASPDDN
VDELTKKCAVLKDTAEHTSVQLFICFYTNDIHYYHRL
>BlonOBP30
DAPFRLPDNEVRNLKQIHDSCQSNKKTYCDENILKVLVQNQNHRQAGIHMLCMAEKAGLVQHNGSLNVGTIRKKVALGAKPGT
SVDTIVNTCAQRKGSAEATAVQLWICFVQNDVHYYHRL
>BlonOBP31
DPEGDRAKFKKAHEDCQADPDTKVSEDYMRNAARGEHTHGEEHGKAHGLCMSKKLGFQDEHGKVVKAEVKKVLSRGITDSA
KLEAAVAKCAVDKDTPEDSARALWDCIREESGPRGRGPPPHEHH
>BlonOBP32
DPSVLGKDVLTNSSKFHARNYTNDNESNQKESDDVFKNEITKNLKQCVSDSSNGDEDGGNYQNNREKTQYRRNSQSDNSDDY
RYPNNDNYYNSGYSRQYGQRMKRGSNNSEYQNQCISQCVFGHMEVLDDDNVPSETMVIKWVQDHIANDDLKRIQTLREIRKC
FARLSTSDIDDGCEFSKELTKCLNIDME
>BlonOBP33
HSSFRLPEREVKKLEHVHDTCQANKKTYCDENLLRNLVNNQNDSQVGIHMLCMAEEAGLMKHDGTLNVDTIKDKISLGAKRG
ASVDSLVTECAKSKNSAESTAVQMWICFVQNDIHYYHRL
>BlonOBP34
DSSFRLPNDEVKKLKDIHEACQADKATYCDEDLLRNLVENQFNKQVGIHMLCMAEKAGLMKHNGRLNEDTIIDKIVLGAKSGA
LITSLTSQCAQSKETPEATAVQLWLCFVQRNIHYYHRL
>TcasOBP0A
QSLSEDEMRENARKLMTSCKDKVGASDADVEALKMHQMPESREGFCMLECVFDSAKIMQDGKFSKSGMIEGFKPLIGDDKAK
LESLEKLSATCESELGDGEDKCETAKRLVECVIKNGKTHGFEVPPPRE
>TcasOBP4A
VDQEFVEKFLQKMEKIGEECAEETHATSDDIADLIEQRDPKTHEGKCLIFCYHKKFNTMKEDGSLDKVGSVLALEEVRDADFEL
YKNILTIFVTCGDKAKIYDDPCETATALTMCGRDEAKALGLQDAIFG
>TcasOBP4B
LDQEFVDEFLEKMQEFGAQCAEETDATSDDIAELIARKLPPSTHEGKCMIFCMQKKFNMMKENGGIDRAGAIAALKPLQKADPE
LHQKVLKIFVTCGMRVKPSPDPCDTATELALCGKKEAEAIGLEDALLT
>TcasOBP4C
QDFIDKFVAKVKSIGETCVPETNASKDDISSLLAHKMPDSHEGKCLIFCFHKQFQIQNDDGSINREGAIKALEPLKADDAELYEKV
ISIFKKCESTPVDGDSCLYAASLAECAVKEGRAMGLDNLIVLEIE
>TcasOBP4D
MDESFLQQTRDRVKAIVKECVTEEKATDSDFDDIMALKIPTSHEGKCVFFCSHKKFNMQHPDGSINKEGALDTFEVVKDVDAEF
HDKVITVYNHCLSTPVDPDPCVYSVNLFQCFMKEAKAAGIHELIIK
>TcasOBP4E
IDKEFVQELRQKLRSHVEACAKEVNAGPDDVSAIFAHKLPATHEGKCIFFCMHKLYNAQNEDGSLNMAGALANLELIKDMDPD
VYTKVSTSFKNCESAPFDSDPCLYAANLVTCIVKEGRAVGLDEVLVE
>TcasOBP4F

GLDPKFLEKLTQEVQAVGTSCGEKEHATADDMIEIMEEKFPPTSHEAKCVVACFYKHYKMMKEDGTFDKDAAVKAFDEIKAQD
AEIHAKILKVIDACDAKKQMSDDHCVSAASMAGCVKTEAIANGLTKEAFMAS
>TcasOBP4J
ELDKEFLMQFLQKIKKVSEDCIAETQATKNDIKTLLEHKIPDSHEGKCMIFCFHKHFQIQNEDGSLNKVAAISLLEPIKDHSQDIYD
KVVKIFNTCFDSAERDDDSCIYASNLAECAIRESKSLGLDDLLVIE
>TcasOBP5A
EDAKEKKCDIPPTAPKKIEDVINQCQDEIKLAILTEALEALNINEHTKSRAKRDTFSDDEKRIAGCLLQCVYRKMKAVNEKGFPTV
EGLVALYSEGVTQKEYIIATLQAVNVCLNKAQKKHLTKPQSLEAEHGKTCDIAYDVFDCVSERIGEYCGQTP
>TcasOBP5B
ISEEMQELVNQLHSTCVAETGVSEDLINKVNSDKVMIDDEKLKCYIKCLLTETGCISDDGVVDVEATIALLPEDMKAKTTPVIRSC
GAKMGANPCESAWLTHKCYLETSPADYVLI
>TcasOBP5C
ISEEMQELANTLHATCVDETGVSEDAIESARKGNFAPDDKLKCYMKCIMEQMACIDDEGIIDVEATIAVLPEEYQAKAEPIVRKC
GTKIGANACDNAFLTNKCWYEEDPEDYFLV
>TcasOBP5D
IEMDDDMKELINNLHNTCTGETGATDDQIENARKGNFAEDDSFKCYFKCVFDQMGCMTDDGKVDSEAVIAVMPPELADKIAST
VRGCTEVGANPCETAWLANKCYQKSNPDMYFVP
>TcasOBP5E
YEFNDPLFNQILANELVELESSAYPHHRSRRDEDAVTEKCRPFRKKKLCCAEETFDELHDKDRDFKRECFKQVVGSKDGPREFDP
FRCDKVDKHRRDMTCVSQCVGQKKDVLDKDGNVKEAEFGEFVKETMAKESWFVSIQDKVVSTCLAEARNATANRDTSDTESC
NPAGVKLMHCMFREIQLGCPTEQIKDQKACARARDKIKRHNEFLPPPPQFLNDE
>TcasOBP5F
EIVVPDDLKDYINELHDHCLKEMGLTEGDHKNYNIHVKDPKMMCYMKCLMTTSKWMNMDESIQYDFILSSVHPAVKNILLPAL
DKCRDIPKGTMECEKAYNFNMCLFNADPENWFFI
>TcasOBP5G
KVDIPPDLQAEIDGYYDICYKQIGLTKDDLKAYKIGDRDPKIMCFMKCVFVEAKWMDENENLQYDYIKNTIHHSIRHITLPELEN
CGKKAEGDKCEKSFSFFNCMNKAEPEDWVLIQ
>TcasOBP5H
KVEIPPDLEAEIDEYFEQCFEPNGVTMDDIKAYKMGDKDPKIMCFMRCLFVSGKWMDENENMQYDYIKETIHHAIRHITIPELEN
CGKEAQTGDKCEKSFNFFMCMNRAEPEDWILDYKS
>TcasOBP6A
RSFSHDELDTDLSFIKTCNRTSPISMRTMNEVLINKKLGHGESSAFKCFLHCLFMKYGWMDSDGGFLLHDIKQTLEESDVEIASLE
FILYKCTATESNNRCERAFVFTQCFWDKMAEQQPSEDQFFYNIEDKK
>TcasOBP6B
AILEDSELMKVVENCVKKTNANESEFSSPNFLETTPSQPALCTAKCLLESLEIVNSEGNINMETLKEYAQPFESPAREAVATCGEEI
KSVTTCDDMEKYRKCVEPLIKNS
>TcasOBP6C
YFFMSQKFAEVREECLSENSMTMDELHEGWKMENLPESHLCFLKCLLEKREVIDENGVPQKEKIDEILTVKQLSDEKREEISTCIT
NVEKIENCETMSEIMRCFPKKRRD
>TcasOBP6D
LDVEKIRNELMADKNFVELRNKCLDKLGLKEEDLRDLKFDGDVSEDLMCFGKCIQEEDGLLDSEGNLNEEKLEKKIETMPFLSR
VSDDTKNNIMECLKEIGKIETCQDFGKQRDCIHKYV
>TcasOBP6E
EKESEEALQQIFTELDGPAAELRDQCLEKNSMKVTDLKTYNTSNDIPEKELCFYKCFYEGVEFIDANGNLNVNNMKEIPAISELG

DEVLNEITACVEKIGKIRCCGDLRKIEQCYQNITM
>TcasOBP6F
QKKGKYWTTISECLTEHSMGVEDMKKFDLPAEKMSEEMLCFNKCFYDKLLITDENGEINTDNLMSIPLVNAIDASKHDDLVTCL
KKVGKIEECDGVKKIEQCFVEFI
>TcasOBP6G
ENEHEILEVRALCMNETGVSEETARNYKPAEDPASEEILCMVKCIFEKIGCLKDDGSFCVDTMKKKNYIMDVINEENEEKIYECL
RGVGKITNCRDMAAVEECFVKNDSK
>TcasOBP7C
LENNNGNKLGDVLKNHTKSIYNSTKNQNEDENIMKQETHKLLSECGVESDNSYDDDDSYGTRRGNKRRGNNYDNFGNRKRPN
RRYSGNESNNYNRYGNRNRGNRYSGNDDENNSNESTNRGNRRNNGNRNRYNNENYSDEEDSNESNQSNNQRRYNRTMSNTG
YYYGDGGYHNECNDNHGYRNQWRPFGGNVGYDYGNMRRGYGRNLDMNLRAKRSNDNDDSQCVSQCVFGYLEVLDDNRVP
SETLVIKWLQDHLSNDMKRIRALREARRCFARLSTSDTEDGCEFSQSLSKCLNLELE
>TcasOBP7D
EDDDRQETIRQYRDDCIAETKVDPALIDRADNGDFTDDAKLQCFSKCFYQKAGFVSETGDLLFDVIKDKIPKEANREKALAIIDK
CKELKGADSCETVYLVHKCYFLHSYGTDKKTE
>TcasOBP8A
ENLDMFDPAGLQACMKKLSVGETELAKALEDKSKDPPEKIMCLFKCALEDSGFLQDGVVDKSKWPMPECVQDVVKITNCNDM
VALKHCFD
>TcasOBP8B
LDCGIHINKNDALKATINKCLISNKTLEDLWDMAPMSSESDSSSEEVPPVDGKMLQNFRIKRASVRLTNTETNETTPEPKAVSSEA
QATENCIIQCIFDNLQMTDSTGYPVHTKILDGLLKNTTNRELRDFLQDTTDECFQVMDKEDTMDPCSYSNKLVTCLAEKGRSNC
ADWPVGELPFKP
>TcasOBP9A
AMSEAQLKAAVKLVRNMCQPKSKATNEDIEKMHHGDWNIDRTAMCYMHCALNSNKLITKENVFNRDYAITLAEKNLPTALKTA
SIEAANLCKDSAKTLDDKCVAAYEISKCLYESNPEKYFLP
>TcasOBP9B
AMSEAQMKAALKLVRNVCQPKTKATNEQIEAMHTGNWDLDKNGKCYMWCILNMYKLIGKDNSFDWEAGIATLKAQAPESVR
DPAIASVNNCKDAVKTTSDKCEAAYEIAHCMYLDNPEKYFLP
>TcasOBP2A
EENDINEIRSVEENCQKQTGVSVEKVNNFELVDDPLVKENALCILKAYGIMDEDGNIYEDKLKEQITSELGEKNAEQVAKKCTIK
KESPQETAHESLWCVGEQKPIPGASPDEKN
>TcasOBP3A
QPEDRHQIALQCIDIVGIDQKVVEDAINIEIPKNNPKYKEFLACSYKKQGYQNENGEILMENIKKFLQKFYHPSDLQELNSCSGHN
GTNHAENAYQALQCIYNRLSNMTVVGN
>TcasOBP4G
EEDNVGKIESVEKKCQEKTGVSEESLQKIMRLEEVDDPLVKENALCTLKAYGVMDDDGNIFPDKFEEKLKPEIGADEAKRVAEK
CAVKKDSPEETAHQTLWCATEENALTDTSQEQ
>TcasOBP4H
DEDNLNTENVQSIEEDCQKETGVSDESLQELSETGDSDDPLVKKNALCILKAYGVIDDQGEISEDKLEEKLEPDRGKEEAEKVAK
SCAVKKDSPEETAHEALLCMQQKSQK
>TcasOBP4I
TPSLDDFKKVQKDCQKKTGVSDESINKVNNLEPVCDDLLLQENALCILKTYEVMDEEGKICPDKLMEVLEPKFGKEKAEKLIEK
CTLEKDTPQLLAHATLFCLSVQKYVV
>TcasOBP7A

LENEGQNPDTANCVALGGQRIKDSEIAKMAHCILTKTNLMTDKGTFNSNLLKERLRQSVHSDELVDKVVMMCTVEKETPLKSA
FSGYKCLRYLVPWFPLD
>TcasOBP7B
QHLTEEQKNNWRKWSNECKVLIGVSQEAINKIRNNEFDSVDDKIKKHGLCFAKKASLADSSGNIIINQIKIKLKRVIEDDEEVDRI
VTKCTIRKNTPEETTFETFRCLRENSSKFVPV
>TcasOBP7E
LTDEQKEKIKNYHKECSAVSGVSQDVITKARKGEFIEDPKFKEHLFCFSKKAGFQNEAGDFQEEVIRKKLNAELNDLDATNKLIA
KCAVKKDSPQQTAFETIKCYYENTPTHVSLA
>TcasOBP7F
LTDEQKSKLEEYSKECLKESKVDESVLKEAEKGVYLDDPKLMNHVYCLVKKINSQKDKGELEVTQIKEKLMMQINDEKEVDKL
IQLCLVQEKSARYSLGKCEVSS
>TcasOBP7G
FNNPEDELRRSAACLEQSKVSSESIKNLQIGNFDDDERLKEYLFCVSKNAGYQDPAGHLQHEMIRLRFKGGRYSDDTINEVLQQC
GHQKDTPQETAFQFMKCAYQNAFPRNYK
>TcasOBP7H
LTKEQIDKLEPISKECRELNGISEDTILKVRRGEAVNEPKLKNHVLCVSKKTGLASETGETNVEVLRTKLRKVSENDDEVNSIIQK
CVVKKSTPEETAFEIFVCLRKVKPNFSPAN
>TcasOBP7I
LSEQQTEKLNQLSKECRALTGVSQETITNARNGNFEEDPKLKLQVLCIGKKVGIMNESSQIDENVLKAKLRKVSDNDEEVNKIYN
KCAVKKPAPEETAFETIKCVMKNKPKFSPVE
>TcasOBP7J
LTKEQKEKLDKISKECKNQSGVSQELIDKARTGELINDPKLKAQIYCVSKKAGLATEAGEINMDNLKTKLKKVAANDDEVNKIIQ
KCVVKKPTPEETAFEVYKCLHANKPNFSVVD
>TcasOBP7K
TRFFNHDEIQKLECFPDIGGGLKAELWPNVVSCVFHRKGFTDDKGEFKIDVLKQKLSKFQDDKYLVNEIAELCVDKHDFTMTAA
MKSALCLNKHAPWFSPYQD
>TcasOBP7L
KQDFHKKCLASSGANADTIAKVRNGKFSNDPQTQKYFGCMLRSVGVVNQAGQLQVAALRKQVPKDMKRDEAMKIYMSCKD
KKGANNDETAYLLYKCFWEASPRHVKIDGQ
>TcasOBP7M
FSLSNREQAIFLSTYSTCLETSKVDSERALRTASGIIDDEPKLKEFLFCINKQNGVQDDAGNFVKDAVRKRIEHPLLTDKTMEIIVN
KCTRKRETGEETAYQFLKCSYFTIMNEKHQ
>TcasOBP9C
DESVYLSNHEACVKLSGVDETLLETIYEGDVFEDMKFKTYIHCFFKKSGFQDENGVMHFDAIKSSFHKDFSQTENIDKTITECEE
KKLNGESALETAFLHFKCFMGEL
>TcasOBP10A
KKCFLAEDTDKLEVMINECKTKTGVPDDILQKARNGEKIDDPKLREHALCMMKKSEMMNDAGEMQMDKIRARIKHAVSNEAE
GTRIMNECAVKKDTPLATAYEMICCLIRNKNSVDE
>TcasOBP10B
ETAKEKLRKYSDECKSVSGVSEELLNKVRNHEDVHDPKLDEHGFCILKKAGFMNEAGDILADTIKTKLKENSEHPDTVDALVEK
CNEKKDTPQHTASHLFTCLVDKKVHSH
>TcasOBP10C
RKEWFDKDPQDVAKWQKECFEASGVSMESMNKLPNITLSEDPKLGENAFCLLKKLGFISEDGTLLIEKLRTSLKNQWGDEIANK
LVNECARQKSTPQETAHEMFLCIPAKLK

>TcasOBP10D
KQQKQDTLDEEKEKMKKWTQECIQESGVTSEILQQLRNQKRVEDPKLKEYTFCTFKKNGFMNEDGKLQYDVIKSTLMKVSGSEEEANKVVKDCVVEKSTPQDTAFETVDCWYRYKKN
>CbowOBP1
ELTEKQMKATKKLIRNTCQNKAKATTEELDAMVKGNFNQGKNAQCYQLCILNTYKLLKSDNTFDWQAGVNALKANAPERIAGPGSASIKNCKDALKTKDDKCKGATEIAQCIYEDNPENYFLP
>CbowOBP2
LNEKQMKAAVKMVRNVCQPKFKATDVDIDKMHKGDWNIDHTAMCYMHCAMNMYKLMNTDNSFNYQSALAQLNQLPDSYKKATEICMEQCKDSAVTLSDKCISAYELAKCMYFCNPEKYFLP
>CbowOBP3
MRAAKGDYQDDMKLKKQILCFNKKVGLQDENGDIVLDVAKSKLFDIVKDEKKTMDILKKCAVKKDTPENTAFESAKCLHKLAPEEKLVI
>CbowOBP4
MKHREEIGLECLRQVNIQRDTIENAKATLNFPEDRKYKDFLACSYKKQGFQSQDGVILYNSIKDFLSRYYKRNDLKVMDNCKENIREDHGEMALNALRCIMDNLKNMEEKSRR
>CbowOBP5
KPLSEEGREKAQKINEECAKESGIEEDNLEKILADEFPEDDKMKEHSFCFLTTLGVMDKDGKIDKDVMTDTLKLFAPEGKEVEIMEKCAVETDDAKETAFAIGKCVHEQVKSV
>CbowOBP6
GQLPEDEKERLRQVHLSCQADSKTYCDEDLLRKLGDNVNNPQVGIHMLCMSVKAGLQERNGDLNRSFIKSRIALVTEQAKVDGYVQKCAVKKETPEKTAAMLWLCFVQNGINYYHKL
>CbowOBP7
LSEEMQELADMLHATCVHETGARQDDIENARKGIFAEDEKFKCYIKCIMAQMACIDEDGIIDEDATIAVLPEEYRNQAEPVIKKCGTKKGSNPCENAWLTHKCYQNEAPEDYFLV
>CbowOBP8
VGKFPDGRPYPDGFEDCLKSSNAKLEEVLNKPKANISEEVYCFFKCLSERVGFIDQQGNVHIDKMDVTQIFQGAVEEVPDELKSCLGGVNKVESCQDMSKICECFLKMAP
>CbowOBP9
VRNTAGKITQNESAKKTLGNCKTETGATMADIESLKEKKIPKTKTGRCFMECLFSKAKIMDNGRFNKKGMVVAFTPALKGDLTKMGKLRELSEVCEKEIGLNKLENCEGGKKIVECVAKHGKSYGMSFSTTK
>CbowOBP10
EKKLPIEAEECLKITNTDLKDMMAHPKEMSESHYCFFKCIFEKRGIINKVDGTVVPDVLDDIKDVAVLQVASEEKLAELKKCMADVEKIEKCTDMENFRVCFDKLMS
>CbowOBP11
YITEEGWGEPLIALANSLHNKCVPITGVTQASIDQVKEGNFIEDEKMKRYVLCLWLVSEVISEKFELNTEIFKLLPKKLQDGHNIIGCTKKINGTDVSELYEKTYSVTKCIQKANPDEFIMF
>CbowOBP12
LECGIAKANRNEIRQALSMCVKNNDTLEDILEMSSLSSSTTSSPTEDSDDEDSQEDTIKSTSSTTKSPRRIKSSRIKRARSFSNTKQYASKATERNREESNNSNNTNNKIDNLKDAQKDDSEDDNEVSQETPKKRQDMSENCIVHCVLEHLNLTDETGLPDHSKLSEELLKTASGRELRNFLQESTDECFQEVNEENDLDSCSYTTKLITCLADKGKSNCADWPAGALPF
>CbowOBP13
GVTEEQKKKIESYHKECSKQTGIDEDLVNKARNGQYTDTQILKDYLFCTSKLAGFINDNNELQKDVILQKTSVSTKDSAAAQKMFEACAVPQKNGPETSYHVLKCYYEKSGLSLV
>CbowOBP14

APSDSSNSTIKDYCIKEVEISEEKVNKFEKNPDDTPDEDIMCYTHCILVTLGVVDDDGKIIIEKFTKIFDKYDMECVKKIPKILECTD
LINLNKCAATDE
>CbowOBP15
EETEKEKMKRIHEECQSDPATKVEESVLKAAEEGDVDVTKIGPHTLCMNVKVGLQKENGDIVKDELRAGLRRVPGVDESKIESI
VEECGQREGGTAEEAAIKLFQCLQKRSKITHHHHHHE
>CbowOBP16
YLSEEDYGPKLSAVANKVHNACIKKHAVNEDTIMQVRKGNFVEDELIKKYISCIWLLSTVLDESGNLNIKIINDLCPPKGKDTLPK
IYHDCHAENAGVSQLDEKVYNIMKCWYEKDPELFFVL
>CbowOBP17
IELPSELQEYVEDLHKICVTKSGISEDDHAAYDVKTNPHDPKLQCYMKCLMLEAKWMNPQGDIQYDFIIDTSHPQIKDLLVAAIN
KCRAIDNGANLCEKASNFNFCMYDADPVNWFLI
>CbowOBP18
GKNQTNNCDIPASAPKRIEETINSCQEEIKLAILTEALEALNVNEHIHSRAKRSAFSKDEKRIAGCLLQCVYRKMNAVNERGFPTA
EGLISLYTEGIRHKDYVLATMQAVNHCLYHVQKNHLTTPQSIDEHGKTCDIAYDVFDCVSEEIGKYCGQTP
>CbowOBP19
TDREDKMKIHNVCVAETGVSQEFIDKMIAGEFCDDTNFKNYLVCFLKNDGVFLDNGELKADGANRQIREFADDEDTVSGFMAN
CAVQMATVEESAFHYSKCMYNTLYG
>CbowOBP20
LTEEQQQIMESLHAECISQTGATEDMIVNARNGDFSEDNKLKCYMKCVFEELGVLDDDGKVDIDGILAMLPDEYKDVATTVFNK
CGTQAGTDVCDAIFQTHKCYYAANSEHYFLP
>CbowOBP21
WMSDDKFGDKLEKMYRLWHDDCMKKTGAPENTVELIRSGIFPDDPRMKAYNRCLYTDVMDKNARLLPEKLDYYIYPAFGKTG
LKMYLDCEEKVKDEANYDDRVYKMQQCIYEANPDVSFNFF
>CbowOBP22
QMVSPSQLNTILQYHTECREKTKLPNSLVTGLIAGQFPNDPVLKSHLLCVHQKLGVQDADGNLRKEFISETLGAVLPASVDSKEL
LNKCAVQKSSPEDTALDLDRCLYQTVQPMRG
>CbowOBP23
LSEENMKEIEEFQKSCVAEVHTSPDVLSQIMSGDVSDDPKIKAHLLCFAKKAGVMTESGETIMDKLKQKLNQYLGSKADGFFEK
CNIEITTPEDTAFNVYKCLSEMLQEGK
>CbowOBP24
MNVHAGLQKPNGDIDKDDLRRALSEGIRDVITVDDIVDDCGQRVGSTAEEASVNLFRCIFGHSNAYVHEWKPSMLRQTSGAEGF
FTSSLVLSVILATLSVRLI
>CbowOBP25
YNFEDTEFNQILANDLEDVYSFTYSHPRSRRDDKAVEEDKCHPPRRGRPLCCAEETMRKLHDDKKEIKRACFKEITGKEKPERPD
RHHGPPFDPFSCEKIEQHRRDMMCIQQCVGEKLDYLDADGKPKPEQFEKYVEGIFEKEDYLLPLKDKIVSVCLDEAKNATEKVS
SDPCKSTGLVLEHCIFINTQLNCPEDQIKDKKMCSKFQDRLRQGFDKRSSPSPEAEDE
>CbowOBP26
KVDPKVIEEIIEEFTETVAKCSDEINPNADDIAALTEMKHIPDSHEGKCMIYCIYRSFDAVEEDGHVKFEGGMAFLSKIKESDPDMF
DKMSAIYKKCTETDYFDKDPCISSANFVSCNIKAGKEANISSDISSW
>AcorOBP15
ETLKEHGQKVMEKIIDYATSCADSLGVSPEDIKLLMEKKLPESKEGQCIPSCVNKKFGLQKADGTIDKEYRNSDMEEVKAIDEEV
YNKMNSIWDKCVLNGAEGSDECDTGIKLVSCMKEESEKVGLNKEAMGF
>AcorOBP14
LTIEEKATVTKIGKKCIEETKVDVKLVEKGERGEFADDPKLKEFVFCFLKASDIINADGYPKPDEIKVRLANDAPVSEIDDVLSQCE

SKAATPVDRAADLWKCYWKKSPVHIPLQ
>AcorOBP1
MSEEMEELAKQLHNDCVAQTGVDEAHITTVKDQKGFPDDEKFKCYLKCLMTEMAIVGDDGVVDVEAAVGVLPDEYKAKAEP
VIRKCGVKPGANPCDNVYQTHKCYYDTDPQSYMIV
>AcorOBP2P
SLQCLTETNADLTLVHKGQKGEFVDDPKVKAFVFCLLKKSQIVDDDGYPRPDVIKEKLSKDIPPDVITKVLAKCNPT
>AcorOBP5
SGMDSGVVKNIISLDTFPKPSDKYFKYLECMYFDQGYLDSDGLISYETIEDFILDFYDVDTVKQALEPCVVLQEGQNGGERAYN
AAKCLIQNLEALEKRYEKQNKNADNTT
>AcorOBP4
YLTEAQIKATQKLIRRTCKTKAKITNEEELDRLPKGNWDDVSHTSRCYLHCCLSMLKVINSDGFIDLEAGMRQSAILPPERRASSE
IAIDTCKDKGEGLTDKCDIAYEIAKCLYDFEPKFYLIP
>AcorOBP3
LNEAQMKAGIKLIKNTCRTKTKITDEQIAKMHEGVWDDADDVTKCYCHCALGMMKMQAKNGAFEYELFEKQKPMIPETIRET
LIASVDNCINAGEGLTKKCDLSYAFFKCVYLYDPEHYMFP
>AcorOBP6
DAEYEAEKQKLRQKAVGLMTECKDKVGASAEDVQALTNKQLPTTDKGFCLIECIFTNGNVMKNGKLDVQGTLQVLDTALSKN
PDAKKKTTAVLQTCEKEVGAGGANGCETAKLIAECFKKEAKK
>AcorOBP7
TTKLLAIFQEHALKTGLDCLSEVDATMDDLKSIINHDMPTTRAKMCLITCIHEKFGIQDANGKMLKDQTIAFLDVLKDDPPYHNL
AKDHFLHCLETVSETDEKCTIGANLMRCIVVGGNEKGIF
>AcorOBP8
VTKLQRMIKDAIAHLGTECLSETSDATMSDVQDLVDHVRPTTRKALCLITCIHTKAGMQDEHGKLKEEGGLNFVEPLKQEDMD
YYEISKEHFINCINTVPDDAEACIVGGRFNDCIIIGGKTKGILD
>AcorOBP9
LTDEQKAKLKVVSDKCIASSGADPSSVEKGRKGEFGDDPKLKEFIFCLLKATEMLDDNADVRLDKIKAKISKDLTEAEIDTLLGK
CKPTATVPVEKAAEFWKCYWANTPKRIELV
>AcorOBP10
KEAKVDPALIDKADAGEFADTKELKCFAKCFYVKAGFITEQGELLMDVVKAKLPPEHEREKALAIIELCKDLKGADACETAYAIH
KCYFQNAHAANLHKN
>AcorOBP11
HYRGARDTTQKCIDQNSCCSGPPISNFHASDKEASQQCSKEVNFNRGSIRGPLTAEQKDQIKCIAECIGKKKGYLTADGELIKDKL
LSSMKERLQSVAWLAPKLDSMFEDCLPQNENTAKQPKKCNDVGLTVGHCIWKQIQLQCPLNEQQNPQNCKNLQEYLTTHNQFP
PAPPVKC
>AcorOBP12
ICMKKNLPINGDQIIENTSMGQQGYDSSYEDDSKPSSSEDSMSSKEHAMNSLREDSINDSMNRNGDNNLRNNTEITDDCVIRCVL
KQLGMVDPSGYPDHSKISQNLIKGAENRELKDFLQDSTDDCFQMMEQDEHMDSCSFSTQLIKCLAEKGKSNCADWPMSDVPFS
HLF
>AcorOBP13
ASGESVFLHPRVRRDDEASKCHHRHKFCCGDELMSKLHDKYRDTKRECFKQVTGKEFGGGPPFTCEELEERKKEMTCVAECAG
KKKGAVDDKGNIKEDEVKKLVAECTAELEWFKPMLDEVTTKCIAEAKAAAEKYDKKGCNPSDIKFSFCIFKEIQLNCPADQIKDQ
ERCDAMRASLKKHDHPPVH
>DponOBP25
QDFTEEQRKKIIENRQQCIEETKVNPDLIEKADLGDFAEDQALKCFTKCFYQKAGFVNDKGEVQKDVVEAKLPPQADKKRALEI

VDKCALKGKDACETVYLIHKCYFEHTHPEADEKTAKDGKSEEKKA
>DponOBP1
KNNKCDIPLSAPKRIEEVINTCQDEIKIAILSEALEAFKVNEHKVVSRAKRSAFNEDEKKIAGCLLQCVYRKLNAVNEYGFPTVEG
LVSLYTEGVTQKEYVVATRQAVTKCLENAQKTHEISTKTVEASKSCEVAYEVFDCVSLEVAKYCGQTP
>DponOBP17
LSDEMKELAQMLHNTCVAETGVNEDFIRKVNAEKIFADDENLKCYIKCLMAQMACIDDDGIIDEEATIAILPEEYQALAAPVIRA
CGTKHGANPCENAWLSHRCYAEMEPSVSG
>DponOBP32
ELDQTSLPPETKELMAALHKNCIEQIGVSEADVDQLRAANFEEDAKLKCYTRCLMAESGVMDENGAIDVEAFAEILPEDIRGNIQ
TIFRRCSLTNKDIEDQCVKAYEMVKCWHKEDPESYFMI
>DponOBP7
DLTEEQKQKIVANGKACVADTGADPELIKAARQGKFADDAKLKAFALCMSKKIGFQNEAGEIQSDVVQQKLGSAIGDNEAAKK
LVEKCLVSKGSGEETAIQSFKCYYENTPTHIAVF
>DponOBP14
ELDQTSLPPETKELMAALHKNCIEQIGVSEADVDQLRAANFEEDAKLKCYTRCLMAESGVMDENGAIDVEAFAEILPEAVRGNI
QTIFRRCSLTNKDIEDQCVKAYEMVKCWHKEDPESYFMI
>DponOBP12
AMTEAQMKAALKLIRNVCQPKNKATDAQIAAMHNGDWNQDKNGMCYMNCVLNYYKLQLPDNSFDWETGLKVVESQAPPS
MAGFIMETITGCKDAVKTRDDKCKAAVEITKCLYDQNPEKYFLP
>DponOBP5
MSDEMQELANQLHTTCIGETGAAEDAITNARNGDFSEADSFKCYIKCLLSQMAIIDDNDGTIDVDAMVAVLPEEIQEATEPIIRKC
GSIIGANPCDSAWLTHKCYYKEGPEHYFLI
>DponOBP15
HPPRGPPGPPPFLGHPDPESANECRTEVGLTSEDRETKKNGELTEKELCFIRCLGQKNGALSDAGALNIETIKNDLPDHLEDSEAVI
ACLKKVGTVTTCQHIKKVAKCYPEPKEPMDRT
>DponOBP23
LKITLPPELQEYVDDLHKLCLEKGGLTENDHQTYNINDKNEKMMCYMKCLMLESKWMKSGGEIDYDFIETQAYPEVRDLLLSA
LNKCRTIEEGADLCEKSYNFNKCMYEADPVNWFFV
>DponOBP18
LSDEMKELAQMLHNTCVAETGVNEDFIRKVNAEKIFADDENLKCYIKCLMAQMACIDDNGIIDEEATIAILPEEYQALAAPVIRA
CGTKHGANPCENAWLSHRCYAEMEPSAYMLI
>DponOBP10
RLTEKQVAAAVKLVRNMCMGKSKVNPEDIDKMHQGNWDVDYEAQCYMWCGFNMYKMLDKENHFDKKSALQQMEQLPTDL
QDYVIKCMGQCENAVTNFDDKCVVAFEYSKCLYFCDPEKYFLP
>DponOBP2
YDFQDATFNEILSSDFEDIFDTLDNTYLHPRAKRNEEAVNSDEKCRRRHHRKPKLCCGEDVLDSLQEKEKEIVRLCFKDITGGVK
ESKPDRGFGNHRNFDLFSCEAVEKRKSDMICVEQCKLQKQGLVSDDGSPKPEQISTYLKEAFTTQTWFEKVSQGIVEKCVNEAIN
ATKNPVKFYTEGNKLCSRSGIVLKHCLFNSIQLSCPAGQIKDKNACERFQERAKKGKDLFDQPPGPPPFDDNREEQI
>DponOBP21
LECGLSKISSEHFRKIASECVKDNETLNRIWELTSEASMDDESASSDEEVPITQGKEAPNLDLGSSAQKSMKMSRASRTKRSRKSF
NNESPMSQRKPSPTSTTTEQTTTIQSEENEDNADANNVEESGEVCILQCIFEKLEMTDTNGLPDHKKFAAALVESATGRETRDFLQ
DSVDECFQETEEGDFENSCEYSTKLVTCLAGRGKSNCADWPVGDLPF
>DponOBP31
GNSDDLFARIAPADVEMCGKDTGVDRKEFEDAREKRALNHSMLCFLKCAMEKVGFLKDGHLEIDQAKGSLPDKMMEPVVECF

KAVGPISTCDDIQKVEDCLPSS
>DponOBP4
MGKQAGFINEAGDVLKDVLKEKSLKLFNDPALVQKLIDQCIVKKETPQETSYHAHVCLYKNSPGHLALTQFGAISQEKKEKKVQ
IIKECAEESGVSRSAVLSARKGDFQDEPLLKQYFFCINKKSQIQNEAGEYKTDVIRKGLTELFNAEEANRIIEKCARIQDSALNTAF
QSFKCFYNEAPEITGVF
>DponOBP20
LECGLSKISSEHFRKIASECVKDNETLNRIWELTSEASMDDESASSDEEVPITQGKEAPNLDLGSSPHKSMKMSRASRTKRSRKIF
NNESPMSQRKPSPASTTTEQTTTVQSEENEDIADANNVEESGEVCLLQCIFEKLEMTDTNGLPDHKKFAAALVESATGRETRDFL
KDSVDECFQETEEGDFEDSCEYSTKLVTCLAGRGKSNCADWPVGDLPF
>DponOBP13
DRQQVVDFHRPCLDHHEIEDDDLHFALDKIKMRDDDEFYLHFFCVAKQGQLMTEDGTVNTDNFETNMKGIIDEDNMENVAAIV
RLCLIQKDTVLQTIRNAVDCFMGKDHKL
>DponOBP3
DAEINQSTFEAGRNRIMEMSRTCDENPATAVDQKALENYLESNGPAPANAGVHALCITKNLGWQNEDGSVNKPLITEKVKAIFGS
VDAKIERYIEDCTEAKAKPEDTAEQLLNCYRKHSPKTE
>DponOBP8
LDQAWRDHMKEKLTEFGLECAESEQATSEDIEALHNHKPPVTHAGRCVIFCVSKKLNLMNADGTLNVTPQSDWIEKVKETDSE
AFEKMKTVYHHCADTVEVEADACDTSLSYAHCIKEEGHKVGLYTVSAD
>DponOBP30
LTDKQKELLTQHYNQCVAISKVDQAVLQKARAGDFANDPNLKTHIKCISEKIGFQGTDGKFRRDVIEKKLKETIPGDNAKNAKLI
ETCVVANKDPKLQAFNAFKCLYTNAKINLL
>DponOBP6
KITLPPELQEYVDDLHKLCLEKGGLTENDHQTYNINDKNEKMMCYMKCLMLESKWMKSGGEIDYDFIETQAYPEVRDLLLSAL
NKCRTIEEGADLCEKSYNFNKCMYEADPVNWFFV
>DponOBP28
AALTKEEIKERLKAAHDKCQADPQTAIDEAALKAFKDSKGKGQLPANMGPHDLCISKALKWQNADGKVNKELIKERITDNVAD
ASKVDAIVNECAVDKENEIATAENLFKCLLKHHATAVHGH
>DponOBP26
NPVRTHKLLSKSELHEIATSCLEEVQLSGSIVYNILKTEIFPRDNNKYRDFLACSYKKQGFLSEDGTKLLYDNLFHFISHFYGPTEV
QALKHCNLIRREDPGFLCFDTMKCIIDALKQLEFDANADIGIETNQVV
>DponOBP22
DQREKAVEFQRGCMEAHGLLEDELHEIMDGKPIQNEAFYFHFFCVVKKAKLISDNGIVNTDHFEENLKDVIDEEHMAHVAALTR
KCLIQRDDIFTTIKMAIDCFYSSEHKL
>DponOBP11
HDPHGLDSVHKECHNEVASQHYLCMAKGLHLVTPEGKVNVNGVKTHAGHVVSESAKIDQIAKECAVDHASTEETVNHLFKCL
EEKHVLSLAGHVAPQHHH
>DponOBP29
DLTEEQKQKIVANGKACVADTGADPELIKAARQGKFADDAKLKAFALCMSKKIGFQNEAGEIQSDVVQQKLGSAIGDNEAAKK
LVEKCLVSKGSGEETAIQSFKCYYENTPTHIAVF
>DponOBP19
DFDFSNYKEFEHLAGDQREKAISIFKECMAETGATHEMMEKSVEGDIPDDIVFKNHLVCIGKKSGFIDENGLHSKEKLKEKLTLLL
GDEGLVDKILDKCFMEKGTPQDTAFELAKCCHKEYHN
>DponOBP9
KKNKSNDEEKPKSYKKVFKECQKKDETRVDASIIRKLKKHKQVDLPANFGDHKLCVFKGIGLLKADNTVDEDKLKKKISSAKP

QKDNVDSIFTECKSSKSTLQETALNLDRCLTTNSIEF

>DponOBP16

YVPNVNNKIRDFCIDDSGVSIEMVENLLANPEKQLIDVESCYLHCIFTEMGLLSENGNVEVEKFKSLKASEAPYIDLTCLEEIKSIDHCSEMMILRACHV

>DponOBP27

FLTVPKCLISTGARIKDLHNLATGDSLPESSRCFVKCVGEESGLILDGTLHSEHFEALPMVSRLKADVFVDARRCIESVQGIKIESCKDIDNLNDCMKIVYRQKYSDSK

>ItypOBP10

YDFSDSIFNDHLNQIYYTLDNWQHERIRRNAEDVELKCRKPPPPMPKPCCAQDSFRDLMDKEREVLRDCFKEVVGEEHHPGRSNHPNKFDMFSCEAVEKRKNDIICIKQCLGSKLGLVNKDGKLDQAQIGNYVKSTFKNEAWLSPLADQIIGKCLVEAESVAPPKFHIEKLKPCKPSVITFKHCLDREIQLNCPADQIHNQESCERFRNHLNHKNDFDEDQPMMGPPDDD

>ItypOBP11

STGDSTMKFILLVIVVGQMGCVFGAMTESQMKAAFKLIRNVCQPKNKATDAQIEAMHKGDWNQNKNGMCYMNCVLNYYKLQLPDNSFDW

>ItypOBP12

LQKTNNKCEIPTAAPKKIEDVINTCQDEIKIAILSEALEALNINEHKVSRKRRSTFNDDEKKIAGCLLQCVYRKMNAVNQYGFPTVDGLVSLYTEGITQKEYVLATLQSVTKCLGKAQKTYDIPAQNGTASTACDVAYGVFDCVSEEVAKYCGQTP

>ItypOBP13

KVEMLTDKNEIIKCAMECMQAEIDRLTEERNEHLKKLFESHNAQISETKKKQWCYNCEQDAIYHCCWNTAYCSQTCQQQHWQAEHKKVCRRKRQT

>ItypOBP14

QDFTEEQRKKIIQNRQDCIQETKVNPELIEKADQGEFIDDQALKCFTKCFYLKAGFVNDEGEVQKDVVEAKLPPQADKKKALEIVDKCAVKGKDACETVYLIHKCYFEHTHPDLPAKAEEEKKA

>ItypOBP15

MIQHHRTNSDLTAVKMKILAVLFVICVLFQFTIARNGGNLHYSKISMKKVQKRCQKNEESRIDPDVLKKLRKGEEVVQLPDNFPDHVTCLMKGMEYLNDDNTVNEEKVRNMVQRRVTDDQDVDAIVGECKAVKTALKETALNLINCLRKHELLWNHNFHD

>ItypOBP2

YNFQDEDFXSAVVVRDGRIVDSIDSGPVHPRVRRDQEAATVAEEKCPKRHRRPKLCCAEETLDALHAKKKEITKACFKEVTGLEKQDRHDHGPHFKRFDLFNCKEVEKRKSDMICIDQCVGQKKGLLDDSGAPIRDQLIQHLKQHFSNESWFDQTVVEKITSNCLAAAKNATETPIKFSTEGLKACNPSGITLKHCLFREIQLSCPADQIKDKTACDRFQDRIQKEIEIDDLRLAPDDQQ

>ItypOBP3

RMTEKQLAAAVKLVRNMCLSKEKAKLEEVDKMHEGNWDIDHKTQCYMWCVLSQYKLIGKPNHFDRESANIQVDTLLPESMHDYVVGCLDKCENAATNFDDKCVAAYEYAKCLYFCNPKEYFLP

>ItypOBP4

DLTQQQKDKLLADGKACVAETGVSTDLIQAARQGKFTEDDKLKAFSFCMSKRLGFQNDAGDIQTEVVKQKLGGALGDLGVAAQLVTKCLVPKATPQETAFESFRCYYQNTPTHLTVF

>ItypOBP5

MRQPGGNNKNTQQDYEMWTPSTGYQPSGSNNDFNVRTRYDGNTRFNRPSSEECRDQGNGNIPRSPFGSSNLPRRQRSSYFNREDDDNDNDCISQCVLGYMQLLDTDRSPSETLIIKWLQEHVTRNEMDRIKALRDTRKCFGKLVTTDIEDGCEYAKELSKCLELDLE

>ItypOBP6

EEVTTSTKRQLTREKKKKIGKTCMLETGVRIETILRAIKEDIPKNDEKYKSYLVCSYKKQGYLSEDGGTMLYDNLYSFLQESAGYAKEDLHYIDDCKTITAETPGDLCLKKLVGILDGLHKVEKNREIDTNTIES

>ItypOBP7

QTDKQKELLAQHYKECLAKSKVNEATLQKARIGQFADDDKLKEHILCVAQKIGFQNSAGQFQNQVIETKLREALKGDAAKTKK

LISDCAITNPDPKLQAFNAFKCVYQKASINLL
>ItypOBP8
EVSKEELEKLKEIHDTCLTESGVDQSMPEKAFKGEFTDDPKFKEHLLCFHKK
>ItypOBP9
EKKCNSSNCMYDRMLETVGKEFIEQCFKETGVTPEDIRSVMEQNGYGEKQIVFPKMLDKENWYFGKRWSNQYRLY
>AquaOBP1
AGASSGIDDECVKIANLTLEDVEGFRMDVEPNQNQYCFFKCMMEKMGRIKPDGTVDLDAVNKCPKFSKFSDSDKKTIENCLGR
LENLVECEDMKRFRKCFEGFGAPNE
>AquaOBPC1
QKPDGASESIKACLSETGVPMELMQNMEHNGEVSDDPAFKEYLFCLGKKQGVMTDSGETNKDIFKSMVRYTLGKNVKDDESIE
KCLIKMETPQESAFQSMKCVMKLLME
>AquaOBP2
ESPPPVFIKCIEETNVNPEEFFPMKKVAELEEPMLCFFKCVIEKLEILKSDGTLDLNSLEKCPVFTKFSDDDKKKVKTCLGGIEKIN
DCTDMKYYLDCLMMTIKP
>AquaOBPC2
LDKATTRVKNVHEACQADPATHADEYLIEKARSEPVDPIVVGPHTLCMNVHAGLQKENGDIDKEDLRRALSEGIHDEAKVDAIV
KDCGVRDGRTPEEASISLFRCIFGHENAYVHEWKPPMIIRSSSTGSAASSLGVSMITFFVYSVLFV
>AquaOBP3
QMSEKQIKATKKLIRNTCQNKSKATTEELDAMLTGNFDQSKNAQCYQFCILNTYKLLKKDNSFDWEAGINALKANAPERIAGPG
SVSIKNCKDAIKTTSDKCKGSMEIAECIYKDNPDNYFLP
>AquaOBPC3
DESPQQRLERTHSECQSDPATVVKDSVLEEAGKGNVDVEAIGPHTLCMNVKLGMQKPNGDIDKDELRQAVGKLPDVDAATIDKI
VVECGKRDGGTAHEAAVALYECYAKLTSQNHSKH
>AquaOBP4
MNEKQMEAALKMVKNVCKPKTKATDADIDKMHKGDWNIDHTAMCFIFCALNMYKLMNTDNTLNYESALIQLKQLPDSFREP
TRQCMENCKDAAVTLGDKCIAAYELAKCMYICNPEKFFLH
>AquaOBPC4
HHVDPGTNERFEEIAILSLQTSFHRHAQHVDSYLGIFGIVAKLPKQIFVAVGLGIRLTGEMDLFQFFFLVLRKVMSHSSYDNAHEN
QHSLHLQAKFHDLVTETTL
>AquaOBP5
QQIVSFTTEDLNNDLRYIKTCNRTSPISMSTINELLINKKLVNGESSAFKCFLHCLFTKYGWMDEEGGFLLHVIKVSLEEADVEIAS
LEFILYICTAIESADSCERSFLFTQCFWNKMDEVGIMVNCDIATNSIKRKSDLYNLKSAATPFQLIETLRKQVLAKEDELVKAEQIN
KNFERMIQLVNILGQVDSFLTDRTKTMIKKIAMLADADDGKYEQEYFGHSKNNLKKK
>AquaOBPC5
DYGDEFLALTKKWHNKCIAITGVTQAMIDELKNGTFLDNEKVKRYTLCLWLVSEVMNPDYTLNEALLTKLMPKKVVEGVASYL
ACAKSAKESGIAEPHENIWGLVKCIYNRDPATFIMF
>AquaOBP6
KLDRKLIGELMGMVTKAAAKCADEVKPSSDDISKLLEHKIPDSHAGKCMVLCVNKELGFEKDDGSVDFENGKVIMDKIEQSDP
ELFQKLYDIYKKCEASDYMDADDPCQTSANLAACGIKGAEEVGIPFDISSM
>AquaOBPC6
FLERKDYGPIFTKVVDECVDICIDVTGARDEDIKQVAIGNFVDDEKVKRYIYCLWRVSRVMNSNLEVNRTYLDYILPKKEVEDNF
DDMESTCLADAKKKYGNEEHYEIIYKFEECLFKKNPEDFIMPGKST
>AquaOBP7
SSVGVRINNGNFVQNLSPKKTFGSCKTEAGATMADIEALKARTIPKTKTGRCFMQCLFNKARIMDDGKFDKNGMVVAFTPALK

GDLTKIGKLKELSEVCEKEIGLDKHVNCEGGKKVVDCIAKYGNSYGLSLTNSRTM
>AquaOBPC7
APSIDPYADDMKEREKMGLDCLKDVNIDRQVIDRAVATLSFPRDDEKYKEFLACSYKKQGYQTDDGVIQFDHIKDFLSRFYKRS
DLKLIDNCRSDIEKKSWR
>AquaOBP8
AKDSSAKFEVRTHDDAIKAHEECREENSVPDEIYEQFLEYTFPDHKHTNCYVKCFVEKMGLFTVRKGFNEANIVSQFVQDNQSF
RSTIQHGLEKCIDHNEWESDVCTWANRVFSCWLKINRHVVRKSLGDGKDD
>PstrOBP1
MREKCHRETGVDIEHVERTVEGYFHESEVLGCYFSCILNSFDLLDHDGHIDFDKLVIRLKGAESFREHGMEMVAACRGTTGKNP
CDSAFKVFQCFQKTNPAKYFVI
>PstrOBP2
PTIRTKIALAVEPSKVEGVVQKCAKKDENPGKTANKLWACFAQNGAEYYHKL
>PstrOBP3
MQLPSVILCVLIGVSIALAQVPQDFGQLLRAHTQCQSTARISPTAININELSSGNFPNDPAFKRHLLCINKALGIQDINGNLQINAIT
RLATAMAPPGTNRNSVQEVVNRCAVQRRDEEETAYQTDSCLLQARRLYSGK
>PstrOBP4
LGIQNENGDVIVEGLKKDIERHISDPAKVKEVVDKCSLRPADASKEAAATELSRCVNKYLGIVYSHHAHHE
>PstrOBP5
NNQQMDSCLMHYGIQASQVTKELPVKKMSCLLRCRSVEKGVLHPNGLVDLHRAINDIKAKTSQHLNREIIREYQACLSQLGPVQ
ECMDMVEFMHCNKEAIKAYYKSKPNELQLIKHMVVNTNWKHQPERKPYAFRPVG
>PstrOBP6
KPVEGERHPFPNTLDQCKKELKLDPPKGTEGNNGPLMFCLFNKLGIIDDNGKIDGNTLRKVILDMNVPESTANEIVKRCAEEKSN
EENVEDESVELFKCLHRDVKL
>PstrOBP7
ISDEIKAKLMEIQKECMSESGITDVVIRQALQGDELTAEMQVNAKSHAFCFAKKLGIMNDDGTFNTEVIKNLLMAALQDAGKAD
TLVATCTKVEATPEDTAFGAFRCMFSVLKS
>PstrOBP8
EGEEEREKLNEQVKKCEEELGGPKNLLEKMEKGEDIGDETKAGKMALCFNVKMGHMDANGDVVEPEFVEHVERLTSNIALRG
KIVDECGKKNGETPEIAALNFVRCMLRLVP
>PstrOBP9
LELPPELQEYVNDLHNICIAKAGISETDHAAYDIVKNPHDPKLMCYMKCLMMEAKWMNKDGIIQYDFIIDTAHPKIKDLLVPAVN
KCRNINDGSDLCEKASNFNFCMYGADPVNWFLI
>PstrOBP10
DDDMEARLKRFQAKMEKQPGYEDCLASSGAKKEDIFTFPPSEVKEVGCFMKCLMEKSGVLGADGNINMEKALDNLKQIPPEY
QEKAKEAVTKCMEGVKVESCEDVMKIKTCMFQIKHH
>PstrOBP11
LTEEQIQLMNSLHAECQGQTGVSEDVINQAKAGEFPQDNALKCYMKCVFDEVGIIGDDGKLDIEGALAILPEEMKDVATPVVVK
CDTQAGSDICDAIFNTLKCYWDTDKRAFFLP
>PstrOBP12
MAKYLMPLTALIVASYCYVDPKDYGPALAKAAAIVHQECRIISGATEEEILQTRKGDFSGGINIKRYNACLWLHLQVANTSLHSNL
EKLKSLEPPHLKGKVAHIYANCAEKIRLTEEKDFVEMAWKSSKCSHDVDPENYIFP
>PstrOBP13
LTVEQIKLLDERRGACLQETGVDTTVLERARKGDFVDDQKLKEHILCVAMKHQFVKDGELQQQQIIKKVGLFLEDEALASELYA
KCSQDQGNILDTAFFTAQCFLKTAPIPII

>PstrOBP14
LQSDAPLDYFMEKGEFEPNSVHLKCFFDCLATDLGLKTADGNINTEALKQLAQGHTHCQEMALFASDSVL
>PstrOBP15
MKKDNTFDWEEGLKVLEANAPPSLLKTATASFKHCKDSAKSLDNKCKAAMEISKCLYDFDPPNYFFP
>PstrOBP16
DLATERQKLKTQQTECETKLGEPKNMMEKMAMGEDMGDMEKAGKMALCINMAMGHMNENGEMIMDKFKEHVNNLTENMT
ENMRKKMMDECGKKTGSTAGESAINFVRCMKNILPHPGPNDFTQN
>PstrOBP17
KPQNSAEEIIAKMAEAYQNCKQGTELTEETFKKILKENGEIDEKFKNFNVCLYNTFNMFDAAGKLDKARFESVINMVYPEKLGAI
MQECIQEGPDTKEVALMISRCIGKHVL
>PstrOBP18
RAKQQDDLQRVIKTCIKASDADEEFVKLTFDGKFQPSLEFKEYLFCFAREAGMVNLDNSMNNDMIEKMTAKILKDKRAAKEIRD
DCDNTLYDPLNTVYYTFKCFITKIKEMSSAKKRRRRS
>PstrOBP19
VMSEKQLAATKKLIRNTCTNKAGVAPEKVDNTYKGIFDFDDKPAMCYAHCVMMTYKLMKKDNTFDWEEGLKVLEANAPPSLL
KSATGAFKHCKNAAKSLDNKCKAALEISKCLYDFDPANYFLP
>PstrOBP20
SSIIDNPLGKLQAKNVLASCKDKSGATREDFETLRLRQIPETKSGKCLMECMFENAGIMKDGKFNRAGAVVVLTPALQGDLTKL
GKLKQLGQTCEDELASKKYANCEGGRKVLECLARNGKKFGVGFVSVKKE
>PstrOBP21
MKDNKFSREGFIAMMSMKFQGNAEKMKIVNEVADVCANEGDSDRCEAGAKVCKCLGETSAAKGLFA
>PstrOBP22
DRPDFVTDEILEMVASDKARCMKEHGTTESMIDAVNEGNIVNDRAITCYMY
>PstrOBP23
HSELQVMKTRQTECENSLGEPKNFMEKLVAKEDLGDMEKAKKMNLCLFIKMGLISETGEILQDKLKAHLKKLTNDENIRKRGM
EQCGKNNGNNPTEVSWNFVNCMKNLFPEVFPIDFQTSPNNVVHDK
>PstrOBP24
MPSIDVQKFRTDVRDLAKELHKTCVNKANIDEGTVQNVKKGIFQNEDKLKVYLTCVWSESTVLENGKFNTKIFNDFLPSEYKDV
SKKVADCFDKFSGEKSIEDRVLKMEKCRYETDPENYIMI
>PstrOBP25
EQGNEQQKLSEAHDACQSNPNTKLSHEAMMHMEGSNDPNLGRHMLCMNVKLGIQNENGDVNVEELKKDLERHLKDAAKVT
EVVGECGKRQAGASKEDVAIALARCLGKYLGNVHNHHGAHGEHHEGHHEGHHEGHH
>PstrOBP26
VSPPGPTDALPFEDDARSAREKCQGTKMTRLEPEIERKLFGHEPLEPHQIEYHALCLCIEMGLMKITGEVSQENVKSHLARVIRDE
AALQRQLERCIEAGPTALQTSMHLFFCLHDAMMTHHDLYSQNGIPLFGPKALDYGSPLNTAATQPNPNTIITNDTSKSTAASNSTA
ASK
>PstrOBP27
KEAEDLFMGHKEAHEECKKETGFDGEAFHKALMGGAPDDKAKQHALCYGSKIGLTDSDGKPSLDKIKKMLEDNYPDTAADLI
QKCVSANGGDDEEVAFNIAQCLAHEMDKN
>PstrOBP28
LDIPDGMATDMMKEVTGCIDKTKIDFELLLKLKEGVFEDNPKVKEFLVCLYKNLGGFNGNNELQTDFIKQKMQMVSGESKIVDE
IVKKCVIQKKTPEETAFECTKCVYNALPEEEKKSMKET
>PstrOBP29
ALPASEVRFLKQVHDSCQSNPATYCDENKLRNLLSNLDDRQVGIHMSCMAIKAGLQLPNGDLNPPTMRIKIGLAVQPSLVEGVM

EKCSKRAENAGKTANQMWACFIQNGADYYHKL
>PstrOBP30
ELRSELKHIKDRQTECEKSLGEPKNFMEKLVGKEDLGDMEKAKKMCLCIFVRMGLITETGEIVQDKLKEHLKGLTDDEEIRKRG
MEQCGK
>PstrOBP31
LTDEQQKRINEKRDECVKETGVDTTVLAEARKGNFANDAKLKQHILCVGEKHNFIKDGVVQKDVVMTQVTAILGDADLTQQLY
DKCIQDKGSLLETAFAIAQCYISNTHVAVI
>PstrOBP32
FHINDEEEISLRRIHGDCLQQYYIPSHFLTHRHEYHRIPKLKEHLACLAQNYGFMSETGHFDTSIIYNKLSNACDSEDAELIMNKC
AIELDDIFETAAKFWYCLEDEGLPYMSRFIDYYQ

CSP sequences

>BlonCSP1
EDSKYTTKYDNVDLDEIIKSERLLKNYVNCLLEKGKCTPDGSELKKVLPDALLTDCSKCSETQKKGSKKIIRHLIDNKADWYKQ
LEAKYDKEGTYKKKYDAEIKH
>BlonCSP2
DNKYTTKYDDIDIESILRSERLLQNYINCLLDKGRCTPDGAELKKNIPDALQYGCTKCSEVQRKNTKRIINFLIKEKKSSFDALEK
KYDPQRNYRKRFADEIKREGILIDV
>BlonCSP3
DDKYTTKYDDIDIEQILNSERLLNNYFECVMERGNCTPDGLELRKNIPDALQTDCSKCSDKQKNGTKKVIKYLAKNKADMFKE
LSTKYDPEGMYKAKHKDEMAKEGITI
>BlonCSP4
SVGEKTKYTTKYDDVDIEEIIKNERLLKNYVECLLEKGHCTPDGLELRKNMPDAIQTDCVKCSERQKEGSQIIMKYLIDNKPDY
WASLEEKYDPTGSYKKKYLEAKKAEVNVKPLRAEP
>BlonCSP5
QYTNKYDNVDVDKILTNTRVLTNYIKCMMEEGPCTPEGRELKKTLPEALARGCDKCNEKQKATAEKVIKHLTNKRPEDWKRLT
KKFDPEGLYKKKYEDQLKSVNTQ
>BlonCSP6
DNSGDQYTSKYDNINVDEILGNKRVLANYIKCILDEGPCTPEGREFRKYIPDAIVTNCAKCTEAQVKIIRKTSKFLITNRPGDWERI
RRKYDPDEKYKDSFNKFLKGEN
>BlonCSP7
KPGEQYTTKYDNIDLDAIIKNDRLLRGYVDCLIGKKTCTKDGEELKRVVPDALKTECSKCNETQKRGVRKMMHHLIENKRDWY
KELETIYDPQGTYKKRYEALAKKEGLRI
>BlonCSP8
RPDDHYTTKYDNIDLDAIIKNDRLLRGYVDCLLGSKPCTKDGEELKKVVPDALQTECSKCNETQKNGVRKMMHHLIENKRDW
YKELEALYDPKGTYKTRYDALAKKEGLTI
>BlonCSP9
RPGDHYTSQFDSIDLQKIMKNDRLLRHYVDCLLGKAKCPKEGEELKKVIPDALKTECSKCSETQKNGIKQMVHHLIENKHDWY
KELEAIYDPDGTYRKRYEDLAKKEGLDI
>BlonCSP10
MPMEFYATKYDHVDVEAILNNRRMVNYYTTCLLGKGPCPPDGLEFKRVLPDALMTNCAKCTEKQKTVTLRTIKRLKKEYPKIW
AQLQEKWDPEDKYVKVFEATYGDRWIESNETKPNFEIGNRFGDSDDATTTPPKTITMAPKTNTPPLVTKIMNPVTVSSVTTPSSLF
TNKVETTKKKISTKTTKTPIVTTKSTTTTTIKPASSTTRFPPITLSPVPNIGASIQATVSLSTNIVGNIFRGIGALGTRVAATGANIAQV
VVKNLSIPLS

>BlonCSP11
TNLEDILTPEGKFTSKYDNIDVDSVFRNKRLLKTYIDCLKELNCTTKSGQFLKVALADAVENDCQRCSNKQREILEKVLKMLINQ
RPDVWADLEKIYNKNGRFNDRLNRFKIQGSN
>BlonCSP12
KPAENGYSTKYDDFDIKAVLASKRLVAKYGDCIMDRGSCTPEGKFLKDILPDAIASKCSKCNEKQKKIAGMMLQHLLLYNRPLF
DEITDKYDPKGDIKKMYGIGVQNESKDYENYDEA
>BlonCSP13
CEQKYSYNTDNINVKSILENDRLLQNYFNCLLDKGRCTKEGAELKKRIPEAIQTQCSKCSEKHVNQIREVIQFLMTRKPDMWKK
LLDKYDPDRLYTKRYEEMRKKQKHHH
>BlonCSP14
DCLLGSKPCTKDGEELKKVVPDALKTECSKCNEKQKNGVRKMMNHLIKNKRDWYNELEAVYDPQGTYKKRYEELAKKEGLNI
>BlonCSP15
IPEAIQTQCSKCSEKHVNQIREVIQFLMTRKPDMWKKLLDKYDPDRLYTKRYEEMRKK
>BlonCSP16
FPQETERPAISDEQLETTLQDKRYLMRQLKCALGEAPCDPVGRRLKSLAPLVLQGNCTKCSPQEQRQIRKVLSYMQVNFPKEWN
KILNQYSG
>TcasCSP2
QLTRISDEAIESTLNDRRYLLRQLKCATGEAPCDPVGRRLKSLAPLVLRGSCPQCTPQEMKQIQKVLAFVQKNYPKEWNKILHQY
AG
>TcasCSP3A
ATYDVYPTKYDNVDIDAILHNKRLFDNYLQCLLKKGKCNEEAAILRDVIPDALITGCRKCNDHQKVSVEKVIRFLIKERNSDWQ
QLISVYDPKGEYQTQYAHYLEKI
>TcasCSP7A
EQYTTKYDNINVDEILASERLLKNYFNCIMDRGACTPDADELKRVLPDALKSDCAKCSEKQKEMTKKVIHFLSHNKQQMWKEL
TAKYDPDGIYFEKYKDKFDS
>TcasCSP7B
RPEDQYTIKYDNVNLKEILQSDRLTENYVNCLLEKKPCTPDGEELKRVLPDALKTSCAKCTDKQKQGAKTVIQHLYKNKQDWW
KQLEAKYDPEHTYVKAHEDELKAL
>TcasCSP7C
QLGLAGNNYIEKQLLCALDKAPCDALGNQIKGALPEIIGKNCERCDSRQVANARRIARYVQTKHPDVWNALVKKYSV
>TcasCSP7D
AENKYTNKYDNVDVDKILNNDRVLTNYIKCLMDEGPCTSEGRELKKTLPDALSSGCTKCNQKQKETAEKVIRHLTQKRARDWE
RLSKKYDPQGQYKKRYEEHVATSRAA
>TcasCSP7E
APAEFYESRYDHLDVESILNNRRMVNYYAACLLSKGPCPPQGVDLKRVLPEALQTNCAKCTEKQRTAAYRSIKRLKKEYPKIWE
QLRAVWDPDDVFIRKFETSFESGKPSGVISTNTSPPSPILSNRFGENEEADAASNVISSTPLPPTTSTTTRTTLTTKFTTKPSTKPTNK
PVVVTKPPQAPPFATVGANLQATVSFGTNLVGGIVRSLGTLGSRVVESGTKLANMVISAAIRP
>TcasCSP7F
KTLHRSTRDDKYTTRYDNVDVDRILHSKRLLLNYINCLLEKGPCSPEGRELKKILPDALVTNCSKCSEVQKKQAGKILTFVLLNY
RNEWNQLVAKYDPDGIYRKQYEIDDDYDYSELDSAKK
>TcasCSP7G
EDTTHKYTTKYDNIDLENVVKNERLLKSYVDCLLEKGRCSPDGLELKKNMPDAIETDCSKCSEKQKEGSDFIMRYLIDNKPDY
WKALEAKYDPDGTYKKRYFESQKDEVSKVEA
>TcasCSP7H

EEITHKYTTKYDNIDLENVVKNERLLKSYVDCLLEKGRCSPDGLELKSK
>TcasCSP7I
ASVPYETVDIDKLLADDKMVTEYMACLRGEGPCNPAEKDLEEHIPLVLGNYCADCNDKQKNFVIKLATFVIKNRFDEWRQVQK
RFDPDLSHADDFNKFILGS
>TcasCSP7J
RPQEKYTTKYDNIDLEEILKSDRLLKNYFNCLMERGTCSPDGEELKKALPDALHSGCSKCTEKQKEGSRKIIHYLIDNKRDWWN
ELEAKYDKDGVYRQKYKDVIEKEGIKL
>TcasCSP7K
ENSKYTTKYDNVDLDEIIKSDRLLKNYVNCLLEKGKCTPDGAELKRHLPDALHTECSKCSETQKNGSKKIMRHLIDHKRDWWN
ELEEKYDKEGEYRKKYEAEIKGKKD
>TcasCSP7L
ADKYTTKYDNIDLNQILKSDRLLKNYVNCLLDRGKCSPDGQELKNNLADALQTSCSKCSQRQKDGSRTIIRYLIKNKRDWWNE
LEAKYDPTGIYKNKYADELKAEGIVL
>TcasCSP7M
EEYTNQYNDELDAALKSERLMKSYFECLLGTGKCTPSGEELKKDIPDALKNECAKCNDKHKEGIRKVIHYLVKQKPEWWEQLQ
KKFDPQGIYKKRYQNYLDKEGLKA
>TcasCSP7N
EEYLVPQNIDLDEILKNDRLTRNYIDCILGKGKCTPEGEELKRDIPEALQNECAKCNEKHKEGVRKVLHHLIKNKPNWWQELEA
KFDPKGEYKQKYNKLLEKEGLQA
>TcasCSP7O
VEYLILREIDTILKNDQMTRNYLDCVLDKGKCTKEAEKLKKGITETMKNGCVKCEQKQKEDVHKVFQHLMIHRPNWWHELET
KFNPHHEIKLQHLHQSKFNPHEEVKLQHLHQFPHHDFLEREGFIR
>TcasCSP7P
QEYLVPQNIDVDEILKNDRLTRNYLDCVLGKGKCTPEGEELKKDIPEALQNGCAKCNEKHKEGVRKVIHHLIENKPNWWQELES
KFDPQGEYKKKYDELLKKEGLAN
>TcasCSP7Q
EEYTVPQNIDIDEILKNDRLTKNYLDCILEKGKCTPEGEELKKDIPDALQNECAKCNEKHKEGVRKVIRHLIKNKPSWWQELQEK
YDPKGEYKSRYNHFLEEEGLN
>TcasCSP7R
EEYVIPDNIDIDDILSNERLLKNYVNCLLDKGRCTPEGKKLKSTIPEALSTDCAKCNEKVKANVRKVLHHLIDNKPDMWKQLEA
KYDPSGEYRSKYKDELEKNGIHV
>CbowCSP1
EKYTTKYDNVDIDSILNSERLIKNYMDCLMERGPCTPEGKELRDNLPDALKTECHKCSDKQKEVSKKVLRHLVKNKRKEFDELT
GKYDPEGVYKNKYKEDLAKEGIIV
>CbowCSP2
DEKKSRANVPKYTTKYDNVDLDAIINNDRIFRNYIECCLGKRKCTPDGLELRNHIRDAMDNECDKCSETQKKAMKKVGRKLYK
EKPEWWKELCDHFDPDMKYRTKYQKFIDEALAEEDK
>CbowCSP3
RPEDKYTTKYDKVDLDAILQNERLLRSYIDCLLDKKKCSKDGEELKKILPEALKSKCAKCNENQKKGAKKVIRYLLKEKRAWW
DELEAVYDPEGIYRKTYEKELKEEGIQI
>CbowCSP4
GEKYTAKFDNIDYEEILRSERLLKNYIFCLLDKGPCSPDGLGIKNILADALETECSKCSDRQKEGSTKVIRFLIENHAGWWKELTE
KYDPDGIFMQKYRDQWNSNN
>CbowCSP5

KPAEKYTTKYDNVDLDTILKSDRLLKNYVNCLLDKGNCTPDGAELKKVLPDALQTDCSKCSDVQKRGSKKIIRYLIDNKAEWY
KELEAKYDKNGVYKKKYDKELEDAKV
>CbowCSP6
QTYNTKYDHVDIDSILANKRVLSSYIKCIMDEGPCTSEGREFRKHIPEAITNNCAKCSDAQKRIIRKTSRFIRRERPQDWDRVSRK
YDPQQKYTANFNKFLSEN
>CbowCSP7
QNSYSRKYDNVDVDKILKNERVLSNYIKCLLEEGPCTAEGRELKKTLPDALANECEKCNPNQKNTAEKVMKHLMSKRARDWE
RLSKKYDPQGNYKKRYQHLVEKVAN
>CbowCSP8
QILSRNNYIQKQLLCTLDRAPCDHLGSQIRDALPEIIGNNCKSCDQRQTANAKRIAVFVQSKYPDVWNALVKKYSRLE
>CbowCSP9
MEVSYHLIVCIVVFSYVSEDRFVSTTSLNRVERAVEKYSNKYDKFDVAGVLASARLVKRYGDCLMDRGPCPPEGRFLKDIVPDAI
ATECSKCNNIQKKQAGLILQHLLLHYRPLFLELCDKYDPTGKARKQYGIDTNEADEYEDYDEA
>CbowCSP10
YNTAFDRVNVEDVLKNKRLLKRYVDCLLGVPKTCTKDGQLLKDTLPNALKTKCEDCSEPQRKGAKRVANYLIDCKPKWWSDL
AKIYDSDGIYTKQYHDELLAEGINIDGSSKDTEHKTQCYN
>CbowCSP11
MQIKYQDAILVTVVLCAIVTIVNALPQSQAISDEALESTLKDKRYLLRQLKCALGEAPCDPVGRRLKSLAPLVIQGSCSQCTPQEQ
RQVRKVLSYMQVNFPKEWNKVLKQYSG
>CbowCSP12
MKEGKARKLSQCYSFHGHEQCKYISLKMFLPFVVLSCLITLSISAVPEKSRYTTKYDNINLEEIIHNDRLLKNYVDCLLDKGRCTP
DGLELKKNMPDAIETDCSKCSDKQKEGSEIMMRYLIDNKPEYWNPLQEKYDPSGSYKKRYLDAKKTEVSVEPIVKS
>DponCSP10
EEYTSKFDNVDLDQILSSDRLLRNYINCLLDKGKCTPDGIELKKNLPDALENECSKCTPKQRDGAKKVIRYLIENKRDYWDEVA
AKYDPEGTYYKKYQEQAKKENIKL
>DponCSP9
ETTERPAISDEALEKTLSDKRYLQRQLKCAVGEAPCDPVGRRLKSLAPLVLRGSCPQCTEQEKKQIKKVLAYVQVNFPKEWNKM
LQTYAG
>DponCSP4
QSSPYTSKYDNVDVDKILKNDRVLTNYIKCLMEEGPCTPEGRELRKTLPDALASGCSKCNEKQKSTTEKVIRHLQTRRAKDWDR
LSKKYDPEGVYKQKYTAELKTETTA
>DponCSP7
RRVKRSAQTYTTKYDNIDIDQILASNRLLKNYVNCLLDKGGCTQEGKELKKYLPDAIATECSKCSQTQKKIAGRVFQALLLNHR
DDWELLTNKYDPEGNFQKKYLQEDEDYSDLEEA
>DponCSP5
EVTEKSQYTTKYDNVDINEVVHNERLLKNYVNCLLDRGPCSPDGLELKKNMPDAIETDCSKCSDKQREGSEAMMRFLIDNKPE
YWNPLQEKYDPTGSYKKRYLDAKKAEVAIQPAEKTP
>DponCSP3
EVTEKSQYTTKYDNVDINEVVHNERLLKNYVNCLLDRGPCSPDGLELKKNMPDAIETDCSKCSDKQREGLEAMMRFLIDNKPE
YWNPLQEKYDPTGSYKKRYLDAKRAEVAIQPAEKTP
>DponCSP1
EEYTSKFDNVDLDQILSSDRLLRNYINCLLEKGKCTPDGTELKKNLPDALENECSKCTPKQRDGAKKVIRYLIENKRDYWDEVA
AKYDPEGTYYKKYQEQAKKENIKL
>DponCSP6

KPQEKYTTKYDNIDLDAIIRNDRLLRNYIDCVLGKKKCTKDGEELKVHLPDALQSDCSKCSEAQRNGSRKIITHLLKNKRGWFN
ELQAKYDPAGNYLSKYSEELRKEGIVI
>DponCSP11
QILNGNVYVEKQLLCALDRAPCDNLGRQIKDALPEIIGKNCKACDNKQLSNAKRIARFVQNKYPNVWNDLVRKYGNPTN
>DponCSP8
DTPKYTTKYDNVDLEEIIKSDRLMKNYVNCLLEKGKCTPDGAELKRVLPDALHTECSKCSDSQKKGSRKIMRHLIDNKPEWWT
ELENKYDKEGAYKKQYREELKKDGIKL
>DponCSP2
QTYTSRFDNINIDEILSNKRVLNNYVRCVLDEGPCTAEGRELRTHIPEALRTSCAKCTPSQQKFVRKGANFLIKNDPDQWKRIAKK
FDPEGKFAPQFRQFLNA
>ItypCSP1
DKYTSKYDDVDIDQILQSERLLRNYLNCLLDKGRCTPDGAELKKNLPDALENECSKCNESQXKGASKVIRYLIDNKRQYWDEL
AAKYDPEGVFFKKYEAEAKKDLLDQIGRA
>ItypCSP4
KPAVKHYASKYDHIDVETILNNPRMVKYYSACLLSQGPCPPEGVEFKRILPEALHTNCHRCTEKQATVTLRAIKRLKKEYPKIWS
QLSQMWDPDDVYVRKFESTFGNRNKIPSVVVNNGWDLGSSTTSNADEPRPDTTTHQIITSPNIMSFTTSKTSSTPITTSSTANPSTK
TSTTTVGTTTKPPSRPAPIPGLLP
>ItypCSP5
QSPYTSKYDNVDVDKILKNERVLTNYIKCLMEEGPCTPEGRELRKTLPDALASGCSKCNEKQKDTTEKVIRHLMDKRTKDWDR
LSKKYDPQGVYKQRFEKELSARKLA
>AcorCSP1
QDNSQGLFLWKYKVDVNTVISSKRLLINYINCLLDKGPCTTEANELKKILPNAISTQCKDCSITEKQAVGKIFAHLLQYHRDLWN
ELLDKYDPDGTFRKQYELDEDEDYDDEKESN
>AcorCSP2
MPQNRPQVSEEAIDRALKDTRYLMRQLKCAVGEAPCDQVGRRLKSLAPLVLRGACPQCSPGEVKQIQKVLGYVQKNYPREWN
KILQQYAG
>AcorCSP3
GDEKYTTKYDNMDIEHILSNDRLLSKYVQCLLDLAPCTVDGLELKKNMPDALETNCSKCSDTQKVSSEKIISYLIDNRPDYWTP
LQKKYDPTEEYTKKFIEARKVKAKVST
>AcorCSP4
ADPPGGYYTTRYDHLDIENILNQKRLVHYYAACLLEKGPCTPQGTEFKNILPEAIKTNCLRCTEKQRIVTTRTIKRLTKEYPDIWG
QLEQKWDPTGANVKRLLASVNRPRPISGIPSLADRFGNEDQNNLGEITTRSTTSSSTIGGGISSTTSSSVSVPSSTGSSSSSSATTTTT
TTTTTTTTTTRPPTTTFRTIYKPVTARPFNSIGPNLMILNPKVIIDKVLYTADAVLNTVSGVLKG
>AcorCSP5
QNRYTTRYDSIDVDSILSNRRILTNYLKCLMDEGPCTNEGRELKKTLPDALANGCSKCNEKQKSSAEKVIRHLIKNRSNDWKRLT
AKYDPSGQYRKKYEAQYNIKA
>AquaCSP1
LPQSSTDRPSISDDALESTLQDKRYLLRQLKCAIGEAPCDPVGRRLKSLAPLVLQGSCTQCTPQEQRQIRKVLGYMQVNFPKEWN
KILKQYSG
>AquaCSP2
MPSSPSAANVATSNVRTPTRYCVTSSITSPRNGSTCKTNTIPTRSTTTNTRVKPKSAALKSKSLNDLQQQQKKKRQQ
>AquaCSP3
VVVNCDEKNINKLLNNQVIVSRQIMCVLEKSPCDQLGRQLKAALPEVIVRNCRNCSPQQAQNAQKLTTFLQTKYPDVWAMLLR
KYKT

>AquaCSP4
DEKYTTKYDNIDYEQILQSERLLKNYVYCLLDKGPCSPDGKELKSVLHEAFETDCEKCNDHQKMALRNIIQFLIEHHKHWWEQ
LADKYDPDHSFKQKFEKELEHEKNSSGP
>AquaCSP5
HEKKNRADIPKYTTRYDNTDIDAIINNDRLFMNYMYCCIGKGKCTPDGLELKSHIRDALEHDCDKCSETQKKSMKKIGKKLYKE
KPEWWKELCDHFDPDHKYRTRYDSFIQQALAEKDD
>AquaCSP6
DQKYTNRFDNVDVDSVLGNNRILSNYIKCLMEKGPCTPEGRELKKLLPDALQSDCSKCTDIQRKNSQKVITFLRANRPGEWKIL
LDKYDPNGAYRARHHI
>AquaCSP7
VPIQHYATKYDHIDIEMILNNRRMVNYYAACLLNKGPCPPEGLEFKRILPEALRTNCMKCTEKQKTVTMRTIKRLKKEYPKVWA
QLQNEWDPDGSFTAKFEETYGDRQSGPVPSVSLQLLNRVGTDDGSSKDDGIAMSEPPSISAPITTKEVGESTNKSEKPSTKDNSIV
MKGITSTIASTSIPTTTKKAMVSTKKLENKSVKPSLVTKKVTTSTESATIVNKIITNAPSVTSTITSNYYAPPVRFRPLANIGAGIEAT
VSLGTDIVGNLVRGIGAIGDRLVQTGAEIAGVVLKSITRPL
>AquaCSP8
AVPEKTKYTTKYDNVDLEEIIKNDRLLKNYVNCLLEKGKCTPDGLELKKNMPDAIETDCSKCSEKQKEGSEIIMRYLIDNKPDY
WNPLQEKYDPSGSYKKRYLDTKKTEVNVEPIVKS
>AquaCSP9
QNTYNGKYDNIDVDKILKNERVLANYVKCLMEEGPCTPEGRELKKTLPDALKNGCDKCNPNQRGTAEKVMKHLMTKRARDW
DRLTKKYDPQGHYKKRYQEQLEKAAKSQE
>AquaCSP10
DTYNTKYDHVDVDSILANKRVLASYIRCILDEGPCTPDGREFRKHIPEAITNNCAKCSDPQKKIIRKTSRFIERERPQDWNKISKKF
DPQQKFTASFRQFLNEN
>PstrCSP1
EKYTTKYDGVDVDEILKSDRLFNNYFKCLMDKGKCTPDGSELKRVLPDALKTNCSKCSEKQKSGTEHVIKFLIDNKPEQWEAL
QNKYDPEHIYTTMFRDEAKNHGINV
>PstrCSP2
TSEVTQKSSTEKSSVSDEALEATLKDKRYLMRQLKCALGEGPCDPVGRRLKSLAPLVLQGSCSQCTEQEQRQIRKVLGYMQVNF
PKEWNKILRQYAG
>PstrCSP3
ADEVKYTTRFDGIDIDQVLHNDRILKSYINCLLKDAACSPDARELKRLLPDALA
>PstrCSP4
EKLYTTRYDNIDLDKILKNTRLLNVYIGCLMSKPRAVCNNEGTYLKAKLPEAIDNDCKLCTDRQKIGAFKAIDYLISNHAAIWREI
QMKFDPDDEFRVRNKVLLEYCHQHEDDFNDEEFLALFKRYVKPRVH
>PstrCSP5
MSPDVNDVIYSMKTFKIKVDLRLLILVHLVLLSSAALPENSKYTTKYDNVNLEEIVHSERLLKNYVDCLLDKGHCTPDGLELKK
NMPDAIETDCSKCSEKQKEGSDFIMKYLIDNKPEYWNPLQEKYDPTGSYKTRYFESKKLEVKVEPISQAS
>PstrCSP6
MFKREEARKLSDTSLTTNRNGTAIWRRFTIRTERTKRNTRRK
>PstrCSP7
KPAEDKYTTKYDNIDLDAIIKSDRLLKNYVDCLMDRKGCTKEGETLKAILPDALKTKCAKCSESQKNGAKKMIRYMLKNKKD
MWKELEAVYDPEGIYKKTFADELKAEGIEI
>PstrCSP8
QGGPRGALTGRNYMERQLLCALEKGPCDVLGNQIKGALPEIIGNNCRACDPKQRSNARKMANVIRERYPEVWNALVEKYSRA
